# Cooperative Palladium/Isothiourea Catalyzed Enantioselective Formal (3+2) Cycloaddition of Vinylcyclopropanes and α,β‐Unsaturated Esters

**DOI:** 10.1002/anie.202202621

**Published:** 2022-04-28

**Authors:** Jacqueline Bitai, Alastair J. Nimmo, Alexandra M. Z. Slawin, Andrew D. Smith

**Affiliations:** ^1^ EaStCHEM, School of Chemistry University of St Andrews St Andrews, Fife KY16 9ST UK

**Keywords:** Cooperative Catalysis, Cycloaddition, Isothiourea, Palladium Catalysis, α,β-Unsaturated Acyl Ammonium

## Abstract

A protocol for the enantioselective synthesis of substituted vinylcyclopentanes has been realised using cooperative palladium and isothiourea catalysis. Treatment of vinylcyclopropanes with Pd(PPh_3_)_4_ generates a zwitterionic π‐allyl palladium intermediate that intercepts a catalytically generated α,β‐unsaturated acyl ammonium species prepared from the corresponding α,β‐unsaturated *para*‐nitrophenyl ester and the isothiourea (*R*)‐BTM. Intermolecular formal (3+2) cycloaddition between these reactive intermediates generates functionalised cyclopentanes in generally good yields and excellent diastereo‐ and enantiocontrol (up to >95 : 5 dr, 97 : 3 er), with the use of LiCl as an additive proving essential for optimal stereocontrol. To the best of our knowledge a dual transition metal/organocatalytic process involving α,β‐unsaturated acyl ammonium intermediates has not been demonstrated previously.

## Graphical abstract

Cooperative palladium and isothiourea catalysis allows the formal (3+2) cycloaddition of vinylcyclopropanes and α,β‐unsaturated esters to generate functionalized cyclopentanes via an α,β‐unsaturated acyl ammonium intermediate with the addition of LiCl crucial to obtain high product diastereo‐ and enantioselectivity.

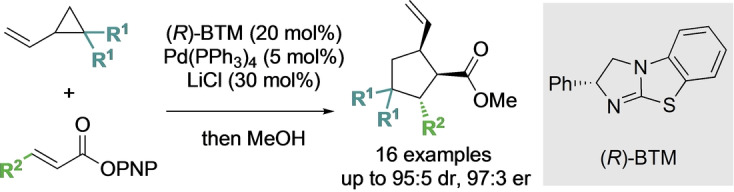

## Introduction

Catalytic transformations are essential in modern chemistry, enabling efficient and economical processes to be developed. Of particular interest are protocols that allow multiple catalytic transformations to be combined within a single synthetic sequence.^[1]^ Despite numerous potential advantages, designing a multi‐catalytic process poses several challenges, with the most profound being the compatibility of the reaction components.^[2]^ The catalysts, substrates, formed intermediates and products, as well as additional reagents must interact synergistically for an effective process. In recent years, significant developments have been made in combining transition metal catalysts with organocatalysts,^[3]^ with a range of effective protocols developed. Distinctions between these processes can be made based upon the modes of activation used to transform the substrates.^[4]^ Cooperative catalysis represents one of those modes, relying on the simultaneous activation of two separate functionalities within the substrates by the respective catalysts.^[5]^

**Figure 1:**
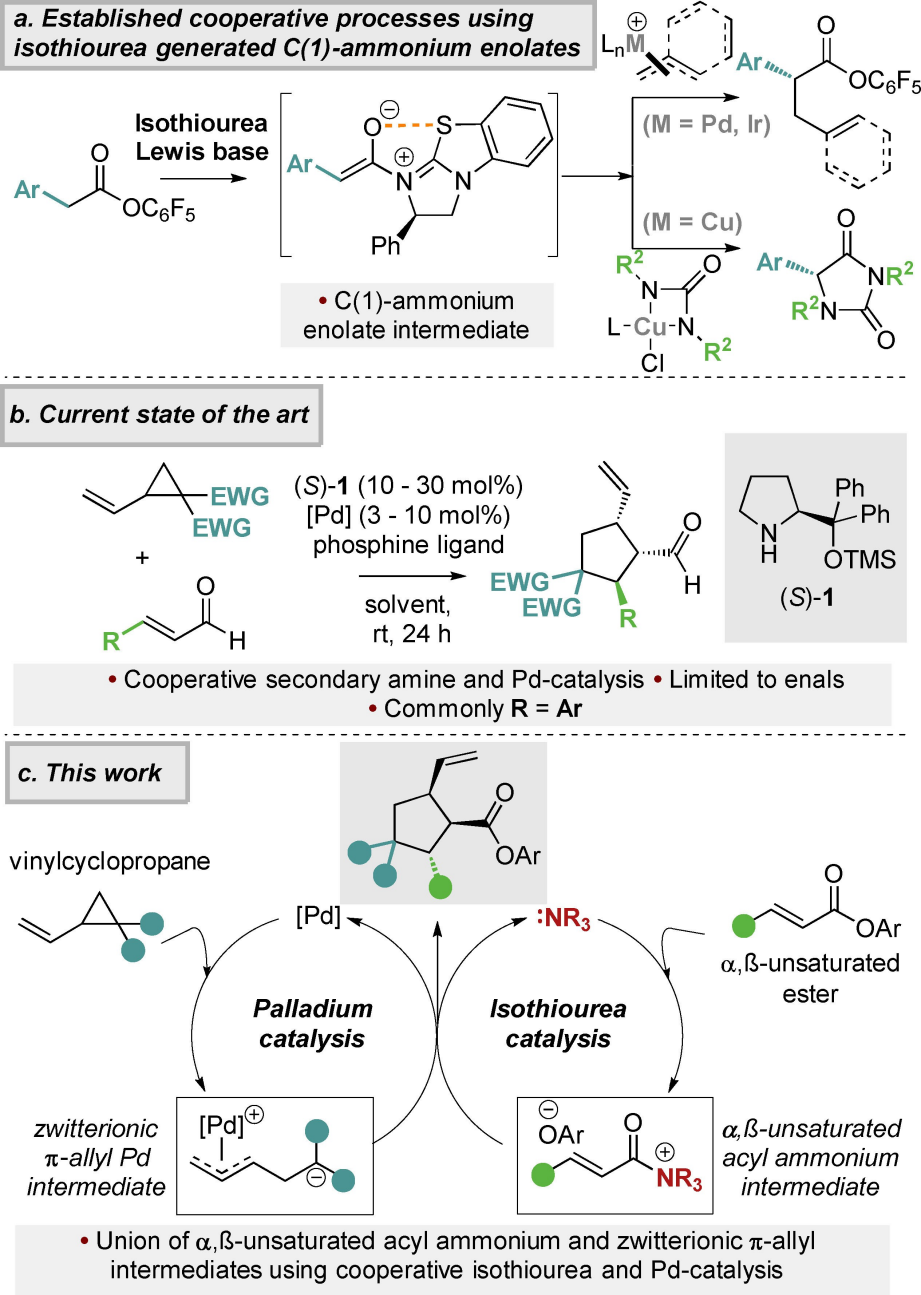
[a] Previous cooperative catalysis using isothiourea derived C(1)‐ammonium enolates. [b] State of the art cooperative catalysis using enals and vinylcyclopropanes [c] Proposed use of α,β‐unsaturated acyl ammonium intermediates in cooperative catalysis using isothioureas.

Over the last two decades, enantiopure tertiary amine Lewis bases have been shown to be effective catalysts for the synthesis of chiral building blocks.^[6]^ Within this area, isothiourea catalysts have been widely exploited through harnessing the reactivity of acyl ammonium,^[7]^ α,β‐unsaturated acyl ammonium^[8]^ and C(1)‐ammonium enolate intermediates.[^9^, ^10^] In recent years, the development of cooperative catalytic procedures using isothioureas and transition metals has become a promising area of research.^[11]^ These protocols commonly employ C(1)‐ammonium enolate intermediates, generated from electron‐deficient aryl esters, using the in situ liberated aryloxide to promote catalyst turnover.^[12]^ These catalytically generated nucleophiles have been combined with palladium‐, iridium‐ and copper‐derived electrophiles, as demonstrated by the work of Snaddon,^[13]^ Hartwig^[14]^ and Gong,^[15]^ respectively (Figure 1A).

A combination of isothiourea and palladium catalysts has also been used to promote tandem allylic amination/[2,3]‐sigmatropic rearrangements,^[16]^ while cooperative ruthenium and isothiourea catalysts have been used to facilitate the DKR of secondary alcohols.^[17]^ In these processes, catalyst deactivation through isothiourea coordination with the transition‐metal co‐catalyst was avoided successfully, but irreversible binding has been observed with gold, resulting in isolable chiral Au^I^ and Au^III^‐isothiourea complexes.^[18]^

Among the potential substrates for palladium‐catalyzed transformations, vinylcyclopropanes (VCPs) have been widely explored, particularly in formal (3+2) cycloadditions since the seminal work by Tsuji and co‐workers.^[19]^ The first catalytic enantioselective variant of this transformation was reported by Trost and co‐workers, using azlactone‐derived Michael acceptors as reaction partner and a chiral phosphine ligand to induce asymmetry.^[20]^ A range of variants have since been developed, with nitroolefins, α,β‐unsaturated keto esters and imines, activated indoles and benzofurans, and unsubstituted acrylic esters used as Michael acceptors, with the use of simple α,β‐unsaturated esters currently representing a widely accepted limitation.^[21]^ VCPs have also been employed in a dual catalytic setting as showcased by the independent reports from Vitale,^[22]^ Jørgensen,^[23]^ Wang^[24]^ and Rios,^[25]^ detailing the cooperative use of palladium and secondary amine catalysts to facilitate VCP ring opening and formal (3+2) cycloaddition with enals (Figure 1B), with the chiral organocatalyst (*S*)‐**1** responsible for enantioinduction. A range of related enantioselective processes have since been developed.^[26]^ Building upon these precedents, in this manuscript we demonstrate the feasibility of harnessing an α,β‐unsaturated acyl ammonium intermediate in combination with palladium‐catalyzed ring‐opening of vinylcyclopropanes to promote an enantioselective formal (3+2) cycloaddition of α,β‐unsaturated esters (Figure 1C). In this scenario, a zwitterionic palladium π‐allyl intermediate would be generated from the VCP, with the α,β‐unsaturated acyl ammonium intermediate prepared through N‐acylation of the isothiourea catalyst with the α,β‐unsaturated aryl ester. Intermolecular formal (3+2) cycloaddition, followed by aryloxide catalyst turnover, delivers the desired cyclopentane product. To the best of our knowledge a cooperative transition metal and organocatalytic process involving α,β‐unsaturated acyl ammonium intermediates has not been demonstrated previously.

## Results and Discussion

### Investigation of Optimal Reaction Conditions

To assess the feasibility of this process, dinitrile substituted vinylcyclopropane **2** and β‐CF_3_ substituted α,β‐unsaturated *para*‐nitrophenyl ester **3** were treated with (2*S*,3*R*)‐HyperBTM **5** and commercially available Pd(PPh_3_)_4_ in CH_2_Cl_2_ at rt (Table 1). Cyclopentane **4** was isolated in 59 % yield as an inseparable mixture of two diastereoisomers (67 : 33 dr) with low enantioselectivity (58 : 42 er, entry 1). Screening of a range of solvents (see Supporting Information for full details) indicated that DMF, THF and acetone resulted in improved stereoselectivity (entries 2–4), with acetone chosen for further investigation based on its industrial classification as a preferred solvent.^[27]^ Varying the isothiourea catalyst showed that (*R*)‐BTM **6** gave reduced diastereocontrol (entry 5), while (*S*)‐TM⋅HCl **7** in the presence of *i*‐Pr_2_NEt (20 mol %), used to generate the free base in situ, gave **4** in improved diastereo‐ and enantioselectivity (95 : 5 dr, 15 : 85 er) (entry 6). Variation in reactant stoichiometry, concentration, palladium source and ligand, or aryl ester substitution led to no further improvement in enantioselectivity (see Supporting Information for full details). Control experiments showed that in the absence of Pd(PPh_3_)_4_, no reaction occurred (entry 7). However, in the absence of (*S*)‐TM⋅HCl and *i*‐Pr_2_NEt, >90 % conversion to product as a 50 : 50 mixture of racemic diastereoisomers was observed (entry 8). Further control experiments investigated the effect of the salt *i*‐Pr_2_NEt⋅HCl, formed in situ upon deprotonation of the isothiourea catalyst TM⋅HCl **7**. Conducting the reaction with the free base TM in the absence of any salt led to a drastic decrease in diastereo‐ and enantioselectivity (entry 9). Alternatively, the use of isothiourea (2*S*,3*R*)‐HyperBTM **5** as its HCl salt was also investigated (entry 10). Compared to the use of the free base (entry 4), a significant improvement in stereoselectivity was observed, suggesting the salt additive plays a significant role in this cooperative process.

**Table 1:** Variation of Reaction Conditions.^[a]^

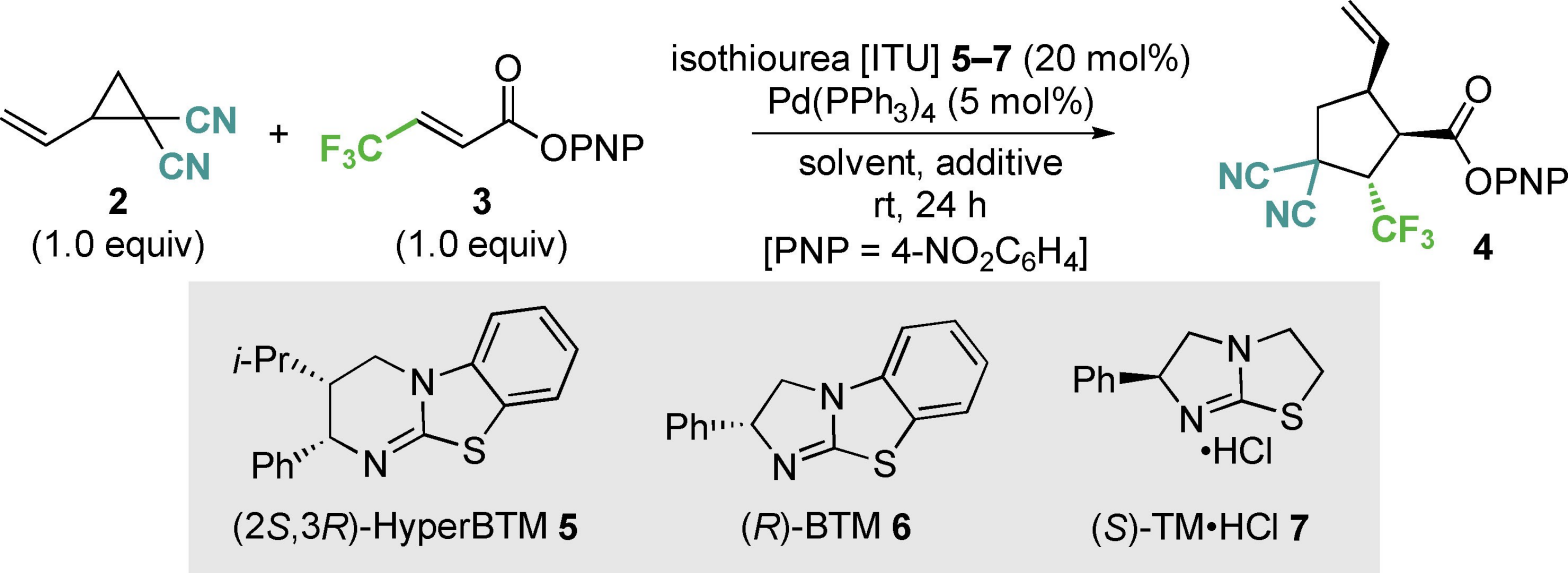						
						
Entry	ITU	solvent	variation	yield [%]^[b]^	dr^[c]^	er^[d]^
1	**5**	CH_2_Cl_2_	–	59	67 : 33	58 : 42
2	**5**	DMF	–	87	81 : 19	78 : 22
3	**5**	THF	–	55	83 : 17	78 : 22
4	**5**	acetone	–	93	77 : 23	72 : 28
5	**6**	acetone	–	83	65 : 35	73 : 27
6	**7**⋅HCl	acetone	*i‐*Pr_2_NEt^[e]^	81	95 : 5	15 : 85
7	**7**⋅HCl	acetone	No Pd, *i‐*Pr_2_NEt^[e]^	0	–	–
8	–	acetone	No ITU	95	50 : 50	50 : 50
9	(*S*)‐TM	acetone	–	99	67 : 33	32 : 68
10	**5**⋅HCl	acetone	–	78	>95 : 5	79 : 21
11	**6**	acetone	*i‐*Pr_2_NEt⋅HCl^[e]^	90	>95 : 5	83 : 17
12	**6**	acetone	Bu_4_NBr^[e]^	85	>95 : 5	80 : 20
13	**6**	acetone	LiCl^[e]^	92	>95 : 5	86 : 14
14	**6**	acetone	LiBr^[e]^	87	>95 : 5	84 : 16
15	**6**	acetone	NaOAc^[e]^	82	66 : 34	69 : 31
16	**6**	toluene	LiCl^[f]^	77	72 : 27	69 : 31
17	**6**	THF	LiCl^[f]^	63	95 : 5	92 : 8
18	**6**	EtOAc	LiCl^[f]^	88	91 : 9	91 : 9
19	**6**	EtOAc : THF 3 : 2	LiCl^[f]^	92 (78)	95 : 5	94 : 6

[a] Reactions performed on a 0.1 mmol scale. [b] Combined yield of product diastereoisomers determined by ^1^H NMR analysis of the crude material using 1,3,5‐trimethoxybenzene as internal standard. Isolated yield on gram scale in parentheses. [c] Determined by ^19^F{^1^H} NMR analysis of the crude material. [d] Determined by HPLC analysis on a chiral stationary phase. [e] 20 mol % additive. [f] 30 mol % additive. rt=room temperature.

The effect of salt additives (20–30 mol %) on the stereoselectivity of the reaction was further investigated with (*R*)‐BTM **6** instead of (*S*)‐TM⋅HCl **7** (entries 11–19). Salts containing halide ions led to high diastereoselectivity, irrespective of the nature of the cation (entries 11–14) with LiCl being optimal. In contrast, the use of NaOAc led to a comparable stereoselectivity to that observed without any salt additive (entry 15). The effect of halide ions in transition metal catalysis is well documented,^[28]^ with experimental^[29]^ and computational^[30]^ studies indicating that the addition of Cl^−^ ions generally increases the rate of π‐σ‐π isomerization within Pd π‐allyl intermediates. Screening of various isothiourea catalysts in the presence of 30 mol % LiCl as additive (see Supporting Information for details) all furnished the desired product in excellent diastereoselectivity (>95 : 5 dr) with similar product enantioselectivity (ca. 85 : 15 er). As a final optimization step, toluene, THF and EtOAc were selected for an extended solvent screen. In the presence of 30 mol % LiCl, THF and EtOAc gave the product with improved enantioselectivity (>90 : 10 er, entries 17, 18), with a combination of EtOAc and THF in a 3 : 2 ratio proving optimal, furnishing the product in >90 % conversion, 95 : 5 dr and 94 : 6 er (entry 19). To demonstrate the practicality of the developed cooperative catalysis process, the reaction was performed at gram scale, giving cyclopentane **4** (1.49 g) in 78 % isolated yield and excellent diastereo‐ and enantioselectivity (95 : 5 dr, 93 : 7 er).

### Scope and Limitations

With the optimal cooperative catalysis conditions established, the generality of this protocol was investigated through variation of the α,β‐unsaturated ester as well as the VCP reaction component (Table 2). An additional in situ derivatization through the addition of MeOH and DMAP after completing the catalytic process was employed to give the corresponding methyl ester products as these generally proved more stable to chromatographic purification than the corresponding PNP esters. Using these conditions, the CF_3_ and C_2_F_5_ containing cyclopentane methyl esters **8** and **9** were obtained in 75 % and 53 % yield respectively and with excellent stereoselectivity (>95 : 5 dr, >93 : 7 er). Using a CF_2_H substituent gave **10** in similar yield and dr, but with slightly reduced enantioselectivity (90 : 10 er). The medicinally relevant difluorophosphonate group CF_2_P(O)(OEt)_2_ ^[31]^ was also tolerated, although derivatization with MeOH and purification proved difficult, giving **11** in moderate yield, >95 : 5 dr but reduced enantioselectivity (81 : 19 er). The incorporation of other electron‐withdrawing β‐substituents was also tolerated, with ethyl and *tert*‐butyl ester derivatives **12** and **13** isolated with excellent diastereo‐ and enantioselectivity (≥91 : 9 dr, up to 95 : 5 er). The relative and absolute (1*R*,2*S*,5*S*)‐configuration within **13** was determined by single crystal X‐ray diffraction analysis, with all other products assigned by analogy.^[32]^ β‐Amide substituents also provided good reactivity, giving the products **14–16** in high yields (64–75 %) and excellent stereoselectivity (>95 : 5 dr, ≥91 : 9 er).

**Table 2:**
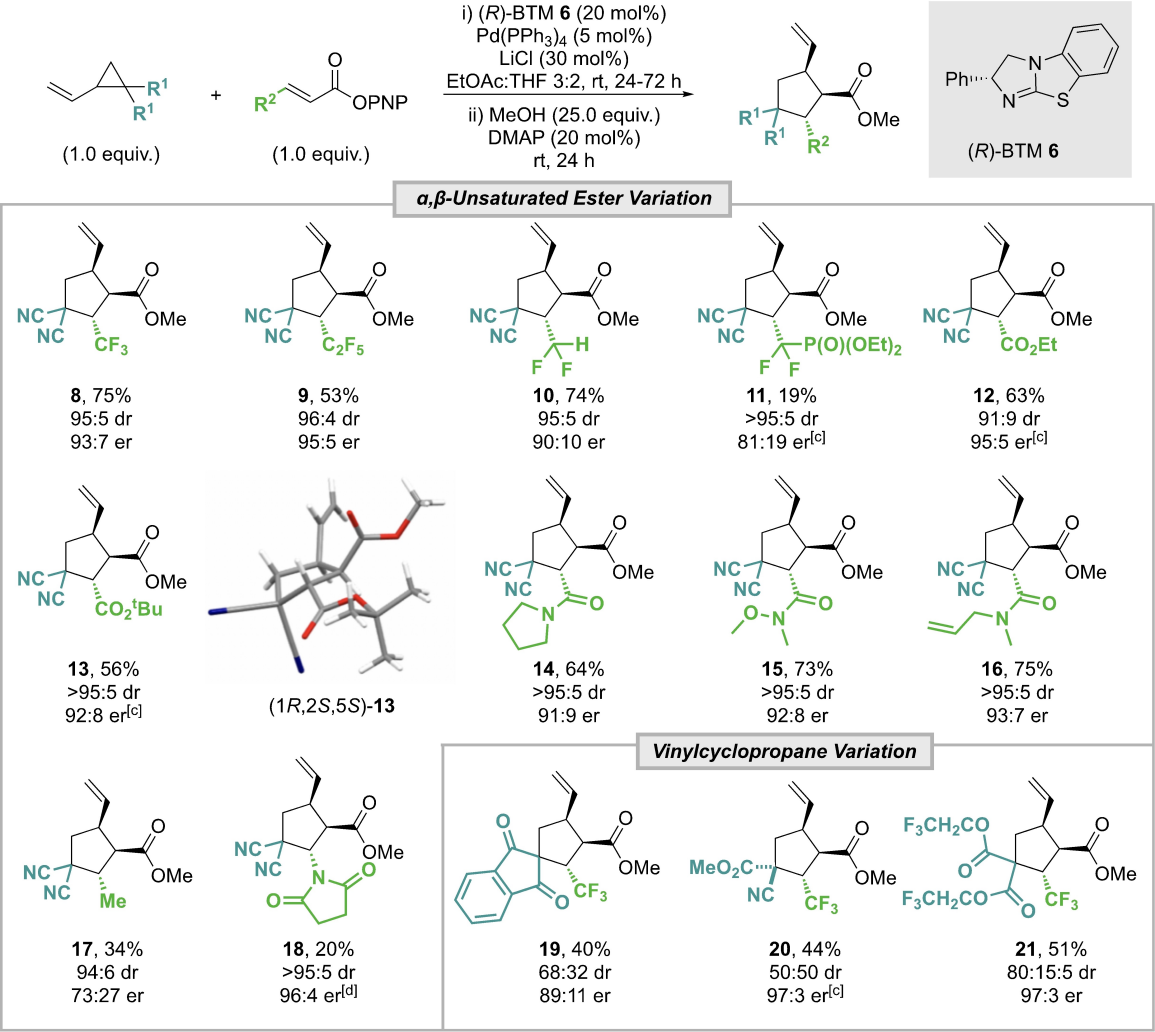
Scope and Limitations of the formal (3+2)‐cycloaddition using vinylcyclopropanes and α,β‐unsaturated esters.^[a,b]^

[a] Reactions performed on a 1.0 mmol scale; isolated yield of combined diastereoisomers; [b] dr determined by ^1^H or ^19^F{^1^H} NMR analysis of the crude reaction product; er determined by HPLC or GC analysis on a chiral stationary phase. [c] er determined by chiral stationary phase HPLC analysis from the intermediate PNP ester product. [d] er determined by ^19^F{^1^H} NMR analysis after derivatisation with (*S*)‐1‐(4′‐fluorophenyl)ethanol.

Limitations of this methodology showed that when R^2^=Me, reduced product yield and enantiocontrol was observed (94 : 6 dr, 73 : 27 er), while when R^2^=Ar no product was observed with only starting materials returned (see Supporting Information for full list of attempted substrates and proposed rationale for these observations). While an *N*‐succinimide substituent was tolerated, the corresponding cyclopentane product **18** was isolated in a moderate 20 % yield, but with excellent stereoselectivity (>95 : 5 dr, 96 : 4 er). Variation of the VCP reaction component was next explored. Use of a 1,3‐indanedione substituted VCP gave the spirocyclic product **19** in moderate 40 % yield but with good stereoselectivity (68 : 32 dr, 89 : 11 er). Employing an unsymmetrically substituted VCP bearing a nitrile and an ester group gave **20** as a 50 : 50 mixture of diastereoisomers with excellent enantioselectivity (97 : 3 er). The use of di(trifluoroethyl) ester substituted VCP gave cyclopentane **21** in 51 % isolated yield with excellent enantioselectivity (80 : 15 : 5 dr, 97 : 3 er).

**Scheme 1:**
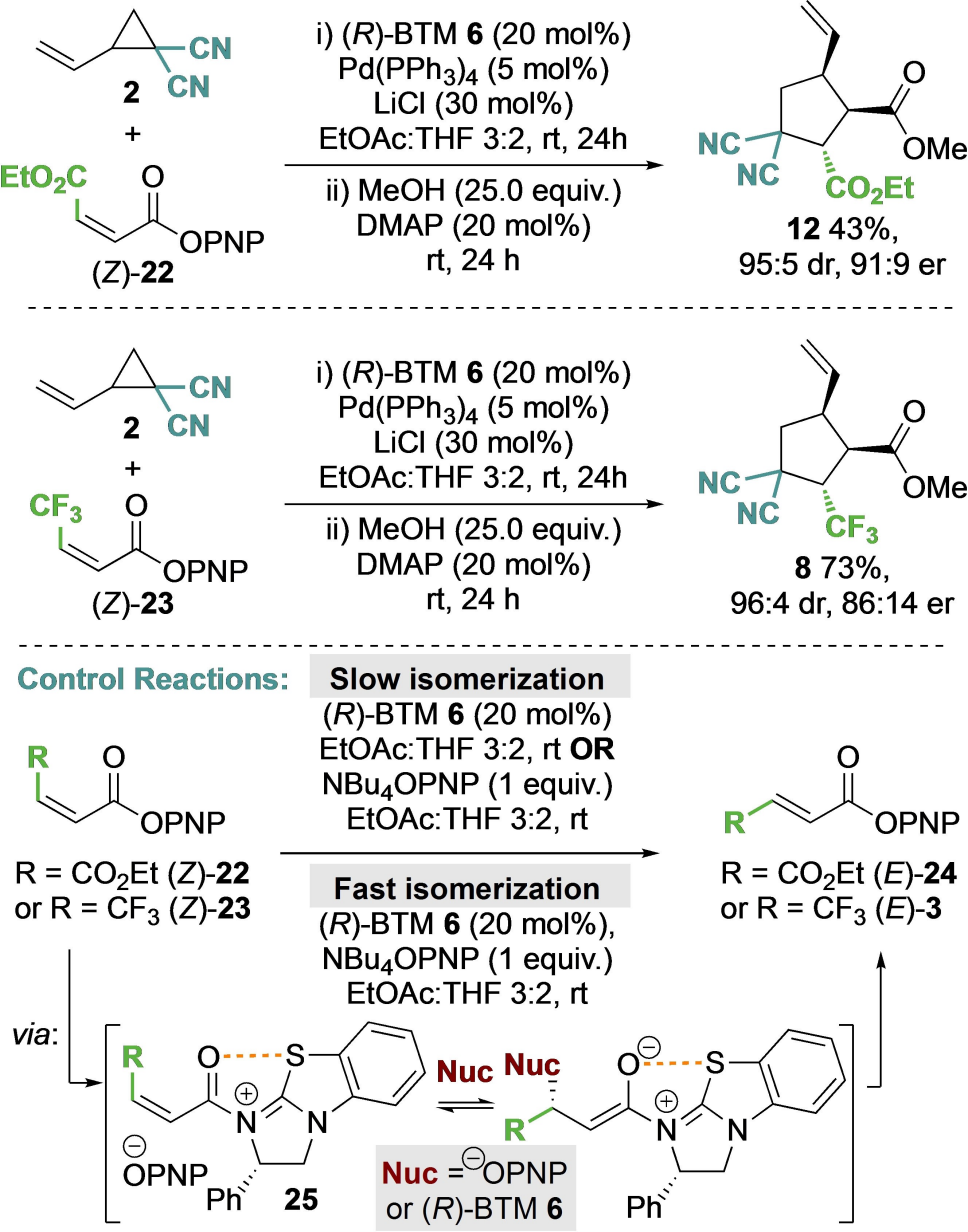
Effect of olefin configuration on product outcome and isomerization studies.

The effect of olefin configuration on product yield and stereoselectivity was next investigated using both maleate and CF_3_‐substituted α,β‐unsaturated PNP ester derivatives **22** and **23** (Scheme 1). In both cases when using the (*Z*)‐enoate, the corresponding cyclopentane products **12** and **8** were obtained in high dr and in the same enantiomeric series as from the (*E*)‐enoate, but with reduced er (91 : 9 er and 86 : 14 er respectively). In the literature, the isomerization of maleate esters to the corresponding fumarate derivatives has been widely investigated. The use of bromine radicals,^[33]^ zwitterionic organocatalysts,^[34]^ secondary amines,^[35]^ aminals,^[36]^ imidazolium ionic liquids,^[37]^ thiophenolate,^[38]^ as well as enzymatic catalysis,^[39]^ have all been shown to promote isomerization. In all cases the proposed mechanism involves reversible nucleophilic addition that leads to the thermodynamically preferred (*E*)‐enoate. Intrigued by these precedents and the observed effect of olefin configuration on the stereochemical outcome of the developed process, a series of control reactions to probe enoate isomerization were carried out.

Reaction monitoring showed that addition of BTM **6** (20 mol %) or NBu_4_OPNP (1 equiv) to (*Z*)‐maleate **22** led to relatively slow isomerization, giving a 13 : 87 and 53 : 47 mixture of (*Z*):(*E*)‐enoates respectively after 24 hours but indicating the feasibility of isomerization. However, treatment of (*Z*)‐maleate **22** with BTM **6** (20 mol %) and NBu_4_OPNP (1 equiv) led to rapid isomerization, giving a 3 : 97 mixture of (*Z*) : (*E*)‐enoates within 2 hours. To demonstrate the key role of the PNP ester functionality, control reactions showed that treatment of diethyl maleate with either BTM **6** (20 mol %) or NBu_4_OPNP (1 equiv), or both, led to no isomerization after 24 hours. Further studies used ^19^F{^1^H} NMR spectroscopy to monitor in situ isomerization of (*Z*)‐CF_3_‐substituted ester **23** upon treatment with BTM **6** (20 mol %). Rapid isomerization to generate a 14 : 86 mixture of (*Z*) : (*E*)‐enoates within one hour was observed, but no intermediates could be detected using this technique. Taken together these observations are consistent with a possible mechanism for this isomerization process involving initial *N*‐acylation of (*R*)‐BTM **6** with (*Z*)‐**22** or (*Z*)‐**23** to give the corresponding α,β‐unsaturated acyl ammonium ion pair **25**. Subsequent reversible conjugate addition of *para*‐nitrophenolate (or potentially (*R*)‐BTM **6**), followed by bond rotation and elimination, will lead to the thermodynamically favored (*E*)‐enoate.

**Scheme 2:**
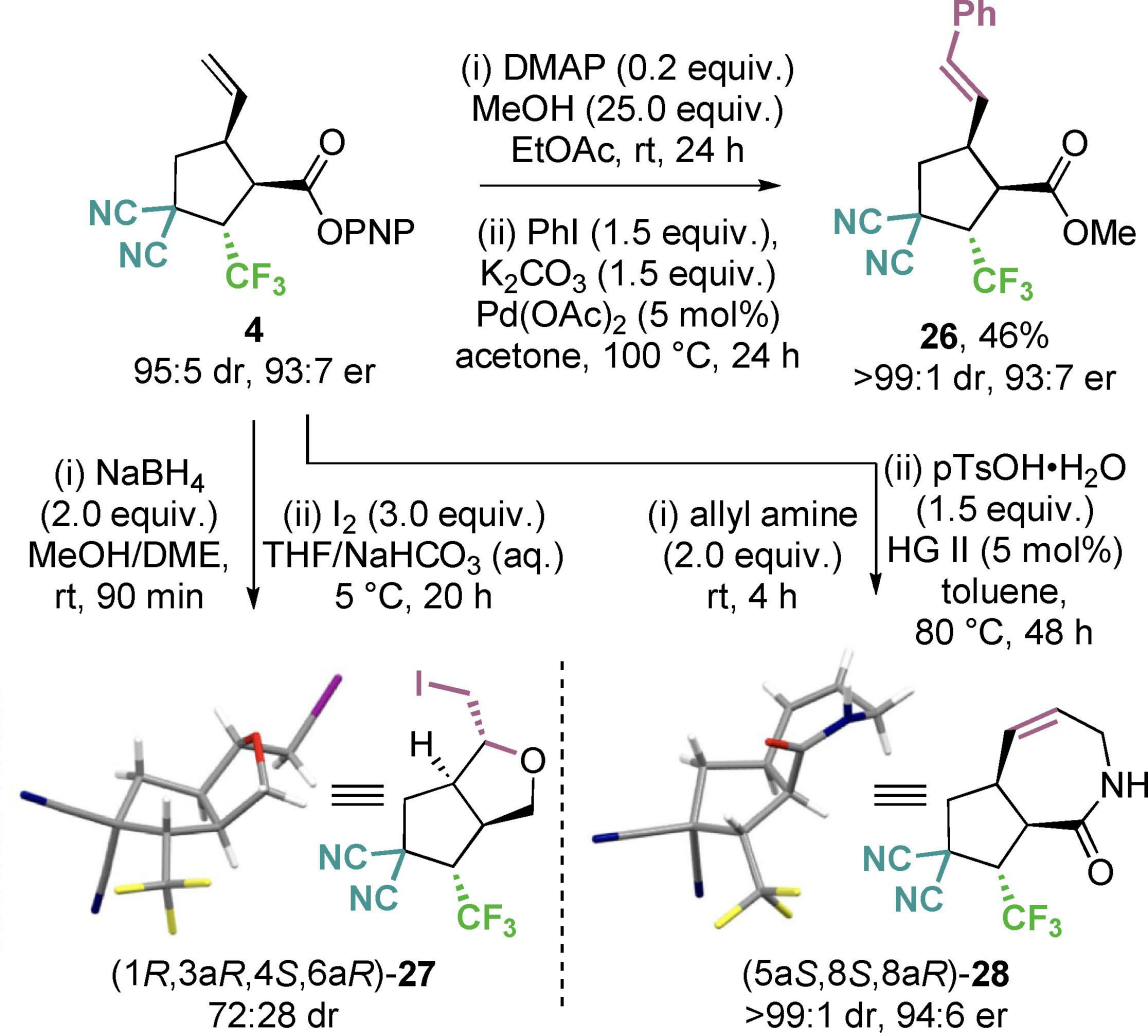
Derivatization studies.

To further demonstrate the utility of the developed cooperative catalysis process derivatization of PNP ester **4** to give a range of products was also explored (Scheme 2). Treatment of **4** with MeOH and DMAP, followed by subsequent Heck coupling gave (*E*)‐alkene **26** in modest 42 % yield over 2 steps without erosion of enantiopurity. Alternatively, reduction of the ester to the alcohol, followed by treatment with I_2_ facilitated intramolecular iodocyclisation to give a separable mixture of diastereoisomers (72 : 28 dr). The relative and absolute configuration of the major diastereoisomer **27** was confirmed by single crystal X‐ray diffraction analysis.^[32]^ As a final derivatization, treatment of PNP ester **4** with allyl amine, followed by ring‐closing metathesis furnished bicyclic lactam **28**. The structure and relative configuration of lactam **28** was further confirmed by single crystal X‐ray diffraction analysis.^[32]^

**Scheme 3:**
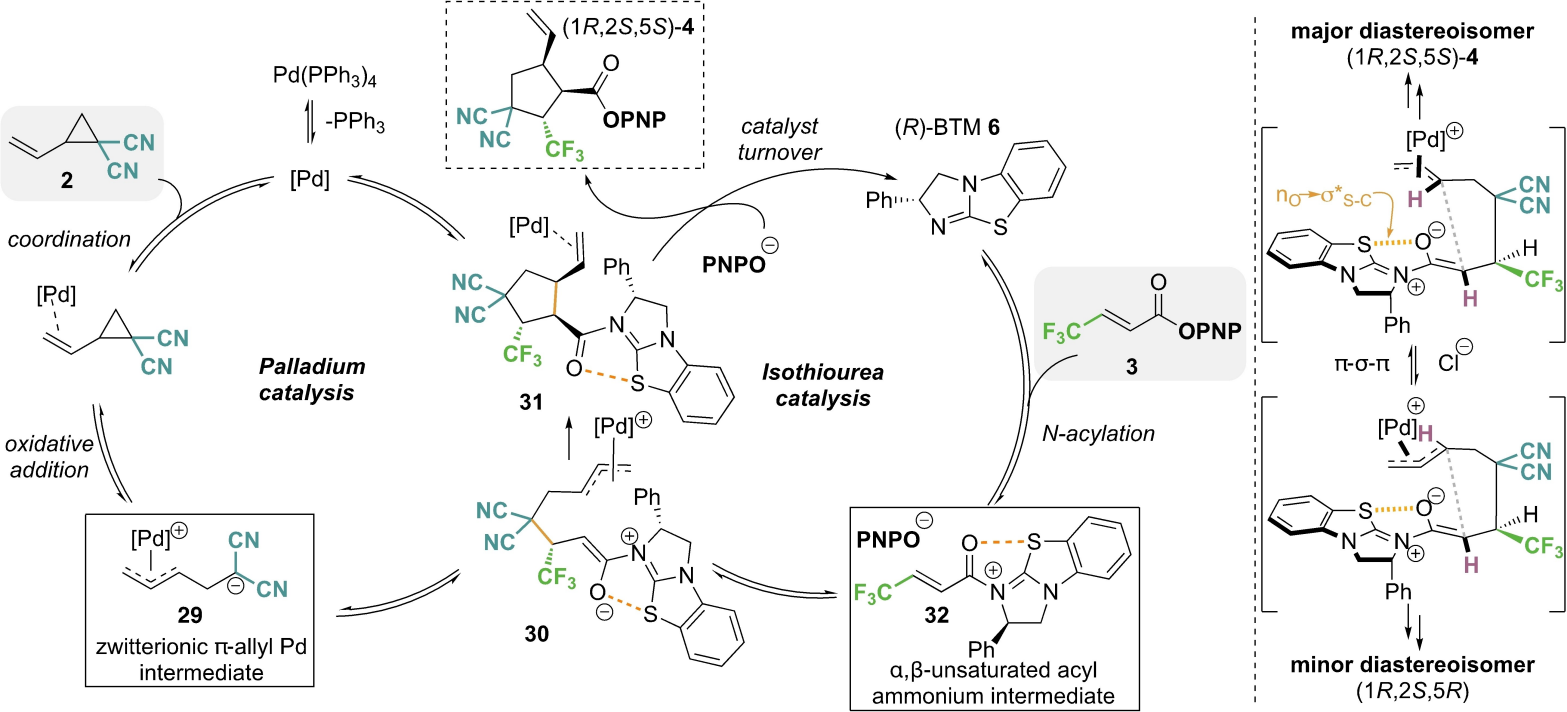
Proposed mechanism and tentative stereochemical rationale.

Based upon the general understanding of both palladium π‐allyl and α,β‐unsaturated acyl ammonium catalysis, the following mechanism for the developed cooperative catalysis is proposed (Scheme 3). Starting from commercially available Pd(PPh_3_)_4_, ligand dissociation enables reversible coordination to the vinylcyclopropane **2**. Subsequent oxidative addition generates zwitterionic palladium π‐allyl intermediate **29**. Concurrently, isothiourea catalyst (*R*)‐BTM **6** undergoes reversible *N*‐acylation with PNP ester **3**, generating α,β‐unsaturated acyl ammonium ion pair **32**. Subsequent Michael addition gives **30**, followed by intramolecular ring closure to generate cyclopentane **31**. Decomplexation of the palladium catalyst and irreversible turnover of the isothiourea catalyst by *para*‐nitrophenoxide furnishes **4**. The stereochemical outcome can be rationalised by invoking a stabilising 1,5‐S⋅⋅⋅O chalcogen bonding interaction[^40^, ^41^, ^42^, ^43^] between the carbonyl oxygen and the isothiourea sulfur atom (n_O_ to σ*_C‐S_) that restricts the conformational freedom of the α,β‐unsaturated acyl ammonium ion. Conjugate addition anti‐to the stereodirecting phenyl group in the s‐*cis* conformation generates the enolate intermediate **30** that then undergoes intramolecular ring closure. The addition of Cl^−^ ions is postulated to promote π‐σ‐π isomerization, leading to excellent diastereoselectivity in favour of the (1*R*,2*S*,5*S*)‐isomer (95 : 5 dr).

## Conclusion

This manuscript describes the first application of α,β‐unsaturated acyl ammonium intermediates in a dual cooperative catalytic process. The simultaneous activation of vinylcyclopropanes and α,β‐unsaturated *p*‐nitrophenyl esters in the presence of catalytic Pd(PPh_3_)_4_ (5 mol %) and (*R*)‐BTM **6** (20 mol %) facilitates intermolecular, formal (3+2) cycloaddition to generate functionalized cyclopentanes. The addition of LiCl (30 mol %) was crucial for obtaining high levels of diastereo‐ and enantioselectivity. β‐electron‐withdrawing substituents within the α,β‐unsaturated ester are required for optimal reactivity, making the current methodology complementary to existing enantioselective processes. Further applications of the use of cooperative catalysis using a combination of isothiourea and transition metal catalysts are underway in this laboratory.^[44]^

## Conflict of interest

The authors declare no conflict of interest.

1

## Supporting information

As a service to our authors and readers, this journal provides supporting information supplied by the authors. Such materials are peer reviewed and may be re‐organized for online delivery, but are not copy‐edited or typeset. Technical support issues arising from supporting information (other than missing files) should be addressed to the authors.

Supporting Information

Supporting Information

Supporting Information

Supporting Information

Supporting Information

Supporting Information

Supporting Information

Supporting Information

Supporting Information

Supporting Information

## Data Availability

The data that support the findings of this study are openly available in University of St Andrews Research Portal at https://doi.org/10.17630/7f6e1710‐b881‐44f7‐9c94‐13129f53d824.
