# Supplementary material for: Cooperative Palladium/Isothiourea Catalyzed Enantioselective Formal (3+2) Cycloaddition of Vinylcyclopropanes and α,β‐Unsaturated Esters
Source: Angew Chem Int Ed Engl. 2022 Apr 28;61(25):e202202621. doi: 10.1002/anie.202202621 (PMC9324207; doi:10.1002/anie.202202621)
Supplement: Supplementary file 2 — Supporting Information [file ANIE-61-0-s006.rtf]

 


Data Collection

	A colorless prism crystal of C16H20N2O4 having approximate dimensions of 0.200 x 0.100 x 0.100 mm was mounted in a loop. All measurements were made on a Rigaku XtaLAB P100 diffractometer using multi-layer mirror monochromated Cu-Ka radiation.


	Cell constants and an orientation matrix for data collection corresponded to a primitive monoclinic cell with dimensions:

           a  =    9.560(3) Å
           b  =   15.563(4) Å        b  =   90.427(6)o
           c  =   11.591(4) Å
           V  =  1724.5(9) Å3

For Z = 4 and F.W. = 304.34, the calculated density is 1.172 g/cm3. Based on the reflection conditions of:

           0k0:  k = 2n


packing considerations, a statistical analysis of intensity distribution, and the successful solution and refinement of the structure, the space group was determined to be:

P21 (#4) 


	The data were collected at a temperature of -100 + 1oC to a maximum 2q value of 135.7o. 

Data Reduction

	Of the 18151 reflections were collected, where 5516 were unique (Rint = 0.0520); equivalent reflections were merged. Data were collected and processed using CrystalClear (Rigaku). 1 

	The linear absorption coefficient, m, for Cu-Ka radiation is 7.006 cm-1. An empirical absorption correction was applied which resulted in transmission factors ranging from 0.727 to 0.932. The data were corrected for Lorentz and polarization effects. A correction for secondary extinction2 was applied (coefficient = 0.029630). 


Structure Solution and Refinement

	The structure was solved by direct methods3 and expanded using Fourier techniques. The non-hydrogen atoms were refined anisotropically. Hydrogen atoms were refined using the riding model. The final cycle of full-matrix least-squares refinement4 on F2 was based on 5516 observed reflections and 406 variable parameters and converged (largest parameter shift was 0.00 times its esd) with unweighted and weighted agreement factors of: 

R1 = S ||Fo| - |Fc|| / S |Fo| = 0.0556 


wR2 = [ S ( w (Fo2 - Fc2)2 )/ S w(Fo2)2]1/2 = 0.1343 


	The goodness of fit5 was 1.05. Unit weights were used.  The maximum and minimum peaks on the final difference Fourier map corresponded to 0.62 and -0.63 e/Å3, respectively. The final Flack parameter 6 was 0.04(6),  indicating that the present absolute structure is correct. 7 

	Neutral atom scattering factors were taken from International Tables for Crystallography (IT), Vol. C, Table 6.1.1.4 8.  Anomalous dispersion effects were included in Fcalc9; the values for Df' and Df" were those of Creagh and McAuley10. The values for the mass attenuation coefficients are those of Creagh and Hubbell11. All calculations were performed using the CrystalStructure12 crystallographic software package except for refinement, which was performed using SHELXL Version 2018/313.


References 


(1) CrystalClear: Data Collection and Processing Software, Rigaku Corporation (1998-2015). Tokyo 196-8666, Japan.

(2) Larson, A.C. (1970), Crystallographic Computing, 291-294. F.R. Ahmed, ed. Munksgaard, Copenhagen (equation 22, with V replaced by the cell volume).

(3) SHELXT Version 2018/2: Sheldrick, G. M. (2014). Acta Cryst. A70, C1437.

(4) Least Squares function minimized: (SHELXL Version 2018/3)

		Sw(Fo2-Fc2)2     where w = Least Squares weights.

(5) Goodness of fit is defined as:

 	 	 [Sw(Fo2-Fc2)2/(No-Nv)]1/2

	 	 where:	 No  = number of observations
	 	 	 	 Nv  = number of variables 


(6) Parsons, S., Flack, H.D. and Wagner, T. Acta Cryst. B69 (2013) 249-259.

(7) Flack, H.D. and Bernardinelli (2000), J. Appl. Cryst. 33, 114-1148.

(8) International Tables for Crystallography, Vol.C (1992). Ed. A.J.C. Wilson, Kluwer Academic Publishers, Dordrecht, Netherlands, Table 6.1.1.4, pp. 572. 

(9) Ibers, J. A. & Hamilton, W. C.; Acta Crystallogr., 17, 781 (1964). 

(10) Creagh, D. C. & McAuley, W.J .; "International Tables for Crystallography", Vol C, (A.J.C. Wilson, ed.), Kluwer Academic Publishers, Boston, Table 4.2.6.8, pages 219-222 (1992). 

(11) Creagh, D. C. & Hubbell, J.H..; "International Tables for Crystallography", Vol C, (A.J.C. Wilson, ed.), Kluwer Academic Publishers, Boston, Table 4.2.4.3, pages 200-206 (1992). 

(12) CrystalStructure 4.3: Crystal Structure Analysis Package, Rigaku Corporation (2000-2019). Tokyo 196-8666, Japan.

(13) SHELXL Version 2018/3: Sheldrick, G. M. (2008). Acta Cryst. A64, 112-122.


EXPERIMENTAL DETAILS 


A. Crystal Data 


Empirical Formula	C16H20N2O4

Formula Weight	304.34

Crystal Color, Habit	colorless, prism

Crystal Dimensions	0.200 X 0.100 X 0.100 mm

Crystal System	monoclinic

Lattice Type	Primitive

Lattice Parameters	a =   9.560(3) Å
	b =  15.563(4) Å
	c =  11.591(4) Å
	b =  90.427(6) o
	V = 1724.5(9) Å3

Space Group	P21 (#4)

Z value	4

Dcalc	1.172 g/cm3

F000	648.00

m(CuKa)	7.006 cm-1

B. Intensity Measurements 


Diffractometer	XtaLAB P100

Radiation	CuKa (l = 1.54187 Å)
	multi-layer mirror monochromated

Voltage, Current	40kV, 30mA

Temperature	-100.0oC

Detector Aperture	83.8 x 33.5 mm

Data Images	3983 exposures

Pixel Size	0.172 mm

2qmax	135.7o

No. of Reflections Measured	Total: 18151
	Unique: 5516 (Rint = 0.0520)
	Parsons quotients (Flack x parameter): 2171

Corrections	Lorentz-polarization
		Absorption
		(trans. factors: 0.727 - 0.932)
		Secondary Extinction
		(coefficient: 2.96300e-002)

C. Structure Solution and Refinement 


Structure Solution	Direct Methods (SHELXT Version 2018/2)

Refinement	Full-matrix least-squares on F2

Function Minimized	S w (Fo2 - Fc2)2 

Least Squares Weights	w = 1/ [ s2(Fo2) + (0.0945 . P)2 
	 + 0.2230 .  P ]
	 where P = (Max(Fo2,0) + 2Fc2)/3

2qmax cutoff	135.7o

Anomalous Dispersion	All non-hydrogen atoms

No. Observations (All reflections)	5516

No. Variables	406

Reflection/Parameter Ratio	13.59

Residuals: R1 (I>2.00s(I))	0.0556

Residuals: R (All reflections)	0.0566

Residuals: wR2 (All reflections)	0.1343

Goodness of Fit Indicator	1.052

Flack parameter (Parsons' quotients = 2171)	0.04(6)

Max Shift/Error in Final Cycle	0.001

Maximum peak in Final Diff. Map	0.62 e/Å3

Minimum peak in Final Diff. Map	-0.63 e/Å3


Table 1. Atomic coordinates and Biso/Beq

atom	   x	   y	   z	 Beq
O8    	 0.1222(2)	 0.44396(14)	 0.66312(19)	 3.48(4)
O9    	-0.1016(2)	 0.43392(15)	 0.7135(2)	 4.30(5)
O10   	 0.0772(3)	 0.73903(16)	 0.8035(2)	 4.69(5)
O11   	 0.0361(2)	 0.60073(16)	 0.85357(17)	 3.63(4)
O28   	 0.3819(2)	 0.57511(15)	 0.77652(19)	 3.68(4)
O29   	 0.6123(2)	 0.59333(15)	 0.7574(2)	 3.93(4)
O30   	 0.4266(3)	 0.29750(16)	 0.94821(19)	 4.43(5)
O31   	 0.4858(2)	 0.43765(14)	 0.96857(16)	 3.53(4)
N15   	-0.1494(3)	 0.7872(2)	 0.5772(3)	 4.69(6)
N16   	 0.3070(4)	 0.7889(2)	 0.5717(3)	 4.97(7)
N35   	 0.2085(3)	 0.2096(2)	 0.7142(3)	 4.38(6)
N36   	 0.6510(3)	 0.2214(2)	 0.7253(3)	 4.61(6)
C1    	-0.0316(3)	 0.56195(18)	 0.6229(2)	 2.67(4)
C2    	 0.0803(3)	 0.62751(18)	 0.6613(2)	 2.60(4)
C3    	 0.0801(3)	 0.6955(2)	 0.5650(2)	 3.02(5)
C4    	 0.0716(3)	 0.6376(2)	 0.4566(2)	 3.39(5)
C5    	-0.0362(3)	 0.5692(2)	 0.4864(3)	 3.27(5)
C6    	-0.0119(4)	 0.4879(2)	 0.4206(3)	 4.20(6)
C7    	-0.0931(6)	 0.4594(3)	 0.3385(4)	 5.84(10)
C8    	 0.0068(3)	 0.47431(18)	 0.6674(2)	 2.76(5)
C9    	-0.0704(5)	 0.3515(3)	 0.7669(5)	 6.23(11)
C10   	 0.0640(3)	 0.6649(2)	 0.7813(2)	 3.11(5)
C11   	 0.0238(5)	 0.6164(3)	 0.9796(3)	 5.17(9)
C12   	 0.1601(6)	 0.6505(5)	 1.0255(4)	 7.35(13)
C13   	-0.0045(8)	 0.5258(5)	 1.0230(4)	 8.04(15)
C14   	-0.0995(6)	 0.6754(5)	 0.9999(4)	 8.12(16)
C15   	-0.0471(3)	 0.7500(2)	 0.5725(3)	 3.54(5)
C16   	 0.2061(3)	 0.7498(2)	 0.5691(3)	 3.59(5)
C21   	 0.5280(3)	 0.45331(19)	 0.7327(2)	 2.74(5)
C22   	 0.4212(3)	 0.39175(19)	 0.7846(2)	 2.69(5)
C23   	 0.4231(3)	 0.31378(19)	 0.7016(2)	 2.83(5)
C24   	 0.4261(3)	 0.3592(2)	 0.5829(2)	 3.06(5)
C25   	 0.5257(3)	 0.4350(2)	 0.5988(2)	 3.23(5)
C26   	 0.4787(7)	 0.5081(3)	 0.5274(3)	 6.76(13)
C27   	 0.5306(13)	 0.5551(5)	 0.4459(6)	 20.1(7)
C28   	 0.4963(3)	 0.5463(2)	 0.7597(2)	 2.87(5)
C29   	 0.5927(5)	 0.6850(3)	 0.7737(5)	 5.47(9)


Table 1. Atomic coordinates and Biso/Beq (continued)

atom	   x	   y	   z	 Beq
C30   	 0.4450(3)	 0.3679(2)	 0.9103(2)	 3.16(5)
C31   	 0.5137(4)	 0.4354(3)	 1.0953(3)	 3.98(6)
C32   	 0.6322(4)	 0.3726(3)	 1.1206(3)	 5.18(8)
C33   	 0.5571(6)	 0.5284(3)	 1.1187(3)	 5.72(9)
C34   	 0.3815(5)	 0.4132(3)	 1.1572(3)	 5.33(8)
C35   	 0.3014(3)	 0.2556(2)	 0.7126(2)	 3.38(5)
C36   	 0.5511(3)	 0.2608(2)	 0.7177(3)	 3.42(5)

Beq = 8/3 p2(U11(aa*)2 + U22(bb*)2 + U33(cc*)2 + 2U12(aa*bb*)cos g + 2U13(aa*cc*)cos b + 2U23(bb*cc*)cos a)


Table 2. Atomic coordinates and Biso involving hydrogen atoms

atom	   x	   y	   z	 Biso
H1    	-0.12437	 0.57916	 0.65473	 3.208
H2    	 0.17285	 0.59767	 0.65921	 3.125
H4A   	 0.04126	 0.67111	 0.38829	 4.065
H4B   	 0.16352	 0.61105	 0.44041	 4.065
H5    	-0.13043	 0.59176	 0.46378	 3.928
H6    	 0.06844	 0.45487	 0.44005	 5.035
H7A   	-0.17433	 0.49078	 0.31678	 7.008
H7B   	-0.07115	 0.40712	 0.30033	 7.008
H9A   	-0.00864	 0.36037	 0.83370	 7.472
H9B   	-0.02387	 0.31421	 0.71087	 7.472
H9C   	-0.15753	 0.32447	 0.79203	 7.472
H12A  	 0.17319	 0.70967	 0.99877	 8.815
H12B  	 0.23699	 0.61461	 0.99759	 8.815
H12C  	 0.15907	 0.64945	 1.10999	 8.815
H13A  	 0.07924	 0.49039	 1.01259	 9.649
H13B  	-0.08260	 0.50084	 0.97921	 9.649
H13C  	-0.02831	 0.52788	 1.10500	 9.649
H14A  	-0.18259	 0.65211	 0.96108	 9.745
H14B  	-0.07869	 0.73256	 0.96890	 9.745
H14C  	-0.11693	 0.67982	 1.08292	 9.745
H21   	 0.62287	 0.43887	 0.76400	 3.283
H22   	 0.32704	 0.41936	 0.77815	 3.229
H24A  	 0.46068	 0.31970	 0.52251	 3.673
H24B  	 0.33144	 0.37936	 0.56066	 3.673
H25   	 0.62149	 0.41746	 0.57407	 3.878
H26   	 0.38622	 0.52521	 0.54602	 8.106
H27A  	 0.62270	 0.54451	 0.41920	 24.109
H27B  	 0.47643	 0.60009	 0.41281	 24.109
H29A  	 0.56576	 0.69619	 0.85373	 6.568
H29B  	 0.51885	 0.70550	 0.72150	 6.568
H29C  	 0.68020	 0.71513	 0.75693	 6.568
H32A  	 0.71507	 0.38930	 1.07673	 6.219
H32B  	 0.60336	 0.31452	 1.09819	 6.219
H32C  	 0.65434	 0.37369	 1.20330	 6.219
H33A  	 0.48067	 0.56704	 1.09648	 6.866
H33B  	 0.64050	 0.54214	 1.07360	 6.866
H33C  	 0.57812	 0.53548	 1.20103	 6.866


Table 2. Atomic coordinates and Biso involving hydrogens/Beq (continued)

atom	   x	   y	   z	 Beq
H34A  	 0.34501	 0.35845	 1.12794	 6.396
H34B  	 0.31198	 0.45851	 1.14418	 6.396
H34C  	 0.40097	 0.40807	 1.24006	 6.396


Table 3. Anisotropic displacement parameters

atom	  U11	  U22	  U33	  U12	  U13	  U23
O8    	0.0377(10)	0.0396(12)	0.0551(12)	 0.0066(9)	-0.0029(8)	 0.0027(9)
O9    	0.0371(10)	0.0368(13)	0.0896(17)	-0.0031(9)	 0.0016(10)	 0.0136(11)
O10   	0.0897(18)	0.0416(14)	0.0468(12)	-0.0076(12)	 -0.0003(11)	-0.0082(10)
O11   	0.0539(12)	0.0504(13)	0.0335(10)	-0.0110(10)	 0.0014(8)	 0.0013(9)
O28   	0.0403(11)	0.0437(13)	0.0559(12)	 0.0095(9)	-0.0018(8)	-0.0055(10)
O29   	0.0444(11)	0.0364(12)	0.0684(14)	-0.0045(9)	 0.0032(9)	-0.0071(10)
O30   	0.0817(16)	0.0442(14)	0.0425(11)	-0.0121(12)	-0.0112(10)	 0.0072(10)
O31   	0.0608(12)	0.0406(12)	0.0328(10)	-0.0051(10)	-0.0101(8)	 0.0002(8)
N15   	0.0556(17)	0.0470(18)	0.075(2)	 0.0105(14)	-0.0083(14)	 0.0041(14)
N16   	0.0629(17)	0.060(2)	0.0659(18)	-0.0238(16)	 0.0104(14)	 0.0010(15)
N35   	0.0566(16)	0.0583(18)	0.0513(14)	-0.0196(15)	-0.0068(12)	-0.0021(13)
N36   	0.0561(16)	0.0496(18)	0.0691(18)	 0.0145(14)	-0.0136(13)	-0.0046(13)
C1    	0.0303(11)	0.0300(14)	0.0412(13)	 0.0007(10)	-0.0045(9)	-0.0013(10)
C2    	0.0325(12)	0.0305(14)	0.0359(13)	 0.0001(11)	-0.0023(9)	 0.0008(10)
C3    	0.0410(14)	0.0360(16)	0.0377(14)	-0.0022(12)	-0.0012(10)	 0.0033(11)
C4    	0.0544(16)	0.0390(16)	0.0352(13)	-0.0014(14)	-0.0032(11)	 0.0011(12)
C5    	0.0450(14)	0.0386(17)	0.0405(14)	 0.0014(12)	-0.0122(11)	 0.0008(12)
C6    	0.076(2)	0.0378(18)	0.0450(16)	-0.0018(16)	-0.0118(15)	-0.0026(13)
C7    	0.113(3)	0.052(2)	0.057(2)	 -0.002(2)	-0.026(2)	-0.0113(17)
C8    	0.0294(12)	0.0331(15)	0.0422(13)	 -0.0007(11)	-0.0071(10)	 0.0011(11)
C9    	0.064(2)	0.044(2)	0.129(4)	-0.0020(18)	 0.007(2)	 0.032(2)
C10   	0.0391(14)	0.0408(18)	0.0380(14)	-0.0032(11)	-0.0022(10)	 -0.0002(11)
C11   	0.080(2)	0.084(3)	0.0328(16)	-0.016(2)	 0.0065(14)	 -0.0015(16)
C12   	0.104(4)	0.126(5)	0.049(2)	-0.028(3)	-0.024(2)	 -0.001(2)
C13   	0.144(5)	0.117(5)	0.044(2)	-0.037(4)	 0.004(3)	 0.022(2)
C14   	0.105(4)	0.139(6)	0.065(3)	 0.019(4)	 0.036(3)	-0.010(3)
C15   	0.0533(17)	0.0318(16)	0.0491(16)	 -0.0012(14)	-0.0075(12)	 0.0039(12)
C16   	0.0503(16)	0.0420(17)	0.0442(15)	-0.0071(14)	 0.0029(12)	 0.0029(13)
C21   	0.0326(12)	0.0340(15)	0.0373(13)	 0.0018(11)	-0.0033(10)	 0.0015(10)
C22   	0.0343(12)	0.0358(15)	0.0321(13)	 0.0006(11)	-0.0043(9)	-0.0021(10)
C23   	0.0362(13)	0.0353(15)	0.0358(13)	-0.0016(11)	-0.0047(10)	-0.0034(11)
C24   	0.0363(13)	0.0450(17)	0.0349(13)	 0.0026(12)	-0.0034(10)	-0.0036(12)
C25   	0.0439(14)	0.0389(16)	0.0400(14)	 0.0010(13)	 0.0055(11)	-0.0056(12)
C26   	0.176(5)	0.045(2)	0.0354(18)	 0.010(3)	 -0.002(2)	 0.0029(15)
C27   	0.53(2)	0.172(9)	0.062(4)	 0.202(13)	 -0.003(7)	 0.009(5)
C28   	0.0371(14)	0.0376(15)	0.0344(12)	 -0.0009(11)	-0.0031(10)	-0.0013(11)
C29   	0.068(2)	0.038(2)	0.102(3)	-0.0079(17)	 0.009(2)	-0.0128(19)


Table 3. Anisotropic displacement parameters (continued)

atom	  U11	  U22	  U33	  U12	  U13	  U23
C30   	0.0442(14)	0.0404(17)	0.0353(13)	 -0.0010(12)	-0.0045(10)	 0.0010(12)
C31   	0.0655(19)	0.051(2)	0.0345(15)	-0.0021(16)	-0.0115(13)	-0.0018(13)
C32   	0.067(2)	0.073(3)	0.0567(19)	-0.002(2)	-0.0229(16)	 0.0060(19)
C33   	0.104(3)	0.063(3)	0.0503(19)	-0.012(2)	-0.021(2)	-0.0134(18)
C34   	0.080(3)	0.078(3)	0.0440(18)	 0.004(2)	 0.0056(16)	 0.0001(17)
C35   	0.0445(15)	0.0461(18)	0.0379(13)	-0.0058(14)	-0.0054(11)	-0.0019(12)
C36   	0.0493(16)	0.0362(17)	0.0444(15)	 0.0006(14)	-0.0073(12)	-0.0053(12)


The general temperature factor expression: exp(-2p2(a*2U11h2 + b*2U22k2 + c*2U33l2 + 2a*b*U12hk + 2a*c*U13hl + 2b*c*U23kl))


Table 4. Fragment Analysis


fragment: 1  
	O(8)	O(9)	O(10)	O(11)	N(15)
	N(16)	C(1)	C(2)	C(3)	C(4)
	C(5)	C(6)	C(7)	C(8)	C(9)
	C(10)	C(11)	C(12)	C(13)	C(14)
	C(15)	C(16)


fragment: 2  
	O(28)	O(29)	O(30)	O(31)	N(35)
	N(36)	C(21)	C(22)	C(23)	C(24)
	C(25)	C(26)	C(27)	C(28)	C(29)
	C(30)	C(31)	C(32)	C(33)	C(34)
	C(35)	C(36)


Table 5. Bond lengths (Å)

atom	atom	distance		atom	atom	distance
O8	C8	1.202(3)		O9	C8	1.327(3)	
O9	C9	1.454(5)		O10	C10	1.188(4)	
O11	C10	1.332(4)		O11	C11	1.487(4)	
O28	C28	1.200(3)		O29	C28	1.330(4)	
O29	C29	1.451(5)		O30	C30	1.194(4)	
O31	C30	1.335(4)		O31	C31	1.491(3)	
N15	C15	1.138(5)		N16	C16	1.141(5)	
N35	C35	1.142(4)		N36	C36	1.138(4)	
C1	C2	1.542(4)		C1	C5	1.585(4)	
C1	C8	1.503(4)		C2	C3	1.538(4)	
C2	C10	1.517(4)		C3	C4	1.548(4)	
C3	C15	1.485(4)		C3	C16	1.472(4)	
C4	C5	1.522(4)		C5	C6	1.497(5)	
C6	C7	1.302(6)		C11	C12	1.500(7)	
C11	C13	1.521(8)		C11	C14	1.514(8)	
C21	C22	1.527(4)		C21	C25	1.577(4)	
C21	C28	1.511(4)		C22	C23	1.549(4)	
C22	C30	1.519(4)		C23	C24	1.547(4)	
C23	C35	1.480(4)		C23	C36	1.486(4)	
C24	C25	1.527(4)		C25	C26	1.474(5)	
C26	C27	1.296(9)		C31	C32	1.523(6)	
C31	C33	1.530(6)		C31	C34	1.499(6)	


Table 6. Bond lengths involving hydrogens (Å)

atom	atom	distance		atom	atom	distance
C1	H1	1.000		C2	H2	1.000	
C4	H4A	0.990		C4	H4B	0.990	
C5	H5	1.000		C6	H6	0.950	
C7	H7A	0.950		C7	H7B	0.950	
C9	H9A	0.980		C9	H9B	0.980	
C9	H9C	0.980		C12	H12A	0.980	
C12	H12B	0.980		C12	H12C	0.980	
C13	H13A	0.980		C13	H13B	0.980	
C13	H13C	0.980		C14	H14A	0.980	
C14	H14B	0.980		C14	H14C	0.980	
C21	H21	1.000		C22	H22	1.000	
C24	H24A	0.990		C24	H24B	0.990	
C25	H25	1.000		C26	H26	0.950	
C27	H27A	0.950		C27	H27B	0.950	
C29	H29A	0.980		C29	H29B	0.980	
C29	H29C	0.980		C32	H32A	0.980	
C32	H32B	0.980		C32	H32C	0.980	
C33	H33A	0.980		C33	H33B	0.980	
C33	H33C	0.980		C34	H34A	0.980	
C34	H34B	0.980		C34	H34C	0.980	


Table 7. Bond angles (o)

atom	atom	atom	angle		atom	atom	atom	angle
C8	O9	C9	115.5(3)		C10	O11	C11	120.8(3)
C28	O29	C29	115.5(3)		C30	O31	C31	121.9(2)
C2	C1	C5	104.8(2)		C2	C1	C8	109.5(2)
C5	C1	C8	114.4(2)		C1	C2	C3	104.4(2)
C1	C2	C10	116.4(2)		C3	C2	C10	113.7(2)
C2	C3	C4	100.9(2)		C2	C3	C15	110.3(2)
C2	C3	C16	112.0(2)		C4	C3	C15	110.0(2)
C4	C3	C16	113.5(2)		C15	C3	C16	109.9(3)
C3	C4	C5	104.7(2)		C1	C5	C4	105.2(2)
C1	C5	C6	116.4(3)		C4	C5	C6	111.5(3)
C5	C6	C7	124.6(4)		O8	C8	O9	123.4(3)
O8	C8	C1	124.4(2)		O9	C8	C1	112.3(2)
O10	C10	O11	127.8(3)		O10	C10	C2	124.0(3)
O11	C10	C2	108.1(3)		O11	C11	C12	109.4(3)
O11	C11	C13	100.8(3)		O11	C11	C14	108.6(3)
C12	C11	C13	111.5(4)		C12	C11	C14	113.9(5)
C13	C11	C14	111.7(5)		N15	C15	C3	175.7(4)
N16	C16	C3	177.1(4)		C22	C21	C25	105.7(2)
C22	C21	C28	112.5(2)		C25	C21	C28	112.0(2)
C21	C22	C23	103.6(2)		C21	C22	C30	115.8(2)
C23	C22	C30	113.7(2)		C22	C23	C24	101.3(2)
C22	C23	C35	114.3(2)		C22	C23	C36	111.7(2)
C24	C23	C35	112.1(2)		C24	C23	C36	110.2(2)
C35	C23	C36	107.3(3)		C23	C24	C25	105.1(2)
C21	C25	C24	105.2(2)		C21	C25	C26	114.5(3)
C24	C25	C26	110.0(3)		C25	C26	C27	136.7(7)
O28	C28	O29	124.0(3)		O28	C28	C21	125.2(3)
O29	C28	C21	110.8(2)		O30	C30	O31	127.1(3)
O30	C30	C22	123.8(3)		O31	C30	C22	109.1(3)
O31	C31	C32	109.4(3)		O31	C31	C33	101.5(3)
O31	C31	C34	109.4(3)		C32	C31	C33	111.8(3)
C32	C31	C34	112.9(3)		C33	C31	C34	111.2(3)
N35	C35	C23	175.9(3)		N36	C36	C23	176.9(3)


Table 8. Bond angles involving hydrogens (o)

atom	atom	atom	angle		atom	atom	atom	angle
C2	C1	H1	109.3		C5	C1	H1	109.3
C8	C1	H1	109.3		C1	C2	H2	107.3
C3	C2	H2	107.3		C10	C2	H2	107.3
C3	C4	H4A	110.8		C3	C4	H4B	110.8
C5	C4	H4A	110.8		C5	C4	H4B	110.8
H4A	C4	H4B	108.9		C1	C5	H5	107.8
C4	C5	H5	107.8		C6	C5	H5	107.8
C5	C6	H6	117.7		C7	C6	H6	117.7
C6	C7	H7A	120.0		C6	C7	H7B	120.0
H7A	C7	H7B	120.0		O9	C9	H9A	109.5
O9	C9	H9B	109.5		O9	C9	H9C	109.5
H9A	C9	H9B	109.5		H9A	C9	H9C	109.5
H9B	C9	H9C	109.5		C11	C12	H12A	109.5
C11	C12	H12B	109.5		C11	C12	H12C	109.5
H12A	C12	H12B	109.5		H12A	C12	H12C	109.5
H12B	C12	H12C	109.5		C11	C13	H13A	109.5
C11	C13	H13B	109.5		C11	C13	H13C	109.5
H13A	C13	H13B	109.5		H13A	C13	H13C	109.5
H13B	C13	H13C	109.5		C11	C14	H14A	109.5
C11	C14	H14B	109.5		C11	C14	H14C	109.5
H14A	C14	H14B	109.5		H14A	C14	H14C	109.5
H14B	C14	H14C	109.5		C22	C21	H21	108.8
C25	C21	H21	108.8		C28	C21	H21	108.8
C21	C22	H22	107.8		C23	C22	H22	107.8
C30	C22	H22	107.8		C23	C24	H24A	110.7
C23	C24	H24B	110.7		C25	C24	H24A	110.7
C25	C24	H24B	110.7		H24A	C24	H24B	108.8
C21	C25	H25	109.0		C24	C25	H25	109.0
C26	C25	H25	109.0		C25	C26	H26	111.6
C27	C26	H26	111.6		C26	C27	H27A	120.0
C26	C27	H27B	120.0		H27A	C27	H27B	120.0
O29	C29	H29A	109.5		O29	C29	H29B	109.5
O29	C29	H29C	109.5		H29A	C29	H29B	109.5
H29A	C29	H29C	109.5		H29B	C29	H29C	109.5
C31	C32	H32A	109.5		C31	C32	H32B	109.5
C31	C32	H32C	109.5		H32A	C32	H32B	109.5
H32A	C32	H32C	109.5		H32B	C32	H32C	109.5


Table 8. Bond angles involving hydrogens (o) (continued)

atom	atom	atom	angle		atom	atom	atom	angle
C31	C33	H33A	109.5		C31	C33	H33B	109.5
C31	C33	H33C	109.5		H33A	C33	H33B	109.5
H33A	C33	H33C	109.5		H33B	C33	H33C	109.5
C31	C34	H34A	109.5		C31	C34	H34B	109.5
C31	C34	H34C	109.5		H34A	C34	H34B	109.5
H34A	C34	H34C	109.5		H34B	C34	H34C	109.5


Table 9. Torsion Angles(o)
	(Those having bond angles > 160 or < 20 degrees are excluded.)

atom1	atom2	atom3	atom4	   angle		atom1	atom2	atom3	atom4	   angle
C9	O9	C8	O8	4.2(5) 		C9	O9	C8	C1	-175.2(3) 
C10	O11	C11	C12	-61.2(4) 		C10	O11	C11	C13	-178.8(2) 
C10	O11	C11	C14	63.6(4) 		C11	O11	C10	O10	-2.9(4) 
C11	O11	C10	C2	175.9(2) 		C29	O29	C28	O28	-1.7(4) 
C29	O29	C28	C21	175.6(3) 		C30	O31	C31	C32	61.2(3) 
C30	O31	C31	C33	179.5(2) 		C30	O31	C31	C34	-62.9(3) 
C31	O31	C30	O30	-0.4(4) 		C31	O31	C30	C22	178.3(2) 
C2	C1	C5	C4	-0.1(3) 		C2	C1	C5	C6	123.9(2) 
C5	C1	C2	C3	26.8(2) 		C5	C1	C2	C10	152.9(2) 
C2	C1	C8	O8	-44.9(3) 		C2	C1	C8	O9	134.6(2) 
C8	C1	C2	C3	149.88(19) 		C8	C1	C2	C10	-84.0(3) 
C5	C1	C8	O8	72.4(3) 		C5	C1	C8	O9	-108.1(2) 
C8	C1	C5	C4	-120.0(2) 		C8	C1	C5	C6	4.0(3) 
C1	C2	C3	C4	-42.7(2) 		C1	C2	C3	C15	73.5(2) 
C1	C2	C3	C16	-163.73(19) 		C1	C2	C10	O10	-135.4(3) 
C1	C2	C10	O11	45.7(3) 		C3	C2	C10	O10	-14.1(4) 
C3	C2	C10	O11	167.1(2) 		C10	C2	C3	C4	-170.51(19) 
C10	C2	C3	C15	-54.3(3) 		C10	C2	C3	C16	68.5(3) 
C2	C3	C4	C5	42.9(2) 		C15	C3	C4	C5	-73.6(3) 
C16	C3	C4	C5	162.8(2) 		C3	C4	C5	C1	-26.5(3) 
C3	C4	C5	C6	-153.6(2) 		C1	C5	C6	C7	129.0(3) 
C4	C5	C6	C7	-110.3(3) 		C22	C21	C25	C24	-4.3(3) 
C22	C21	C25	C26	116.5(2) 		C25	C21	C22	C23	29.3(2) 
C25	C21	C22	C30	154.6(2) 		C22	C21	C28	O28	-28.5(4) 
C22	C21	C28	O29	154.2(2) 		C28	C21	C22	C23	151.87(19) 
C28	C21	C22	C30	-82.9(3) 		C25	C21	C28	O28	90.4(3) 
C25	C21	C28	O29	-86.9(2) 		C28	C21	C25	C24	-127.2(2) 
C28	C21	C25	C26	-6.3(3) 		C21	C22	C23	C24	-42.8(2) 
C21	C22	C23	C35	-163.45(19) 		C21	C22	C23	C36	74.5(2) 
C21	C22	C30	O30	-140.7(3) 		C21	C22	C30	O31	40.5(3) 
C23	C22	C30	O30	-20.8(4) 		C23	C22	C30	O31	160.4(2) 
C30	C22	C23	C24	-169.36(19) 		C30	C22	C23	C35	70.0(3) 
C30	C22	C23	C36	-52.1(3) 		C22	C23	C24	C25	40.4(2) 
C35	C23	C24	C25	162.6(2) 		C36	C23	C24	C25	-78.0(3) 
C23	C24	C25	C21	-22.5(3) 		C23	C24	C25	C26	-146.3(2) 
C21	C25	C26	C27	119.8(4) 		C24	C25	C26	C27	-121.9(5) 


Table 10. Intramolecular contacts less than 3.60 Å

atom	atom	distance		atom	atom	distance
O8	O11	3.396(3)		O8	C2	2.885(4)	
O8	C5	3.200(4)		O8	C6	3.156(4)	
O8	C9	2.634(5)		O9	O11	3.328(3)	
O9	C2	3.533(4)		O9	C5	3.431(4)	
O10	N15	3.472(4)		O10	N16	3.569(4)	
O10	C3	2.847(4)		O10	C11	2.843(5)	
O10	C12	3.019(6)		O10	C14	3.012(6)	
O10	C15	2.927(4)		O10	C16	2.996(4)	
O11	C1	2.812(3)		O11	C8	2.932(4)	
O28	O31	3.238(3)		O28	C22	2.880(4)	
O28	C25	3.306(4)		O28	C26	3.213(5)	
O28	C29	2.643(5)		O29	C25	3.180(4)	
O29	C26	3.233(5)		O30	N36	3.572(4)	
O30	C23	2.870(4)		O30	C31	2.860(4)	
O30	C32	3.027(5)		O30	C34	3.052(5)	
O30	C35	3.044(4)		O30	C36	2.988(4)	
O31	C21	2.778(3)		O31	C28	2.955(4)	
N15	C2	3.452(4)		N15	C4	3.447(5)	
N15	C16	3.450(5)		N16	C2	3.481(4)	
N16	C4	3.513(5)		N16	C15	3.439(5)	
N35	C22	3.579(4)		N35	C24	3.480(4)	
N35	C36	3.371(4)		N36	C22	3.514(4)	
N36	C24	3.449(4)		N36	C35	3.386(4)	
C1	C15	2.987(4)		C4	C7	3.466(6)	
C4	C8	3.582(4)		C5	C15	2.987(5)	
C6	C8	2.873(4)		C8	C10	3.291(4)	
C10	C12	2.977(5)		C10	C14	2.991(6)	
C10	C15	2.950(4)		C10	C16	3.113(4)	
C21	C36	3.009(4)		C22	C26	3.534(5)	
C24	C27	3.583(9)		C25	C36	3.050(4)	
C26	C28	2.761(5)		C28	C30	3.317(4)	
C30	C32	3.014(5)		C30	C34	3.014(5)	
C30	C35	3.184(4)		C30	C36	2.971(4)	


Table 11. Intramolecular contacts less than 3.60 Å involving hydrogens

atom	atom	distance		atom	atom	distance
O8	H1	3.161		O8	H2	2.441	
O8	H6	2.638		O8	H9A	2.684	
O8	H9B	2.519		O8	H9C	3.592	
O9	H1	2.370		O9	H6	3.587	
O9	H13B	3.256		O10	H1	3.582	
O10	H2	2.915		O10	H12A	2.478	
O10	H12B	3.330		O10	H14A	3.376	
O10	H14B	2.439		O11	H1	2.779	
O11	H2	2.613		O11	H12A	2.719	
O11	H12B	2.544		O11	H12C	3.277	
O11	H13A	2.550		O11	H13B	2.419	
O11	H13C	3.191		O11	H14A	2.570	
O11	H14B	2.688		O11	H14C	3.283	
O28	H21	3.135		O28	H22	2.480	
O28	H26	2.783		O28	H29A	2.724	
O28	H29B	2.500		O28	H29C	3.598	
O29	H21	2.407		O29	H25	3.467	
O29	H26	3.424		O30	H22	2.891	
O30	H32A	3.436		O30	H32B	2.430	
O30	H34A	2.423		O30	H34B	3.560	
O31	H21	2.718		O31	H22	2.684	
O31	H32A	2.627		O31	H32B	2.677	
O31	H32C	3.305		O31	H33A	2.501	
O31	H33B	2.508		O31	H33C	3.213	
O31	H34A	2.604		O31	H34B	2.657	
O31	H34C	3.288		N15	H1	3.368	
N15	H4A	3.382		N15	H5	3.319	
N16	H2	3.398		N16	H4B	3.438	
N35	H22	3.532		N35	H24B	3.400	
N36	H21	3.425		N36	H24A	3.334	
N36	H25	3.529		C1	H4A	3.285	
C1	H4B	2.932		C1	H6	2.866	
C2	H4A	3.256		C2	H4B	2.700	
C2	H5	3.089		C3	H1	2.865	
C3	H5	2.828		C4	H1	3.110	
C4	H2	2.608		C4	H6	2.850	
C5	H2	2.853		C5	H7A	2.658	


Table 11. Intramolecular contacts less than 3.60 Å involving hydrogens (continued)

atom	atom	distance		atom	atom	distance
C5	H7B	3.335		C6	H1	3.253	
C6	H4A	2.920		C6	H4B	2.555	
C7	H4A	3.582		C7	H4B	3.598	
C7	H5	2.547		C8	H2	2.493	
C8	H5	3.254		C8	H6	2.722	
C8	H9A	2.624		C8	H9B	2.559	
C8	H9C	3.166		C9	H13B	3.388	
C10	H1	2.672		C10	H12A	2.808	
C10	H12B	3.093		C10	H14A	3.164	
C10	H14B	2.782		C12	H13A	2.613	
C12	H13B	3.328		C12	H13C	2.786	
C12	H14A	3.355		C12	H14B	2.692	
C12	H14C	2.773		C13	H9A	3.383	
C13	H12A	3.340		C13	H12B	2.708	
C13	H12C	2.672		C13	H14A	2.694	
C13	H14B	3.353		C13	H14C	2.719	
C14	H12A	2.661		C14	H12B	3.353	
C14	H12C	2.802		C14	H13A	3.351	
C14	H13B	2.732		C14	H13C	2.684	
C15	H1	2.921		C15	H2	3.320	
C15	H4A	2.609		C15	H4B	3.335	
C15	H5	2.876		C16	H2	2.608	
C16	H4A	2.886		C16	H4B	2.654	
C21	H24A	3.263		C21	H24B	2.962	
C21	H26	2.780		C22	H24A	3.263	
C22	H24B	2.735		C22	H25	3.139	
C22	H26	3.473		C23	H21	2.818	
C23	H25	2.903		C24	H21	3.070	
C24	H22	2.632		C24	H26	2.646	
C25	H22	2.836		C25	H27A	2.851	
C25	H27B	3.384		C26	H21	3.244	
C26	H22	3.538		C26	H24A	2.938	
C26	H24B	2.481		C27	H24B	3.593	
C27	H25	2.744		C28	H22	2.563	
C28	H25	3.181		C28	H26	2.703	
C28	H29A	2.658		C28	H29B	2.527	
C28	H29C	3.162		C30	H21	2.651	


Table 11. Intramolecular contacts less than 3.60 Å involving hydrogens (continued)

atom	atom	distance		atom	atom	distance
C30	H32A	3.229		C30	H32B	2.770	
C30	H34A	2.708		C30	H34B	3.317	
C32	H33A	3.365		C32	H33B	2.695	
C32	H33C	2.751		C32	H34A	2.756	
C32	H34B	3.353		C32	H34C	2.674	
C33	H32A	2.685		C33	H32B	3.366	
C33	H32C	2.758		C33	H34A	3.335	
C33	H34B	2.602		C33	H34C	2.783	
C34	H32A	3.350		C34	H32B	2.711	
C34	H32C	2.729		C34	H33A	2.672	
C34	H33B	3.337		C34	H33C	2.720	
C35	H22	2.670		C35	H24A	2.866	
C35	H24B	2.627		C36	H21	2.904	
C36	H22	3.345		C36	H24A	2.584	
C36	H24B	3.327		C36	H25	3.030	
H1	H2	2.856		H1	H5	2.222	
H2	H4A	3.562		H2	H4B	2.546	
H2	H6	3.514		H4A	H5	2.237	
H4A	H6	3.428		H4A	H7A	3.576	
H4B	H5	2.841		H4B	H6	2.595	
H5	H6	2.870		H5	H7A	2.353	
H5	H7B	3.491		H6	H7A	2.773	
H6	H7B	2.219		H9A	H13A	3.012	
H9A	H13B	2.853		H9C	H13B	3.568	
H12A	H13A	3.533		H12A	H14A	3.541	
H12A	H14B	2.456		H12A	H14C	2.983	
H12B	H13A	2.459		H12B	H13B	3.536	
H12B	H13C	3.139		H12B	H14B	3.546	
H12C	H13A	2.823		H12C	H13B	3.596	
H12C	H13C	2.606		H12C	H14B	3.075	
H12C	H14C	2.697		H13A	H14A	3.596	
H13A	H14C	3.591		H13B	H14A	2.549	
H13B	H14C	3.052		H13C	H14A	2.943	
H13C	H14B	3.586		H13C	H14C	2.524	
H21	H22	2.850		H21	H25	2.226	
H22	H24A	3.588		H22	H24B	2.597	
H22	H26	3.209		H24A	H25	2.241	


Table 11. Intramolecular contacts less than 3.60 Å involving hydrogens (continued)

atom	atom	distance		atom	atom	distance
H24A	H26	3.288		H24B	H25	2.839	
H24B	H26	2.336		H25	H26	2.822	
H25	H27A	2.671		H26	H27A	2.722	
H26	H27B	2.123		H29A	H33A	3.557	
H29A	H33B	3.567		H32A	H33A	3.568	
H32A	H33B	2.483		H32A	H33C	2.999	
H32A	H34C	3.574		H32B	H33B	3.572	
H32B	H34A	2.588		H32B	H34C	2.935	
H32C	H33B	3.024		H32C	H33C	2.621	
H32C	H34A	3.086		H32C	H34B	3.590	
H32C	H34C	2.520		H33A	H34A	3.516	
H33A	H34B	2.403		H33A	H34C	3.081	
H33B	H34B	3.502		H33C	H34B	2.884	
H33C	H34C	2.649		


Table 12. Intermolecular contacts less than 3.60 Å

atom	atom	distance		atom	atom	distance
O8	O28	3.465(3)		O8	C22	3.279(3)	
O8	C23	3.544(4)		O8	C24	3.329(4)	
O8	C35	3.441(4)		O9	C211	3.562(3)	
O10	C322	3.575(5)		O11	O28	3.454(3)	
O28	O8	3.465(3)		O28	O11	3.454(3)	
O28	C2	3.272(3)		O28	C10	3.346(4)	
N15	C63	3.484(5)		N15	C243	3.409(4)	
N16	C73	3.513(6)		N16	C244	3.315(4)	
N16	C254	3.419(5)		N35	C45	3.503(4)	
N35	C55	3.583(4)		N35	C9	3.519(6)	
N35	C146	3.521(6)		N36	C97	3.377(6)	
N36	C128	3.570(6)		C2	O28	3.272(3)	
C4	N353	3.503(4)		C5	N353	3.583(4)	
C6	N155	3.484(5)		C7	N165	3.513(6)	
C9	N35	3.519(6)		C9	N361	3.377(6)	
C10	O28	3.346(4)		C12	N362	3.570(6)	
C14	N359	3.521(6)		C21	O97	3.562(3)	
C22	O8	3.279(3)		C23	O8	3.544(4)	
C24	O8	3.329(4)		C24	N155	3.409(4)	
C24	N1610	3.315(4)		C25	N1610	3.419(5)	
C32	O108	3.575(5)		C35	O8	3.441(4)	


Symmetry Operators:

(1)  X-1,Y,Z		(2)  -X+1,Y+1/2,-Z+2
(3)  -X,Y+1/2,-Z+1		(4)  -X+1,Y+1/2,-Z+1
(5)  -X,Y+1/2-1,-Z+1		(6)  -X,Y+1/2-1,-Z+2
(7)  X+1,Y,Z		(8)  -X+1,Y+1/2-1,-Z+2
(9)  -X,Y+1/2,-Z+2		(10)  -X+1,Y+1/2-1,-Z+1


Table 13. Intermolecular contacts less than 3.60 Å involving hydrogens

atom	atom	distance		atom	atom	distance
O8	H22	2.392		O8	H24B	2.541	
O8	H26	3.140		O9	H211	2.704	
O9	H251	3.102		O10	H7B2	2.880	
O10	H32A3	3.361		O10	H32B3	3.457	
O10	H32C3	3.315		O28	H2	2.434	
O28	H12B	2.986		O29	H14	2.801	
O29	H14A4	3.191		O30	H14A5	3.421	
O30	H14C5	3.498		O30	H29A6	2.786	
N15	H62	2.730		N15	H7B2	3.147	
N15	H24A2	3.224		N15	H24B2	2.757	
N15	H29C1	2.881		N16	H7B2	3.275	
N16	H24A7	2.528		N16	H257	2.710	
N16	H29B	2.957		N16	H32C3	2.944	
N35	H4A8	2.726		N35	H58	2.856	
N35	H7A8	3.440		N35	H9A	3.432	
N35	H9B	2.754		N35	H14C5	2.557	
N35	H27A9	3.411		N35	H33C6	3.527	
N36	H4A9	3.325		N36	H4B9	3.136	
N36	H9B4	3.432		N36	H9C4	2.549	
N36	H12C6	2.854		N36	H27B9	2.754	
N36	H33A6	3.415		C2	H26	3.597	
C4	H9B2	3.394		C5	H27A1	3.369	
C7	H13C10	2.978		C7	H27A1	3.170	
C7	H32C11	3.163		C8	H22	3.420	
C9	H4A8	3.347		C9	H12C5	3.559	
C9	H211	3.232		C12	H9C12	3.436	
C12	H33A	3.423		C12	H34B	3.592	
C13	H32A1	3.481		C13	H33B1	3.458	
C13	H34B	3.487		C14	H29C1	3.558	
C14	H33B1	3.353		C15	H62	3.198	
C15	H7B2	3.067		C15	H9B2	3.504	
C15	H29C1	3.427		C16	H7B2	3.159	
C16	H24A7	3.537		C16	H257	3.510	
C16	H29B	3.529		C16	H32C3	3.522	
C26	H2	3.591		C26	H4B	3.552	
C27	H54	3.296		C27	H7A4	3.356	
C27	H33C10	2.894		C27	H34C10	3.524	


Table 13. Intermolecular contacts less than 3.60 Å involving hydrogens (continued)

atom	atom	distance		atom	atom	distance
C28	H2	3.392		C29	H14	3.462	
C29	H14A4	3.086		C29	H32B3	3.134	
C29	H34A3	2.989		C29	H34C3	3.476	
C32	H7A13	3.451		C32	H7B13	3.547	
C32	H12A6	3.442		C32	H29A6	3.349	
C32	H29B6	3.498		C33	H7A13	3.481	
C33	H27A14	3.543		C34	H13A	3.540	
C34	H29A6	3.417		C34	H29C6	3.293	
C35	H9B	3.241		C35	H14C5	3.191	
C35	H27B9	3.540		C36	H9C4	3.074	
C36	H27B9	2.934		H1	O291	2.801	
H1	C291	3.462		H1	H211	3.501	
H1	H29C1	3.067		H2	O28	2.434	
H2	C26	3.591		H2	C28	3.392	
H2	H22	3.427		H2	H26	2.682	
H4A	N352	2.726		H4A	N367	3.325	
H4A	C92	3.347		H4A	H9B2	2.511	
H4A	H9C2	3.367		H4A	H12C10	3.441	
H4B	N367	3.136		H4B	C26	3.552	
H4B	H26	2.789		H4B	H27B	3.016	
H5	N352	2.856		H5	C271	3.296	
H5	H27A1	2.522		H6	N158	2.730	
H6	C158	3.198		H6	H24B	3.099	
H6	H26	3.447		H7A	N352	3.440	
H7A	C271	3.356		H7A	C3211	3.451	
H7A	C3311	3.481		H7A	H13C10	2.891	
H7A	H27A1	2.431		H7A	H32A11	3.363	
H7A	H32C11	2.775		H7A	H33B11	3.412	
H7A	H33C11	2.800		H7B	O108	2.880	
H7B	N158	3.147		H7B	N168	3.275	
H7B	C158	3.067		H7B	C168	3.159	
H7B	C3211	3.547		H7B	H13C10	2.973	
H7B	H32A11	3.300		H7B	H32C11	2.895	
H9A	N35	3.432		H9A	H12A5	3.434	
H9A	H14B5	3.140		H9A	H14C5	3.202	
H9A	H22	3.404		H9B	N35	2.754	
H9B	N361	3.432		H9B	C48	3.394	


Table 13. Intermolecular contacts less than 3.60 Å involving hydrogens (continued)

atom	atom	distance		atom	atom	distance
H9B	C158	3.504		H9B	C35	3.241	
H9B	H4A8	2.511		H9B	H12C5	3.549	
H9B	H14C5	3.443		H9C	N361	2.549	
H9C	C125	3.436		H9C	C361	3.074	
H9C	H4A8	3.367		H9C	H12A5	3.017	
H9C	H12C5	2.951		H9C	H211	2.770	
H9C	H251	3.586		H12A	C323	3.442	
H12A	H9A12	3.434		H12A	H9C12	3.017	
H12A	H32A3	3.120		H12A	H32B3	2.920	
H12B	O28	2.986		H12B	H33A	2.692	
H12B	H34B	3.047		H12C	N363	2.854	
H12C	C912	3.559		H12C	H4A14	3.441	
H12C	H9B12	3.549		H12C	H9C12	2.951	
H12C	H33A	3.336		H12C	H34B	3.334	
H13A	C34	3.540		H13A	H34A	3.523	
H13A	H34B	2.734		H13B	H32A1	2.840	
H13B	H33B1	2.943		H13C	C714	2.978	
H13C	H7A14	2.891		H13C	H7B14	2.973	
H13C	H32A1	3.281		H13C	H33B1	3.192	
H13C	H34B	3.454		H14A	O291	3.191	
H14A	O3012	3.421		H14A	C291	3.086	
H14A	H29A1	2.786		H14A	H29C1	2.870	
H14A	H33B1	2.743		H14B	H9A12	3.140	
H14B	H29C1	3.367		H14B	H34A12	3.397	
H14C	O3012	3.498		H14C	N3512	2.557	
H14C	C3512	3.191		H14C	H9A12	3.202	
H14C	H9B12	3.443		H14C	H33B1	3.159	
H21	O94	2.704		H21	C94	3.232	
H21	H14	3.501		H21	H9C4	2.770	
H22	O8	2.392		H22	C8	3.420	
H22	H2	3.427		H22	H9A	3.404	
H24A	N158	3.224		H24A	N169	2.528	
H24A	C169	3.537		H24A	H27B9	3.549	
H24A	H29B9	3.347		H24A	H34C10	3.593	
H24B	O8	2.541		H24B	N158	2.757	
H24B	H6	3.099		H25	O94	3.102	
H25	N169	2.710		H25	C169	3.510	


Table 13. Intermolecular contacts less than 3.60 Å involving hydrogens (continued)

atom	atom	distance		atom	atom	distance
H25	H9C4	3.586		H26	O8	3.140	
H26	C2	3.597		H26	H2	2.682	
H26	H4B	2.789		H26	H6	3.447	
H27A	N357	3.411		H27A	C54	3.369	
H27A	C74	3.170		H27A	C3310	3.543	
H27A	H54	2.522		H27A	H7A4	2.431	
H27A	H33C10	2.565		H27B	N367	2.754	
H27B	C357	3.540		H27B	C367	2.934	
H27B	H4B	3.016		H27B	H24A7	3.549	
H27B	H33C10	2.832		H29A	O303	2.786	
H29A	C323	3.349		H29A	C343	3.417	
H29A	H14A4	2.786		H29A	H32B3	2.516	
H29A	H32C3	3.532		H29A	H34A3	2.673	
H29A	H34C3	3.487		H29B	N16	2.957	
H29B	C16	3.529		H29B	C323	3.498	
H29B	H24A7	3.347		H29B	H32B3	2.941	
H29B	H32C3	3.221		H29B	H34A3	3.221	
H29B	H34C3	3.274		H29C	N154	2.881	
H29C	C144	3.558		H29C	C154	3.427	
H29C	C343	3.293		H29C	H14	3.067	
H29C	H14A4	2.870		H29C	H14B4	3.367	
H29C	H32B3	3.554		H29C	H34A3	2.611	
H29C	H34C3	3.102		H32A	O106	3.361	
H32A	C134	3.481		H32A	H7A13	3.363	
H32A	H7B13	3.300		H32A	H12A6	3.120	
H32A	H13B4	2.840		H32A	H13C4	3.281	
H32B	O106	3.457		H32B	C296	3.134	
H32B	H12A6	2.920		H32B	H29A6	2.516	
H32B	H29B6	2.941		H32B	H29C6	3.554	
H32C	O106	3.315		H32C	N166	2.944	
H32C	C713	3.163		H32C	C166	3.522	
H32C	H7A13	2.775		H32C	H7B13	2.895	
H32C	H29A6	3.532		H32C	H29B6	3.221	
H33A	N363	3.415		H33A	C12	3.423	
H33A	H12B	2.692		H33A	H12C	3.336	
H33B	C134	3.458		H33B	C144	3.353	
H33B	H7A13	3.412		H33B	H13B4	2.943	


Table 13. Intermolecular contacts less than 3.60 Å involving hydrogens (continued)

atom	atom	distance		atom	atom	distance
H33B	H13C4	3.192		H33B	H14A4	2.743	
H33B	H14C4	3.159		H33C	N353	3.527	
H33C	C2714	2.894		H33C	H7A13	2.800	
H33C	H27A14	2.565		H33C	H27B14	2.832	
H34A	C296	2.989		H34A	H13A	3.523	
H34A	H14B5	3.397		H34A	H29A6	2.673	
H34A	H29B6	3.221		H34A	H29C6	2.611	
H34B	C12	3.592		H34B	C13	3.487	
H34B	H12B	3.047		H34B	H12C	3.334	
H34B	H13A	2.734		H34B	H13C	3.454	
H34C	C2714	3.524		H34C	C296	3.476	
H34C	H24A14	3.593		H34C	H29A6	3.487	
H34C	H29B6	3.274		H34C	H29C6	3.102	


Symmetry Operators:

(1)  X-1,Y,Z		(2)  -X,Y+1/2,-Z+1
(3)  -X+1,Y+1/2,-Z+2		(4)  X+1,Y,Z
(5)  -X,Y+1/2-1,-Z+2		(6)  -X+1,Y+1/2-1,-Z+2
(7)  -X+1,Y+1/2,-Z+1		(8)  -X,Y+1/2-1,-Z+1
(9)  -X+1,Y+1/2-1,-Z+1		(10)  X,Y,Z-1
(11)  X-1,Y,Z-1		(12)  -X,Y+1/2,-Z+2
(13)  X+1,Y,Z+1		(14)  X,Y,Z+1
