# Supplementary material for: Cooperative Palladium/Isothiourea Catalyzed Enantioselective Formal (3+2) Cycloaddition of Vinylcyclopropanes and α,β‐Unsaturated Esters
Source: Angew Chem Int Ed Engl. 2022 Apr 28;61(25):e202202621. doi: 10.1002/anie.202202621 (PMC9324207; doi:10.1002/anie.202202621)

checkCIF/PLATON report

Structure factors have been supplied for datablock(s) 13

THIS REPORT IS FOR GUIDANCE ONLY. IF USED AS PART OF A REVIEW PROCEDURE FOR PUBLICATION, IT SHOULD NOT REPLACE THE EXPERTISE OF AN EXPERIENCED CRYSTALLOGRAPHIC REFEREE.

No syntax errors found. CIF dictionary Interpreting this report

Datablock: 13

```
Bond precision:   C-C = 0.0048 A          Wavelength=1.54187

Cell:              a=9.560(3)     b=15.563(4)        c=11.591(4)
                   alpha=90       beta=90.427(6)     gamma=90
Temperature:       173 K

                Calculated                  Reported
Volume          1724.5(9)                   1724.5(9)
Space group     P 21                        P 1 21 1
Hall group      P 2yb                       P 2yb
Moiety formula  C16 H20 N2 O4               C16 H20 N2 O4
Sum formula     C16 H20 N2 O4               C16 H20 N2 O4
Mr              304.34                      304.34
Dx,g cm-3       1.172                       1.172
Z               4                           4
Mu (mm-1)       0.699                       0.701
F000            648.0                       648.0
F000'           650.09
h,k,lmax        11,18,13                    11,18,13
Nref            6251[ 3250]                 5516
Tmin,Tmax       0.919,0.932                 0.727,0.932
Tmin'           0.869

Correction method= # Reported T Limits: Tmin=0.727 Tmax=0.932
AbsCorr = MULTI-SCAN

Data completeness= 1.70/0.88       Theta(max)= 67.833

R(reflections)= 0.0556( 5334)                       wR2(reflections)=
                                                    0.1343( 5516)
S = 1.052                  Npar= 406
```

The following ALERTS were generated. Each ALERT has the format
test-name_ALERT_alert-type_alert-level.
Click on the hyperlinks for more details of the test.

Alert level B

```
PLAT220_ALERT_2_B NonSolvent   Resd 2  C   Ueq(max)/Ueq(min) Range        7.5 Ratio
PLAT230_ALERT_2_B Hirshfeld Test Diff for    C26      --C27      .       17.1 s.u.
PLAT242_ALERT_2_B Low    'MainMol' Ueq as Compared to Neighbors of        C26 Check
```

Alert level C

```
DIFMN02_ALERT_2_C  The minimum difference density is < -0.1*ZMAX*0.75
            _refine_diff_density_min given =     -0.630
            Test value =     -0.600
DIFMN03_ALERT_1_C  The minimum difference density is < -0.1*ZMAX*0.75
            The relevant atom site should be identified.
DIFMX02_ALERT_1_C  The maximum difference density is > 0.1*ZMAX*0.75
            The relevant atom site should be identified.
PLAT097_ALERT_2_C Large Reported Max.  (Positive) Residual Density       0.62 eA-3
PLAT098_ALERT_2_C Large Reported Min.  (Negative) Residual Density      -0.63 eA-3
PLAT213_ALERT_2_C Atom C27             has ADP max/min Ratio .....        3.3 prolat
PLAT220_ALERT_2_C NonSolvent   Resd 1  C   Ueq(max)/Ueq(min) Range        3.1 Ratio
PLAT222_ALERT_3_C NonSolvent Resd 2  H   Uiso(max)/Uiso(min) Range        7.4 Ratio
PLAT242_ALERT_2_C Low    'MainMol' Ueq as Compared to Neighbors of        C11 Check
PLAT340_ALERT_3_C Low Bond Precision on  C-C Bonds ..............     0.00475 Ang.
PLAT911_ALERT_3_C Missing FCF Refl Between Thmin & STh/L=    0.600         34 Report
PLAT915_ALERT_3_C No Flack x Check Done: Low Friedel Pair Coverage         77 %
```

Alert level G

```
PLAT002_ALERT_2_G Number of Distance or Angle Restraints on AtSite          2 Note
PLAT172_ALERT_4_G The CIF-Embedded .res File Contains DFIX Records          1 Report
PLAT791_ALERT_4_G Model has Chirality at C1            (Sohnke SpGr)        R Verify
PLAT791_ALERT_4_G Model has Chirality at C2            (Sohnke SpGr)        S Verify
PLAT791_ALERT_4_G Model has Chirality at C5            (Sohnke SpGr)        S Verify
PLAT791_ALERT_4_G Model has Chirality at C21           (Sohnke SpGr)        R Verify
PLAT791_ALERT_4_G Model has Chirality at C22           (Sohnke SpGr)        S Verify
PLAT791_ALERT_4_G Model has Chirality at C25           (Sohnke SpGr)        S Verify
PLAT860_ALERT_3_G Number of Least-Squares Restraints ............           2 Note
PLAT909_ALERT_3_G Percentage of I>2sig(I) Data at Theta(Max) Still        89% Note
PLAT912_ALERT_4_G Missing # of FCF Reflections Above STh/L=  0.600          8 Note
PLAT913_ALERT_3_G Missing # of Very Strong Reflections in FCF ....          1 Note
PLAT933_ALERT_2_G Number of HKL-OMIT Records in Embedded .res File          3 Note
PLAT978_ALERT_2_G Number C-C Bonds with Positive Residual Density.          0 Info
```

0 **ALERT level A** = Most likely a serious problem - resolve or explain
3 **ALERT level B** = A potentially serious problem, consider carefully
12 **ALERT level C** = Check. Ensure it is not caused by an omission or oversight
14 **ALERT level G** = General information/check it is not something unexpected

2 ALERT type 1 CIF construction/syntax error, inconsistent or missing data

```
12 ALERT type 2 Indicator that the structure model may be wrong or deficient
 7 ALERT type 3 Indicator that the structure quality may be low
 8 ALERT type 4 Improvement, methodology, query or suggestion
 0 ALERT type 5 Informative message, check
```

It is advisable to attempt to resolve as many as possible of the alerts in all categories. Often the minor alerts point to easily fixed oversights, errors and omissions in your CIF or refinement strategy, so attention to these fine details can be worthwhile. In order to resolve some of the more serious problems it may be necessary to carry out additional measurements or structure refinements. However, the purpose of your study may justify the reported deviations and the more serious of these should normally be commented upon in the discussion or experimental section of a paper or in the "special_details" fields of the CIF. checkCIF was carefully designed to identify outliers and unusual parameters, but every test has its limitations and alerts that are not important in a particular case may appear. Conversely, the absence of alerts does not guarantee there are no aspects of the results needing attention. It is up to the individual to critically assess their own results and, if necessary, seek expert advice.

Publication of your CIF in IUCr journals

A basic structural check has been run on your CIF. These basic checks will be run on all CIFs submitted for publication in IUCr journals (*Acta Crystallographica*, *Journal of Applied Crystallography*, *Journal of Synchrotron Radiation*); however, if you intend to submit to *Acta Crystallographica Section C* or *E* or *IUCrData*, you should make sure that full publication checks are run on the final version of your CIF prior to submission.

Publication of your CIF in other journals

Please refer to the *Notes for Authors* of the relevant journal for any special instructions relating to CIF submission.

PLATON version of 20/01/2022; check.def file version of 19/01/2022

Datablock 13 - ellipsoid plot

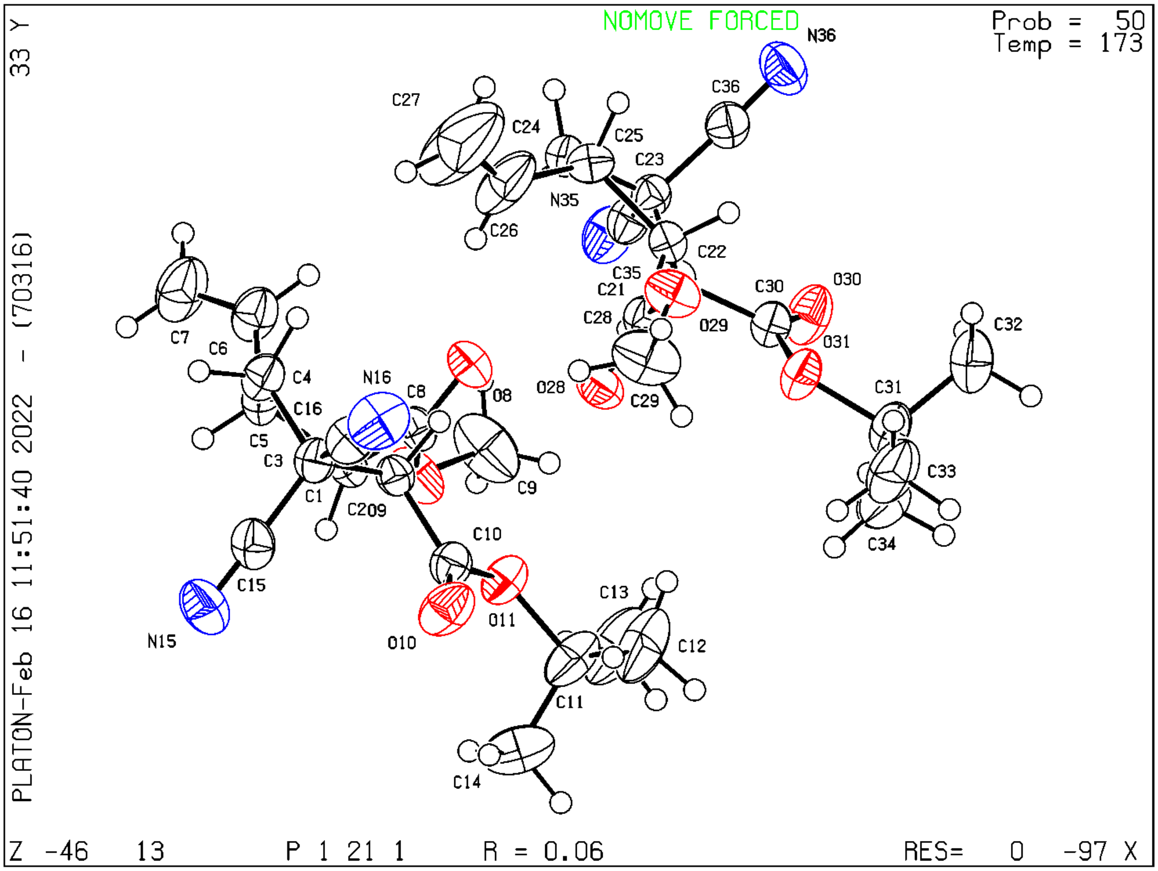

Supplement: Supplementary file 3 — Supporting Information [file ANIE-61-0-s009.pdf]
