# Supplementary material for: Cooperative Palladium/Isothiourea Catalyzed Enantioselective Formal (3+2) Cycloaddition of Vinylcyclopropanes and α,β‐Unsaturated Esters
Source: Angew Chem Int Ed Engl. 2022 Apr 28;61(25):e202202621. doi: 10.1002/anie.202202621 (PMC9324207; doi:10.1002/anie.202202621)
Supplement: Supplementary file 5 — Supporting Information [file ANIE-61-0-s005.rtf]

Data Collection

	A colorless prism crystal of C11H10F3IN2O having approximate dimensions of 0.340 x 0.190 x 0.170 mm was mounted in a loop. All measurements were made on a Rigaku SCX mini diffractometer using graphite monochromated Mo-Ka radiation.


	Cell constants and an orientation matrix for data collection corresponded to a primitive triclinic cell with dimensions:

           a  =    7.6910(5) Å        a  =   79.079(6)o
           b  =   11.5327(8) Å        b  =   79.774(6)o
           c  =   11.9797(8) Å        g  =   73.365(5)o
           V  =  991.04(12) Å3

For Z = 3 and F.W. = 370.11, the calculated density is 1.860 g/cm3. Based on a statistical analysis of intensity distribution, and the successful solution and refinement of the structure, the space group was determined to be:

P1 (#1) 


	The data were collected at a temperature of -100 + 1oC to a maximum 2q value of 55.0o. 

Data Reduction

	Of the 9863 reflections were collected, where 8424 were unique (Rint = 0.0252); equivalent reflections were merged. Data were collected and processed using CrystalClear (Rigaku). 1 

	The linear absorption coefficient, m, for Mo-Ka radiation is 24.495 cm-1. An empirical absorption correction was applied which resulted in transmission factors ranging from 0.531 to 0.659. The data were corrected for Lorentz and polarization effects. 


Structure Solution and Refinement

	The structure was solved by direct methods2 and expanded using Fourier techniques. The non-hydrogen atoms were refined anisotropically. Hydrogen atoms were refined using the riding model. The final cycle of full-matrix least-squares refinement3 on F2 was based on 8424 observed reflections and 487 variable parameters and converged (largest parameter shift was 0.00 times its esd) with unweighted and weighted agreement factors of: 

R1 = S ||Fo| - |Fc|| / S |Fo| = 0.0340 


wR2 = [ S ( w (Fo2 - Fc2)2 )/ S w(Fo2)2]1/2 = 0.0886 


	The goodness of fit4 was 1.05. Unit weights were used.  The maximum and minimum peaks on the final difference Fourier map corresponded to 0.70 and -1.03 e/Å3, respectively. The final Flack parameter 5 was 0.025(11). 

	Neutral atom scattering factors were taken from International Tables for Crystallography (IT), Vol. C, Table 6.1.1.4 6.  Anomalous dispersion effects were included in Fcalc7; the values for Df' and Df" were those of Creagh and McAuley8. The values for the mass attenuation coefficients are those of Creagh and Hubbell9. All calculations were performed using the CrystalStructure10 crystallographic software package except for refinement, which was performed using SHELXL Version 2018/311.


References 


(1) CrystalClear: Data Collection and Processing Software, Rigaku Corporation (1998-2015). Tokyo 196-8666, Japan.

(2) SHELXT Version 2018/2: Sheldrick, G. M. (2014). Acta Cryst. A70, C1437.

(3) Least Squares function minimized: (SHELXL Version 2018/3)

		Sw(Fo2-Fc2)2     where w = Least Squares weights.

(4) Goodness of fit is defined as:

 	 	 [Sw(Fo2-Fc2)2/(No-Nv)]1/2

	 	 where:	 No  = number of observations
	 	 	 	 Nv  = number of variables 


(5) Parsons, S., Flack, H.D. and Wagner, T. Acta Cryst. B69 (2013) 249-259.

(6) International Tables for Crystallography, Vol.C (1992). Ed. A.J.C. Wilson, Kluwer Academic Publishers, Dordrecht, Netherlands, Table 6.1.1.4, pp. 572. 

(7) Ibers, J. A. & Hamilton, W. C.; Acta Crystallogr., 17, 781 (1964). 

(8) Creagh, D. C. & McAuley, W.J .; "International Tables for Crystallography", Vol C, (A.J.C. Wilson, ed.), Kluwer Academic Publishers, Boston, Table 4.2.6.8, pages 219-222 (1992). 

(9) Creagh, D. C. & Hubbell, J.H..; "International Tables for Crystallography", Vol C, (A.J.C. Wilson, ed.), Kluwer Academic Publishers, Boston, Table 4.2.4.3, pages 200-206 (1992). 

(10) CrystalStructure 4.3: Crystal Structure Analysis Package, Rigaku Corporation (2000-2019). Tokyo 196-8666, Japan.

(11) SHELXL Version 2018/3: Sheldrick, G. M. (2008). Acta Cryst. A64, 112-122.


EXPERIMENTAL DETAILS 


A. Crystal Data 


Empirical Formula	C11H10F3IN2O

Formula Weight	370.11

Crystal Color, Habit	colorless, prism

Crystal Dimensions	0.340 X 0.190 X 0.170 mm

Crystal System	triclinic

Lattice Type	Primitive

Lattice Parameters	a =   7.6910(5) Å
	b =  11.5327(8) Å
	c =  11.9797(8) Å
	a =  79.079(6) o
	b =  79.774(6) o
	g =  73.365(5) o
	V = 991.04(12) Å3

Space Group	P1 (#1)

Z value	3

Dcalc	1.860 g/cm3

F000	534.00

m(MoKa)	24.495 cm-1

B. Intensity Measurements 


Diffractometer	SCX mini

Radiation	MoKa (l = 0.71075 Å)
	graphite monochromated

Voltage, Current	50kV, 40mA

Temperature	-100.0oC

Detector Aperture	75.0 mm (diameter)

Data Images	540 exposures

Pixel Size	0.073 mm

2qmax	55.0o

No. of Reflections Measured	Total: 9863
	Unique: 8424 (Rint = 0.0252)
	Parsons quotients (Flack x parameter): 3245

Corrections	Lorentz-polarization
		Absorption
		(trans. factors: 0.531 - 0.659)

C. Structure Solution and Refinement 


Structure Solution	Direct Methods (SHELXT Version 2018/2)

Refinement	Full-matrix least-squares on F2

Function Minimized	S w (Fo2 - Fc2)2 

Least Squares Weights	w = 1/ [ s2(Fo2) + (0.0501 . P)2 
	 + 0.0000 .  P ]
	 where P = (Max(Fo2,0) + 2Fc2)/3

2qmax cutoff	55.0o

Anomalous Dispersion	All non-hydrogen atoms

No. Observations (All reflections)	8424

No. Variables	487

Reflection/Parameter Ratio	17.30

Residuals: R1 (I>2.00s(I))	0.0340

Residuals: R (All reflections)	0.0400

Residuals: wR2 (All reflections)	0.0886

Goodness of Fit Indicator	1.046

Flack parameter (Parsons' quotients = 3245)	0.025(11)

Max Shift/Error in Final Cycle	0.001

Maximum peak in Final Diff. Map	0.70 e/Å3

Minimum peak in Final Diff. Map	-1.03 e/Å3


Table 1. Atomic coordinates and Biso/Beq

atom	   x	   y	   z	 Beq
I7    	-0.25087(9)	-0.05418(7)	 1.04321(5)	 5.523(17)
I17   	 1.25075(6)	 0.62457(4)	 0.03308(5)	 4.114(14)
I27   	 0.64025(5)	 0.79561(4)	 0.82245(4)	 3.519(12)
F1    	-0.0637(8)	 0.4267(6)	 0.5752(6)	 6.24(15)
F2    	 0.2042(11)	 0.3429(8)	 0.4989(5)	 7.23(17)
F3    	 0.1645(11)	 0.4894(6)	 0.5950(7)	 7.28(18)
F4    	 0.5918(10)	 0.1555(6)	 0.3062(6)	 6.03(14)
F5    	 0.4469(7)	 0.3429(6)	 0.2865(5)	 5.05(12)
F6    	 0.5220(8)	 0.2480(6)	 0.1405(5)	 4.97(11)
F7    	-0.0642(8)	 0.7469(8)	 0.4643(6)	 6.73(16)
F8    	-0.0188(10)	 0.8268(8)	 0.2873(6)	 7.6(2)
F9    	 0.1504(8)	 0.6526(6)	 0.3473(5)	 5.12(11)
O2    	-0.1437(7)	 0.1757(4)	 0.8555(5)	 3.23(9)
O12   	 0.9722(7)	 0.3740(4)	-0.0321(4)	 2.75(8)
O22   	 0.3078(7)	 0.8646(5)	 0.6461(5)	 3.36(9)
N9    	 0.4585(11)	 0.4304(7)	 0.7774(8)	 4.66(15)
N10   	 0.5625(11)	 0.1348(8)	 0.5799(8)	 4.77(16)
N19   	 0.6135(11)	 0.4699(7)	 0.4364(6)	 4.29(14)
N20   	 0.9793(11)	 0.1125(7)	 0.4466(6)	 4.24(14)
N29   	 0.5245(10)	 0.6942(6)	 0.1915(6)	 3.74(12)
N30   	 0.1943(11)	 1.0578(7)	 0.1698(6)	 4.52(15)
C1    	 0.0117(10)	 0.0880(6)	 0.9001(6)	 2.87(11)
C3    	-0.1101(11)	 0.1821(8)	 0.7341(8)	 3.68(14)
C3a   	 0.0919(9)	 0.1778(6)	 0.7040(6)	 2.69(11)
C4    	 0.1365(9)	 0.2996(6)	 0.7016(6)	 2.55(10)
C5    	 0.3360(9)	 0.2558(6)	 0.7331(6)	 2.58(11)
C6    	 0.3083(9)	 0.1601(6)	 0.8406(6)	 2.71(11)
C6a   	 0.1779(9)	 0.0946(6)	 0.8085(5)	 2.20(10)
C7    	-0.0136(12)	-0.0395(8)	 0.9210(8)	 3.98(15)
C8    	 0.1121(13)	 0.3883(9)	 0.5919(8)	 4.55(18)
C9    	 0.4055(10)	 0.3539(7)	 0.7587(7)	 3.45(13)
C10   	 0.4657(10)	 0.1923(8)	 0.6428(7)	 3.26(13)
C11   	 1.0518(8)	 0.4367(5)	 0.0300(6)	 2.34(10)
C13   	 0.7792(9)	 0.4079(6)	-0.0014(6)	 2.64(11)
C13a  	 0.7523(8)	 0.4160(5)	 0.1264(5)	 2.21(10)
C14   	 0.7603(9)	 0.2938(5)	 0.2075(6)	 2.26(10)
C15   	 0.8402(8)	 0.3125(6)	 0.3103(5)	 2.26(10)


Table 1. Atomic coordinates and Biso/Beq (continued)

atom	   x	   y	   z	 Beq
C16a  	 0.9236(9)	 0.4539(5)	 0.1424(6)	 2.30(10)
C16   	 1.0004(9)	 0.3678(7)	 0.2482(6)	 2.75(11)
C17   	 1.0727(10)	 0.5559(6)	-0.0420(7)	 3.06(12)
C18   	 0.5789(11)	 0.2613(8)	 0.2352(7)	 3.67(14)
C19   	 0.7092(10)	 0.3997(6)	 0.3821(6)	 2.83(11)
C20   	 0.9122(10)	 0.1994(7)	 0.3875(6)	 3.07(12)
C21   	 0.4941(9)	 0.8276(6)	 0.5956(6)	 2.62(11)
C23a  	 0.3028(9)	 0.7471(5)	 0.5055(5)	 2.34(10)
C23   	 0.2194(10)	 0.7785(8)	 0.6273(6)	 3.36(13)
C24   	 0.1910(8)	 0.8249(7)	 0.4098(6)	 2.68(11)
C25   	 0.3322(9)	 0.8650(6)	 0.3126(6)	 2.55(10)
C26a  	 0.4841(8)	 0.7855(5)	 0.4838(6)	 2.21(10)
C26   	 0.4631(10)	 0.8929(6)	 0.3841(6)	 2.57(10)
C27   	 0.6070(10)	 0.7271(7)	 0.6743(6)	 3.07(12)
C28   	 0.0643(12)	 0.7621(10)	 0.3765(8)	 4.50(18)
C29   	 0.4383(9)	 0.7655(6)	 0.2454(6)	 2.53(11)
C30   	 0.2522(11)	 0.9722(6)	 0.2319(6)	 3.18(12)

Beq = 8/3 p2(U11(aa*)2 + U22(bb*)2 + U33(cc*)2 + 2U12(aa*bb*)cos g + 2U13(aa*cc*)cos b + 2U23(bb*cc*)cos a)


Table 2. Atomic coordinates and Biso involving hydrogen atoms

atom	   x	   y	   z	 Biso
H1    	 0.03094	 0.10991	 0.97300	 3.450
H3    	 0.14535	 0.14272	 0.63140	 3.222
H3A   	-0.18660	 0.25910	 0.69656	 4.412
H3B   	-0.13644	 0.11204	 0.70999	 4.412
H4    	 0.05533	 0.34047	 0.76567	 3.055
H6    	 0.24612	 0.01154	 0.78918	 2.643
H6A   	 0.42629	 0.10138	 0.85655	 3.257
H6B   	 0.25228	 0.20086	 0.90891	 3.257
H7A   	 0.09656	-0.09797	 0.94981	 4.776
H7B   	-0.02673	-0.06174	 0.84762	 4.776
H11   	 1.17495	 0.38418	 0.04724	 2.802
H13   	 0.63747	 0.47936	 0.14777	 2.652
H13A  	 0.72378	 0.34550	-0.01593	 3.163
H13B  	 0.72337	 0.48776	-0.04531	 3.163
H14   	 0.85151	 0.22727	 0.16943	 2.711
H16   	 0.88949	 0.54125	 0.15529	 2.763
H16A  	 1.03494	 0.41393	 0.29871	 3.300
H16B  	 1.10900	 0.30266	 0.22450	 3.300
H17A  	 0.95159	 0.61596	-0.04417	 3.671
H17B  	 1.12554	 0.54232	-0.12156	 3.671
H21   	 0.54756	 0.89958	 0.57780	 3.139
H23   	 0.32580	 0.65774	 0.50304	 2.802
H23A  	 0.08556	 0.81446	 0.63122	 4.035
H23B  	 0.24332	 0.70423	 0.68531	 4.035
H24   	 0.11244	 0.90053	 0.44031	 3.214
H26   	 0.59162	 0.71598	 0.46494	 2.655
H26A  	 0.58271	 0.89453	 0.33771	 3.085
H26B  	 0.40831	 0.97214	 0.41288	 3.085
H27A  	 0.72878	 0.69351	 0.63219	 3.682
H27B  	 0.54588	 0.65996	 0.69937	 3.682


Table 3. Anisotropic displacement parameters

atom	  U11	  U22	  U33	  U12	  U13	  U23
I7    	0.0795(4)	0.1070(5)	0.0373(3)	-0.0631(4)	-0.0086(3)	 0.0163(3)
I17   	0.0413(3)	0.0331(3)	0.0844(4)	-0.0124(2)	-0.0106(3)	-0.0085(2)
I27   	0.0422(3)	0.0491(3)	0.0421(3)	-0.0124(2)	-0.0183(2)	 0.0068(2)
F1    	0.059(3)	0.078(4)	0.092(5)	-0.019(3)	-0.043(3)	 0.037(3)
F2    	0.104(5)	0.125(6)	0.029(3)	-0.027(4)	 0.002(3)	 0.013(3)
F3    	0.117(5)	0.076(4)	0.097(5)	-0.065(4)	-0.047(4)	 0.048(4)
F4    	0.096(4)	0.071(4)	0.073(4)	-0.057(3)	-0.012(3)	 0.019(3)
F5    	0.032(2)	0.096(4)	0.067(4)	-0.022(3)	 0.008(2)	-0.024(3)
F6    	0.073(3)	0.081(4)	0.056(3)	-0.049(3)	-0.019(3)	-0.006(3)
F7    	0.038(3)	0.147(6)	0.075(4)	-0.047(3)	 0.003(3)	 -0.001(4)
F8    	0.075(4)	0.146(7)	0.075(4)	-0.051(4)	-0.047(3)	 0.037(4)
F9    	0.077(4)	0.080(4)	0.058(3)	-0.051(3)	-0.009(3)	-0.014(3)
O2    	0.030(2)	0.033(2)	0.053(3)	-0.0045(18)	 0.002(2)	 -0.002(2)
O12   	0.036(2)	0.035(2)	0.035(2)	-0.007(2)	-0.0047(19)	-0.010(2)
O22   	0.040(3)	0.042(3)	0.043(3)	 0.004(2)	-0.011(2)	-0.017(2)
N9    	0.050(4)	0.058(4)	0.079(5)	-0.026(3)	 0.002(4)	-0.025(4)
N10   	0.051(4)	0.072(5)	0.063(5)	-0.022(4)	 0.015(4)	-0.033(4)
N19   	0.059(4)	0.048(4)	0.044(4)	 0.008(3)	-0.004(3)	-0.014(3)
N20   	0.056(4)	0.044(4)	0.040(4)	 0.010(3)	-0.003(3)	 0.005(3)
N29   	0.051(4)	0.038(3)	0.052(4)	-0.007(3)	 0.001(3)	-0.018(3)
N30   	0.065(5)	0.045(4)	0.043(4)	 0.007(3)	-0.007(3)	 0.005(3)
C1    	0.043(4)	0.036(3)	0.029(3)	-0.012(3)	 -0.001(3)	-0.005(3)
C3    	0.037(4)	0.051(4)	0.056(5)	-0.020(3)	-0.011(3)	 -0.002(4)
C3a   	0.031(3)	0.039(3)	0.036(3)	-0.010(3)	-0.005(3)	-0.011(3)
C4    	0.030(3)	0.035(3)	0.031(3)	-0.008(3)	-0.008(3)	 -0.000(3)
C5    	0.028(3)	0.037(3)	0.036(3)	-0.012(3)	-0.003(3)	-0.009(3)
C6    	0.029(3)	0.035(3)	0.035(3)	 0.000(2)	-0.007(3)	-0.006(3)
C6a   	0.033(3)	0.029(3)	0.021(3)	-0.006(2)	 -0.001(2)	-0.007(2)
C7    	0.051(4)	0.045(4)	0.058(5)	-0.024(4)	-0.005(4)	 0.003(4)
C8    	0.058(5)	0.065(5)	0.052(5)	-0.031(4)	-0.019(4)	 0.021(4)
C9    	0.036(4)	0.046(4)	0.053(5)	-0.016(3)	 -0.001(3)	-0.012(4)
C10   	0.034(4)	0.055(4)	0.041(4)	-0.022(3)	 0.004(3)	-0.016(4)
C11   	0.027(3)	0.027(3)	0.032(3)	-0.003(2)	-0.003(2)	-0.004(2)
C13   	0.033(3)	0.036(3)	0.028(3)	-0.005(3)	-0.006(3)	-0.003(3)
C13a  	0.026(3)	0.020(3)	0.032(3)	 0.001(2)	-0.003(2)	 0.000(2)
C14   	0.031(3)	0.022(3)	0.031(3)	-0.004(2)	 -0.001(2)	-0.005(2)
C15   	0.026(3)	0.029(3)	0.028(3)	-0.002(2)	 -0.002(2)	-0.006(2)


Table 3. Anisotropic displacement parameters (continued)

atom	  U11	  U22	  U33	  U12	  U13	  U23
C16a  	0.034(3)	0.020(3)	0.032(3)	-0.004(2)	-0.006(3)	-0.005(2)
C16   	0.027(3)	0.048(4)	0.029(3)	-0.011(3)	-0.004(2)	 -0.001(3)
C17   	0.034(3)	0.038(3)	0.041(4)	-0.011(3)	-0.004(3)	 0.004(3)
C18   	0.048(4)	0.054(4)	0.043(4)	-0.027(4)	-0.005(3)	 0.000(4)
C19   	0.043(4)	0.033(3)	0.029(3)	 -0.003(3)	-0.008(3)	-0.006(3)
C20   	0.040(4)	0.037(4)	0.030(3)	 0.001(3)	 -0.001(3)	-0.003(3)
C21   	0.039(3)	0.026(3)	0.035(3)	-0.009(3)	-0.012(3)	 0.001(3)
C23a  	0.031(3)	0.024(3)	0.032(3)	-0.007(2)	-0.005(2)	 0.000(2)
C23   	0.032(3)	0.061(5)	0.034(4)	-0.012(3)	 -0.001(3)	-0.007(3)
C24   	0.024(3)	0.045(4)	0.030(3)	-0.010(3)	 0.001(2)	 -0.001(3)
C25   	0.033(3)	0.028(3)	0.032(3)	-0.006(2)	 0.000(3)	-0.004(3)
C26a  	0.026(3)	0.020(3)	0.037(3)	-0.005(2)	-0.004(2)	-0.003(2)
C26   	0.040(3)	0.023(3)	0.036(3)	-0.010(2)	-0.007(3)	 -0.002(2)
C27   	0.037(3)	0.035(3)	0.041(4)	-0.006(3)	-0.008(3)	 0.001(3)
C28   	0.042(4)	0.086(7)	0.047(5)	-0.030(4)	-0.018(4)	 0.010(4)
C29   	0.032(3)	0.028(3)	0.033(3)	-0.004(3)	-0.003(3)	-0.004(3)
C30   	0.043(4)	0.036(4)	0.035(4)	 0.000(3)	 -0.001(3)	-0.008(3)


The general temperature factor expression: exp(-2p2(a*2U11h2 + b*2U22k2 + c*2U33l2 + 2a*b*U12hk + 2a*c*U13hl + 2b*c*U23kl))


Table 4. Fragment Analysis


fragment: 1  
	I(7)	F(1)	F(2)	F(3)	O(2)
	N(9)	N(10)	C(1)	C(3)	C(3a)
	C(4)	C(5)	C(6)	C(6a)	C(7)
	C(8)	C(9)	C(10)


fragment: 2  
	I(17)	F(4)	F(5)	F(6)	O(12)
	N(19)	N(20)	C(11)	C(13)	C(13a)
	C(14)	C(15)	C(16a)	C(16)	C(17)
	C(18)	C(19)	C(20)


fragment: 3  
	I(27)	F(7)	F(8)	F(9)	O(22)
	N(29)	N(30)	C(21)	C(23a)	C(23)
	C(24)	C(25)	C(26a)	C(26)	C(27)
	C(28)	C(29)	C(30)


Table 5. Bond lengths (Å)

atom	atom	distance		atom	atom	distance
I7	C7	2.154(9)		I17	C17	2.154(9)	
I27	C27	2.156(9)		F1	C8	1.336(12)	
F2	C8	1.321(11)		F3	C8	1.346(14)	
F4	C18	1.338(10)		F5	C18	1.322(9)	
F6	C18	1.335(12)		F7	C28	1.332(10)	
F8	C28	1.332(11)		F9	C28	1.327(12)	
O2	C1	1.437(8)		O2	C3	1.422(10)	
O12	C11	1.439(10)		O12	C13	1.421(8)	
O22	C21	1.429(8)		O22	C23	1.424(12)	
N9	C9	1.144(14)		N10	C10	1.126(11)	
N19	C19	1.144(10)		N20	C20	1.156(9)	
N29	C29	1.127(9)		N30	C30	1.146(9)	
C1	C6a	1.540(9)		C1	C7	1.506(12)	
C3	C3a	1.520(11)		C3a	C4	1.533(11)	
C3a	C6a	1.549(9)		C4	C5	1.562(10)	
C4	C8	1.510(11)		C5	C6	1.556(9)	
C5	C9	1.481(13)		C5	C10	1.483(10)	
C6	C6a	1.551(11)		C11	C16a	1.532(9)	
C11	C17	1.512(9)		C13	C13a	1.525(10)	
C13a	C14	1.545(8)		C13a	C16a	1.554(10)	
C14	C15	1.545(10)		C14	C18	1.512(12)	
C15	C16	1.556(10)		C15	C19	1.485(9)	
C15	C20	1.474(9)		C16a	C16	1.548(9)	
C21	C26a	1.529(11)		C21	C27	1.513(9)	
C23a	C23	1.551(10)		C23a	C24	1.542(9)	
C23a	C26a	1.546(10)		C24	C25	1.544(9)	
C24	C28	1.511(15)		C25	C26	1.564(12)	
C25	C29	1.487(9)		C25	C30	1.471(9)	
C26a	C26	1.543(8)		


Table 6. Bond lengths involving hydrogens (Å)

atom	atom	distance		atom	atom	distance
C1	H1	1.000		C3	H3A	0.990	
C3	H3B	0.990		C3a	H3	1.000	
C4	H4	1.000		C6	H6A	0.990	
C6	H6B	0.990		C6a	H6	1.000	
C7	H7A	0.990		C7	H7B	0.990	
C11	H11	1.000		C13	H13A	0.990	
C13	H13B	0.990		C13a	H13	1.000	
C14	H14	1.000		C16a	H16	1.000	
C16	H16A	0.990		C16	H16B	0.990	
C17	H17A	0.990		C17	H17B	0.990	
C21	H21	1.000		C23a	H23	1.000	
C23	H23A	0.990		C23	H23B	0.990	
C24	H24	1.000		C26a	H26	1.000	
C26	H26A	0.990		C26	H26B	0.990	
C27	H27A	0.990		C27	H27B	0.990	


Table 7. Bond angles (o)

atom	atom	atom	angle		atom	atom	atom	angle
C1	O2	C3	106.8(5)		C11	O12	C13	108.3(5)
C21	O22	C23	106.9(5)		O2	C1	C6a	106.4(5)
O2	C1	C7	111.1(7)		C6a	C1	C7	109.5(6)
O2	C3	C3a	104.5(7)		C3	C3a	C4	114.6(6)
C3	C3a	C6a	103.4(6)		C4	C3a	C6a	104.0(6)
C3a	C4	C5	101.9(5)		C3a	C4	C8	115.7(7)
C5	C4	C8	115.0(6)		C4	C5	C6	99.4(5)
C4	C5	C9	114.4(6)		C4	C5	C10	111.5(6)
C6	C5	C9	112.5(7)		C6	C5	C10	109.0(6)
C9	C5	C10	109.6(6)		C5	C6	C6a	104.3(6)
C1	C6a	C3a	103.7(5)		C1	C6a	C6	113.6(6)
C3a	C6a	C6	106.1(5)		I7	C7	C1	111.7(5)
F1	C8	F2	106.6(9)		F1	C8	F3	106.2(7)
F1	C8	C4	111.0(7)		F2	C8	F3	106.9(8)
F2	C8	C4	114.2(7)		F3	C8	C4	111.5(9)
N9	C9	C5	179.3(9)		N10	C10	C5	174.0(9)
O12	C11	C16a	105.8(5)		O12	C11	C17	109.5(6)
C16a	C11	C17	113.4(5)		O12	C13	C13a	104.7(6)
C13	C13a	C14	115.6(6)		C13	C13a	C16a	103.5(5)
C14	C13a	C16a	104.8(5)		C13a	C14	C15	102.5(6)
C13a	C14	C18	113.4(5)		C15	C14	C18	116.0(6)
C14	C15	C16	101.3(5)		C14	C15	C19	113.5(5)
C14	C15	C20	114.9(6)		C16	C15	C19	109.9(6)
C16	C15	C20	109.6(5)		C19	C15	C20	107.5(5)
C11	C16a	C13a	104.7(6)		C11	C16a	C16	113.8(5)
C13a	C16a	C16	106.2(5)		C15	C16	C16a	104.4(5)
I17	C17	C11	108.4(5)		F4	C18	F5	105.7(7)
F4	C18	F6	107.0(8)		F4	C18	C14	111.4(7)
F5	C18	F6	107.6(7)		F5	C18	C14	113.7(8)
F6	C18	C14	111.1(6)		N19	C19	C15	177.1(9)
N20	C20	C15	175.7(9)		O22	C21	C26a	104.6(6)
O22	C21	C27	111.4(5)		C26a	C21	C27	112.2(5)
C23	C23a	C24	114.0(5)		C23	C23a	C26a	103.4(6)
C24	C23a	C26a	105.6(5)		O22	C23	C23a	105.3(6)
C23a	C24	C25	105.6(5)		C23a	C24	C28	113.3(6)
C25	C24	C28	116.3(6)		C24	C25	C26	100.5(5)
C24	C25	C29	113.2(6)		C24	C25	C30	113.8(5)


Table 7. Bond angles (o) (continued)

atom	atom	atom	angle		atom	atom	atom	angle
C26	C25	C29	108.7(5)		C26	C25	C30	112.3(6)
C29	C25	C30	108.2(6)		C21	C26a	C23a	104.5(5)
C21	C26a	C26	111.3(5)		C23a	C26a	C26	106.0(5)
C25	C26	C26a	103.3(6)		I27	C27	C21	110.8(5)
F7	C28	F8	108.0(7)		F7	C28	F9	107.5(9)
F7	C28	C24	109.8(8)		F8	C28	F9	106.4(9)
F8	C28	C24	112.0(9)		F9	C28	C24	112.9(7)
N29	C29	C25	176.2(8)		N30	C30	C25	177.5(10)


Table 8. Bond angles involving hydrogens (o)

atom	atom	atom	angle		atom	atom	atom	angle
O2	C1	H1	109.9		C6a	C1	H1	109.9
C7	C1	H1	109.9		O2	C3	H3A	110.9
O2	C3	H3B	110.8		C3a	C3	H3A	110.8
C3a	C3	H3B	110.8		H3A	C3	H3B	108.9
C3	C3a	H3	111.5		C4	C3a	H3	111.5
C6a	C3a	H3	111.5		C3a	C4	H4	108.0
C5	C4	H4	108.0		C8	C4	H4	108.0
C5	C6	H6A	110.9		C5	C6	H6B	110.9
C6a	C6	H6A	110.9		C6a	C6	H6B	110.9
H6A	C6	H6B	108.9		C1	C6a	H6	111.0
C3a	C6a	H6	111.0		C6	C6a	H6	111.0
I7	C7	H7A	109.3		I7	C7	H7B	109.3
C1	C7	H7A	109.3		C1	C7	H7B	109.3
H7A	C7	H7B	107.9		O12	C11	H11	109.3
C16a	C11	H11	109.3		C17	C11	H11	109.3
O12	C13	H13A	110.8		O12	C13	H13B	110.8
C13a	C13	H13A	110.8		C13a	C13	H13B	110.8
H13A	C13	H13B	108.9		C13	C13a	H13	110.8
C14	C13a	H13	110.8		C16a	C13a	H13	110.8
C13a	C14	H14	108.2		C15	C14	H14	108.2
C18	C14	H14	108.2		C11	C16a	H16	110.6
C13a	C16a	H16	110.6		C16	C16a	H16	110.6
C15	C16	H16A	110.9		C15	C16	H16B	110.9
C16a	C16	H16A	110.9		C16a	C16	H16B	110.9
H16A	C16	H16B	108.9		I17	C17	H17A	110.0
I17	C17	H17B	110.0		C11	C17	H17A	110.0
C11	C17	H17B	110.0		H17A	C17	H17B	108.4
O22	C21	H21	109.5		C26a	C21	H21	109.5
C27	C21	H21	109.5		C23	C23a	H23	111.2
C24	C23a	H23	111.2		C26a	C23a	H23	111.2
O22	C23	H23A	110.7		O22	C23	H23B	110.7
C23a	C23	H23A	110.7		C23a	C23	H23B	110.7
H23A	C23	H23B	108.8		C23a	C24	H24	107.1
C25	C24	H24	107.1		C28	C24	H24	107.1
C21	C26a	H26	111.6		C23a	C26a	H26	111.6
C26	C26a	H26	111.6		C25	C26	H26A	111.1
C25	C26	H26B	111.1		C26a	C26	H26A	111.1


Table 8. Bond angles involving hydrogens (o) (continued)

atom	atom	atom	angle		atom	atom	atom	angle
C26a	C26	H26B	111.1		H26A	C26	H26B	109.1
I27	C27	H27A	109.5		I27	C27	H27B	109.5
C21	C27	H27A	109.5		C21	C27	H27B	109.5
H27A	C27	H27B	108.1


Table 9. Torsion Angles(o)
	(Those having bond angles > 160 or < 20 degrees are excluded.)

atom1	atom2	atom3	atom4	   angle		atom1	atom2	atom3	atom4	   angle
C1	O2	C3	C3a	-40.5(7) 		C3	O2	C1	C6a	30.8(8) 
C3	O2	C1	C7	-88.3(6) 		C11	O12	C13	C13a	-38.0(6) 
C13	O12	C11	C16a	29.9(5) 		C13	O12	C11	C17	-92.7(5) 
C21	O22	C23	C23a	-37.7(6) 		C23	O22	C21	C26a	39.8(6) 
C23	O22	C21	C27	-81.6(7) 		O2	C1	C6a	C3a	-8.8(7) 
O2	C1	C6a	C6	105.9(6) 		O2	C1	C7	I7	-60.3(7) 
C6a	C1	C7	I7	-177.6(5) 		C7	C1	C6a	C3a	111.3(6) 
C7	C1	C6a	C6	-134.0(6) 		O2	C3	C3a	C4	-79.2(7) 
O2	C3	C3a	C6a	33.2(7) 		C3	C3a	C4	C5	151.7(6) 
C3	C3a	C4	C8	-82.9(7) 		C3	C3a	C6a	C1	-14.3(7) 
C3	C3a	C6a	C6	-134.3(6) 		C4	C3a	C6a	C1	105.6(5) 
C4	C3a	C6a	C6	-14.3(5) 		C6a	C3a	C4	C5	39.6(5) 
C6a	C3a	C4	C8	165.0(4) 		C3a	C4	C5	C6	-49.2(6) 
C3a	C4	C5	C9	-169.2(5) 		C3a	C4	C5	C10	65.7(6) 
C3a	C4	C8	F1	65.4(9) 		C3a	C4	C8	F2	-55.1(10) 
C3a	C4	C8	F3	-176.4(5) 		C5	C4	C8	F1	-176.2(6) 
C5	C4	C8	F2	63.2(11) 		C5	C4	C8	F3	-58.0(8) 
C8	C4	C5	C6	-175.0(6) 		C8	C4	C5	C9	64.9(8) 
C8	C4	C5	C10	-60.2(8) 		C4	C5	C6	C6a	40.0(6) 
C9	C5	C6	C6a	161.4(5) 		C10	C5	C6	C6a	-76.8(6) 
C5	C6	C6a	C1	-129.8(4) 		C5	C6	C6a	C3a	-16.5(5) 
O12	C11	C16a	C13a	-9.6(5) 		O12	C11	C16a	C16	105.9(5) 
O12	C11	C17	I17	-166.0(4) 		C16a	C11	C17	I17	76.1(6) 
C17	C11	C16a	C13a	110.5(6) 		C17	C11	C16a	C16	-134.0(6) 
O12	C13	C13a	C14	-84.1(6) 		O12	C13	C13a	C16a	29.9(6) 
C13	C13a	C14	C15	148.5(5) 		C13	C13a	C14	C18	-85.7(6) 
C13	C13a	C16a	C11	-12.0(5) 		C13	C13a	C16a	C16	-132.7(4) 
C14	C13a	C16a	C11	109.5(4) 		C14	C13a	C16a	C16	-11.2(6) 
C16a	C13a	C14	C15	35.3(5) 		C16a	C13a	C14	C18	161.1(5) 
C13a	C14	C15	C16	-45.5(5) 		C13a	C14	C15	C19	72.2(5) 
C13a	C14	C15	C20	-163.6(4) 		C13a	C14	C18	F4	-179.5(6) 
C13a	C14	C18	F5	-60.3(8) 		C13a	C14	C18	F6	61.3(8) 
C15	C14	C18	F4	-61.2(8) 		C15	C14	C18	F5	58.0(7) 
C15	C14	C18	F6	179.6(5) 		C18	C14	C15	C16	-169.6(5) 
C18	C14	C15	C19	-51.9(7) 		C18	C14	C15	C20	72.3(6) 
C14	C15	C16	C16a	38.5(5) 		C19	C15	C16	C16a	-81.8(6) 
C20	C15	C16	C16a	160.3(5) 		C11	C16a	C16	C15	-131.6(6) 


Table 9. Torsion angles (o) (continued)

atom1	atom2	atom3	atom4	angle		atom1	atom2	atom3	atom4	angle
C13a	C16a	C16	C15	-17.0(6) 		O22	C21	C26a	C23a	-25.2(5) 
O22	C21	C26a	C26	88.8(5) 		O22	C21	C27	I27	-69.0(7) 
C26a	C21	C27	I27	174.2(4) 		C27	C21	C26a	C23a	95.7(6) 
C27	C21	C26a	C26	-150.3(5) 		C23	C23a	C24	C25	134.5(6) 
C23	C23a	C24	C28	-97.1(7) 		C24	C23a	C23	O22	-94.1(7) 
C23	C23a	C26a	C21	3.2(5) 		C23	C23a	C26a	C26	-114.5(5) 
C26a	C23a	C23	O22	20.0(6) 		C24	C23a	C26a	C21	123.2(5) 
C24	C23a	C26a	C26	5.5(6) 		C26a	C23a	C24	C25	21.7(6) 
C26a	C23a	C24	C28	150.1(4) 		C23a	C24	C25	C26	-39.7(6) 
C23a	C24	C25	C29	76.0(7) 		C23a	C24	C25	C30	-159.9(5) 
C23a	C24	C28	F7	65.3(8) 		C23a	C24	C28	F8	-174.7(5) 
C23a	C24	C28	F9	-54.6(8) 		C25	C24	C28	F7	-172.1(5) 
C25	C24	C28	F8	-52.1(8) 		C25	C24	C28	F9	68.0(8) 
C28	C24	C25	C26	-166.3(5) 		C28	C24	C25	C29	-50.5(7) 
C28	C24	C25	C30	73.5(8) 		C24	C25	C26	C26a	42.6(5) 
C29	C25	C26	C26a	-76.4(5) 		C30	C25	C26	C26a	163.9(5) 
C21	C26a	C26	C25	-143.3(5) 		C23a	C26a	C26	C25	-30.2(6) 


Table 10. Intramolecular contacts less than 3.60 Å

atom	atom	distance		atom	atom	distance
I7	O2	3.331(5)		I17	C16a	3.551(7)	
I27	O22	3.428(6)		F1	C3	3.164(11)	
F1	C3a	3.011(9)		F2	N10	3.252(10)	
F2	C3a	2.971(9)		F2	C5	3.044(9)	
F2	C10	2.851(10)		F3	N9	3.266(13)	
F3	C5	2.964(9)		F3	C9	2.862(11)	
F3	C10	3.551(10)		F4	N20	3.546(12)	
F4	C15	2.996(11)		F4	C19	3.507(12)	
F4	C20	3.005(12)		F5	N19	3.147(12)	
F5	C13a	2.971(8)		F5	C15	3.003(8)	
F5	C19	2.770(11)		F6	C13	3.148(10)	
F6	C13a	2.942(10)		F7	C23a	2.951(10)	
F7	C23	3.308(12)		F8	N30	3.467(12)	
F8	C25	2.931(11)		F8	C29	3.346(10)	
F8	C30	2.947(13)		F9	N29	3.246(9)	
F9	C23a	2.908(11)		F9	C25	3.081(10)	
F9	C29	2.859(10)		O2	C4	3.072(8)	
O2	C6	3.403(9)		O12	C14	3.154(7)	
O12	C16	3.390(9)		O22	C24	3.265(10)	
O22	C26	3.145(8)		N9	C4	3.562(13)	
N9	C6	3.533(12)		N9	C10	3.421(14)	
N10	C3a	3.591(10)		N10	C4	3.501(10)	
N10	C6	3.393(11)		N10	C9	3.437(13)	
N19	C14	3.552(10)		N19	C16	3.455(9)	
N19	C20	3.386(10)		N20	C14	3.583(9)	
N20	C16	3.438(10)		N20	C19	3.398(9)	
N29	C24	3.548(9)		N29	C26	3.433(11)	
N29	C30	3.359(9)		N30	C24	3.545(9)	
N30	C26	3.502(10)		N30	C29	3.395(9)	
C1	C4	3.296(9)		C3	C6	3.594(12)	
C3	C7	3.071(11)		C3	C8	3.353(14)	
C3a	C7	3.412(11)		C3a	C10	2.883(11)	
C6a	C10	3.021(10)		C8	C9	3.167(14)	
C8	C10	3.070(11)		C11	C14	3.370(9)	
C13	C16	3.588(10)		C13	C17	3.122(12)	
C13	C18	3.374(10)		C13a	C17	3.467(10)	
C13a	C19	2.997(10)		C16a	C19	3.095(9)	


Table 10. Intramolecular contacts less than 3.60 Å (continued)

atom	atom	distance		atom	atom	distance
C18	C19	3.043(13)		C18	C20	3.245(12)	
C21	C24	3.507(11)		C23a	C27	3.288(11)	
C23a	C29	3.093(9)		C23	C26	3.429(10)	
C23	C27	3.002(11)		C23	C28	3.479(14)	
C26a	C29	2.998(10)		C28	C29	3.029(11)	
C28	C30	3.238(13)		


Table 11. Intramolecular contacts less than 3.60 Å involving hydrogens

atom	atom	distance		atom	atom	distance
I7	H1	3.176		I17	H11	2.961	
I17	H16	3.215		I27	H21	3.075	
F1	H3	3.209		F1	H3A	2.498	
F1	H4	2.529		F2	H3	2.648	
F2	H4	3.196		F3	H4	2.592	
F4	H14	2.575		F5	H13	2.618	
F5	H14	3.196		F6	H13	3.064	
F6	H13A	2.521		F6	H14	2.556	
F7	H23	2.970		F7	H23A	2.803	
F7	H24	2.473		F8	H24	2.616	
F9	H23	2.507		F9	H24	3.180	
O2	H3	3.174		O2	H4	2.724	
O2	H6	3.111		O2	H6B	3.322	
O2	H7A	3.271		O2	H7B	2.640	
O12	H13	3.159		O12	H14	2.848	
O12	H16	3.098		O12	H16B	3.306	
O12	H17A	2.727		O12	H17B	2.526	
O22	H23	3.151		O22	H24	3.013	
O22	H26	3.162		O22	H26B	2.900	
O22	H27A	3.268		O22	H27B	2.589	
N9	H4	3.573		N9	H6B	3.456	
N10	H3	3.135		N10	H6	3.562	
N10	H6A	3.278		N19	H13	3.412	
N19	H16A	3.309		N20	H14	3.534	
N20	H16B	3.314		N29	H26	3.470	
N29	H26A	3.318		N30	H24	3.448	
N30	H26B	3.442		C1	H3	3.200	
C1	H3A	3.152		C1	H3B	2.657	
C1	H4	3.127		C1	H6A	3.182	
C1	H6B	2.575		C3	H1	3.122	
C3	H4	2.619		C3	H6	2.979	
C3	H7B	2.828		C3a	H1	3.152	
C3a	H6A	3.239		C3a	H6B	3.021	
C3a	H7B	3.242		C4	H3A	2.670	
C4	H3B	3.397		C4	H6	3.199	
C4	H6A	3.253		C4	H6B	2.719	
C5	H3	2.769		C5	H6	3.016	


Table 11. Intramolecular contacts less than 3.60 Å involving hydrogens (continued)

atom	atom	distance		atom	atom	distance
C6	H1	2.561		C6	H3	3.056	
C6	H4	2.552		C6a	H3A	3.253	
C6a	H3B	2.815		C6a	H4	2.698	
C6a	H7A	2.681		C6a	H7B	2.644	
C7	H3B	2.926		C7	H6	2.455	
C8	H3	2.727		C8	H3A	3.050	
C9	H4	2.727		C9	H6A	2.896	
C9	H6B	2.641		C10	H3	2.715	
C10	H4	3.356		C10	H6	3.139	
C10	H6A	2.577		C10	H6B	3.322	
C11	H13	3.187		C11	H13A	3.163	
C11	H13B	2.701		C11	H14	3.269	
C11	H16A	3.163		C11	H16B	2.574	
C13	H11	3.126		C13	H14	2.638	
C13	H16	3.002		C13	H17A	2.982	
C13	H17B	3.425		C13a	H11	3.155	
C13a	H16A	3.245		C13a	H16B	3.021	
C13a	H17A	3.312		C14	H13A	2.677	
C14	H13B	3.410		C14	H16	3.201	
C14	H16A	3.262		C14	H16B	2.757	
C15	H13	2.805		C15	H16	3.003	
C16a	H13A	3.249		C16a	H13B	2.852	
C16a	H14	2.773		C16a	H17A	2.648	
C16a	H17B	3.376		C16	H11	2.542	
C16	H13	3.073		C16	H14	2.618	
C17	H13B	3.016		C17	H16	2.531	
C18	H13	2.666		C18	H13A	3.091	
C19	H13	2.883		C19	H14	3.365	
C19	H16	3.201		C19	H16A	2.566	
C19	H16B	3.323		C20	H14	2.678	
C20	H16A	2.851		C20	H16B	2.573	
C21	H23	3.087		C21	H23A	3.139	
C21	H23B	2.675		C21	H24	3.566	
C21	H26A	3.042		C21	H26B	2.550	
C23a	H21	3.231		C23a	H26A	3.291	
C23a	H26B	2.902		C23a	H27B	3.079	
C23	H21	3.137		C23	H24	2.544	


Table 11. Intramolecular contacts less than 3.60 Å involving hydrogens (continued)

atom	atom	distance		atom	atom	distance
C23	H26	3.141		C23	H26B	3.472	
C23	H27B	2.697		C24	H23A	2.623	
C24	H23B	3.377		C24	H26	3.115	
C24	H26A	3.266		C24	H26B	2.711	
C25	H23	2.980		C25	H26	2.900	
C26a	H23A	3.216		C26a	H23B	2.947	
C26a	H24	2.881		C26a	H27A	2.675	
C26a	H27B	2.760		C26	H21	2.539	
C26	H23	3.184		C26	H24	2.641	
C27	H23	3.566		C27	H23B	2.862	
C27	H26	2.561		C28	H23	2.607	
C28	H23A	3.261		C29	H23	3.168	
C29	H24	3.350		C29	H26	2.963	
C29	H26A	2.584		C29	H26B	3.330	
C30	H24	2.616		C30	H26A	2.878	
C30	H26B	2.658		H1	H4	3.300	
H1	H6	2.730		H1	H6A	3.094	
H1	H6B	2.200		H1	H7A	2.364	
H1	H7B	2.870		H3	H3A	2.580	
H3	H3B	2.301		H3	H4	2.891	
H3	H6	2.286		H3	H7B	3.504	
H3A	H4	2.621		H3B	H4	3.578	
H3B	H6	3.086		H3B	H7B	2.392	
H4	H6A	3.520		H4	H6B	2.512	
H6	H6A	2.276		H6	H6B	2.845	
H6	H7A	2.397		H6	H7B	2.424	
H11	H14	3.448		H11	H16	2.716	
H11	H16A	3.063		H11	H16B	2.187	
H11	H17A	2.868		H11	H17B	2.457	
H13	H13A	2.602		H13	H13B	2.281	
H13	H14	2.891		H13	H16	2.270	
H13	H17A	3.527		H13A	H14	2.572	
H13B	H14	3.590		H13B	H16	3.145	
H13B	H17A	2.602		H13B	H17B	3.270	
H14	H16A	3.578		H14	H16B	2.604	
H16	H16A	2.268		H16	H16B	2.843	
H16	H17A	2.394		H16	H17B	3.486	


Table 11. Intramolecular contacts less than 3.60 Å involving hydrogens (continued)

atom	atom	distance		atom	atom	distance
H21	H26	2.634		H21	H26A	2.851	
H21	H26B	2.316		H21	H27A	2.420	
H21	H27B	2.872		H23	H23A	2.678	
H23	H23B	2.284		H23	H24	2.847	
H23	H26	2.275		H23	H27B	3.140	
H23A	H24	2.313		H23B	H24	3.461	
H23B	H26	3.424		H23B	H27B	2.265	
H24	H26A	3.593		H24	H26B	2.584	
H26	H26A	2.310		H26	H26B	2.887	
H26	H27A	2.365		H26	H27B	2.742	


Table 12. Intermolecular contacts less than 3.60 Å

atom	atom	distance		atom	atom	distance
I7	F81	3.542(7)		I17	N292	3.413(9)	
F1	N193	3.102(11)		F1	C193	3.243(12)	
F2	F5	2.876(8)		F2	C163	3.556(10)	
F3	F7	3.255(9)		F3	F9	3.200(9)	
F3	C23a	3.364(10)		F3	C23	3.578(13)	
F4	N10	3.214(11)		F4	C264	3.368(10)	
F5	F2	2.876(8)		F5	C163	3.468(9)	
F7	F3	3.255(9)		F7	C213	3.424(8)	
F7	C26a3	3.344(9)		F7	C273	3.261(9)	
F8	I75	3.542(7)		F9	F3	3.200(9)	
O2	O126	3.256(8)		O2	C136	3.307(10)	
O12	O27	3.256(8)		O12	C17	3.462(9)	
O12	C47	3.391(8)		O12	C67	3.386(8)	
O22	C6a8	3.401(9)		N9	C176	3.468(10)	
N10	F4	3.214(11)		N10	N20	3.281(11)	
N10	C32	3.588(14)		N10	C20	3.377(11)	
N19	F12	3.102(11)		N19	N29	3.547(9)	
N19	C23a	3.541(9)		N20	N10	3.281(11)	
N20	N309	3.518(10)		N20	C32	3.588(12)	
N20	C249	3.325(10)		N20	C309	3.369(10)	
N29	I173	3.413(9)		N29	N19	3.547(9)	
N29	C13a	3.348(8)		N29	C16a	3.542(8)	
N30	N2010	3.518(10)		N30	C2010	3.428(10)	
C1	O126	3.462(9)		C3	N103	3.588(14)	
C3	N203	3.588(12)		C3	C103	3.585(13)	
C4	O126	3.391(8)		C6	O126	3.386(8)	
C6a	O224	3.401(9)		C10	C32	3.585(13)	
C13	O27	3.307(10)		C13a	N29	3.348(8)	
C16a	N29	3.542(8)		C16	F22	3.556(10)	
C16	F52	3.468(9)		C17	N97	3.468(10)	
C19	F12	3.243(12)		C20	N10	3.377(11)	
C20	N309	3.428(10)		C21	F72	3.424(8)	
C23a	F3	3.364(10)		C23a	N19	3.541(9)	
C23	F3	3.578(13)		C24	N2010	3.325(10)	
C26a	F72	3.344(9)		C26	F48	3.368(10)	
C27	F72	3.261(9)		C30	N2010	3.369(10)	


Symmetry Operators:

(1)  X,Y-1,Z+1		(2)  X+1,Y,Z
(3)  X-1,Y,Z		(4)  X,Y-1,Z
(5)  X,Y+1,Z-1		(6)  X-1,Y,Z+1
(7)  X+1,Y,Z-1		(8)  X,Y+1,Z
(9)  X+1,Y-1,Z		(10)  X-1,Y+1,Z


Table 13. Intermolecular contacts less than 3.60 Å involving hydrogens

atom	atom	distance		atom	atom	distance
I7	H6A1	3.505		I7	H26A2	3.532	
I17	H7A3	3.111		I17	H134	3.356	
I17	H13B4	3.557		I27	H65	3.359	
I27	H6A5	3.506		I27	H7B6	3.497	
I27	H13B7	3.529		I27	H17A7	3.137	
F1	H16A1	3.286		F1	H27A1	3.175	
F2	H16A1	2.805		F3	H17B8	3.509	
F3	H23	2.570		F3	H23B	3.129	
F3	H27A1	3.510		F4	H26A9	2.980	
F4	H26B9	2.853		F5	H16A1	3.021	
F5	H16B1	3.004		F6	H111	2.972	
F6	H16B1	3.075		F7	H211	3.212	
F7	H261	2.769		F7	H26A1	3.226	
F7	H27A1	2.432		F8	H26A1	2.918	
F9	H16A1	3.301		O2	H13A8	2.587	
O12	H110	2.939		O12	H410	2.468	
O12	H6B10	2.581		O22	H35	3.074	
O22	H65	2.527		O22	H7B5	3.239	
N9	H3A4	2.998		N9	H13A7	3.314	
N9	H13B7	3.455		N9	H17B8	2.700	
N9	H23B	3.204		N9	H27B	2.862	
N10	H3A4	3.322		N10	H3B4	2.939	
N10	H219	2.753		N10	H26B9	3.531	
N19	H23	2.740		N19	H26	2.876	
N20	H34	2.865		N20	H3A4	3.575	
N20	H3B4	3.123		N20	H2411	2.375	
N20	H26B11	3.223		N29	H13	2.503	
N29	H16	2.864		N30	H112	2.753	
N30	H6B12	3.265		N30	H1413	2.797	
N30	H16B13	2.885		C1	H13A8	3.363	
C9	H3A4	3.017		C9	H17B8	2.971	
C10	H3A4	3.184		C10	H3B4	3.137	
C10	H219	3.465		C11	H410	3.541	
C11	H6B10	3.190		C13	H110	3.469	
C13	H410	3.268		C18	H16B1	3.530	
C20	H34	3.556		C20	H2411	3.337	
C21	H65	3.390		C23	H7B5	3.467	


Table 13. Intermolecular contacts less than 3.60 Å involving hydrogens (continued)

atom	atom	distance		atom	atom	distance
C29	H13	3.541		H1	O128	2.939	
H1	N3014	2.753		H1	C138	3.469	
H1	H13A8	3.055		H1	H148	2.874	
H3	O229	3.074		H3	N201	2.865	
H3	C201	3.556		H3	H219	3.585	
H3	H26B9	3.551		H3A	N91	2.998	
H3A	N101	3.322		H3A	N201	3.575	
H3A	C91	3.017		H3A	C101	3.184	
H3B	N101	2.939		H3B	N201	3.123	
H3B	C101	3.137		H3B	H6A1	3.531	
H3B	H23A9	3.589		H4	O128	2.468	
H4	C118	3.541		H4	C138	3.268	
H4	H13A8	3.310		H4	H13B8	3.384	
H4	H17B8	3.115		H6	I279	3.359	
H6	O229	2.527		H6	C219	3.390	
H6	H219	3.301		H6A	I74	3.505	
H6A	I279	3.506		H6A	H3B4	3.531	
H6B	O128	2.581		H6B	N3014	3.265	
H6B	C118	3.190		H6B	H118	2.798	
H7A	I172	3.111		H7B	I2715	3.497	
H7B	O229	3.239		H7B	C239	3.467	
H7B	H23A9	3.067		H7B	H23B9	3.567	
H11	F64	2.972		H11	H6B10	2.798	
H13	I171	3.356		H13	N29	2.503	
H13	C29	3.541		H13A	O210	2.587	
H13A	N916	3.314		H13A	C110	3.363	
H13A	H110	3.055		H13A	H410	3.310	
H13B	I171	3.557		H13B	I2716	3.529	
H13B	N916	3.455		H13B	H410	3.384	
H13B	H27B16	3.577		H14	N3011	2.797	
H14	H110	2.874		H16	N29	2.864	
H16A	F14	3.286		H16A	F24	2.805	
H16A	F54	3.021		H16A	F94	3.301	
H16B	F54	3.004		H16B	F64	3.075	
H16B	N3011	2.885		H16B	C184	3.530	
H17A	I2716	3.137		H17B	F310	3.509	
H17B	N910	2.700		H17B	C910	2.971	


Table 13. Intermolecular contacts less than 3.60 Å involving hydrogens (continued)

atom	atom	distance		atom	atom	distance
H17B	H410	3.115		H17B	H23B10	2.872	
H21	F74	3.212		H21	N105	2.753	
H21	C105	3.465		H21	H35	3.585	
H21	H65	3.301		H23	F3	2.570	
H23	N19	2.740		H23A	H3B5	3.589	
H23A	H7B5	3.067		H23A	H27A1	3.413	
H23B	F3	3.129		H23B	N9	3.204	
H23B	H7B5	3.567		H23B	H17B8	2.872	
H24	N2013	2.375		H24	C2013	3.337	
H26	F74	2.769		H26	N19	2.876	
H26A	I73	3.532		H26A	F45	2.980	
H26A	F74	3.226		H26A	F84	2.918	
H26B	F45	2.853		H26B	N105	3.531	
H26B	N2013	3.223		H26B	H35	3.551	
H27A	F14	3.175		H27A	F34	3.510	
H27A	F74	2.432		H27A	H23A4	3.413	
H27B	N9	2.862		H27B	H13B7	3.577	


Symmetry Operators:

(1)  X-1,Y,Z		(2)  X-1,Y-1,Z+1
(3)  X+1,Y+1,Z-1		(4)  X+1,Y,Z
(5)  X,Y+1,Z		(6)  X+1,Y+1,Z
(7)  X,Y,Z+1		(8)  X-1,Y,Z+1
(9)  X,Y-1,Z		(10)  X+1,Y,Z-1
(11)  X+1,Y-1,Z		(12)  X,Y+1,Z-1
(13)  X-1,Y+1,Z		(14)  X,Y-1,Z+1
(15)  X-1,Y-1,Z		(16)  X,Y,Z-1
