# Supplementary material for: Cooperative Palladium/Isothiourea Catalyzed Enantioselective Formal (3+2) Cycloaddition of Vinylcyclopropanes and α,β‐Unsaturated Esters
Source: Angew Chem Int Ed Engl. 2022 Apr 28;61(25):e202202621. doi: 10.1002/anie.202202621 (PMC9324207; doi:10.1002/anie.202202621)

checkCIF/PLATON report

Structure factors have been supplied for datablock(s) 27

THIS REPORT IS FOR GUIDANCE ONLY. IF USED AS PART OF A REVIEW PROCEDURE FOR PUBLICATION, IT SHOULD NOT REPLACE THE EXPERTISE OF AN EXPERIENCED CRYSTALLOGRAPHIC REFEREE.

No syntax errors found. CIF dictionary Interpreting this report

Datablock: 27

```
Bond precision:   C-C = 0.0099 A          Wavelength=0.71075

Cell:           a=7.6910(5)        b=11.5327(8)      c=11.9797(8)
                alpha=79.079(6)    beta=79.774(6)    gamma=73.365(5)
Temperature:    173 K

                 Calculated                   Reported
Volume           991.04(12)                   991.04(12)
Space group      P 1                          P 1
Hall group       P 1                          P 1
Moiety formula   C11 H10 F3 I N2 O            C11 H10 F3 I N2 O
Sum formula      C11 H10 F3 I N2 O            C11 H10 F3 I N2 O
Mr               370.11                       370.11
Dx,g cm-3        1.860                        1.860
Z                3                            3
Mu (mm-1)        2.449                        2.449
F000             534.0                        534.0
F000'            532.87
h,k,lmax         9,14,15                      9,14,15
Nref             9048[ 4524]                  8424
Tmin,Tmax        0.578,0.659                  0.531,0.659
Tmin'            0.431

Correction method= # Reported T Limits: Tmin=0.531 Tmax=0.659
AbsCorr = MULTI-SCAN

Data completeness= 1.86/0.93       Theta(max)= 27.491

R(reflections)= 0.0340( 7412)                wR2(reflections)=
                                             0.0886( 8424)
S = 1.046                  Npar= 487
```

The following ALERTS were generated. Each ALERT has the format

test-name_ALERT_alert-type_alert-level.

Click on the hyperlinks for more details of the test.

Alert level C

```
PLAT342_ALERT_3_C Low Bond Precision on  C-C Bonds ...............    0.00994 Ang.
PLAT911_ALERT_3_C Missing FCF Refl Between Thmin & STh/L=    0.600         88 Report
PLAT913_ALERT_3_C Missing # of Very Strong Reflections in FCF ....         32 Note
PLAT934_ALERT_3_C Number of (Iobs-Icalc)/Sigma(W) > 10 Outliers ..          1 Check
```

Alert level G

```
PLAT019_ALERT_1_G _diffrn_measured_fraction_theta_full/*_max < 1.0      0.997 Report
PLAT242_ALERT_2_G Low    'MainMol' Ueq as Compared to Neighbors of         C8 Check
PLAT242_ALERT_2_G Low    'MainMol' Ueq as Compared to Neighbors of        C18 Check
PLAT242_ALERT_2_G Low    'MainMol' Ueq as Compared to Neighbors of        C28 Check
PLAT398_ALERT_2_G Deviating  C-O-C   Angle From 120 for O2       .      106.8 Degree
PLAT398_ALERT_2_G Deviating  C-O-C   Angle From 120 for O12      .      108.3 Degree
PLAT398_ALERT_2_G Deviating  C-O-C   Angle From 120 for O22      .      106.9 Degree
PLAT431_ALERT_2_G Short Inter HL..A Contact  I17      ..N29      .       3.41 Ang.
                                                   1+x,y,z  =      1_655 Check
PLAT791_ALERT_4_G Model has Chirality at C1         (Sohnke SpGr)          R Verify
PLAT791_ALERT_4_G Model has Chirality at C3A        (Sohnke SpGr)          R Verify
PLAT791_ALERT_4_G Model has Chirality at C4         (Sohnke SpGr)          S Verify
PLAT791_ALERT_4_G Model has Chirality at C6A        (Sohnke SpGr)          R Verify
PLAT791_ALERT_4_G Model has Chirality at C11        (Sohnke SpGr)          R Verify
PLAT791_ALERT_4_G Model has Chirality at C13A       (Sohnke SpGr)          R Verify
PLAT791_ALERT_4_G Model has Chirality at C14        (Sohnke SpGr)          S Verify
PLAT791_ALERT_4_G Model has Chirality at C16A       (Sohnke SpGr)          R Verify
PLAT791_ALERT_4_G Model has Chirality at C21        (Sohnke SpGr)          R Verify
PLAT791_ALERT_4_G Model has Chirality at C23A       (Sohnke SpGr)          R Verify
PLAT791_ALERT_4_G Model has Chirality at C24        (Sohnke SpGr)          S Verify
PLAT791_ALERT_4_G Model has Chirality at C26A       (Sohnke SpGr)          R Verify
PLAT910_ALERT_3_G Missing # of FCF Reflection(s) Below Theta(Min).          2 Note
PLAT912_ALERT_4_G Missing # of FCF Reflections Above STh/L=  0.600         11 Note
PLAT941_ALERT_3_G Average HKL Measurement Multiplicity ..........         2.2 Low
PLAT978_ALERT_2_G Number C-C Bonds with Positive Residual Density.          0 Info
```

0 **ALERT level A** = Most likely a serious problem - resolve or explain
0 **ALERT level B** = A potentially serious problem, consider carefully
4 **ALERT level C** = Check. Ensure it is not caused by an omission or oversight
24 **ALERT level G** = General information/check it is not something unexpected

1 ALERT type 1 CIF construction/syntax error, inconsistent or missing data
8 ALERT type 2 Indicator that the structure model may be wrong or deficient
6 ALERT type 3 Indicator that the structure quality may be low
13 ALERT type 4 Improvement, methodology, query or suggestion
0 ALERT type 5 Informative message, check

It is advisable to attempt to resolve as many as possible of the alerts in all categories. Often the minor alerts point to easily fixed oversights, errors and omissions in your CIF or refinement strategy, so attention to these fine details can be worthwhile. In order to resolve some of the more serious problems it may be necessary to carry out additional measurements or structure refinements. However, the purpose of your study may justify the reported deviations and the more serious of these should normally be commented upon in the discussion or experimental section of a paper or in the "special_details" fields of the CIF. checkCIF was carefully designed to identify outliers and unusual parameters, but every test has its limitations and alerts that are not important in a particular case may appear. Conversely, the absence of alerts does not guarantee there are no aspects of the results needing attention. It is up to the individual to critically assess their own results and, if necessary, seek expert advice.

Publication of your CIF in IUCr journals

A basic structural check has been run on your CIF. These basic checks will be run on all CIFs submitted for publication in IUCr journals (*Acta Crystallographica*, *Journal of Applied Crystallography*, *Journal of Synchrotron Radiation*); however, if you intend to submit to *Acta Crystallographica Section C* or *E* or *IUCrData*, you should make sure that full publication checks are run on the final version of your CIF prior to submission.

Publication of your CIF in other journals

Please refer to the *Notes for Authors* of the relevant journal for any special instructions relating to CIF submission.

PLATON version of 20/01/2022; check.def file version of 19/01/2022

Datablock 27 - ellipsoid plot

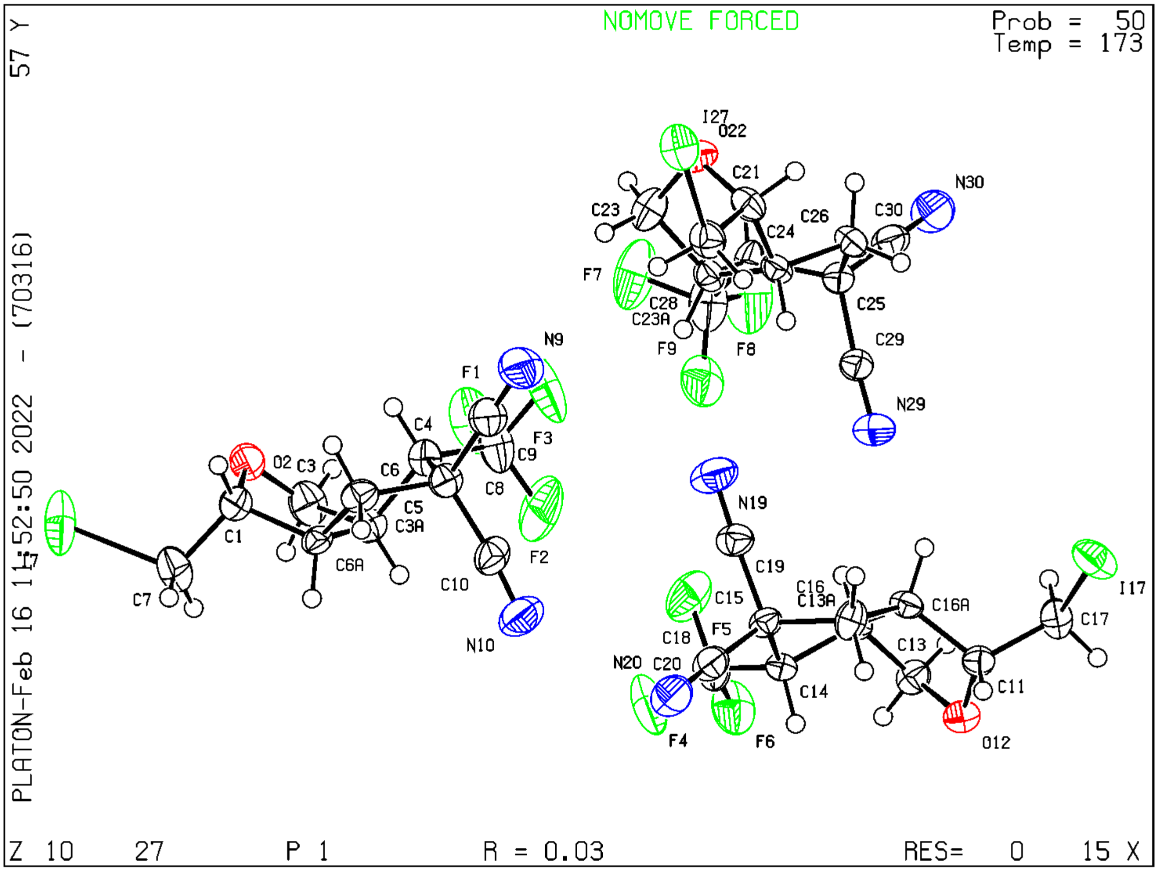

Supplement: Supplementary file 6 — Supporting Information [file ANIE-61-0-s003.pdf]
