# Supplementary material for: Cooperative Palladium/Isothiourea Catalyzed Enantioselective Formal (3+2) Cycloaddition of Vinylcyclopropanes and α,β‐Unsaturated Esters
Source: Angew Chem Int Ed Engl. 2022 Apr 28;61(25):e202202621. doi: 10.1002/anie.202202621 (PMC9324207; doi:10.1002/anie.202202621)
Supplement: Supplementary file 8 — Supporting Information [file ANIE-61-0-s010.rtf]

	A colorless platelet crystal of C12H10F3N3O having approximate dimensions of 0.100 x 0.100 x 0.010 mm was mounted in a loop. All measurements were made on a Rigaku XtaLAB P200 diffractometer using multi-layer mirror monochromated Mo-Ka radiation.


	Cell constants and an orientation matrix for data collection corresponded to a primitive tetragonal cell (laue class: 4/mmm) with dimensions:

           a  =    7.5386(8) Å
           c  =   42.598(5) Å
           V  =  2420.9(5) Å3

For Z = 8 and F.W. = 269.23, the calculated density is 1.477 g/cm3. Based on the reflection conditions of:

           h00:  h = 2n
           00l:  l = 4n


and the successful solution and refinement of the structure, the space group was determined to be:

P43212 (#96) 


	The data were collected at a temperature of -100 + 1oC to a maximum 2q value of 50.7o. 

Data Reduction

	Of the 18286 reflections were collected, where 2212 were unique (Rint = 0.0418); equivalent reflections were merged. Data were collected and processed using CrystalClear (Rigaku). 1 

	The linear absorption coefficient, m, for Mo-Ka radiation is 1.288 cm-1. An empirical absorption correction was applied which resulted in transmission factors ranging from 0.751 to 0.999. The data were corrected for Lorentz and polarization effects. 


Structure Solution and Refinement

	The structure was solved by direct methods2 and expanded using Fourier techniques. The non-hydrogen atoms were refined anisotropically. Some hydrogen atoms were refined isotropically and the rest were refined using the riding model.The final cycle of full-matrix least-squares refinement3 on F2 was based on 2212 observed reflections and 176 variable parameters and converged (largest parameter shift was 0.00 times its esd) with unweighted and weighted agreement factors of: 

R1 = S ||Fo| - |Fc|| / S |Fo| = 0.0509 


wR2 = [ S ( w (Fo2 - Fc2)2 )/ S w(Fo2)2]1/2 = 0.1324 


	The goodness of fit4 was 1.14. Unit weights were used.  The maximum and minimum peaks on the final difference Fourier map corresponded to 0.23 and -0.16 e/Å3, respectively. The final Flack parameter 5 was 0.4(3),  indicating that inversion-distinguishing power is too weak.6   

	Neutral atom scattering factors were taken from International Tables for Crystallography (IT), Vol. C, Table 6.1.1.4 7.  Anomalous dispersion effects were included in Fcalc8; the values for Df' and Df" were those of Creagh and McAuley9. The values for the mass attenuation coefficients are those of Creagh and Hubbell10. All calculations were performed using the CrystalStructure11 crystallographic software package except for refinement, which was performed using SHELXL Version 2018/312.


References 


(1) CrystalClear: Data Collection and Processing Software, Rigaku Corporation (1998-2015). Tokyo 196-8666, Japan.

(2) SHELXT Version 2018/2: Sheldrick, G. M. (2014). Acta Cryst. A70, C1437.

(3) Least Squares function minimized: (SHELXL Version 2018/3)

		Sw(Fo2-Fc2)2     where w = Least Squares weights.

(4) Goodness of fit is defined as:

 	 	 [Sw(Fo2-Fc2)2/(No-Nv)]1/2

	 	 where:	 No  = number of observations
	 	 	 	 Nv  = number of variables 


(5) Parsons, S., Flack, H.D. and Wagner, T. Acta Cryst. B69 (2013) 249-259.

(6) Flack, H.D. and Bernardinelli (2000), J. Appl. Cryst. 33, 114-1148.

(7) International Tables for Crystallography, Vol.C (1992). Ed. A.J.C. Wilson, Kluwer Academic Publishers, Dordrecht, Netherlands, Table 6.1.1.4, pp. 572. 

(8) Ibers, J. A. & Hamilton, W. C.; Acta Crystallogr., 17, 781 (1964). 

(9) Creagh, D. C. & McAuley, W.J .; "International Tables for Crystallography", Vol C, (A.J.C. Wilson, ed.), Kluwer Academic Publishers, Boston, Table 4.2.6.8, pages 219-222 (1992). 

(10) Creagh, D. C. & Hubbell, J.H..; "International Tables for Crystallography", Vol C, (A.J.C. Wilson, ed.), Kluwer Academic Publishers, Boston, Table 4.2.4.3, pages 200-206 (1992). 

(11) CrystalStructure 4.3: Crystal Structure Analysis Package, Rigaku Corporation (2000-2019). Tokyo 196-8666, Japan.

(12) SHELXL Version 2018/3: Sheldrick, G. M. (2008). Acta Cryst. A64, 112-122.


EXPERIMENTAL DETAILS 


A. Crystal Data 


Empirical Formula	C12H10F3N3O

Formula Weight	269.23

Crystal Color, Habit	colorless, platelet

Crystal Dimensions	0.100 X 0.100 X 0.010 mm

Crystal System	tetragonal

Lattice Type	Primitive

Lattice Parameters	a =   7.5386(8) Å
	c =  42.598(5) Å
	V = 2420.9(5) Å3

Space Group	P43212 (#96)

Z value	8

Dcalc	1.477 g/cm3

F000	1104.00

m(MoKa)	1.288 cm-1

B. Intensity Measurements 


Diffractometer	XtaLAB P200

Radiation	MoKa (l = 0.71075 Å)
	multi-layer mirror monochromated

Voltage, Current	45kV, 66mA

Temperature	-100.0oC

Detector Aperture	83.8 x 70.0 mm

Data Images	688 exposures

Pixel Size	0.172 mm

2qmax	50.7o

No. of Reflections Measured	Total: 18286
	Unique: 2212 (Rint = 0.0418)
	Parsons quotients (Flack x parameter): 723

Corrections	Lorentz-polarization
		Absorption
		(trans. factors: 0.751 - 0.999)

C. Structure Solution and Refinement 


Structure Solution	Direct Methods (SHELXT Version 2018/2)

Refinement	Full-matrix least-squares on F2

Function Minimized	S w (Fo2 - Fc2)2 

Least Squares Weights	w = 1/ [ s2(Fo2) + (0.0485 . P)2 
	 + 2.1962 .  P ]
	 where P = (Max(Fo2,0) + 2Fc2)/3

2qmax cutoff	50.7o

Anomalous Dispersion	All non-hydrogen atoms

No. Observations (All reflections)	2212

No. Variables	176

Reflection/Parameter Ratio	12.57

Residuals: R1 (I>2.00s(I))	0.0509

Residuals: R (All reflections)	0.0532

Residuals: wR2 (All reflections)	0.1324

Goodness of Fit Indicator	1.140

Flack parameter (Parsons' quotients = 723)	0.4(3)

Max Shift/Error in Final Cycle	0.000

Maximum peak in Final Diff. Map	0.23 e/Å3

Minimum peak in Final Diff. Map	-0.16 e/Å3


Table 1. Atomic coordinates and Biso/Beq

atom	   x	   y	   z	 Beq
F1    	 0.4551(4)	 1.0434(4)	 0.62423(6)	 4.63(6)
F2    	 0.3643(4)	 1.2748(3)	 0.59964(6)	 4.61(6)
F3    	 0.6395(4)	 1.1984(4)	 0.59756(6)	 4.71(6)
O1    	 0.6795(4)	 0.9960(4)	 0.52018(6)	 3.05(5)
N2    	 0.8172(5)	 0.7540(5)	 0.53927(8)	 3.30(6)
N9    	 0.0685(6)	 0.8814(6)	 0.61368(10)	 4.72(8)
N10   	 0.0692(6)	 1.2250(6)	 0.53416(11)	 5.04(9)
C1    	 0.6881(5)	 0.8761(5)	 0.53980(8)	 2.61(6)
C3    	 0.7985(6)	 0.5835(6)	 0.55535(10)	 3.64(8)
C4    	 0.6429(6)	 0.4818(6)	 0.54266(10)	 3.78(9)
C5a   	 0.4168(5)	 0.7079(5)	 0.55980(9)	 2.74(7)
C5    	 0.4792(6)	 0.5408(5)	 0.54363(10)	 3.38(8)
C6    	 0.2683(5)	 0.8037(6)	 0.54276(9)	 3.05(7)
C7    	 0.2419(5)	 0.9728(5)	 0.56288(9)	 3.05(7)
C8    	 0.4407(5)	 1.0290(5)	 0.56866(9)	 2.74(7)
C8a   	 0.5532(5)	 0.8585(5)	 0.56631(8)	 2.62(6)
C9    	 0.1443(6)	 0.9239(6)	 0.59185(10)	 3.53(8)
C10   	 0.1429(6)	 1.1140(6)	 0.54684(10)	 3.76(8)
C11   	 0.4734(6)	 1.1350(6)	 0.59777(10)	 3.65(8)

Beq = 8/3 p2(U11(aa*)2 + U22(bb*)2 + U33(cc*)2 + 2U12(aa*bb*)cos g + 2U13(aa*cc*)cos b + 2U23(bb*cc*)cos a)


Table 2. Atomic coordinates and Biso involving hydrogen atoms

atom	   x	   y	   z	 Biso
H2    	 0.876(5)	 0.753(5)	 0.5188(4)	 2.6(8)
H3A   	 0.78182	 0.60406	 0.57812	 4.367
H3B   	 0.90809	 0.51304	 0.55250	 4.367
H4    	 0.66409	 0.36898	 0.53346	 4.531
H5    	 0.36820	 0.67095	 0.58064	 3.293
H5A   	 0.39167	 0.47194	 0.53319	 4.054
H6A   	 0.15903	 0.73098	 0.54221	 3.663
H6B   	 0.30328	 0.83408	 0.52101	 3.663
H8    	 0.47579	 1.10576	 0.55056	 3.286
H8A   	 0.61564	 0.83620	 0.58665	 3.149


Table 3. Anisotropic displacement parameters

atom	  U11	  U22	  U33	  U12	  U13	  U23
F1    	0.078(2)	0.0587(17)	0.0390(12)	-0.0060(14)	 0.0029(13)	 0.0005(12)
F2    	0.0669(18)	0.0441(15)	0.0642(15)	 0.0074(14)	 0.0125(14)	-0.0101(12)
F3    	0.0546(17)	0.0607(17)	0.0635(16)	-0.0181(14)	-0.0078(13)	-0.0051(13)
O1    	0.0367(15)	0.0377(15)	0.0415(13)	-0.0015(12)	 0.0051(12)	 0.0029(12)
N2    	0.0280(18)	0.055(2)	0.0426(17)	 0.0073(16)	 0.0012(14)	 0.0067(16)
N9    	0.053(2)	0.060(3)	0.066(2)	 0.002(2)	 0.021(2)	 0.006(2)
N10   	0.059(3)	0.055(2)	0.078(3)	 0.022(2)	-0.006(2)	 0.005(2)
C1    	0.0234(18)	0.038(2)	0.0374(18)	-0.0039(16)	-0.0029(15)	 0.0006(16)
C3    	0.038(2)	0.046(2)	0.054(2)	 0.0205(19)	 0.0030(19)	 0.0103(19)
C4    	0.055(3)	0.033(2)	0.055(2)	 0.010(2)	 0.013(2)	 0.0098(18)
C5a   	0.0287(19)	0.0315(19)	0.0440(19)	-0.0019(15)	 0.0028(16)	 0.0051(16)
C5    	0.046(3)	0.029(2)	0.053(2)	-0.0047(17)	 0.0044(19)	 0.0032(18)
C6    	0.029(2)	0.039(2)	0.048(2)	 0.0009(16)	 -0.0013(16)	 0.0027(18)
C7    	0.031(2)	0.040(2)	0.044(2)	 0.0037(16)	 0.0024(16)	 0.0034(17)
C8    	0.030(2)	0.034(2)	0.0400(19)	 0.0010(15)	 0.0030(16)	 0.0033(16)
C8a   	0.0278(18)	0.037(2)	0.0349(18)	 0.0000(16)	-0.0017(15)	 0.0039(16)
C9    	0.036(2)	0.042(2)	0.056(2)	 0.0096(19)	 0.007(2)	 0.0040(19)
C10   	0.038(2)	0.050(3)	0.055(2)	 0.005(2)	 0.0029(19)	 -0.000(2)
C11   	0.046(3)	0.050(3)	0.043(2)	 -0.001(2)	 0.0047(18)	 0.0006(19)


The general temperature factor expression: exp(-2p2(a*2U11h2 + b*2U22k2 + c*2U33l2 + 2a*b*U12hk + 2a*c*U13hl + 2b*c*U23kl))


Table 4. Bond lengths (Å)

atom	atom	distance		atom	atom	distance
F1	C11	1.329(5)		F2	C11	1.339(5)	
F3	C11	1.341(5)		O1	C1	1.233(5)	
N2	C1	1.340(5)		N2	C3	1.463(6)	
N9	C9	1.138(6)		N10	C10	1.141(6)	
C1	C8a	1.525(5)		C3	C4	1.502(6)	
C4	C5	1.313(7)		C5a	C5	1.511(6)	
C5a	C6	1.517(5)		C5a	C8a	1.557(5)	
C6	C7	1.549(6)		C7	C8	1.577(5)	
C7	C9	1.483(6)		C7	C10	1.469(6)	
C8	C8a	1.543(5)		C8	C11	1.496(6)	


Table 5. Bond lengths involving hydrogens (Å)

atom	atom	distance		atom	atom	distance
N2	H2	0.98(2)		C3	H3A	0.990	
C3	H3B	0.990		C4	H4	0.950	
C5a	H5	1.000		C5	H5A	0.950	
C6	H6A	0.990		C6	H6B	0.990	
C8	H8	1.000		C8a	H8A	1.000	


Table 6. Bond angles (o)

atom	atom	atom	angle		atom	atom	atom	angle
C1	N2	C3	121.7(3)		O1	C1	N2	122.1(3)
O1	C1	C8a	122.0(3)		N2	C1	C8a	115.9(3)
N2	C3	C4	110.8(3)		C3	C4	C5	123.4(4)
C5	C5a	C6	114.1(3)		C5	C5a	C8a	118.9(3)
C6	C5a	C8a	103.0(3)		C4	C5	C5a	126.1(4)
C5a	C6	C7	102.8(3)		C6	C7	C8	100.7(3)
C6	C7	C9	108.6(3)		C6	C7	C10	113.9(3)
C8	C7	C9	114.1(3)		C8	C7	C10	111.1(3)
C9	C7	C10	108.4(3)		C7	C8	C8a	106.8(3)
C7	C8	C11	115.4(3)		C8a	C8	C11	114.1(3)
C1	C8a	C5a	111.8(3)		C1	C8a	C8	110.0(3)
C5a	C8a	C8	104.8(3)		N9	C9	C7	177.9(5)
N10	C10	C7	178.6(5)		F1	C11	F2	107.2(3)
F1	C11	F3	106.7(3)		F1	C11	C8	114.1(4)
F2	C11	F3	107.1(4)		F2	C11	C8	111.6(3)
F3	C11	C8	109.8(3)


Table 7. Bond angles involving hydrogens (o)

atom	atom	atom	angle		atom	atom	atom	angle
C1	N2	H2	111(2)		C3	N2	H2	117(2)
N2	C3	H3A	109.5		N2	C3	H3B	109.5
C4	C3	H3A	109.5		C4	C3	H3B	109.5
H3A	C3	H3B	108.0		C3	C4	H4	118.3
C5	C4	H4	118.3		C5	C5a	H5	106.7
C6	C5a	H5	106.7		C8a	C5a	H5	106.7
C4	C5	H5A	117.0		C5a	C5	H5A	116.9
C5a	C6	H6A	111.2		C5a	C6	H6B	111.2
C7	C6	H6A	111.2		C7	C6	H6B	111.2
H6A	C6	H6B	109.1		C7	C8	H8	106.7
C8a	C8	H8	106.7		C11	C8	H8	106.6
C1	C8a	H8A	110.0		C5a	C8a	H8A	110.0
C8	C8a	H8A	110.0


Table 8. Torsion Angles(o)
	(Those having bond angles > 160 or < 20 degrees are excluded.)

atom1	atom2	atom3	atom4	   angle		atom1	atom2	atom3	atom4	   angle
C1	N2	C3	C4	60.1(5) 		C3	N2	C1	O1	-160.0(3) 
C3	N2	C1	C8a	20.4(5) 		O1	C1	C8a	C5a	103.4(4) 
O1	C1	C8a	C8	-12.7(5) 		N2	C1	C8a	C5a	-77.0(4) 
N2	C1	C8a	C8	166.9(3) 		N2	C3	C4	C5	-60.2(5) 
C3	C4	C5	C5a	-5.9(7) 		C5	C5a	C6	C7	-176.0(3) 
C6	C5a	C5	C4	144.4(4) 		C5	C5a	C8a	C1	36.6(4) 
C5	C5a	C8a	C8	155.7(3) 		C8a	C5a	C5	C4	22.4(6) 
C6	C5a	C8a	C1	-90.8(3) 		C6	C5a	C8a	C8	28.4(3) 
C8a	C5a	C6	C7	-45.7(3) 		C5a	C6	C7	C8	44.0(3) 
C5a	C6	C7	C9	-76.1(3) 		C5a	C6	C7	C10	163.0(3) 
C6	C7	C8	C8a	-26.0(3) 		C6	C7	C8	C11	-154.0(3) 
C9	C7	C8	C8a	90.1(4) 		C9	C7	C8	C11	-37.9(4) 
C10	C7	C8	C8a	-147.0(3) 		C10	C7	C8	C11	85.0(4) 
C7	C8	C8a	C1	119.5(3) 		C7	C8	C8a	C5a	-0.9(3) 
C7	C8	C11	F1	70.1(4) 		C7	C8	C11	F2	-51.6(5) 
C7	C8	C11	F3	-170.2(3) 		C8a	C8	C11	F1	-54.2(5) 
C8a	C8	C11	F2	-175.9(3) 		C8a	C8	C11	F3	65.5(4) 
C11	C8	C8a	C1	-111.7(3) 		C11	C8	C8a	C5a	127.9(3) 


Table 9. Possible hydrogen bonds

Donor	 H	Acceptor	 D...A	D-H	H...A	  D-H...A
 N2	 H2	  O11		2.924(4)	0.98(2)	1.97(2)	  164(3)  


Symmetry Operators:

(1)  Y,X,-Z+1


Table 10. Intramolecular contacts less than 3.60 Å

atom	atom	distance		atom	atom	distance
F1	N9	3.192(5)		F1	C7	3.114(5)	
F1	C8a	2.929(4)		F1	C9	2.865(5)	
F2	N10	3.588(5)		F2	C7	2.913(5)	
F2	C9	3.140(5)		F2	C10	3.052(5)	
F3	C1	3.477(5)		F3	C8a	2.960(5)	
O1	C3	3.567(5)		O1	C5a	3.389(5)	
O1	C6	3.554(5)		O1	C8	2.751(5)	
N2	C5a	3.161(5)		N2	C5	3.018(6)	
N9	C6	3.426(6)		N9	C8	3.576(6)	
N9	C10	3.391(6)		N10	C6	3.532(6)	
N10	C8	3.491(6)		N10	C9	3.393(6)	
C1	C4	2.995(6)		C1	C5	2.983(6)	
C1	C6	3.214(5)		C1	C7	3.579(5)	
C1	C11	3.539(6)		C3	C5a	3.032(6)	
C3	C8a	2.817(6)		C4	C8a	3.088(6)	
C5a	C9	2.956(6)		C8a	C9	3.306(6)	
C9	C11	2.958(6)		C10	C11	3.307(6)	


Table 11. Intramolecular contacts less than 3.60 Å involving hydrogens

atom	atom	distance		atom	atom	distance
F1	H5	3.429		F1	H8	3.177	
F1	H8A	2.543		F2	H8	2.589	
F3	H8	2.454		F3	H8A	2.776	
O1	H2	2.36(4)		O1	H6B	3.088	
O1	H8	2.172		O1	H8A	3.115	
N2	H4	3.133		N2	H8A	2.601	
N9	H5	3.099		N9	H6A	3.320	
N10	H6B	3.480		N10	H8	3.270	
C1	H3A	2.715		C1	H3B	3.246	
C1	H5	3.352		C1	H6B	3.026	
C1	H8	2.402		C3	H5	3.481	
C3	H5A	3.317		C3	H8A	2.703	
C4	H2	2.88(4)		C4	H5	2.990	
C4	H8A	3.270		C5a	H3A	2.965	
C5a	H4	3.356		C5a	H8	3.058	
C5	H2	3.55(3)		C5	H3A	2.755	
C5	H3B	3.262		C5	H6A	2.808	
C5	H6B	2.752		C5	H8A	3.062	
C6	H5A	2.699		C6	H8	2.782	
C6	H8A	3.227		C7	H5	2.580	
C7	H8A	3.166		C8	H5	2.801	
C8	H6A	3.290		C8	H6B	2.711	
C8a	H2	3.26(3)		C8a	H3A	2.627	
C8a	H5A	3.459		C8a	H6A	3.288	
C8a	H6B	2.703		C9	H5	2.591	
C9	H6A	2.569		C9	H6B	3.317	
C9	H8	3.349		C10	H6A	2.897	
C10	H6B	2.669		C10	H8	2.515	
C11	H8A	2.539		H2	H3A	2.853	
H2	H3B	2.320		H2	H4	3.363	
H2	H8A	3.549		H3A	H4	2.747	
H3A	H5	3.160		H3A	H8A	2.183	
H3B	H4	2.285		H3B	H8A	3.593	
H4	H5A	2.195		H5	H5A	2.523	
H5	H6A	2.318		H5	H6B	2.864	
H5	H8A	2.258		H5A	H6A	2.653	
H5A	H6B	2.858		H6B	H8	2.733	


Table 11. Intramolecular contacts less than 3.60 Å involving hydrogens (continued)

atom	atom	distance		atom	atom	distance
H8	H8A	2.758		


Table 12. Intermolecular contacts less than 3.60 Å

atom	atom	distance		atom	atom	distance
F1	F21	3.554(4)		F1	F32	3.300(4)	
F1	N93	3.354(5)		F1	C34	3.363(5)	
F2	F12	3.554(4)		F2	F32	2.823(4)	
F2	N91	3.396(5)		F2	C45	3.569(5)	
F2	C55	3.235(5)		F3	F11	3.300(4)	
F3	F21	2.823(4)		F3	C45	3.168(5)	
O1	N26	2.924(4)		O1	N107	3.459(5)	
O1	C68	3.504(5)		N2	O16	2.924(4)	
N2	N26	3.413(5)		N2	C16	3.434(5)	
N2	C67	3.425(5)		N2	C97	3.569(6)	
N9	F14	3.354(5)		N9	F22	3.396(5)	
N9	C5a4	3.519(6)		N9	C8a4	3.490(6)	
N10	O19	3.459(5)		N10	N1010	3.351(7)	
N10	C311	3.505(6)		N10	C1010	3.522(6)	
C1	N26	3.434(5)		C3	F13	3.363(5)	
C3	N1012	3.505(6)		C4	F213	3.569(5)	
C4	F313	3.168(5)		C5a	N93	3.519(6)	
C5	F213	3.235(5)		C6	O114	3.504(5)	
C6	N29	3.425(5)		C8a	N93	3.490(6)	
C9	N29	3.569(6)		C10	N1010	3.522(6)	


Symmetry Operators:

(1)  X+1/2,-Y+1/2+2,-Z+1/4+1		(2)  X+1/2-1,-Y+1/2+2,-Z+1/4+1
(3)  X+1/2,-Y+1/2+1,-Z+1/4+1		(4)  X+1/2-1,-Y+1/2+1,-Z+1/4+1
(5)  X,Y+1,Z		(6)  Y,X,-Z+1
(7)  X+1,Y,Z		(8)  Y,X+1,-Z+1
(9)  X-1,Y,Z		(10)  Y-1,X+1,-Z+1
(11)  X-1,Y+1,Z		(12)  X+1,Y-1,Z
(13)  X,Y-1,Z		(14)  Y-1,X,-Z+1


Table 13. Intermolecular contacts less than 3.60 Å involving hydrogens

atom	atom	distance		atom	atom	distance
F1	H3A1	2.658		F1	H3B1	3.170	
F2	H52	3.094		F2	H5A2	3.204	
F3	H3A2	3.345		F3	H42	3.024	
O1	H23	1.97(2)		O1	H3B3	3.406	
O1	H42	2.870		O1	H6A4	2.954	
O1	H6B4	3.131		N2	H23	2.68(2)	
N2	H6A5	2.586		N9	H3A6	3.367	
N9	H3A1	2.957		N9	H51	2.839	
N9	H8A1	2.701		N10	H3B7	2.608	
N10	H47	3.241		N10	H5A2	3.062	
N10	H6B8	3.002		C1	H23	2.54(2)	
C3	H6A5	2.990		C4	H5A3	3.544	
C4	H6B3	3.353		C4	H89	3.120	
C5	H43	3.513		C5	H5A3	3.460	
C5	H89	3.293		C6	H26	3.15(3)	
C6	H3B6	3.514		C6	H43	3.496	
C8	H42	3.414		C9	H3A1	3.570	
C10	H26	3.59(4)		C10	H3B7	3.499	
C10	H5A2	3.337		C11	H42	3.561	
H2	O13	1.97(2)		H2	N23	2.68(2)	
H2	C13	2.54(2)		H2	C65	3.15(3)	
H2	C105	3.59(4)		H2	H23	2.07(4)	
H2	H6A5	2.361		H2	H6B5	3.280	
H3A	F110	2.658		H3A	F39	3.345	
H3A	N95	3.367		H3A	N910	2.957	
H3A	C910	3.570		H3A	H6A5	3.368	
H3B	F110	3.170		H3B	O13	3.406	
H3B	N1011	2.608		H3B	C65	3.514	
H3B	C1011	3.499		H3B	H6A5	2.544	
H3B	H6B3	3.552		H4	F39	3.024	
H4	O19	2.870		H4	N1011	3.241	
H4	C53	3.513		H4	C63	3.496	
H4	C89	3.414		H4	C119	3.561	
H4	H5A3	3.192		H4	H6B3	2.697	
H4	H89	2.546		H5	F29	3.094	
H5	N910	2.839		H5A	F29	3.204	
H5A	N109	3.062		H5A	C43	3.544	


Table 13. Intermolecular contacts less than 3.60 Å involving hydrogens (continued)

atom	atom	distance		atom	atom	distance
H5A	C53	3.460		H5A	C109	3.337	
H5A	H43	3.192		H5A	H5A3	2.954	
H5A	H89	2.927		H6A	O112	2.954	
H6A	N26	2.586		H6A	C36	2.990	
H6A	H26	2.361		H6A	H3A6	3.368	
H6A	H3B6	2.544		H6B	O112	3.131	
H6B	N108	3.002		H6B	C43	3.353	
H6B	H26	3.280		H6B	H3B3	3.552	
H6B	H43	2.697		H8	C42	3.120	
H8	C52	3.293		H8	H42	2.546	
H8	H5A2	2.927		H8A	N910	2.701	


Symmetry Operators:

(1)  X+1/2-1,-Y+1/2+1,-Z+1/4+1		(2)  X,Y+1,Z
(3)  Y,X,-Z+1		(4)  Y,X+1,-Z+1
(5)  X+1,Y,Z		(6)  X-1,Y,Z
(7)  X-1,Y+1,Z		(8)  Y-1,X+1,-Z+1
(9)  X,Y-1,Z		(10)  X+1/2,-Y+1/2+1,-Z+1/4+1
(11)  X+1,Y-1,Z		(12)  Y-1,X,-Z+1
