# Supplementary material for: Cooperative Palladium/Isothiourea Catalyzed Enantioselective Formal (3+2) Cycloaddition of Vinylcyclopropanes and α,β‐Unsaturated Esters
Source: Angew Chem Int Ed Engl. 2022 Apr 28;61(25):e202202621. doi: 10.1002/anie.202202621 (PMC9324207; doi:10.1002/anie.202202621)

checkCIF/PLATON report

Structure factors have been supplied for datablock(s) 28

THIS REPORT IS FOR GUIDANCE ONLY. IF USED AS PART OF A REVIEW PROCEDURE FOR PUBLICATION, IT SHOULD NOT REPLACE THE EXPERTISE OF AN EXPERIENCED CRYSTALLOGRAPHIC REFEREE.

No syntax errors found. CIF dictionary Interpreting this report

Datablock: 28

Bond precision: C-C = 0.0054 A Wavelength=0.71075

Cell:	a=7.5386(8)	b=7.5386(8)	c=42.598(5)
	alpha=90	beta=90	gamma=90
Temperature:	173 K		

	Calculated	Reported
Volume	2420.9(6)	2420.9(5)
Space group	P 43 21 2	P 43 21 2
Hall group	P 4nw 2abw	P 4nw 2abw
Moiety formula	C12 H10 F3 N3 O	C12 H10 F3 N3 O
Sum formula	C12 H10 F3 N3 O	C12 H10 F3 N3 O
Mr	269.23	269.23
Dx,g cm-3	1.477	1.477
Z	8	8
Mu (mm-1)	0.129	0.129
F000	1104.0	1104.0
F000’	1104.73	
h,k,lmax	9,9,51	9,9,50
Nref	2213[1415]	2212
Tmin,Tmax	0.987,0.999	0.751,0.999
Tmin’	0.987	

Correction method= # Reported T Limits: Tmin=0.751 Tmax=0.999
AbsCorr = MULTI-SCAN

Data completeness= 1.56/1.00 Theta(max)= 25.352

R(reflections)= 0.0509(2129) wR2(reflections)= 0.1324(2212)

S = 1.140 Npar= 176

The following ALERTS were generated. Each ALERT has the format
test-name_ALERT_alert-type_alert-level.
Click on the hyperlinks for more details of the test.

Alert level B

```
PLAT417_ALERT_2_B Short Inter D-H..H-D       H2       ..H2       .       2.07 Ang.
                                                y,x,1-z  =      5_556 Check
```

Author Response: Genuine close contact.

Alert level C

```
STRVA01_ALERT_4_C           Flack test results are ambiguous.
           From the CIF: _refine_ls_abs_structure_Flack    0.400
           From the CIF: _refine_ls_abs_structure_Flack_su    0.300
PLAT340_ALERT_3_C Low Bond Precision on  C-C Bonds ..............    0.00542 Ang.
PLAT906_ALERT_3_C Large K Value in the Analysis of Variance ......      3.016 Check
PLAT911_ALERT_3_C Missing FCF Refl Between Thmin & STh/L=    0.600          2 Report
```

Alert level G

```
PLAT002_ALERT_2_G Number of Distance or Angle Restraints on AtSite          2 Note
PLAT032_ALERT_4_G Std. Uncertainty on Flack Parameter Value High .      0.300 Report
PLAT172_ALERT_4_G The CIF-Embedded .res File Contains DFIX Records          1 Report
PLAT242_ALERT_2_G Low    'MainMol' Ueq as Compared to Neighbors of        C11 Check
PLAT434_ALERT_2_G Short Inter HL..HL Contact F2       ..F3       .       2.82 Ang.
                                      -1/2+x,5/2-y,5/4-z  =      8_476 Check
PLAT791_ALERT_4_G Model has Chirality at C5A         (Sohnke SpGr)          S Verify
PLAT791_ALERT_4_G Model has Chirality at C8          (Sohnke SpGr)          S Verify
PLAT791_ALERT_4_G Model has Chirality at C8A         (Sohnke SpGr)          R Verify
PLAT860_ALERT_3_G Number of Least-Squares Restraints ............           1 Note
PLAT978_ALERT_2_G Number C-C Bonds with Positive Residual Density.          2 Info
```

0 **ALERT level A** = Most likely a serious problem - resolve or explain
1 **ALERT level B** = A potentially serious problem, consider carefully
4 **ALERT level C** = Check. Ensure it is not caused by an omission or oversight
10 **ALERT level G** = General information/check it is not something unexpected

0 ALERT type 1 CIF construction/syntax error, inconsistent or missing data
5 ALERT type 2 Indicator that the structure model may be wrong or deficient
4 ALERT type 3 Indicator that the structure quality may be low
6 ALERT type 4 Improvement, methodology, query or suggestion
0 ALERT type 5 Informative message, check

It is advisable to attempt to resolve as many as possible of the alerts in all categories. Often the minor alerts point to easily fixed oversights, errors and omissions in your CIF or refinement strategy, so attention to these fine details can be worthwhile. In order to resolve some of the more serious problems it may be necessary to carry out additional measurements or structure refinements. However, the purpose of your study may justify the reported deviations and the more serious of these should normally be commented upon in the discussion or experimental section of a paper or in the "special_details" fields of the CIF. checkCIF was carefully designed to identify outliers and unusual parameters, but every test has its limitations and alerts that are not important in a particular case may appear. Conversely, the absence of alerts does not guarantee there are no aspects of the results needing attention. It is up to the individual to critically assess their own results and, if necessary, seek expert advice.

Publication of your CIF in IUCr journals

A basic structural check has been run on your CIF. These basic checks will be run on all CIFs submitted for publication in IUCr journals (*Acta Crystallographica*, *Journal of Applied Crystallography*, *Journal of Synchrotron Radiation*); however, if you intend to submit to *Acta Crystallographica Section C* or *E* or *IUCrData*, you should make sure that full publication checks are run on the final version of your CIF prior to submission.

Publication of your CIF in other journals

Please refer to the *Notes for Authors* of the relevant journal for any special instructions relating to CIF submission.

PLATON version of 20/01/2022; check.def file version of 19/01/2022

Datablock 28 - ellipsoid plot

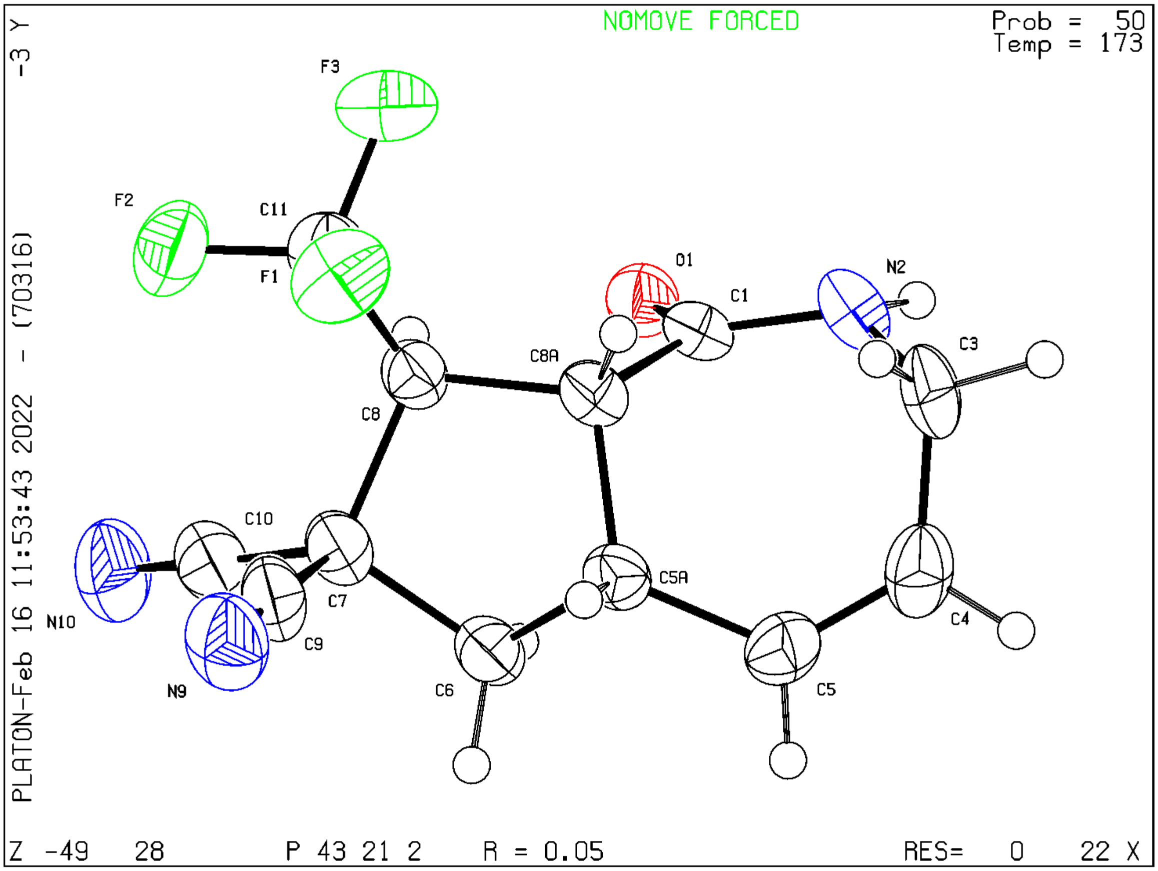

Supplement: Supplementary file 9 — Supporting Information [file ANIE-61-0-s004.pdf]
