# Supplementary material for: Cooperative Palladium/Isothiourea Catalyzed Enantioselective Formal (3+2) Cycloaddition of Vinylcyclopropanes and α,β‐Unsaturated Esters
Source: Angew Chem Int Ed Engl. 2022 Apr 28;61(25):e202202621. doi: 10.1002/anie.202202621 (PMC9324207; doi:10.1002/anie.202202621)
Supplement: Supplementary file 10 — Supporting Information [file ANIE-61-0-s008.pdf]

Angewandte Chemie
Eine Zeitschrift der Gesellschaft Deutscher Chemiker

Supporting Information

Cooperative Palladium/Isothiourea Catalyzed Enantioselective Formal (3+2) Cycloaddition of Vinylcyclopropanes and α,β-Unsaturated Esters

*J. Bitai, A. J. Nimmo, A. M. Z. Slawin, A. D. Smith**

Supporting Information

Table of Contents

1 General Information 2

2 General Procedures 4

3 Evaluation of reaction conditions (additional results) 7

4 Unsuccessful Substrates 17

5 Proposed Effect of LiCl on Stereochemical Outcome 18

6 Isomerization Studies 20

7 Synthesis of Catalysts 27

8 Preparation of vinylcyclopropanes 31

9 Preparation of Michael acceptors 36

10 Cooperative Pd and ITU catalysis products 58

11 Gram scale catalytic reaction 79

12 Product Derivatisations 79

13 Single crystal X-ray diffraction analysis 89

14 References 92

Appendix I: ^{1}H, ^{13}C{^{1}H}, ^{19}F{^{1}H} and ^{31}P{^{1}H} NMR spectra of novel compounds 95

Appendix II: HPLC and GC traces of novel compounds 203

1 General Information

All reagents and solvents were obtained from commercial suppliers and were used without further purification unless otherwise stated. Purification was carried out according to standard laboratory methods. Tetramisole·HCl was obtained from Sigma-Aldrich. $Pd_2dba_3·CHCl_3$ was purchased from Strem Chemicals Inc. and recrystallized from $CHCl_3$/acetone following the procedure reported by Ananikov and co-workers.[1]

Reactions involving moisture sensitive reagents were carried out in flame-dried glassware under an inert atmosphere (N_2 or Ar) using standard vacuum line techniques. Anhydrous solvents (Et_2O, CH_2Cl_2, THF and toluene) were obtained after passing through an alumina column (Mbraun SPS-800). Petrol is defined as petroleum ether 40–60 °C.

Room temperature (rt) refers to 20–25 °C. Temperatures of 0 °C and –78 °C were obtained using ice/water and CO_2(s)/acetone baths, respectively. Temperatures of 0 °C to –78 °C for overnight reactions were obtained using an immersion cooler (HAAKE EK 90) with EtOH or acetone as bath medium. Reactions involving heating were performed using DrySyn blocks and a contact thermocouple.

Under reduced pressure refers to the use of either a Büchi Rotavapor R-200 with a Büchi V-491 heating bath and Büchi V-800 vacuum controller, a Büchi Rotavapor R-210 with a Büchi V-491 heating bath and Büchi V-850 vacuum controller, a Heidolph Laborota 4001 with vacuum controller, an IKA RV10 rotary evaporator with a IKA HB10 heating bath and ILMVAC vacuum controller, or an IKA RV10 rotary evaporator with a IKA HB10 heating bath and Vacuubrand CVC3000 vacuum controller. Rotary evaporator condensers are fitted to Julabo FL601 Recirculating Coolers filled with ethylene glycol and set to –5 °C.

Analytical thin layer chromatography (TLC) was performed on pre-coated aluminium plates (Kieselgel 60 F254 silica) and visualisation was achieved using ultraviolet light (254 nm) and/or staining with either aqueous $KMnO_4$ solution, ethanolic phosphomolybdic acid, or ethanolic Vanillin solution followed by heating. Manual column chromatography was performed in glass columns fitted with porosity 3 sintered discs over Kieselgel 60 silica or Millipore® Silica Gel 60 (for aryl ester compounds) using the solvent system stated. Automated chromatography was performed on a Biotage

Isolera Four running Biotage OS578 with a UV/Vis detector using the method stated and cartridges filled with Kieselgel 60 silica or a Biotage Selekt running SELEKT 1.4.2-13403 with a UV/Vis detector using the method stated and Biotage Sfär Silica D 60 μm cartridges.

Melting points were recorded on an Electrothermal 9100 melting point apparatus, (dec) refers to decomposition.

Optical rotations were measured on a Perkin Elmer Precisely/Model-341 polarimeter operating at the sodium D line with a 100 mm path cell at 20 °C. Concentrations (*c*) are stated in g/100 mL.

HPLC analyses were obtained on either a Shimadzu HPLC consisting of a DGU-20A5 degassing unit, LC-20AT liquid chromatography pump, SIL-20AHT autosampler, CMB-20A communications bus module, SPD-M20A diode array detector and a CTO-20A column oven or a Shimadzu HPLC consisting of a DGU-20A5R degassing unit, LC-20AD liquid chromatography pump, SIL-20AHT autosampler, SPD-20A UV/Vis detector and a CTO-20A column oven. Separation was achieved using either a DAICEL CHIRALCEL OD-H column or DAICEL CHIRALPAK AD-H, AS-H, IA and IC columns using the method stated. HPLC traces of enantiomerically enriched compounds were compared with authentic racemic spectra.

GC analyses were obtained on a Shimadzu GC consisting of a Shimadzu AOC-20i auto injector and a Shimadzu GC-2025 gas chromatograph. Analysis was performed using Shimadzu GCsolution v2.41 software and separation was achieved using a Restek Rt-βDEXcst column (length: 30 m, thickness: 0.25 mm, film thickness: 0.25 μm).

Infrared spectra were recorded on a Shimadzu IRAffinity-1 Fourier transform IR spectrophotometer fitted with a Specac Quest ATR accessory (diamond puck). Spectra were recorded of either thin films or solids, with characteristic absorption wavenumbers (ν_{max}) reported in cm^{-1}.

^{1}H, $^{13}C\{^{1}H\}$, $^{19}F\{^{1}H\}$ and $^{31}P\{^{1}H\}$ NMR spectra were acquired on either a Bruker AV400 with a BBFO probe (^{1}H 400 MHz; $^{19}F\{^{1}H\}$ 377 MHz, $^{31}P\{^{1}H\}$ 162 MHz), a Bruker AVII 400 with a BBFO probe (^{1}H 400 MHz; $^{19}F\{^{1}H\}$ 376 MHz, $^{31}P\{^{1}H\}$ 162 MHz), a Bruker AVIII-HD 500 with a SmartProbe BBFO+ probe (^{1}H 500 MHz, $^{13}C\{^{1}H\}$ 126 MHz, $^{31}P\{^{1}H\}$ 202 MHz) or a Bruker AVIII 500 with a CryoProbe Prodigy BBO probe (^{1}H 500 MHz, $^{13}C\{^{1}H\}$ 126 MHz,

$^{31}P\{^1H\}$ 202 MHz) in the deuterated solvent stated. All chemical shifts are quoted in parts per million (ppm) relative to the residual solvent peak. All coupling constants, *J*, are quoted in Hz. Multiplicities are indicated as s (singlet), d (doublet), t (triplet), q (quartet), p (pentet), m (multiplet), and multiples thereof. The abbreviation Ar denotes aromatic, app denotes apparent and br denotes broad. NMR peak assignments were confirmed using 2D ^1H correlated spectroscopy (COSY), 2D ^1H nuclear Overhauser effect spectroscopy (NOESY), 2D ^1H–^{13}C heteronuclear multiple-bond correlation spectroscopy (HMBC), and 2D ^1H–^{13}C heteronuclear single quantum coherence (HSQC) where necessary.

Mass spectrometry (*m*/*z*) data were acquired by either electrospray ionisation (ESI), electron impact (EI), or nanospray ionisation (NSI) at either the University of St Andrews Mass Spectrometry Facility, the EPSRC UK National Mass Spectrometry Facility at Swansea University or SIRCAMS at University of Edinburgh.

2 General Procedures

2.1 General Procedure A: Preparation of vinyl cyclopropanes

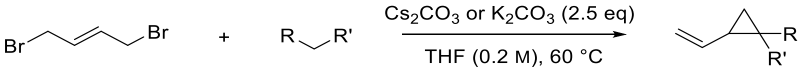

Adapting the procedure reported by Plietker and co-workers[2], malonate derivative (1.0 eq) and 1,4-dibromobut-2-ene (1.0 eq) were dissolved in THF (0.2 M) under N_2 atmosphere, followed by the addition of Cs_2CO_3 or K_2CO_3 (2.5 eq). The mixture was heated at reflux until TLC indicated full conversion of starting materials, subsequently allowed to cool to room temperature and filtered over Celite with Et_2O (equal volume). The combined organic phases were washed with sat. aq. $NaHCO_3$ (equal volume), H_2O and brine, dried over $MgSO_4$, filtered and the solvent removed under reduced pressure. The crude product was purified by silica column chromatography as specified.

2.2 General Procedure B: Basic ester hydrolysis with LiOH

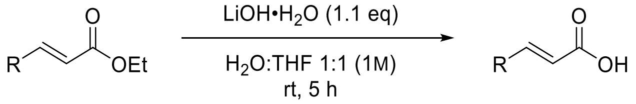

Adopting the procedure reported by Smith and co-workers[3], the corresponding ethyl ester (1.0 eq) was dissolved in a 1:1 mixture of H_2O : THF (1 M), followed by the addition of $LiOH{\cdot}H_2O$ (1.1 eq). The reaction mixture was stirred at room temperature for the time stated and subsequently adjusted to pH 2 with 2 M HCl. The aqueous phase was extracted with CH_2Cl_2 (3 × equal volume), the combined organic phases were dried over $MgSO_4$, filtered and the solvent removed under reduced pressure to afford the corresponding carboxylic acid, which was used without further purification.

2.3 General Procedure C: Acidic ester hydrolysis

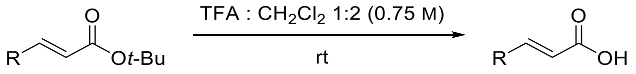

Adopting the procedure reported by Smith and co-workers,[3] the corresponding *t*-butyl ester (1.0 eq) was dissolved in TFA : CH_2Cl_2 1:2 (0.75 M). The reaction mixture was stirred at room temperature overnight and the solvent was removed under reduced pressure to afford the corresponding carboxylic acid, which was used without further purification.

2.4 General Procedure D: Preparation of α,β-unsaturated aryl oxide esters

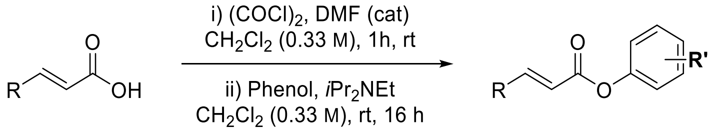

Following the procedure reported by Smith and co-workers[4], oxalyl chloride (1.05 eq) and DMF (cat.) were added to a stirred solution of α,β-unsaturated acid (1.0 eq) in anhydrous CH_2Cl_2 (0.33 M) at room temperature under N_2 atmosphere. The mixture was allowed to stir for 1 h, after which a solution of the corresponding phenol (1.0 eq) and *i*-Pr_2NEt (2.0 eq) in anhydrous CH_2Cl_2 (0.33 M) was added dropwise and the mixture allowed to stir overnight. The solvent was removed under reduced pressure and the crude product purified as specified.

2.5 General Procedure E: Cooperative Pd/ITU formal (3+2) cycloaddition

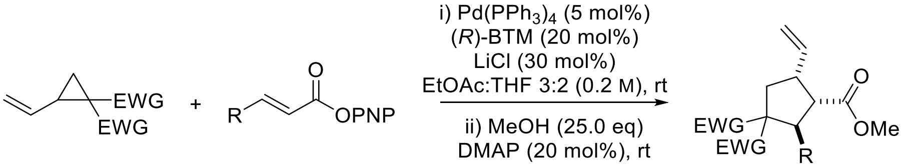

Prior to the reaction, EtOAc and anhydrous THF were purged with Ar for 30 min. An oven-dried Schlenk tube containing a magnetic stir bar was charged with $Pd(PPh_3)_4$ (5 mol%), (*R*)-BTM (20 mol%) and PNP ester (1.0 eq) and evacuated and flushed with N_2 three times. LiCl in THF (30 mol%, 0.5 M) was added followed by the remaining amount of THF. Vinylcyclopropane (1.0 eq) was added as a stock solution in EtOAc (118 mg/mL) followed by the addition of remaining EtOAc. The reaction was stirred at room temperature (25 °C) and the reaction progress monitored by ^1H NMR analysis. Once the starting materials had been consumed, anhydrous MeOH (25.0 eq) and DMAP (20 mol%) were added. No further precautions to exclude air or moisture were necessary at this point. The reaction mixture was stirred at room temperature until complete by ^1H NMR analysis (usually 24 h) and filtered over a short plug of silica with EtOAc. The EtOAc filtrate was washed with 1 M NaOH (2 × equal volume) and brine (1 × equal volume), dried over $MgSO_4$, filtered and the solvent removed under reduced pressure. The crude product was purified by silica column chromatography as specified.

Racemic samples were prepared using (±)-TM·HCl (10 mol%), *i*-Pr_2NEt (10 mol%) and LiCl (20 mol%) in acetone (0.2 M).

3 Evaluation of reaction conditions (additional results)

3.1 General Procedure for the optimisation of cooperative catalysis conditions

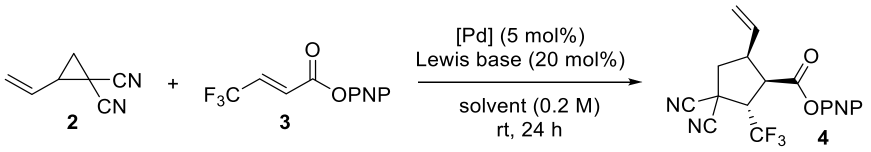

An oven-dried 4 mL vial containing a magnetic stir bar and equipped with a Teflon septum insert screw cap was charged with palladium catalyst [Pd] (5 mol%), isothiourea catalyst (20 mol%) and (*E*)-4,4,4-trifluorobut-2-enoic acid, 4-nitrophenyl ester **3** (1.0 eq). The vial was evacuated and flushed with N_2 three times. The appropriate solvent (0.2 M), purged prior with Ar for 30 minutes, was added followed by the addition of 2-vinylcyclopropane-1,1-dicarbonitrile **2** (1.0 eq). The reaction vial was sealed and allowed to stir at room temperature for 24 h. 1,3,5-Trimethoxybenzene (0.33 eq, 0.33 M solution in acetone) was added at the end of the reaction and the reaction mixture filtered over a short plug of silica with EtOAc. The solvent was removed under reduced pressure and the crude mixture was analysed by ^{1}H and ^{19}F-NMR and chiral stationary phase HPLC.

3.2 Calculation of ^{1}H NMR yield and dr

As an example, ^{1}H NMR spectra from a typical reaction from the optimisation process have been chosen and the majority of peaks assigned to the corresponding compounds. The first example depicts a reaction with partial conversion to product and remaining, unreacted starting materials. This example highlights that the chosen internal standard, which shows a singlet at 6.10 ppm, does not overlap with any other signals. In addition, the conversion of starting materials can also be assessed, if required, by integrating the signals at 6.75 ppm (dq, green dot) and at 1.86 ppm (dd, blue dot). The second example depicts the more usual case with full conversion of starting materials, which will be used to demonstrate how the ^{1}H NMR yield was derived during the optimisation process.

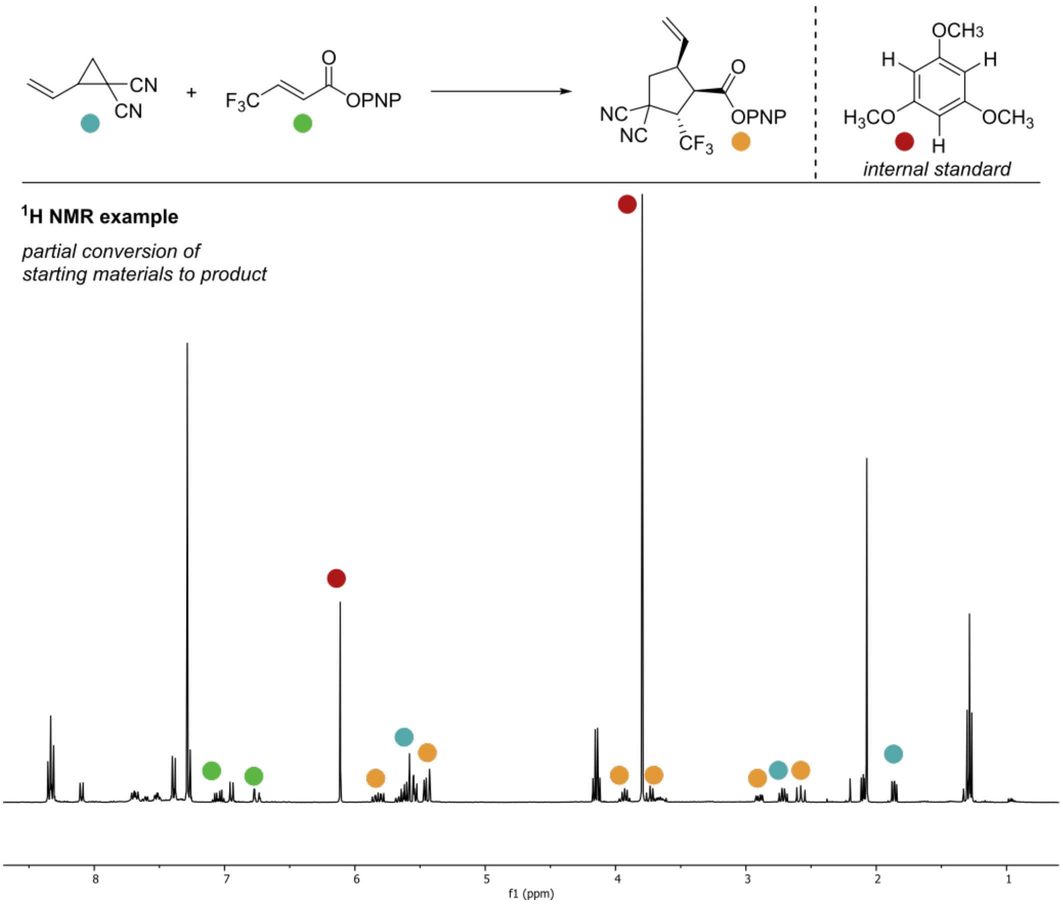
CN
CN
+
F3C
O
OPNP
NC
NC
CF3
OPNP
O
OCH3
H
H
H3CO
OCH3
H
internal standard
1H NMR example
partial conversion of
starting materials to product
8
7
6
5
4
3
2
1
f1 (ppm)

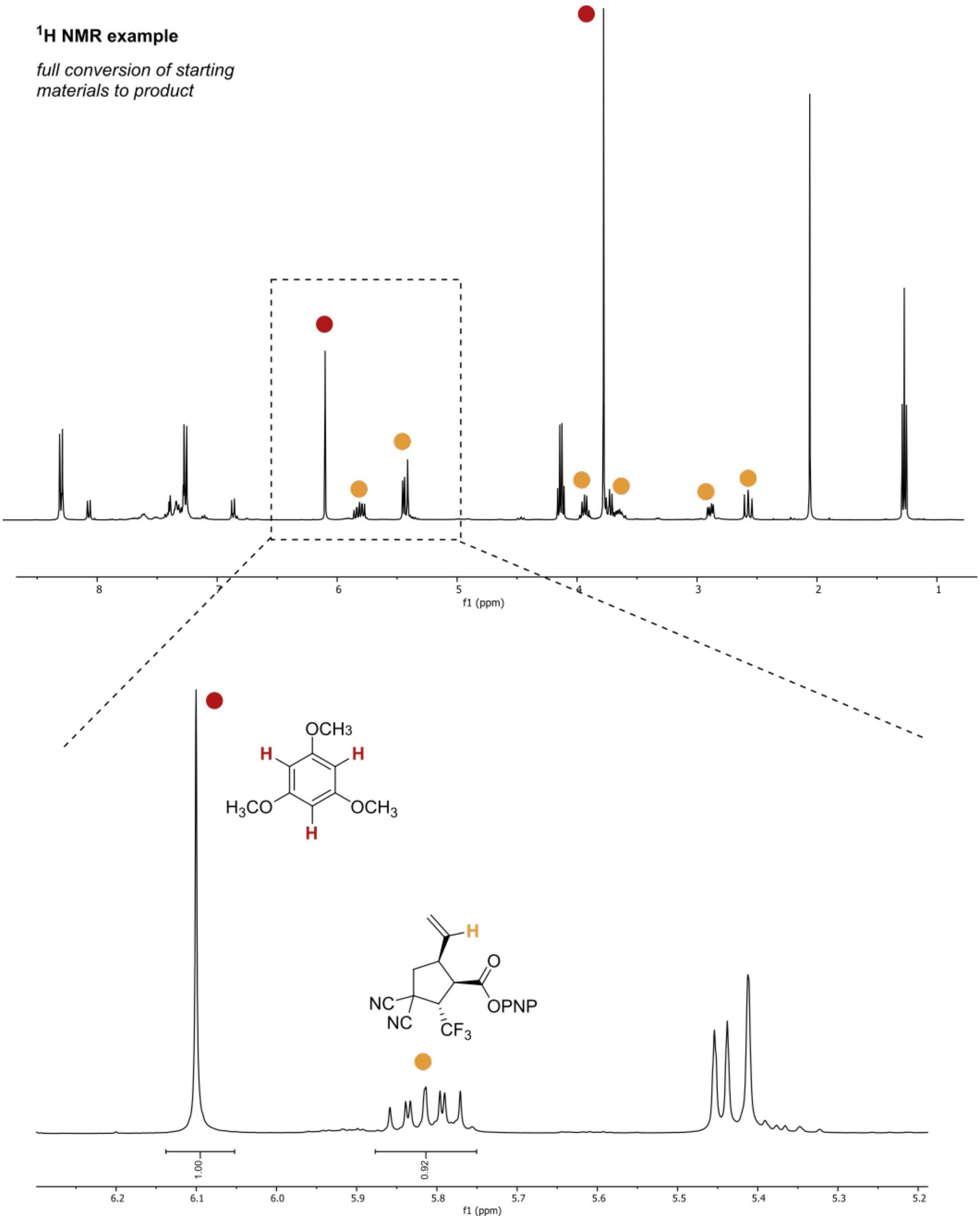

Determination of ^{1}H NMR yield:

The peak corresponding to the aromatic protons of the internal standard (highlighted in red) and the multiplet corresponding to the vinylic proton of the product (highlighted in yellow) were chosen as reference signals to determine the ^{1}H NMR yield (see figure above). As the internal standard peak corresponds to 3 protons, but the multiplet (ddd) of the product only to 1 proton, only 0.33 eq of internal standard are used to allow a direct comparison of the integrals. The integral of the internal standard peak is set to 1.0. Integration of the product multiplet (0.92) and multiplication by 100 gives the corresponding ^{1}H NMR yield (92%).

Determination of dr by $^{19}F\{^1H\}$ NMR analysis:

The dr is determined by $^{19}F\{^1H\}$ NMR analysis of the crude reaction mixture. The singlets corresponding to the CF_3 group of the two major diastereoisomers at −67.0 ppm and −67.3 ppm are integrated and the sum of the two integrals set to 100. The resulting value for the integrals, rounded to the nearest integer corresponds to the diastereomeric ratio of the product (9:91 dr in this example).

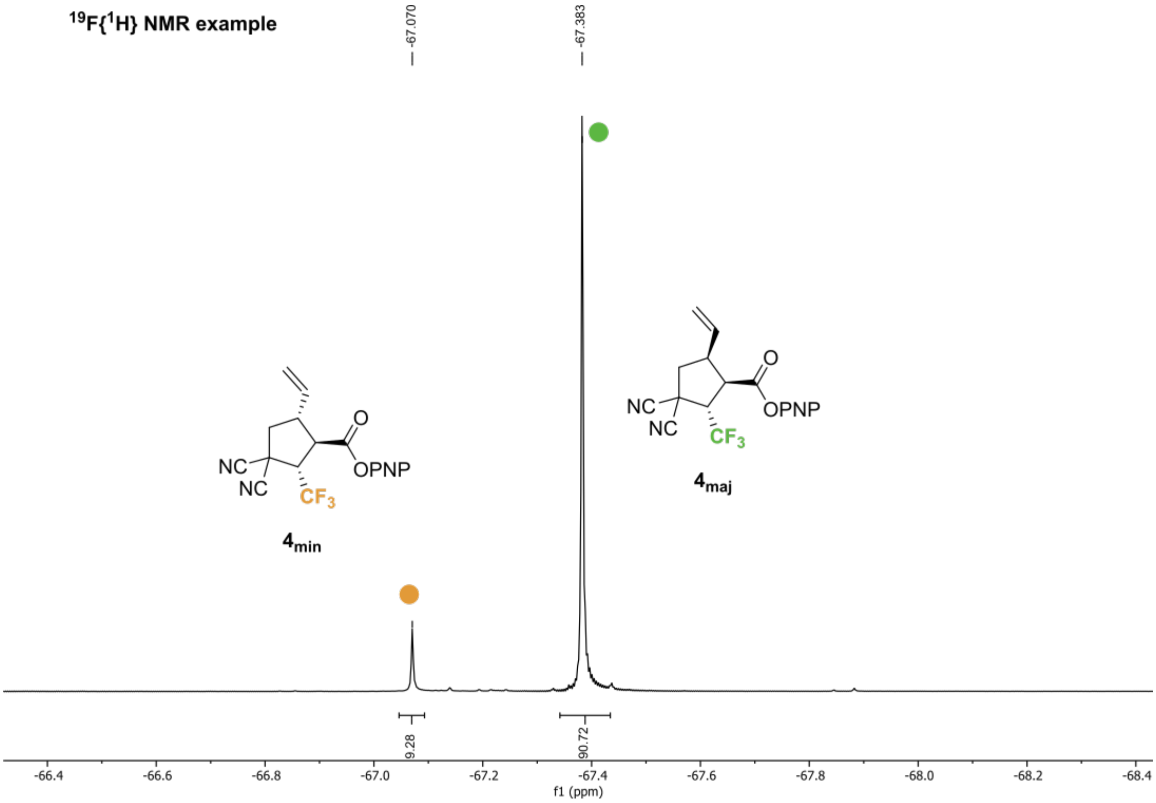

3.3 Solvent Screen

Table S1 Evaluation of reaction solvents in the cooperative Pd and ITU catalysis.

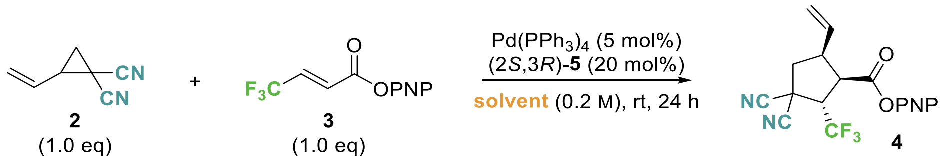

entry[a]	solvent	^{1}H NMR yield [%][b]	dr[c]	er_{maj}[d]	er_{min}[d]
1	CH_2Cl_2	(59)[e]	67:33	58:42	54:46
2	MeCN	83	70:30	59:41	64:36
3	DMF	87	81:19	78:22	91:9
4	THF	55	83:17	78:22	76:24
5	1,4-dioxane	49	82:18	62:38	69:31
6	Et_2O	62	67:33	52:48	53:47
7	TBME	63	70:30	51:49	55:45
8	acetone	72	81:19	75:25	76:24
9	*i*-PrOAc	56	72:28	59:41	61:39
10	2-MeTHF	35	77:23	71:29	69:31

[a] Reactions performed on a 0.1 mmol scale. [b] Combined NMR yield of diastereoisomers determined by ^{1}H NMR analysis using 1,3,5-trimethoxybenzene as internal standard. [c] Determined by ^{19}F{^{1}H} NMR analysis of the crude material. [d] Determined by chiral stationary phase HPLC analysis. [e] Combined isolated yield of diastereoisomer.

3.4 Palladium Source and Ligand Screen

Table S2 Evaluation of palladium precursors and achiral ligands.

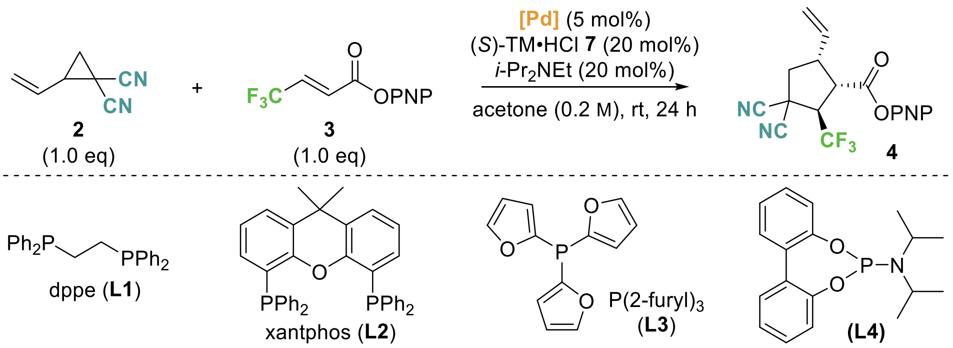

entry[a]	Pd source	ligand[b]	¹H NMR yield [%][c]	dr[d]	er_{maj}[e]	er_{min}[e]
1	$Pd(PPh_3)_4$	-	81	95:5	15:85	-
2	$Pd(dba)_2$	-	0	-	-	-
3	$Pd_2dba_3{\cdot}CHCl_3$	-	0	-	-	-
4	$Pd_2dba_3{\cdot}CHCl_3$	**L1**	0	-	-	-
5	$Pd_2dba_3{\cdot}CHCl_3$	**L2**	81	76:24	14:86	8:92
6	$Pd_2dba_3{\cdot}CHCl_3$	**L3**	50	>95:5	17:83	-
7	$Pd_2dba_3{\cdot}CHCl_3$	**L4**	49	87:13	29:71	16:84
8	$Pd(PPh_3)_4$[f]	-	46	95:5	17:83	-

[a] Reactions performed on a 0.1 mmol scale. [b] 20 mol% of monodentate ligands (L3,L4) or 10 mol% of bidentate ligands (L1, L2) used. [c] Combined NMR yield of diastereoisomers determined by ¹H NMR analysis using 1,3,5-trimethoxybenzene as internal standard. [d] Determined by ¹⁹F{¹H} NMR analysis of the crude material. [e] Determined by chiral stationary phase HPLC analysis. [f] 2.5 mol% $Pd(PPh_3)_4$ used.

Table S3 Evaluation of chiral ligands

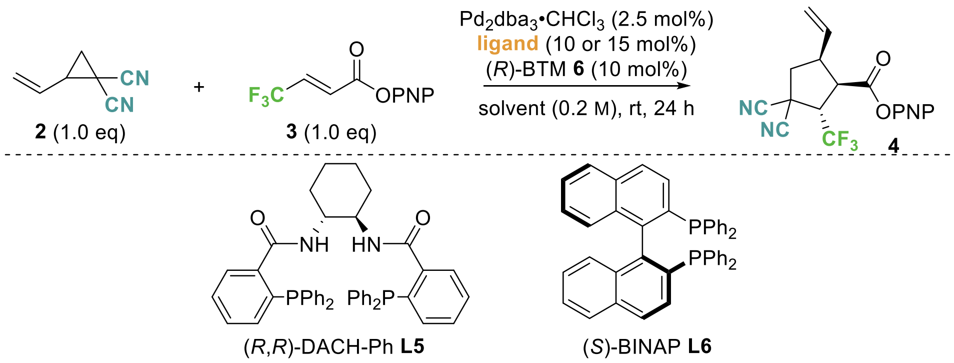

entry[a]	ligand (mol%)	solvent	^{1}H NMR yield [%][b]	dr[c]	er$_{maj}$[d]
1[e]	PPh_3 (15)	acetone	0	-	-
2[f]	PPh_3 (15)	acetone	77	75:25	70:30
3	(*R*,*R*)-DACH-Ph (10)	acetone	0	-	-
4	(*R*,*R*)-DACH-Ph (10)	toluene	0	-	-
5[g]	(*R*,*R*)-DACH-Ph (10)	toluene	55	62:38	34:66
6[g]	(*R*,*R*)-DACH-Ph (10)	THF	99	79:21	46:54
7	(S)-BINAP (10)	THF	15	56:44	83:17
8[g]	(S)-BINAP (10)	THF	28	53:47	87:13
9[h]	(S)-BINAP (10)	THF	17	89:11	65:35

[a] Reactions performed on a 0.15 mmol scale. [b] Combined NMR yield of diastereoisomers determined by ^{1}H NMR analysis using 1,3,5-trimethoxybenzene as internal standard. [c] Determined by ^{19}F{^{1}H} NMR analysis of the crude material. [d] Determined by chiral stationary phase HPLC analysis. [e] 10 mol% (S)-TM·HCl and 10 mol% i-Pr_2NEt used. [f] 48 h. [g] Reaction performed without (R)-BTM. [h] 30 mol% LiCl used.

3.5 Screening of aryloxide leaving group

Table S4 Evaluation of aryl oxide leaving groups.

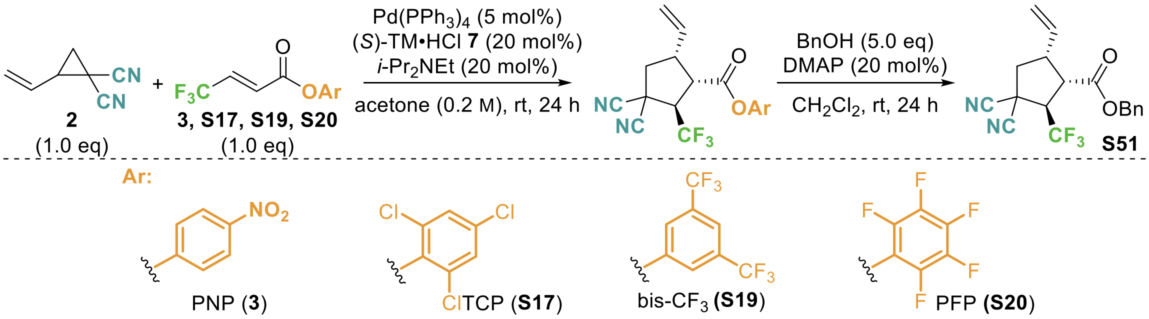

entry[a]	Ar	^{1}H NMR yield [%][b]	dr[c]	er_{maj}[d]	er_{min}[d]
1	PNP	81	95:5	15:85	-
2	TCP[e]	84	76:24	44:56	45:55
3	bis-CF$_3$	72	94:6	13:87	-
4	PFP[f]	45	94:6	16:84	-

[a] Reactions performed on a 0.1 mmol scale. [b] Combined NMR yield of diastereoisomers determined by ^{1}H NMR analysis of the crude material using 1,3,5-trimethoxybenzene as internal standard. [c] Determined by ^{19}F{^{1}H} NMR analysis of the crude material. [d] Determined from the benzyl ester product by chiral stationary phase HPLC analysis. [e] 72 h reaction time with BnOH. [f] 48 h reaction time with BnOH.

3.6 Screening of reaction temperature

Table S5 Influence of reaction temperature on the cooperative catalysis.

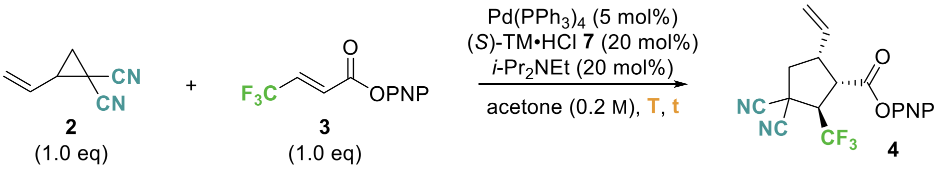

entry[a]	T [°C]	time [h]	^{1}H NMR yield [%][b]	dr[c]	er_{maj}[d]
1	25	24	81	95:5	15:85
2	0	24	42	>95:5	16:84
3	−22	96	86	>95:5	18:82

[a] Reactions performed on a 0.1 mmol scale. [b] Combined NMR yield of diastereoisomers determined by ^{1}H NMR analysis using 1,3,5-trimethoxybenzene as internal standard. [c] Determined by ^{19}F{^{1}H} NMR analysis of the crude material. [d] Determined by chiral stationary phase HPLC analysis.

3.7 Screening of substrate stoichiometry and concentration

Table S6 Influence of reactant stoichiometry and concentration.

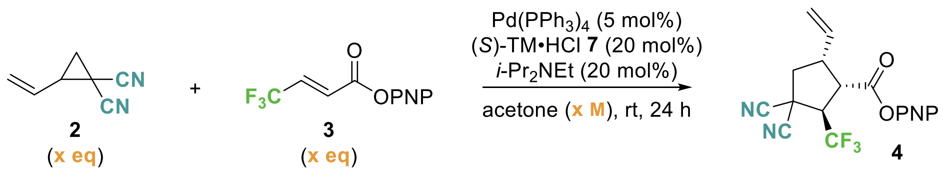

entry[a]	VCP : PNP	conc. [M]	^{1}H NMR yield [%][b]	dr[c]	er_{maj}[d]
1	1.0 : 1.0	0.2	81	95:5	15:85
2	1.5 : 1.0	0.2	88	>95:5	17:83
3	1.0 : 1.5	0.2	74	94:6	19:81
4	1.0 : 1.0	0.1	68	>95:5	16:84
5	1.0 : 1.0	0.4	70	>95:5	18:82
6	1.0 : 1.0	0.8	63	94:6	19:81

[a] Reactions performed on a 0.1 mmol scale. [b] Combined NMR yield of diastereoisomers determined by ^{1}H NMR analysis of the crude material using 1,3,5-trimethoxybenzene as internal standard. [c] Determined by ^{19}F{^{1}H} NMR analysis of the crude material. [d] Determined by chiral stationary phase HPLC analysis.

3.8 Screening of isothiourea catalysts in the presence of LiCl

Table S7 Evaluation of isothiourea catalysts in the presence of LiCl.

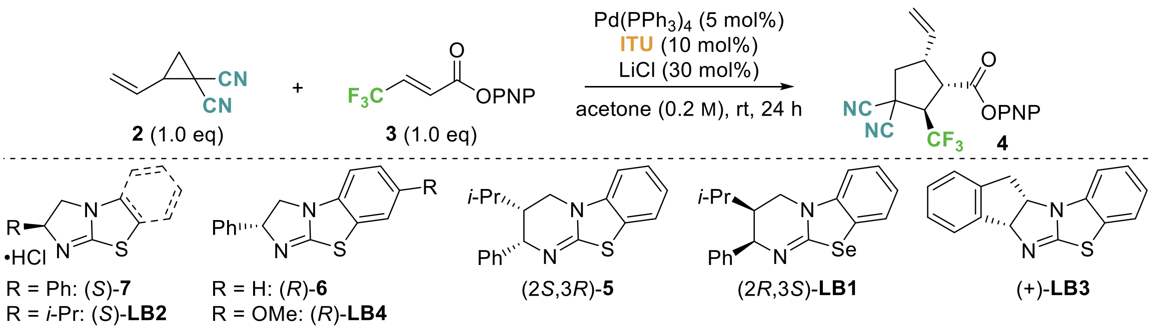

entry[a]	ITU	^{1}H NMR yield [%][b]	dr[c]	er_{maj}[d]
1	-	97	88:12	49:51
2	(*S*)TM·HCl **7**[e]	80	>95:5	18:82
3	(*R*)-BTM **6**	71	>95:5	84:16
4	(2*S*,3*R*)HyperBTM **5**	80	95:5	75:25
5	(2*R*,3*S*)HyperSe **LB1**	84	>95:5	17:83
6	(*S*)-*i*-PrBTM·HCl **LB2**[e]	89	>95:5	25:75
7	(+)-fused BTM **LB3**	90	>95:5	84:16
8	(*R*)-OMeBTM **LB4**	83	>95:5	85:15

[a] Reactions performed on a 0.1 mmol scale. [b] Combined NMR yield of diastereoisomers determined by ^{1}H NMR analysis using 1,3,5-trimethoxybenzene as internal standard. [c] Determined by ^{19}F{^{1}H} NMR analysis of the crude material. [d] Determined by chiral stationary phase HPLC analysis. [e] 10 mol% i-Pr$_2$NEt and 20 mol% LiCl used.

4 Unsuccessful Substrates

Unsuccessful Michael acceptors:

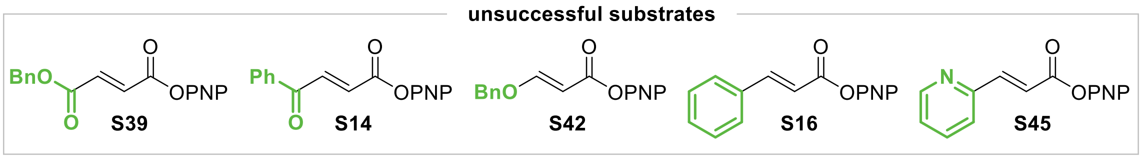

Notes: The use of benzyl ester containing Michael acceptor **S39** resulted in partial conversion of starting materials, with slow decomposition of product observed, indicative of an incompatibility of the benzyl ester functionality with the current dual catalytic process. Incorporation of a β-ketone substituent (**S14**) led to rapid conversion of starting materials, but resulted in a messy reaction mixture with no product signals detectable. Benzyl ether and 2-pyridine containing Michael acceptors **S42** and **S45** proved insoluble under reaction conditions, with no product formation detected. Phenyl containing substrate **S16** also showed no reactivity under the catalysis conditions. Notably, full consumption of VCP was observed in these three cases.

Previous investigations have shown that α,β-unsaturated acyl ammonium ions (and indeed acyl ammonium ions) are formed in only low concentration from a,b-unsaturated aryl esters (and anhydrides).[3,5] β-EWG substituents typically lead to increased reactivity of these intermediates due to increased electrophilicity, presumably increasing the rate of the initial Michael addition in the desired stepwise cyclisation process. To rationalise the observed outcomes (successful cyclopentane formation vs. only consumption of VCP), the relative rates of the cyclisation process against the dimerization/oligomerisation of the vinylcyclopropane needs to be considered. In the absence of a suitable activating β-substituent, the rate of dimerization/oligomerisation of the vinylcyclopropane seems to be higher than the rate for the desired cyclisation process, resulting only in the consumption of VCP.

Unsuccessful VCPs:

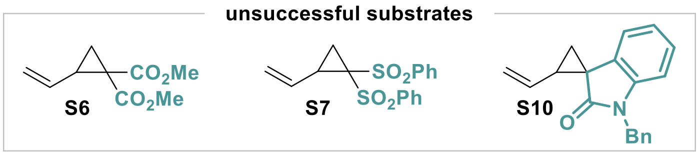

Note: only starting materials were returned in all 3 cases.

5 Proposed Effect of LiCl on Stereochemical Outcome

In general, the effect of halide ions in transition metal catalysis is well documented, and in the case of Cl^- ions has been rationalised as typically leading to an increase in the rate of π-σ-π isomerisation, attributed to enhanced stability caused by Cl^- coordination to an intermediate, tetracoordinate η^1-complex. This can be rationalised by considering the structural changes in the Pd-allyl complex during the isomerisation process. Transitioning from the η^3-complex to the η^1-complex generates a vacant coordination site (yellow box), which can be occupied by access ligand or a solvent molecule to provide additional stabilisation for the η^1-intermediate. In comparison to the η^3-complex, the η^1-complex is higher in energy, therefore, the η^3-η^1-η^3 isomerisation process is associated with a certain energy barrier. Increased stabilisation of the η^1-intermediate reduces the energy barrier to its formation, leading to an increase in rate for η^3-η^1-η^3 isomerisation. As Cl^- ions seem to provide particularly efficient stabilisation compared to excess ligand (e.g. PPh_3) or coordinating solvents (e.g. Et_2O), this simplified consideration can provide a rationale for the observed "halide effect".

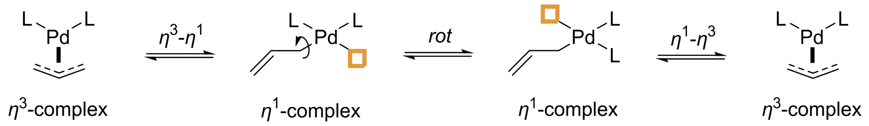

□ = *vacant coordination site occupied by access ligand or solvent for stabilisation*

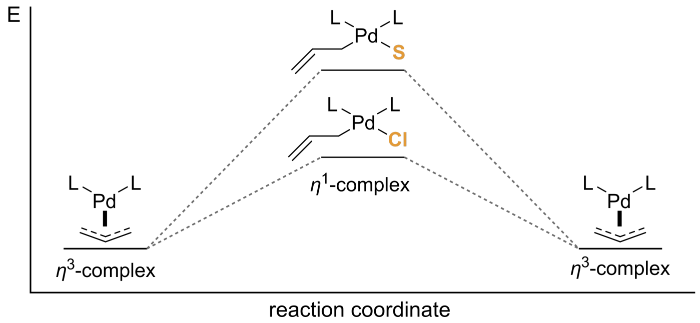

If this assumption is applied to the developed process, initial Michael addition is proposed to be unselective with regards to the allyl orientation, leading to a mixture of isomers A and B. In the absence of LiCl, isomerisation between these two isomers is assumed to be slow compared to subsequent intramolecular ring closure, leading to a low diastereoselectivity. However, in the presence of LiCl, the rate of π-σ-π-isomerisation is

increased, increasing the amount of intermediate A, which results in an increased diastereoselectivity in favour of the major (1*R*,2*S*,5*S*)-diastereoisomer.

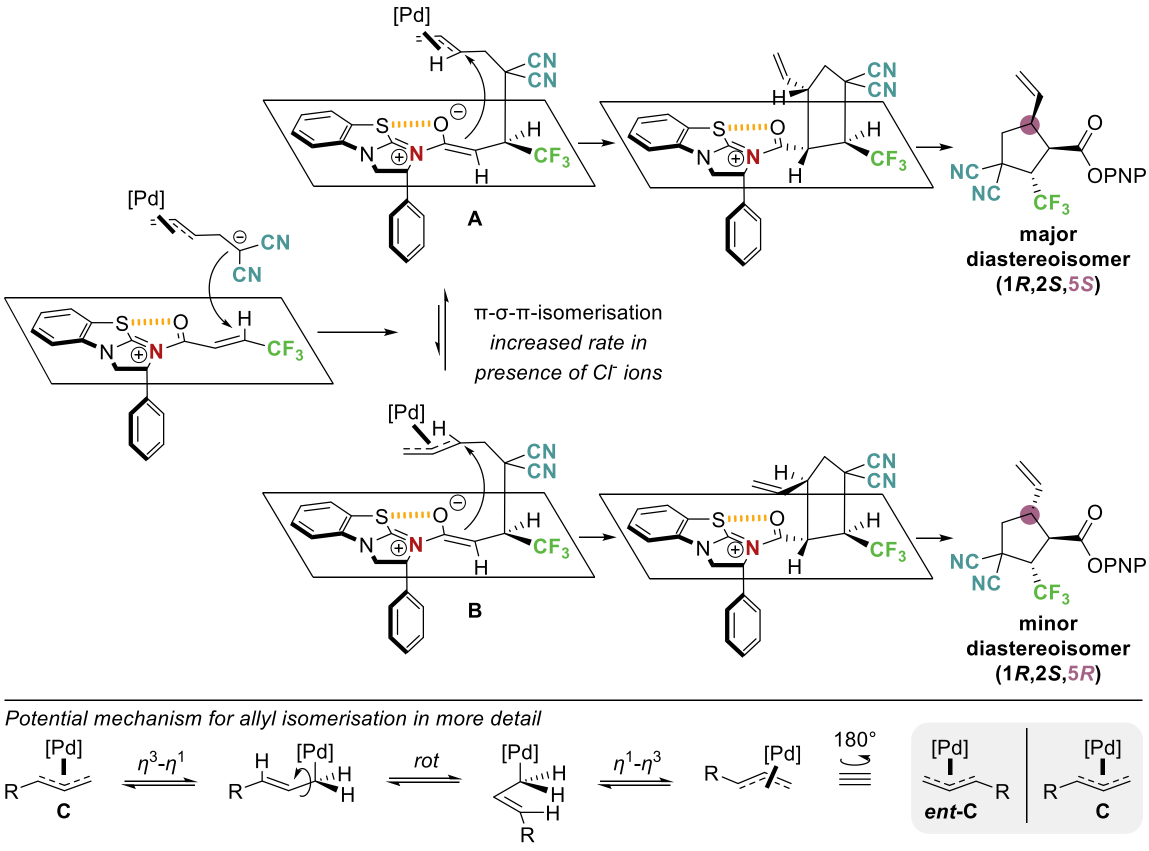

6 Isomerization Studies

6.1 In situ $^{19}F\{^1H\}$ NMR monitoring

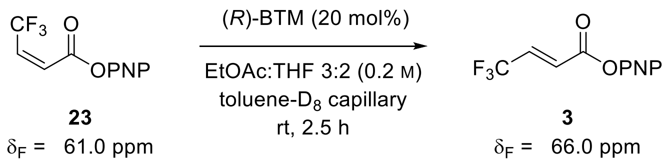

A sealed capillary containing toluene-D8 was placed into an NMR tube and loaded into the NMR spectrometer at room temperature. The sample was locked to toluene-D8 and shimmed. The tube was then removed from the spectrometer. PNP ester (*Z*)-**23** (52.2 mg, 0.20 mmol, 1.0 eq) and (*R*)-BTM (10.0 mg, 0.04 mmol, 0.2 eq) were dissolved in EtOAc:THF 3:2 (0.2 M) and immediately transferred into the NMR tube, which was returned to the NMR spectrometer and shimmed before the experiment was initiated. An automated loop sequence was used to acquire the $^{19}F\{^1H\}$ spectra [ns = 32, sweep width 80 ppm (spectral centre –60 ppm)].

General Considerations: $^{19}F\{^1H\}$ spectra were collected at 470 MHz. The initial spectrum was obtained 971 s after the reaction started. Subsequent spectra were then acquired every 425 s. 19 spectra were collected and processed using MestReNova 10.0 software. Time points were taken from the timestamps recorded in the MestReNova software. The ratios of PNP ester (*Z*)-**23** and (*E*)-**3** were calculated as a percentage, with the total amount of (*Z*)-**23** and (*E*)-**3** equal to 100%.

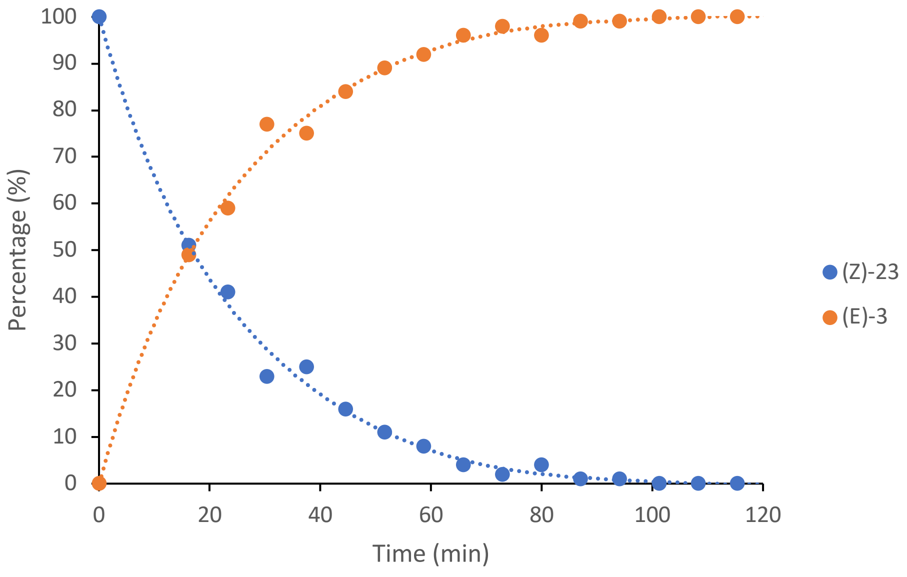

*Figure S1. Reaction profile for the $^{19}F\{^1H\}$ monitored isomerization of (Z)-**23** to (E)-**3**.*

Table S8 (*Z*)-**23** : (*E*)-**3** ratios determined by in situ $^{19}F\{^{1}H\}$ NMR with time.

**Time (min)**	**(*Z*)-23 : (*E*)-3**
0.0	100:0
16.2	51:49
23.3	41:59
30.3	23:77
37.4	25:75
44.5	16:84
51.6	11:89
58.7	8:92
65.8	4:96
72.8	2:98
79.9	4:96
87.0	1:99
94.1	0:100
101.2	0:100
108.3	0:100
115.4	0:100

Identification of reaction species

Excluding the PNP esters (*Z*)-**23** and (*E*)-**3**, two significant peaks were observed at –61.6 and –65.8 ppm respectively. The species at –61.6 ppm was present in the $^{19}F\{^{1}H\}$ spectrum of (*Z*)-**23** in EtOAc:THF 3:2 but was not present when the spectrum was taken in $CDCl_3$. The species at –65.8 ppm was shown to be **S18**, presumably resulting from the hydrolysis of (*E*)-**3** (or the corresponding α,β-unsaturated acyl ammonium species) under the reaction conditions. The identity of **S18** was confirmed by spiking the NMR sample with an authentic sample of **S18**.

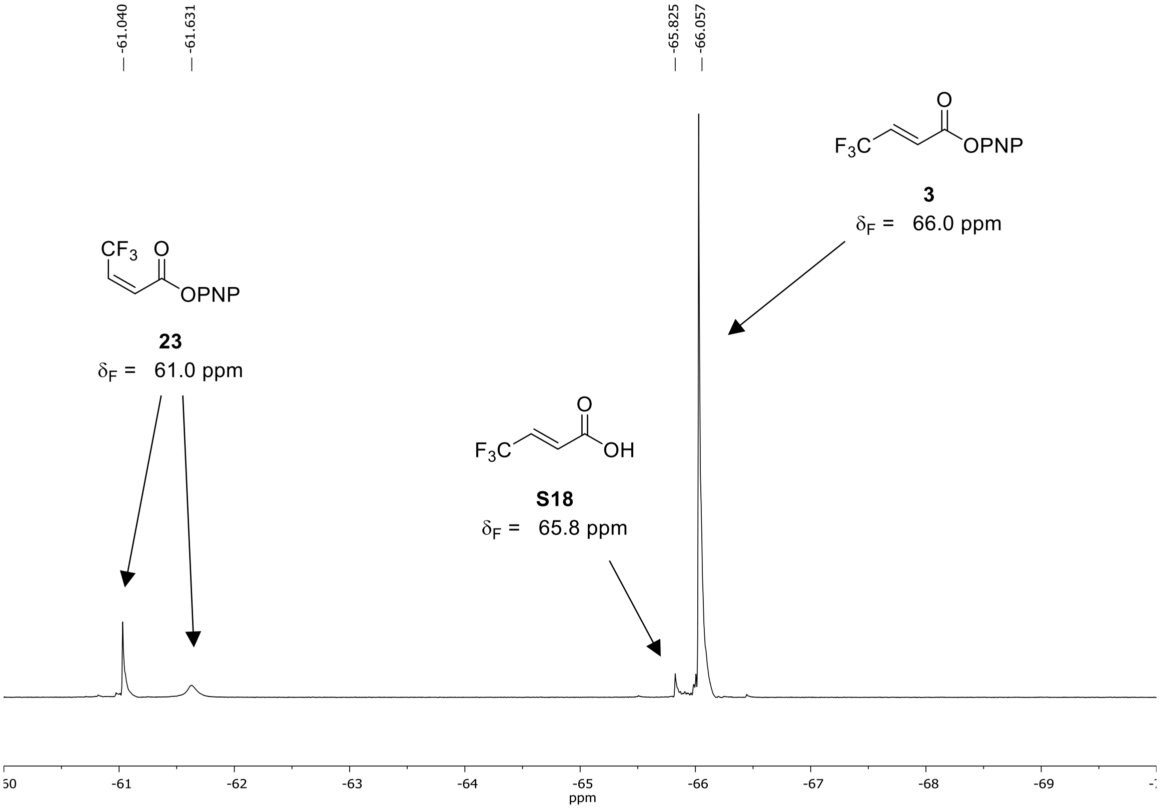

Figure S2. Representative $^{19}F\{^{1}H\}$ NMR spectrum showing all significant reaction species, ns = 32, sweep width 80 ppm (spectral centre –60 ppm), range shown: –60 - –70 ppm.

6.2 Maleate to fumarate ester isomerization

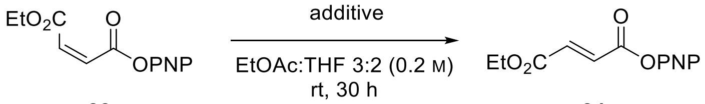

22 **24**

PNP ester (*Z*)-**22** (159 mg, 0.60 mmol, 1.0 eq) and additive(s) were weighed into an oven-dried 4 mL screw-top vial. The vial was capped and purged before addition of EtOAc:THF 3:2 (0.2 M) to start the reaction. Aliquots (0.1 mL) of the reaction mixture were taken at intervals and quenched with 1 M HCl (1 mL) then extracted with Et_2O (1 mL). The organic phase was dried over $MgSO_4$, filtered then concentrated to give a sample for ^{1}H NMR.

The additives tested in the isomerization process were:

i) (*R*)-BTM (20 mol%)

ii) NBu_4OPNP (1.0 eq)

iii) (*R*)-BTM (20 mol%) + NBu_4OPNP (1.0 eq)

iv) PPh_3 (20 mol%)

v) LiCl (30 mol%)

No isomerization of (*Z*)-**22** was observed in the absence of an additive or when PPh_3 (20 mol%) or LiCl (30 mol%) were added. The addition of either (*R*)-BTM (20 mol%) (Figure S3) or NBu_4OPNP (1.0 eq) (Figure S4) resulted in relatively slow isomerization giving a 13:87 and 53:47 mixture of (*Z*)-**22**:(*E*)-**24** PNP esters, respectively, after 24 h. Adding both (*R*)-BTM (20 mol%) and NBu_4OPNP (1.0 eq) resulted in rapid isomerization giving a 3:97 mixture of (*Z*)-**22**:(*E*)-**24** PNP esters in 2 h (Figure S5). A comparison of the rates of isomerization of (*Z*)-**22** from experiments i), ii), and iii) is shown in Figure S6. The formation of (*E*)-**24** is omitted for clarity.

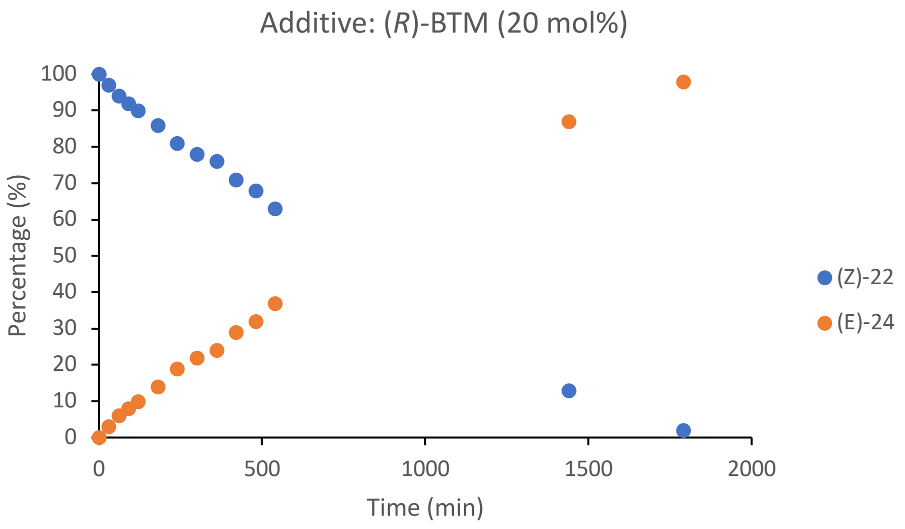

Figure S3. Reaction profile for isomerization reaction i) (R)-BTM (20 mol%).

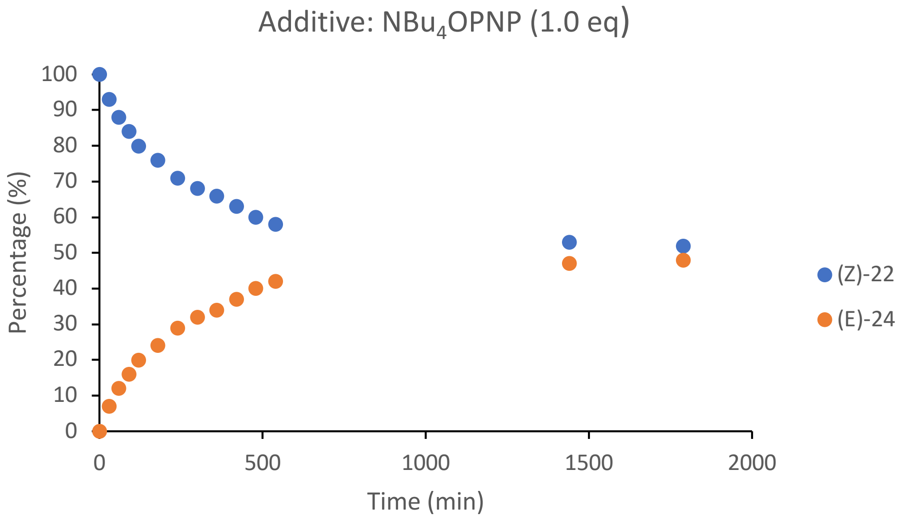

Figure S4. Reaction profile for isomerization reaction ii) NBu_4OPNP (1.0 eq).

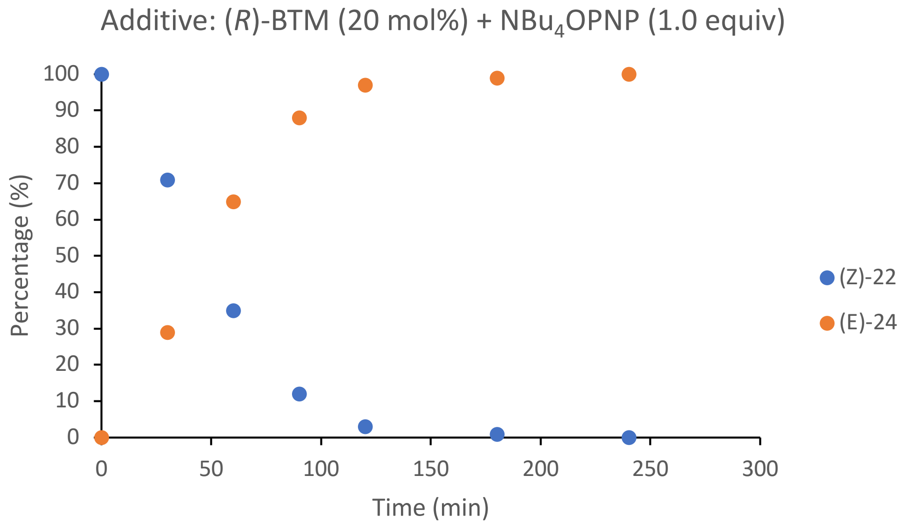

Figure S5. *Reaction profile for isomerization reaction iii) (R)-BTM (20 mol%) + NBu_4OPNP (1.0 eq).*

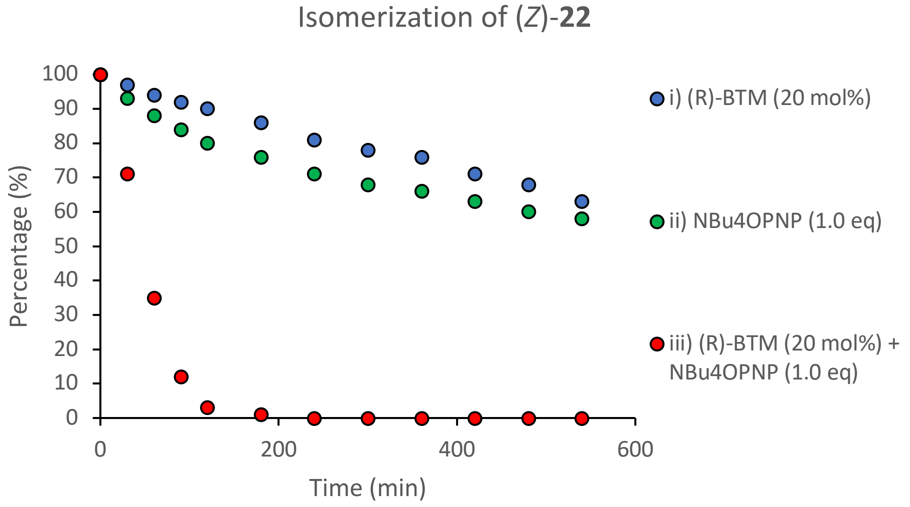

Figure S6. *Comparison of reaction profiles for the isomerization of (Z)-**22** over the first 9 h.*

Table S9 (*Z*)-**22** : (*E*)-**24** ratios determined by ^{1}H NMR with time.

**Time (min)**	**(*Z*)-22 : (*E*)-24**		
	**i)**	**ii)**	**iii)**
0	100:0	100:0	100:0
30	97:3	93:7	71:29
60	94:6	88:12	35:65
90	92:8	84:16	12:88
120	90:10	80:20	3:97
180	86:14	76:24	1:99
240	81:19	71:29	0:100
300	78:22	68:32	0:100
360	76:24	66:34	0:100
420	71:29	63:37	0:100
480	68:32	60:40	0:100
540	63:37	58:42	0:100
1440	13:87	53:47	0:100
1790	2:98	52:48	0:100

7 Synthesis of Catalysts

Isothiourea catalysts (*R*)-BTM[6] **6**, (2*S*,3*R*)-HyperBTM[7] **5**, (2*R*,3*S*)-HyperSe[5] **LB1** and (*R*)-OMe-BTM[8] **LB4** were synthesised according to published procedures.

(*S*)-2-Isopropyl-2,3-dihydrobenzo[d]imidazo[2,1-b]thiazole ((*S*)-*i*-Pr-BTM) (S1)

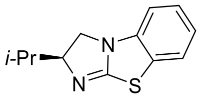

$C_{12}H_{14}N_2S$
218.32

Adapting the procedure from Shiina and co-workers,[9] a 50 mL pressure tube was charged with 2-chlorobenzothiazole (3.77 mL, 29.0 mmol, 1.0 eq), (*S*)-valinol (3.0 g, 29.0 mmol, 1.0 eq) and *i*-Pr_2NEt (7.4 mL, 43.5 mmol, 1.5 eq), sealed and the mixture stirred at 130 °C for 45 h. The reaction mixture was allowed to cool to room temperature, dissolved in 10 mL CH_2Cl_2 (gentle heating advantageous) and purified directly by silica column chromatography (2% *i*-PrOH to 6% *i*-PrOH in CH_2Cl_2, R_f 0.23 in 4% *i*-PrOH in CH_2Cl_2) to afford the intermediate (S)-2-(benzo[d]thiazol-2-ylamino)-3-methylbutan-1-ol as white solid (5.1 g, 74%).

^{1}H NMR (500 MHz, $CDCl_3$) δ_H: 0.92 (3H, d, *J* 6.8, C(3)H(C*H*$_3$)), 0.99 (3H, d, *J* 6.8, C(3)H(C*H*$_3$)), 1.88 – 1.99 (1H, m, C(3)*H*), 3.43 (1H, br s, C(2)*H*), 3.77 (1H, dd, *J* 11.7, 6.1, C(1)H^AH^B), 3.87 (1H, dd, *J* 11.7, 3.1, C(1)H^AH^B), 4.60 (1H, brs, N*H*), 6.59 (1H, brs, O*H*), 7.06 – 7.10 (1H, m, Ar(5)*H* or Ar(6)*H*), 7.26 – 7.31 (1H, m, Ar(5)*H* or Ar(6)*H*), 7.53 – 7.55 (1H, m, Ar(4)*H* or Ar(7)*H*), 7.55 – 7.57 (1H, m, Ar(4)*H* or Ar(7)*H*).

The intermediate alcohol (2.36 g, 10.0 mmol, 1.0 eq) was dissolved in anhydrous CH_2Cl_2 (100 mL). Et_3N (5.5 mL, 40.0 mmol, 4.0 eq) was added and the mixture cooled in an ice bath for 10 min. MsCl (1.0 mL, 13.0 mmol, 1.3 eq) was added dropwise, the reaction mixture warmed to room temperature and stirred until TLC (4% *i*-PrOH in CH_2Cl_2) indicated complete conversion (ca. 2 h). MeOH (0.6 mL) was added and the reaction mixture heated at reflux overnight. After cooling to room temperature, the mixture was washed with H_2O, dried over $MgSO_4$, filtered and the solvent removed under reduced pressure. The crude product was purified by silica column chromatography (hexane : *i*-PrOH : Et_3N 95:4:1, R_f 0.19) to afford (*S*)-*i*-Pr-BTM as pale yellow oil (2.1 g, 95%), which solidifies upon cooling.

$[\boldsymbol{\alpha}]_{\boldsymbol{D}}^{\boldsymbol{20}}$ −130.1 (*c* 1.05 in $CHCl_3$) {Lit.[9] $[\boldsymbol{\alpha}]_{\boldsymbol{D}}^{\boldsymbol{23}}$ −141.6 (*c* 0.35 in benzene)}.

chiral HPLC analysis Chiralcel AD-H (95:5 hexane : *i*-PrOH, flow rate 1.5 mlmin^{-1}, 211 nm, 40 °C) t_R (2*R*): 6.5 min, t_R (2*S*): 10.3 min, 0.1 : 99.9 er.

^{1}H NMR (500 MHz, $CDCl_3$) δ_H: 0.97 (3H, d, *J* 6.7, CH(C*H*$_3$)), 1.05 (3H, d, *J* 6.7, CH(C*H*$_3$)), 1.90 (1H, oct, *J* 6.7, C*H*(CH$_3$)$_2$), 3.49 (1H, app. t, *J* 8.7, C(3)*H*^{A}H^B), 3.87 (1H, dd, *J* 9.8, 8.9, C(3)H^A*H*^{B}), 4.38 (1H, ddd, *J* 9.8, 8.5, 6.5, C(2)*H*), 6.65 (1H, dd, *J* 7.7, 0.7, Ar(5)*H* or Ar(8)*H*), 6.93 (1H, td, *J* 7.7, 1.2, Ar(6)*H* or Ar(7)*H*), 7.17 (1H, td, *J* 7.7, 1.2, Ar(6)*H* or Ar(7)*H*), 7.24 – 7.27 (1H, m, Ar(5)*H* or Ar(8)*H*).

A racemic sample was prepared using (±)-valinol following the same procedure. Spectroscopic data in accordance with literature.[9]

(*S*)-2-Isopropyl-2,3-dihydrobenzo[d]imidazo[2,1-b]thiazole hydrochloride ((*S*)-*i*-Pr-BTM·HCl) (LB2)

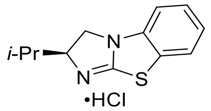

$C_{12}H_{15}ClN_2S$
254.78

To a solution of (*S*)-*i*-Pr-BTM **S1** (1.16 g, 5.3 mmol, 1.0 eq) in Et_2O (21 mL) was added HCl (2 M in Et_2O, 10.6 mmol, 2.0 eq) and the reaction mixture stirred for 5 min at room temperature. The solvent was removed under reduced pressure to yield the title compound as a white solid (1.34 g, 99%). **m.p.** (Et_2O)229 – 230 °C.

$[\alpha]_D^{20}$ −85.5 (*c* 1.08 in MeCN).

^{1}H NMR (500 MHz, DMSO-*d*$_6$) δ_H: 0.95 (3H, d, *J* 6.7, CH(C*H*$_3$)), 0.99 (3H, d, *J* 6.7, CH(C*H*$_3$)), 2.01 (1H, oct, *J* 6.7, C*H*(CH$_3$)$_2$), 4.28 – 4.34 (1H, m, C(3)*H*^{A}H^B), 4.55 – 4.66 (2H, m, C(2)*H* and C(3)H^A*H*^{B}), 7.37 (1H, ddd, *J* 8.4, 7.3, 1.3, Ar(7)*H*), 7.50 (1H, dd, *J* 8.1, 1.2, Ar(5)*H*), 7.53 – 7.58 (1H, m, Ar(6)*H*), 7.99 – 8.05 (1H, m, Ar(8)*H*), 11.68 (1H, s, N*H*).

^{13}C{^{1}H} NMR (126 MHz, DMSO-*d*$_6$) δ_C: 18.0 (CH(CH$_3$)), 18.1 (CH(CH$_3$)), 32.4 (*C*H(CH$_3$)$_2$), 48.8 (*C*(3)H$_2$), 70.3 (*C*(2)H), 113.0 (Ar*C*(5)H), 124.9 (Ar*C*(7)H), 125.3 (Ar*C*(8)H), 127.6 (Ar*C*(8a)), 128.4 (Ar*C*(6)H), 135.1 (Ar*C*(4a)), 169.8 (*C*=N).

HRMS (ESI$^+$) $C_{12}H_{15}N_2S$ [M]$^+$ found 219.0948, requires 219.0950 (−0.9 ppm).

ν_{max} (film, cm^{-1}) 3003, 2960 (C-H), 2870, 2605 (N-H), 1600, 1589, 1577, 1504 (C=C), 1465, 1446, 1373, 1284, 1257, 756.

(4b*R*,11a*S*)-4b,11a-Dihydro-12*H*-benzo[*d*]indeno[1',2':4,5]imidazo[2,1-*b*]thiazole ((+)-(*R*,*S*)-fused-BTM) (LB3)

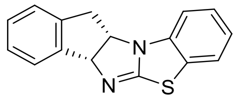

$C_{16}H_{12}N_2S$
264.35

Adapting the procedure reported by Shiina and co-workers,[9] a 25 mL pressure tube was charged with 2-chlorobenzothiazole (0.65 mL, 5.0 mmol, 1.0 eq), (1*R*,2*R*)-*trans*-1-amino-2-indanol (746 mg, 5.0 mmol, 1.0 eq) and *i*-Pr_2NEt (1.3 mL, 7.5 mmol, 1.5 eq), sealed and the mixture stirred at 130 °C for 69 h. The reaction mixture was allowed to cool to room temperature, dissolved in 2.5 mL MeOH and purified directly by silica column chromatography (2% to 4% *i*-PrOH in CH_2Cl_2, R_f 0.25 in 2% *i*-PrOH in CH_2Cl_2) to afford the intermediate 1-(benzo[*d*]thiazol-2-ylamino)-2,3-dihydro-1*H*-inden-2-ol as a beige solid (871 mg, 62%).

^{1}H NMR (500 MHz, CD_3OD) δ_H: 2.89 (1H, dd, *J* 15.7, 6.6, C(3)H^AH^B), 3.27 – 3.34 (1H, m, C(3)H^AH^B), 4.45 (1H, td, *J* 6.8, 5.6, C(2)*H*), 5.22 (1H, d, *J* 5.6, C(1)*H*), 7.06 – 7.12 (1H, m, ArC*H*), 7.19 – 7.31 (4H, m, 4 × ArC*H*), 7.32 – 7.35 (1H, m, ArC*H*), 7.46 (1H, ddd, *J* 8.1, 1.2, 0.6, ArC*H*), 7.61 (1H, ddd, *J* 7.9, 1.2, 0.6, ArC*H*).

The intermediate alcohol (824 mg, 3.12 mmol, 1.0 eq) was suspended in anhydrous CH_2Cl_2 (32 mL). Et_3N (1.7 mL, 12.5 mmol, 4.0 eq) was added and the mixture cooled in an ice bath for 10 min. MsCl (0.31 mL, 4.05 mmol, 1.3 eq) was added dropwise, the reaction mixture warmed to room temperature and stirred until TLC (4% *i*-PrOH in CH_2Cl_2) indicated complete conversion (ca. 3 h; additional 0.1 mL MsCl added to reach full consumption of alcohol). MeOH (0.2 mL) was added and the reaction mixture heated at reflux overnight. After cooling to room temperature, the mixture was washed with H_2O, dried over $MgSO_4$, filtered and the solvent removed under reduced pressure. The crude product was purified by silica column chromatography (2% MeOH in CH_2Cl_2, R_f 0.24), followed by recrystallisation from CH_2Cl_2/hexane to afford (+)-(*R*,*S*)-fused-BTM as light brown needles (479 mg, 58%).

m.p. (CH_2Cl_2/hexane) 162 – 165 °C {Lit.[9] 161 – 163 °C (CH_2Cl_2/hexane)}.

$[\alpha]_D^{20}$ +908 (*c* 1.0 in MeOH) {Lit.[9] $[\alpha]_D^{23}$ +881.8 (*c* 1.0 in MeOH)}.

^{1}H NMR (500 MHz, $CDCl_3$) δ_H: 3.41 (1H, dd, *J* 17.0, 1.9, C(12)H^AH^B), 3.53 (1H, dd, *J* 17.0, 7.0, C(12)H^AH^B), 4.97 (1H, ddd, *J* 8.5, 7.0, 1.9, C(11a)*H*), 6.10 (1H, d, *J* 8.5, C(4b)*H*), 6.82 (1H,

dd, *J* 7.9, 1.6, ArC(10)*H*), 6.96 (1H, td, *J* 7.7, 1.1, Ar(8)*H*), 7.21 (1H, td, *J* 7.7, 1.2, Ar(9)*H*), 7.24 – 7.28 (3H, m, 3 × Ar*H*), 7.28 – 7.33 (1H, m, Ar*H*), 7.54 – 7.57 (1H, m, ArC(7)*H*). Spectroscopic data in accordance with literature.[9]

8 Preparation of vinylcyclopropanes

2-Vinylcyclopropane-1,1-dicarbonitrile (2)

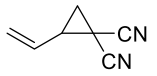

$C_7H_6N_2$
118.14

Following **General Procedure A**, malononitrile (1.98 g, 30.0 mmol, 1.0 eq), 1,4-dibromobut-2-ene (6.42 g, 30.0 mmol, 1.0 eq) and K_2CO_3 (10.4 g, 75.0 mmol, 2.5 eq) in THF (150 mL) at reflux for 24 h gave the title compound after purification by silica column chromatography (petrol : EtOAc 5:1, R_f 0.19) as a colourless oil (2.39 g, 67%).

^{1}H NMR (400 MHz, $CDCl_3$) δ_H: 1.81 (1H, dd, *J* 8.3, 6.2, C(3)H^AH^B), 2.04 (1H, dd, *J* 9.1, 6.2, C(3)H^AH^B), 2.62 – 2.72 (1H, m, C(2)*H*), 5.45 – 5.65 (3H, m, C*H*=CH_2).

Spectroscopic data in accordance with literature.[2]

2-Vinylspiro[cyclopropane-1,2'-indene]-1',3'-dione (S2)

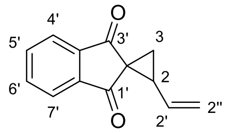

$C_{13}H_{10}O_2$
198.22

Following **General Procedure A**, 1,3-indanedione (731 mg, 5.0 mmol, 1.0 eq), 1,4-dibromobut-2-ene (1.07 g, 5.0 mmol, 1.0 eq) and K_2CO_3 (1.73 g, 12.5 mmol, 2.5 eq) in THF (25 mL) at reflux for 16 h gave the title compound after purification by silica column chromatography (petrol : EtOAc 9:1 to 6:1, R_f 0.33 in petrol : EtOAc 6:1) as a yellow solid (244 mg, 25%). **m.p.** (EtOAc) 126 – 128 °C {Lit.[10] 128 – 130 °C (EtOAc)}.

^{1}H NMR (300 MHz, $CDCl_3$) δ_H: 1.98 (1H, dd, *J* 8.1, 4.0, C(3)H^AH^B), 2.13 (1H, dd, *J* 8.7, 4.0, C(3)H^AH^B), 2.81 (1H, app. q, *J* 8.7, C(2)*H*), 5.14 (1H, dd, *J* 10.3, 1.5, C(2'')H^AH^B), 5.28 (1H, dd, *J* 17.1, 1.5, C(2'')H^AH^B), 6.02 (1H, app. dt, *J* 17.1, 9.9, C(2')*H*), 7.72 – 7.86 (2H, m, ArC(5',6')*H*), 7.88 – 8.00 (2H, m, ArC(4',7')*H*).

Spectroscopic data in accordance with literature.[10]

1-Cyano-2-vinylcyclopropane-1-carboxylic acid, methyl ester (S3)

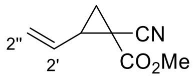

$C_8H_9NO_2$
151.17

Following **General Procedure A**, methyl cyanoacetate (0.88 mL, 10.0 mmol, 1.0 eq), 1,4-dibromobut-2-ene (2.14 g, 10.0 mmol, 1.0 eq) and K_2CO_3 (3.4 g, 25.0 mmol, 2.5 eq) in THF (50 mL) at reflux for 24 h gave the title compound after purification by silica column chromatography (petrol : EtOAc 9:1 to 6:1) as a colourless oil (1.18 g, 78%) as a partially separable mixture of diastereoisomers (2:1 dr).

Data for major diastereoisomer (R_f 0.38 in petrol : EtOAc 6:1)

^{1}H NMR (400 MHz, $CDCl_3$) δ_H: 1.63 (1H, dd, *J* 7.9, 5.1, C(3)H^AH^B), 1.94 (1H, dd, *J* 9.0, 5.1, C(3)H^AH^B), 2.48 – 2.56 (1H, m, C(2)*H*), 3.78 (3H, s, OCH_3), 5.33 (1H, ddd, *J* 10.2, 1.2, 0.6, C(2'')H^AH^B), 5.36 – 5.44 (1H, m, C(2'')H^AH^B), 5.53 – 5.68 (1H, m, C(2')*H*).

Data for minor diastereoisomer (R_f 0.33 in petrol : EtOAc 6:1)

^{1}H NMR (400 MHz, $CDCl_3$) (selected) δ_H: 1.85 – 1.92 (2H, m, C(3)H_2), 2.54 – 2.63 (1H, m, C(2)*H*), 3.76 (3H, s, OCH_3), 5.23 (1H, ddd, *J* 10.3, 1.4, 0.6, C(2'')H^AH^B).

Spectroscopic data in accordance with literature.[2]

Bis(2,2,2-trifluoroethyl) malonate (S4)

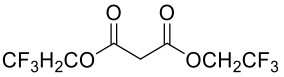

$C_7H_6F_6O_4$
268.11

Following the procedure reported by Waser and co-workers[11], conc. H_2SO_4 (0.47 mL, 8.75 mmol, 0.25 eq) was added to a solution of malonic acid (3.64 g, 35.0 mmol, 1.0 eq) and trifluoroethanol (10.2 mL, 140 mmol, 4.0 eq) in toluene (20 mL) and heated to reflux for 8 h. The reaction mixture was allowed to cool to room temperature and diluted with CH_2Cl_2 (20 mL). The organic phase was washed with 1 M NaOH (1 × 40 mL), H_2O (1 × 40 mL) and brine (1 × 40 mL), dried over $MgSO_4$, filtered and the solvent was removed under reduced pressure to yield the title compound as a colourless oil (1.88 g, 20%), which was used without further purification.

^{1}H NMR (400 MHz, $CDCl_3$) δ_H: 3.61 (2H, s, CH_2), 4.55 (4H, q, *J* 8.2, 2 × OCH_2).

^{19}F{^{1}H} NMR (376 MHz, $CDCl_3$) δ_F: −73.8 (6F, s, 2 × CF_3).

Spectroscopic data in accordance with literature.[12]

2-Vinylcyclopropane-1,1-dicarboxylic acid, bis(2,2,2-trifluoroethyl) ester (S5)

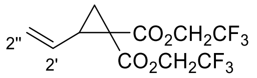

$C_{11}H_{10}F_6O_4$
320.19

Following **General Procedure A**, bis(2,2,2-trifluoroethyl)malonate **S4** (1.8 g, 6.7 mmol, 1.0 eq), 1,4-dibromobut-2-ene (1.44 g, 6.7 mmol, 1.0 eq) and K_2CO_3 (2.32 g, 16.7 mmol, 2.5 eq) in THF (33 mL) at reflux for 24 h gave the title compound after purification by Biotage® Select™ [Sfär 50 g, 120 mL min^{-1}, Et_2O in hexane (2% to 20%, 10 CV), R_f 0.32 in 10% Et_2O in hexane] as a colourless oil (1.71 g, 80%).

^{1}H NMR (400 MHz, $CDCl_3$) δ_H: 1.72 (1H, dd, *J* 9.1, 5.2, C(3)H^AH^B), 1.89 (1H, dd, *J* 8.0, 5.2, C(3)H^AH^B), 2.68 – 2.79 (1H, m, C(2)*H*), 4.41 – 4.65 (4H, m, 2 × CH_2), 5.21 (1H, ddd, *J* 10.0, 1.6, 0.7, C(2'')H^AH^B), 5.34 (1H, ddd, *J* 17.0, 1.6, 0.7, C(2'')H^AH^B), 5.46 (1H, ddd, *J* 17.0, 10.0, 7.8, C(2')*H*).

^{19}F{^{1}H} NMR (376 MHz, $CDCl_3$) δ_F: −74.0 (3F, m, CF_3), −73.8 (3F, m, CF_3).

Spectroscopic data in accordance with literature.[13]

2-Vinylcyclopropane-1,1-dicarboxylic acid, dimethyl ester (S6)

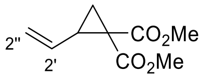

$C_9H_{12}O_4$
184.19

Following **General Procedure A**, dimethyl malonate (1.14 mL, 10.0 mmol, 1.0 eq), 1,4-dibromobut-2-ene (2.14 g, 10.0 mmol, 1.0 eq) and Cs_2CO_3 (8.1 g, 25.0 mmol, 2.5 eq) in THF (50 mL) at reflux for 16 h gave the title compound after purification by Biotage® Isolera™ 4 [SNAP KP-Sil 50 g, 100 mL min^{-1}, petrol : Et_2O (90:10 15 CV), R_f 0.16] as a colourless oil (1.37 g, 74%).

^{1}H NMR (400 MHz, $CDCl_3$) δ_H: 1.59 (1H, dd, *J* 9.0, 5.0, C(3)H^AH^B), 1.72 (1H, dd, *J* 7.6, 4.9, C(3)H^AH^B), 2.58 (1H, app. q, *J* 8.3, C(2)*H*), 3.74 (6H, s, 2 × OCH_3), 5.14 (1H, ddd, *J* 10.0, 1.6, 0.6, C(2'')H^AH^B), 5.29 (1H, ddd, *J* 17.0, 1.7, 0.6, C(2'')H^AH^B), 5.43 (1H, ddd, *J* 17.0, 10.1, 8.2, C(2')*H*).

Spectroscopic data in accordance with literature.[2]

1,1-Bis(phenylsulfonyl)-2-vinylcyclopropane (S7)

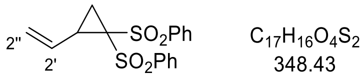

Following **General Procedure A**, bis(phenylsulfonyl)methane (500 mg, 1.69 mmol, 1.0 eq), 1,4-dibromobut-2-ene (361 mg, 1.69 mmol, 1.0 eq) and Cs_2CO_3 (1.38 g, 4.23 mmol, 2.5 eq) in THF (10 mL) at reflux for 16 h gave the title compound after purification by Biotage® Isolera™ 4 [SNAP KP-Sil 25 g, 100 mL min^{-1}, petrol : EtOAc (80:20 15 CV), R_f 0.31] as an off-white solid (403 mg, 68%). **m.p.** (EtOAc) 111 – 113 °C {Lit.[14] 107.5 – 108 °C (EtOH)}.

^{1}H NMR (400 MHz, $CDCl_3$) δ_H: 1.59 (1H, dd, *J* 9.0, 5.0, C(3)*H*^{A}H^B), 1.72 (1H, dd, *J* 7.6, 4.9, C(3)H^A*H*^{B}), 2.58 (1H, app. q, *J* 8.3, C(2)*H*), 3.74 (6H, s, 2 × OC*H*$_3$), 5.14 (1H, ddd, *J* 10.0, 1.6, 0.6, C(2'')*H*^{A}H^B), 5.29 (1H, ddd, *J* 17.0, 1.7, 0.6, C(2'')H^A*H*^{B}), 5.43 (1H, ddd, *J* 17.0, 10.1, 8.2, C(2')*H*).

Spectroscopic data in accordance with literature.[2]

1-Benzylindoline-2,3-dione (S8)

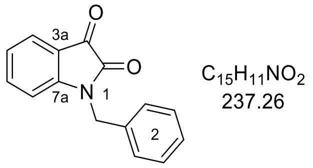

Following the procedure reported by Bower and co-workers[15], to a solution of isatin (1.47 g, 10.0 mmol, 1.0 eq) in MeCN (100 mL) were added K_2CO_3 (4.15 g, 30.0 mmol, 3.0 eq) and benzyl bromide (1.78 mL, 15.0 mmol, 1.5 eq). The mixture was heated at reflux for 18 h, allowed to cool to room temperature and filtered over Celite with EtOAc. The combined organic filtrates were concentrated under reduced pressure and the crude product was recrystallised from toluene (20 mL) to afford the title compound as an orange, crystalline solid (1.9 g, 80%). **m.p.** (toluene) 128 – 131 °C {Lit.[15] 129 – 131 °C (EtOH)}.

^{1}H NMR (500 MHz, $CDCl_3$) δ_H: 4.94 (2H, s, NC*H*$_2$), 6.77 (1H, d, *J* 7.9, ArC(7)*H*), 7.09 (1H, td, *J* 7.6, 0.8, ArC(5)*H*), 7.28 – 7.39 (5H, m, 5 × Ar^2*H*), 7.48 (1H, td, *J* 7.8, 1.4, ArC(6)*H*), 7.62 (1H, dd, *J* 7.4, 1.3, ArC(4)*H*).

Spectroscopic data in accordance with literature.[15]

1-Benzylindolin-2-one (S9)

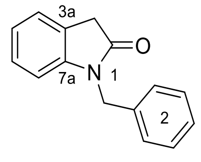

$C_{15}H_{13}NO$
223.28

Adapting a procedure reported by Overman and co-workers[16], a suspension of *N*-benzyl isatin **S8** (428 mg, 1.8 mmol, 1.0 eq) and hydrazine hydrate (65% solution, 5 mL) was heated at reflux for 24 h. The reaction mixture was cooled to room temperature, diluted with H_2O (10 mL) and extracted with EtOAc (3 × 10 mL). The combined organic phases were dried over $MgSO_4$, filtered and the solvent was removed under reduced pressure. The crude product was purified by silica column chromatography (*n*-hexane : EtOAc 3:1, R_f 0.20) to yield the title compound as a viscous, orange oil (372 mg, 92%).

^{1}H NMR (400 MHz, $CDCl_3$) δ_H: 3.65 (2H, s, C(3)H_2), 4.95 (2H, s, NCH_2), 6.75 (1H, d, *J* 7.8, ArC(7)*H*), 7.03 (1H, td, *J* 7.5, 1.0, ArC(5)*H*), 7.15 – 7.23 (1H, m, ArC(6)*H*), 7.24 – 7.38 (6H, m, ArC(4)*H*, 5 × Ar^2*H*).

Spectroscopic data in accordance with literature.[16]

1'-Benzyl-2-vinylspiro[cyclopropane-1,3'-indolin]-2'-one (S10)

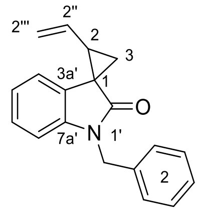

$C_{19}H_{17}NO$
275.35

Following **General Procedure A**, 1-benzylindolin-2-one **S9** (344 mg, 1.54 mmol, 1.0 eq), 1,4-dibromobut-2-ene (330 g, 1.54 mmol, 1.0 eq) and K_2CO_3 (532 g, 3.85 mmol, 2.5 eq) in THF (8 mL) at reflux for 48 h gave the crude product (73:27 dr). Purification by silica column chromatography (5 to 10% Et_2O in *n*-hexane) allowed partial separation of the diastereoisomers to give **371$_{maj}$** (R_f 0.25) as a red solid (>99:1 dr, 103 mg, 24%).

m.p. (Et_2O) 93 – 96 °C {Lit.[17] 97 – 99 °C (EtOAc)}.

^{1}H NMR (400 MHz, $CDCl_3$) δ_H: 1.98 (1H, dd, *J* 8.7, 4.7, C(3)H^AH^B), 2.05 (1H, dd, *J* 7.8, 4.7, C(3)H^AH^B), 2.47 – 2.60 (1H, m, C(2)*H*), 4.95 (1H, d, *J* 15.7, NCH^AH^B), 5.00 (1H, d, *J* 15.7, NCH^AH^B), 5.15 (1H, dd, *J* 10.3, 1.7, C(2''')H^AH^B), 5.27 (1H, dd, *J* 17.2, 1.7, C(2''')H^AH^B), 6.29 (1H, ddd, *J* 17.2, 10.3, 9.4, C(2'')*H*), 6.77 (1H, d, *J* 7.7, ArC(4 or 7)*H*), 6.85 (1H, ddd, *J* 7.4,

1.3, 0.6, ArC(4 or 7)*H*), 7.00 (1H, td, *J* 7.5, 1.0, ArC(5 or 6)*H*), 7.13 (1H, td, *J* 7.7, 1.3, ArC(5 or 6)*H*), 7.22 – 7.37 (5H, m, 5 × Ar^2H).

The minor diastereoisomer was not isolated. Spectroscopic data in accordance with literature.[17]

9 Preparation of Michael acceptors

The following PNP ester starting materials were available in the laboratory, previously prepared according to literature procedures:

S11 was prepared according to Reference [18], **S12**, **S13**, **S14** and **S15** were prepared according to Reference [3], **S16** and **S17** were prepared according to Reference [19].

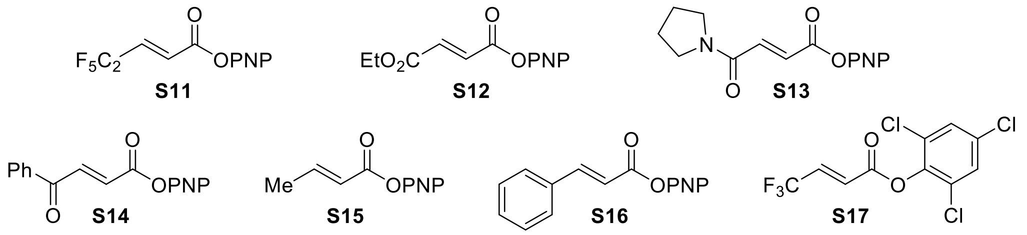

(*E*)-4,4,4-Trifluorobut-2-enoic acid (S18)

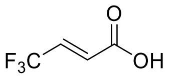

$C_4H_3F_3O_2$
140.06

Following the procedure reported by Smith and co-workers[4], (*E*)-4,4,4-trifluorobut-2-enoic acid, ethyl ester (8.9 mL, 59 mmol, 1.0 eq) was dissolved in THF (100 mL) and NaOH (1 M, 65 mL, 65 mmol, 1.1 eq) was added. The reaction mixture was stirred at room temperature for 4 h and subsequently acidified with HCl (1 M) to pH 2. Excess THF was removed under reduced pressure, the remaining aqueous phase diluted with brine and extracted with Et_2O (3 × 50 mL). The combined organic phases were dried over $MgSO_4$, filtered and the solvent was removed under reduced pressure to yield the title compound as a colourless solid (7.1 g, 86%), which was used without further purification. (Note: product slowly sublimes if left for a prolonged time under reduced pressure)

m.p. (Et_2O) 52 – 55 °C {Lit.[20] 54-55 °C (pentane)}.

^{1}H NMR (400 MHz, $CDCl_3$) δ_H: 6.52 (1H, dq, *J* 15.8, 1.9, C(2)*H*), 6.90 (1H, dq, *J* 15.8, 6.4, C(3)*H*), 12.14 (1H, br s, O*H*).

^{19}F{^{1}H} NMR (376 MHz, $CDCl_3$) δ_F: −66.0 (3F, s, C*F*$_3$)

Spectroscopic data in accordance with literature.[21]

(*E*)-4,4,4-Trifluorobut-2-enoic acid, 4-nitrophenyl ester (3)

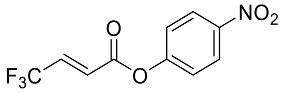

$C_{10}H_6F_3NO_4$
261.16

Following **General Procedure D**, (*E*)-4,4,4-trifluorobut-2-enoic acid **S18** (2.98 g, 21.3 mmol, 1.0 eq), oxalyl chloride (1.89 mL, 22.4 mmol, 1.05 eq) and DMF (3 drops) in anhydrous CH_2Cl_2 (65 mL) followed by 4-nitrophenol (2.96 g, 21.3 mmol, 1.0 eq) and *i*-Pr_2NEt (7.4 mL, 42.6 mmol, 2.0 eq) in anhydrous CH_2Cl_2 (65 mL) gave a brown solid, which was triturated with Et_2O. The colourless solid (*i*-Pr_2NEt·HCl) was filtered and washed with Et_2O. The combined filtrates were concentrated to give a light brown solid, which was recrystallised from hexane (80 mL). The hot solution was decanted from insoluble residues to yield the title compound as an off-white crystalline solid (4.3 g, 77%).

m.p. (hexane) 100 – 103 °C {Lit.[4] 93 – 95 °C (hexane)}.

^{1}H NMR (400 MHz, $CDCl_3$) δ_H: 6.72 (1H, dq, J_{HF} 15.8, 1.9, C(2)*H*), 7.02 (1H, dq, J_{HF} 15.8, 6.4, C(3)*H*), 7.33 – 7.41 (2H, m, Ar(2,6)*H*), 8.26 – 8.35 (2H, m, Ar(3,5)*H*).

^{19}F{^{1}H} NMR (377 MHz, $CDCl_3$) δ_F: −65.7 (3F, s, CF_3).

Spectroscopic data in accordance with literature.[4]

(*E*)-4,4,4-Trifluorobut-2-enoic acid, 3,5-bis(trifluoromethyl)phenyl ester (S19)

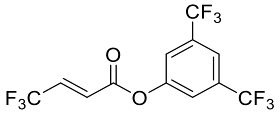

$C_{12}H_5F_9O_2$
352.16

Following **General Procedure D**, (*E*)-4,4,4-trifluorobut-2-enoic acid **S18** (1.1 g, 8.0 mmol, 1.0 eq), oxalyl chloride (0.71 mL, 8.4 mmol, 1.05 eq) and DMF (2 drops) in anhydrous CH_2Cl_2 (24 mL) followed by 3,5-bis(trifluoromethyl)phenol (1.21 mL, 8.0 mmol, 1.0 eq) and *i*-Pr_2NEt (2.7 mL, 16.0 mmol, 2.0 eq) in anhydrous CH_2Cl_2 (24 mL) gave a yellow solid, which was triturated with Et_2O. The colourless solid (*i*-Pr_2NEt·HCl) was filtered, washed with Et_2O and the filtrate was concentrated under reduced pressure. The crude oil was purified by Biotage® Isolera™ 4 [SNAP KP-Sil 50 g, 100 mL min^{-1}, petrol : Et_2O (100:0 2 CV, 100:0 to 90:10 10 CV, 90:10 2 CV), R_f 0.17 in petrol] to afford the title compound as a colourless, volatile oil (2.05 g, 73%).

^{1}H NMR (400 MHz, $CDCl_3$) δ_H: 6.73 (1H, dq, J_{HF} 15.8, 1.9, C(2)*H*), 7.03 (1H, dq, J_{HF} 15.8, 6.3, C(3)*H*), 7.65 – 7.70 (2H, m, Ar(2,6)*H*), 7.80 – 7.85 (1H, m, Ar(4)*H*).

^{19}F{^{1}H} NMR (376 MHz, $CDCl_3$) δ_F: −66.0 (3F, s, C(4)F_3), −63.2 (6F, s, 2 × ArCF_3).
Spectroscopic data in accordance with literature.[4]

(*E*)-4,4,4-Trifluorobut-2-enoic acid, pentafluorophenyl ester (S20)

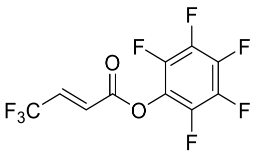

$C_{10}H_2F_8O_2$
306.11

Following **General Procedure D**, (*E*)-4,4,4-trifluorobut-2-enoic acid **S18** (1.1 g, 8.0 mmol, 1.0 eq), oxalyl chloride (0.71 mL, 8.4 mmol, 1.05 eq) and DMF (2 drops) in anhydrous CH_2Cl_2 (24 mL) followed by pentafluorophenol (1.47 g, 8.0 mmol, 1.0 eq) and *i*-Pr_2NEt (2.7 mL, 16.0 mmol, 2.0 eq) in anhydrous CH_2Cl_2 (24 mL) gave a brown solid, which was triturated with Et_2O. The colourless solid (*i*-Pr_2NEt·HCl) was filtered, washed with Et_2O and the combined filtrates were concentrated under reduced pressure. The crude oil was purified by Biotage® Isolera™ 4 [SNAP KP-Sil 50 g, 100 mL min^{-1}, petrol : Et_2O (100:0 5 CV, 100:0 to 95:5 5 CV, 95:5 5 CV), R_f 0.27 in petrol] to afford the title compound as a colourless, volatile oil (1.56 g, 64%).

^{1}H NMR (500 MHz, $CDCl_3$) δ_H: 6.76 (1H, dq, *J* 15.8, 1.9, C(2)*H*), 7.06 (1H, dq, *J* 15.9, 6.3, C(3)*H*).

^{13}C{^{1}H} NMR (126 MHz, $CDCl_3$) δ_C: 121.3 (q, $^{1}J_{CF}$ 270, CF_3), 125.5 (q, $^{3}J_{CF}$ 6.2, *C*(2)H), 124.3 (t, $^{2}J_{CF}$ 14.4, ArC(1)), 135.3 (q, $^{2}J_{CF}$ 36.3, *C*(3)H), 136.8 – 137.1 (m, ArC), 138.7 – 139.2 (m, Ar*C*),), 140.0 (dq, J_{CF} 12.2, 3.9, ArC), 140.9 (tt, J_{CF} 13.6, 3.8, ArC), 142.0 (dq, J_{CF} 12.2, 3.9, ArC), 159.7 (*C*=O).

^{19}F{^{1}H} NMR (376 MHz, $CDCl_3$) δ_F: −161.8 – −161.6 (m, ArC(4)*F*), −156.7 (t, 21.7, ArC(3,5)*F*), −152.4 (d, 16.7, ArC(2,6)*F*), −66.1 (s, CF_3).

HRMS (EI^+) $C_{10}H_2F_8O_2$ $[M]^+$ found 305.9934, requires 305.9927 (+2.3 ppm).

ν_{max} (film, cm^{-1}) 1778 (C=O), 1517, 1300, 1120, 995.

(*E*)-4,4-Difluorobut-2-enoic acid, 2-fluoro-4-nitrophenyl ester (S21)

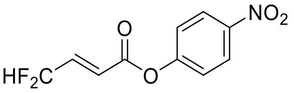

$C_{10}H_7F_2NO_4$
243.17

Following **General Procedure D**, (*E*)-4,4-difluorobut-2-enoic acid (387 mg, 3.17 mmol, 1.0 eq), oxalyl chloride (0.29 mL, 3.33 mmol, 1.05 eq) and DMF (1 drops) in anhydrous CH_2Cl_2 (10 mL) followed by 4-nitrophenol (441 mg, 3.17 mmol, 1.0 eq) and *i*-Pr_2NEt (1.1 mL, 6.3 mmol, 2.0 eq) in anhydrous CH_2Cl_2 (10 mL) gave an orange solid, which was triturated with Et_2O. The colourless solid (*i*-Pr_2NEt·HCl) was filtered, washed with Et_2O and the filtrate concentrated under reduced pressure. The crude solid was purified by silica column chromatography (20% to 25% Et_2O in hexane, R_f 0.29 in 25% Et_2O in hexane) to afford the title compound as a yellow solid (649 mg, 84%). **m.p.** (hexane) 68 – 70 °C.

^{1}H NMR (300 MHz, $CDCl_3$) δ_H: 6.33 (1H, tdd, *J* 54.5, 3.8, 0.8, C(4)*H*) 6.51 (1H, dtd, *J* 15.9, 2.8, 0.8, C(2)*H*), 7.05 (1H, dtd, *J* 15.9, 10.3, 3.8, C(3)*H*), 7.29 – 7.42 (2H, m, Ar(2,6)*H*), 8.24 – 8.39 (2H, m, Ar(3,5)*H*).

^{19}F{^{1}H} NMR (282 MHz, $CDCl_3$) δ_F: −116.9 (s, C(4)F_2).

HRMS (EI^+) $C_{10}H_7F_2NO_4$ $[M]^+$ found 243.0333, requires 243.0337 (−0.4 ppm).

ν_{max} (film, cm^{-1}) 3118, 3082, 1735 (C=O), 1699, 1678, 1620, 1591, 1531 (NO_2), 1487, 1390, 1344 (NO_2), 1307, 1257, 1197, 1172, 1037, 1012, 979, 964, 952.

Spectroscopic data in accordance with literature.[22]

Diethyl (1,1-difluoro-2-oxoethyl)phosphonate (S22)

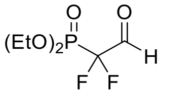

$C_6H_{11}F_2O_4P$
216.12

Note: strict exclusion of moisture must be assured. Once $CeCl_3$·$7H_2O$ has been dehydrated it must not be exposed to air at any point.

Adapting the procedure from Pajkert and Röschenthaler,[23] $CeCl_3$·$7H_2O$ (2.93 g, 7.8 mmol, 1.05 eq) was placed into a flame-dried 2-necked round bottom flask, heated to 200 °C at 0.1 mbar over to 2 h and kept at this temperature for 18 h. After cooling to room temperature, anhydrous THF (dried over CaH_2, 0.33 M, 23 mL) was added under N_2 to the dried $CeCl_3$ and the suspension cooled to −78 °C. LDA (1.4 M solution in THF, 5.65 mL, 8.25 mmol, 1.1 eq) was added dropwise and the suspension was stirred vigorously for 20 min, followed by the dropwise addition of diethyl (difluoromethyl)phosphonate (1.18 mL, 7.5 mmol, 1.0 eq). After 1 h at −78 °C, anhydrous DMF (0.64 mL, 8.25 mmol, 1.1 eq) was added, the reaction warmed to room temperature and quenched by the addition of 3 M HCl (15 mL). After the complete dissolution of all cerium salts (ca. 30 min),

the phases were separated and the aqueous phase was extracted with CH_2Cl_2 (3 × 20 mL). The combined organic phases were washed with brine (1 × 30 mL), dried over $MgSO_4$, filtered and the solvent was removed under reduced pressure. The crude residue was purified by distillation over P_2O_5 to yield the title compound as a colourless oil (835 mg, 51%). **b.p.** 45 – 47 °C (0.1 mbar) {Lit.[23] 74 – 77 °C (0.1 mmHg)}

^{1}H NMR (400 MHz, $CDCl_3$) δ_H: 1.40 (6H, td, *J* 7.1, 0.7, 2 × OCH_2CH_3), 4.33 (4H, dq, *J* 8.2, 7.1, 2 × OCH_2CH_3), 9.60 (1H, app. q, *J* 3.4, C*H*O).

^{19}F{^{1}H} NMR (377 MHz, $CDCl_3$) δ_F: −123.5 (d, $^2J_{FP}$ 96.6, C*F*$_2$).

^{31}P{^{1}H} NMR (162 MHz, $CDCl_3$) δ_P: 2.5 (t, $^2J_{PF}$ 96.6, *P*(O)(OEt)$_2$).

Spectroscopic data in accordance with literature.[23]

(*E*)-4-(Diethoxyphosphoryl)-4,4-difluorobut-2-enoic acid, *t*-butyl ester (S23)

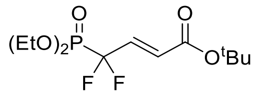

$C_{12}H_{21}F_2O_5P$
314.27

t-Butyl diethylphosphonoacetate (0.46 mL, 1.97 mmol, 1.1 eq), LiCl (84 mg, 1.97 mmol, 1.1 eq) and *i*-Pr_2NEt (0.34 mL, 1.97 mmol, 1.1 eq) were stirred in anhydrous MeCN (6.5 mL) for 30 min at room temperature. A solution of aldehyde **S22*** (386 mg, 1.79 mmol, 1.0 eq) in anhydrous MeCN (6.5 mL) was added slowly and the reaction mixture allowed to stir overnight. The resulting white suspension was diluted with H_2O (10 mL) and extracted with EtOAc (3 × 10 mL). The combined organic phases were washed with brine, dried over $MgSO_4$, filtered and concentrated under reduced pressure. The crude product was purified by silica column chromatography (5% Et_2O in CH_2Cl_2, R_f 0.45) to afford the title compound as a colourless oil (484 mg, 86%).

^{1}H NMR (500 MHz, $CDCl_3$) δ_H: 1.37 (6H, td, *J* 7.1, 0.7, 2 × OCH_2CH_3), 1.48 (9H, s, C(C*H*$_3$)$_3$), 4.23 – 4.32 (4H, m, 2 × OCH_2CH_3), 6.32 (1H, app. dq, *J* 15.8, 2.7, C(2)*H*), 6.77 (1H, dtd, *J* 15.8, 12.8, 2.2, C(3)*H*).

^{13}C{^{1}H} NMR (126 MHz, $CDCl_3$) δ_C: 16.3 (d, $^3J_{CP}$ 5.4, 2 × OCH_2CH_3) 27.9 (C(CH_3)$_3$), 65.0 (d, $^2J_{CP}$ 6.6, 2 × OCH_2CH_3), 81.9 (*C*(CH_3)$_3$), 116.3 (td, $^1J_{CF}$ 260, $^1J_{CP}$ 217, *C*(4)), 129.8 (td, $^3J_{CF}$ 9.4, $^3J_{CP}$ 5.5, *C*(2)H), 134.1 (td, $^2J_{CF}$ 21.9, $^2J_{CP}$ 13.0, *C*(3)H), 163.5 (*C*=O).

^{19}F{^{1}H} NMR (376 MHz, $CDCl_3$) δ_F: −111.2 (d, $^2J_{FP}$ 108, C(4)*F*$_2$).

^{31}P{^{1}H} NMR (162 MHz, $CDCl_3$) δ_P: 5.1 (t, $^2J_{PF}$ 108, C(4)*P*(O)(OEt)$_2$).

HRMS (ESI$^+$) $C_{12}H_{21}F_2O_5PNa$ $[M+Na]^+$ found 337.0973, requires 337.0987 (−4.1 ppm).

ν_{max} (film, cm^{-1}) 2981, 2935 (C-H), 2875, 1718 (C=O), 1664, 1654, 1479, 1394 ($C(CH_3)_3$), 1369 ($C(CH_3)_3$), 1319, 1273 (P=O), 1153, 1101, 1012, 970.

* *Alternatively, the corresponding crude acetal prior to distillation over P_2O_5 can also be used.*

(*E*)-4-(Diethoxyphosphoryl)-4,4-difluorobut-2-enoic acid (S24)

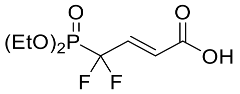

$C_8H_{13}F_2O_5P$
258.16

Following **General Procedure C**, ester **S23** (454 mg, 1.44 mmol, 1.0 eq) in TFA : CH_2Cl_2 1:2 (2.1 mL) after 16 h gave the title compound as an off-white solid (358 mg, 96%), which was used without further purification. **m.p.** (MeOH) 65 – 68 °C.

^{1}H NMR (500 MHz, $CDCl_3$) δ_H: 1.39 (6H, t, *J* 7.1, 2 × OCH_2CH_3), 4.23 – 4.38 (4H, m, 2 × OCH_2CH_3), 6.40 (1H, app. dq, *J* 15.8, 2.6, C(2)*H*), 6.94 (1H, dtd, *J* 15.8, 12.6, 1.7, C(3)*H*), 9.63 (1H, s, O*H*).

^{13}C{^{1}H} NMR (126 MHz, $CDCl_3$) δ_C: 16.3 (d, $^3J_{CP}$ 5.4, 2 × OCH_2CH_3), 65.5 (d, $^2J_{CP}$ 6.8, 2 × OCH_2CH_3), 116.0 (td, $^1J_{CF}$ 260, $^1J_{CP}$ 217, *C*(4)), 127.7 (td, $^3J_{CF}$ 9.4, $^3J_{CP}$ 5.6, *C*(2)H), 136.5 (td, $^2J_{CF}$ 21.9, $^2J_{CP}$ 13.1, *C*(3)H), 167.7 (*C*=O).

^{19}F{^{1}H} NMR (376 MHz, $CDCl_3$) δ_F: −112.2 (d, $^2J_{FP}$ 108, C(4)F_2).

^{31}P{^{1}H} NMR (202 MHz, $CDCl_3$) δ_P: 4.9 (t, $^2J_{PF}$ 108, C(4)*P*(O)(OEt)$_2$).

HRMS (ESI$^+$) $C_8H_{13}F_2O_5PNa$ [M+Na]$^+$ found 281.0351, requires 281.0361 (−3.5 ppm).

ν_{max} (film, cm^{-1}) 2991 (O-H), 2659, 2559, 1720 (C=O), 1668, 1481, 1446, 1390, 1251 (P=O), 1174, 1056, 1022 (P-OEt), 989, 966.

(*E*)-4-(Diethoxyphosphoryl)-4,4-difluorobut-2-enoic acid, 4-nitrophenol ester (S25)

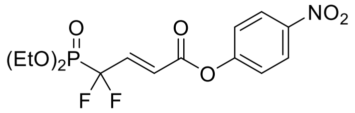

$C_{14}H_{16}F_2NO_7P$
379.25

Following **General Procedure D**, acid **S24** (430 mg, 1.66 mmol, 1.0 eq), oxalyl chloride (0.15 mL, 1.75 mmol, 1.05 eq) and DMF (1 drop) in anhydrous CH_2Cl_2 (5.0 mL) followed by 4-nitrophenol (232 mg, 1.66 mmol, 1.0 eq) and *i*-Pr_2NEt (0.57 mL, 3.33 mmol, 2.0 eq) in anhydrous CH_2Cl_2 (5.0 mL) gave an orange solid, which was triturated with Et_2O. The colourless solid (*i*-Pr_2NEt·HCl) was filtered, washed with Et_2O and the filtrate was concentrated under reduced pressure. The resulting crude oil was purified by silica column chromatography (5% Et_2O in CH_2Cl_2, R_f 0.32) to afford the title compound as a colourless oil (572 mg, 90%).

^{1}H NMR (500 MHz, $CDCl_3$) δ_H: 1.42 (6H, t, *J* 7.1, 2 × OCH_2CH_3), 4.27 – 4.39 (4H, m, 2 × OCH_2CH_3), 6.63 (1H, app. dq, *J* 15.9, 2.6, C(2)*H*), 7.13 (1H, dtd, *J* 15.9, 12.4, 2.1, C(3)*H*), 7.32 – 7.39 (2H, m, Ar(2,6)*H*), 8.27 – 8.34 (2H, m, Ar(3,5)*H*).

^{13}C{^{1}H} NMR (126 MHz, $CDCl_3$) δ_C: 16.4 (d, $^3J_{CP}$ 5.3, 2 × OCH_2CH_3), 65.3 (d, $^2J_{CP}$ 6.9, 2 × OCH_2CH_3), 115.9 (td, $^1J_{CF}$ 260, $^1J_{CP}$ 217, *C*(4)), 122.3 (Ar*C*(2,6)H), 125.3 (Ar*C*(3,5)H), 126.4 (td, $^3J_{CF}$ 9.6, $^3J_{CP}$ 5.4, *C*(2)H), 138.4 (td, $^2J_{CF}$ 22.2, $^2J_{CP}$ 13.1, *C*(3)H), 145.6 (Ar*C*(4)), 154.8 (Ar*C*(1)), 161.8 (*C*=O).

^{19}F{^{1}H} NMR (376 MHz, $CDCl_3$) δ_F: −112.0 (d, $^2J_{FP}$ 106, C(4)F_2).

^{31}P{^{1}H} NMR (202 MHz, $CDCl_3$) δ_P: 4.5 (t, $^2J_{PF}$ 106, C(4)*P*(O)(OEt)$_2$).

HRMS (ESI$^+$) $C_{14}H_{16}F_2NO_7PNa$ [M+Na]$^+$ found 402.0518, requires 402.0525 (−1.6 ppm).

ν_{max} (film, cm^{-1}) 3116, 3084, 2987, 2870, 1749 (C=O), 1662, 1616 (C=C), 1593, 1523 (NO_2), 1490 (C=C_{Ar}), 1346 (NO_2), 1271 (P=O), 1207, 1182, 1161, 1143, 1097, 1008, 968, 948.

***t*-Butyl ethyl fumarate (S26)**

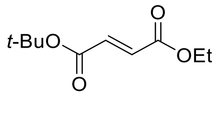

$C_{10}H_{16}O_4$
200.23

Adapting the procedure reported by Smith and co-workers[3], di-*t*-butyl dicarbonate (5.4 g, 25.0 mmol, 1.25 eq) and DMAP (489 mg, 4.0 mmol, 0.2 eq) were added to a stirred solution of monoethyl fumarate (2.8 g, 20.0 mmol, 1.0 eq) in THF (30 mL) at 0 °C. The reaction mixture was stirred at room temperature for 16 h, diluted with EtOAc (30 mL) and

washed sequentially with 10% sulfuric acid (50 mL), 1 M NaOH (50 mL) and brine (50 mL). The organic phase was dried over $MgSO_4$, filtered and the solvent was removed under reduced pressure. The crude product was purified by silica column chromatography (petrol : EtOAc 5:1, R_f 0.61) to afford the title compound as a colourless oil (1.9 g, 48%).

^{1}H NMR (400 MHz, $CDCl_3$) δ_H: 1.31 (3H, t, *J* 7.1, OCH_2CH_3), 1.50 (9H, s, $C(CH_3)_3$), 4.24 (2H, q, *J* 7.1, OCH_2CH_3), 6.73 (1H, d, *J* 15.7, C(2)*H* or C(3)*H*), 6.77 (1H, d, *J* 15.7, C(2)*H* or C(3)*H*).

Spectroscopic data in accordance with literature.[24]

(*E*)-4-(*t*-Butoxy)-4-oxobut-2-enoic acid (S27)

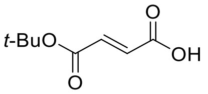

$C_8H_{12}O_4$
172.18

Following **General Procedure B**, *t*-butyl ethyl fumarate **S26** (1.9 g, 9.5 mmol, 1.0 eq) and $LiOH{\cdot}H_2O$ (438 mg, 10.4 mmol, 1.1 eq) in H_2O : THF 1:1 (10 mL) after 6 h gave the title compound as a white solid (1.49 g, 86%), which was used without further purification.

m.p. (CH_2Cl_2) 61 – 64 °C {Lit.[25] 65 – 68 °C(EtOAc)}.

^{1}H NMR (400 MHz, $CDCl_3$) δ_H: 1.51 (9H, s, $C(CH_3)_3$), 6.74 (1H, d, *J* 15.7, C(2)*H* or C(3)*H*), 6.86 (1H, d, *J* 15.7, C(2)*H* or C(3)*H*).

Spectroscopic data in accordance with literature.[25]

***t*-Butyl (4-nitrophenyl) fumarate (S28)**

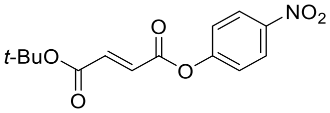

$C_{14}H_{15}NO_6$
293.09

Following **General Procedure D**, acid **S27** (1.2 g, 7.0 mmol, 1.0 eq), oxalyl chloride (0.62 mL, 7.3 mmol, 1.05 eq) and DMF (1 drop) in anhydrous CH_2Cl_2 (21 mL) followed by 4-nitrophenol (974 mg, 7.0 mmol, 1.0 eq) and *i*-Pr_2NEt (2.4 mL, 14.0 mmol, 2.0 eq) in anhydrous CH_2Cl_2 (21 mL) gave a brown solid, which was triturated with Et_2O. The colourless solid (*i*-$Pr_2NEt{\cdot}HCl$) was filtered, washed with Et_2O and the filtrate was concentrated under reduced pressure. The crude solid was recrystallised from hexane (30 mL) and the hot solution decanted to separate from insoluble residue to afford the title compound as an off-white, crystalline solid (1.56 g, 76%). **m.p.** (hexane) 72 – 74 °C.

^{1}**H NMR** (400 MHz, $CDCl_3$) δ_H: 1.54 (9H, s, C(C*H*$_3$)$_3$), 6.94 (1H, d, *J* 15.8, C(2)*H* or C(3)*H*), 7.01 (1H, d, *J* 15.7, C(2)*H* or C(3)*H*), 7.31 – 7.38 (2H, m, Ar(2,6)*H*), 8.27 – 8.33 (2H, m, Ar(3,5)*H*).

Spectroscopic data in accordance with literature.[26]

(*E*)-4-(Methoxymethylamino)-4-oxo-2-butenoic acid (S29)

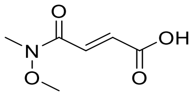

$C_6H_9NO_4$
159.14

Following the procedure reported by Jacobi and co-workers,[27] a stirred solution of maleic anhydride (5.07 g, 51.7 mmol, 1.0 eq) and (*N*,*O*)-dimethylhydroxylamine hydrochloride (5.55 g, 56.8 mmol, 1.1 eq) in $CHCl_3$ (60 mL) was cooled to 0 °C. Pyridine (9.2 mL, 113 mmol, 2.2 eq) was added slowly and the reaction was allowed to warm to room temperature and stir for 24 h. The solvent was removed under reduced pressure, the residue was diluted with H_2O (20 mL) and brine (20 mL) and extracted with CH_2Cl_2 (4 × 30 mL). The combined extracts were washed with 1 M HCl (40 mL) and brine (40 mL), dried over $MgSO_4$, filtered and the solvent was removed under reduced pressure. The crude product was recrystallised from CH_2Cl_2 to afford the title compound as a yellow solid (2.74 g, 33%). **m.p.** (CH_2Cl_2) 129 – 131 °C.

^{1}**H NMR** (500 MHz, $CDCl_3$) δ_H: 3.31 (3H, s, NC*H*$_3$), 3.76 (3H, s, OC*H*$_3$), 6.91 (1H, d, *J* 15.6, C(2)*H*), 7.54 (1H, d, *J* 15.6, C(3)*H*).

Spectroscopic data in accordance with literature.[28]

(*E*)-4-(Methoxy(methyl)amino)-4-oxobut-2-enoic acid, 4-nitrophenol ester (S30)

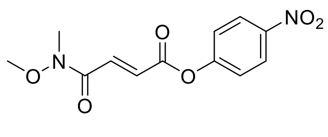

$C_{12}H_{12}N_2O_6$
280.07

Following **General Procedure D**, acid **S29** (1.35 g, 8.4 mmol, 1.0 eq), oxalyl chloride (0.75 mL, 8.9 mmol, 1.05 eq) and DMF (3 drops) in anhydrous CH_2Cl_2 (25 mL) followed by 4-nitrophenol (1.17 g, 8.4 mmol, 1.0 eq) and *i*-Pr_2NEt (2.9 mL, 16.8 mmol, 2.0 eq) in anhydrous CH_2Cl_2 (25 mL) gave a brown solid, which was triturated with Et_2O. The colourless solid (*i*-$Pr_2NEt{\cdot}HCl$) was filtered, washed with Et_2O and the filtrate was concentrated under reduced pressure. The crude solid was purified by silica column

chromatography (petrol : EtOAc 1:1, R_f 0.24) to afford the title compound as a white solid (674 mg, 29%). **m.p.** (EtOAc) 93 – 95 °C.

^{1}H NMR (500 MHz, $CDCl_3$) δ_H: 3.33 (3H, s, NC*H*3), 3.78 (3H, s, OC*H*3), 7.09 (1H, d, *J* 15.5, C(2)*H* or C(3)*H*), 7.34 – 7.38 (2H, m, Ar(2,6)*H*), 7.67 (1H, d, *J* 15.5, C(2)*H* or C(3)*H*), 8.28 – 8.32 (2H, m, Ar(3,5)*H*).

^{13}C{^{1}H} NMR (126 MHz, $CDCl_3$) δ_C: 32.4 (NCH_3), 62.4 (OCH_3), 122.3 (Ar*C*(2,6)H), 125.3 (Ar*C*(3,5)H), 130.5 (*C*(2)H or *C*(3)H), 134.6 (*C*(2)H or *C*(3)H), 145.5 (Ar*C*(4)), 155.0 (Ar*C*(1)), 163.1 (*C*(1)=O), 163.9 (*C*(4)=O).

HRMS (ESI$^+$) $C_{12}H_{13}N_2O_6$ $[M+H]^+$ found 281.0764, requires 281.0768 (−1.4 ppm).

ν_{max} (film, cm^{-1}) 3115, 2953 (C-H), 1741 (C=O_{ester}), 1660 (C=O_{amide}), 1635 (C=C), 1631, 1614, 1591 (C=C_{Ar}), 1517 (NO_2), 1469, 1390, 1346 (NO_2), 1294, 1261, 1211, 1103.

(*E*)-4-(Allyl(methyl)amino)-4-oxobut-2-enoic acid, ethyl ester (S31)

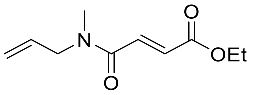

$C_{10}H_{15}NO_3$
197.23

Adapting the procedure reported by Snaddon and co-workers[29], monoethyl fumarate (1.0 g, 7.0 mmol, 1.0 eq) and methyl allyl amine (0.74 mL, 7.7 mmol, 1.1 eq) were dissolved in CH_2Cl_2 (20 mL), followed by the addition of EDCI·HCl (1.48 g, 7.7 mmol, 1.1 eq) and DMAP (85 g, 0.7 mmol, 0.1 eq) at 0 °C. The reaction mixture was allowed to warm to room temperature overnight and subsequently washed with 1 M HCl (2 × 20 mL) and brine (2 × 20 mL). The organic phase was dried over $MgSO_4$, filtered and the solvent was removed under reduced pressure to afford the crude product as a dark red oil. Filtration over a short plug of silica with petrol : EtOAc 3:1 afforded a rotameric mixture (4:3) of the title compound as a pale yellow oil (1.1 g, 82%), which was used without further purification.

^{1}H NMR (400 MHz, $CDCl_3$) *major rotamer* δ_H: 1.27 – 1.34 (3H, m, OCH2C*H*3), 3.01 (3H, s, NC*H*3), 4.00 (2H, dt, *J* 4.9, 1.8, NC*H*2), 4.20 – 4.29 (2H, m, OC*H*2CH3), 5.14 – 5.28 (2H, m, CH=C*H*2), 5.71 – 5.85 (1H, m, C*H*=CH2), 6.79 (1H, d, *J* 15.3, C(2)*H* or C(3)*H*), 7.29 (1H, d, *J* 15.3, C(2)*H* or C(3)*H*).

minor rotamer (selected) δ_H: 3.06 (3H, s, NC*H*3), 4.07 (2H, dt, *J* 6.0, 1.5, NC*H*2), 6.80 (1H, d, *J* 15.3, C(2)*H* or C(3)*H*), 7.40 (1H, d, *J* 15.3, C(2)*H* or C(3)*H*).

^{13}C{^{1}H} NMR (126 MHz, $CDCl_3$) *major rotamer* δ_C: 14.1 (OCH_2CH_3), 34.0 (NCH_3), 52.3 (NCH_2), 61.1 (OCH_2CH_3), 117.3 ($CH=CH_2$), 131.3 (*C*(2)H or *C*(3)H), 132.1 (*C*H=CH2), 133.7

(*C*(2)H or *C*(3)H), 165.1 (*C*(4)=O), 165.7 (*C*(1)=O). *minor rotamer (selected)* δ_C: 34.9 ((NCH$_3$), 50.4 (NCH$_2$), 117.9 (CH=CH$_2$), 131.5 (*C*(2)H or *C*(3)H), 164.4 (*C*(4)=O).

HRMS (ESI$^+$) $C_{10}H_{15}NO_3Na$ [M+Na]$^+$ found 220.0941, requires 220.0944 (−1.3 ppm).

ν$_{max}$ (film, cm^{-1}) 3080 (=C-H), 2983 (C-H), 2877, 1720 (C=O$_{ester}$), 1653 (C=O$_{amide}$), 1625 (C=C), 1402, 1284, 1176, 1031, 974.

(*E*)-4-(Allyl(methyl)amino)-4-oxobut-2-enoic acid (S32)

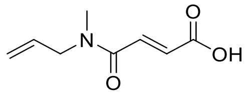

$C_8H_{11}NO_3$
169.18

Following **General Procedure B**, ethyl ester **S31** (1.1 g, 5.7 mmol, 1.0 eq) and LiOH·H$_2$O (264 mg, 6.3 mmol, 1.1 eq) in H$_2$O : THF 1:1 (6 mL) after 8 h gave a rotameric mixture (4:3) of the title compound as an off-white solid (722 mg, 75%), which was used without further purification.

^{1}H NMR (500 MHz, CDCl$_3$) *major rotamer* δ_H: 3.04 (3H, s, NC*H*$_3$), 3.98 – 4.03 (2H, m, NC*H*$_2$), 5.15 – 5.30 (2H, m, CH=C*H*$_2$), 5.71 – 5.85 (1H, m, C*H*=CH$_2$), 6.80 (1H, d, *J* 15.3, C(2)*H* or C(3)*H*), 7.35 (1H, d, *J* 15.3, C(2)*H* or C(3)*H*), 11.02 (1H, br s, O*H*). *minor rotamer (selected)* δ_H: 3.07 (3H, s, NC*H*$_3$), 4.08 (2H, d, *J* 6.0, NC*H*$_2$), 6.81 (1H, d, *J* 15.3, C(2)*H* or C(3)*H*), 7.46 (1H, d, *J* 15.3, C(2)*H* or C(3)*H*).

^{13}C{^{1}H} NMR (126 MHz, CDCl$_3$) *major rotamer* δ_C: 34.2 (NCH$_3$), 52.5 (NCH$_2$), 117.5 (CH=CH$_2$), 130.7 (*C*(2)H or *C*(3)H), 131.9 (CH=CH$_2$), 135.3 (*C*(2)H or *C*(3)H)), 165.1 (*C*(4)=O), 169.6 (*C*(1)=O).

minor rotamer (selected) δ_C: 35.0 (NCH$_3$), 50.5 (NCH$_2$), 118.25 (CH=CH$_2$), 130.9 (*C*(2)H or *C*(3)H), 164.5 (*C*(4)=O), 169.6 (*C*(1)=O).

HRMS (ESI$^-$) $C_8H_{10}NO_3$ [M−H]$^-$ found 168.0660, requires 168.0666 (−3.6 ppm).

ν$_{max}$ (film, cm^{-1}) 2933 (O-H), 2756 (NC-H), 1712 (C=O$_{ester}$), 1658 (C=O$_{amide}$), 1600 (C=C), 1408, 1168.

(*E*)-4-(Allyl(methyl)amino)-4-oxobut-2-enoic acid, 4-nitrophenol ester (S33)

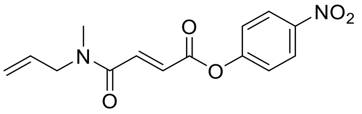

$C_{14}H_{14}N_2O_5$
290.28

Adapting the procedure reported by Smith and co-workers[30], acid **S32** (677 mg, 4.0 mmol, 1.0 eq) and EDCI·HCl (997 mg, 5.2 mmol, 1.3 eq) were dissolved in anhydrous CH_2Cl_2 (7 mL) and stirred for 10 min at room temperature. 4-Nitrophenol (835 mg, 6.0 mmol, 1.5 eq) was added, the reaction mixture stirred for 16 h and quenched by the addition of H_2O (7 mL). The phases were separated and the aqueous phase was extracted with CH_2Cl_2 (3 × 10 mL). The combined organic phases were dried over $MgSO_4$, filtered and the solvent was removed under reduced pressure. The crude product was purified by silica column chromatography (5 to 10% Et_2O in CH_2Cl_2, R_f 0.22 in 5% Et_2O in CH_2Cl_2) to afford a rotameric mixture (4:3) of the title compound as a colourless oil (583 mg, 50%).

^{1}H NMR (500 MHz, $CDCl_3$) *major rotamer* δ_H: 3.04 (3H, s, NC*H*$_3$), 4.03 (2H, dt, *J* 4.8, 1.8, NC*H*$_2$), 5.15 – 5.30 (2H, m, CH=C*H*$_2$), 5.71 – 5.87 (1H, m, C*H*=CH$_2$), 6.97 (1H, d, *J* 15.3, C(3)*H*), 7.29 – 7.36 (2H, m, Ar(2,6)*H*), 7.48 (1H, d, *J* 15.3, C(2)*H*), 8.23 – 8.30 (2H, m, Ar(3,5)*H*). *minor rotamer (selected)* δ_H: 3.09 (3H, s, NC*H*$_3$), 4.09 (2H, dt, *J* 6.0, 1.5, NC*H*$_2$), 6.98 (1H, d, *J* 15.3, C(3)*H*), 7.61 (1H, d, *J* 15.3, C(2)*H*).

^{13}C{^{1}H} NMR (126 MHz, $CDCl_3$) *major rotamer* δ_C: 34.2 (NCH$_3$), 52.4 (NCH$_2$), 117.4 (CH=CH$_2$), 122.3 (Ar*C*(2,6)H), 125.2 (Ar*C*(3,5)H), 129.4 (*C*(3)H), 132.0 (CH=CH$_2$), 136.8 (*C*(2)H), 145.5 (Ar*C*(4)), 155 (Ar*C*(1)), 163.2 (*C*(1)=O), 164.3 (*C*(4)=O). *minor rotamer (selected)* δ_C: 35.0 (NCH$_3$), 50.4 (NCH$_2$), 118.2 (CH=CH$_2$), 125.3 (Ar*C*(3,5)H), 129.5 (*C*(3)H), 131.8 (CH=CH$_2$), 136.8 (*C*(2)H), 163.6 (*C*(4)=O).

HRMS (ESI$^+$) $C_{14}H_{15}N_2O_5$ [M+H]$^+$ found 291.0973, requires 291.0975 (−0.8 ppm).

ν_{max} (film, cm^{-1}) 3082 (=CH$_2$), 2931 (C-H), 1741 (C=O$_{ester}$), 1654 (C=O$_{amide}$), 1616 (C=C), 1591(C=C$_{Ar}$), 1519 (C-NO$_2$), 1489 (C=C$_{Ar}$), 1344 (C-NO$_2$), 1199, 1124.

(*E*)-3-(2,5-Dioxopyrrolidin-1-yl)prop-2-enoic acid, *t*-butyl ester (S34)

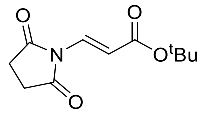

$C_{11}H_{15}NO_4$
225.24

Adapting the procedure of Mola et. al[31], succinimide (495 mg, 5.0 mmol, 1.0 eq) and DABCO (112 mg, 1.0 mmol, 0.2 eq) were dissolved in MeCN (50 mL). *t*-Butyl propiolate

(0.82 mL, 6.0 mmol, 1.2 eq) was added dropwise and the reaction mixture stirred at room temperature until TLC (petrol : EtOAc 4:1) indicated full conversion (ca. 2 h). The solvent was removed under reduced pressure and the crude product was purified by silica column chromatography (2% Et_2O in CH_2Cl_2, R_f 0.26) to afford the title compound as a pale yellow solid (987 mg, 87%). **m.p.** (CH_2Cl_2) 99 – 102 °C.

^{1}H NMR (500 MHz, $CDCl_3$) δ_H: 1.49 (9H, s, C(CH_3)$_3$), 2.81 (4H, s, 2 × CH_2), 6.89 (1H, d, *J* 14.8, C(2)*H*), 7.64 (1H, d, *J* 14.8, C(3)*H*).

^{13}C{^{1}H} NMR (126 MHz, $CDCl_3$) δ_C: 27.7 (2 x CH_2), 28.1 (C(CH_3)$_3$), 81.0 (*C*(CH_3)$_3$), 112.8 (*C*(2)H), 130.0 (*C*(3)H), 165.9 (*C*(1)=O), 174.4 (2 × *C*=O_{pyrr}).

HRMS (ESI$^+$) $C_{11}H_{15}NO_4Na$ [M+Na]$^+$ found 248.0888, requires 248.0893 (−2.0 ppm).

ν_{max} (film, cm^{-1}) 3107, 3076, 2980, 2933 (C-H), 1789 (C=O_{imide}), 1714 (C=O_{ester}), 1693, 1639 (C=C), 1477, 1454, 1369, 1290, 1255, 1151, 1107, 987.

(*E*)-3-(2,5-Dioxopyrrolidin-1-yl)prop-2-enoic acid (S35)

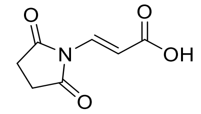

$C_7H_7NO_4$
169.14

Following **General Procedure C**, ester **S34** (922 mg, 4.0 mmol, 1.0 eq) in TFA : CH_2Cl_2 1:2 (5 mL) after 4 h gave the title compound as a pale yellow solid (683 mg, 98%), which was used without further purification. **m.p.** (CH_2Cl_2) 224 – 226 °C.

^{1}H NMR (500 MHz, DMSO-*d$_6$*) δ_H: 2.72 (4H, s, 2 × CH_2), 6.71 (1H, d, *J* 14.8, C(2)*H*), 7.50 (1H, d, *J* 14.8, C(3)*H*), 12.56 (1H, br s, O*H*).

^{13}C{^{1}H} NMR (126 MHz, DMSO-*d$_6$*) δ_C: 28.4 (2 × CH_2), 109.3 (*C*(2)H), 131.6 (*C*(3)H), 168.1 (*C*(1)=O), 176.5 (2 × N*C*=O).

HRMS (ESI$^-$) $C_7H_6NO_4$ [M−H]$^-$ found 168.0296, requires 168.0302 (−3.9 ppm).

ν_{max} (film, cm^{-1}) 3111, 2954 (O-H), 2650, 2590, 2474, 1772 (C=O_{imide}), 1732 (C=O_{acid}), 1678, 1597, 1429, 1365, 1234, 1161, 1111, 985 (=C-H), 898, 779.

(*E*)-3-(2,5-Dioxopyrrolidin-1-yl)prop-2-enoic acid, 4-nitrophenol ester (S36)

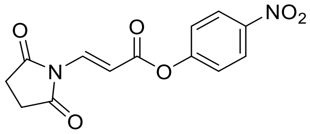

$C_{13}H_{10}N_2O_6$
290.23

Following **General Procedure D**, acid **S35** (665 mg, 3.9 mmol, 1.0 eq), oxalyl chloride (0.35 mL, 4.1 mmol, 1.05 eq) and DMF (1 drop) in anhydrous CH_2Cl_2 (12 mL) followed by 4-nitrophenol (542 mg, 3.9 mmol, 1.0 eq) and *i*-Pr_2NEt (1.3 mL, 7.8 mmol, 2.0 eq) in anhydrous CH_2Cl_2 (12 mL) gave a brown solid, which was purified by silica column chromatography (5% Et_2O in CH_2Cl_2, R_f 0.43) to afford an off-white solid. Trituration with Et_2O removed remaining 4-nitrophenol impurities to afford the title compound as a white crystallin solid (860 mg, 76%). **m.p.** (Et_2O) 184 – 187 °C.

^{1}H NMR (500 MHz, $CDCl_3$) δ_H: 2.89 (4H, s, 2 × C*H*$_2$), 7.22 (1H, d, *J* 14.8, C(2)*H*), 7.32 – 7.39 (2H, m, Ar(2,6)*H*), 7.95 (1H, d, *J* 14.8, C(3)*H*), 8.26 – 8.33 (2H, m, Ar(3,5)*H*).

^{13}C{^{1}H} NMR (126 MHz, $CDCl_3$) δ_C: 27.8 (2 × *C*H$_2$), 108.8 (*C*(2)H), 122.4 (Ar*C*(2,6)H), 125.2 (Ar*C*(3,5)H), 133.2 (*C*(3)H), 145.3 (Ar*C*(4)), 155.3 (Ar*C*(1)), 164.3 (*C*(1)=O), 174.1 (2 × *C*=O$_{imide}$).

HRMS (ESI$^+$) $C_{14}H_{14}N_2O_7Na$ $[M+MeOH+Na]^+$ found 345.0690, requires 345.0693 (−0.8 ppm).

ν_{max} (film, cm^{-1}) 3134, 3109, 3008 (=C-H), 2947 (C-H), 2866 (C-H), 1784 (C=O$_{imide}$), 1739, 1726 (C=O$_{ester}$), 1716, 1627, 1618, 1591, 1519 (NO_2), 1490, 1425, 1379, 1350, 1205, 1093, 850.

Benzyl *t*-butyl fumarate (S37)

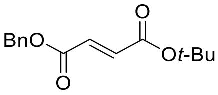

$C_{15}H_{18}O_4$
262.31

Adapting the procedure reported by Smith and co-workers[3], acid **S27** (700 mg, 4.0 mmol, 1.0 eq), DCC (923 mg, 4.4 mmol, 1.1 eq) and DMAP (99 mg, 0.8 mmol, 0.2 eq) were dissolved in CH_2Cl_2 (4 mL) and cooled to 0 °C. BnOH (0.43 mL, 4.1 mmol, 1.02 eq) was added dropwise and the reaction mixture allowed to warm to room temperature overnight. The mixture was filtered to remove the by-product dicyclohexylurea, the solid was washed with CH_2Cl_2 and the combined filtrates concentrated. The crude product was purified by silica column chromatography (petrol : EtOAc 9:1, R_f 0.43) to afford the title compound as an off-white solid (758 mg, 71%). **m.p.** (EtOAc) 85 – 88 °C.

^{1}**H NMR** (400 MHz, $CDCl_3$) δ_H: 1.49 (9H, s, C(C*H*$_3$)$_3$), 5.23 (2H, s, OC*H*$_2$), 6.78 (1H, d, *J* 15.8, C(2)*H* or C(3)*H*), 6.82 (1H, d, *J* 15.8, C(2)*H* or C(3)*H*), 7.33 – 7.40 (5H, m, 5 × Ar*H*). Spectroscopic data in accordance with literature.[3]

(*E*)-4-(Benzyloxy)-4-oxobut-2-enoic acid (S38)

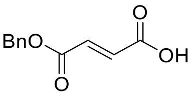

$C_{11}H_{10}O_4$
206.20

Following **General Procedure C**, benzyl *t*-butyl fumarate **S37** (758 mg, 2.8 mmol, 1.0 eq) in TFA : CH_2Cl_2 1:2 (4 mL) after 4 h gave the title compound as an off-white solid (600 mg, >99%), which was used without further purification.

^{1}**H NMR** (400 MHz, DMSO d$_6$) δ_H: 5.23 (2H, s, OC*H*$_2$), 6.74 (2H, s, C*H*=C*H*), 7.32 – 7.44 (5H, m, 5 × Ar*H*). Spectroscopic data in accordance with literature.[32]

Benzyl (4-nitrophenyl) fumarate (S39)

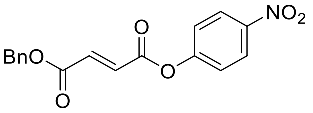

$C_{17}H_{13}NO_6$
327.29

Adapting the procedure reported by Smith and co-workers[30], acid **S38** (594 mg, 2.8 mmol, 1.0 eq) and EDCI·HCl (718 mg, 3.7 mmol, 1.3 eq) were dissolved in anhydrous CH_2Cl_2 (5 mL) and stirred for 10 min at room temperature. 4-Nitrophenol (600 mg, 4.3 mmol, 1.5 eq) was added, the reaction mixture stirred for 16 h and quenched by the addition of H_2O (5 mL). The phases were separated, and the aqueous phase was extracted with CH_2Cl_2 (3 × 5 mL). The combined organic phases were dried over $MgSO_4$, filtered and the solvent was removed under reduced pressure. The crude product was purified by silica column chromatography (petrol : EtOAc 6:1, R_f 0.26) to afford the title compound as a white solid (370 mg, 39%). **m.p.** (EtOAc) 75 – 78 °C {Lit.[3] 74 – 76 °C (EtOAc)}.

^{1}**H NMR** (400 MHz, $CDCl_3$) δ_H: 5.29 (2H, s, OC*H*$_2$), 7.07 (1H, d, *J* 15.8, C(2)*H* or C(3)*H*), 7.13 (1H, d, *J* 15.8, C(2)*H* or C(3)*H*), 7.32 – 7.43 (7H, m, 5 × Ar*H*$_{Bn}$, Ar(2,6)*H*), 8.27 – 8.33 (2H, m, Ar(3,5)*H*). Spectroscopic data in accordance with literature.[3]

(*E*)-3-(Phenylmethoxy)-2-propenoic acid, methyl ester (S40)

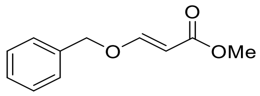

$C_{11}H_{12}O_3$
192.21

Adopting the procedure reported by García-Tellado and co-workers,[33] to a stirred solution of methyl propiolate (0.26 mL, 2.9 mmol, 1.0 eq) and BnOH (0.34 mL, 3.2 mmol, 1.1 eq) in anhydrous CH_2Cl_2 (10 mL) was added DABCO (33.0 mg, 0.29 mmol, 0.1 eq) and the reaction was stirred at room temperature until TLC (Petrol : EtOAc 4:1) indicated complete conversion (ca. 2 h). The reaction mixture was concentrated under reduced pressure and purified by silica column chromatography (Petrol : EtOAc 9:1 to 4:1, R_f 0.49 in Petrol : EtOAc 4:1) to give the title compound as a colourless liquid (568 mg, 99%).

^{1}H NMR (400 MHz, $CDCl_3$) δ_H: 3.73 (3H, s, CO_2CH_3), 4.93 (2H, s, CH_2-O), 5.35 (1H, d, *J* 12.6, C(2)*H*), 7.34 – 7.45 (5H, m, Ar-H_5), 7.71 (1H, d, *J* 12.6, C(3)*H*).

Spectroscopic data in accordance with literature.[34]

(*E*)-3-(Benzyloxy)prop-2-enoic acid (S41)

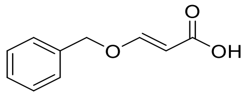

$C_{10}H_{10}O_3$
178.19

Methyl ester **S40** (569 mg, 2.96 mmol, 1.0 eq) was stirred in a mixture of NaOH (1 M, 3.3 mL, 1.1 eq) and 1,4-dioxane (3.3 mL) at room temperature until TLC (petrol : EtOAc 4:1) indicated complete conversion (ca. 48 h). The reaction mixture was acidified with 2 M HCl to pH 1 and extracted with CH_2Cl_2 (3 × 10 mL). The combined organic extracts were dried over $MgSO_4$, filtered and concentrated under reduced pressure to yield the title compound as a white solid (500 mg, 94%), which was used without further purification.

^{1}H NMR (400 MHz, DMSO-d_6) δ_H: 4.99 (2H, s, OCH_2), 5.25 (1H, d, *J* 12.4, C(2)*H*), 7.27 – 7.44 (5H, m, 5 × Ar*H*), 7.61 (1H, d, *J* 12.4, C(3)*H*).

Spectroscopic data in accordance with literature.[35]

(*E*)-3-(Benzyloxy)prop-2-enoic acid, 4-nitrophenol ester (S42)

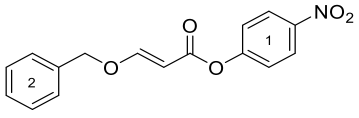

$C_{16}H_{13}NO_5$
299.28

Following **General Procedure D**, acid **S41** (500 mg, 2.8 mmol, 1.0 eq), oxalyl chloride (0.25 mL, 2.9 mmol, 1.05 eq) and DMF (1 drop) in anhydrous CH_2Cl_2 (8.5 mL) followed by 4-nitrophenol (390 mg, 2.8 mmol, 1.0 eq) and *i*-Pr_2NEt (0.98 mL, 5.6 mmol, 2.0 eq) in anhydrous CH_2Cl_2 (8.5 mL) gave a brown solid, which was triturated with Et_2O. The colourless solid (*i*-Pr_2NEt·HCl) was filtered, washed with Et_2O and the filtrate was concentrated under reduced pressure. The crude solid was purified by Biotage® Isolera™ 4 [SNAP KP-Sil 50 g, 100 mL min^{-1}, petrol : EtOAc 90:10 to 75:25 (10 CV), R_f 0.45 in petrol : EtOAc 4:1] followed by recrystallisation from Et_2O to afford the title compound as a white crystalline solid (265 mg, 31%). **m.p.** (Et_2O) 106 – 109 °C.

^{1}H NMR (500 MHz, $CDCl_3$) δ_H: 5.02 (2H, s, OC*H*$_2$), 5.51 (1H, d, *J* 12.5, C(2)*H*), 7.27 – 7.34 (2H, m, Ar^1(2,6)*H*), 7.35 – 7.46 (5H, m, 5 × Ar^2*H*), 7.88 (1H, d, *J* 12.5, C(3)*H*), 8.23 – 8.31 (2H, m, Ar^1(3,5)*H*).

^{13}C{^{1}H} NMR (126 MHz, $CDCl_3$) δ_C: 73.6 (O*C*H$_2$), 96.0 (*C*(2)H), 122.5 (Ar^1*C*(2,6)H), 125.1 (Ar^1*C*(3,5)H), 127.8 (Ar^2*C*(3,5)H), 128.9 (Ar^2*C*(2,4,6)H), 134.6 (Ar^2*C*(1)), 145.0 (Ar^1*C*(4)), 155.6 (Ar^1*C*(1)), 164.7 (*C*(3)H), 165.0 (*C*(1)=O).

HRMS (ESI$^+$) $C_{16}H_{13}NO_5Na$ $[M+Na]^+$ found 322.0683, requires 322.0685 (−0.8 ppm).

ν_{max} (film, cm^{-1}) 3120, 3101, 3086, 2845 (C-H), 1718 (C=O), 1685, 1614, 1589 (C=C_{Ar}), 1514 (NO_2), 1489 (C=C_{Ar}), 1334 (NO_2), 1236, 1205, 1165, 1105, 985, 974.

(*E*)-3-(Pyridin-2-yl)-2-propenoic acid, ethyl ester (S43)

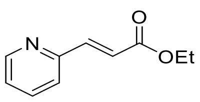

$C_{10}H_{11}NO_2$
177.20

Triethylphosphonoacetate (2.2 mL, 11.0 mmol, 1.1 eq), LiCl (466 mg, 11.0 mmol, 1.1 eq) and *i*-Pr_2NEt (1.9 mL, 11.0 mmol, 1.1 eq) were stirred in anhydrous MeCN (33 mL) for 30 min at room temperature. A solution of 2-pyridinecarboxaldehyde (0.95 mL, 10.0 mmol, 1.0 eq) in anhydrous MeCN (33 mL) was added slowly and the reaction mixture allowed to stir overnight. The resulting orange suspension was diluted with H_2O (40 mL) and extracted with EtOAc (3 × 30 mL). The combined organic phases were washed with brine,

dried over $MgSO_4$, filtered and concentrated under reduced pressure. The crude product was purified by Biotage® Select™ [Sfär 50 g, 120 mL min^{-1}, EtOAc in petrol (12% to 100%, 20 CV), R_f 0.5 in petrol : EtOAc 1:1] to afford the title compound as a yellow oil (1.34 g, 75%).

^{1}H NMR (500 MHz, $CDCl_3$) δ_H: 1.33 (3H, t, *J* 7.1, OCH_2CH_3), 4.27 (2H, q, *J* 7.1, OCH_2CH_3), 6.92 (1H, d, *J* 15.8, C(2)*H*), 7.26 – 7.30 (1H, m, Ar(5)*H*), 7.43 (1H, d, *J* 7.7, Ar(3)*H*), 7.68 (1H, d, *J* 15.8, C(3)*H*), 7.70 – 7.75 (1H, m, Ar(4)*H*), 8.62 – 8.69 (1H, m, Ar(6)*H*).

Spectroscopic data in accordance with literature.[36]

(*E*)-3-(Pyridin-2-yl)-2-propenoic acid (S44)

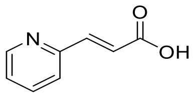

$C_8H_7NO_2$
149.15

Following **General Procedure B**, ethyl ester **S43** (976 mg, 5.5 mmol, 1.0 eq) and LiOH·H_2O (254 mg, 6.1 mmol, 1.1 eq) in H_2O : THF 1:1 (5 mL) were stirred for 2 h and the reaction mixture acidified with 2 M HCl to pH 2. The formed precipitate was filtered, washed with H_2O and Et_2O and dried under reduced pressure to afford the title compound as a white solid (585 mg, 71%). **m.p.** 160 °C (dec).

^{1}H NMR (500 MHz, DMSO d_6) δ_H: 6.82 (1H, d, *J* 15.7, C(2)*H*), 7.39 (1H, ddd, *J* 7.6, 4.8, 1.2, Ar(5)*H*), 7.59 (1H, d, *J* 15.7, C(3)*H*), 7.71 (1H, d, *J* 7.8, Ar(3)*H*), 7.85 (1H, app. td, *J* 7.7, 1.8, Ar(4)*H*), 8.63 (1H, d, *J* 4.8, Ar(6)*H*), 12.62 (1H, br s, O*H*).

Spectroscopic data in accordance with literature.[37]

(*E*)-3-(Pyridin-2-yl)-2-propenoic acid, 4-nitrophenol ester (S45)

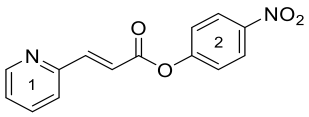

$C_{14}H_{10}N_2O_4$
270.24

Following **General Procedure D**, acid **S44** (559 g, 3.74 mmol, 1.0 eq), oxalyl chloride (0.34 mL, 3.93 mmol, 1.05 eq) and DMF (1 drop) in anhydrous CH_2Cl_2 (12 mL) followed by 4-nitrophenol (520 mg, 3.74 mmol, 1.0 eq) and *i*-Pr_2NEt (1.29 mL, 7.48 mmol, 2.0 eq) in anhydrous CH_2Cl_2 (12 mL) gave a black solid, which was purified by Biotage® Select™ [Sfär 50 g, 120 mL min^{-1}, EtOAc in petrol (15% to 100%, 20 CV), R_f 0.47 in petrol : EtOAc

1:1] to give a pale yellow solid. Trituration with Et_2O removed remaining *p*-nitrophenol impurities to afford the title compound as a beige solid (326 mg, 32%).

m.p. (Et_2O) 190 – 192 °C.

^{1}H NMR (500 MHz, $CDCl_3$) δ_H: 7.18 (1H, d, *J* 15.6, C(2)*H*), 7.33 – 7.43 (3H, m, Ar^1(5)*H*, Ar^2(2,6)*H*), 7.50 (1H, d, *J* 7.7, Ar^1(3)*H*), 7.79 (1H, app. td, *J* 7.7, 1.8, Ar^1(4)*H*), 7.90 (1H, d, *J* 15.6, C(3)*H*), 8.28 – 8.35 (2H, m, Ar^2(3,5)*H*), 8.71 (1H, d, *J* 4.6, Ar(6)*H*).

^{13}C{^{1}H} NMR (126 MHz, $CDCl_3$) δ_C:120.6 (*C*(2)H), 122.4 (Ar^2*C*(2,6)H), 125.0 (Ar^1*C*(3,5)H), 125.2 (Ar^2*C*(3,5)H), 137.1 (Ar^1*C*(4)H), 145.3 (Ar^2*C*(4)), 146.1 (*C*(3)H), 150.2 (Ar^1*C*(6)H), 152.0 (Ar^1*C*(2)), 155.5 (Ar^2*C*(1)), 164.1 (*C*=O).

HRMS (ESI$^+$) $C_{14}H_{11}N_2O_4$ $[M+H]^+$ found 271.0704, requires 271.0713 (−3.6 ppm).

ν_{max} (film, cm^{-1}) 3113, 3066, 1764, 1728 (C=O), 1637, 1577 (C=C_{Ar}), 1512 (NO_2), 1433, 1344 (NO_2), 1321, 1219, 1130, 985.

(*Z*)-4-ethoxy-4-oxobut-2-enoic acid (S46)

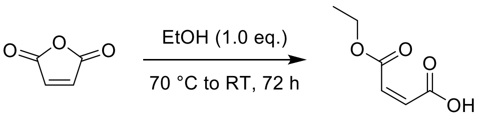

Following the procedure of Smith and co-workers[3], maleic anhydride (0.98 g, 10 mmol, 1.0 eq.) was melted in a sealed tube at 70 °C. EtOH (0.58 mL, 10 mmol, 1.0 eq.) was added and the reaction mixture stirred at rt for 72 h. Upon completion, the reaction mixture was dried under reduced pressure to afford the title compound as a colourless oil (1.31 g, 91%), which was used without further purification.

^{1}H NMR (400 MHz, $CDCl_3$) δ_H: 1.40 (3H, t, *J* 7.1, CH_2CH_3), 4.39 (2H, q, *J* 7.1, CH_2CH_3), 6.40 (1H, d, *J* 12.8, C*H*=CH), 6.51 (1H, d, *J* 12.8, CH=C*H*).

Spectroscopic data in accordance with literature.[3]

Ethyl (4-nitrophenyl) maleate (22)

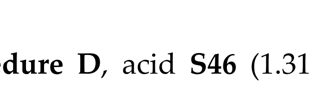

$C_{12}H_{11}NO_6$
265.22

Following **General Procedure D**, acid **S46** (1.31 g, 9.1 mmol, 1.0 eq), oxalyl chloride (0.81 mL, 9.6 mmol, 1.05 eq) and DMF (3 drops) in anhydrous CH_2Cl_2 (28 mL) followed by 4-nitrophenol (1.27 g, 9.1 mmol, 1.0 eq) and *i*-Pr_2NEt (3.2 mL, 18.0 mmol, 2.0 eq) in

anhydrous CH_2Cl_2 (28 mL) gave a dark solid, which was purified by silica column chromatography (Petrol : EtOAc 9:1 to 6:1, R_f 0.1 in Petrol : EtOAc 9:1) to give the title compound as a yellow solid (770 mg, 32%). **m.p.** (EtOAc) 59-61 °C {lit[3] 58-60 °C}.

^{1}H NMR (400 MHz, $CDCl_3$) δ_H: 1.33 (3H, t, *J* 7.1, CH_2CH_3), 4.29 (2H, q, *J* 7.1, CH_2CH_3), 6.46 (1H, d, *J* 11.8, C*H*=CH), 6.50 (1H, d, *J* 11.8, CH=C*H*), 7.39 – 7.46 (2H, m, ArC(2,6)*H*), 8.28 – 8.36 (2H, m, ArC(3,5)*H*). Spectroscopic data in accordance with literature.[3]

Methyl 2-(bis(2,2,2-trifluoroethoxy)phosphoryl)acetate (S47)

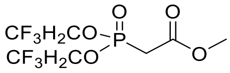

$C_7H_9F_6O_5P$
318.11

Following the procedure of Nakao and co-workers[38], TMSBr (16.5 mL, 125 mmol, 2.5 eq) was added to a solution of trimethyl phosphonoacetate (8.1 mL, 50 mmol, 1.0 eq) in anhydrous CH_2Cl_2 (100 mL) under Ar and stirred at rt for 24 h. The solvent was removed under reduced pressure to give 2-(bis(trimethylsilyl)oxy)-phosphoryl)acetate, which was dissolved in anhydrous $CHCl_3$ (330 mL) under Ar. PPh_3 (32.8 g, 125 mmol, 2.5 eq) and I_2 (31.7 g, 125 mmol, 2.5 eq) were added and the reaction mixture stirred at rt for 15 min. Imidazole (34.0 g, 500 mmol, 10 eq) was added, the mixture stirred at rt for 15 min followed by 30 min at 50 °C. 2,2,2-Trifluoroethanol (14.6 mL, 200 mmol, 4.0 eq) was added and the reaction mixture stirred at 60 °C for 5 h. Once complete, the reaction mixture was filtered, the filtrate was concentrated under reduced pressure and the crude product was purified by silica column chromatography (hexane : EtOAc 2:1, R_f 0.21) to give the title compound as a colourless oil (11.2 g, 70%).

^{1}H NMR (400 MHz, $CDCl_3$) δ_H: 3.19 (2H, d, $^2J_{HP}$ 21.1, C*H*$_2$P), 3.80 (3H, s, OC*H*$_3$), 4.42 – 4.54 (4H, m, OC*H*$_2$CF$_3$).

^{19}F{^{1}H} NMR (376 MHz, $CDCl_3$) δ_F: -75.4 (s).

Spectroscopic data in accordance with literature.[38]

(*Z*)-4,4,4-trifluorobut-2-enoic acid (S48)

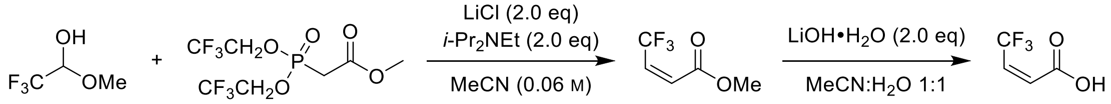

A flame-dried flask was charged with methyl 2-(bis(2,2,2-trifluoroethoxy)-phosphoryl)acetate **S47** (11.1 g, 34.8 mmol, 1.2 eq), LiCl (2.46 g, 58.0 mmol, 2.0 eq), *i*-Pr_2NEt (10.1 mL, 58.0 mmol, 2.0 eq) and MeCN (240 mL). The reaction mixture was stirred at rt for 30 min, followed by the slow addition of a solution of 2,2,2-trifluoro-1-methoxyethan-1-ol (3.10 mL, 29.0 mmol, 1.0 eq) in MeCN (240 mL). The reaction mixture was stirred at rt until complete by TLC (ca. 20 h), concentrated under reduced pressure to a volume of ca. 180 mL and was diluted with an equal amount of H_2O (180 mL). LiOH·H_2O (1.24 g, 29.7 mmol) was added in portions and the reaction mixture was stirred at rt for 24 h. Once complete, MeCN was removed under reduced pressure and the remaining aqueous solution acidified to pH 2 with 2 M HCl. The aqueous phase was extracted with EtOAc (2 × 150 mL) and the combined organic phases were washed with H_2O (3 × 150 mL), dried over $MgSO_4$ and the solvent was removed under reduced pressure to afford the title compound (1.44 g, 35% over two steps) as a colourless glass as a mixture of isomers (5:1 (*Z*):(*E*)), which was used without further purification.

Note: Caution should be taken when drying the product under reduced pressure as the (E)-isomer was found to be volatile. It was found that drying a small sample of a 5:1 (Z):(E) mixture of isomers increased the ratio to (Z):(E) 17:1, but with significant loss in mass.

ν_{max} (film, cm^{-1}) 3075 (br, O-H), 1717 (C=O), 1433, 1310, 1283, 1196, 1121, 926.

HRMS (ESI^-) $C_4H_2O_2F_3$ $[M-H]^-$ found 139.0009, requires 139.0012 (−2.2 ppm).

Data for (Z)-isomer:

^{1}H NMR (400 MHz, $CDCl_3$) δ_H: 6.19 (1H, dq, *J* 12.5, 8.0, C(3)*H*), 6.35 (1H, d, *J* 12.5, C(2)*H*), 10.9 (1H, br s, O*H*).

^{13}C{^{1}H} NMR (126 MHz, $CDCl_3$) δ_C: 120.9 (q, $^1J_{CF}$ 272.1, *C*F_3), 128.8 (q, $^3J_{CF}$ 5.4, *C*(2)H), 129.5 (q, $^2J_{CF}$ 37.5, *C*(3)H), 168.4 (*C*=O).

^{19}F{^{1}H} NMR (376 MHz, $CDCl_3$) δ_F: -60.5 (s).

(*Z*)-4,4,4-Trifluorobut-2-enoic acid, 4-nitrophenyl ester (23)

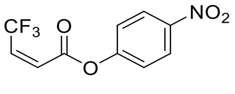

$C_{10}H_6F_3NO_4$
261.16

Note: Attempted synthesis following ***General Procedure D*** *resulted only in the formation of the corresponding (E)-isomer* ***3****!*

4,4,4-trifluorobut-2-enoic acid **S48** (5:1 mixture of (*Z*):(*E*) isomers) (0.28 g, 2.0 mmol, 1.0 eq) and DCC (0.41 g, 2.0 mmol, 1.0 eq) were dissolved in anhydrous CH_2Cl_2 (20 mL) and the reaction mixture was stirred at rt for 30 min. 4-Nitrophenol (0.28 g, 2.0 mmol, 1.0 eq) was added and the reaction mixture stirred at rt for 20 h. The solvent was removed under reduced pressure to give the crude product as a 5:1 mixture of (*Z*):(*E*) isomers, which was purified by silica column chromatography (hexane : EtOAc 9:1, R_f 0.57 in hexane : EtOAc 7:3) to give the title compound as a colourless solid (0.34 g, 66%).

m.p. 70-72 °C.

^{1}H NMR (400 MHz, $CDCl_3$) δ_H: 6.32 (1H, dq, *J* 12.4, 7.9, C(3)*H*), 6.54 (1H, d, *J* 12.4, C(2)*H*), 7.34 – 7.42 (2H, m, ArC(2,6)*H*), 8.27 – 8.38 (2H, m, ArC(3,5)*H*).

^{13}C{^{1}H} NMR (126 MHz, $CDCl_3$) δ_C: 121.0 (q, $^1J_{CF}$ 272.2, *C*F3), 122.2 (Ar*C*(2,6)H), 125.4 (ArC(3,5)H), 128.2 (q, $^3J_{CF}$ 5.3, *C*(2)H), 129.9 (q, $^2J_{CF}$ 37.3, *C*(3)H), 145.8 (ArC(4)), 154.4 (Ar*C*(1)), 160.6 (*C*=O).

^{19}F{^{1}H} NMR (376 MHz, $CDCl_3$) δ_F: -60.5 (s).

ν_{max} (film, cm^{-1}) 3075 (C-H), 1755 (C=O), 1531, 1410, 1346, 1128, 1013, 941, 866.

HRMS (MALDI (no matrix)$^+$) $C_{10}H_6NO_4F_3Na$ [M+Na]$^+$ found 284.0133, requires 284.0141 (−2.8 ppm).

The minor (*E*)-isomer 3 could also be isolated (R_f 0.85 in hexane : EtOAc 7:3) (0.06 g, 12%).

10 Cooperative Pd and ITU catalysis products

(1*R*,2*S*,5*S*)-3,3-Dicyano-2-(trifluoromethyl)-5-vinylcyclopentane-1-carboxylic acid, 4-nitrophenyl ester (4)

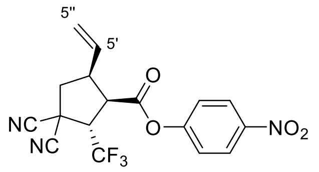

$C_{17}H_{12}F_3N_3O_4$
379.30

Following **General Procedure E**, PNP ester **3** (261 mg, 1.0 mmol, 1.0 eq), $Pd(PPh_3)_4$ (57.8 mg, 0.05 mmol, 5 mol%), (*R*)-BTM (50.5 mg, 0.2 mmol, 20 mol%), LiCl (0.5 M in THF, 0.6 mL, 0.3 mmol, 30 mol%) and VCP **2** (118 mg, 1.0 mmol, 1.0 eq) in THF : EtOAc 3:2 (5 mL) for 24 h gave the crude product (95:5 dr). Purification by silica column chromatography (*n*-hexane : EtOAc 4:1, R_f 0.23) gave a pale yellow oil, which was triturated with Et_2O to afford the title compound as an inseparable mixture of diastereomers (95:5 dr) as an off-white solid (97 mg, 51%). **m.p.** (Et_2O) 96 – 99 °C.

$[\alpha]_D^{20}$ +43.7 (*c* 1.1 in $CHCl_3$).

HRMS (ESI⁻) $C_{17}H_{11}F_3N_3O_4$ $[M-H]^-$ found 378.0699, requires 378.0707 (−2.1 ppm).

ν_{max} ($CHCl_3$, cm^{-1}) 3086 (=C-H), 2951 (C-H), 2258 (C≡N), 1762 (C=O), 1618, 1593 (C=C_{Ar}), 1525 (C-NO_2), 1489 (C=C_{Ar}), 1346 (C-NO_2), 1271, 1201, 1128.

Data for major diastereoisomer 4_{maj}

chiral HPLC analysis Chiralcel OD-H (hexane : *i*-PrOH 97:3, flow rate 1.0 $mlmin^{-1}$, 254 nm, 40 °C) t_R (1*S*,2*R*,5*R*): 30.1 min, t_R (1*R*,2*S*,5*S*): 32.3 min, 7:93 er.

^{1}H NMR (500 MHz, $CDCl_3$) δ_H: 2.58 (1H, dd, *J* 13.2, 12.0, C(4)H^AH^B), 2.90 (1H, dd, *J* 13.2, 6.0, C(4)H^AH^B), 3.61 – 3.70 (1H, m, C(5)*H*), 3.74 (1H, dd, *J* 10.8, 8.4, C(1)*H*), 3.85 – 3.98 (1H, m, C(2)*H*), 5.41 – 5.47 (2H, m, C(5'')H_2), 5.82 (1H, ddd, *J* 17.1, 10.2, 7.9, C(5')*H*), 7.25 – 7.31 (2H, m, Ar(2,6)*H*), 8.28 – 8.34 (2H, m, Ar(3,5)*H*).

^{13}C{^{1}H} NMR (126 MHz, $CDCl_3$) δ_C: 34.3 (*C*(3)), 43.5 (*C*(4)H_2), 44.1 (*C*(5)H), 45.7 (*C*(1)H), 55.0 (q, $^2J_{CF}$ 29.8, *C*(2)H) , 111.9 (*C*N), 112.9 (*C*N), 121.4 (*C*(5'')H_2), 122.2 (Ar*C*(2,6)H), 123.6 (q, $^1J_{CF}$ 280, *C*F_3), 125.4 (Ar*C*(3,5)H), 131.7 (*C*(5')H), 145.9 (Ar*C*(4)), 154.3 (Ar*C*(1)), 168.2 (*C*=O).

^{19}F{^{1}H} NMR (376 MHz, $CDCl_3$) δ_F: −67.3 (s, C*F*$_3$).

Data for minor diastereoisomer ***4**$_{min}$*

chiral HPLC analysis Chiralcel OD-H (hexane : *i*-PrOH 97:3, flow rate 1.0 mlmin^{-1}, 254 nm, 40 °C) t_R (1*S*,2*R*,5*S*): 40.4 min, t_R (1*R*,2*S*,5*R*): 45.2 min, <5:95 er.

^{1}H NMR (500 MHz, $CDCl_3$) (*selected*) δ_H: 3.00 (1H, dd, *J* 13.7, 6.8, C(4)H^AH^B), 3.30 – 3.38 (2H, m, C(1)*H* and C(5)*H*), 5.37 – 5.41 (2H, m, C(5'')H_2), 5.92 (1H, ddd, *J* 16.9, 10.2, 7.5, C(5')*H*).

^{13}C{^{1}H} NMR (126 MHz, $CDCl_3$) (*selected*) δ_C: 44.6 (*C*(4)H_2), 47.1 (*C*(5)H), 49.7 (*C*(1)H), 120.2 (*C*(5'')H_2), 134.0 (*C*(5')H), 167.9 (*C*=O).

^{19}F{^{1}H} NMR (376 MHz, $CDCl_3$) δ_F: −67.0 (s, CF_3).

(1*R*,2*S*,5*S*)-3,3-Dicyano-2-(trifluoromethyl)-5-vinylcyclopentane-1-carboxylic acid, methyl ester (8)

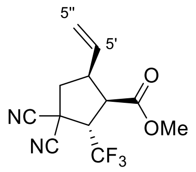

$C_{12}H_{11}F_3N_2O_2$
272.23

Following **General Procedure E**, PNP ester **3** (261 mg, 1.0 mmol, 1.0 eq), $Pd(PPh_3)_4$ (57.8 mg, 0.05 mmol, 5 mol%), (*R*)-BTM (50.5 mg, 0.2 mmol, 20 mol%), LiCl (0.5 M in THF, 0.6 mL, 0.3 mmol, 30 mol%) and VCP **2** (118 mg, 1.0 mmol, 1.0 eq) in THF : EtOAc 3:2 (5 mL) for 24 h followed by MeOH (1.0 mL) and DMAP (24.4 mg, 0.2 mmol, 20 mol%) for 24 h gave the crude product (95:5 dr). Purification by silica column chromatography (*n*-hexane : EtOAc 4:1 to 3:1, R_f 0.42 in *n*-hexane : EtOAc 3:1) gave the title compound as an inseparable mixture of diastereomers (95:5 dr) as a pale yellow oil (205 mg, 75%), which slowly solidifies at ca. −18 °C.

8 was also synthesised starting from isolated cyclopentane PNP ester **4**:

To a solution of PNP ester **4** (379 mg, 1.0 mmol, 1.0 eq, 95:5 dr) in EtOAc (4.0 mL) were added anhydrous MeOH (1.0 mL, 25.0 mmol, 25.0 eq) and DMAP (24 mg, 0.2 mmol, 0.2 eq) and the reaction mixture was stirred at room temperature for 24 h. The reaction mixture was diluted with EtOAc (10 mL), washed with 1 M NaOH (2 × 10 mL) and brine (10 mL), dried over $MgSO_4$, filtered and the solvent was removed under reduced pressure. The crude product was purified by silica column chromatography (*n*-hexane : EtOAc 4:1,

R_f 0.35) to afford the title compound as an inseparable mixture of diastereoisomers (95:5 dr) as a colourless oil (247 mg, 90%), which slowly solidifies at ca. −18 °C.

m.p. (EtOAc) 69 – 70 °C. $[\alpha]_D^{20}$ +30.6 (*c* 1.31 in $CHCl_3$).

HRMS (ESI$^-$) $C_{12}H_{10}F_3N_2O_2$ [M−H]$^-$ found 271.0698, requires 271.0699 (−0.6 ppm).

ν_{max} (film, cm^{-1}) 3088 (C=CH_2), 2956 (C-H), 1724 (C=O), 1643 (C=C), 1444, 1408, 1369, 1271, 1247, 1224, 1168, 1132, 935.

*Data for major diastereoisomer **8**$_{maj}$*

Chiral GC analysis, Restek Rt-βDEXcst (length: 30 m, thickness: 0.25 mm, film thickness: 0.25 μm, carrier gas: He, linear velocity: 28 $cmsec^{-1}$, temperature: 120 °C (60 min), 120 to 140 °C (20 min)) t_R (1*R*,2*S*,5*S*): 64.5 min, t_R (1*S*,2*R*,5*R*): 65.5 min, 93:7 er.

^{1}H NMR (500 MHz, $CDCl_3$) δ_H: 2.45 – 2.55 (1H, m, C(4)H^AH^B), 2.74 (1H, dd, *J* 11.8, 4.3, C(4)H^AH^B), 3.36 – 3.46 (2H, m, C(1)*H*, C(5)*H*), 3.74 (3H, s, OCH_3), 3.87 (1H, p, *J* 7.7, C(2)*H*), 5.21 – 5.28 (2H, m, C(5′′)H_2), 5.56 – 5.65 (1H, m, C(5′)*H*).

^{13}C{^{1}H} NMR (126 MHz, $CDCl_3$) δ_C: 34.3 (*C*(3)), 43.4 (*C*(4)H_2), 44.2 (*C*(5)H), 45.9 (*C*(1)H), 52.9 (OCH_3), 55.1 (q, $^2J_{CF}$ 29.7, *C*(2)H), 112.2 (*C*N), 113.3 (*C*N), 120.2 (*C*(5′′)H_2), 123.8 (q, $^1J_{CF}$ 279, *C*F_3), 131.8 (*C*(5′)H), 170 (*C*=O).

^{19}F{^{1}H} NMR (377 MHz, $CDCl_3$) δ_F: −67.5 (CF_3).

*Data for minor diastereoisomer **8**$_{min}$ (selected)*

chiral GC analysis, Restek Rt-βDEXcst (length: 30 m, thickness: 0.25 mm, film thickness: 0.25 μm, carrier gas: He, linear velocity: 28 $cmsec^{-1}$, temperature: 120 °C (60 min), 120 to 140 °C (20 min)) t_R (1*R*,2*S*,5*R*): 69.8 min, t_R (1*S*,2*R*,5*S*): 72.7 min, >99:1 er.

^{1}H NMR (500 MHz, $CDCl_3$) δ_H: 2.86 (1 H, dd, *J* 13.7, 7.6, C(4)H^AH^B), 3.02 (1H, t, *J* 9.7, C(1)*H*), 3.78 (3H, s, OCH_3), 5.79 (1H, ddd, *J* 17.5, 10.1, 7.6, C(5′)*H*).

^{13}C{^{1}H} NMR (126 MHz, $CDCl_3$) δ_C: 44.5 (*C*(4)H_2), 46.5 (*C*(5)H), 49.6 (*C*(1)H), 53.2 (OCH_3), 119.3 (*C*(5′′)H_2), 134.4 (*C*(5′)H).

^{19}F{^{1}H} NMR (377 MHz, $CDCl_3$) δ_F: −67.3 (CF_3).

(1*R*,2*S*,5*S*)-3,3-Dicyano-2-(perfluoroethyl)-5-vinylcyclopentane-1-carboxylic acid, methyl ester (9)

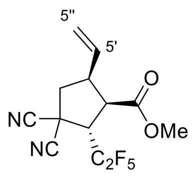

$C_{13}H_{11}F_5N_2O_2$
322.24

Following **General Procedure E**, PNP ester **S11** (311 mg, 1.0 mmol, 1.0 eq), $Pd(PPh_3)_4$ (57.8 mg, 0.05 mmol, 5 mol%), (*R*)-BTM (50.5 mg, 0.2 mmol, 20 mol%), LiCl (0.5 M in THF, 0.6 mL, 0.3 mmol, 30 mol%) and VCP **2** (118 mg, 1.0 mmol, 1.0 eq) in THF : EtOAc 3:2 (5 mL) for 24 h followed by MeOH (1.0 mL) and DMAP (24.4 mg, 0.2 mmol, 20 mol%) for 24 h gave the crude product (91:5:4 dr). Purification by silica chromatography (hexane : EtOAc 9:1, R_F 0.19) gave the title compound as a single diastereoisomer (>95:5 dr) as a colourless solid (172 mg, 53%). **m.p.** 67 – 69 °C. $[\alpha]_D^{20}$ +30.5 (*c* 2.0 in $CHCl_3$).

Chiral GC analysis Restek Rt-βDEXcst (length: 30 m, thickness: 0.25 mm, film thickness: 0.25 μm, carrier gas: He, linear velocity: 28 cmsec^{-1}, temperature: 60 to 140 °C (27 min), 140 °C (20 min)) t_R (1*S*,2*R*,5*R*): 37.8 min, t_R (1*R*,2*S*,5*S*): 38.2 min, 95:5 er.

^{1}H NMR (500 MHz, $CDCl_3$) δ_H: 2.45 – 2.54 (1H, m, C(4)H^AH^B), 2.70 – 2.76 (1H, m, C(4)H^AH^B), 3.40 – 3.50 (2H, m, C(1)*H*, C(5)*H*), 3.74 (3H, s, OCH_3), 3.95 (1H, ddd, *J* 21.2, 6.4, 4.9, C(2)*H*), 5.23 – 5.30 (2H, m, C(5'')H_2), 5.56 – 5.65 (1H, m, C(5')*H*).

^{13}C{^{1}H} NMR (126 MHz, $CDCl_3$) δ_C: 34.5 (*C*(3)), 42.9 (*C*(4)H_2), 44.8 (*C*(5)H), 45.9 (*C*(1)H), 52.8 (dd, $^2J_{CF}$ 22.0, 19.4, *C*(2)H), 52.9 (OCH_3), 112.5 (CN), 113.3 (tq, $^1J_{CF}$ 258.7, $^2J_{CF}$ 39.0, CF_2CF_3), 113.4 (CN), 118.0 (qt, $^1J_{CF}$ 286.2, $^2J_{CF}$ 35.1, CF_2CF_3), 120.2 (*C*(5'')H_2), 131.6 (*C*(5')*H*), 180.0 (*C*=O).

^{19}F{^{1}H} NMR (470 MHz, $CDCl_3$) δ_F: -120.8 (d, $^2J_{FF}$ 277.0, $CF^AF^BCF_3$), -116.5 (d, $^2J_{FF}$ 277.0, $CF^AF^BCF_3$), -83.7 (s, CF_2CF_3).

ν_{max} (film, cm^{-1}) 2957 (C-H), 1734 (C=O), 1267, 1244, 1202, 1144, 1028.

HRMS (EI$^+$) $C_{13}H_{11}O_2N_2F_5$ $[M]^+$ found 322.0724, requires 322.0735 (–3.4 ppm).

(1*R*,2*S*,5*S*)-3,3-Dicyano-2-(difluoromethyl)-5-vinylcyclopentane-1-carboxylic acid, methyl ester (10)

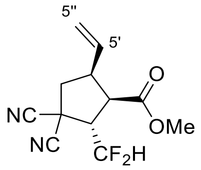

$C_{12}H_{12}F_2N_2O_2$
254.24

Following **General Procedure E**, PNP ester **S21** (243 mg, 1.0 mmol, 1.0 eq), $Pd(PPh_3)_4$ (57.8 mg, 0.05 mmol, 5 mol%), (*R*)-BTM (50.5 mg, 0.2 mmol, 20 mol%), LiCl (0.5 M in THF, 0.6 mL, 0.3 mmol, 30 mol%) and VCP **2** (118 mg, 1.0 mmol, 1.0 eq) in THF : EtOAc 3:2 (5 mL) for 24 h followed by MeOH (1.0 mL) and DMAP (24.4 mg, 0.2 mmol, 20 mol%) for 24 h gave the crude product (95:5dr). Purification by silica column chromatography (*n*-hexane : EtOAc 4:1, R_f 0.26) gave the title compound as an inseparable mixture of diastereomers (95:5 dr) as an off-white solid (187 mg, 74%). **m.p.** (EtOAc) 43 – 46 °C.
$[\boldsymbol{\alpha}]_D^{20}$ +33.1 (*c* 1.19 in $CHCl_3$).
HRMS (ESI^-): $C_{12}H_{11}F_2N_2O_2$ $[M{-}H]^-$ found 253.0757, requires 253.0794 (−3.7 ppm).
ν_{max} (film, cm^{-1}): 3088 (C-H), 2989, 2956 (C-H), 2256 (C≡N), 1732 (C=O), 1643 (C=C), 1438, 1240, 1207, 1178, 1087, 1055, 929.

Data for major diastereoisomer ***10**$_{maj}$*

Chiral GC analysis Restek Rt-βDEXcst (length: 30 m, thickness: 0.25 mm, film thickness: 0.25 μm, carrier gas: He, linear velocity: 28 $cmsec^{-1}$, temperature: 90 to 135 °C (45 min), 135 °C (40 min), 135 to 170 °C (35 min)) t_R (1*R*,2*S*,5*S*): 97.2 min, t_R (1*S*,2*R*,5*R*): 98.1 min, 90:10 er.

^{1}H NMR (500 MHz, $CDCl_3$) δ_H: 2.48 (1H, dd, *J* 13.1, 11.4, C(4)H^AH^B), 2.71 (1H, ddd, *J* 13.1, 6.1, 1.8 Hz, C(4)H^AH^B), 3.26 (1H, dd, *J* 10.4, 8.0, C(1)*H*), 3.29 – 3.39 (1H, m, C(5)*H*), 3.51 – 3.60 (1H, m, C(2)*H*), 3.71 (3H, s, OCH_3), 5.18 – 5.25 (2H, m, C(5'')H_2), 5.61 (1H, ddd, *J* 17.6, 10.1, 7.8, C(5')*H*), 6.06 (1H, td, *J* 54.6, 5.6, CF_2H).

^{13}C{^{1}H} NMR (126 MHz, $CDCl_3$) δ_C: 34.2 (dd, $^3J_{CF}$ 5.7, 1.5 *C*(3)), 43.0 (*C*(4)H_2), 44.0 (*C*(5)H), 46.1 (d, $^3J_{CF}$ 4.2, *C*(1)H), 52.6 (OCH_3), 54.4 (t, $^2J_{CF}$ 22.7. *C*(2)H), 112.9 (CN), 113.8 (CN), 114.4 (t, $^1J_{CF}$ 244, CF_2H), 119.8 (C(5'')H_2), 132.4 (*C*(5')H), 170.7 (*C*=O).

^{19}F{^{1}H} NMR (377 MHz, $CDCl_3$) δ_F: −121.1 (1F, d, $^2J_{FF}$ 297, CF^AF^B), −117.8 (1F, d, $^2J_{FF}$ 297, CF^AF^B).

Data for minor diastereoisomer ***10***$_{min}$ *(selected)*

Chiral GC analysis Restek Rt-βDEXcst (length: 30 m, thickness: 0.25 mm, film thickness: 0.25 μm, carrier gas: He, linear velocity: 28 cmsec^{-1}, temperature: 90 to 135 °C (45 min), 135 °C (40 min), 135 to 170 °C (35 min)) t_R (1*R*,2*S*,5*R*): 100.1 min, t_R (1*S*,2*R*,5*S*): 102.9 min, 88:12 er.

^{1}H NMR (500 MHz, $CDCl_3$) δ_H: 2.39 (1H, dd, *J* 13.4, 10.1, C(4)H^AH^B), 3.76 (3H, s, OCH_3), 5.78 (1H, ddd, *J* 17.0, 10.2, 7.6, C(5′)*H*).

^{13}C{^{1}H} NMR (126 MHz, $CDCl_3$) δ_C: 43.9 (*C*(4)H_2), 52.9 (OCH_3), 134.9 (*C*(5′)H).

^{19}F{^{1}H} NMR (377 MHz, $CDCl_3$) δ_F: −122.3 (1F, d, $^2J_{FF}$ 296, CF^AF^B), −119.8 (1F, d, $^2J_{FF}$ 296, CF^AF^B).

(1*R*,2*S*,5*S*)-3,3-Dicyano-2-((diethoxyphosphoryl)difluoromethyl)-5-vinylcyclopentane-1-carboxylic acid, methyl ester (11)

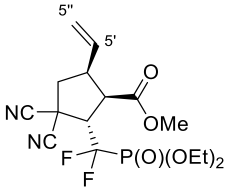

$C_{16}H_{21}F_2N_2O_5P$
390.32

Following **General Procedure E**, PNP ester **S25** (379 mg, 1.0 mmol, 1.0 eq), $Pd(PPh_3)_4$ (57.8 mg, 0.05 mmol, 5 mol%), (*R*)-BTM (50.5 mg, 0.2 mmol, 20 mol%), LiCl (0.5 M in THF, 0.6 mL, 0.3 mmol, 30 mol%) and VCP **2** (118 mg, 1.0 mmol, 1.0 eq) in THF : EtOAc 3:2 (5 mL) for 24 h followed by MeOH (1.0 mL) and DMAP (122 mg, 1.0 mmol, 1.0 eq) for 96 h gave the crude product (dr n.d.). Purification by silica column chromatography (*n*-hexane : EtOAc 2:1 to 1:1, R_f 0.23 in *n*-hexane : EtOAc 1:1) gave the title compound as a single diastereoisomer (>95:5 dr) as a white solid (73 mg, 19%). **m.p.** ($CHCl_3$) 59 – 61 °C. $[\alpha]_D^{20}$ +9.4 (*c* 1.06 in CH_2Cl_2).

Chiral HPLC analysis**,** Chiralpak AD-H, (*n*-hexane : *i*-PrOH 96:4, flow rate 1.5 mLmin^{-1}, 254 nm, 40 °C) t_R (1*R*,2*S*,5*S*): 26.8 min, t_R (1*S*,2*R*,5*R*): 30.9 min, 81:19 er. (determined from PNP ester from crude reaction mixture (0.2 mL aliquot, filtered over silica) before addition of MeOH)

^{1}H NMR (500 MHz, $CDCl_3$) δ_H: 1.38 (6H, t, *J* 7.1, 2 × OCH_2CH_3), 2.44 (1H, app. t, *J* 12.9, C(4)H^AH^B), 2.63 (1H, dd, *J* 12.7, 5.3, C(4)H^AH^B), 3.29 – 3.39 (1H, m, C(5)*H*), 3.41 – 3.48 (1H,

m, C(1)*H*), 3.69 (3H, s, OC*H*3), 3.90 (1H, dddd, *J* 25.0, 6.9, 5.1, 3.2, C(2)*H*), 4.21 – 4.36 (4H, m, 2 × OC*H*2CH3), 5.14 – 5.24 (2H, m, C(5'')*H*2), 5.59 (1H, ddd, *J* 16.8, 10.6, 7.4, C(5')*H*).

^{13}C{^{1}H} NMR (126 MHz, CDCl3) δ_C: 16.3 (t, $^{3}J_{CP}$ 4.6, 2 × OCH2CH3), 34.0 – 34.2 (m, *C*(3)), 42.9 (*C*(4)H2), 45.0 (*C*(5)H), 46.3 – 46.5 (m, *C*(1)H), 52.5 (OCH3), 55.2 (dt, $^{2}J_{CP}$ 21, $^{2}J_{CF}$ 18, *C*(2)H), 65.4 (d, $^{2}J_{CP}$ 6.9, OCH2CH3), 65.7 (d, $^{2}J_{CP}$ 6.9, OCH2CH3), 113.4 (*C*N), 114.4 (*C*N), 117.8 (td, $^{1}J_{CF}$ 266, $^{1}J_{CP}$ 215, *C*F2P), 119.3 (*C*(5'')H2), 132.2 (*C*(5')H), 171.7 (*C*=O).

^{19}F{^{1}H} NMR (377 MHz, CDCl3) δ_F: −118.2 (1F, dd, $^{2}J_{FF}$ 307, $^{2}J_{FP}$ 104, C*F*^{A}F^{B}), −111.5 (1F, dd, $^{2}J_{FF}$ 307, $^{2}J_{FP}$ 94, CF^{A}*F*^{B}).

^{31}P{^{1}H} NMR (202 MHz, CDCl3) δ_P: 3.36 (1P, dd, $^{2}J_{PF}$ 104, 94).

HRMS (ESI$^+$): $C_{16}H_{21}F_2N_2O_5PNa$ [M+Na]$^+$ found 413.1038, requires 413.1048 (−2.5 ppm).

ν$_{max}$ (film, cm^{-1}): 2991 (C-H), 2958, 1728 (C=O), 1689, 1643 (C=C), 1438, 1371, 1280 (P=O), 1215 1178, 1051 (P-O), 1020, 966.

(1*R*,2*S*,5*S*)-3,3-Dicyano-5-vinylcyclopentane-1,2-dicarboxylic acid, 2-ethyl 1-methyl diester (12$_{maj}$) and (1*R*,2*S*,5*R*)-3,3-Dicyano-5-vinylcyclopentane-1,2-dicarboxylic acid, 2-ethyl 1-methyl diester (12$_{min}$)

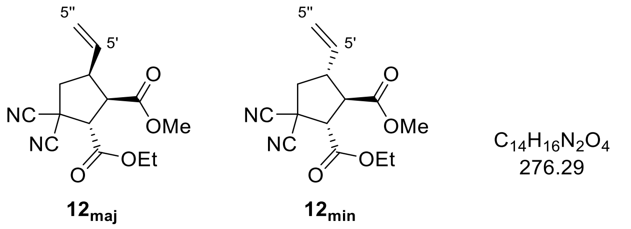

12$_{maj}$ **12$_{min}$**

Following **General Procedure E**, PNP ester **S12** (265 mg, 1.0 mmol, 1.0 eq), $Pd(PPh_3)_4$ (57.8 mg, 0.05 mmol, 5 mol%), (*R*)-BTM (50.5 mg, 0.2 mmol, 20 mol%), LiCl (0.5 M in THF, 0.6 mL, 0.3 mmol, 30 mol%) and VCP **2** (118 mg, 1.0 mmol, 1.0 eq) in THF : EtOAc 3:2 (5 mL) for 39 h followed by MeOH (1.0 mL) and DMAP (24.4 mg, 0.2 mmol, 20 mol%) for 24 h gave the crude product (88:7:5 dr). Purification by silica column chromatography (*n*-hexane : EtOAc 5:1 to 4:1, R_f 0.25 in *n*-hexane : EtOAc 4:1) gave the title compound as an inseparable mixture of diastereomers (91:1 dr) as a colourless oil (173 mg, 63%).

$[\alpha]_D^{20}$ +30.8 (*c* 1.03 in CHCl3).

HRMS (ESI$^+$): $C_{14}H_{16}N_2O_4Na$ [M+Na]$^+$ found 299.0991, requires 299.1002 (−3.8 ppm).

ν$_{max}$ (film, cm^{-1}): 3086 (C-H), 2985 (C-H), 2956, 2916, 2254 (C≡N), 1732 (C=O), 1643 (C=C), 1438, 1232, 1193, 1176, 1014, 931.

Data for major diastereoisomer ***12**$_{maj}$*

Chiral HPLC analysis, Chiralpak AD-H, (*n*-hexane : *i*-PrOH 95:5, flow rate 1.0 mLmin^{-1}, 254 nm, 40 °C) t_R (1*S*,2*R*,5*R*): 27.9 min, t_R (1*R*,2*S*,5*S*): 36.8 min, 5:95 er. (determined from PNP ester from crude reaction mixture (0.2 mL aliquot, filtered over silica) before addition of MeOH)

^{1}H NMR (500 MHz, $CDCl_3$) δ_H: 1.33 (3H, t, *J* 7.2, OCH_2CH_3), 2.41 (1H, dd, *J* 13.3, 11.0, C(4)H^AH^B), 2.71 (1H, dd, *J* 13.3, 6.5, C(4)H^AH^B), 3.28 – 3.38 (1H, m, C(5)*H*), 3.56 (1H, dd, *J* 10.6, 9.1, C(1)*H*), 3.69 (3H, s, OCH_3), 3.99 (1H, d, *J* 9.1, C(2)*H*), 4.23 – 4.37 (2H, m, OCH_2CH_3), 5.14 – 5.22 (2H, m, C(5'')H_2), 5.60 (1H, ddd, *J* 17.0, 10.2, 8.1, C(5')*H*).

^{13}C{^{1}H} NMR (126 MHz, $CDCl_3$) δ_C: 13.9 (OCH_2CH_3), 36.4 (*C*(3)), 43.0 (*C*(4)H_2), 43.3 (*C*(5)H), 47.8 (*C*(1)H), 52.5 (OCH_3), 55.7 (*C*(2)H), 62.9 (OCH_2CH_3), 113.9 (*C*N), 114.4 (*C*N), 119.3 (C(5'')H_2), 133.2 (*C*(5')H), 167.1 (*C*(2')=O), 171.2 (*C*(1')=O).

Data for minor diastereoisomer ***12**$_{min}$ *(selected)*

Chiral HPLC analysis, Chiralpak AD-H, (*n*-hexane : *i*-PrOH 95:5, flow rate 1.0 mLmin^{-1}, 254 nm, 40 °C) t_R (1*S*,2*R*,5*S*): 27.9 min, t_R (1*R*,2*S*,5*R*): 36.8 min, 25:75 er. (determined from PNP ester from crude reaction mixture (0.2 mL aliquot, filtered over silica) before addition of MeOH)

^{1}H NMR (500 MHz, $CDCl_3$) δ_H: 2.50 (1H, dd, *J* 14.0, 8.0, C(4)H^AH^B), 2.81 (1H, dd, *J* 14.0, 9.0, C(4)H^AH^B), 3.02 – 3.10 (1H, m, C(5)*H*), 3.17 (1H, app. t, *J* 10.1, C(1)*H*), 3.74 (3H, s, OCH_3), 3.83 (1H, d, *J* 10.5, C(2)*H*), 5.81 (1H, ddd, *J* 17.0, 10.3, 7.8, C(5')*H*).

^{13}C{^{1}H} NMR (126 MHz, $CDCl_3$) δ_C: 35.4 (*C*(3)), 43.5 (*C*(4)H_2), 45.7 (*C*(5)H), 50.8 (*C*(1)H), 52.7 (OCH_3), 56.1 (*C*(2)H), 114.1 (*C*N), 114.8 (*C*N), 118.2 (*C*(5'')H_2), 135.9 (*C*(5')H), 171.4 (*C*(1')=O).

(1*R*,2*S*,5*S*)-3,3-Dicyano-5-vinylcyclopentane-1,2-dicarboxylic acid, 2-(*t*-butyl) 1-methyl diester (13)

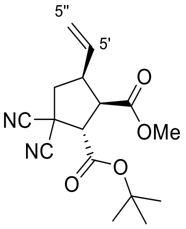

$C_{16}H_{20}N_2O_4$
304.35

Following **General Procedure E**, PNP ester **S28** (293 mg, 1.0 mmol, 1.0 eq), $Pd(PPh_3)_4$ (57.8 mg, 0.05 mmol, 5 mol%), (*R*)-BTM (50.5 mg, 0.2 mmol, 20 mol%), LiCl (0.5 M in THF, 0.6 mL, 0.3 mmol, 30 mol%) and VCP **2** (118 mg, 1.0 mmol, 1.0 eq) in THF : EtOAc 3:2 (5 mL) for 24 h followed by MeOH (1.0 mL) and DMAP (24.4 mg, 0.2 mmol, 20 mol%) for 24 h gave the crude product (88:12 dr). Purification by silica column chromatography (*n*-hexane : EtOAc 6:1, R_f 0.24) gave the title compound as a single diastereoisomer (>95:5 dr) as a white solid (169 mg, 56%). **m.p.** (EtOAc) 70 – 72 °C. $[\alpha]_D^{20}$ +37.9 (*c* 1.07 in $CHCl_3$). **Chiral HPLC** analysis**,** Chiralcel OD-H, (*n*-hexane : *i*-PrOH 96:4, flow rate 1.0 mLmin^{-1}, 254 nm, 40 °C) t_R (1*S*,2*R*,5*R*): 17.4 min, t_R (1*R*,2*S*,5*S*): 34.1 min, 8:92 er. (determined from PNP ester from crude reaction mixture (0.2 mL aliquot, filtered over silica) before addition of MeOH)

^{1}H NMR (500 MHz, $CDCl_3$) δ_H: 1.54 (9H, s, OC(C*H*$_3$)$_3$), 2.39 (1H, dd, *J* 13.2, 11.2, C(4)*H*^{A}H^B), 2.70 (1H, dd, *J* 13.2, 6.5, C(4)H^A*H*^{B}), 3.27 – 3.37 (1H, m, C(5)*H*), 3.54 (1H, dd, *J* 10.8, 9.0, C(1)*H*), 3.71 (3H, s, OC*H*$_3$), 3.93 (1H, d, *J* 9.0, C(2)*H*), 5.15 – 5.21 (2H, m, C(5'')*H*$_2$), 5.60 (1H, ddd, *J* 17.1, 10.1, 8.1, C(5')*H*).

^{13}C{^{1}H} NMR (126 MHz, $CDCl_3$) δ_C: 27.8 (OC(CH$_3$)$_3$), 36.7 (*C*(3)), 43.1 (*C*(4)H$_2$), 43.2 (*C*(5)H), 47.5 (*C*(1)H), 52.5 (OCH$_3$), 56.4 (*C*(2)H), 84.8 (OC(CH$_3$)$_3$), 114.1 (CN), 114.6 (CN), 119.2 (*C*(5'')H$_2$), 133.4 (*C*(5')H), 166.0 (*C*(2')=O), 171.4 (*C*(1')=O).

HRMS (ESI$^+$): $C_{16}H_{20}N_2O_4Na$ [M+Na]$^+$ found 327.1305, requires 327.1315 (−3.1 ppm).

ν$_{max}$ (film, cm^{-1}): 3003, 2987 (C-H), 2924, 1730 (C=O), 1641 (C=C), 1440 (C-H), 1381, 1369 (C-(CH$_3$)$_3$), 1251, 1232, 1174, 1155.

(1*R*,2*S*,5*S*)-3,3-Dicyano-2-(pyrrolidine-1-carbonyl)-5-vinylcyclopentane-1-carboxylic acid, methyl ester (14)

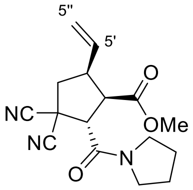

$C_{16}H_{19}N_3O_3$
301.35

Following **General Procedure E**, PNP ester **S13** (290 mg, 1.0 mmol, 1.0 eq), $Pd(PPh_3)_4$ (57.8 mg, 0.05 mmol, 5 mol%), (*R*)-BTM (50.5 mg, 0.2 mmol, 20 mol%), LiCl (0.5 M in THF, 0.6 mL, 0.3 mmol, 30 mol%) and VCP **2** (118 mg, 1.0 mmol, 1.0 eq) in THF : EtOAc 3:2 (5 mL) for 39 h followed by MeOH (1.0 mL) and DMAP (24.4 mg, 0.2 mmol, 20 mol%) for 24 h gave the crude product (79:21 dr). Purification by silica column chromatography (5 to 10% Et_2O in CH_2Cl_2, R_f 0.20 in 5% Et_2O in CH_2Cl_2) gave the title compound as a single diastereoisomer (>95:5 dr) as an off-white solid (192 mg, 64%). **m.p.** (Et_2O) 86 – 88 °C. $[\boldsymbol{\alpha}]_D^{20}$ +13.2 (*c* 1.02 in $CHCl_3$).

Chiral HPLC analysis**,** Chiralpak IA, (*n*-hexane : *i*-PrOH 95:5, flow rate 1.0 mLmin^{-1}, 211 nm, 30 °C) t_R (1*S*,2*R*,5*R*): 30.7 min, t_R (1*R*,2*S*,5*S*): 39.4 min, 9:91 er.

^{1}H NMR (500 MHz, $CDCl_3$) δ_H: 1.85 – 2.10 (4H, m, 2 × C*H*$_2$), 2.52 (1H, dd, *J* 13.4, 8.1, C(4)*H*^{A}H^B), 2.78 (1H, dd, *J* 13.4, 6.8, C(4)H^A*H*^{B}), 3.42 (1H, app. dtd, *J* 9.4, 8.1, 6.8, C(5)*H*), 3.48 – 3.59 (2H, m, NC*H*$_2$), 3.65 (3H, s, OC*H*$_3$), 3.67 – 3.72 (2H, m, NC*H*$_2$), 3.80 (1H, app. t, *J* 9.0 C(1)*H*), 3.99 (1H, d, *J* 8.8, C(2)*H*), 5.15 – 5.22 (2H, m, C(5'')*H*$_2$), 5.67 (1H, ddd, *J* 17.0, 10.2, 8.3, C(5')*H*).

^{13}C{^{1}H} NMR (126 MHz, $CDCl_3$) δ_C: 24.2 ($CH_{2(pyrr)}$), 26.0 ($CH_{2(pyrr)}$), 36.2 (*C*(3)), 44.2 (*C*(5)H), 44.3 (*C*(4)H$_2$), 46.7 (N*C*H$_2$), 47.2 (N*C*H$_2$), 50.8 (*C*(1)H), 52.3 (O*C*H$_3$), 53.3 (*C*(2)H), 113.8 (*C*N), 115.7 (*C*N), 119.1 (*C*(5'')H$_2$), 133.8 (*C*(5')H), 164.7 (*C*(2')=O), 171.3 (*C*(1')=O).

HRMS (ESI$^+$): $C_{16}H_{20}N_3O_3$ [M+H]$^+$ found 302.1490, requires 302.1499 (−3.0 ppm).

ν_{max} (film, cm^{-1}): 2985 (C-H), 2953, 2889, 1737 (C=O$_{ester}$), 1637 (C=O$_{amide}$), 1446 (C-H), 1313, 1247, 1207, 1082, 1026, 987, 935.

(1*R*,2*S*,5*S*)-3,3-Dicyano-2-(methoxy(methyl)carbamoyl)-5-vinyl cyclopentane-1-carboxylic acid, methyl ester (15)

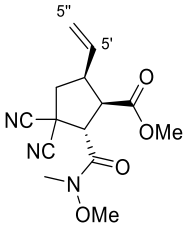

$C_{14}H_{17}N_3O_4$
291.31

Following **General Procedure E**, PNP ester **S30** (280 mg, 1.0 mmol, 1.0 eq), $Pd(PPh_3)_4$ (57.8 mg, 0.05 mmol, 5 mol%), (*R*)-BTM (50.5 mg, 0.2 mmol, 20 mol%), LiCl (0.5 M in THF, 0.6 mL, 0.3 mmol, 30 mol%) and VCP **2** (118 mg, 1.0 mmol, 1.0 eq) in THF : EtOAc 3:2 (5 mL) for 24 h followed by MeOH (1.0 mL) and DMAP (24.4 mg, 0.2 mmol, 20 mol%) for 24 h gave the crude product (>95:5 dr). Purification by silica column chromatography (0 to 5% Et_2O in CH_2Cl_2, R_f 0.37 in 5% Et_2O in CH_2Cl_2) gave the title compound as a single diastereoisomer (>95:5 dr) as a pale yellow solid (212 mg, 73%). **m.p.** (Et_2O) 72 – 75 °C. $[\alpha]_D^{20}$ +5.0 (*c* 1.14 in $CHCl_3$).

Chiral HPLC analysis**,** Chiralpak AD-H, (*n*-hexane : *i*-PrOH 95:5, flow rate 1.0 mLmin^{-1}, 211 nm, 40 °C) t_R (1*S*,2*R*,5*R*): 15.5 min, t_R (1*R*,2*S*,5*S*): 18.9 min, 8:92 er.

^{1}H NMR (500 MHz, $CDCl_3$) δ_H: 2.62 – 2.72 (2H, m, C(4)H_2), 3.27 – 3.37 (1H, m, C(5)*H*), 3.30 (3H, s, NCH_3), 3.69 (3H, s, OCH_3), 3.75 (1H, app. t, *J* 8.1, C(1)*H*), 3.83 (3H, s, N-OCH_3), 4.13 (1H, d, *J* 8.0, C(2)*H*), 5.18 – 5.25 (2H, m, C(5'')H_2), 5.75 (1H, ddd, *J* 17.0, 10.4, 8.0, C(5')*H*).

^{13}C{^{1}H} NMR (126 MHz, $CDCl_3$) δ_C: 33.0 (N*C*H_3), 35.9 (*C*(3)), 43.8 (*C*(4)H_2), 45.1 (*C*(5)H), 49.7 (*C*(1)H), 52.3 (O*C*H_3), 53.5 (*C*(2)H), 61.3 (N-O*C*H_3), 114.6 (*C*N), 116.3 (*C*N), 119.2 (*C*(5'')H_2), 133.2 (*C*(5')H), 167.4 (*C*(2')=O), 171.4 (*C*(1')=O).

HRMS (ESI$^+$): $C_{14}H_{17}N_3O_4Na$ [M+Na]$^+$ found 314.1104, requires 314.1111 (−2.3 ppm).

ν_{max} (film, cm^{-1}): 3088, 2985 (C-H), 2953, 2252 (C≡N), 1730 (C=O_{ester}), 1662 (C=O_{amide}), 1593, 1435, 1371, 1246, 1195, 1172, 991, 927.

(1*R*,2*S*,5*S*)-2-(Allyl(methyl)carbamoyl)-3,3-dicyano-5-vinylcyclopentane-1-carboxylic acid, methyl ester (16)

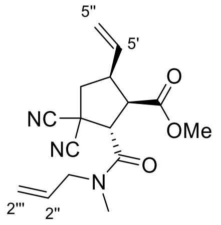

$C_{16}H_{19}N_3O_3$
301.35

Following **General Procedure E**, PNP ester **S33** (290 mg, 1.0 mmol, 1.0 eq), $Pd(PPh_3)_4$ (57.8 mg, 0.05 mmol, 5 mol%), (*R*)-BTM (50.5 mg, 0.2 mmol, 20 mol%), LiCl (0.5 M in THF, 0.6 mL, 0.3 mmol, 30 mol%) and VCP **2** (118 mg, 1.0 mmol, 1.0 eq) in THF : EtOAc 3:2 (5 mL) for 39 h followed by MeOH (1.0 mL) and DMAP (24.4 mg, 0.2 mmol, 20 mol%) for 24 h gave the crude product (93:7 dr). Purification by silica column chromatography (4 to 6% Et_2O in CH_2Cl_2, R_f 0.32 in 4% Et_2O in CH_2Cl_2) gave the title compound as a single diastereoisomer (>95:5 dr) as a colourless oil (226 mg, 75%) as a rotameric mixture (5:4). $[\alpha]_D^{20}$ +14.4 (*c* 1.12 in $CHCl_3$).

Chiral HPLC analysis**,** Chiralpak IA, (*n*-hexane : *i*-PrOH 96:4, flow rate 1.0 mLmin^{-1}, 211 nm, 40 °C) t_R (1*S*,2*R*,5*R*): 17.8 min, t_R (1*R*,2*S*,5*S*): 25.9 min, 7:93 er.

^{1}H NMR (500 MHz, $CDCl_3$) *major rotamer* δ_H: 2.49 – 2.58 (1H, m, C(4)*H*^{A}H^B), 2.73 – 2.81 (1H, m, C(4)H^A*H*^{B}), 3.15 (3H, s, NC*H*$_3$), 3.35 – 3.45 (1H, m, C(5)*H*), 3.63 (3H, s, OC*H*$_3$), 3.79 (1H, app. t, *J* 9.0, C(1)*H*), 3.98 (1H, ddt, *J* 15.1, 6.1, 1.5, NC*H*^{A}H^B), 4.02 – 4.18 (2H, m, C(2)*H*, NCH^A*H*^{B}), 5.11 – 5.25 (4H, m, C(2′′′)*H*$_2$, C(5′′)*H*$_2$), 5.60 – 5.77 (2H, m, C(2′′)*H*, C(5′)*H*). *minor rotamer* δ_H: 2.98 (3H, s, NC*H*$_3$), 3.62 (3H, s, OC*H*$_3$), 3.74 (1H, app. t, *J* 8.9, C(1)*H*), 5.27 (1H, dq, *J* 10.3, 1.3, C(2′′′)*H*^{A}H^B), 5.83 (1H, ddt, *J* 16.9, 10.3, 5.2, C(2′′)*H*).

^{13}C{^{1}H} NMR (126 MHz, $CDCl_3$) *major rotamer* δ_C: 35.3 (NCH_3), 36.2 (*C*(3)), 44.0 (*C*(5)H), 44.2 (*C*(4)H_2), 51.0 (NCH_2), 51.0 (*C*(2)H), 51.3 *C*(1)H, 52.3 (OCH_3), 113.7 (CN), 115.6 (*C*N), 118.0 (*C*(2′′)H_2), 119.1 (*C*(5′′)H_2), 131.8 (*C*(2′)H), 133.8 (*C*(5′)H), 166.4 (*C*(2′)=O), 171.2 (*C*(1′)=O). *minor rotamer* δ_C: 34.6 (NCH_3), 36.8 (*C*(3)), 44.3 (*C*(4)H_2), 44.4 (*C*(5)H), 50.9 (*C*(2)H), 51.5 (*C*(1)H), 52.3 (OCH_3), 52.6 (NCH_2), 113.9 (*C*N), 115.6 (*C*N), 118.1 (*C*(2′′)H_2), 119.2 (*C*(5′′)H_2), 132.0 (*C*(2′)H), 133.7 (*C*(5′)H), 167.0 (*C*(2′)=O), 171.1 (*C*(1′)=O).

HRMS (ESI$^+$): $C_{16}H_{19}N_3O_3Na$ $[M+Na]^+$ found 324.1309, requires 324.1319 (−2.9 ppm).

ν_{max} (film, cm^{-1}): 3084, 2985 (C-H), 2953, 2858, 2249 (C≡N), 1730 (C=O_{ester}), 1651 (C=O_{amide}), 1641, 1436, 1371, 1265, 1197, 1176, 991, 925.

(1*R*,2*S*,5*S*)-3,3-Dicyano-2-methyl-5-vinylcyclopentane-1-carboxylic acid, methyl ester (17)

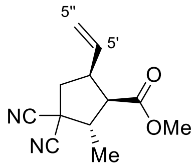

$C_{12}H_{14}N_2O_2$
218.26

Following **General Procedure E**, PNP ester **S15** (207 mg, 1.0 mmol, 1.0 eq), $Pd(PPh_3)_4$ (57.8 mg, 0.05 mmol, 5 mol%), (*R*)-BTM (50.5 mg, 0.2 mmol, 20 mol%), LiCl (0.5 M in THF, 0.6 mL, 0.3 mmol, 30 mol%) and VCP (473 mg, 4.0 mmol, 4.0 eq) in THF : EtOAc 3:2 (5 mL) for 96 h followed by MeOH (1.0 mL) and DMAP (24.4 mg, 0.2 mmol, 20 mol%) for 24 h gave the crude product (94:6 dr). Purification by silica chromatography (hexane : EtOAc 9:1, R_F 0.28) gave the title compound as a single diastereoisomer (>95:5 dr) as a colourless solid (75 mg, 34%). **m.p.** 54 – 56 °C. $[\alpha]_D^{20}$ +14.0 (*c* 2.0 in $CHCl_3$).

Chiral GC analysis Restek Rt-βDEXcst (length: 30 m, thickness: 0.25 mm, film thickness: 0.25 μm, carrier gas: He, linear velocity: 28 $cmsec^{-1}$, temperature: 60 to 220 °C (53 min)) t_R (1*R*,2*S*,5*S*): 38.3 min, t_R (1*S*,2*R*,5*R*): 38.7 min, 73:27 er.

^{1}H NMR (400 MHz, $CDCl_3$) δ_H: 1.39 (3H, d, *J* 6.6, CH_3), 2.33 (1H, dd, *J* 13.5, 9.7, C(4)H^AH^B), 2.74 (1H, dd, *J* 13.5, 7.3, C(4)H^AH^B), 2.91 (1H, t, *J* 10.4, C(1)*H*), 3.03 (1H, dq, *J* 10.4, 6.6, C(2)*H*), 3.24 – 3.35 (1H, m, C(5)*H*), 3.70 (3H, s, OCH_3), 5.10 – 5.20 (2H, m, C(5′′)H_2), 5.57 – 5.69 (1H, m, C(5′)*H*).

^{13}C{^{1}H} NMR (126 MHz, $CDCl_3$) δ_C: 14.8 (CH_3), 40.0 (*C*(3)), 42.8 (*C*(4)H_2), 43.2 (*C*(5)H), 47.2 (*C*(2), 52.2 (OCH_3), 52.8 (*C*(1)H), 114.0 (CN), 115.0 (CN), 118.7 (*C*(5′′)H_2), 134.7 (*C*(5′)*H*), 171.2 (*C*=O).

ν_{max} (film, cm^{-1}): 2955 (C-H), 1732 (C=O), 1437, 1377, 1283, 1196, 1177, 995, 926.

HRMS (EI^+): $C_{12}H_{14}O_2N_2$ $[M]^+$ found 218.1052, requires 218.1050 (+0.9 ppm).

(1*R*,2*S*,5*S*)-3,3-Dicyano-2-(2,5-dioxopyrrolidin-1-yl)-5-vinylcyclopentane-1-carboxylic acid, methyl ester (18)

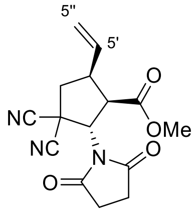

$C_{15}H_{15}N_3O_4$
301.30

Following **General Procedure E**, PNP ester **S36** (290 mg, 1.0 mmol, 1.0 eq), $Pd(PPh_3)_4$ (57.8 mg, 0.05 mmol, 5 mol%), (*R*)-BTM (101 mg, 0.4 mmol, 40 mol%), LiCl (0.5 M in THF, 0.6 mL, 0.3 mmol, 30 mol%) and VCP **2** (207 mg, 1.75 mmol, 1.75 eq) in THF : EtOAc 3:2 (5 mL) for 66 h followed by MeOH (1.0 mL) and DMAP (24.4 mg, 0.2 mmol, 20 mol%) for 24 h gave the crude product (68:32 dr). Purification by silica column chromatography (2 to 6% Et_2O in CH_2Cl_2, R_f 0.29 in 4% Et_2O in CH_2Cl_2) gave a yellow solid. Further purification by silica column chromatography (*n*-hexane : EtOAc 2:1 to 1:1, R_f 0.27 in *n*-hexane : EtOAc 1:1) gave the title compound as a single diastereoisomer (>95:5 dr) as a white solid (60 mg, 20%). **m.p.** (EtOAc) 150 – 153 °C. $[\boldsymbol{\alpha}]_D^{20}$ +39.3 (*c* 1.01 in $CHCl_3$).

$^{19}F\{^1H\}$ NMR (377 MHz, $CDCl_3$) δ_F (1*R*,2*S*,5*S*): −113.1, δ_F (1*S*,2*R*,5*R*): −113.3, 96:4 er.*

^1H NMR (500 MHz, $CDCl_3$) δ_H: 2.64 (1H, dd, *J* 13.6, 7.4, C(4)H^AH^B), 2.83 (4H, s, 2 × C$H_{2,(imide)}$), 2.86 (1H, dd, *J* 13.6, 6.5, C(4)H^AH^B), 3.60 (1H, app. p, *J* 7.7, C(5)*H*), 3.68 (3H, s, OCH_3), 4.34 (1H, app. t, *J* 8.1, C(1)*H*), 5.20 – 5.27 (2H, m, C(5'')H_2), 5.48 (1H, d, *J* 7.8, C(2)*H*), 5.77 (1H, ddd, *J* 17.0, 10.3, 8.1, C(5')*H*).

$^{13}C\{^1H\}$ NMR (126 MHz, $CDCl_3$) δ_C: 28.0 (2 × $CH_{2,(imide)}$), 38.4 (*C*(3)), 42.2 (*C*(4)H_2), 44.4 (*C*(5)H), 47.4 (*C*(1)H), 52.5 (OCH_3), 59.6 (*C*(2)H), 113.6 (*C*N), 114.8 (*C*N), 119.6 (*C*(5'')H_2), 132.9 (*C*(5')H), 170.6 (*C*(1')=O), 176.1 (2 × N*C*=O).

HRMS (ESI^+): $C_{15}H_{15}N_3O_4Na$ $[M+Na]^+$ found 324.0949, requires 324.0955 (−1.7 ppm).

ν_{max} (film, cm^{-1}): 2999, 2962 (C-H), 1780 ($C{=}O_{imide}$), 1728 ($C{=}O_{ester}$), 1708 ($C{=}O_{imide}$), 1645 (C=C), 1435, 1375, 1361, 1213, 1161, 1089, 956, 941.

* The enantiomeric ratio was determined by derivatisation with (1*S*)-1-(4-fluorophenyl)ethan-1-ol: An aliquot (0.2 mL) of the crude reaction mixture after 66 h was concentrated under reduced pressure and dissolved in 0.5 mL CH_2Cl_2. (1*S*)-1-(4-Fluorophenyl)ethan-1-ol (15 μL, 120 μmol, 3.0 eq) and DMAP (1.0 mg, 8.0 μmol, 0.2 eq) were added and the reaction mixture stirred at room temperature until ^1H NMR analysis

indicated full conversion of the intermediate PNP ester (ca. 48 h). The er was determined directly from the crude mixture by $^{19}F\{^1H\}$ NMR analysis.

(1*R*,2*S*,5*S*)-3,3-Dicyano-2-(2,5-dioxopyrrolidin-1-yl)-5-vinylcyclopentane-1-carboxylic acid, (*S*)-1-(4-fluorophenyl)ethyl ester (S49) and (1*S*,2*R*,5*R*)-3,3-Dicyano-2-(2,5-dioxopyrrolidin-1-yl)-5-vinylcyclopentane-1-carboxylic acid, (*S*)-1-(4-fluorophenyl)ethyl ester (S50)

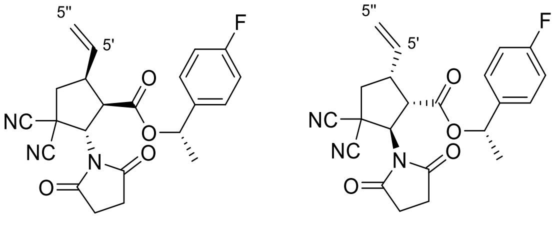

(1*R*,2*S*,5*S*)-**S49** (1*S*,2*R*,5*R*)-**S50**

$C_{22}H_{20}FN_3O_4$
409.42

Following **General Procedure E**, PNP ester **S36** (145 mg, 0.5 mmol, 1.0 eq), $Pd(PPh_3)_4$ (28.9 mg, 0.025 mmol, 5 mol%), (±)-TM·HCl (30.0 mg, 0.12 mmol, 25 mol%), *i*-Pr_2NEt (25.7 μL, 0.12 mmol, 25 mol%) and VCP **276** (118 mg, 1.0 mmol, 2.0 eq) in acetone (2.5 mL) for 84 h gave the crude product. Purification by silica column chromatography (1 to 5% Et_2O in CH_2Cl_2, R_f 0.37 in 5% Et_2O in CH_2Cl_2) gave a yellow gum, which was triturated with Et_2O to give the PNP ester product as an off-white solid (60 mg, 15%). The PNP ester was derivatised with (1*S*)-1-(4-fluorophenyl)ethan-1-ol (33.6 mg, 0.24 mmol, 1.7 eq) and DMAP (3.4 mg, 0.03 mmol, 0.2 eq) in CH_2Cl_2 (1.0 mL) and the conversion monitored by ^1H NMR analysis. After full consumption of PNP ester was observed (ca. 36 h), the reaction mixture was diluted with CH_2Cl_2 (5 mL) and washed with 1 M NaOH (2 × 5 mL) and brine (5 mL). The organic phase was dried over $MgSO_4$, filtered and the solvent was removed under reduces pressure. Purification of the crude product by silica column chromatography (*n*-hexane : EtOAc 4:1 to 1:1) gave the title compound as a 1:1 mixture of diastereoisomers as a colourless glass (26 mg, 46%).

HRMS (ESI^+): $C_{22}H_{20}FN_3O_4Na$ $[M+Na]^+$ found 432.1323, requires 432.1330 (−1.6 ppm).

ν_{max} ($CHCl_3$, cm^{-1}): 3084, 2985, 2939 (C-H), 2254 (C≡N), 1784 ($C=O_{imide}$), 1720 ($C=O_{ester}$), 1645 (C=C), 1606, 1512 ($C=C_{Ar}$), 1373, 1219, 1159, 1056, 906.

*Data for (1R,2S,5S)-**S49**:*

^{1}H NMR (500 MHz, $CDCl_3$) δ_H: 1.49 (3H, d, *J* 6.6, C*H*$_3$), 2.60 (1H, dd, *J* 13.6, 7.1, C(4)*H*^{A}H^B), 2.79 (4H, s, 2 × C*H*$_{2,(imide)}$), 2.81 – 2.84 (1H, m, C(4)H^A*H*^{B}), 3.51 – 3.60 (1H, m, C(5)*H*), 4.30 (1H, app. t, *J* 8.0, C(1)*H*), 4.94 (1H, d, *J* 10.3, C(5′′)*H*^{A}H^B), 5.04 (1H, d, *J* 17.0, C(5′′)H^A*H*^{B}), 5.44 (1H, d, *J* 7.9, C(2)*H*), 5.51 (1H, ddd, *J* 17.0, 10.3, 7.0, C(5′)*H*), 5.84 (1H, q, *J* 6.6, OC*H*), 6.99 – 7.07 (2H, m, Ar(3,5)*H*), 7.30 (2H, ddd, *J* 8.9, 5.3, 2.5, Ar(2,6)*H*).

^{13}C{^{1}H} NMR (126 MHz, $CDCl_3$) δ_C: 22.0 (*C*H$_3$), 27.9 (2 × *C*H$_{2,(imide)}$), 38.2 (*C*(3)), 42.3 (*C*(4)H$_2$), 44.6 (*C*(5)H), 47.4 (*C*(1)H), 59.6 (*C*(2)H), 73.5 (OCH), 113.5 (*C*N), 114.9 (*C*N), 115.5 (d, $^2J_{CF}$, 19.1, Ar*C*(3,5)H), 119.6 (*C*(5′′)H$_2$), 128.7 (d, $^3J_{CF}$, 8.3, Ar*C*(2,6)H), 132.4 (*C*(5′)H), 136.3 (d, $^4J_{CF}$, 3.2, Ar*C*(1)), 162.6 (d, $^1J_{CF}$, 247, Ar*C*(4)), 169.3 (*C*=O), 176.0 (2 × N*C*=O).

^{19}F{^{1}H} NMR (377 MHz, $CDCl_3$) δ_F: −113.1 (ArC(4)*F*).

*Data for (1S,2R,5R)-**S50**:*

^{1}H NMR (500 MHz, $CDCl_3$) δ_H: 1.53 (3H, d, *J* 6.7, C*H*$_3$), 2.64 (1H, dd, *J* 13.5, 7.3, C(4)*H*^{A}H^B), 2.79 (4H, s, 2 × C*H*$_{2,(imide)}$), 2.87 (1H, dd, *J* 13.5, 6.5, C(4)H^A*H*^{B}), 3.61 – 3.68 (1H, m, C(5)*H*), 4.31 (1H, app. t, *J* 8.0, C(1)*H*), 5.19 – 5.27 (2H, m, C(5′′)*H*$_2$), 5.47 (1H, d, *J* 7.8, C(2)*H*), 5.78 (1H, ddd, *J* 17.0, 10.4, 8.1, C(5′)*H*), 5.84 (1H, q, *J* 6.7, OC*H*), 6.99 – 7.07 (2H, m, Ar(3,5)*H*), 7.30 (2H, ddd, *J* 8.9, 5.3, 2.5, Ar(2,6)*H*).

^{13}C{^{1}H} NMR (126 MHz, $CDCl_3$) δ_C: 21.6 (*C*H$_3$), 27.9 (2 × *C*H$_{2,(imide)}$), 38.3 (*C*(3)), 42.4 (*C*(4)H$_2$), 44.6 (*C*(5)H), 47.4 (*C*(1)H), 59.6 (*C*(2)H), 73.5 (OCH), 113.5 (*C*N), 114.9 (*C*N), 115.4 (d, $^2J_{CF}$, 19.1, Ar*C*(3,5)H), 119.6 (*C*(5′′)H$_2$), 128.2 (d, $^3J_{CF}$, 8.2, Ar*C*(2,6)H), 132.9 (*C*(5′)H), 136.0 (d, $^4J_{CF}$, 3.1, Ar*C*(1)), 162.5 (d, $^1J_{CF}$, 247, Ar*C*(4)), 169.3 (*C*=O), 176.0 (2 × N*C*=O).

^{19}F{^{1}H} NMR (377 MHz, $CDCl_3$) δ_F: −113.3 (ArC(4)*F*).

(2*S*,3*R*,4*S*)-1',3'-Dioxo-2-(trifluoromethyl)-4-vinyl-1',3'-dihydrospiro [cyclopentane-1,2'-indene]-3-carboxylic acid, methyl ester (19$_{maj}$) and (2*S*,3*R*,4*R*)-1',3'-Dioxo-2-(trifluoro-methyl)-4-vinyl-1',3'-dihydrospiro [cyclopentane-1,2'-indene]-3-carboxylic acid, methyl ester (19$_{min}$)

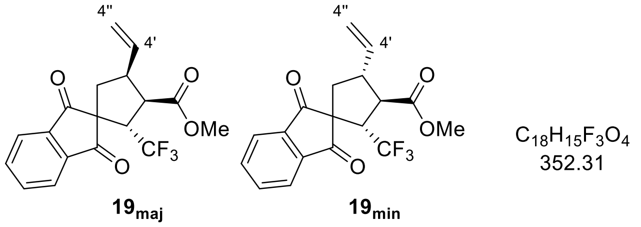

Following **General Procedure E**, PNP ester **3** (261 mg, 1.0 mmol, 1.0 eq), Pd(PPh$_3$)$_4$ (57.8 mg, 0.05 mmol, 5 mol%), (*R*)-BTM (50.5 mg, 0.2 mmol, 20 mol%), LiCl (0.5 M in THF, 0.6 mL, 0.3 mmol, 30 mol%) and VCP **S2** (198 mg, 1.0 mmol, 1.0 eq) in THF : EtOAc 3:2 (5 mL) for 48 h followed by MeOH (1.0 mL) and DMAP (48.8 mg, 0.4 mmol, 40 mol%) for 72 h gave the crude product (68:32 dr). Purification by silica column chromatography (*n*-hexane : EtOAc 5:1, R$_f$ 0.25) gave the title compound as an inseparable mixture of diastereomers (68:32 dr) as an off-white solid (142 mg, 40%). **m.p.** (EtOAc) 79 – 82 °C. $[\alpha]_D^{20}$ +34.9 (*c* 1.21 in CHCl$_3$).

HRMS (ESI$^+$): $C_{18}H_{15}F_3O_4Na$ [M+Na]$^+$ found 375.0806, requires 375.0815 (−2.3 ppm).

ν_{max} (film, cm^{-1}): 3082 (=C-H), 2956 (C-H), 2854, 1741 (C=O$_{ester}$), 1732 (C=O$_{ester}$), 1705 (C=O$_{ketone}$), 1641 (C=C), 1591 (C=C$_{Ar}$), 1438, 1400, 1350, 1271, 1215, 1157, 1116, 927.

*Data for major diastereoisomer **19$_{maj}$***

Chiral HPLC analysis, Chiralpak AD-H, (*n*-hexane : *i*-PrOH 99:1, flow rate 1.0 mLmin^{-1}, 211 nm, 30 °C) t$_R$ (2*S*,3*R*,4*S*): 26.3 min, Chiralpak AD-H, (*n*-hexane : *i*-PrOH 94:6, flow rate 1.0 mLmin^{-1}, 211 nm, 30 °C) t$_R$ (2*R*,3*S*,4*R*): 14.4 min, 89:11 er.

^{1}H NMR (500 MHz, CDCl$_3$) δ$_H$: 2.00 – 2.06 (1H, m, C(5)H^AH^B), 2.10 – 2.17 (1H, m, C(5)H^AH^B), 3.72 (3H, s, OCH_3), 3.70 – 3.81 (2H, m, C(4)*H*, C(3)*H*), 3.97 (1H, app. p, *J* 9.2, C(2)*H*), 5.04 – 5.19 (2H, m, C(4'')H_2), 5.69 (1H, ddd, *J* 16.9, 10.1, 8.3, C(4')*H*), 7.85 – 7.93 (2H, m, Ar(4',7')*H*), 7.98 – 8.04 (2H, m, Ar(5',6')*H*).

^{13}C{^{1}H} NMR (126 MHz, CDCl$_3$) δ$_C$: 40.8 (*C*(5)H$_2$), 44.1 (*C*(4)H), 52.1 (O*C*H$_3$), 52.2 (q, $^2J_{CF}$ 28.5, *C*(2)H), 52.3 (*C*(3)H) 58.9 (*C*(1)), 118.1 (*C*(4'')H$_2$), 123.8 (Ar*C*(4',7')H), 125.3 (q, $^1J_{CF}$ 280, *C*F$_3$), 135.8 (*C*(4')H), 136.2 (Ar*C*(5',6')H), 140.8 (Ar*C*(4a' or 7a')), 140.9 (Ar*C*(4a' or 7a')), 171.8 (*C*O$_2$CH$_3$), 199.0 (*C*(1' or 3')=O), 199.6 (*C*(1' or 3')=O).

^{19}**F{^{1}H} NMR** (377 MHz, $CDCl_3$) δ_F: −65.5 (C*F*$_3$).

Data for minor diastereoisomer ***19***$_{min}$ *(selected)*

Chiral HPLC analysis**,** Chiralpak AD-H, (*n*-hexane : *i*-PrOH 99:1, flow rate 1.0 mLmin^{-1}, 211 nm, 30 °C) t_R (2*S*,3*R*,4*R*): 28.4 min, Chiralpak AD-H, (*n*-hexane : *i*-PrOH 94:6, flow rate 1.0 mLmin^{-1}, 211 nm, 30 °C) t_R (2*R*,3*S*,4*S*): 15.5 min, 98:2 er.

^{1}**H NMR** (500 MHz, $CDCl_3$) δ_H: 1.98 (1H, dd, *J* 13.7, 8.9, C(5)*H*^{A}H^B), 2.30 (1H, dd, *J* 13.7, 9.1, C(5)H^A*H*^{B}), 3.21 (1H, app. p, *J* 9.1, C(4)*H*), 3.37 (1H, dd, *J* 11.9, 10.6, C(3)*H*), 3.63 – 3.70 (1H, m, C(2)*H*), 3.74 (3H, s, OC*H*$_3$), 5.85 (1H, ddd, *J* 17.0, 10.2, 8.3, C(4')*H*).

^{13}**C{^{1}H} NMR** (126 MHz, $CDCl_3$) δ_C: 40.1 (*C*(5)H$_2$), 47.1 (O*C*H$_3$), 47.8 (*C*(4)H), 50.7 (*C*(3)H), 53.8 (q, $^2J_{CF}$ 28.6, *C*(2)H), 57.6 (*C*(1)), 117.3 (*C*(4'')H$_2$), 123.7 (Ar*C*(4,7)H), 125.0 (q, $^1J_{CF}$ 279, *C*F$_3$), 136.3 (Ar*C*(5,6)H), 137.1 (*C*(4')H), 140.7 (Ar*C*(4a or 7a)), 141.4 (Ar*C*(4a or 7a)), 172.0 (*C*O$_2$CH$_3$), 199.8 (*C*(1' or 3')=O), 200.6 (*C*(1' or 3')=O).

^{19}**F{^{1}H} NMR** (377 MHz, $CDCl_3$) δ_F: −64.2 (C*F*$_3$).

(1*S*,2*S*,3*R*,4*S*)-1-Cyano-2-(trifluoromethyl)-4-vinylcyclopentane-1,3-dicarboxylic acid, dimethyl ester (20) and (1*R*,2*S*,3*R*,4*S*)-1-Cyano-2-(trifluoromethyl)-4-vinylcyclopentane-1,3-dicarboxylic acid, dimethyl ester (20)*

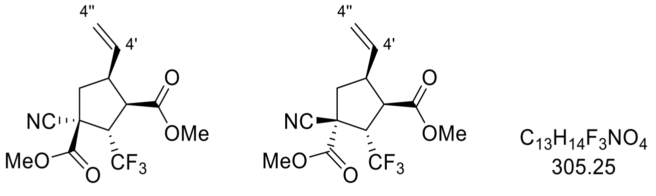

(1*S*,2*S*,3*R*,4*S*)-20 **(1*R*,2*S*,3*R*,4*S*)-20**

Following **General Procedure E**, PNP ester **3** (261 mg, 1.0 mmol, 1.0 eq), Pd(PPh$_3$)$_4$ (57.8 mg, 0.05 mmol, 5 mol%), (*R*)-BTM (50.5 mg, 0.2 mmol, 20 mol%), LiCl (0.5 M in THF, 0.6 mL, 0.3 mmol, 30 mol%) and VCP **S3** (151 mg, 1.0 mmol, 1.0 eq) in THF : EtOAc 3:2 (5 mL) for 48 h followed by MeOH (1.0 mL) and DMAP (24.4 mg, 0.2 mmol, 20 mol%) for 30 h gave the crude product (49:41:6:4 dr). Purification by silica column chromatography (*n*-hexane : EtOAc 5:1) allowed partial separation of the diastereoisomers to give:

20-A (R_f 0.28) as an inseparable mixture of diastereoisomers (92:8 dr) as a colourless oil (60 mg, 20%). $[\alpha]_D^{20}$ +29.0 (*c* 1.24 in $CHCl_3$). The er could not be determined.

HRMS (ESI$^+$) $C_{13}H_{15}F_3NO_4$ [M+H]$^+$ found 306.0943, requires 306.0947 (−0.5 ppm).

ν_{max} (film, cm^{-1}) 3020 (C=CH$_2$), 2962 (C-H), 2848, 1735 (C=O), 1645 (C=C), 1442, 1398, 1338, 1280, 1238, 1215, 1166, 1124, 1066, 1001, 974, 945.

Data for major diastereoisomer ***20-A$_{maj}$***

^{1}H NMR (500 MHz, CDCl$_3$) δ_H: 2.22 (1H, dd, *J* 13.6, 10.4, C(5)H^AH^B), 2.63 (1H, dd, *J* 13.6, 7.5, C(5)H^AH^B), 3.48 (1H, app. t, *J* 10.8, C(3)*H*), 3.62 – 3.72 (1H, m, C(4)*H*), 3.68 (3H, s, C(3′)OC*H*$_3$), 3.84 (3H, s, C(1′)OC*H*$_3$), 3.82 – 3.91 (1H, m, C(2)*H*), 5.08 – 5.19 (2H, m, C(4′′)*H*$_2$), 5.58 (1H, ddd, *J* 16.9, 10.1, 8.9, C(4′)*H*).

^{13}C{^{1}H} NMR (126 MHz, CDCl$_3$) δ_C: 42.8 (*C*(5)H$_2$), 43.8 (*C*(4)H), 46.9 (*C*(3)H), 47.1 (*C*(1)), 52.4 (C(3′)OCH$_3$), 54.1 (C(1′)OCH$_3$), 54.9 (q, $^2J_{CF}$ 28.9, *C*(2)H), 117.4 (CN), 119.0 (*C*(4′′)H$_2$), 124.4 (q, $^1J_{CF}$ 279, *C*F$_3$), 135.0 (*C*(4′)H), 166.6 (*C*(1′)=O), 170.9 (*C*(3′)=O).

^{19}F{^{1}H} NMR (377 MHz, CDCl$_3$) δ_F: −67.2 (C*F*$_3$).

Data for minor diastereoisomer ***20-A$_{min}$*** *(selected)*

^{1}H NMR (500 MHz, CDCl$_3$) δ_H: 2.50 (1H, dd, *J* 14.0, 9.6, C(5)H^AH^B), 3.05 (1H, app. t, *J* 10.3, C(3)*H*), 3.07 – 3.16 (1H, m, C(4)*H*), 3.73 (3H, s, C(3′)OC*H*$_3$), 5.82 (1H, ddd, *J* 17.0, 10.2, 7.8, C(4′)*H*).

^{13}C{^{1}H} NMR (126 MHz, CDCl$_3$) δ_C: 41.6 (*C*(5)H$_2$), 46.8 (*C*(4)H), 49.6 (*C*(3)H), 52.7 (C(3′)OCH$_3$), 54.3 (C(1′)OCH$_3$), 55.7 (q, $^2J_{CF}$ 29.0, *C*(2)H), 118.0 (*C*(4′′)H$_2$), 118.2 (CN), 124.2 (q, $^1J_{CF}$ 280, *C*F$_3$), 136.0 (*C*(4′)H), 166.5 (*C*(1′)=O), 171.3 (*C*(3′)=O).

^{19}F{^{1}H} NMR (377 MHz, CDCl$_3$) δ_F: −66.8 (C*F*$_3$).

20-B (R_f 0.22) as an inseparable mixture of diastereoisomers (84:11:5 dr) as a colourless oil (74 mg, 24%). $[\alpha]_D^{20}$ +30.5 (*c* 1.06 in CHCl$_3$).

HRMS (ESI$^+$) $C_{13}H_{14}F_3NO_4Na$ [M+Na]$^+$ found 328.0766, requires 328.0767 (−0.1 ppm).

ν_{max} (film, cm^{-1}) 3086 (C=CH$_2$), 2958 (C-H), 2852, 2249 (C≡N), 1743 (C=O), 1643 (C=C), 1438, 1402, 1359, 1224, 1166, 1126, 1082, 972, 929.

Data for major diastereoisomer ***20-B$_{maj}$***

Chiral HPLC analysis**,** Chiralpak AD-H, (*n*-hexane : *i*-PrOH 95:5, flow rate 1.0 mLmin^{-1}, 254 nm, 40 °C) t_R (2*S*,3*R*,4*S*): 15.5 min, t_R (2*R*,3*S*,4*R*): 25.7 min, 97:3 er. (determined from PNP ester from crude reaction mixture (0.2 mL aliquot, filtered over silica) before addition of MeOH)

^{1}H NMR (500 MHz, $CDCl_3$) δ_H: 2.29 – 2.37 (1H, m, C(5)H^AH^B), 2.46 – 2.53 (1H, m, C(5)H^AH^B), 3.36 – 3.51 (2H, m, C(3)*H*, C(4)*H*), 3.70 (3H, s, C(3′)OCH_3), 3.90 (3H, s, C(1′)OCH_3), 4.05 (1H, app. p, *J* 8.2, C(2)*H*), 5.10 – 5.22 (2H, m, C(4′′)H_2), 5.57 – 5.66 (1H, m, C(4′)*H*).

^{13}C{^{1}H} NMR (126 MHz, $CDCl_3$) δ_C: 43.2 (*C*(5)H_2), 44.6 (*C*(4)H), 46.3 (*C*(3)H), 48.7 (*C*(1)), 52.4 (C(3′)OCH_3), 53.4 (q, $^{2}J_{CF}$ 28.9, *C*(2)H), 54.5 (C(1′)OCH_3), 115.9 (CN), 119.2 (*C*(4′′)H_2), 124.6 (q, $^{1}J_{CF}$ 279, *C*F_3), 133.3 (*C*(4′)H), 166.7 (*C*(1′)=O), 171.1 (*C*(3′)=O).

^{19}F{^{1}H} NMR (377 MHz, $CDCl_3$) δ_F: −67.5 (C*F*$_3$).

*Data for minor diastereoisomer **20-B$_{min}$** (selected)*

^{1}H NMR (500 MHz, $CDCl_3$) δ_H: 2.67 – 2.74 (1H, m, C(5)H^AH^B), 2.96 – 3.03 (2H, m, C(3)*H*, C(4)*H*), 3.74 (3H, s, C(3′)OCH_3), 5.77 (1H, ddd, *J* 17.8, 10.3, 5.4, C(4′)*H*).

^{13}C{^{1}H} NMR (126 MHz, $CDCl_3$) δ_C: 43.5 (*C*(5)H_2), 46.5 (*C*(4)H), 50.1 (*C*(3)H), 52.7 (C(3′)OCH_3), 53.5 (q, $^{2}J_{CF}$ 28.1, *C*(2)H), 54.7 (C(1′)OCH_3), 116.1 (CN), 118.1 (*C*(4′′)H_2), 124.5 (q, $^{1}J_{CF}$ 279, CF_3), 135.8 (*C*(4′)H), 167.5 (*C*(1′)=O), 171.2 (*C*(3′)=O).

^{19}F{^{1}H} NMR (377 MHz, $CDCl_3$) δ_F: −67.0 (C*F*$_3$).

**The unambiguous assignment of the C(1) centre was not possible with the configuration for the isolated diastereoisomers A and B at C(1) remaining unknown.*

(2*S*,3*R*,4*S*)-2-(Trifluoromethyl)-4-vinylcyclopentane-1,1,3-tricarboxylic acid, 3-methyl 1,1-bis(2,2,2-trifluoroethyl) ester (21$_{maj}$) and (2*S*,3*R*,4*R*)-2-(Trifluoromethyl)-4-vinylcyclo-pentane-1,1,3-tricarboxylic acid, 3-methyl 1,1-bis(2,2,2-trifluoroethyl) ester (21$_{min}$)

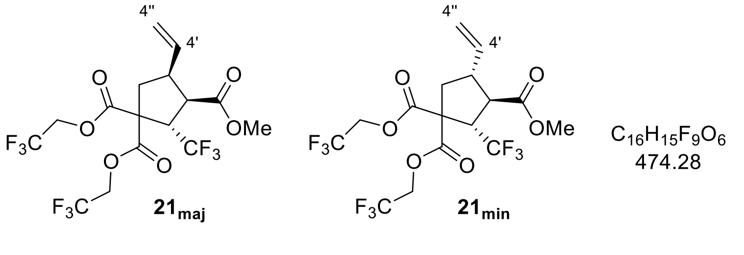

Following **General Procedure E**, PNP ester **3** (261 mg, 1.0 mmol, 1.0 eq), $Pd(PPh_3)_4$ (57.8 mg, 0.05 mmol, 5 mol%), (*R*)-BTM (50.5 mg, 0.2 mmol, 20 mol%), LiCl (0.5 M in THF, 0.6 mL, 0.3 mmol, 30 mol%) and VCP **S5** (320 mg, 1.0 mmol, 1.0 eq) in THF : EtOAc 3:2 (5 mL) for 96 h followed by MeOH (1.0 mL) and DMAP (24.4 mg, 0.2 mmol, 20 mol%) for 24 h gave the crude product (75:19:6 dr). Purification by silica column chromatography

(15% Et_2O in *n*-hexane, R_f 0.27) gave the title compound as an inseparable mixture of diastereomers (80:15:5 dr) as a pale yellow oil (240 mg, 51%). $[\alpha]_D^{20}$ +39.4 (*c* 1.23 in $CHCl_3$).

HRMS (ESI^+): $C_{16}H_{15}F_9O_6Na$ $[M+Na]^+$ found 497.0606, requires 497.0617 (−2.2 ppm).

ν_{max} (film, cm^{-1}): 29800 (C-H), 2960, 1755 ($C{=}O_{ester}$), 1643 (C=C), 1440, 1413, 1278, 1220, 1159 (C-F), 1122, 983, 974, 964.

*Data for major diastereoisomer **21**$_{maj}$*

Chiral GC analysis Restek Rt-βDEXcst (length: 30 m, thickness: 0.25 mm, film thickness: 0.25 μm, carrier gas: He, linear velocity: 28 $cmsec^{-1}$, temperature: 110 °C (95 min)) t_R (2*R*,3*S*,4*R*): 85.4 min, t_R (2*S*,3*R*,4*S*): 88.3 min, 3:97 er.

^{1}H NMR (500 MHz, $CDCl_3$) δ_H: 2.07 (1H, dd, *J* 13.4, 10.6, C(5)H^AH^B), 2.68 (1H, dd, *J* 13.3, 6.7, C(5)H^AH^B), 3.33 (1H, dd, *J* 10.3, 8.2, C(3)*H*), 3.50 – 3.62 (1H, m, C(4)*H*), 3.68 (3H, s, OCH_3), 4.27 (1H, app. p, *J* 8.9, C(2)*H*), 4.37 (1H, dq, *J* 12.5, 8.2, C(1′a)OCH^AH^B), 4.49 (1H, dq, *J* 12.6, 8.2, C(1′b)OCH^AH^B), 4.57 – 4.69 (2H, m, C(1′a)OCH^AH^B, C(1′b)OCH^AH^B), 5.10 (1H, d, *J* 10.2, C(4′′)H^AH^B), 5.14 (1H, d, *J* 17.0, C(4′′)H^AH^B), 5.57 (1H, ddd, *J* 17.0, 10.2, 8.3, C(4′)*H*).

^{13}C{^{1}H} NMR (126 MHz, $CDCl_3$) δ_C: 41.2 (*C*(5)H_2), 44.4 (*C*(4)H), 48.0 (*C*(3)H), 51.7 (q, $^2J_{CF}$ 28.9, *C*(2)H), 52.2 (OCH_3), 59.8 (*C*(1)), 61.6 (q, $^2J_{CF}$ 37.4, C(1′a)OCH_2), 61.6 (q, $^2J_{CF}$ 37.4, C(1′b)OCH_2), 118.3 (*C*(4′′)H_2), 122.3 (q, $^1J_{CF}$ 278, CH_2CF_3), 122.3 (q, $^1J_{CF}$ 278, CH_2CF_3), 125.1 (q, $^1J_{CF}$ 279, *C*(2′)F_3), 134.8 (*C*(4′)H), 167.1 (*C*(1′b)=O), 167.4 (*C*(1′a)=O), 171.6 (*C*(3′)=O).

^{19}F{^{1}H} NMR (377 MHz, $CDCl_3$) δ_F: −73.9 (CH_2CF_3), −73.7 (CH_2CF_3), −66.8 (C(2′)F_3).

*Data for minor diastereoisomer **21**$_{min}$ (selected)*

Chiral GC analysis Restek Rt-βDEXcst (length: 30 m, thickness: 0.25 mm, film thickness: 0.25 μm, carrier gas: He, linear velocity: 28 $cmsec^{-1}$, temperature: 110 °C (95 min)) t_R (2*R*,3*S*,4*S*): 79.2 min, t_R (2*S*,3*R*,4*R*): 81.9 min, 2:98 er.

^{1}H NMR (500 MHz, $CDCl_3$) δ_H: 2.45 (1H, dd, *J* 13.8, 7.2, C(5)H^AH^B), 2.60 – 2.64 (1H, m, C(5)H^AH^B), 2.73 – 2.82 (1H, m, C(4)*H*), 2.89 (1H, dd, *J* 10.1, 8.6, C(3)*H*), 3.73 (3H, s, OCH_3), 4.14 (1H, app. p, *J* 9.2, C(2)*H*), 5.77 (1H, ddd, *J* 17.4, 10.2, 7.5, C(5′)*H*).

^{13}C{^{1}H} NMR (126 MHz, $CDCl_3$) δ_C: 40.1 (*C*(5)H_2), 44.9 (*C*(4)H), 50.2 (*C*(3)H), 52.5 (OCH_3), 117.5 (*C*(4′′)H_2), 136.3 (*C*(4′)H), 166.0 (*C*(1′)=O), 167.9 (*C*(1′)=O), 171.5 (*C*(3′)=O).

^{19}F{^{1}H} NMR (377 MHz, $CDCl_3$) δ_F: −73.8 (CH_2CF_3), −73.7 (CH_2CF_3), −66.9 (C(2′)F_3).

11 Gram scale catalytic reaction

(1*R*,2*S*,5*S*)-3,3-Dicyano-2-(trifluoromethyl)-5-vinylcyclopentane-1-carboxylic acid, 4-nitrophenyl ester (4_{maj})

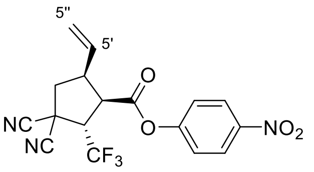

$C_{17}H_{12}F_3N_3O_4$
379.30

Following **General Procedure E**, PNP ester **3** (1.31 g, 5.0 mmol, 1.0 eq), $Pd(PPh_3)_4$ (289 mg, 0.25 mmol, 5 mol%), (*R*)-BTM (252 mg, 1.0 mmol, 20 mol%), LiCl (0.5 M in THF, 3.0 mL, 1.5 mmol, 30 mol%) and VCP **2** (591 mg, 5.0 mmol, 1.0 eq) in THF : EtOAc 3:2 (25 mL) for 24 h gave the crude product (95:5 dr). Purification by silica column chromatography (*n*-hexane : EtOAc 4:1, R_f 0.23) gave a pale yellow oil, which was triturated with Et_2O to afford the title compound as an inseparable mixture of diastereomers (95:5 dr) as an off-white solid (1.49 g, 78%).

chiral HPLC analysis Chiralcel OD-H (hexane : *i*-PrOH 97:3, flow rate 1.0 mlmin^{-1}, 254 nm, 40 °C) *major diastereoisomer:* t_R (1*S*,2*R*,5*R*): 30.1 min, t_R (1*R*,2*S*,5*S*): 32.3 min, 7:93 er. *minor diastereoisomer:* t_R (1*S*,2*R*,5*S*): 40.4 min, t_R (1*R*,2*S*,5*R*): 45.2 min, 5:95 er.

12 Product Derivatisations

Preliminary Investigations

Product derivatisations were initially also trialled with BnOH and $BnNH_2$ on racemic products. For the derivatisation with $BnNH_2$, a mixture of products (amide and cyclised product S) was obtained, which unfortunately could not be optimised to bias the cyclised product.

***rel*-(1*S*,2*R*,5*R*)-3,3-Dicyano-2-(trifluoromethyl)-5-vinylcyclopentane-1-carboxylic acid, benzyl ester (S51)**

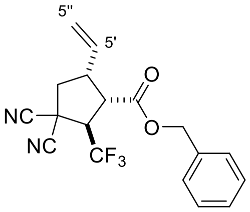

$C_{18}H_{15}F_3N_2O_2$
348.33

Following **General Procedure E**, PNP ester **3** (65.3 mg, 0.25 mmol, 1.0 eq), $Pd(PPh_3)_4$ (14.4 mg, 0.013 mmol, 5 mol%), (±)-TM·HCl (6.0 mg, 0.025 mmol, 10 mol%), *i*-Pr_2NEt (4.3 μL, 0.025 mmol, 10 mol%) and VCP **2** (29.5 mg, 0.25 mmol, 1.0 eq) in acetone (1.3 mL) for 24 h followed by BnOH (0.13, 1.25 mmol, 5.0 eq) and DMAP (6.1 mg, 0.05 mmol, 20 mol%) for 24 h gave the crude product (95:5 dr). Purification by silica column chromatography (petrol : EtOAc 6:1, R_f 0.29) gave the title compound as an inseparable mixture of diastereoisomers (95:5 dr) as a white solid (49 mg, 56%).

m.p. (*n*-hexane) 59 – 61 °C

HRMS (ESI$^-$) $C_{18}H_{14}F_3N_2O_2$ $[M-H]^-$ found 347.1010, requires 347.1012 (−0.8 ppm).

ν_{max} (film, cm^{-1}) 3035 (=CH_2), 2956 (C-H), 1724 (C=O), 1647 (C=C), 1500 (C=C_{Ar}), 1456, 1402, 1265, 1170 (C-F), 1128, 937.

Data for major diastereoisomer ***S51**$_{maj}$*

^{1}H NMR (500 MHz, $CDCl_3$) δ_H: 2.48 (1H, app. t, *J* 12.3, C(4)H^AH^B), 2.72 (1H, dd, *J* 13.1, 5.1, C(4)H^AH^B), 3.35 – 3.47 (2H, m, C(1)*H*, C(5)*H*), 3.89 (1H, app. p, *J* 7.6, C(2)*H*), 5.09 (1H, d, *J* 10.3, C(5′′)H^AH^B), 5.13 (1H, d, *J* 12.0, OCH^AH^B), 5.15 – 5.21 (2H, m, C(5′′)H^AH^B, OCH^AH^B), 5.50 (1H, ddd, *J* 17.2, 10.2, 7.3, C(5′)*H*), 7.30 – 7.41 (5H, m, 5 × Ar*H*).

^{13}C{^{1}H} NMR (126 MHz, $CDCl_3$) δ_C: 34.3 (*C*(3)), 43.4 (*C*(4)H_2), 44.4 (*C*(5)H), 45.8 (*C*(1)H), 55.1 (q, $^2J_{CF}$ 29.6, *C*(2)H), 68.1 (OCH_2), 112.2 (CN), 113.3 (CN), 120.3 (*C*(5′′)H_2), 123.8 (q, $^1J_{CF}$ 280, CF_3), 128.7 (Ar*C*(3,5)H), 128.8 (Ar*C*(2,6)H), 128.9 (Ar*C*(4)H), 131.8 (*C*(5′)H), 134.4 (Ar*C*(1)), 170.1 (*C*=O).

^{19}F{^{1}H} NMR (377 MHz, $CDCl_3$) δ_F: −67.5 (CF_3).

Data for minor diastereoisomer ***S52**$_{min}$* *(selected)*

^{1}H NMR (500 MHz, $CDCl_3$) δ_H: 2.85 (1H, dd, *J* 13.7, 7.4, C(4)H^AH^B), 3.05 (1H, app. t, *J* 9.6, C(1)*H*), 3.08 – 3.15 (1H, m, C(5)*H*), 3.79 (1H, app. p, *J* 8.3, C(2)*H*), 5.76 (1H, ddd, *J* 17.4, 10.3, 7.6, C(5′)*H*).

^{13}C{^{1}H} NMR (126 MHz, $CDCl_3$) δ_C: 44.5 (*C*(4)H_2), 46.6 (*C*(5)H), 49.7 (*C*(1)H), 119.4 (*C*(5′′)H_2), 134.2 (*C*(5′)H).

^{19}F{^{1}H} NMR (377 MHz, $CDCl_3$) δ_F: −67.2 (CF_3).

rel-(1*S*,2*R*,5*R*)-*N*-Benzyl-3,3-dicyano-2-(trifluoromethyl)-5-vinylcyclopentane-1-carboxamide (S52) and *rel*-(1*R*,5*S*,6*R*,8*S*)-3-Benzyl-2-imino-4-oxo-8-(trifluoromethyl)-6-vinyl-3-azabicyclo[3.2.1]octane-1-carbonitrile (S53)

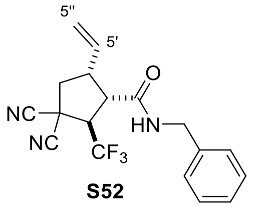

S52
$C_{18}H_{16}F_3N_3O$
347.34

and

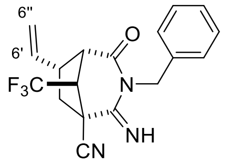

S53
$C_{18}H_{16}F_3N_3O$
347.34

Following **General Procedure J**, PNP ester **3** (65.3 mg, 0.25 mmol, 1.0 eq), $Pd(PPh_3)_4$ (14.4 mg, 0.013 mmol, 5 mol%), (±)-TM·HCl (6.0 mg, 0.025 mmol, 10 mol%), *i*-Pr_2NEt (4.3 μL, 0.025 mmol, 10 mol%) and VCP **2** (29.5 mg, 0.25 mmol, 1.0 eq) in acetone (1.3 mL) for 24 h followed by $BnNH_2$ (0.13, 1.25 mmol, 5.0 eq) for 4 h gave the crude product as a mixture of 3 isomers (**S52** : **S53$_{maj}$** : **S53$_{min}$** 67:30:3). Purification by silica column chromatography (*n*-hexane : EtOAc 4:1) gave:

S52 (R_f 0.22) as a single diastereoisomer as a colourless oil (43 mg, 49%).

^{1}H NMR (500 MHz, $CDCl_3$) δ_H: 2.51 – 2.61 (1H, m, C(4)H^AH^B), 2.66 (1H, dd, *J* 12.8, 5.8, C(4)H^AH^B), 3.03 (1H, dd, *J* 10.3, 7.4, C(1)*H*), 3.24 – 3.35 (1H, m, C(5)*H*), 4.08 (1H, app. p, *J* 7.9, C(2)H), 4.37 – 4.50 (2H, m, NCH_2), 5.17 (1H, d, *J* 10.1, C(5'')H^AH^B), 5.24 (1H, d, *J* 17.1, C(5'')H^AH^B), 5.59 (1H, ddd, *J* 17.1, 10.1, 8.7, C(5')*H*), 5.80 – 5.96 (1H, m, N*H*), 7.21 – 7.26 (2H, m, Ar(2,6)*H*), 7.28 – 7.38 (3H, m, Ar(3,4,5)*H*).

^{13}C{^{1}H} NMR (126 MHz, $CDCl_3$) δ_C: 34.4 (*C*(3)), 44.1 (*C*(4)H_2), 44.3 (NCH_2), 45.3 (*C*(5)H), 47.2 (C(1)H), 55.1 (q, $^2J_{CF}$ 29.4, *C*(2)H), 112.6 (CN), 113.3 (CN), 120.6 (C(5'')H_2), 124.1 (q, $^1J_{CF}$ 279, *C*F_3), 127.9 (Ar*C*(2,6)H), 128.0 (Ar*C*(4)H), 128.8 (Ar*C*(3,5)H), 132.7 (*C*(5')H), 136.9 (Ar*C*(1)), 168.3 (*C*=O).

^{19}F{^{1}H} NMR (377 MHz, $CDCl_3$) δ_F: −67.3 (C*F*$_3$).

HRMS (ESI$^+$) $C_{18}H_{16}F_3N_3ONa$ [M+Na]$^+$ found 370.1126, requires 370.1138 (−3.1 ppm).

ν_{max} (film, cm^{-1}) 3304 (N-H), 3088, 3024 (C=CH), 2943 (C-H), 1651 (C=O), 1608, 1541 (C=O), 1454, 1400, 1269, 1174 (C-F), 1128, 1029, 933.

S53 (R_f 0.42) as an inseparable mixture of diastereoisomers (92:8 dr) as a white solid (21 mg, 24%). **m.p.** ($CHCl_3$) 111 – 114 °C.

HRMS (ESI$^+$) $C_{18}H_{17}F_3N_3O$ [M+H]$^+$ found 348.1308, requires 348.1318 (−2.8 ppm).

ν_{max} (film, cm^{-1}) 3302 (N-H), 3068 (C=CH), 2985 (C-H), 2850, 2256 (C≡N), 1695 (C=N), 1622 (C=O), 1581, 1494 (C=C$_{Ar}$), 1438, 1386, 1280, 1261, 1170, 1126, 1080, 935, 925, 900.

*Data for major diastereoisomer **S53**$_{maj}$*

^{1}H NMR (500 MHz, $CDCl_3$) δ_H: 2.09 (1H, dd, *J* 14.4, 6.4, C(7)*H*^{A}H^B), 2.88 (1H, dd, *J* 14.4, 10.9, C(7)H^A*H*^{B}), 3.17 (1H, q, *J* 9.0, C(8)*H*), 3.32 – 3.41 (1H, m, C(6)*H*), 3.42 – 3.47 (1H, m, C(5)*H*), 5.04 – 5.16 (4H, m, NC*H*$_2$, C(6′′)*H*$_2$), 5.41 (1H, ddd, *J* 17.4, 10.3, 7.5, C(6′)*H*), 7.26 – 7.32 (3H, m, Ar(3,4,5)*H*), 7.37 – 7.44 (2H, m, Ar(2,6)*H*), 8.71 (1H, s, N*H*).

^{13}C{^{1}H} NMR (126 MHz, $CDCl_3$) δ_C: 39.1 (*C*(7)), 42.7 (*C*(6)H), 44.5 (NCH$_2$), 48.0 (*C*(1)), 49.2 (q, $^2J_{CF}$ 29.9, *C*(8)H), 50.3 (*C*(5)H), 115.1 (CN), 119.2 (C(6′′)H$_2$), 123.9 (q, $^1J_{CF}$ 280, *C*F$_3$), 127.8 (Ar*C*(4)H), 128.4 (Ar*C*(3,5)H), 129.1 (Ar*C*(2,6)H), 133.5 (*C*(6′)H), 136.5 (Ar*C*(1)), 159.2 (*C*=N), 168.2 (*C*=O).

^{19}F{^{1}H} NMR (377 MHz, $CDCl_3$) δ_F: −65.9 (C*F*$_3$).

*Data for minor diastereoisomer **S53**$_{min}$ (selected)*

^{1}H NMR (500 MHz, $CDCl_3$) δ_H: 2.26 (1H, dd, *J* 14.4, 6.4, C(7)*H*^{A}H^B), 2.96 – 3.04 (1H, m, C(7)H^A*H*^{B}), 3.26 (1H, q, *J* 9.2, C(8)*H*), 3.54 – 3.59 (1H, m, C(5)*H*), 4.83 (1H, d, *J* 15.8, NC*H*^{A}H^B), 4.95 (1H, d, *J* 15.8, NCH^A*H*^{B}), 5.04 – 5.16 (2H, m, C(6′′)*H*$_2$), 5.64 (1H, ddd, *J* 17.2, 10.3, 6.8, C(6′)*H*), 7.90 (1H, s, N*H*).

^{13}C{^{1}H} NMR (126 MHz, $CDCl_3$) δ_C: 42.4, 44.1 (NCH$_2$), 126.9, 128.5, 129.3.

^{19}F{^{1}H} NMR (377 MHz, $CDCl_3$) δ_F: −65.6 (C*F*$_3$).

Catalysis Product Derivatisations

(1*R*,2*S*,5*S*)-3,3-Dicyano-5-((*E*)-styryl)-2-(trifluoromethyl)cyclopentane-1-carboxylic acid, methyl ester (26)

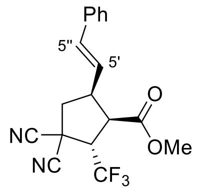

$C_{18}H_{15}F_3N_2O_2$
348.33

Following the procedure reported by Yao and co-workers,[39] a pressure tube was charged with methyl ester **8** (136 mg, 0.5 mmol, 1.0 eq), $Pd(OAc)_2$ (5.6 mg, 0.025 mmol, 5 mol%) and K_2CO_3 (104 mg, 0.75 mmol, 1.5 eq) and evacuated and flushed with N_2 three times. Acetone (5.0 mL, purged with Ar for 30 min) and iodobenzene (0.08 mL, 0.75 mmol, 1.5 eq) were added, the tube was sealed and the reaction mixture was stirred at 100 °C for 24 h. The reaction was allowed to cool to room temperature, diluted with EtOAc and filtered over Celite with EtOAc. The combined filtrates were concentrated under reduces pressure and the crude product was purified by silica column chromatography (15 to 20% Et_2O in *n*-hexane, R_f 0.22 in 20% Et_2O in *n*-hexane) to give the title compound as a single diastereoisomer (>99:1 dr) as a yellow oil (81 mg, 46%). $[\boldsymbol{\alpha}]_{\boldsymbol{D}}^{\mathbf{20}}$ +54.1 (*c* 1.08 in $CHCl_3$).

chiral HPLC analysis Chiralpak AD-H (hexane : *i*-PrOH 93:7, flow rate 1.0 mlmin^{-1}, 254 nm, 40 °C) t_R (1*R*,2*S*,5*S*): 10.4 min, t_R (1*S*,2*R*,5*R*): 22.7 min, 93:7 er.

^{1}H NMR (500 MHz, $CDCl_3$) δ_H: 2.58 (1H, app. t, *J* 12.7, C(4)H^AH^B), 2.82 (1H, dd, *J* 13.0, 6.0, C(4)H^AH^B), 3.48 (1H, dd, *J* 10.7, 8.1, C(1)*H*), 3.56 – 3.65 (1H, m, C(5)*H*), 3.69 (3H, s, OCH_3), 3.95 (1H, app. p, *J* 7.5, C(2)*H*), 5.91 (1H, dd, *J* 15.8, 8.4, C(5′)*H*), 6.57 (1H, d, *J* 15.8, C(5′′)*H*), 7.27 – 7.38 (5H, m, 5 × Ar*H*).

^{13}C{^{1}H} NMR (126 MHz, $CDCl_3$) δ_C: 34.4 (*C*(3)), 43.7 (*C*(5)H), 43.9 (*C*(4)H_2), 46.2 (*C*(1)H), 53.0 (OCH_3), 55.0 (q, $^2J_{CF}$ 29.6, *C*(2)H), 112.3 (*C*N), 113.3 (*C*N), 122.6 (*C*(5′)H), 123.9 (q, $^1J_{CF}$ 279, *C*F_3), 126.4 (Ar*C*(2,6)H), 128.5 (Ar*C*(4)H), 128.8 (Ar*C*(3,5)H), 135.2 (*C*(5′′)H), 135.5 (Ar*C*(1)), 170.7 (*C*=O).

^{19}F{^{1}H} NMR (377 MHz, $CDCl_3$) δ_F: −67.4 (C*F*$_3$).

HRMS (ESI$^+$) $C_{18}H_{16}F_3N_2O_2$ $[M+H]^+$ found 349.1149, requires 349.1158 (−2.6 ppm).

ν_{max} (film, cm^{-1}) 3026 (=CH), 2954 (C-H), 1735 (C=O), 1494 (C=C_{Ar}), 1438, 1398, 1271, 1217, 1172, 1134, 968.

(2*S*,3*R*,4*S*)-3-(Hydroxymethyl)-2-(trifluoromethyl)-4-vinylcyclopentane-1,1-dicarbonitrile (S54)

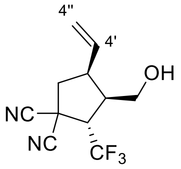

$C_{11}H_{11}F_3N_2O$
244.22

To a solution of PNP ester **4** (379 mg, 1.0 mmol, 1.0 eq) in MeOH (4.7 mL) and DME (0.3 mL) was added $NaBH_4$ (76 mg, 2.0 mmol, 2.0 eq) in portions at 0 °C. After complete addition, the reaction mixture was allowed to warm to room temperature and the reaction progress monitored by TLC. After full consumption of starting material (ca. 1h), the reaction was quenched by the addition of sat. aq. NH_4Cl (20 mL). The aqueous phase was extracted with EtOAc (3 × 20 mL) and the combined organic phases were washed with 1 M NaOH (2 × 20 mL) and brine (20 mL). The organic phase was dried over $MgSO_4$, filtered and the solvent was removed under reduced pressure. The crude product was purified by silica column chromatography (*n*-hexane : EtOAc 4:1, R_f 0.21) to give the title compound as a single diastereoisomer (>99:1 dr) as a pale yellow oil (90 mg, 36%).

$[\alpha]_D^{20}$ +2.8 (*c* 1.29 in $CHCl_3$).

Chiral GC analysis Restek Rt-βDEXcst (length: 30 m, thickness: 0.25 mm, film thickness: 0.25 μm, carrier gas: He, linear velocity: 28 cmsec^{-1}, temperature: 60 to 190 °C (43 min), 190 °C (15 min)) t_R (2*S*,3*R*,4*S*): 51.1 min, t_R (2*R*,3*S*,4*R*): 51.6 min, 93:7 er.

^{1}H NMR (500 MHz, $CDCl_3$) δ_H: 1.71 (1H, br s, O*H*), 2.51 (1H, app. t, *J* 12.7, C(5)H^AH^B), 2.55 – 2.62 (1H, m, C(3)*H*), 2.64 (1H, dd, *J* 12.6, 6.1, C(5)H^AH^B), 3.16 – 3.27 (1H, m, C(4)*H*), 3.62 (1H, app. p, *J* 8.2, C(2)*H*), 3.67 (1H, dd, *J* 10.9, 3.3, OCH^AH^B), 3.87 (1H, d, *J* 10.9, OCH^AH^B), 5.22 – 5.31 (2H, m, C(4′′)H_2), 5.94 (1H, ddd, *J* 17.0, 10.3, 8.1, C(4′)*H*).

^{13}C{^{1}H} NMR (126 MHz, $CDCl_3$) δ_C: 34.2 (*C*(1)), 41.8 (*C*(3)H), 44.5 (*C*(5)H_2), 44.6 (*C*(4)H), 53.1 (q, $^2J_{CF}$ 28.5, *C*(2)H), 60.2 (OCH_2), 113.0 (CN), 114.3 (CN), 119.7 (*C*(4′′)H_2), 124.8 (q, $^1J_{CF}$ 279, *C*F_3), 133.9 (*C*(4′)H).

^{19}F{^{1}H} NMR (377 MHz, $CDCl_3$) δ_F: −67.0 (C*F*$_3$).

HRMS (ESI$^+$) $C_{11}H_{12}F_3N_2O$ [M+H]$^+$ found 245.0896, requires 245.0896 (±0.0 ppm).

ν_{max} (film, cm^{-1}) 3549 (O-H), 3086 (=CH_2), 2953 (C-H), 2893, 2258 (C≡N), 1643 (C=C), 1384, 1267, 1165, 1126, 999, 929.

(1*R*,3a*R*,4*S*,6a*R*)-1-(Iodomethyl)-4-(trifluoromethyl)tetrahydro-1*H*-cyclopenta[c]furan-5,5(3*H*)-dicarbonitrile (27_{maj}) and (1*S*,3a*R*,4*S*,6a*R*)-1-(Iodomethyl)-4-(trifluoromethyl) tetrahydro-1*H*-cyclopenta[c]furan-5,5(3*H*)-dicarbonitrile (27_{min})

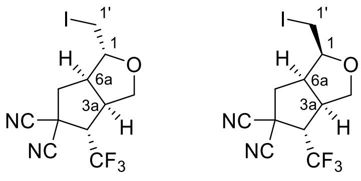

27_{maj} **27_{min}**

$C_{11}H_{10}F_3IN_2O$
370.11

Following the procedure reported by Jørgensen and co-workers[40], alcohol **S51** (76 mg, 0.3 mmol, 1.0 eq) was dissolved in THF : sat. aq. $NaHCO_3$ 3:1 (2.4 mL) and cooled to 0 °C. Iodine (237 mg, 0.93 mmol, 3.0 eq) was added in portions over 5 h and the reaction mixture stirred for another 16 h at 0 °C. The reaction was quenched by the addition of brine (20 mL) and the aqueous phase was extracted with EtOAc (3 × 20 mL). The combined organic phases were washed with sat. aq. $Na_2S_2O_3$ (20 mL), H_2O (20 mL) and brine (20 mL), dried over $MgSO_4$, filtered and the solvent was removed under reduced pressure. The crude product was purified by silica column chromatography (*n*-hexane : EtOAc 5:1 to 4:1) to give:

27_{maj} (R_f 0.34 in *n*-hexane : EtOAc 4:1) as an off-white solid (66 mg, 58%). **m.p.** (EtOAc) 54 – 57 °C. $[\alpha]_D^{20}$ −10.0 (*c* 1.02 in $CHCl_3$). The enantiomeric ratio could not be determined.

^{1}H NMR (500 MHz, $CDCl_3$) δ_H: 2.29 (1H, dd, *J* 13.3, 9.3, C(6)H^AH^B), 2.99 (1H, dq, *J* 9.8, 7.1, C(4)*H*), 3.03 – 3.29 (5H, m, C(1′)H_2, C(3a)*H*, C(6)H^AH^B, C(6a)*H*), 3.90 (1H, dd, *J* 10.3, 2.5, C(3)H^AH^B), 4.07 (1H, dd, *J* 10.3, 6.5, C(3)H^AH^B), 4.11 (1H, td, *J* 5.6, 2.7, C(1)*H*).

^{13}C{^{1}H} NMR (126 MHz, $CDCl_3$) δ_C: 5.4 (*C*(1′)H_2), 36.8 (*C*(5)), 44.2 (*C*(3a)H), 44.5 (*C*(6)H_2), 47.5 (*C*(6a)H), 56.8 (q, $^2J_{CF}$ 28.6, *C*(4)H), 70.5 (*C*(3)H_2), 84.9 (*C*(1)H), 112.0 (CN), 112.9 (CN), 123.9 (q, $^1J_{CF}$ 280, CF_3).

^{19}F{^{1}H} NMR (377 MHz, $CDCl_3$) δ_F: −66.9 (C*F*$_3$).

HRMS (ESI$^-$) $C_{11}H_{10}F_3IN_2OCl$ $[M+Cl]^-$ found 404.9458, requires 404.9484 (−2.6 ppm).

ν_{max} ($CHCl_3$, cm^{-1}) 3020 (CH_2-I), 2956 (C-H), 2887, 2258 (C≡N), 1392, 1271, 1176, 1132, 1070, 916.

27_{min} (R_f 0.39 in *n*-hexane : EtOAc 4:1) as a white solid (24 mg, 21%). **m.p.** (EtOAc) 121 – 124 °C. $[\alpha]_D^{20}$ −70.1 (*c* 1.15 in $CHCl_3$). The enantiomeric ratio could not be determined.

^{1}**H NMR** (500 MHz, $CDCl_3$) δ_H: 2.18 (1H, dd, *J* 12.9, 11.1, C(6)H^AH^B), 2.81 – 2.92 (2H, m, C(4)*H*, C(6)H^AH^B), 3.01 (1H, dd, *J* 10.4, 9.3, C(1′)H^AH^B), 3.19 (1H, td, *J* 9.7, 6.5, C(3a)*H*), 3.30 – 3.39 (2H, m, C(1′)H^AH^B, C(6a)*H*), 3.79 (1H, dd, *J* 10.2, 6.5, C(3)H^AH^B), 3.94 – 4.02 (2H, m, C(1)*H*, C(3)H^AH^B).

^{13}**C{**^{1}**H} NMR** (126 MHz, $CDCl_3$) δ_C: −2.1 (*C*(1′)H_2), 35.7 (*C*(5)), 38.5 (*C*(6)H_2), 43.7 (*C*(3a)H), 45.1 (*C*(6a)H), 58.0 (q, $^2J_{CF}$ 28.5, *C*(4)H), 72.2 (*C*(3)H_2), 81.3 (*C*(1)H), 111.9 (CN), 112.7 (CN), 124.0 (q, $^1J_{CF}$ 278, *C*F_3).

^{19}**F{**^{1}**H} NMR** (377 MHz, $CDCl_3$) δ_F: −67.2 (C*F*$_3$).

HRMS (ESI$^-$) $C_{11}H_{10}F_3IN_2OCl$ $[M+Cl]^-$ found 404.9475, requires 404.9484 (−0.9 ppm).

ν_{max} (film, cm^{-1}) 2958 (C-H), 2875, 2854, 2260 (C≡N), 1390, 1271, 1259, 1168, 1136, 1026, 950.

(1*R*,2*S*,5*S*)-*N*-Allyl-3,3-dicyano-2-(trifluoromethyl)-5-vinylcyclopentane-1-carboxamide (S55)

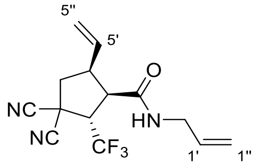

$C_{14}H_{14}F_3N_3O$
297.28

To a solution of PNP ester **4** (379 mg, 1.0 mmol, 1.0 eq) in EtOAc (5.0 mL) was added allyl amine (0.15 mL, 2.0 mmol, 2.0 eq) and the reaction mixture was stirred at room temperature until TLC indicated full conversion (ca. 5 h). The mixture was diluted with EtOAc (10 mL) and washed with 1 M NaOH (2 × 10 mL) and brine (10 mL). The organic phase was dried over $MgSO_4$, filtered and concentrated under reduced pressure. The crude product was purified by silica column chromatography (*n*-hexane : EtOAc 3:1, R_f 0.24) to give the title compound as a single diastereoisomer (>99:1 dr) as a white solid (233 mg, 78%). **m.p.** (EtOAc) 127 – 130 °C. $[\alpha]_D^{20}$ +21.8 (*c* 1.12 in $CHCl_3$).

chiral HPLC analysis Chiralpak IA, (*n*-hexane : *i*-PrOH 97:3, flow rate 1.0 mLmin^{-1}, 211 nm, 40 °C) t_R (1*R*,2*S*,5*S*): 13.8 min, t_R (1*S*,2*R*,5*R*): 23.3 min, 92:8 er.

^{1}**H NMR** (500 MHz, $CDCl_3$) δ_H: 2.55 (1H, app. t, *J* 12.8, C(4)H^AH^B), 2.66 (1H, dd, *J* 12.8, 5.9, C(4)H^AH^B), 3.06 (1H, dd, *J* 10.3, 7.4, C(1)*H*), 3.25 – 3.36 (1H, m, C(5)*H*), 3.81 – 3.95 (2H, m, NC*H*$_2$), 4.03 (1H, app. p, *J* 7.9, C(2)*H*), 5.14 – 5.22 (2H, m, C(1′′)*H*$_2$), 5.22 – 5.32 (2H, m, C(5′′)*H*$_2$), 5.62 (1H, ddd, *J* 17.0, 10.1, 8.7, C(5′)*H*), 5.78 (1H, ddt, *J* 17.2, 10.2, 5.7, C(1′)*H*), 5.83 (1H, br t, *J* 5.8, N*H*).

^{13}**C{^{1}H} NMR** (126 MHz, $CDCl_3$) δ_C: 34.4 (*C*(3)), 42.4 (N*C*H_2), 44.1 (*C*(4)H_2), 45.3 (*C*(5)H), 47.1 (*C*(1)H), 55.1 (q, $^2J_{CF}$ 29.3, *C*(2)H), 112.6 (*C*N), 113.3 (*C*N), 117.3 (*C*(1′′)H_2), 120.6 (*C*(5′′)H_2), 124.1 (q, $^1J_{CF}$ 279, *C*F_3), 132.6 (*C*(5′)H), 133.0 (*C*(1′)H), 168.4 (*C*=O).

^{19}**F{^{1}H} NMR** (377 MHz, $CDCl_3$) δ_F: −67.3 (C*F*$_3$).

HRMS (ESI$^+$) $C_{14}H_{15}F_3N_3O$ [M+H]$^+$ found 298.1157, requires 298.1161 (−1.5 ppm).

ν_{max} (film, cm^{-1}) 3288 (N-H), 3115, 2945 (C-H), 2850, 1681(C=C), 1653 (C=C), 1639 (C=O), 1575 (C=O), 1433, 1371, 1271, 1170, 1134, 1031, 993, 941, 923.

(5a*S*,8*S*,8a*R*)-1-Oxo-8-(trifluoromethyl)-2,3,5a,6,8,8a-hexahydrocyclopent[c]azepine-7,7(2*H*)-dicarbonitrile (28)

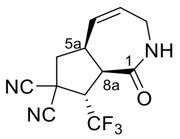

$C_{12}H_{10}F_3N_3O$
269.23

Amide **S52** (148 mg, 0.5 mmol, 1.0 eq) and *p*-TsOH·H_2O (129 mg, 0.75 mmol, 1.5 eq) were placed in a flame-dried round bottom flask under a N_2 atmosphere. Toluene (50 mL, degassed with Ar for 30 min) was added, followed by HG-II catalyst (16 mg, 0.025 mmol, 5 mol%) and the reaction mixture was heated to 80 °C. After 24 h, additional 5 mol% HG-II catalyst were added and stirring at 80 °C continued for another 24 h. The reaction mixture was allowed to cool to room temperature and the solvent was removed under reduced pressure. The crude residue was dissolved in CH_2Cl_2 (50 mL) and washed with 1 M NaOH (50 mL). The aqueous phase was extracted with CH_2Cl_2 (3 × 15 mL), the combined organic phases were dried over $MgSO_4$, filtered and the solvent was removed under reduced pressure. The crude product was purified by silica column chromatography (10 to 20% Et_2O in CH_2Cl_2, R_f 0.33 in 20% Et_2O in CH_2Cl_2) to afford the title compound as a single diastereoisomer (>99:1 dr) as a white solid (28 mg, 20%).

m.p. ($CHCl_3$) 123 – 125 °C. $[\alpha]_D^{20}$ +7.7 (*c* 1.30 in $CHCl_3$).

chiral HPLC analysis Chiralpak AD-H (hexane : *i*-PrOH 92:8, flow rate 1.0 mlmin^{-1}, 211 nm, 40 °C) t_R (5a*S*,8*S*,8a*R*): 14.3 min, t_R (5a*R*,8*R*,8a*S*): 23.2 min, 94:6 er.

^{1}**H NMR** (500 MHz, $CDCl_3$) δ_H: 2.18 (1H, dd, *J* 14.4, 12.7, C(6)H^AH^B), 2.76 (1H, dd, *J* 12.7, 4.8, C(6)H^AH^B), 3.22 – 3.33 (1H, m, C(5a)*H*), 3.43 (1H, app. dt, *J* 16.6, 7.6, C(3)H^AH^B), 3.69 (1H, dd, *J* 9.0, 3.7, C(8a)*H*), 3.99 – 4.10 (1H, m, C(3)H^AH^B), 4.74 (1H, qd, *J* 9.4, 3.7, C(8)*H*), 5.71 (1H, dt, *J* 10.9, 2.7, C(5)*H*), 6.08 – 6.18 (1H, m, C(4)*H*), 7.03 – 7.16 (1H, m, N*H*).

^{13}**C{^{1}H} NMR** (126 MHz, $CDCl_3$) δ_C: 32.8 (*C*(7)), 37.5 (*C*(3)H_2), 40.8 (*C*(5a)H), 44.1 (*C*(8a)H), 44.2 (*C*(6)H_2), 53.1 (q, $^{2}J_{CF}$ 29.4, *C*(8)H), 113.1 (CN), 114.0 (CN), 124.6 (q, $^{1}J_{CF}$ 279, *C*F_3), 129.0 (*C*(5)H), 129.6 (*C*(4)H), 169.4 (*C*=O).

^{19}**F{^{1}H} NMR** (377 MHz, $CDCl_3$) δ_F: −67.2 (C*F*$_3$).

HRMS (ESI$^+$) $C_{12}H_{10}F_3N_3ONa$ [M+Na]$^+$ found 292.0660, requires 292.0668 (−2.8 ppm).

ν_{max} (film, cm^{-1}) 3261 (N-H), 3088, 2960 (C-H), 2256 (C≡N), 1672 (C=O), 1475, 1381, 1305, 1274, 1201, 1159, 1132, 1103, 900.

13 Single crystal X-ray diffraction analysis

Data were collected using CrystalClear[1] and processed (including correction for Lorentz, polarization and absorption) using CrysAlisPro.[2] Structures were solved by dual-space (SHELXT[41]), direct (SIR2011[42]) or charge-flipping (Superflip[43]) methods and refined by full-matrix least-squares against *F*2 (SHELXL-2018/3[44]). Non-hydrogen atoms were refined anisotropically, and all hydrogen atoms were refined using a riding model. All calculations were performed using the CrystalStructure[3] interface.

Data for $\mathbf{13_{maj}}$

X-ray diffraction data were collected at 173 K on a Rigaku XtaLAB P100 diffractometer using multi-layer mirror monochromated Cu-Kα radiation (λ = 1.54187 Å).

	$\mathbf{13_{maj}}$ (1*R*,2*S*,5*S*)
CCDC	2152447
empirical formula	$C_{16}H_{20}N_2O_4$
fw	304.34
crystal description	colourless, prism
crystal size [mm]	0.200 × 0.100 × 0.100
space group	$P2_1$ (#4)
a [Å]	9.560(3)
b [Å]	15.563(4)
c [Å]	11.591(4)
vol [Å]^3	1724.5(9)
β [°]	90.427(6)
Z	4
ρ (calc) [g/cm^3]	1.172
μ [mm^{-1}]	0.700
F(000)	648.00
reflections collected	18151
independent reflections (R_{int})	5516 (0.0520)
data/parameters	5516/406
GOF on F^2	1.052
R_1 [$I > 2\sigma(I)$]	0.0556
wR_2 (all data)	0.1343
largest diff. peak/hole [e/ Å^3]	0.62, −0.63
Flack parameter	0.04(6)

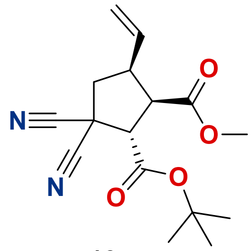

$\mathbf{13_{maj}}$

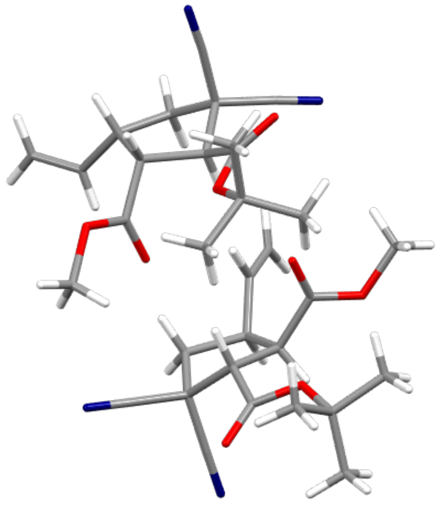

[1] CrystalClear-SM Expert v2.1. Rigaku Americas, The Woodlands, Texas, USA, and Rigaku Corporation, Tokyo, Japan, 2015

[2] CrysAlisPro v1.171.38.46. Rigaku Oxford Diffraction, Rigaku Corporation, Oxford, U.K. 2015

[3] CrystalStructure v4.3.0. Rigaku Americas, The Woodlands, Texas, USA, and Rigaku Corporation, Tokyo, Japan, 2018.

Data for **28**

X-ray diffraction data were collected at 173 K on a Rigaku XtaLAB P200 diffractometer using multi-layer mirror monochromated Mo-Kα radiation (λ = 0.71075 Å).

	**28** (5a*S*,8*S*,8a*R*)
CCDC	2152446
empirical formula	$C_{12}H_{10}F_3N_3O$
fw	269.23
crystal description	colourless, platelet
crystal size [mm]	0.100 × 0.100 × 0.010
space group	$P4_32_12$ (#96)
a [Å]	7.5386(8)
c [Å]	42.598(5)
vol [Å]^3	2420.9(5)
Z	8
ρ (calc) [g/cm^3]	1.477
μ [mm^{-1}]	0.288
F(000)	1104.00
reflections collected	18286
independent reflections (R_{int})	2212 (0.0418)
data/parameters	2212/176
GOF on F^2	1.140
R_1 [$I > 2\sigma(I)$]	0.0509
wR_2 (all data)	0.1324
largest diff. peak/hole [e/ Å^3]	0.23, −0.16

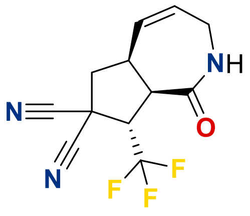

28

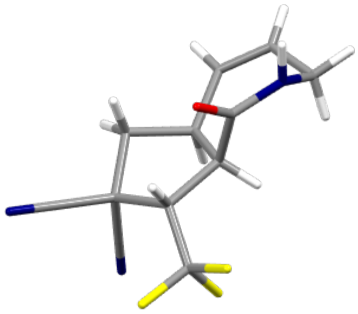

Data for **27**$_{maj}$

X-ray diffraction data were collected at 173 K on a Rigaku SCX mini diffractometer using graphite monochromated Mo-Kα radiation (λ = 0.71075 Å).

	**27**$_{maj}$ (1*R*,3a*R*,4*S*,6a*R*)
CCDC	2152445
empirical formula	$C_{11}H_{10}F_3IN_2O$
fw	370.11
crystal description	colourless, prism
crystal size [mm]	0.340 × 0.190 × 0.170
space group	P1 (#1)
a [Å]	7.6910(5)
b [Å]	11.5327(8)
c [Å]	11.9797(8)
vol [Å]^3	991.04(12)
α [°]	79.079(6)
β [°]	79.774(6)
γ [°]	73.365(5)
Z	3
ρ (calc) [g/cm^3]	1.860
μ [mm^{-1}]	2.4495
F(000)	534.00
reflections collected	9863
independent reflections (R_{int})	8424 (0.0252)
data/parameters	8424/487
GOF on F^2	1.046
R_1 [$I > 2\sigma(I)$]	0.0340
wR_2 (all data)	0.0886
largest diff. peak/hole [e/ Å^3]	0.70, −1.03
Flack parameter	0.025(11)

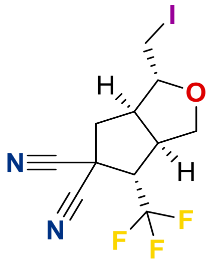

27$_{maj}$

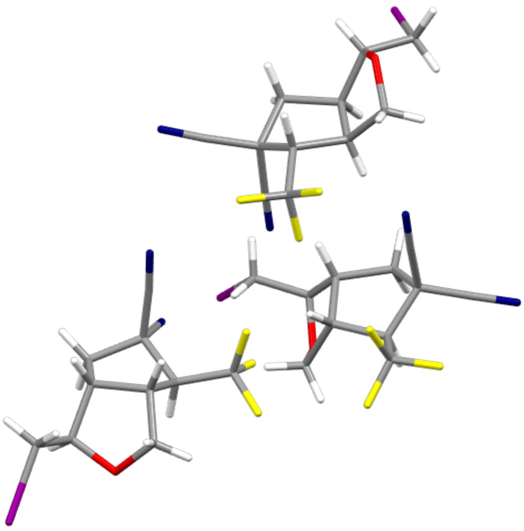

14 References

1 S. S. Zalesskiy and V. P. Ananikov, *Organometallics*, 2012, **31**, 2302–2309.

2 A. P. Dieskau, M. S. Holzwarth and B. Plietker, *J. Am. Chem. Soc.*, 2012, **134**, 5048–5051.

3 A. Matviitsuk, M. D. Greenhalgh, D.-J. B. Antúnez, A. M. Z. Slawin and A. D. Smith, *Angew. Chem. Int. Ed.*, 2017, **56**, 12282–12287.

4 M. D. Greenhalgh, S. Qu, A. M. Z. Slawin and A. D. Smith, *Chem. Sci.*, 2018, **9**, 4909–4918.

5 C. M. Young, A. Elmi, D. J. Pascoe, R. K. Morris, C. McLaughlin, A. M. Woods, A. B. Frost, A. de la Houpliere, K. B. Ling, T. K. Smith, A. M. Z. Slawin, P. H. Willoughby, S. L. Cockroft and A. D. Smith, *Angew. Chem. Int. Ed.*, 2020, **59**, 3705–3710.

6 D. Daniels, S. Smith, T. Lebl, P. Shapland and A. Smith, *Synthesis*, 2014, **47**, 34–41.

7 D. Belmessieri, C. Joannesse, P. A. Woods, C. MacGregor, C. Jones, C. D. Campbell, C. P. Johnston, N. Duguet, C. Concellón, R. A. Bragg and A. D. Smith, *Org. Biomol. Chem.*, 2011, **9**, 559–570.

8 N. R. Guha, R. M. Neyyappadath, M. D. Greenhalgh, R. Chisholm, S. M. Smith, M. L. McEvoy, C. M. Young, C. Rodríguez-Escrich, M. A. Pericàs, G. Hähner and A. D. Smith, *Green Chem.*, 2018, **20**, 4537–4546.

9 I. Shiina, K. Nakata, K. Ono, Y. Onda and M. Itagaki, *J. Am. Chem. Soc.*, 2010, **132**, 11629–11641.

10 F. Wei, C.-L. Ren, D. Wang and L. Liu, *Chem. Eur. J.*, 2015, **21**, 2335–2338.

11 J. Preindl, S. Chakrabarty and J. Waser, *Chem. Sci.*, 2017, **8**, 7112–7118.

12 J. M. Takacs, Z. Xu, X. Jiang, A. P. Leonov and G. C. Theriot, *Org. Lett.*, 2002, **4**, 3843–3845.

13 B. M. Trost, P. J. Morris and S. J. Sprague, *J. Am. Chem. Soc.*, 2012, **134**, 17823–17831.

14 K. Burgess, *J. Org. Chem.*, 1987, **52**, 2046–2051.

15 C. S. Buxton, D. C. Blakemore and J. F. Bower, *Angew. Chem. Int. Ed.*, 2017, **56**, 13824–13828.

16 J. M. Ellis, L. E. Overman, H. R. Tanner and J. Wang, *J. Org. Chem.*, 2008, **73**, 9151–9154.

17 J.-A. Xiao, X.-L. Cheng, Y.-C. Li, Y.-M. He, J.-L. Li, Z.-P. Liu, P.-J. Xia, W. Su and H. Yang, *Org. Biomol. Chem.*, 2019, **17**, 103–107.

18 H. Liu, A. M. Z. Slawin and A. D. Smith, *Org. Lett.*, 2020, **22**, 1301–1305.

19 J. Wu, C. M. Young and A. D. Smith, *Tetrahedron*, 2021, **78**, 131758.

20 H. M. Walborsky and M. Schwarz, *J. Am. Chem. Soc.*, 1953, **75**, 3241–3243.

21 H. Plenkiewicz, W. Dmowski and M. Lipínski, *J. Fluor. Chem.*, 2001, **111**, 227–232.

22 C. Shu, H. Liu, A. M. Z. Slawin, C. Carpenter-Warren and A. D. Smith, *Chem. Sci.*, 2020, **11**, 241–247.

23 R. Pajkert and G.-V. Röschenthaler, *J. Org. Chem.*, 2013, **78**, 3697–3708.

24 P. Zaderenko, M. C. López and P. Ballesteros, *J. Org. Chem.*, 1996, **61**, 6825–6828.

25 A. El-Batta, C. Jiang, W. Zhao, R. Anness, A. L. Cooksy and M. Bergdahl, *J. Org. Chem.*, 2007, **72**, 5244–5259.

26 N. Kaur, B. Zhou, F. Breitbeil, K. Hardy, K. S. Kraft, I. Trantcheva and O. Phanstiel IV, *Mol. Pharm.*, 2008, **5**, 294–315.

27 P. A. Jacobi, C. A. Blum, R. W. DeSimone and U. E. S. Udodong, *Tetrahedron Lett.*, 1989, **30**, 7173–7176.

28 J. Bitai, A. M. Z. Slawin, D. B. Cordes and A. D. Smith, *Molecules*, 2020, **25**, 2463.

29 L. Hutchings-Goetz, C. Yang and T. N. Snaddon, *ACS Catal.*, 2018, **8**, 10537–10544.

30 C. McLaughlin, A. M. Z. Slawin and A. D. Smith, *Angew. Chem. Int. Ed.*, 2019, **58**, 15111–15119.

31 L. Mola, J. Font, L. Bosch, J. Caner, A. M. Costa, G. Etxebarría-Jardí, O. Pineda, D. de Vicente and J. Vilarrasa, *J. Org. Chem.*, 2013, **78**, 5832–5842.

32 A. Ovat, F. Muindi, C. Fagan, M. Brouner, E. Hansell, J. Dvořák, D. Sojka, P. Kopáček, J. H. McKerrow, C. R. Caffrey and J. C. Powers, *J. Med. Chem.*, 2009, **52**, 7192–7210.

33 D. Tejedor, S. J. Álvarez-Méndez, J. M. López-Soria, V. S. Martín and F. García-Tellado, *Eur. J. Org. Chem.*, 2014, **2014**, 198–205.

34 M. Wende and J. A. Gladysz, *J. Am. Chem. Soc.*, 2003, **125**, 5861–5872.

35 S. Karlsson and H.-E. Högberg, *Org. Lett.*, 1999, **1**, 1667–1669.

36 H. Lebel and M. Davi, *Adv. Synth. Catal.*, 2008, **350**, 2352–2358.

37 X. Lu, W. Wang, Q. Dong, X. Bao, X. Lin, W. Zhang, X. Dong and W. Zhao, *Chem. Commun.*, 2015, **51**, 1498–1501.

38 S. Sano, T. Matsumoto, M. Toguchi and M. Nakao, *Synlett*, 2018, **29**, 1461–1464.

39 Z. Yuan, W. Wei, A. Lin and H. Yao, *Org. Lett.*, 2016, **18**, 3370–3373.

40 K. S. Halskov, L. Næsborg, F. Tur and K. A. Jørgensen, *Org. Lett.*, 2016, **18**, 2220–2223.

41 G. M. Sheldrick, *Acta Crystallogr., Sect. A*, 2015, **71**, 3–8.

42 M. C. Burla, R. Caliandro, M. Camalli, B. Carrozzini, G. L. Cascarano, C. Giacovazzo, M. Mallamo, A. Mazzone, G. Polidori and R. Spagna, *J. Appl. Crystallogr.*, 2012, **45**, 357–361.

43 L. Palatinus and G. Chapuis, *J. Appl. Crystallogr.*, 2007, **40**, 786–790.

44 G. M. Sheldrick, *Acta Crystallogr., Sect. C*, 2015, **71**, 3–8.

Appendix I: ^{1}H, $^{13}C\{^{1}H\}$, $^{19}F\{^{1}H\}$ and $^{31}P\{^{1}H\}$ NMR spectra of novel compounds

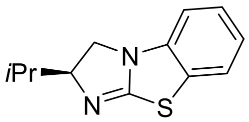

•HCl

LB2

^{1}H, DMSO-d_6, 500 MHz

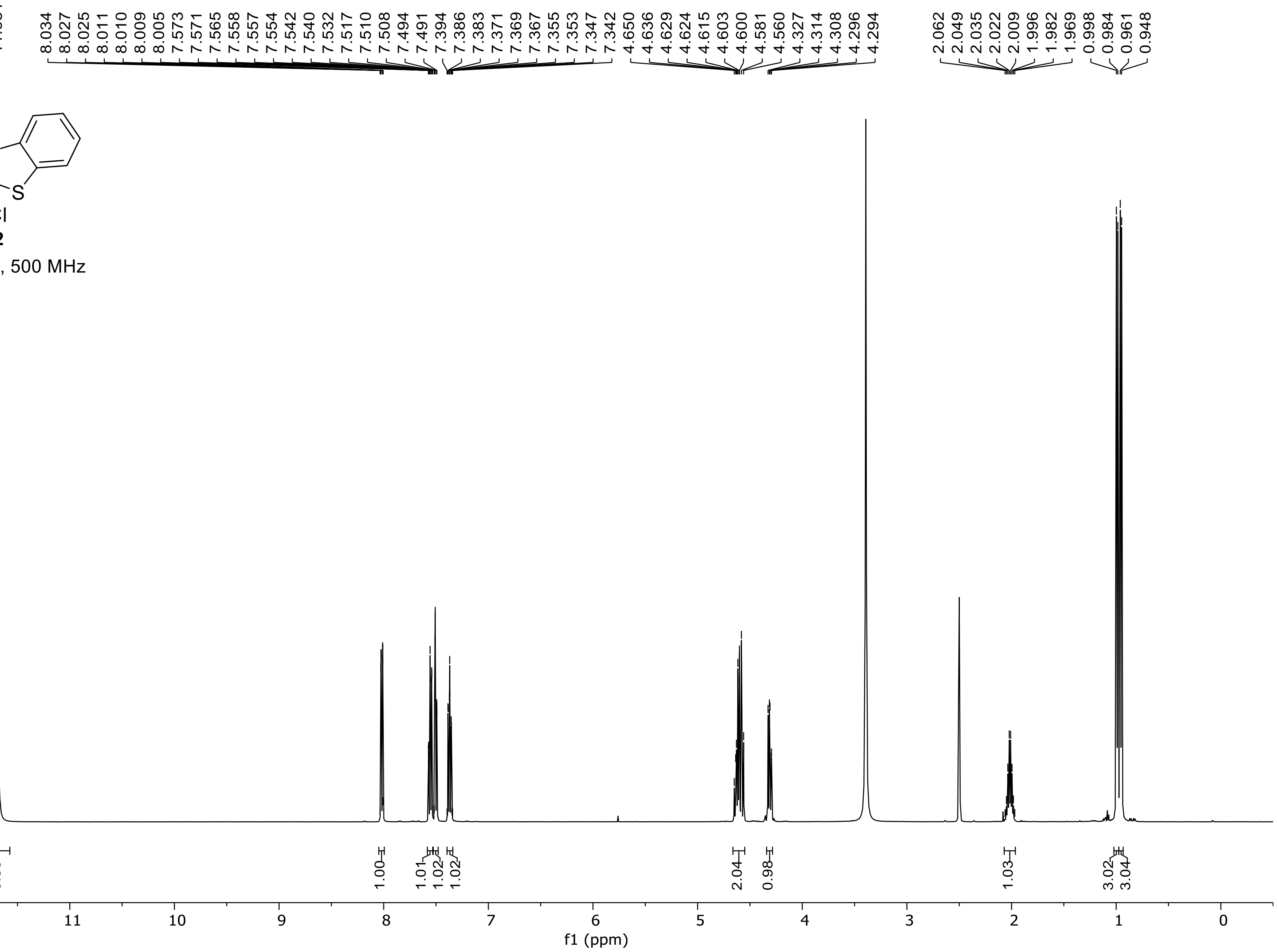

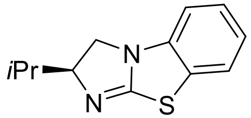

•HCl
LB2
$^{13}C\{^{1}H\}$, DMSO-d_6, 126 MHz

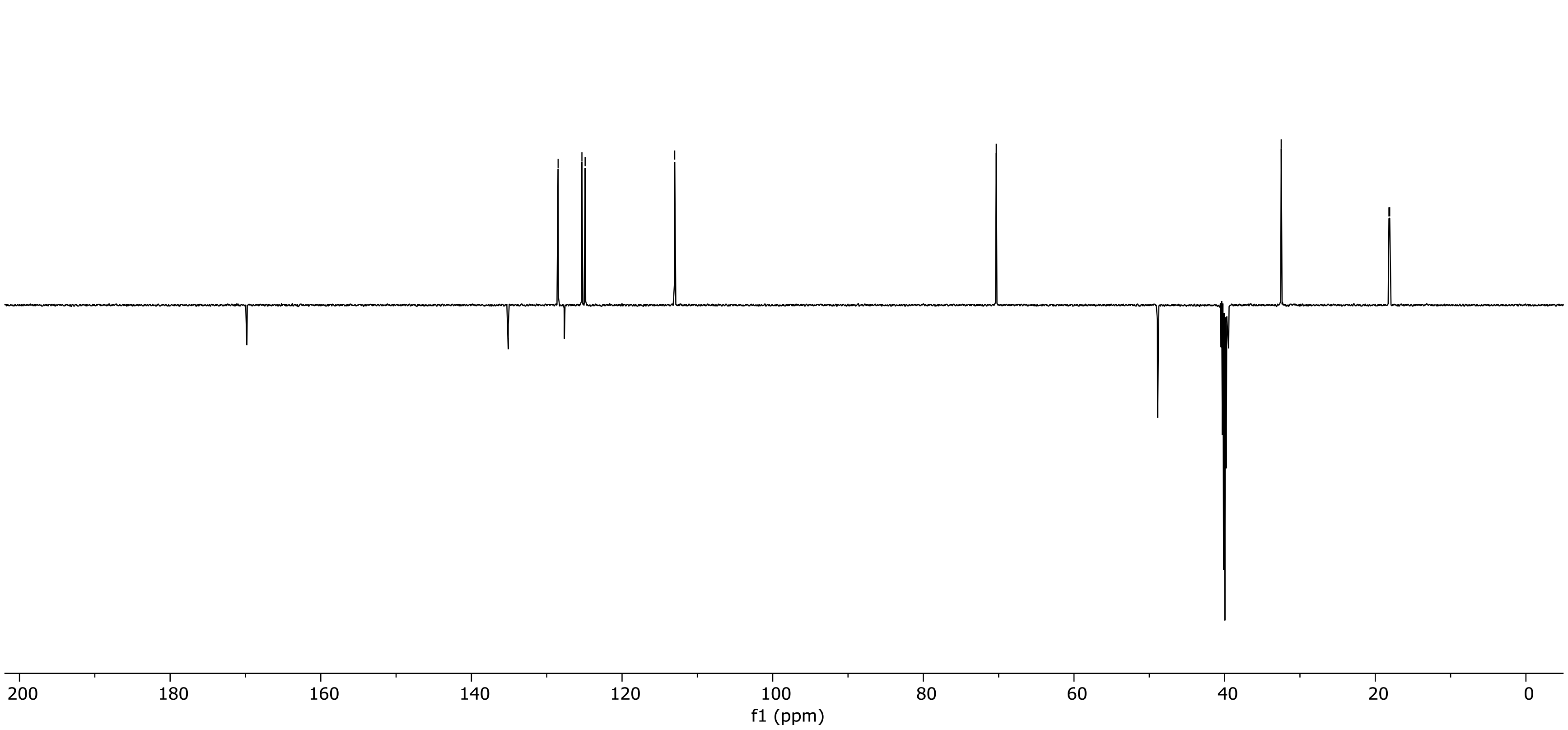

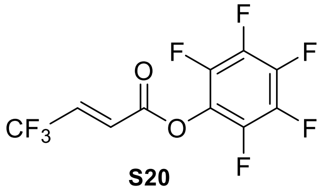

S20

^{1}H, $CDCl_3$, 500 MHz

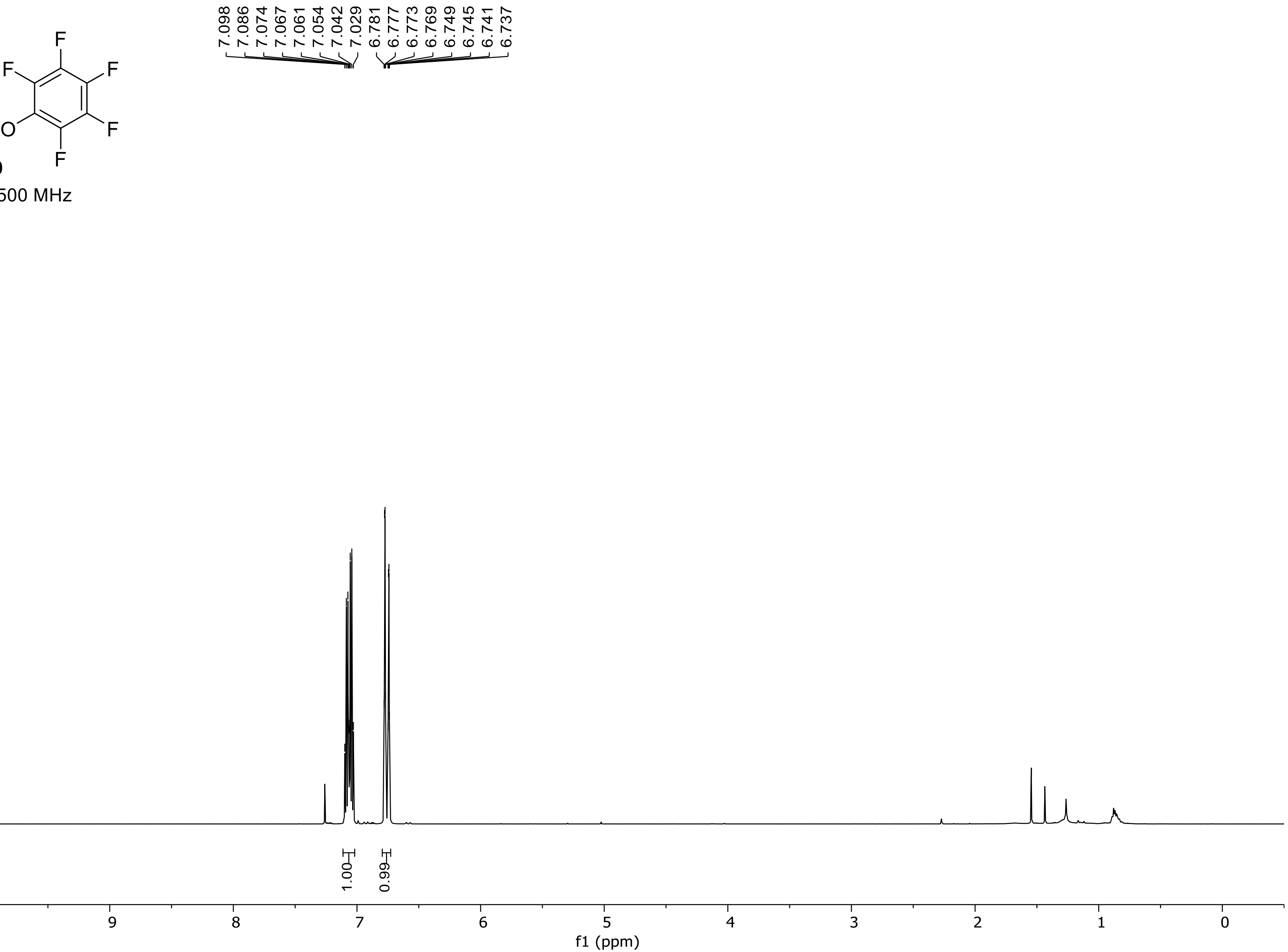

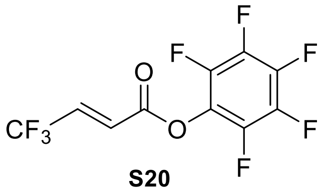

S20

$^{13}C\{^1H\}$, $CDCl_3$, 126 MHz

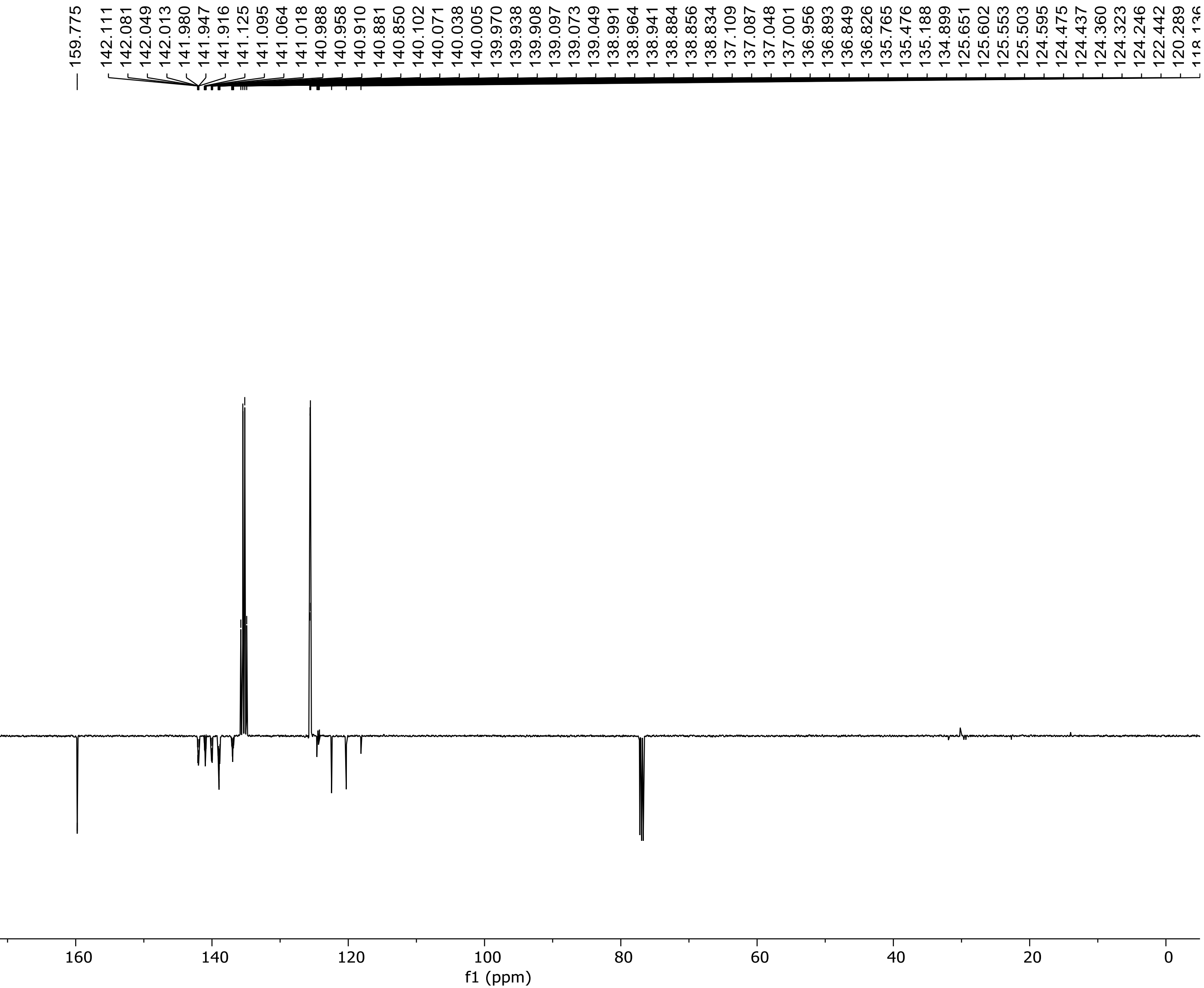

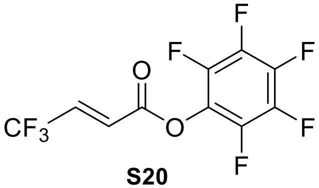

S20

$^{19}F\{^1H\}$, $CDCl_3$, 376 MHz

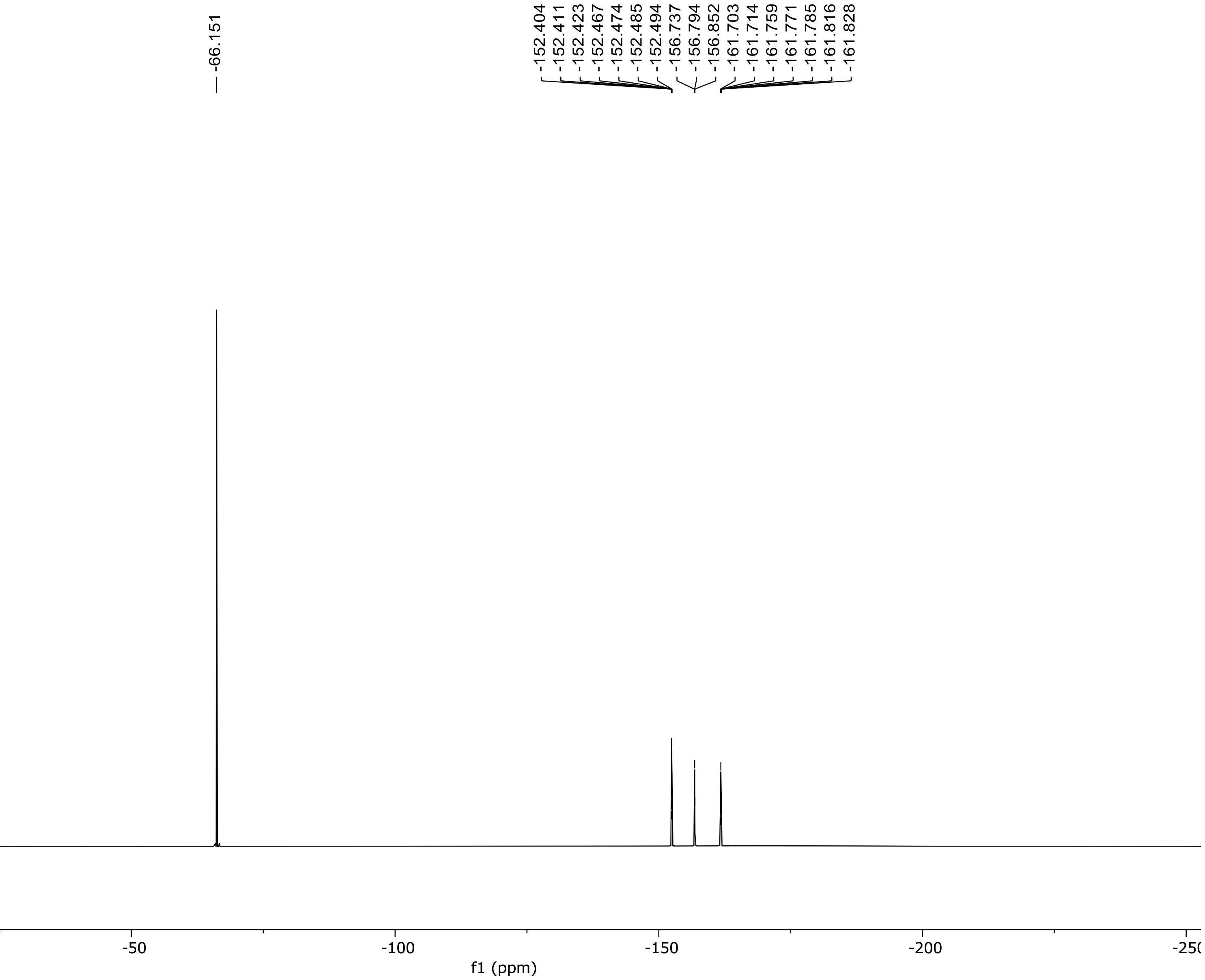

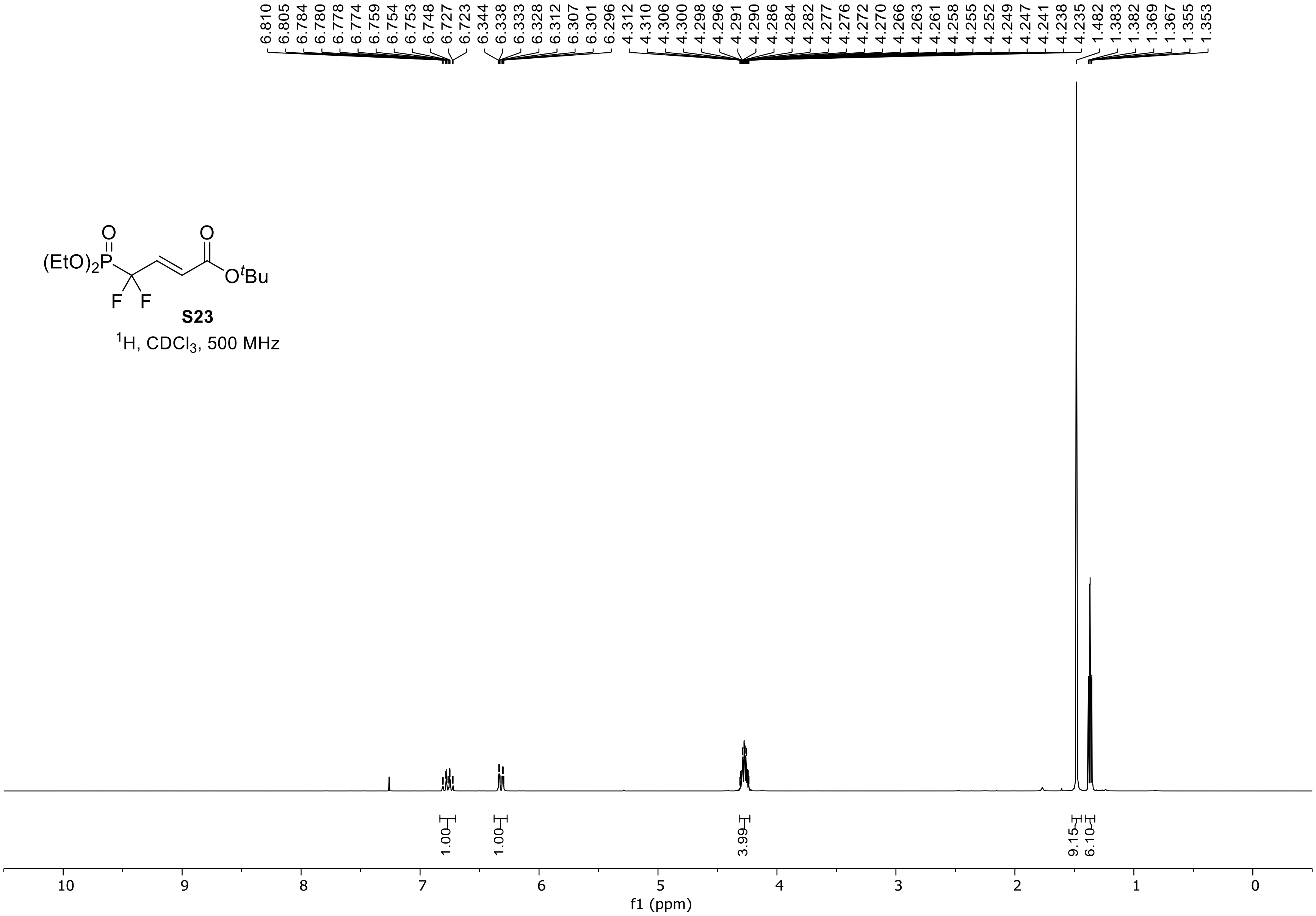
S23
^{1}H, CDCl$_3$, 500 MHz
f1 (ppm)

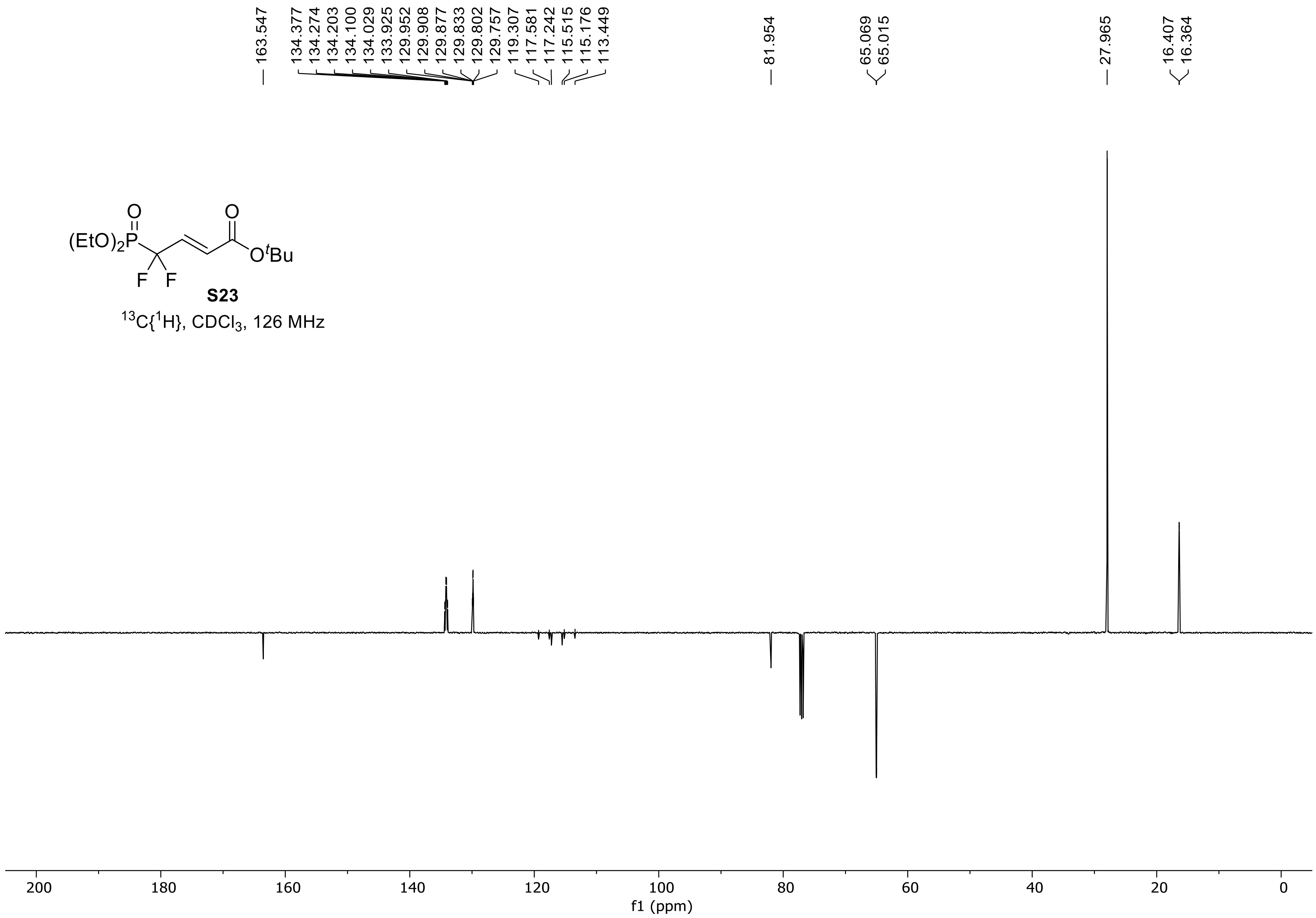

S23

$^{13}C\{^{1}H\}$, $CDCl_3$, 126 MHz

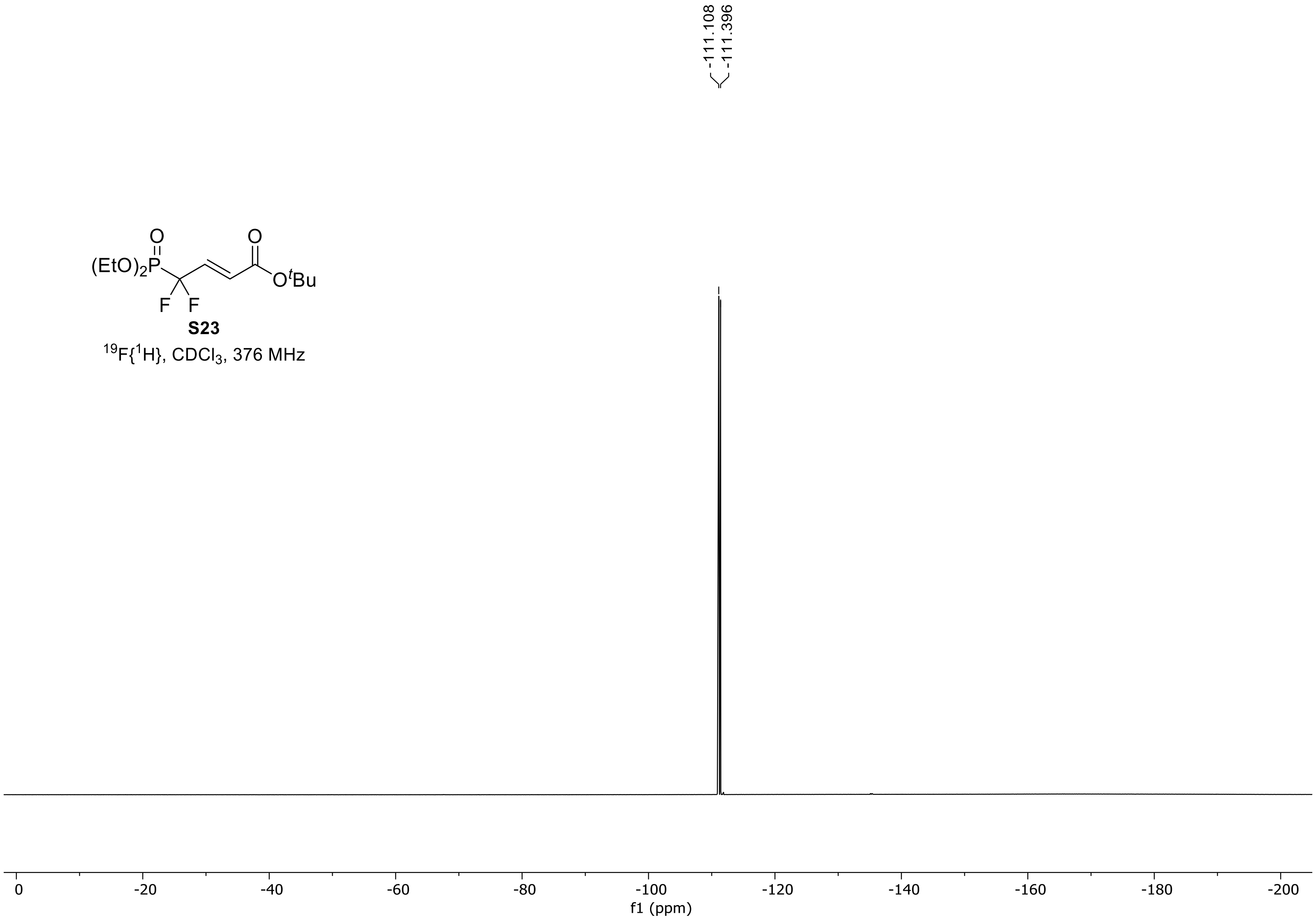

-111.108
-111.396
(EtO)2P
O
O
OtBu
F F
S23
19F{1H}, CDCl3, 376 MHz
0
-20
-40
-60
-80
-100
-120
-140
-160
-180
-200
f1 (ppm)

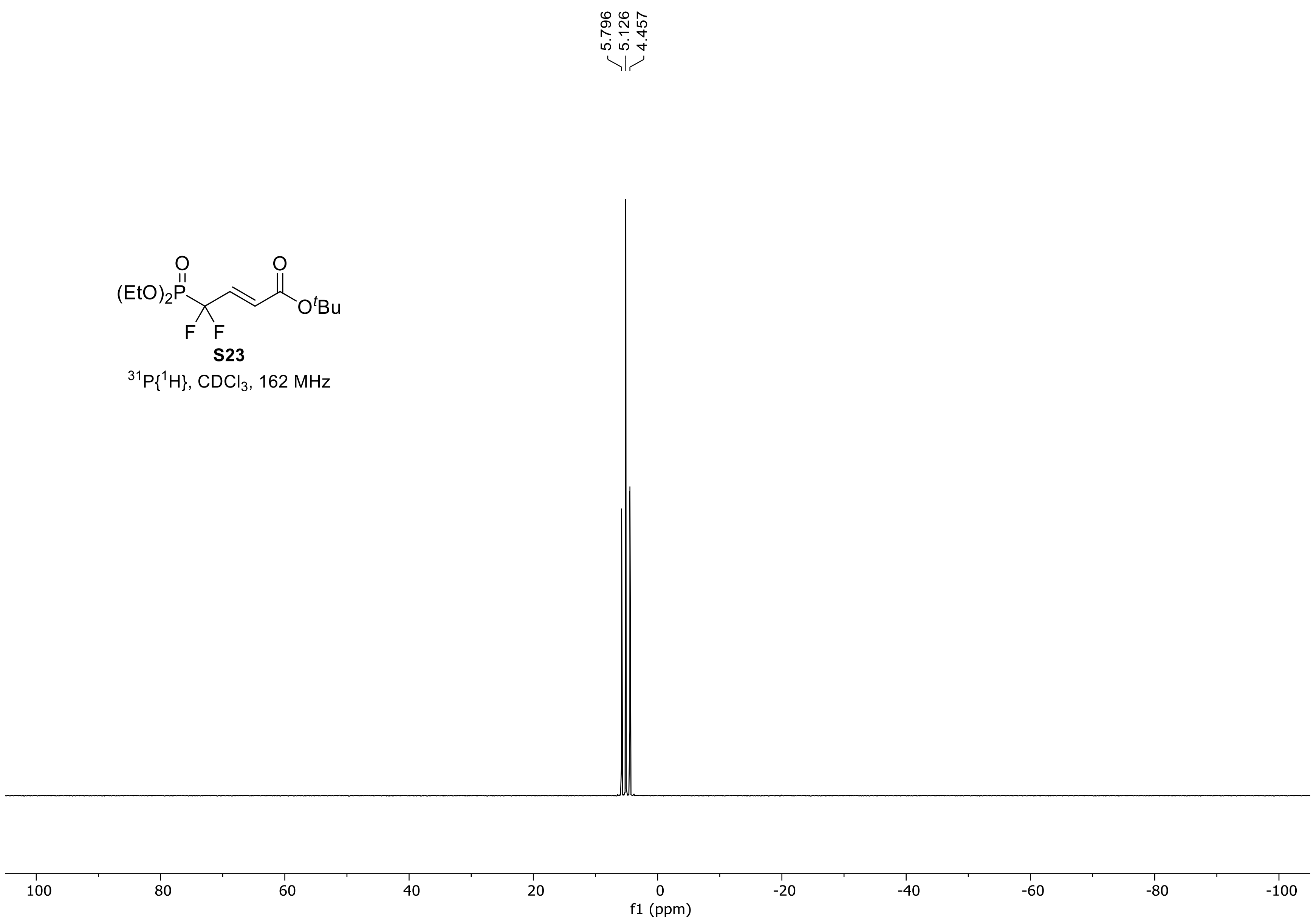
5.796
5.126
4.457
(EtO)2P
O
O
F F
O^tBu
S23
31P{1H}, CDCl3, 162 MHz
100
80
60
40
20
0
-20
-40
-60
-80
-100
f1 (ppm)

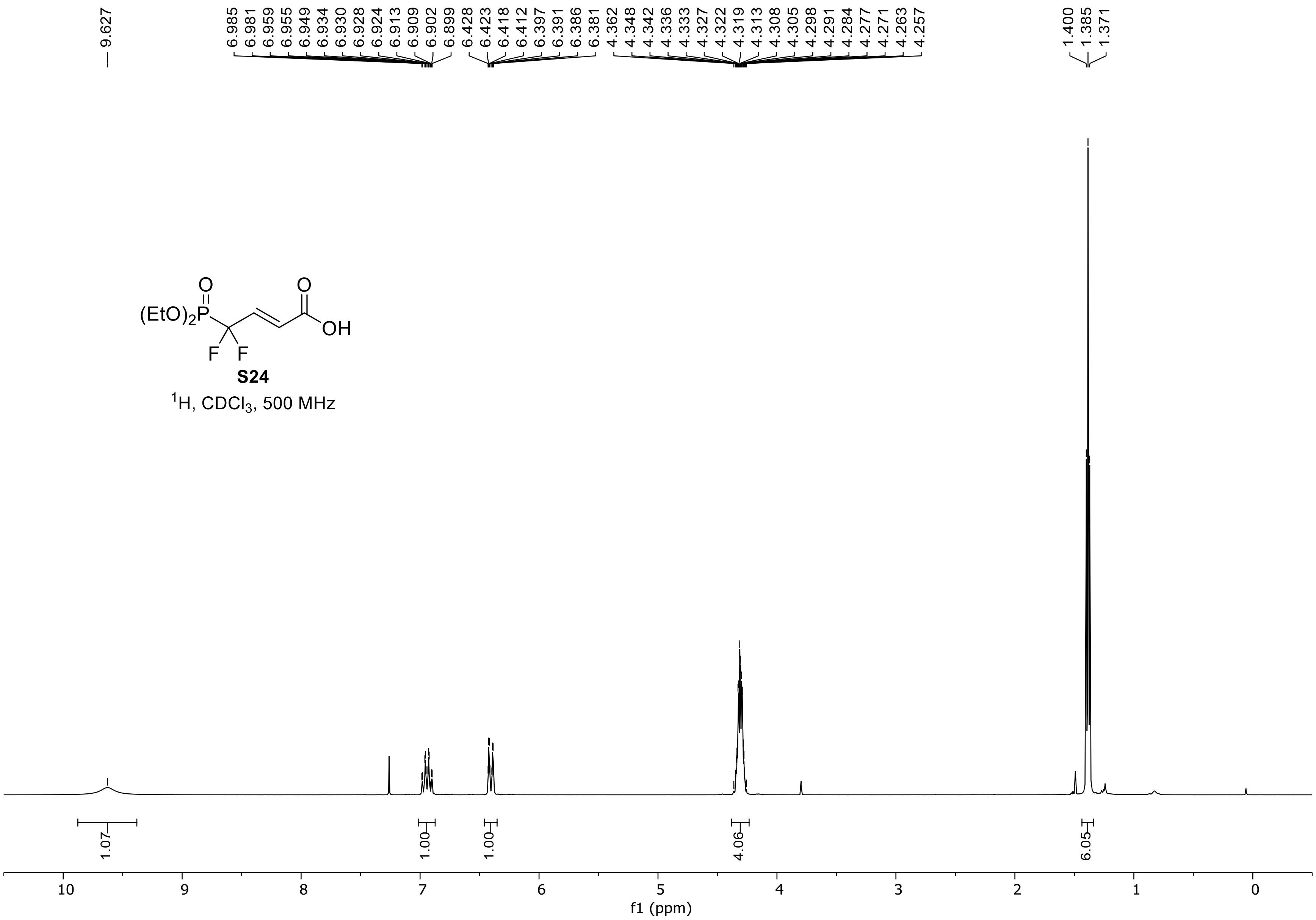

S24

^{1}H, $CDCl_3$, 500 MHz

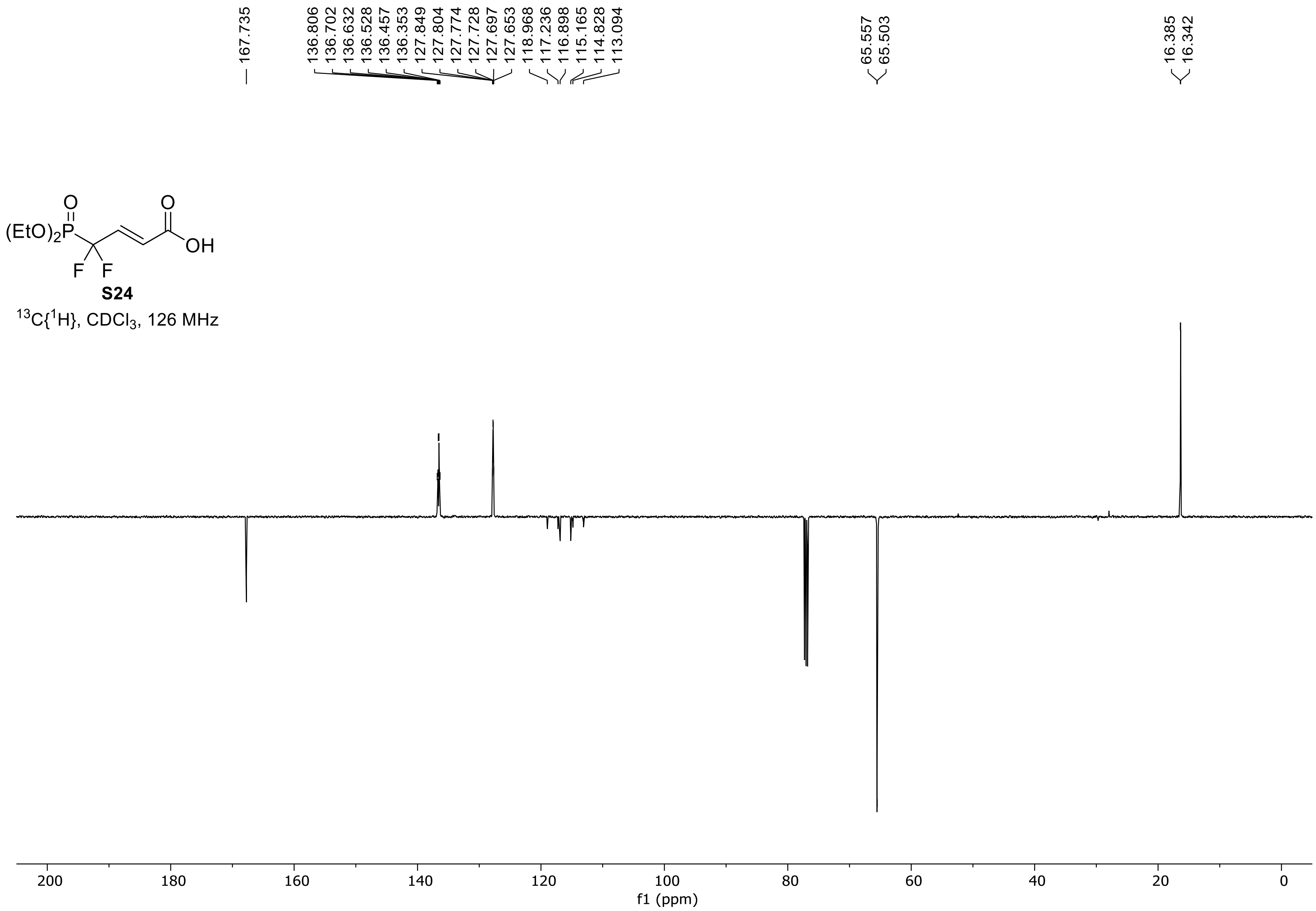
167.735
136.806
136.702
136.632
136.528
136.457
136.353
127.849
127.804
127.774
127.728
127.697
127.653
118.968
117.236
116.898
115.165
114.828
113.094
65.557
65.503
16.385
16.342
(EtO)2P
O
O
OH
F F
S24
13C{1H}, CDCl3, 126 MHz
200
180
160
140
120
100
80
60
40
20
0
f1 (ppm)

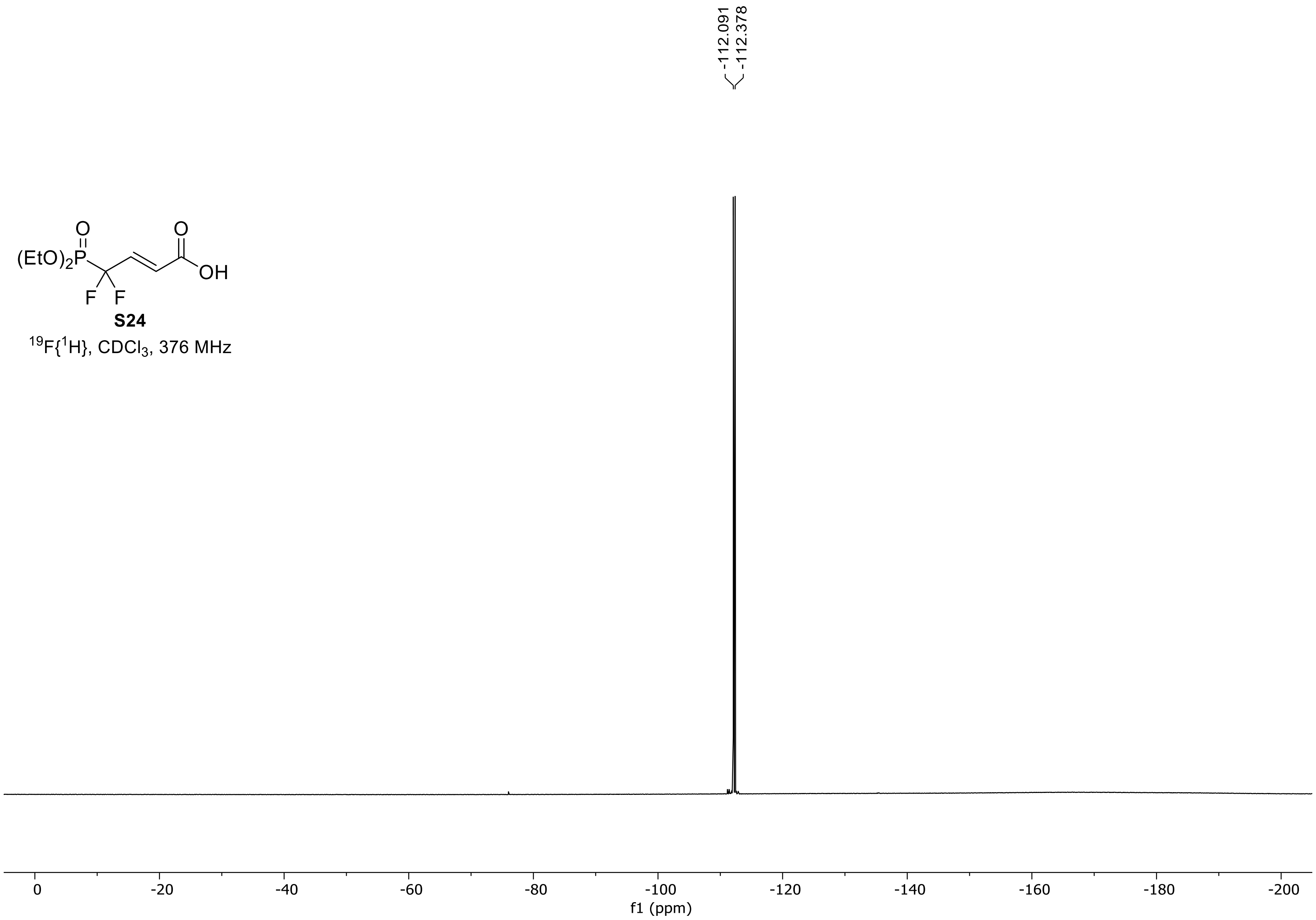

-112.091
-112.378
(EtO)2P
O
O
OH
F F
S24
19F{1H}, CDCl3, 376 MHz
0
-20
-40
-60
-80
-100
-120
-140
-160
-180
-200
f1 (ppm)

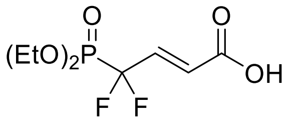

S24

$^{31}P\{^{1}H\}$, $CDCl_3$, 202 MHz

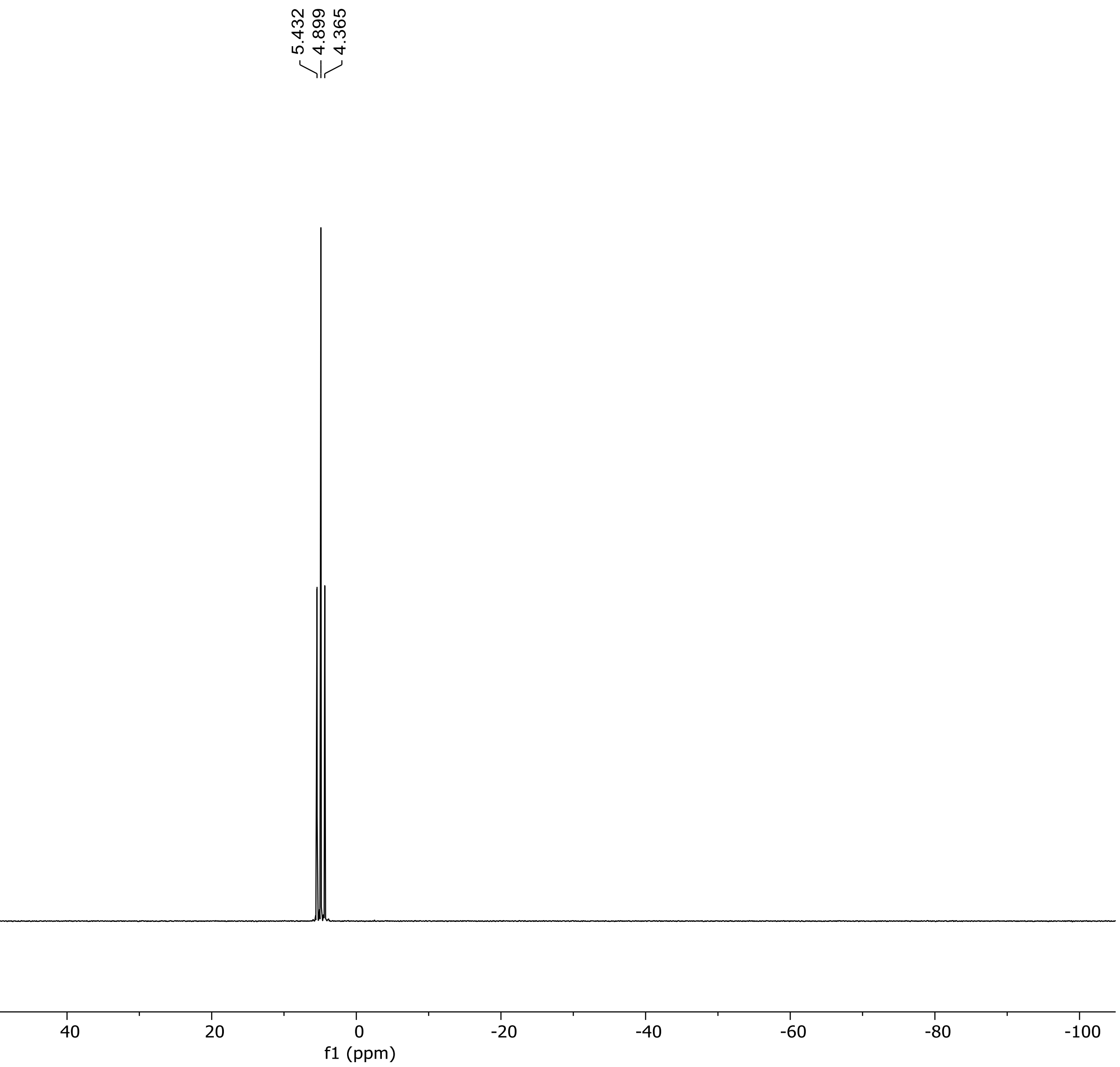

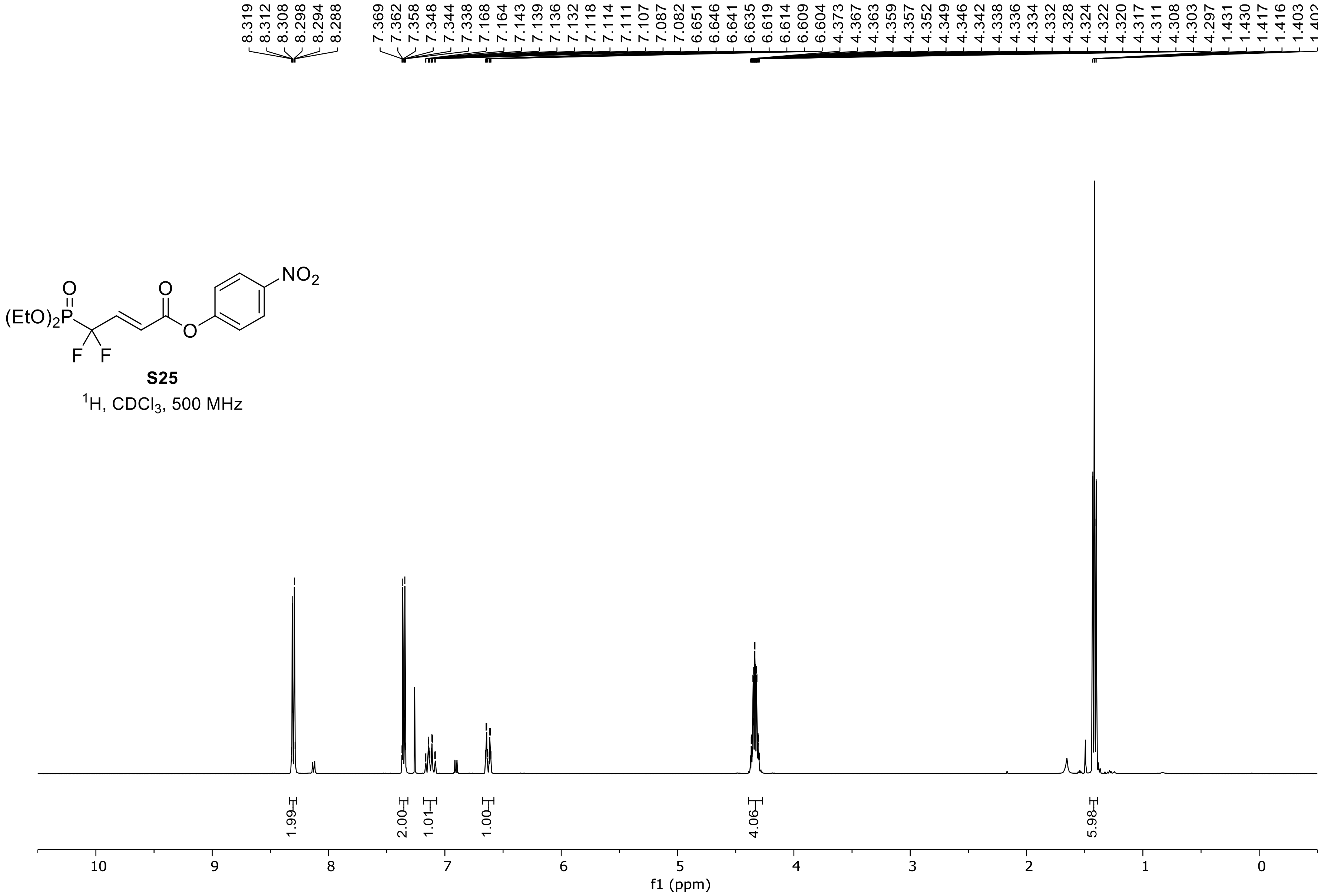

S25

^1H, $CDCl_3$, 500 MHz

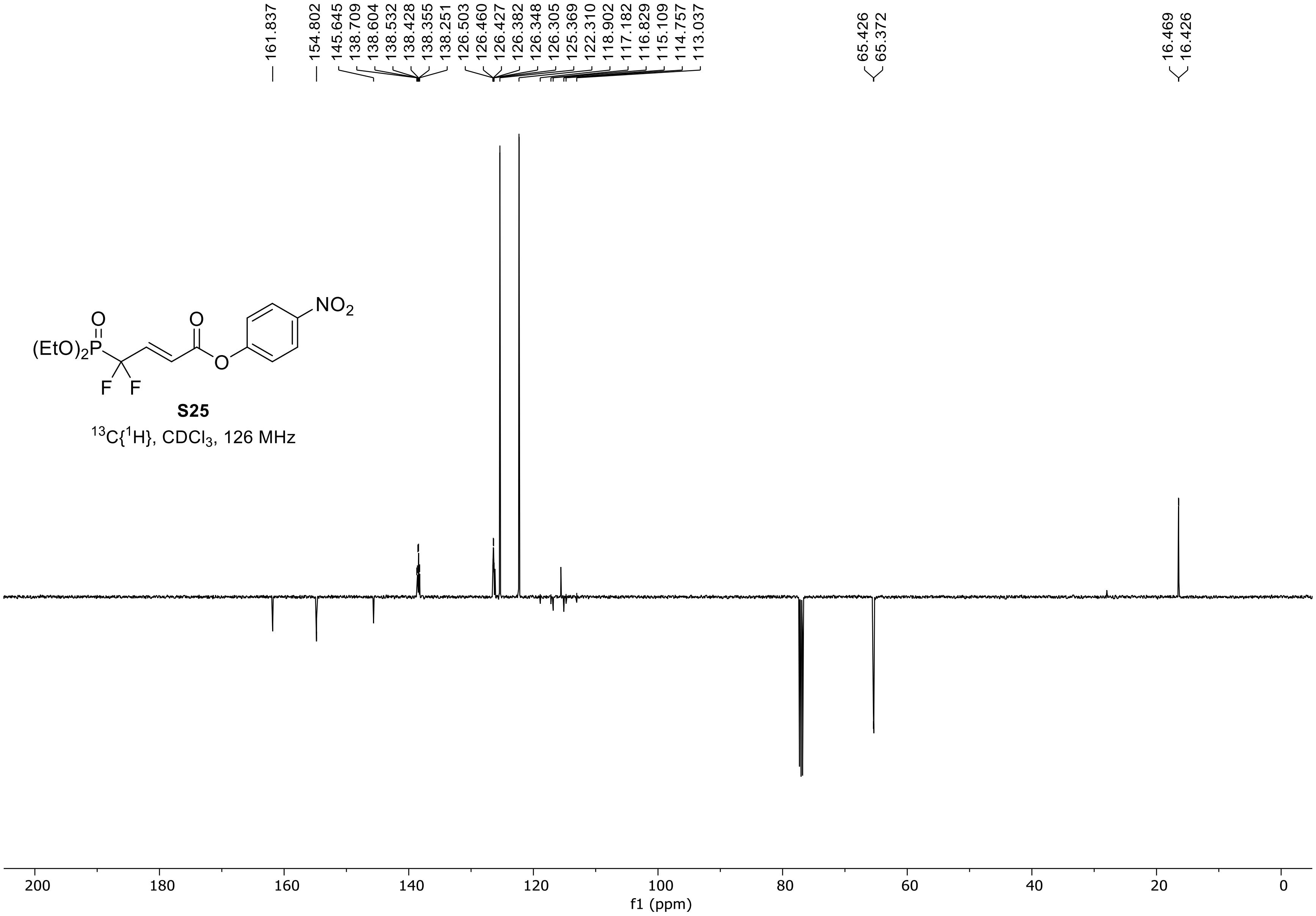

S25

$^{13}C\{^1H\}$, $CDCl_3$, 126 MHz

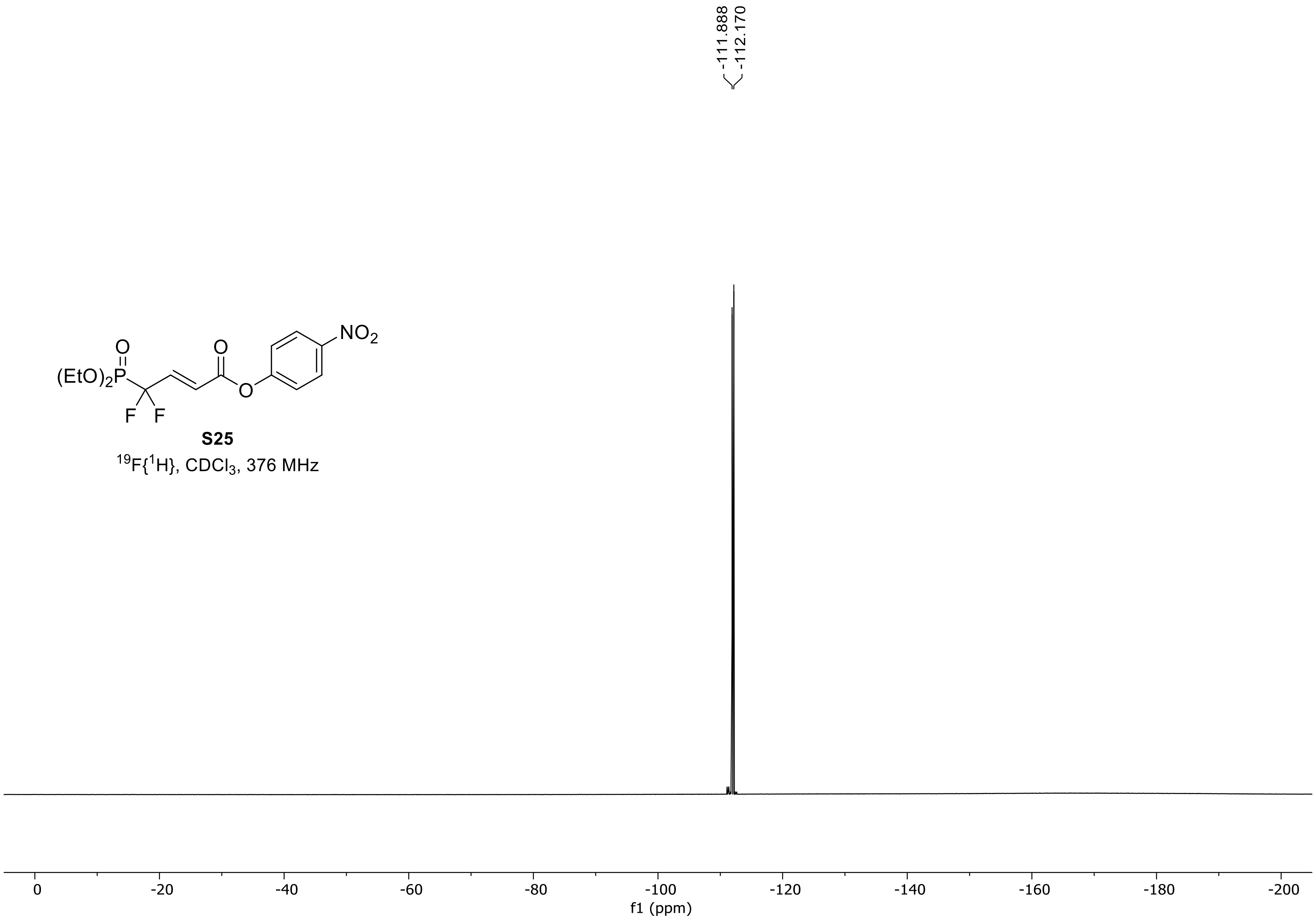

S25

$^{19}F\{^{1}H\}$, $CDCl_3$, 376 MHz

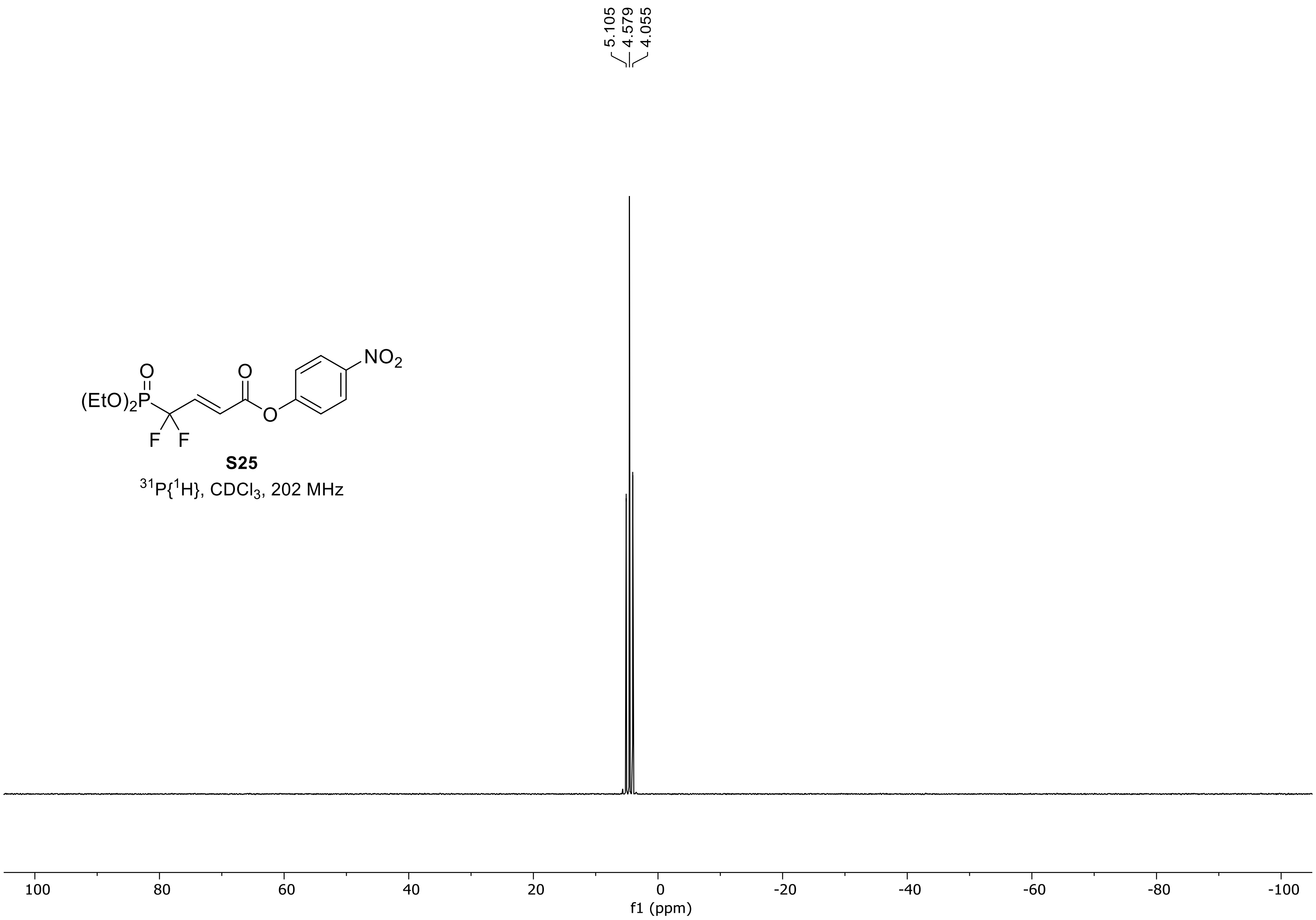
5.105
4.579
4.055
NO2
O
O
(EtO)2P
O
F F
S25
31P{1H}, CDCl3, 202 MHz
100 80 60 40 20 0 -20 -40 -60 -80 -100
f1 (ppm)

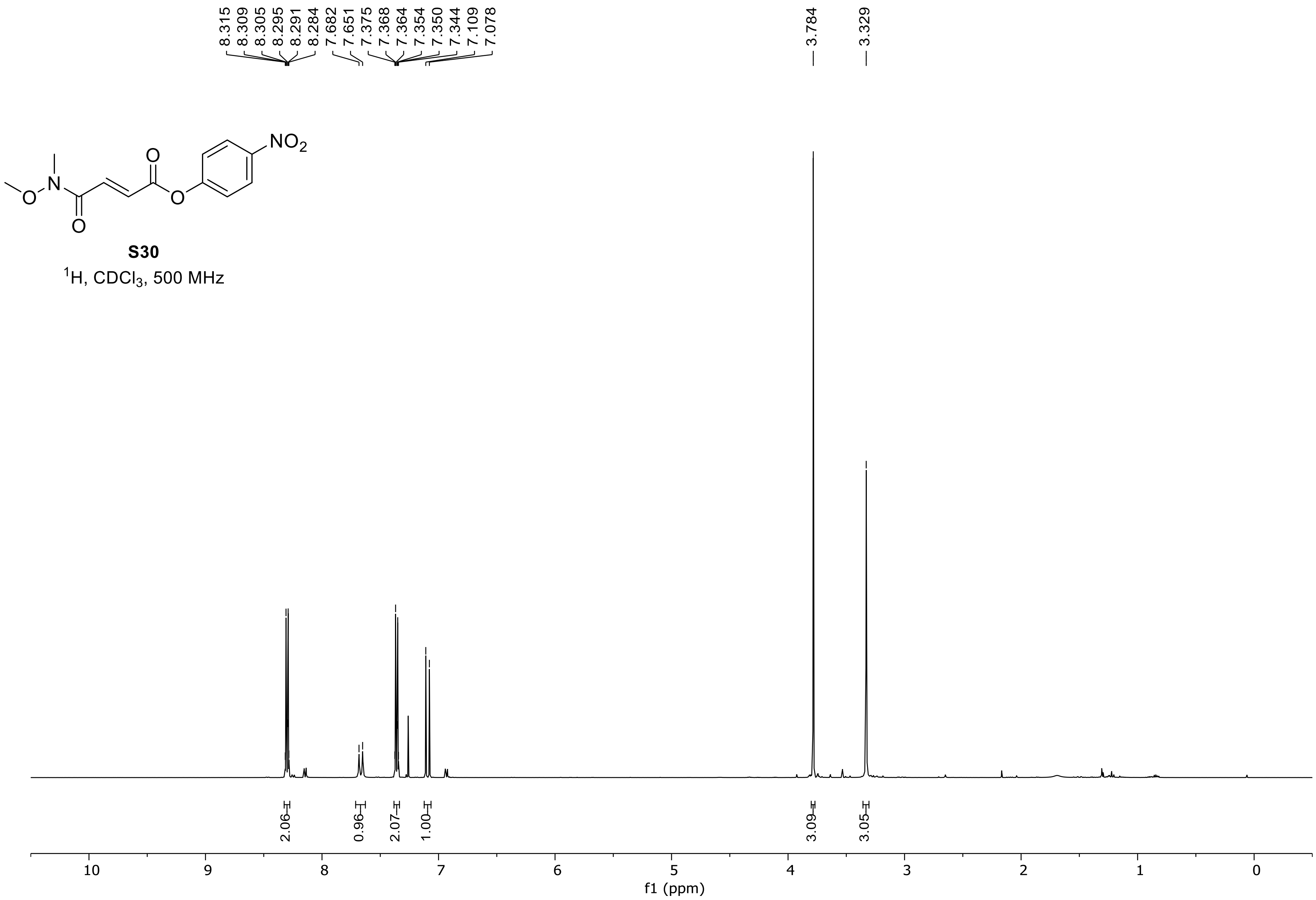
8.315
8.309
8.305
8.295
8.291
8.284
7.682
7.651
7.375
7.368
7.364
7.354
7.350
7.344
7.109
7.078
3.784
3.329
NO2
O
N
S30
1H, CDCl3, 500 MHz
2.06
0.96
2.07
1.00
3.09
3.05
10
9
8
7
6
5
4
3
2
1
0
f1 (ppm)

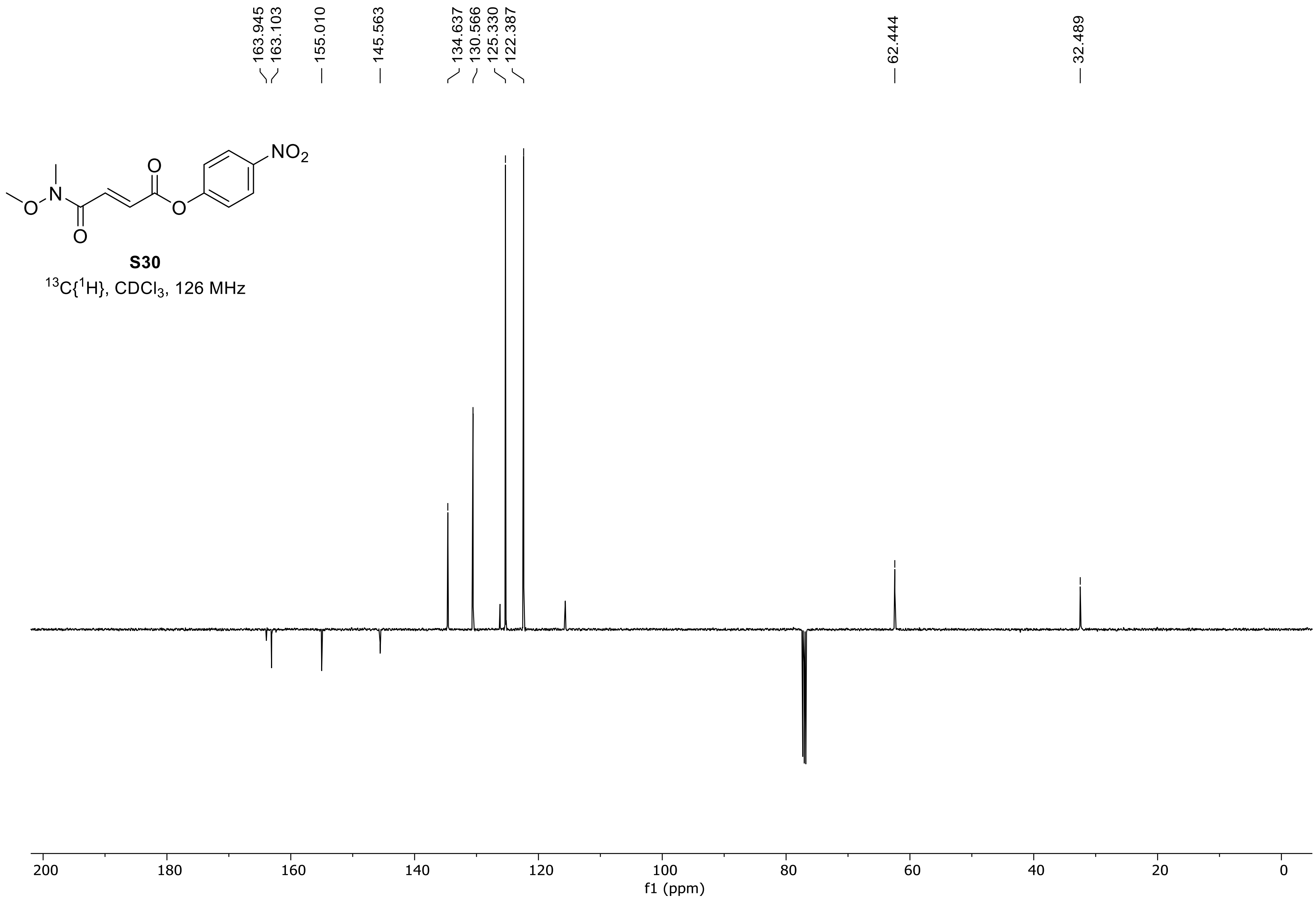
163.945
163.103
155.010
145.563
134.637
130.566
125.330
122.387
62.444
32.489
NO2
O
N
O
O
O
S30
13C{1H}, CDCl3, 126 MHz
200
180
160
140
120
100
80
60
40
20
0
f1 (ppm)

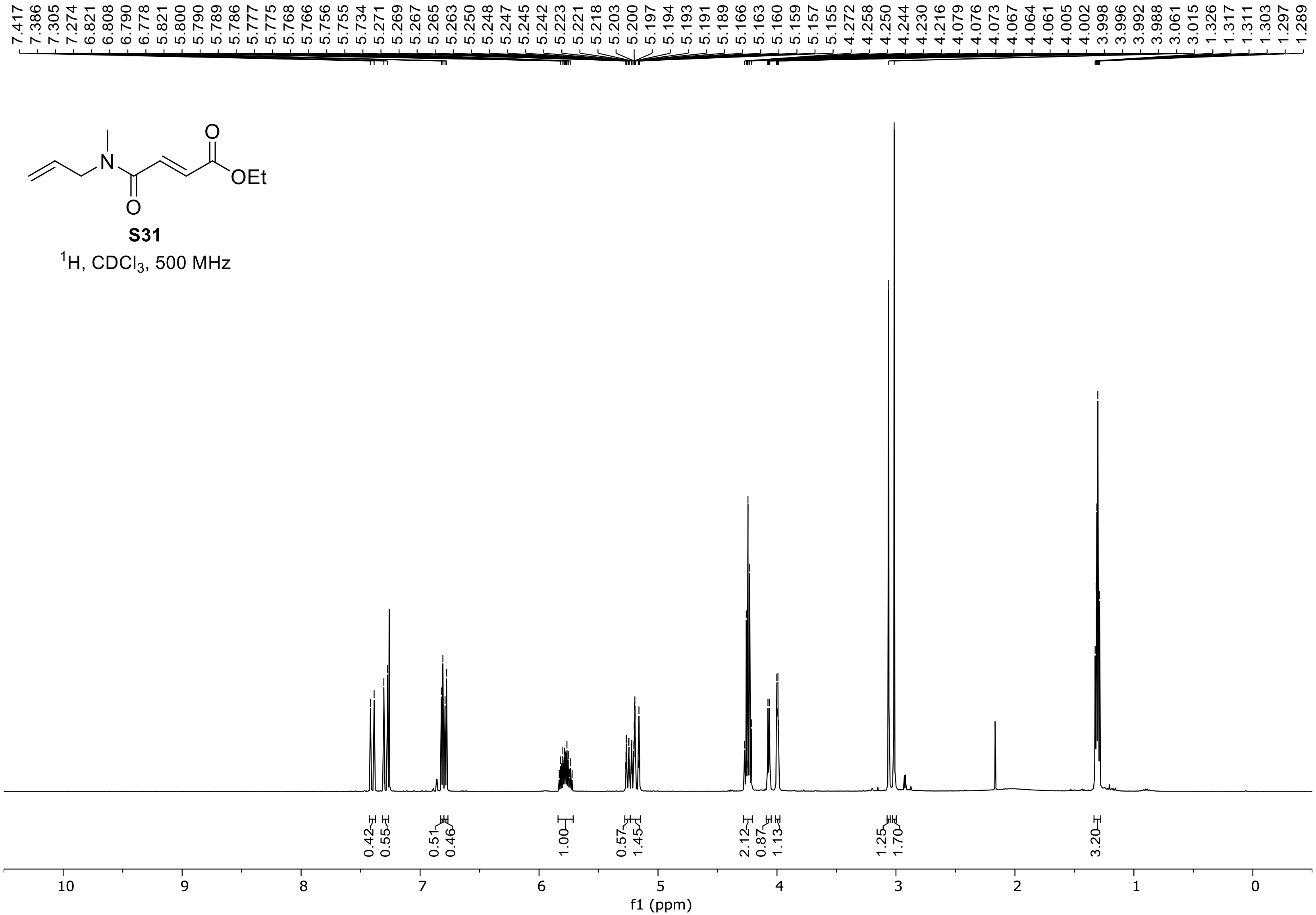
7.417
7.386
7.305
7.274
6.821
6.808
6.790
6.778
5.821
5.800
5.790
5.789
5.786
5.777
5.775
5.768
5.766
5.756
5.755
5.734
5.271
5.269
5.267
5.265
5.263
5.250
5.248
5.247
5.245
5.242
5.223
5.221
5.218
5.203
5.200
5.197
5.194
5.193
5.191
5.189
5.166
5.163
5.160
5.159
5.157
5.155
4.272
4.258
4.250
4.244
4.230
4.216
4.079
4.076
4.073
4.067
4.064
4.061
4.005
4.002
3.998
3.996
3.992
3.988
3.061
3.015
1.326
1.317
1.311
1.303
1.297
1.289
O
N
OEt
O
S31
^{1}H, $CDCl_3$, 500 MHz
0.42
0.55
0.51
0.46
1.00
0.57
1.45
2.12
0.87
1.13
1.25
1.70
3.20
10
9
8
7
6
5
4
3
2
1
0
f1 (ppm)

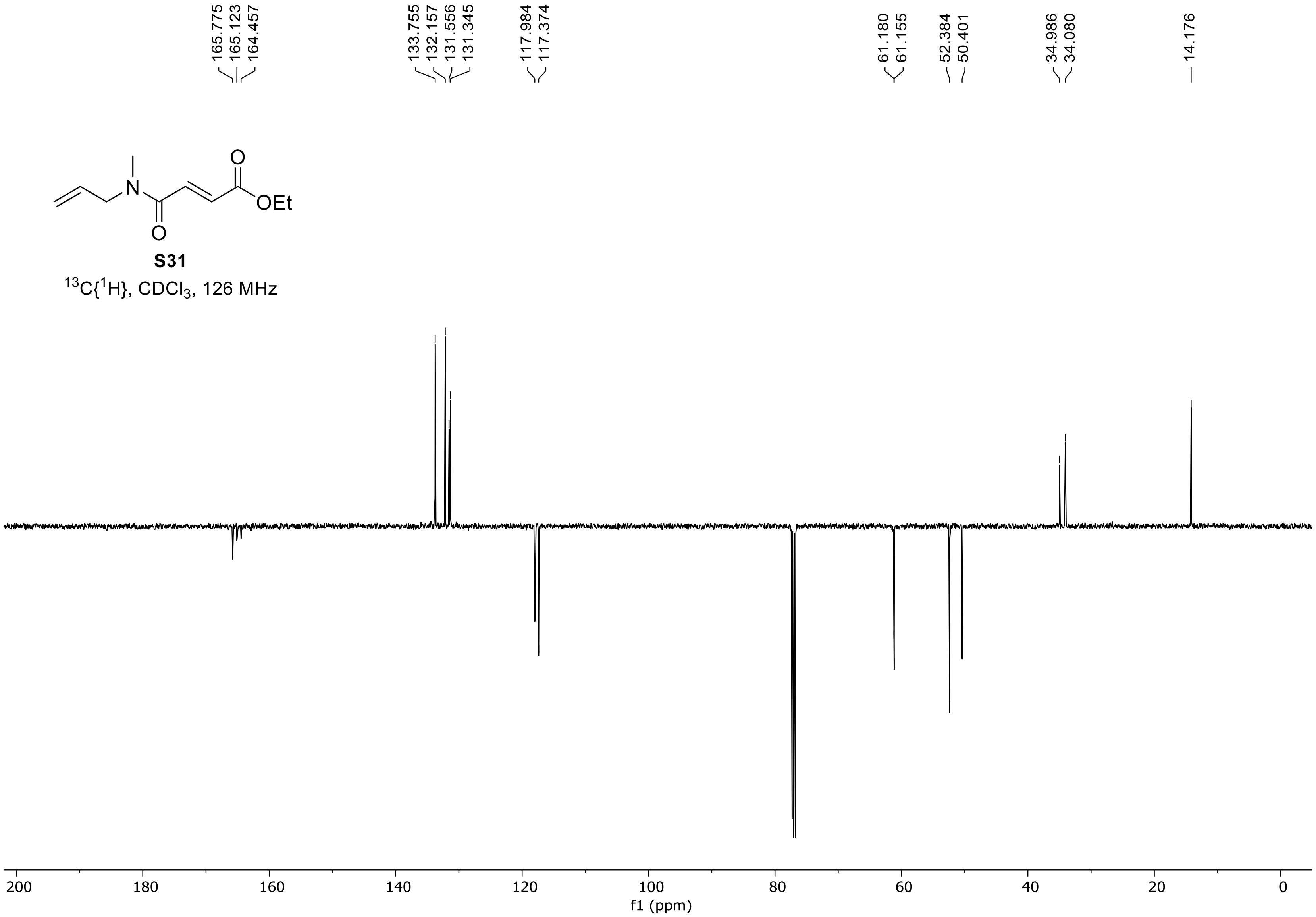

S31

$^{13}C\{^{1}H\}$, $CDCl_3$, 126 MHz

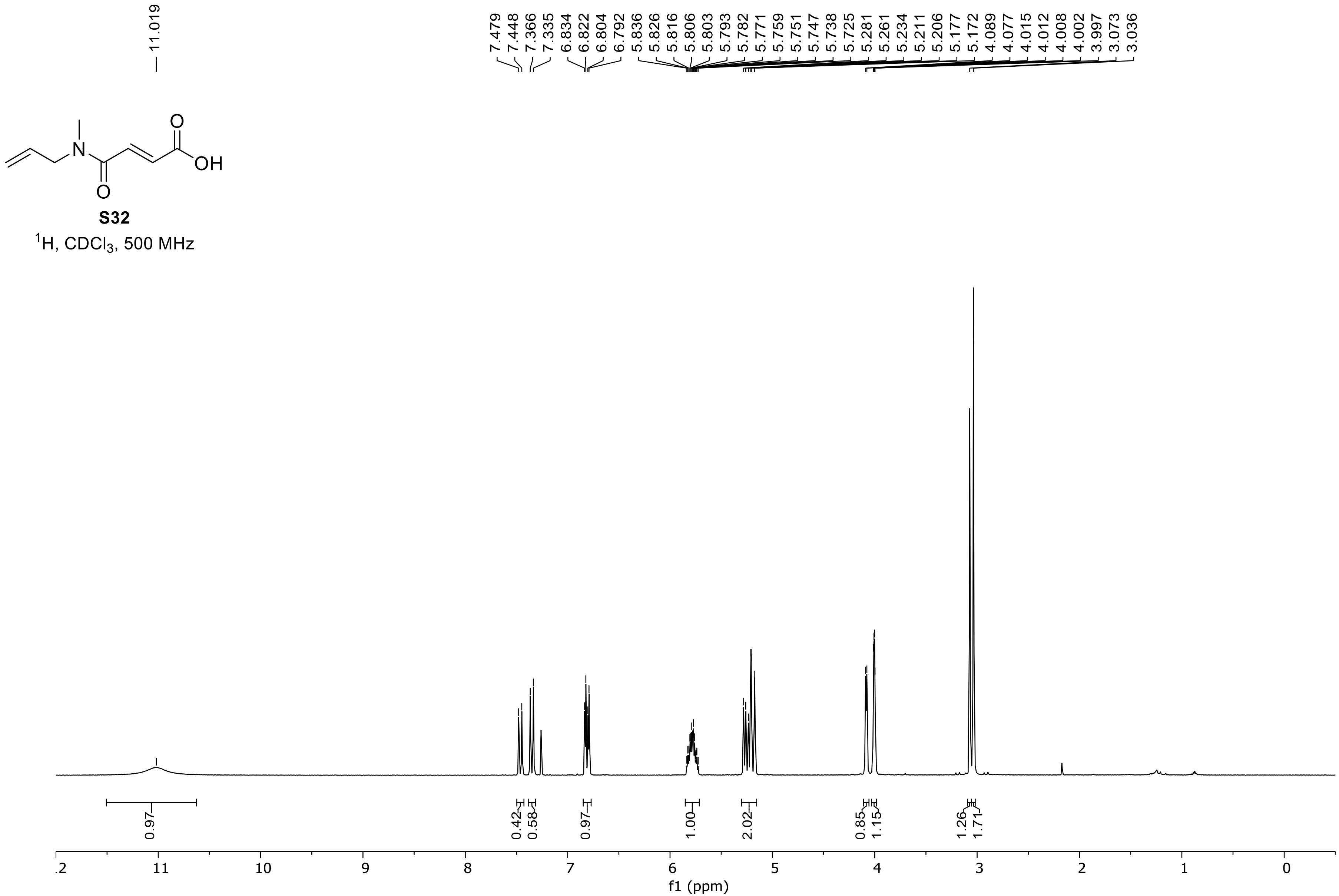

11.019
7.479
7.448
7.366
7.335
6.834
6.822
6.804
6.792
5.836
5.826
5.816
5.806
5.803
5.793
5.782
5.771
5.759
5.751
5.747
5.738
5.725
5.281
5.261
5.234
5.211
5.206
5.177
5.172
4.089
4.077
4.015
4.012
4.008
4.002
3.997
3.073
3.036
O
N
OH
O
S32
^{1}H, $CDCl_3$, 500 MHz
0.97
0.42
0.58
0.97
1.00
2.02
0.85
1.15
1.26
1.71
11
10
9
8
7
6
5
4
3
2
1
0
f1 (ppm)

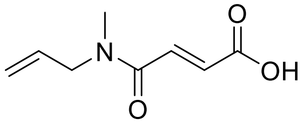

S32

$^{13}C\{^1H\}$, $CDCl_3$, 126 MHz

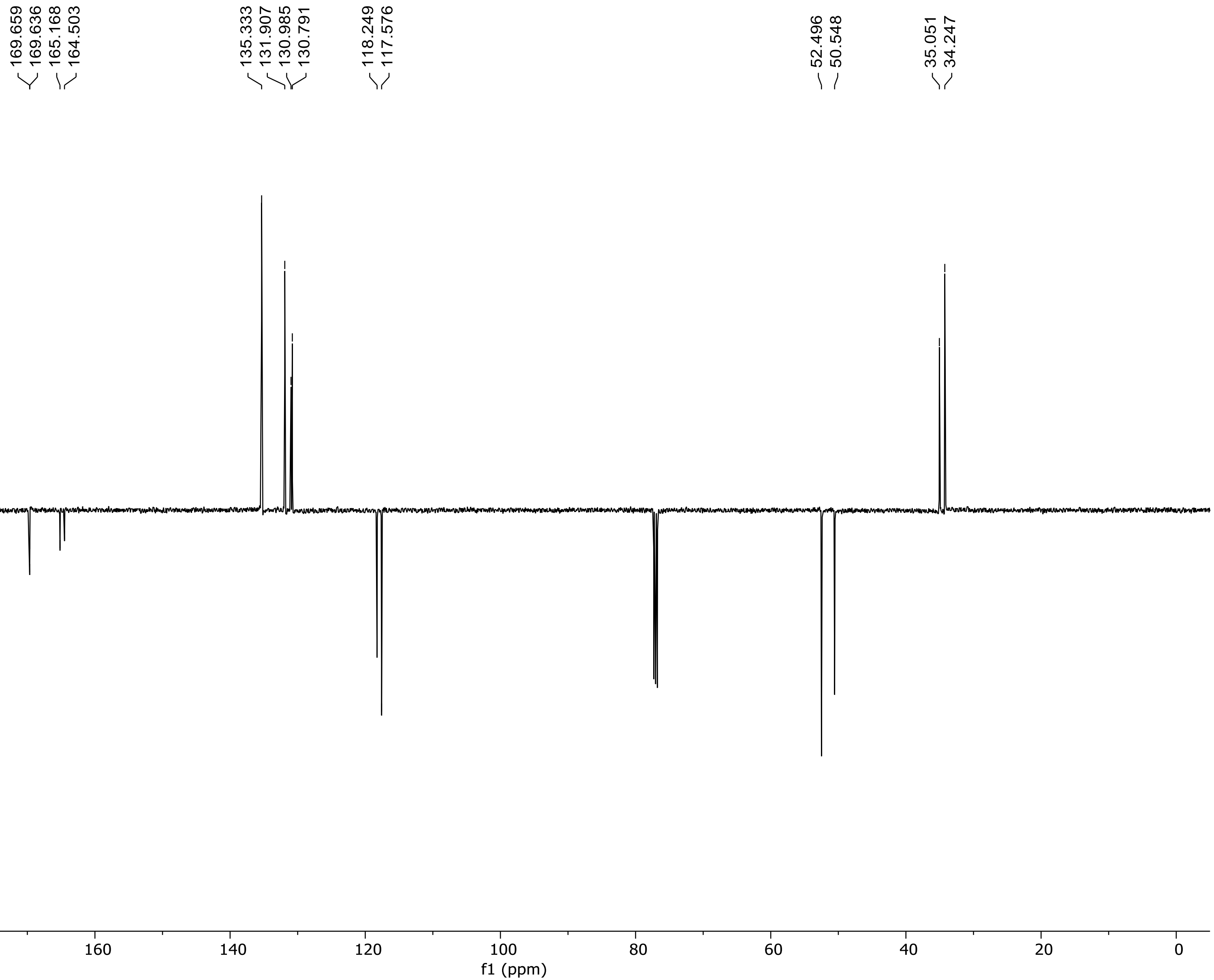

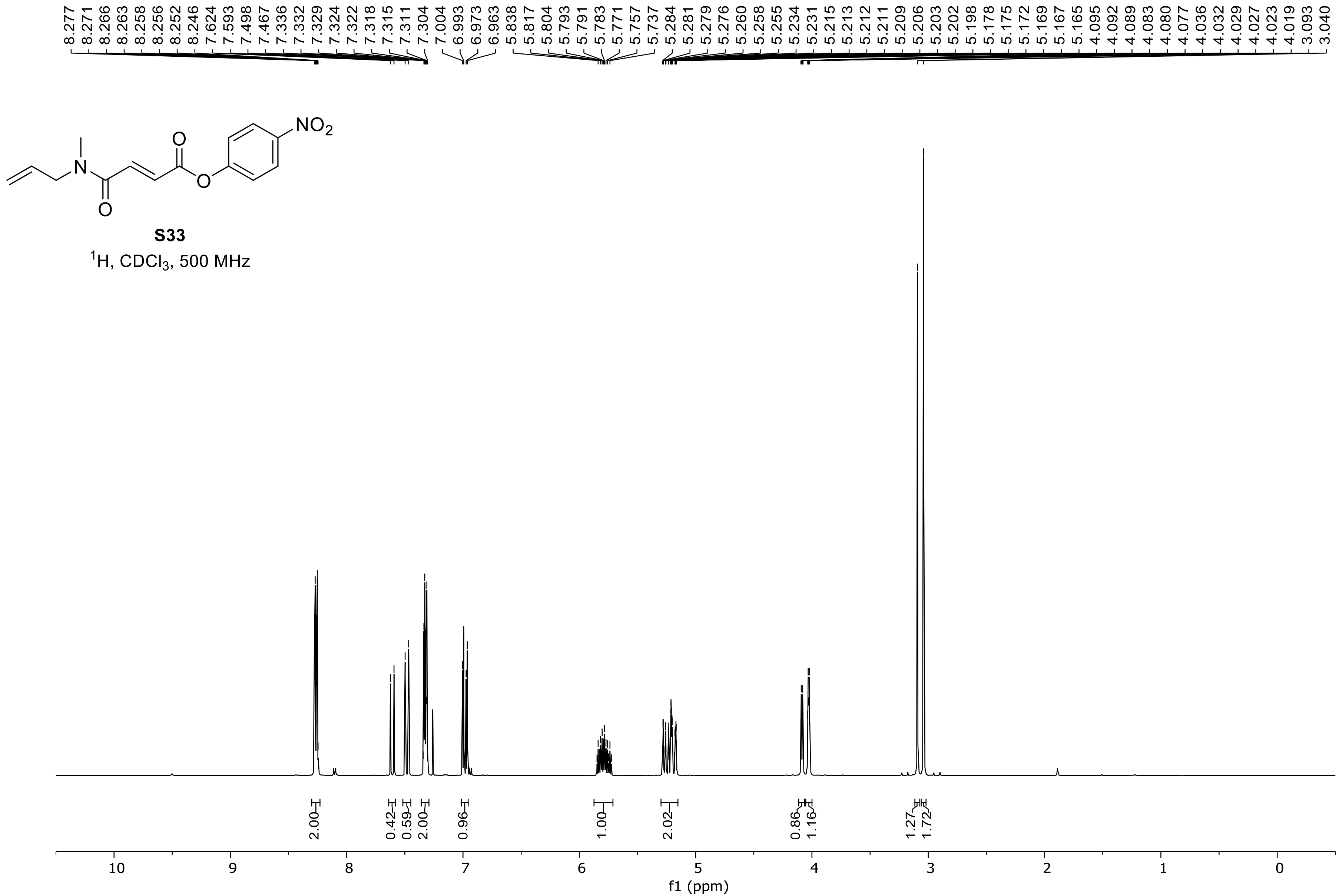

S33
^{1}H, $CDCl_3$, 500 MHz
f1 (ppm)

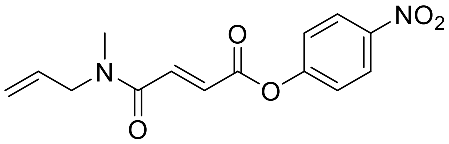

S33

$^{13}C\{^1H\}$, $CDCl_3$, 126 MHz

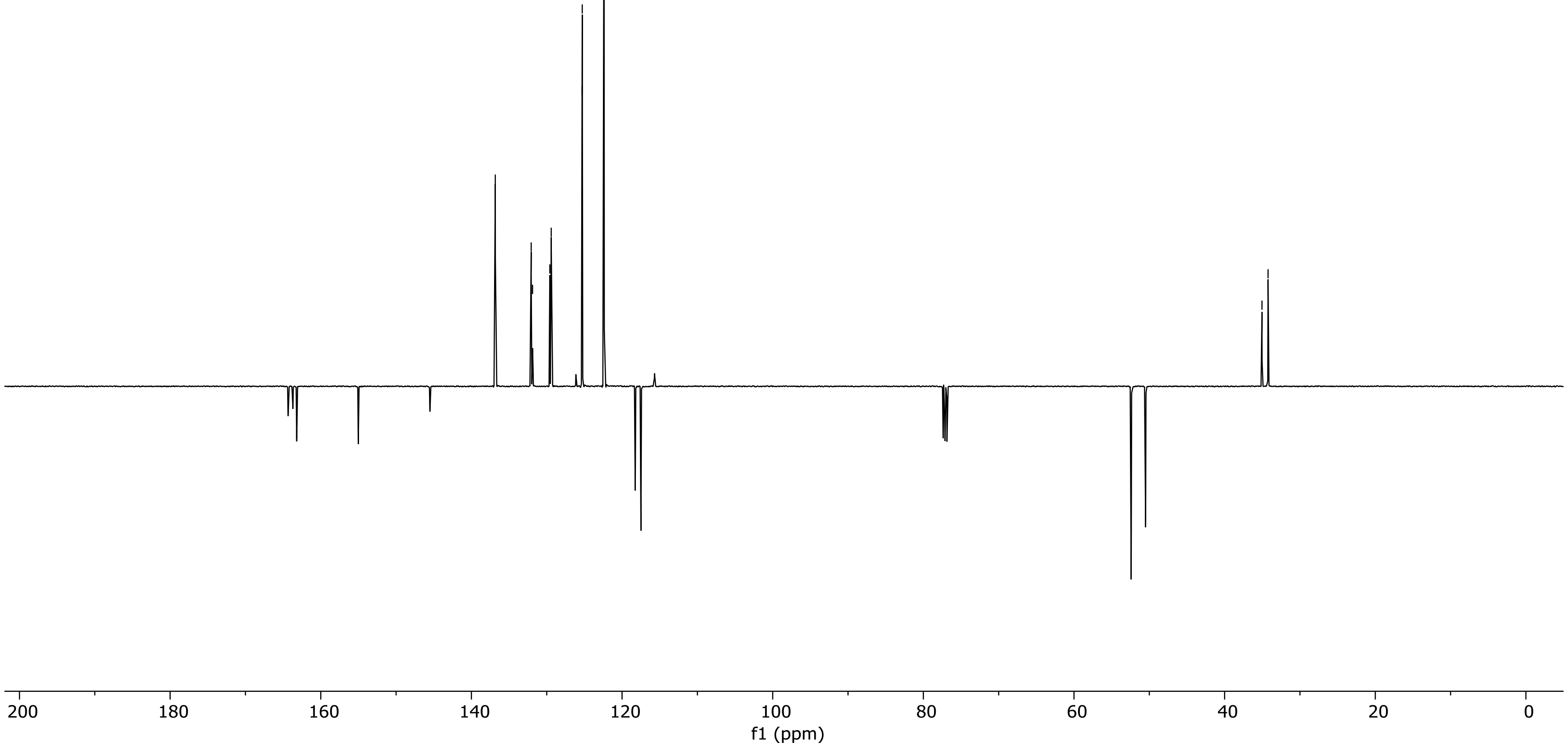

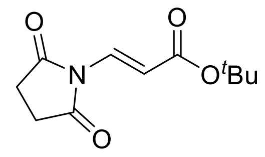

S34

^1H, $CDCl_3$, 500 MHz

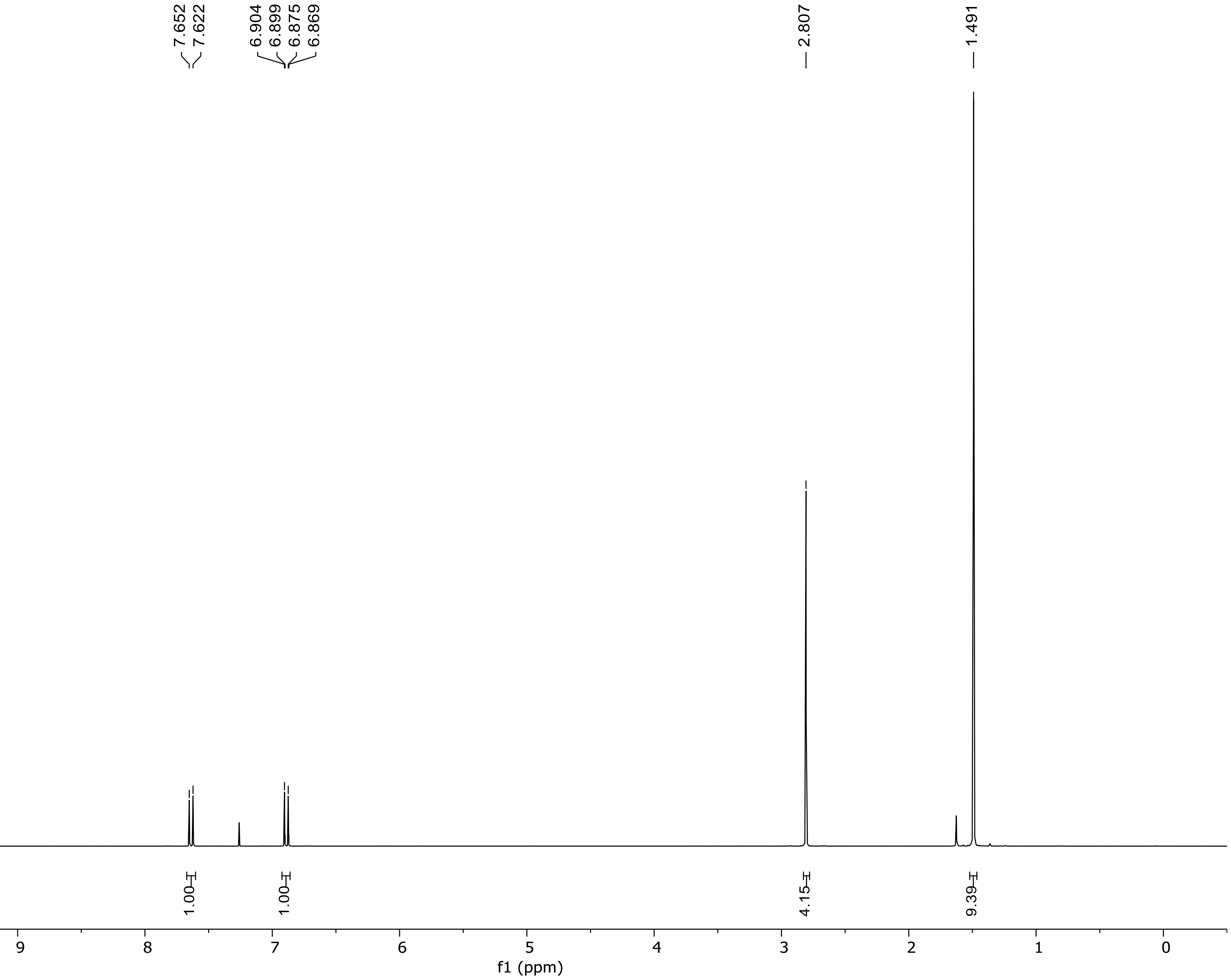

S34

$^{13}C\{^1H\}$, $CDCl_3$ 126 MHz

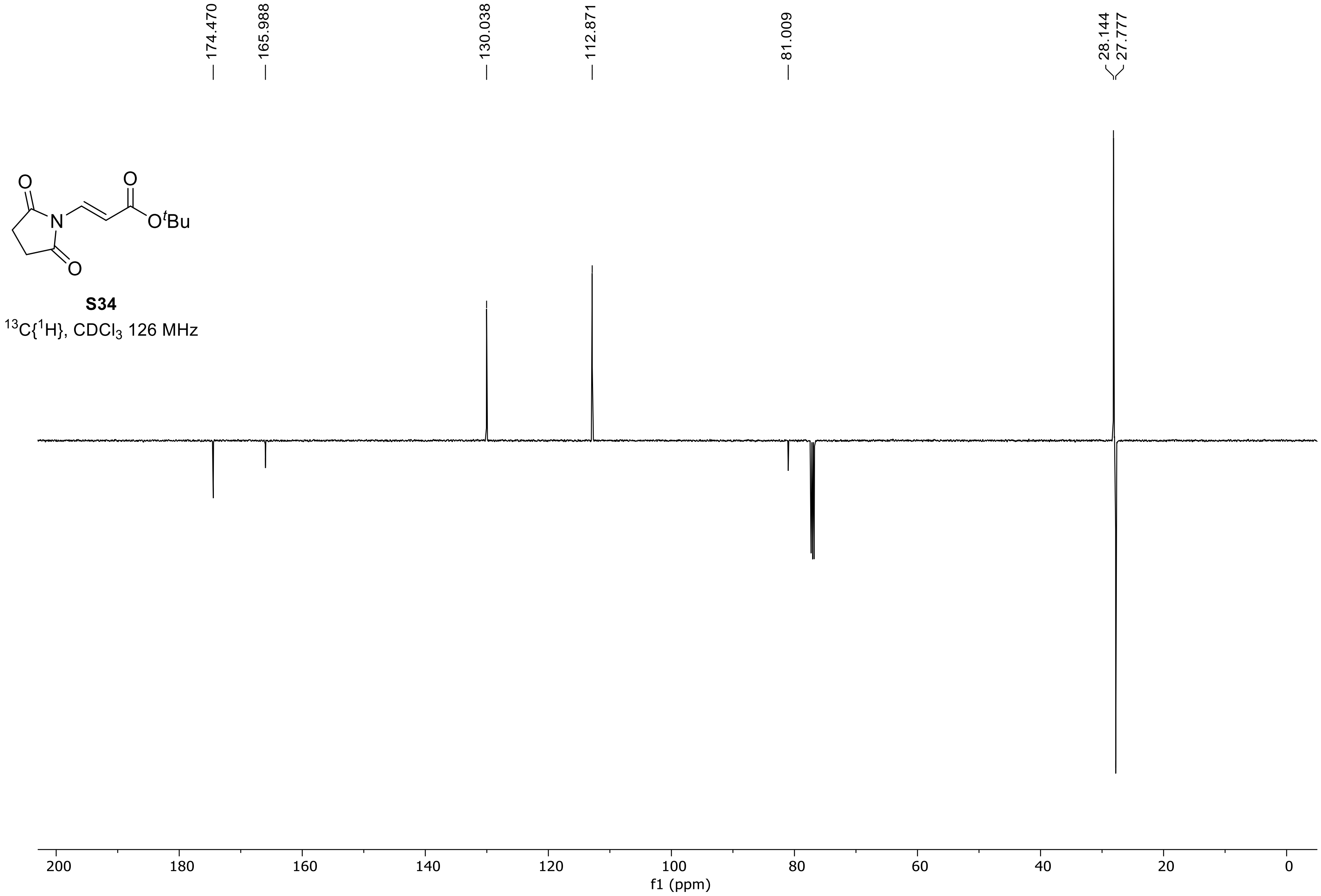

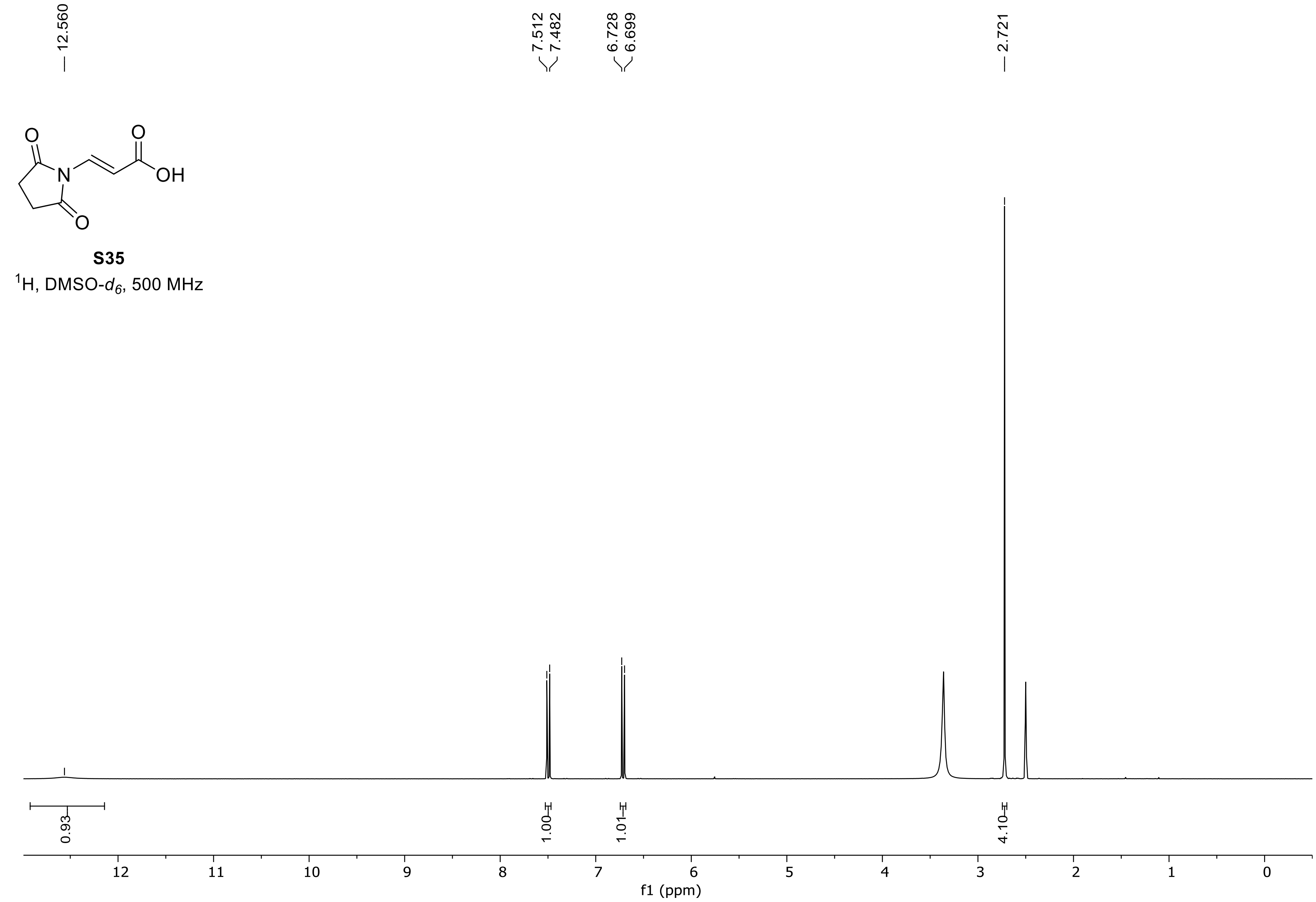

S35

^1H, DMSO-d_6, 500 MHz

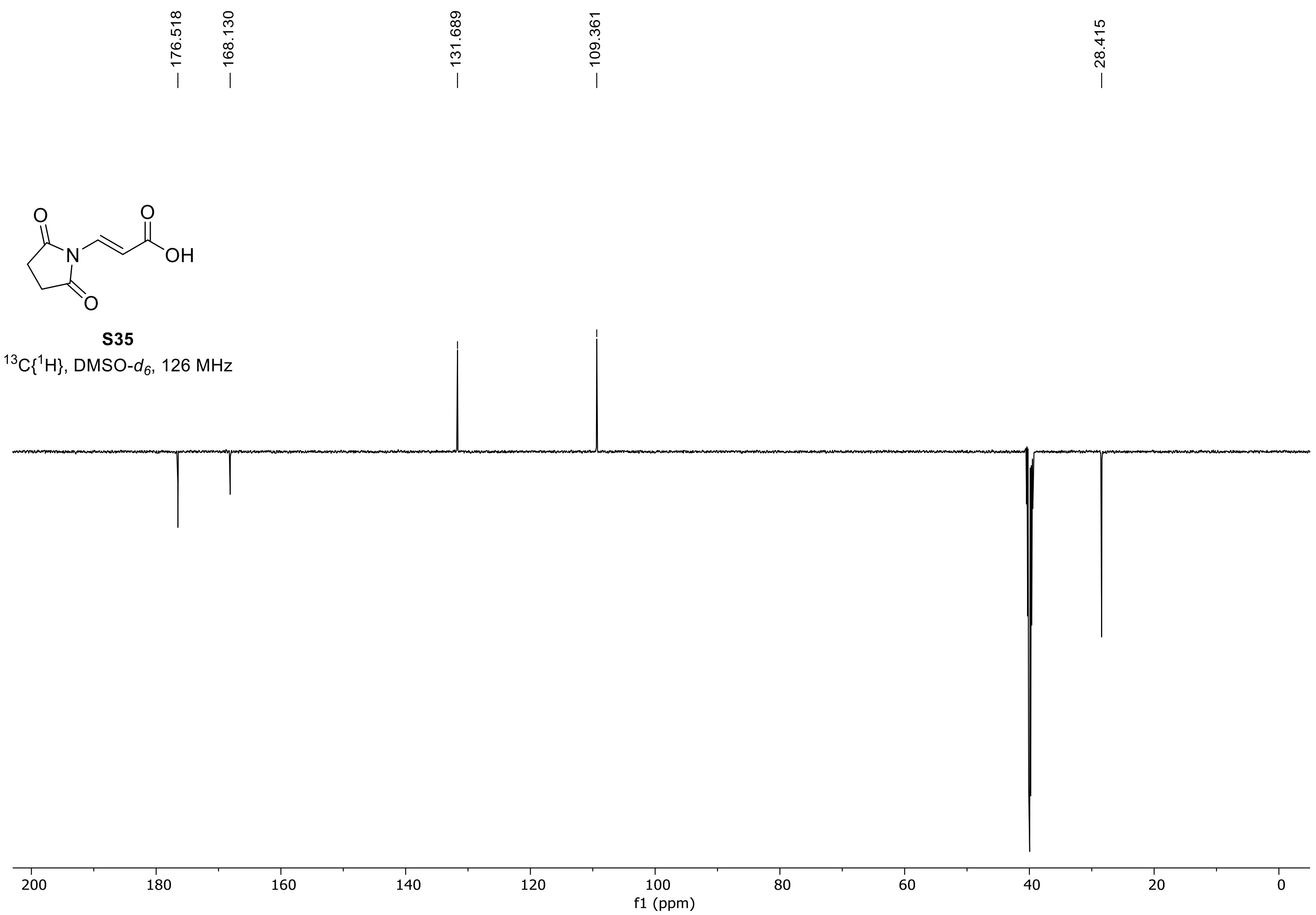

176.518
168.130
131.689
109.361
28.415
O
O
N
OH
O
S35
$^{13}C\{^1H\}$, DMSO-d_6, 126 MHz
200
180
160
140
120
100
80
60
40
20
0
f1 (ppm)

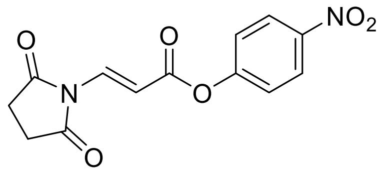

S36

^{1}H, $CDCl_3$, 500 MHz

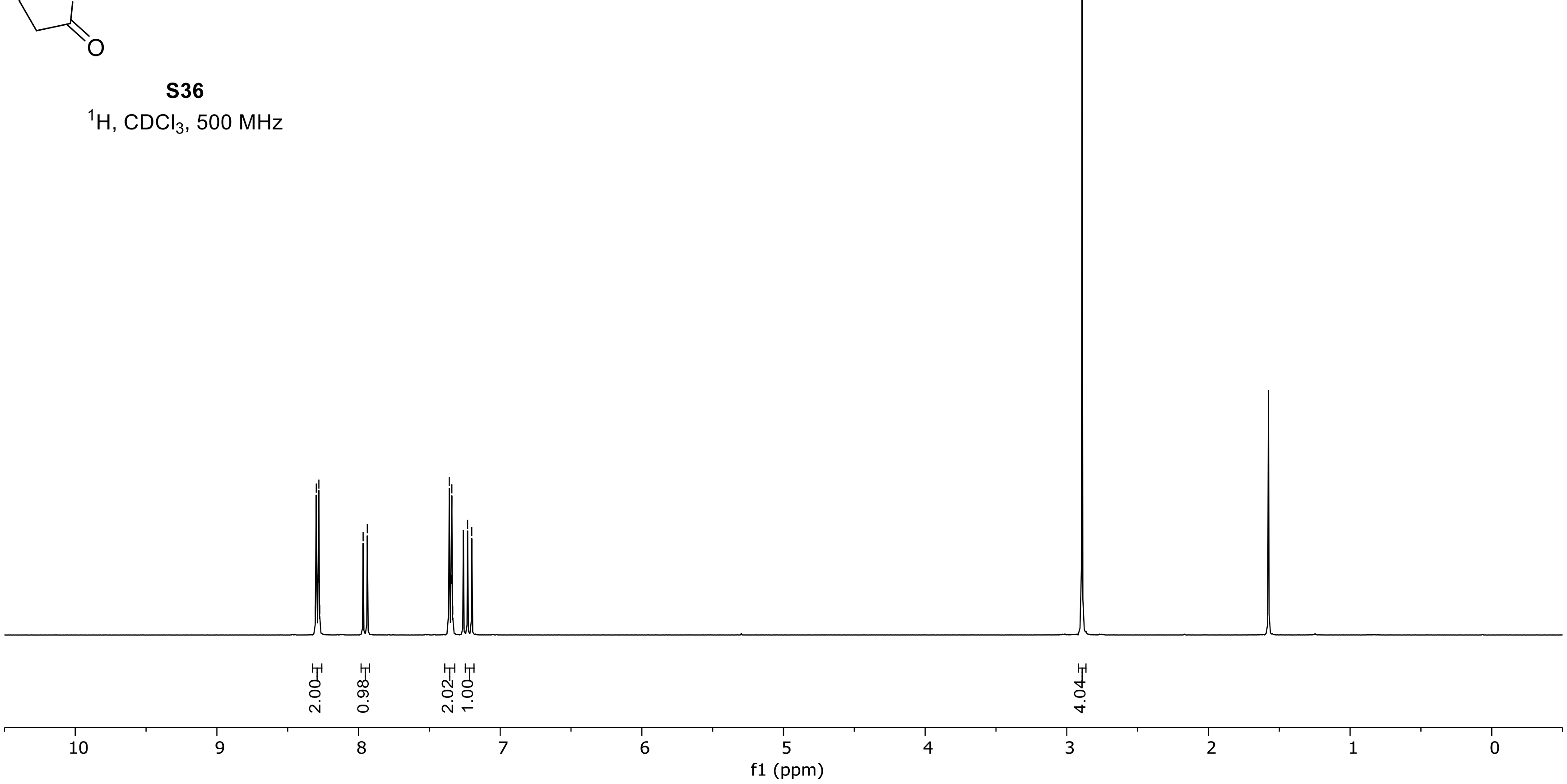

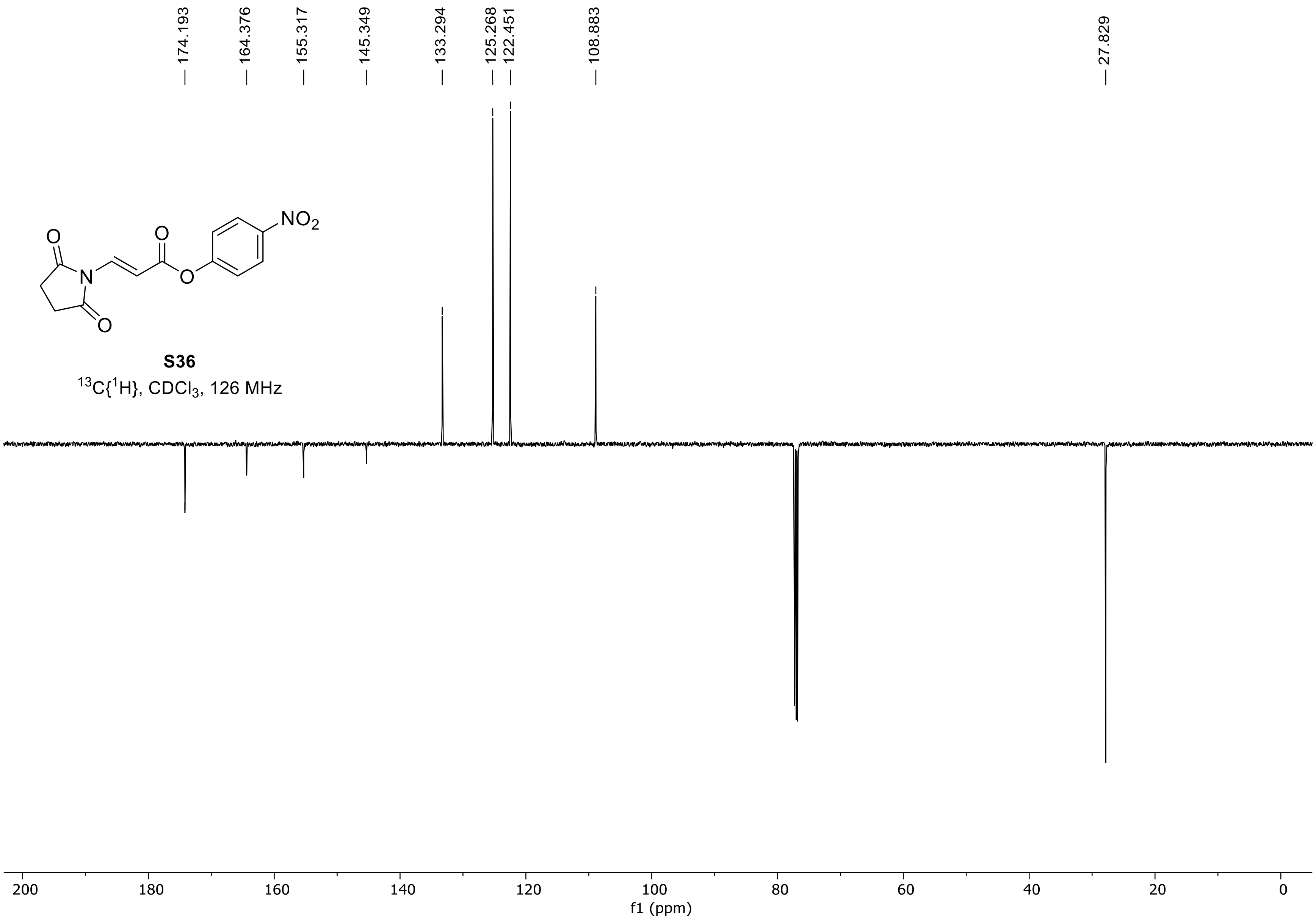

S36

$^{13}C\{^{1}H\}$, $CDCl_3$, 126 MHz

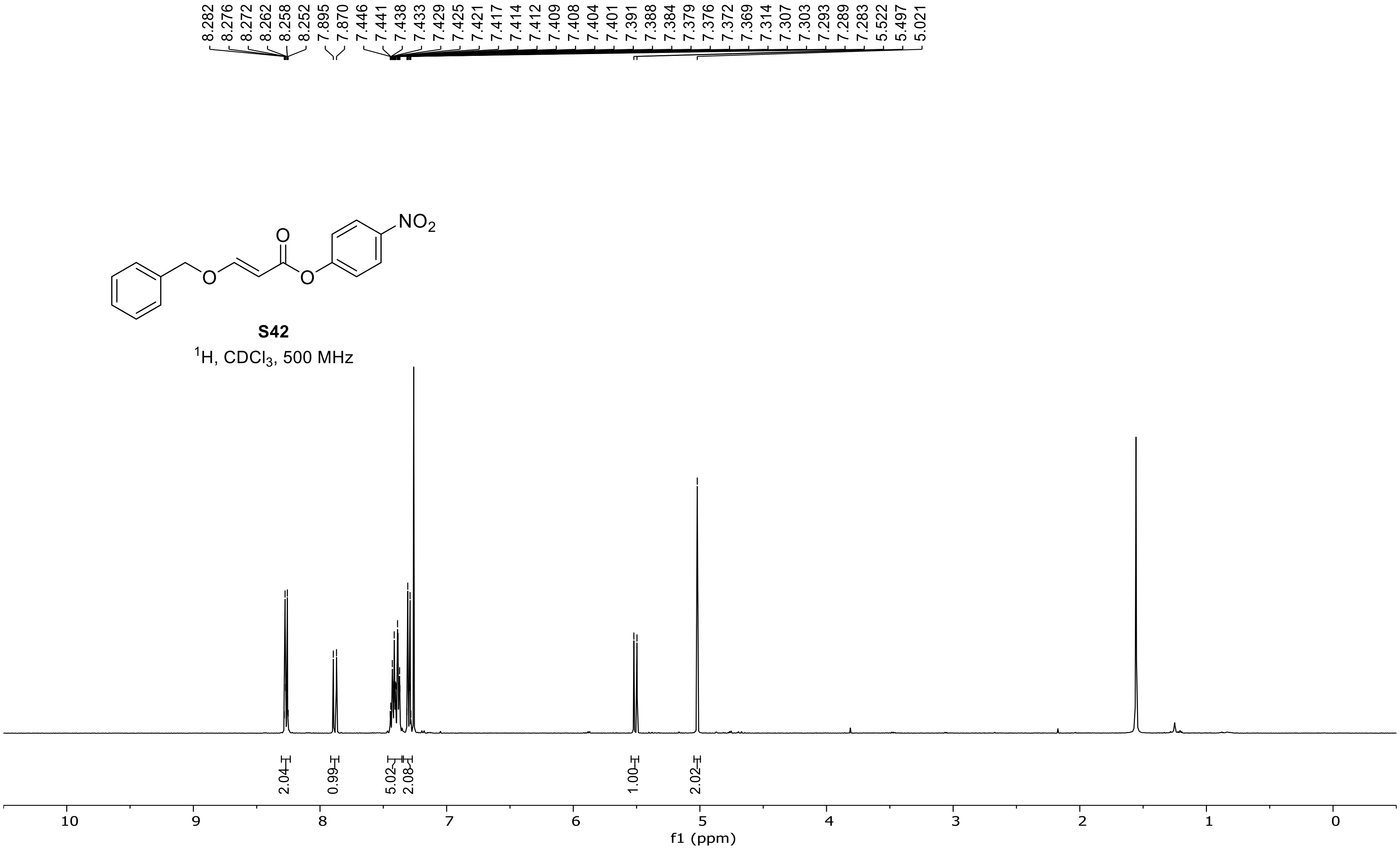

8.282
8.276
8.272
8.262
8.258
8.252
7.895
7.870
7.446
7.441
7.438
7.433
7.429
7.425
7.421
7.417
7.414
7.412
7.409
7.408
7.404
7.401
7.391
7.388
7.384
7.379
7.376
7.372
7.369
7.314
7.307
7.303
7.293
7.289
7.283
5.522
5.497
5.021
NO2
O
O
O
S42
1H, CDCl3, 500 MHz
2.04
0.99
5.02
2.08
1.00
2.02
10
9
8
7
6
5
4
3
2
1
0
f1 (ppm)

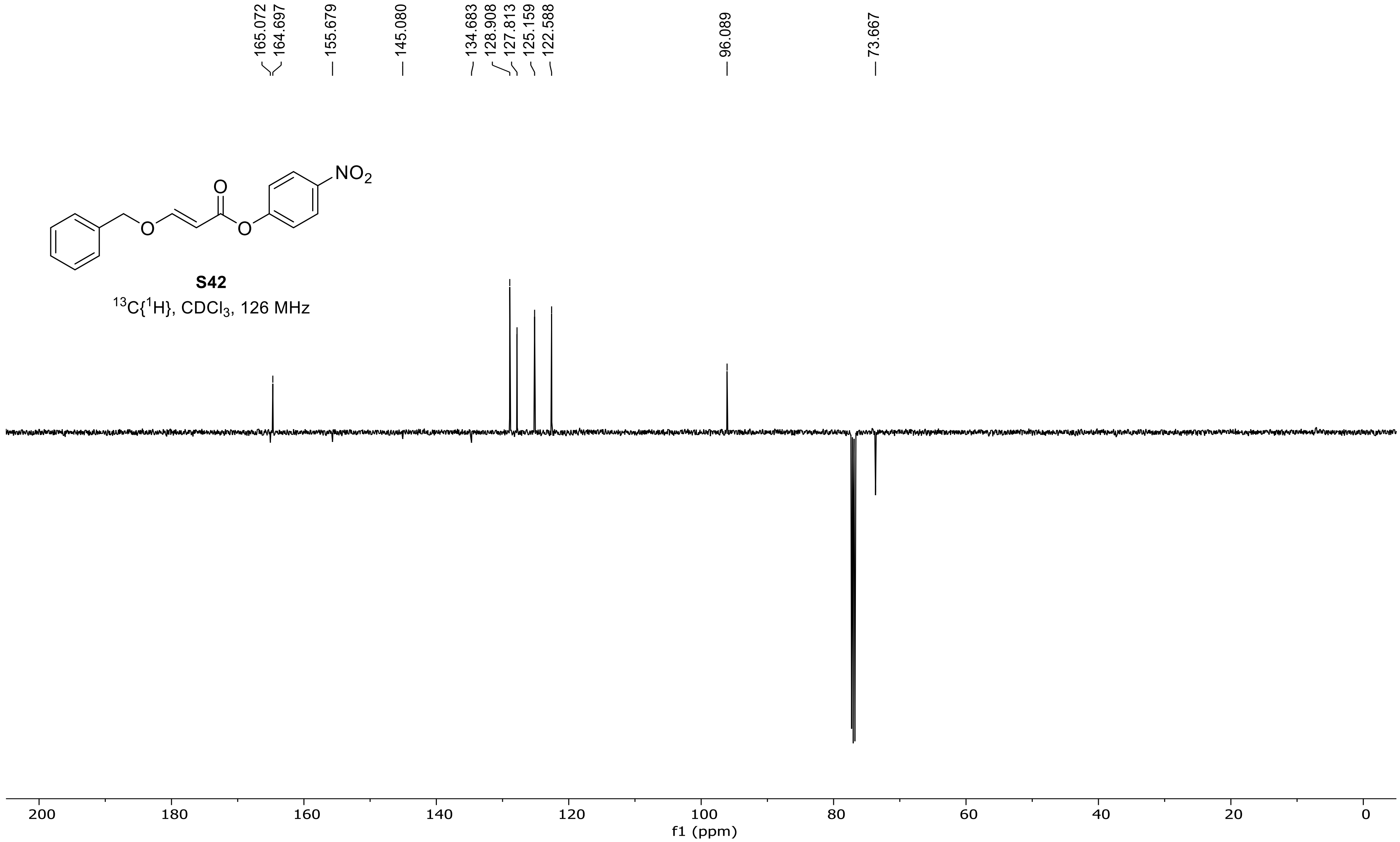
165.072
164.697
155.679
145.080
134.683
128.908
127.813
125.159
122.588
96.089
73.667
NO2
O
O
O
S42
13C{1H}, CDCl3, 126 MHz
200
180
160
140
120
100
80
60
40
20
0
f1 (ppm)

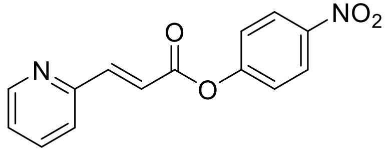

S45

^{1}H, $CDCl_3$, 500 MHz

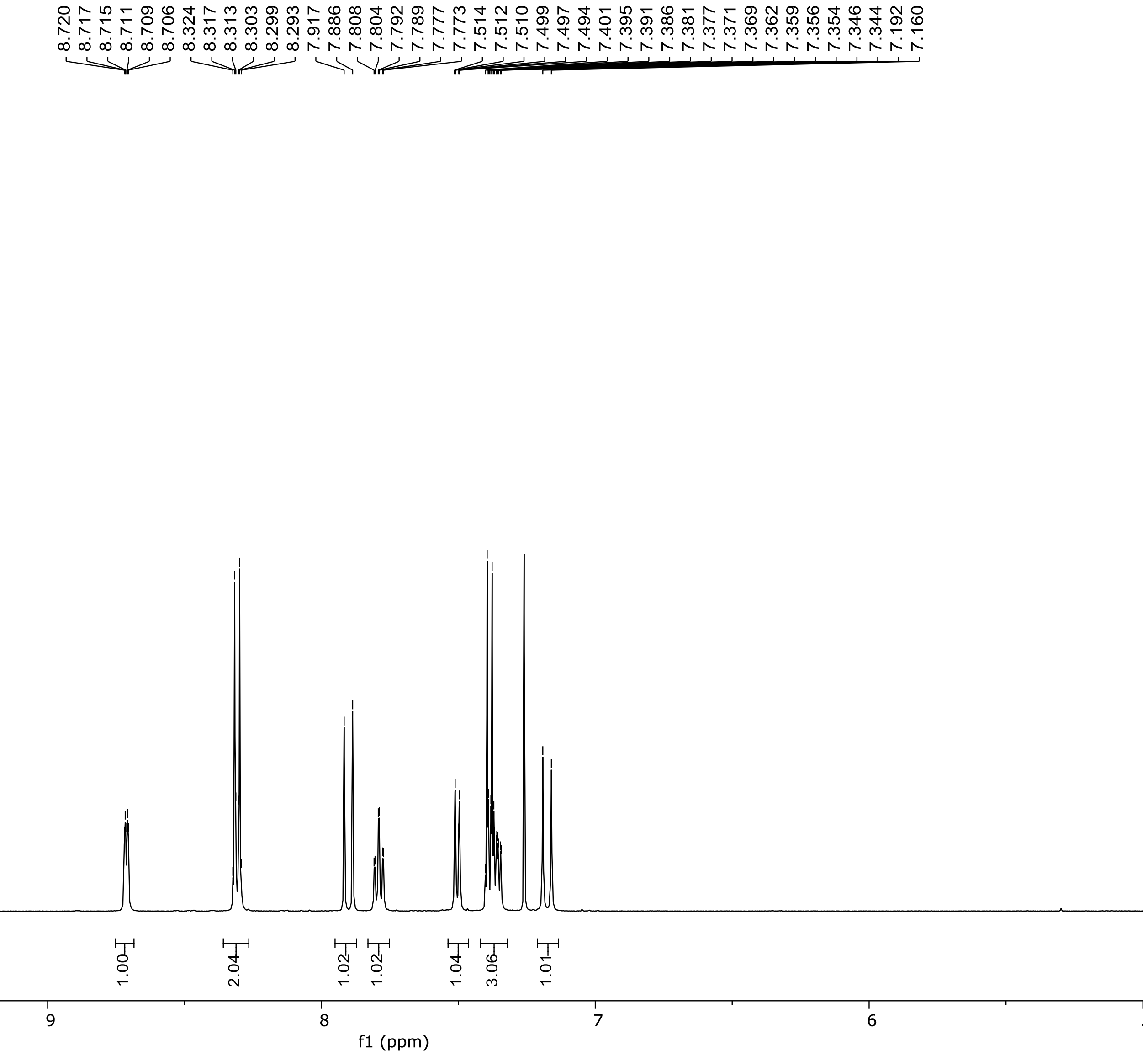

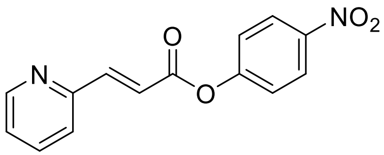

S45

$^{13}C\{^{1}H\}$, $CDCl_3$, 126 MHz

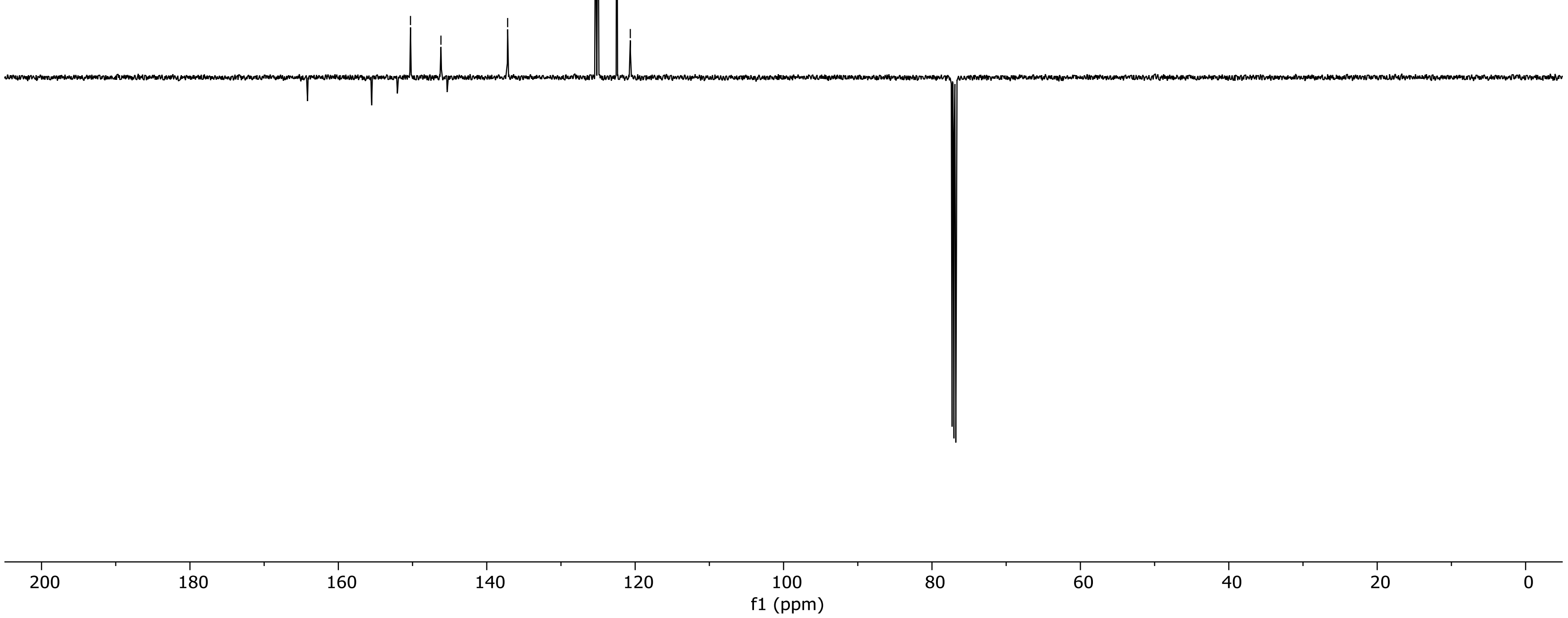

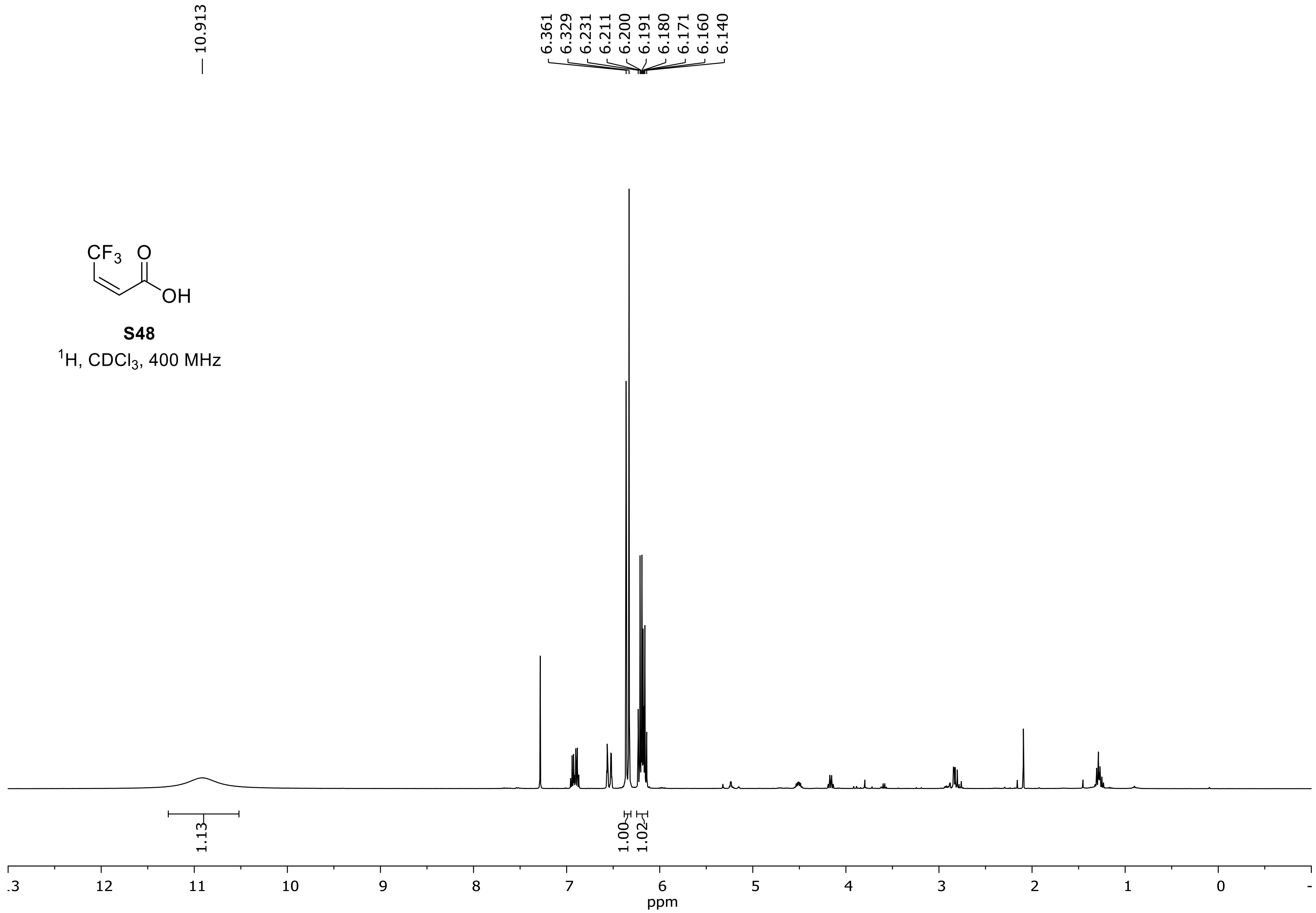
10.913
6.361
6.329
6.231
6.211
6.200
6.191
6.180
6.171
6.160
6.140
CF3
O
OH
S48
1H, CDCl3, 400 MHz
1.13
1.00
1.02
ppm

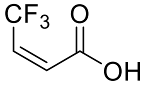

S48

$^{13}C\{^{1}H\}$, $CDCl_3$, 126 MHz

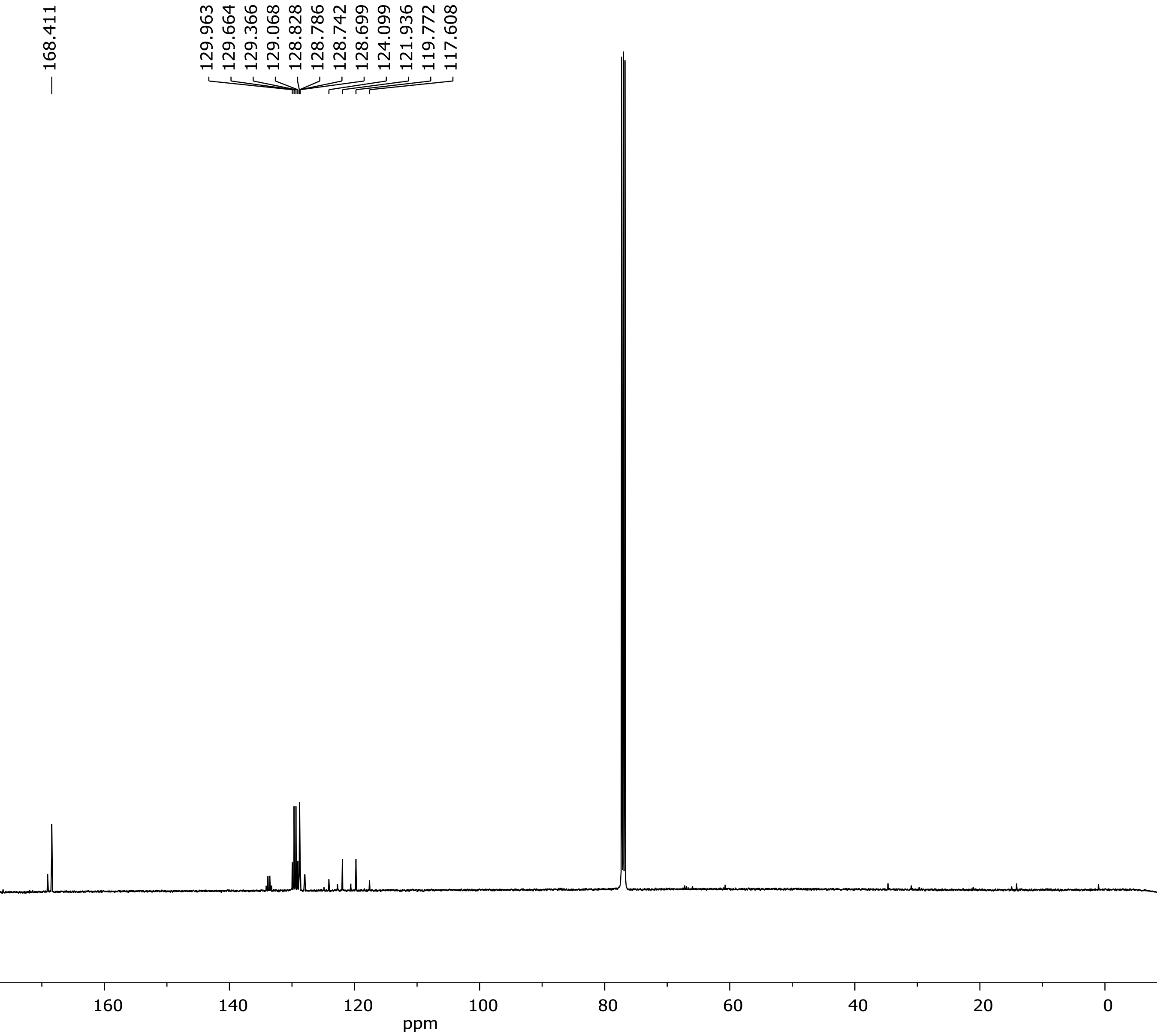

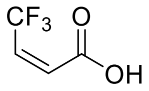

S48

$^{19}F\{^{1}H\}$, $CDCl_3$, 376 MHz

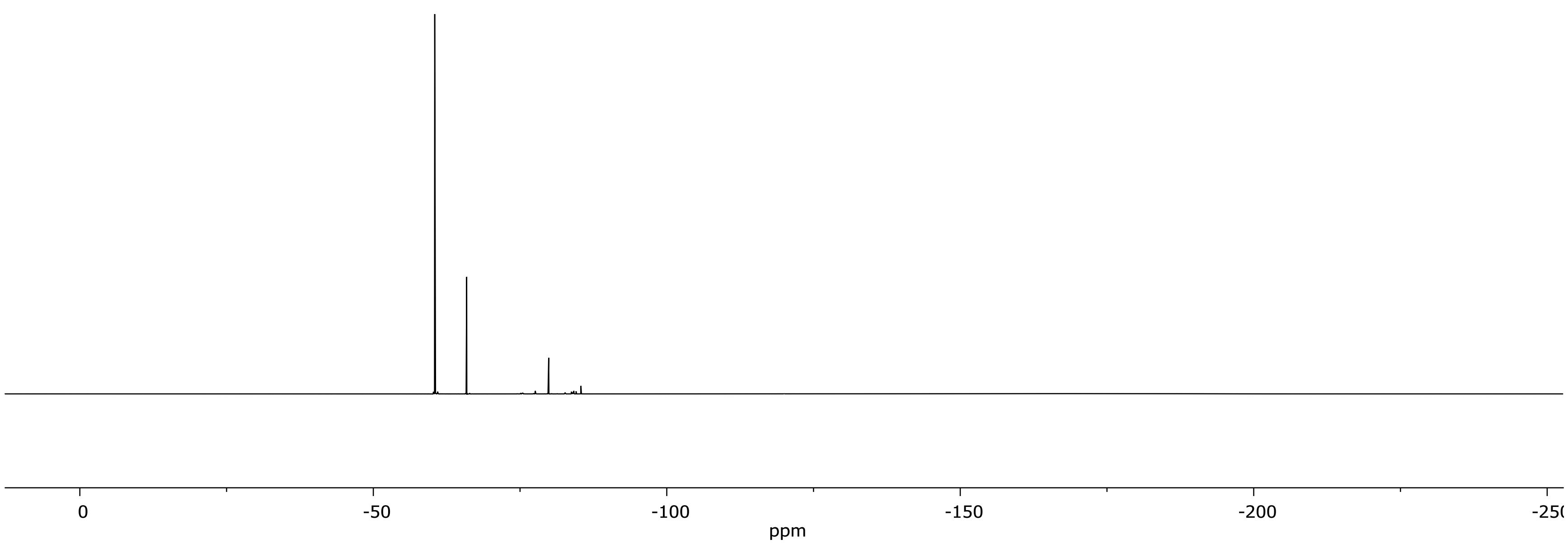

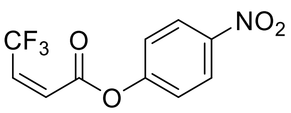

23

^{1}H, $CDCl_3$, 400 MHz

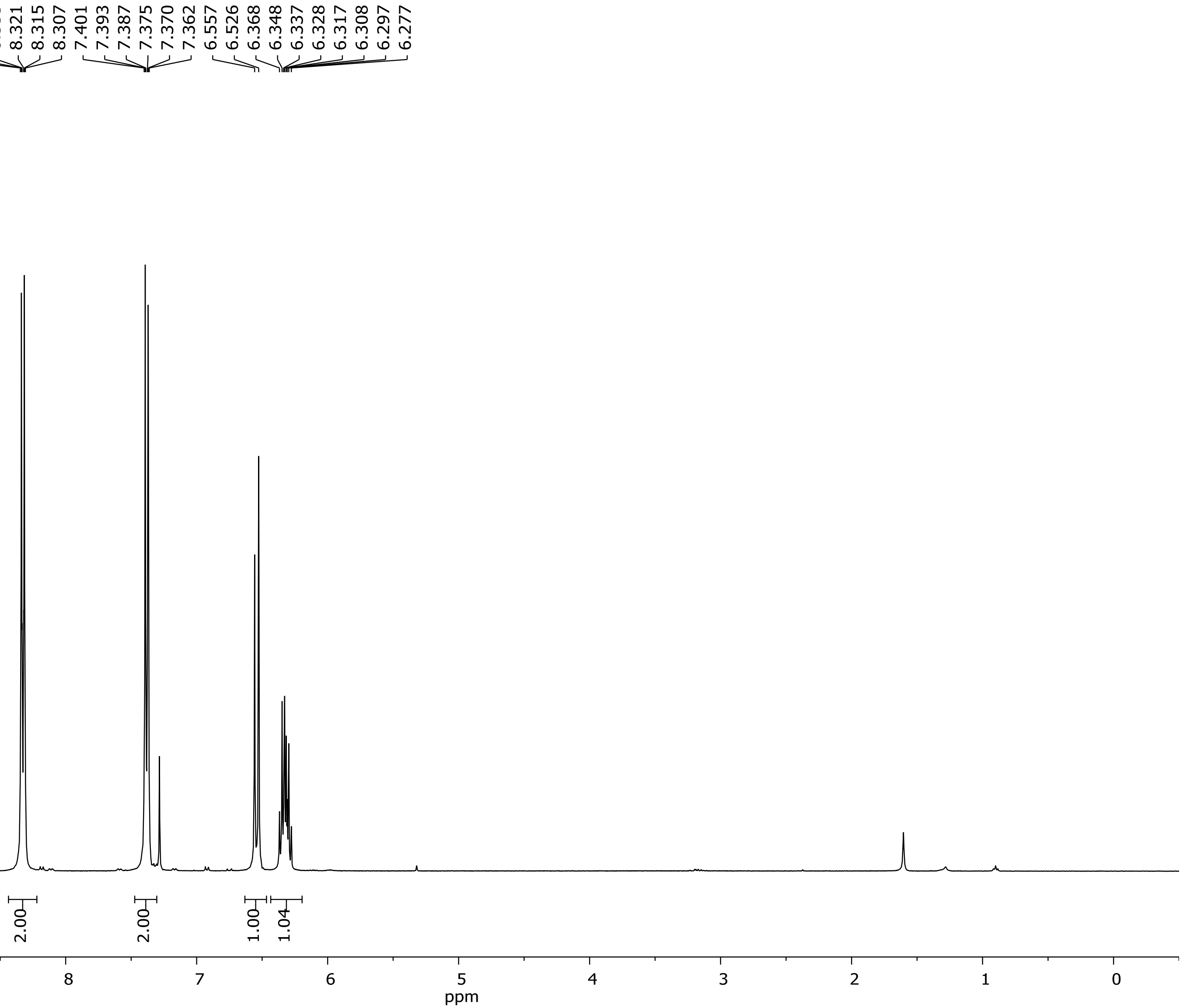

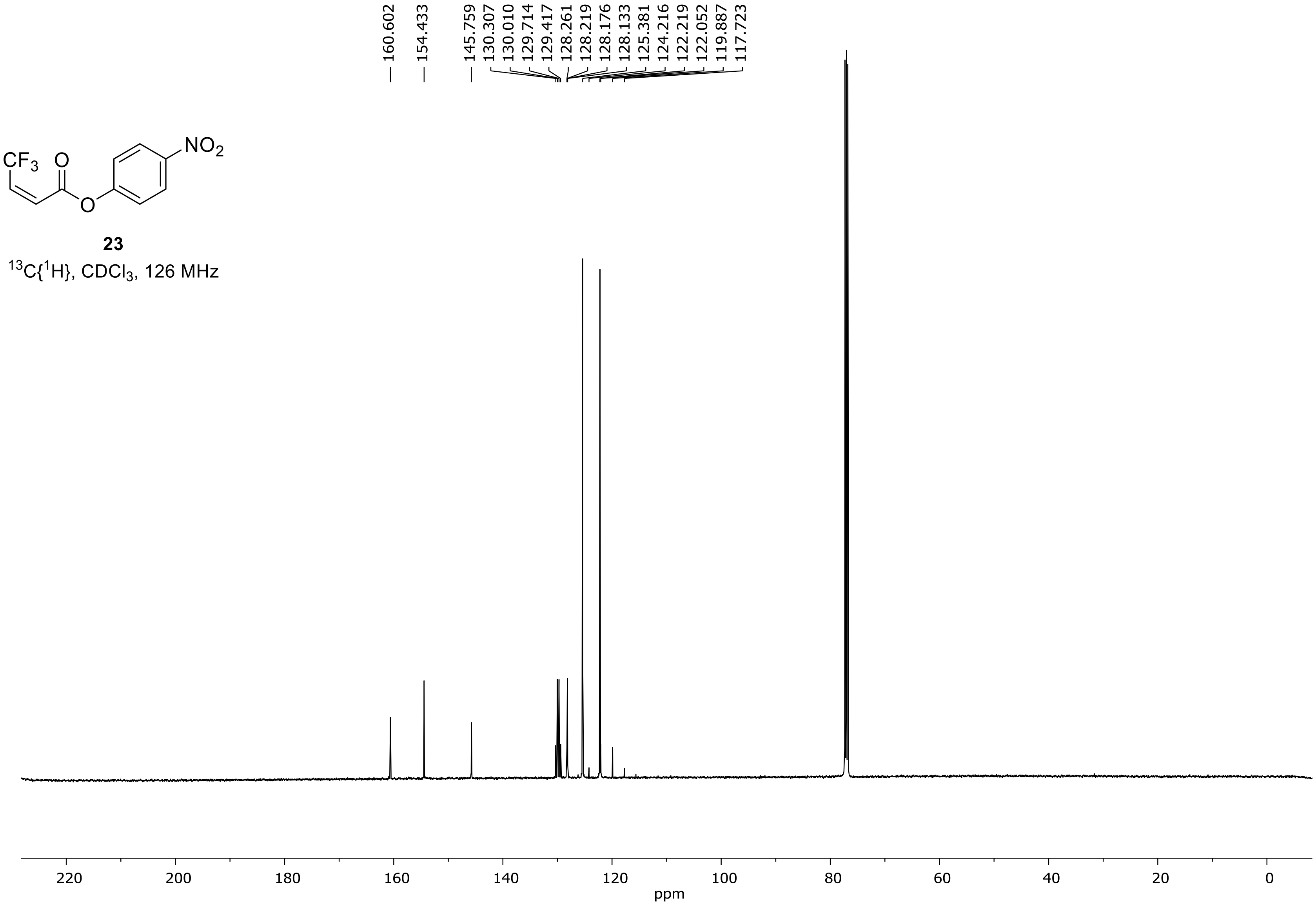

23

$^{13}C\{^{1}H\}$, $CDCl_3$, 126 MHz

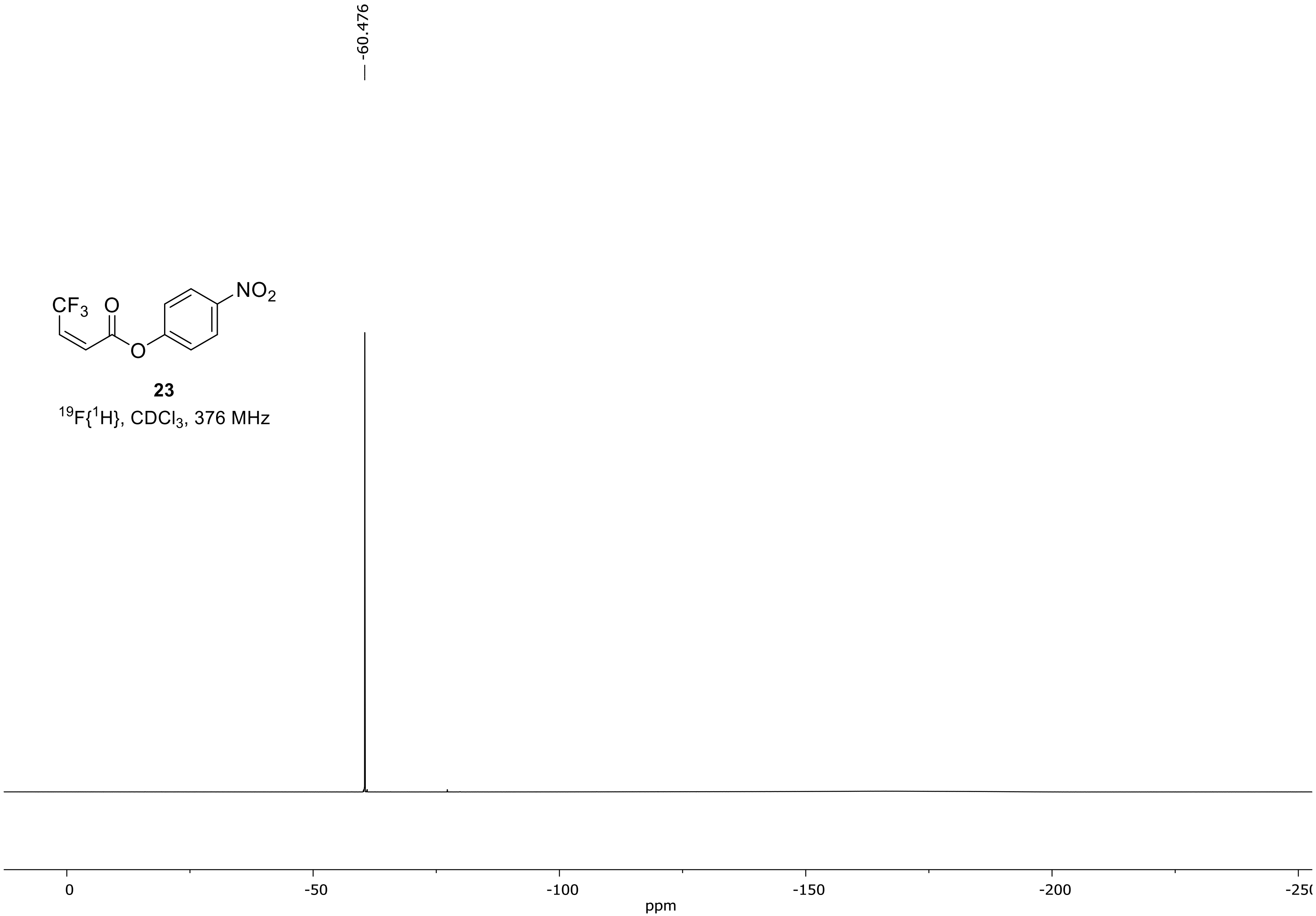

23

$^{19}F\{^{1}H\}$, $CDCl_3$, 376 MHz

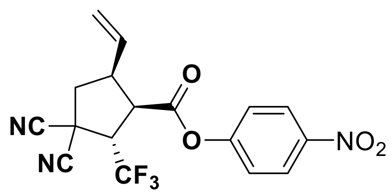

4

^{1}H, $CDCl_3$, 500 MHz

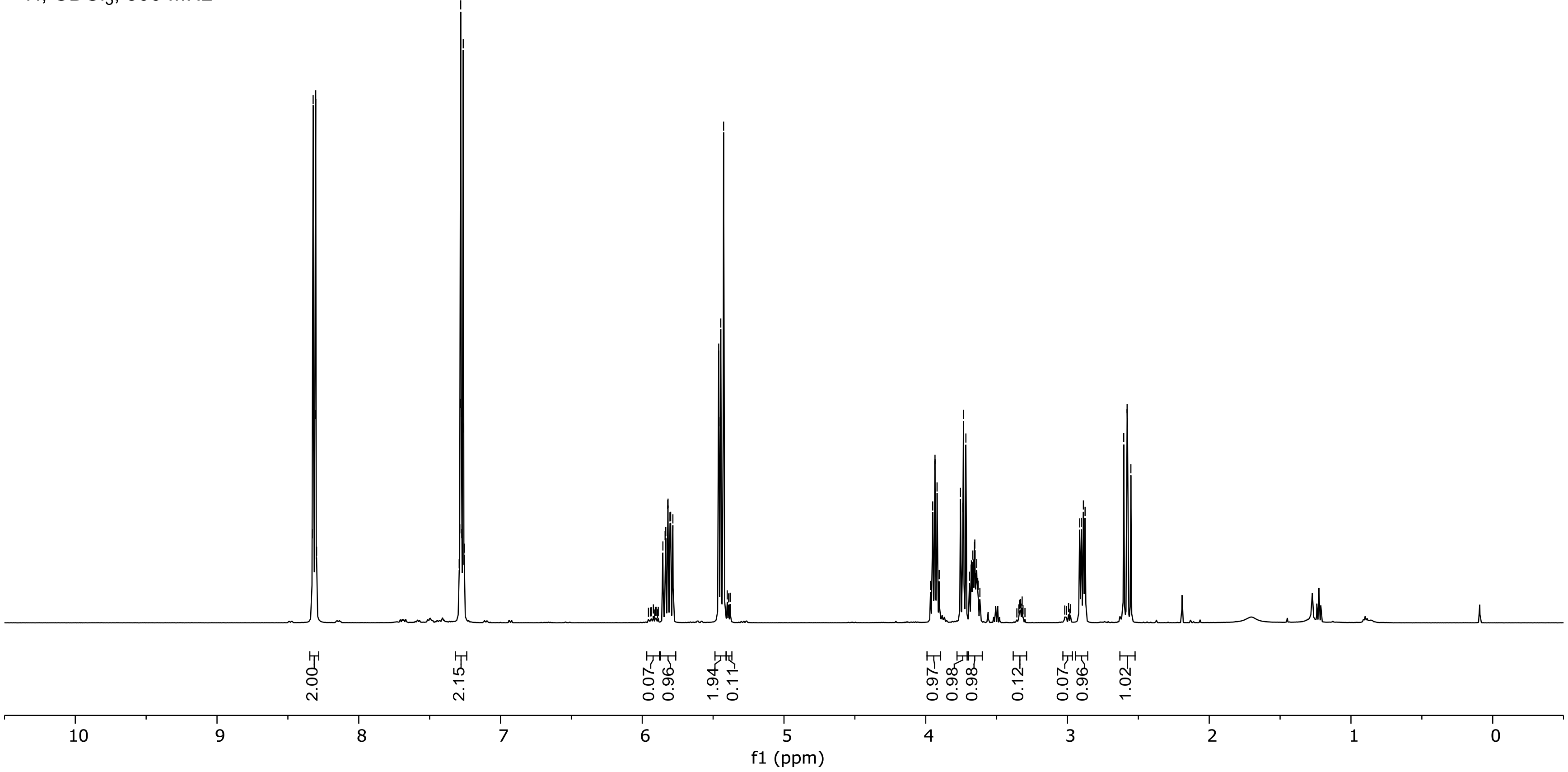

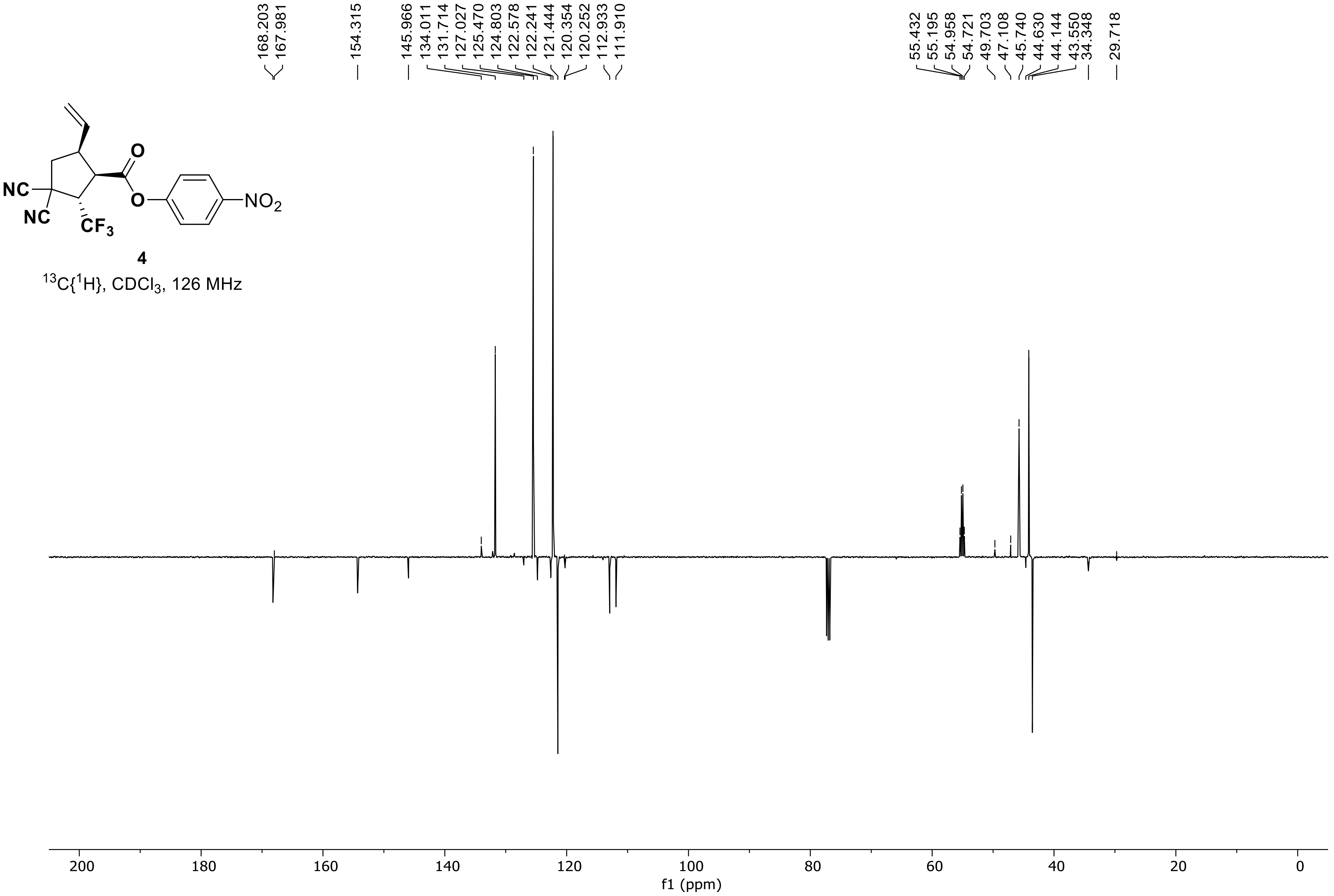
168.203
167.981
154.315
145.966
134.011
131.714
127.027
125.470
124.803
122.578
122.241
121.444
120.354
120.252
112.933
111.910
55.432
55.195
54.958
54.721
49.703
47.108
45.740
44.630
44.144
43.550
34.348
29.718
NC
NC
O
O
CF_3
NO_2
4
$^{13}C\{^1H\}$, $CDCl_3$, 126 MHz
200
180
160
140
120
100
80
60
40
20
0
f1 (ppm)

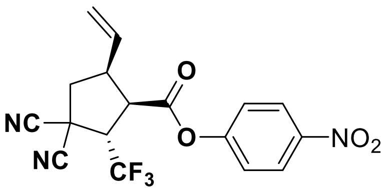

4

$^{19}F\{^{1}H\}$, $CDCl_3$, 376 MHz

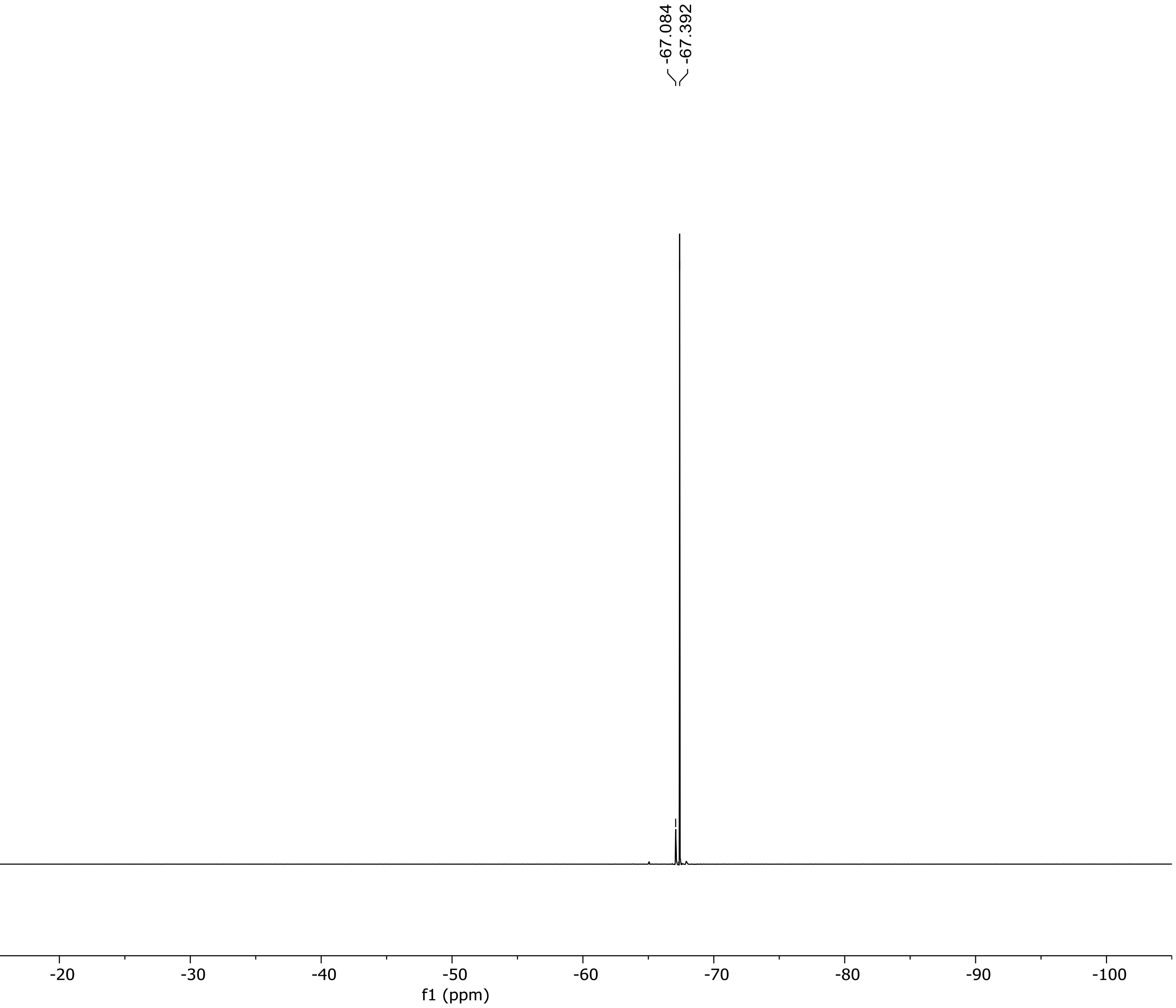

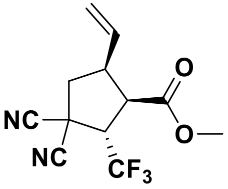

8

^{1}H, $CDCl_3$, 500 MHz

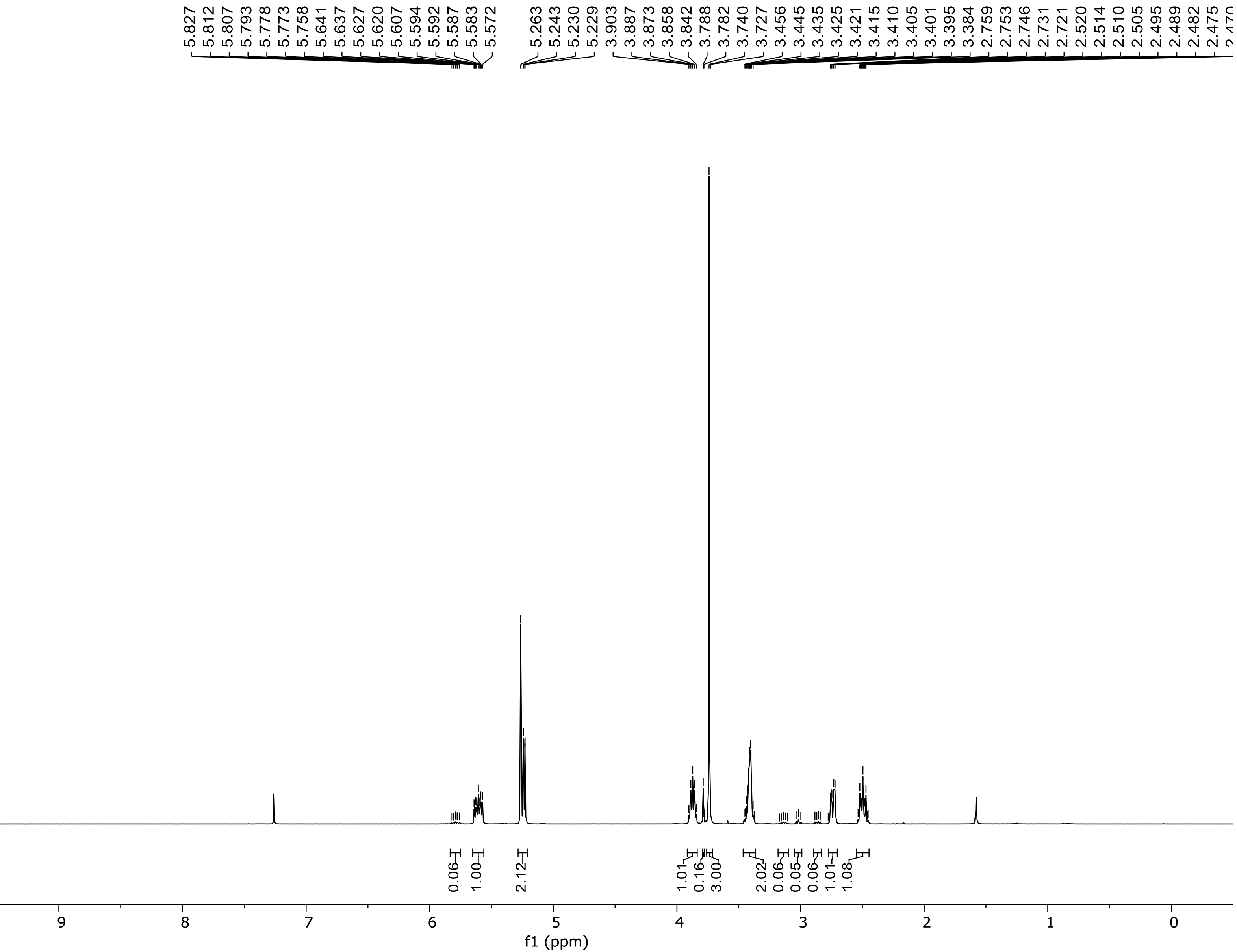

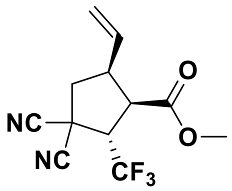

8

$^{13}C\{^1H\}$, $CDCl_3$, 126 MHz

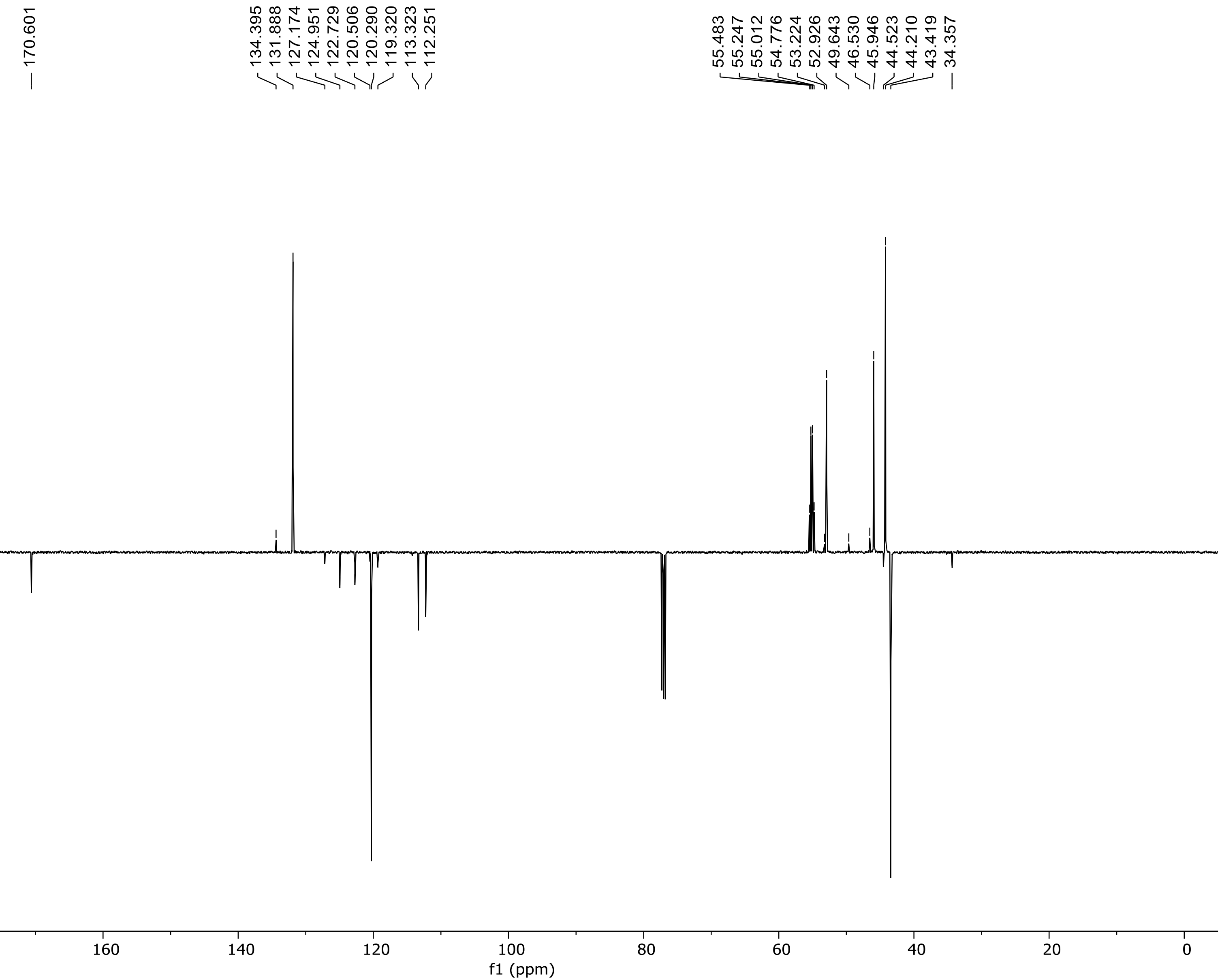

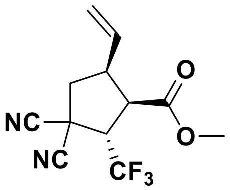

8

$^{19}F\{^{1}H\}$, $CDCl_3$, 376 MHz

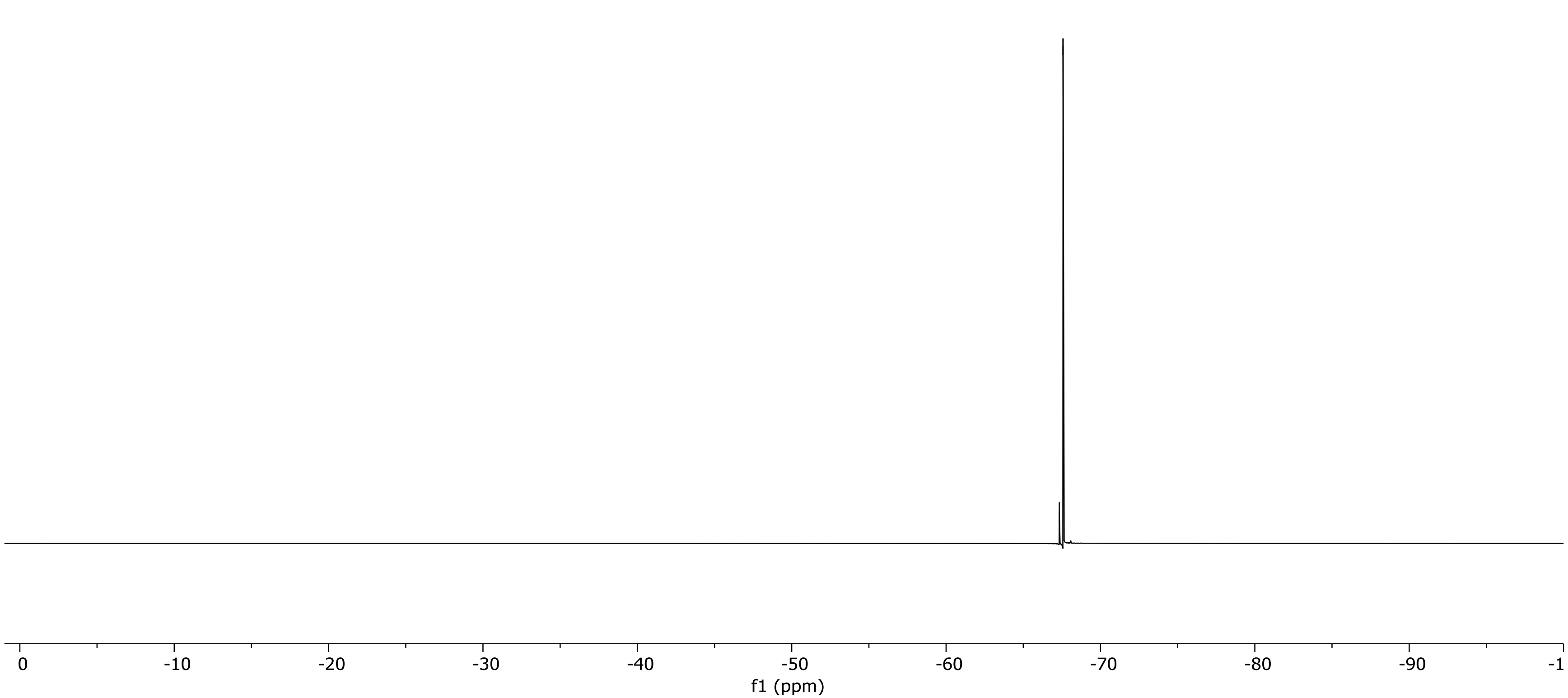

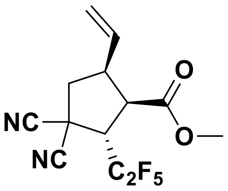

9

^{1}H, $CDCl_3$, 500 MHz

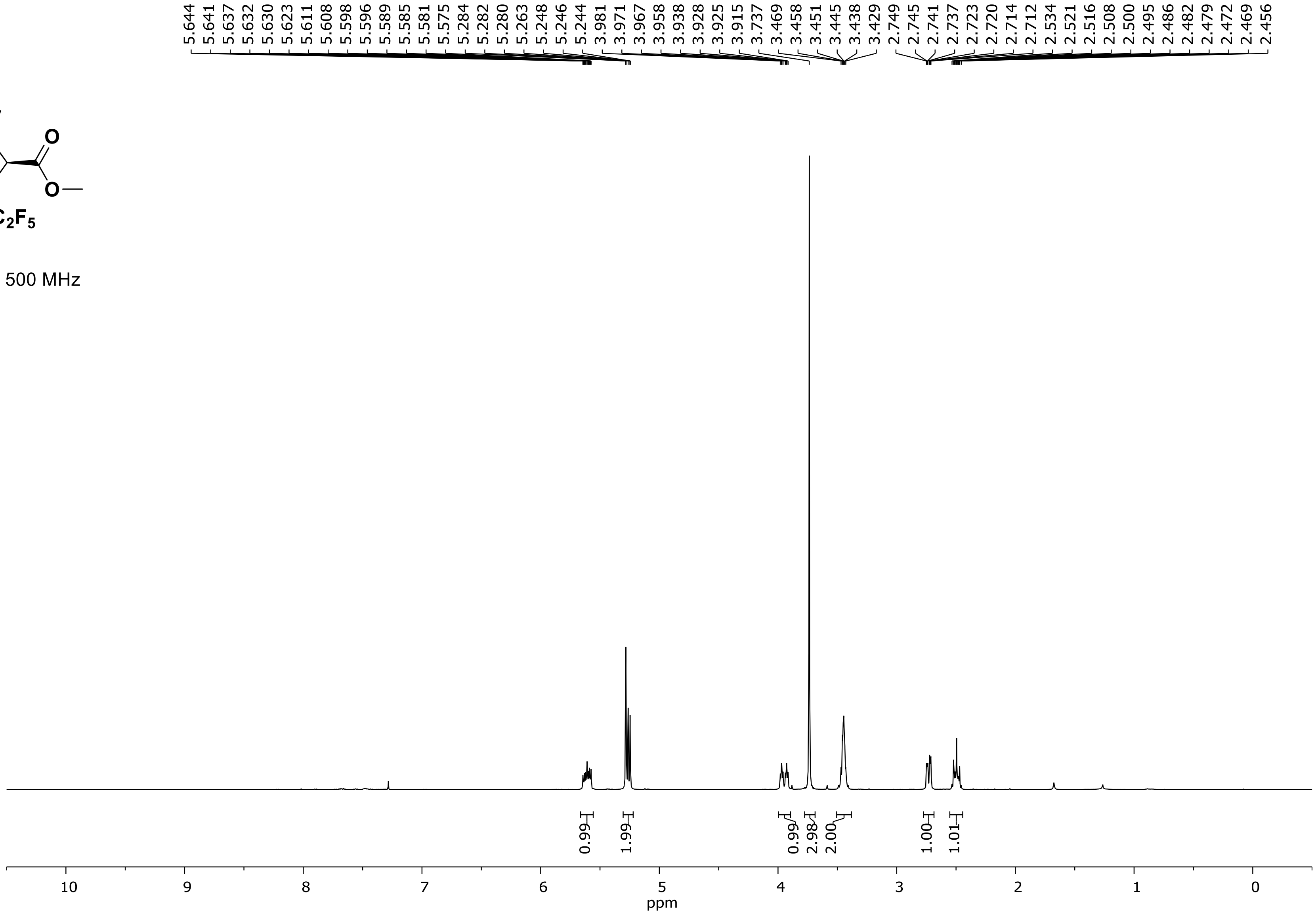

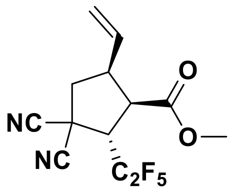

9

$^{13}C\{^{1}H\}$, $CDCl_3$, 126 MHz

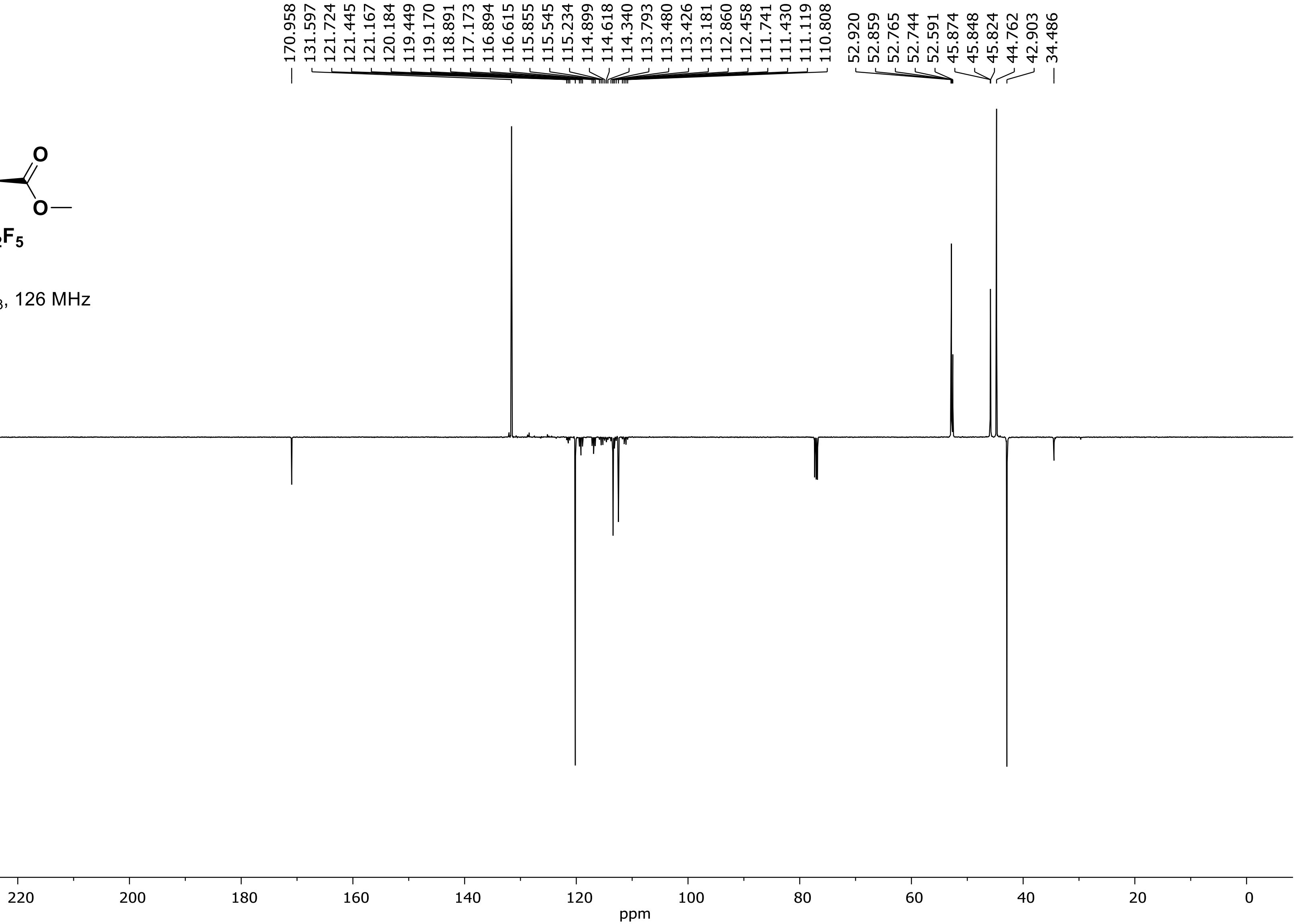

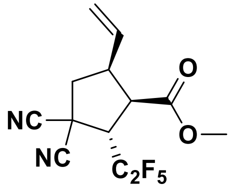

9

$^{19}F\{^1H\}$, $CDCl_3$, 376 MHz

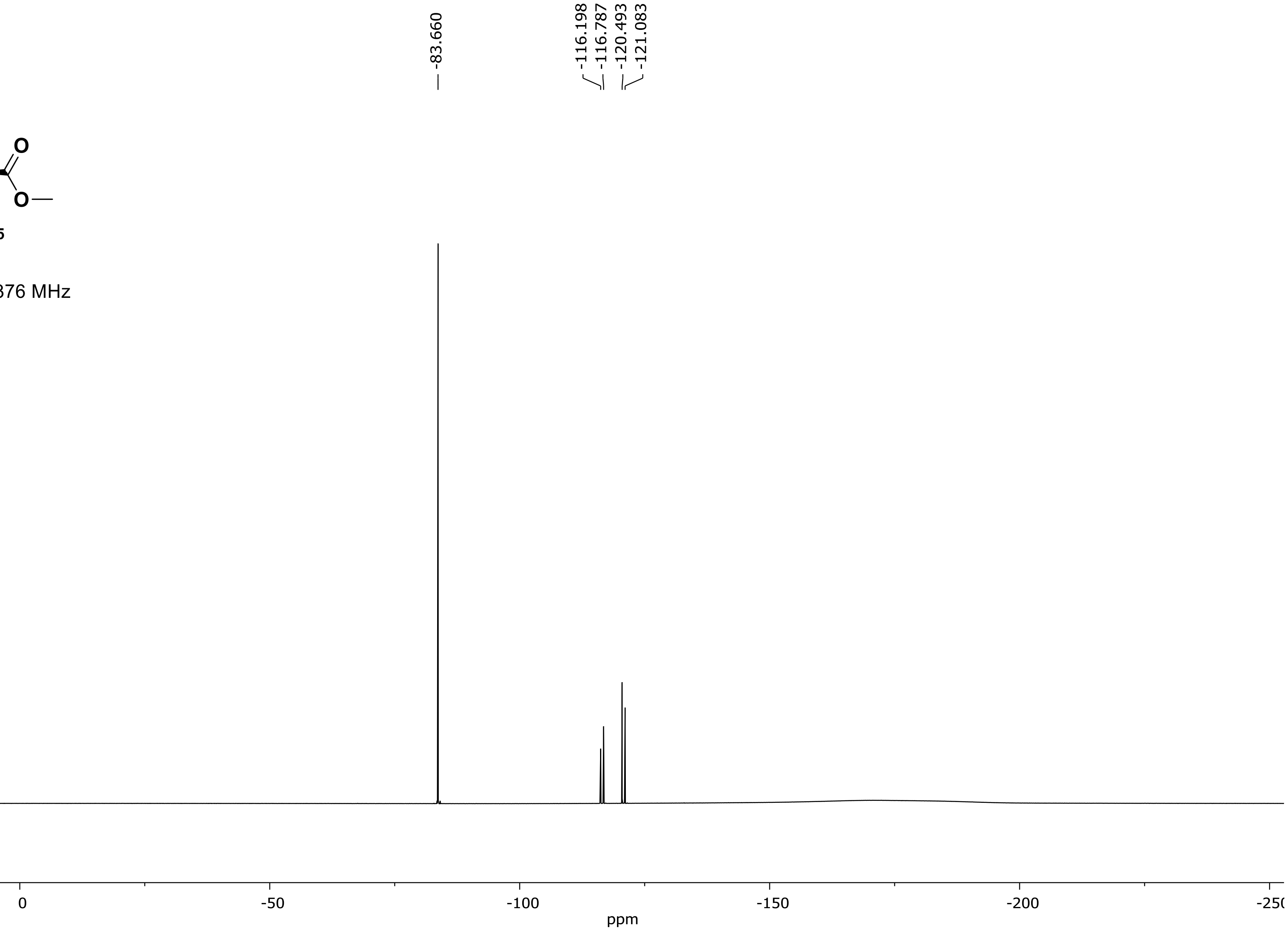

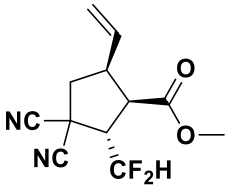

10

^{1}H, $CDCl_3$, 500 MHz

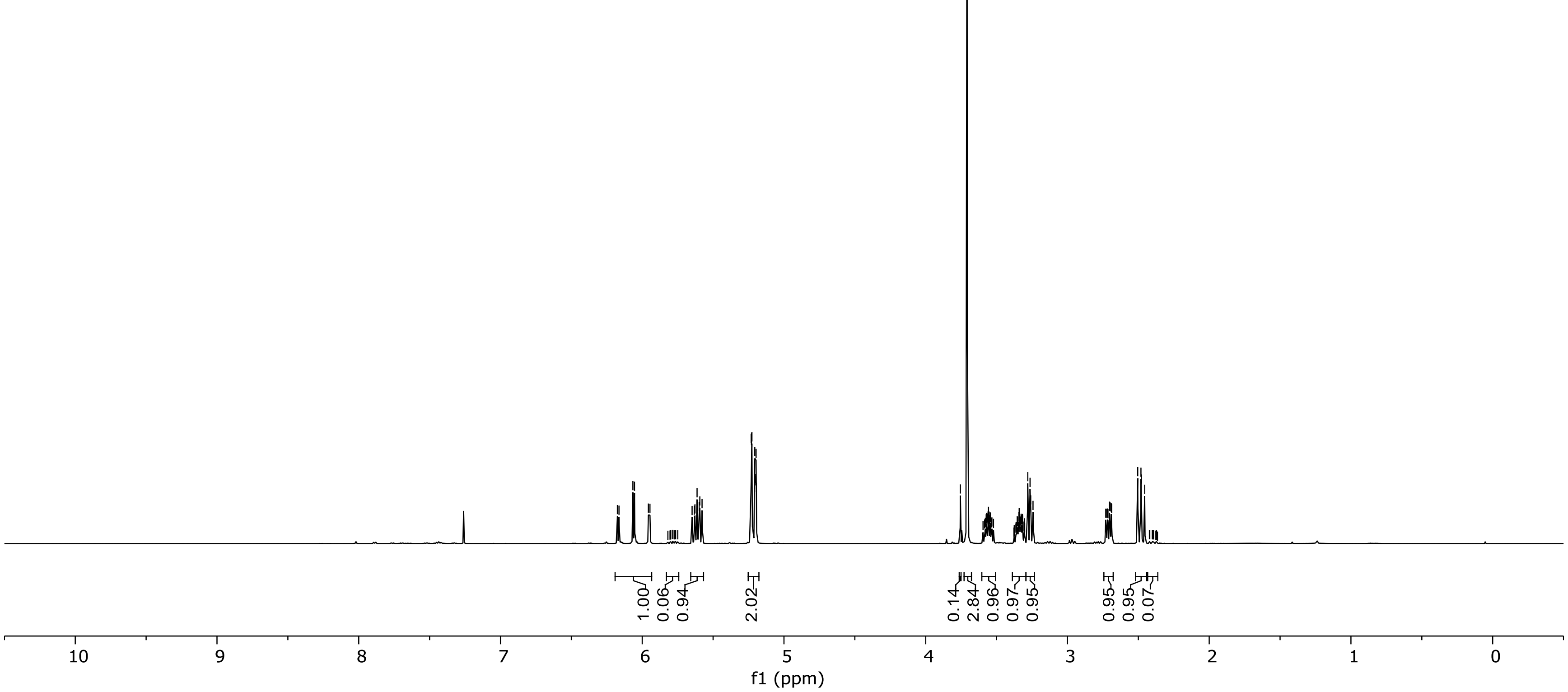

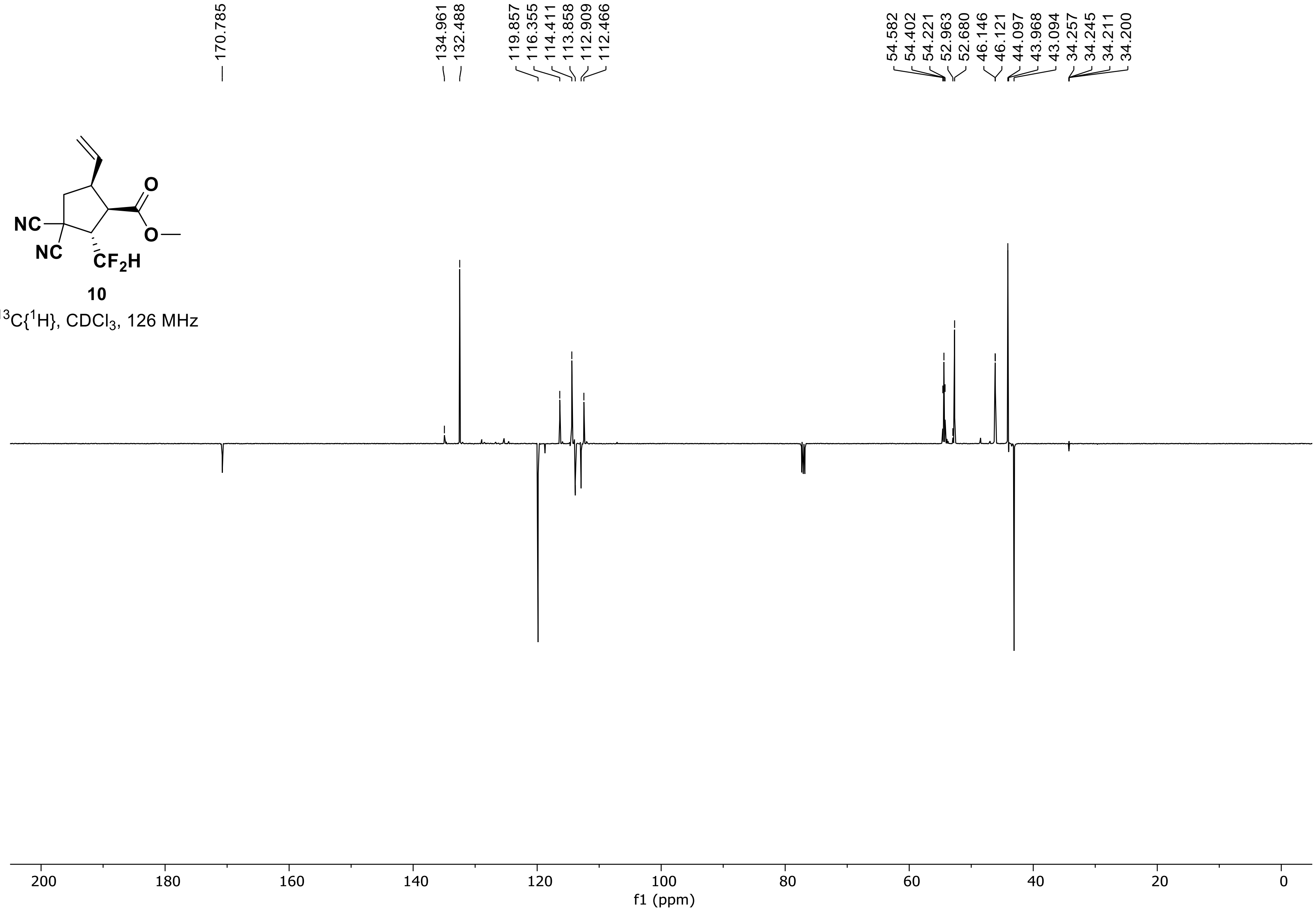
170.785
134.961
132.488
119.857
116.355
114.411
113.858
112.909
112.466
54.582
54.402
54.221
52.963
52.680
46.146
46.121
44.097
43.968
43.094
34.257
34.245
34.211
34.200
NC
NC
O
O
CF2H
10
13C{1H}, CDCl3, 126 MHz
200
180
160
140
120
100
80
60
40
20
0
f1 (ppm)

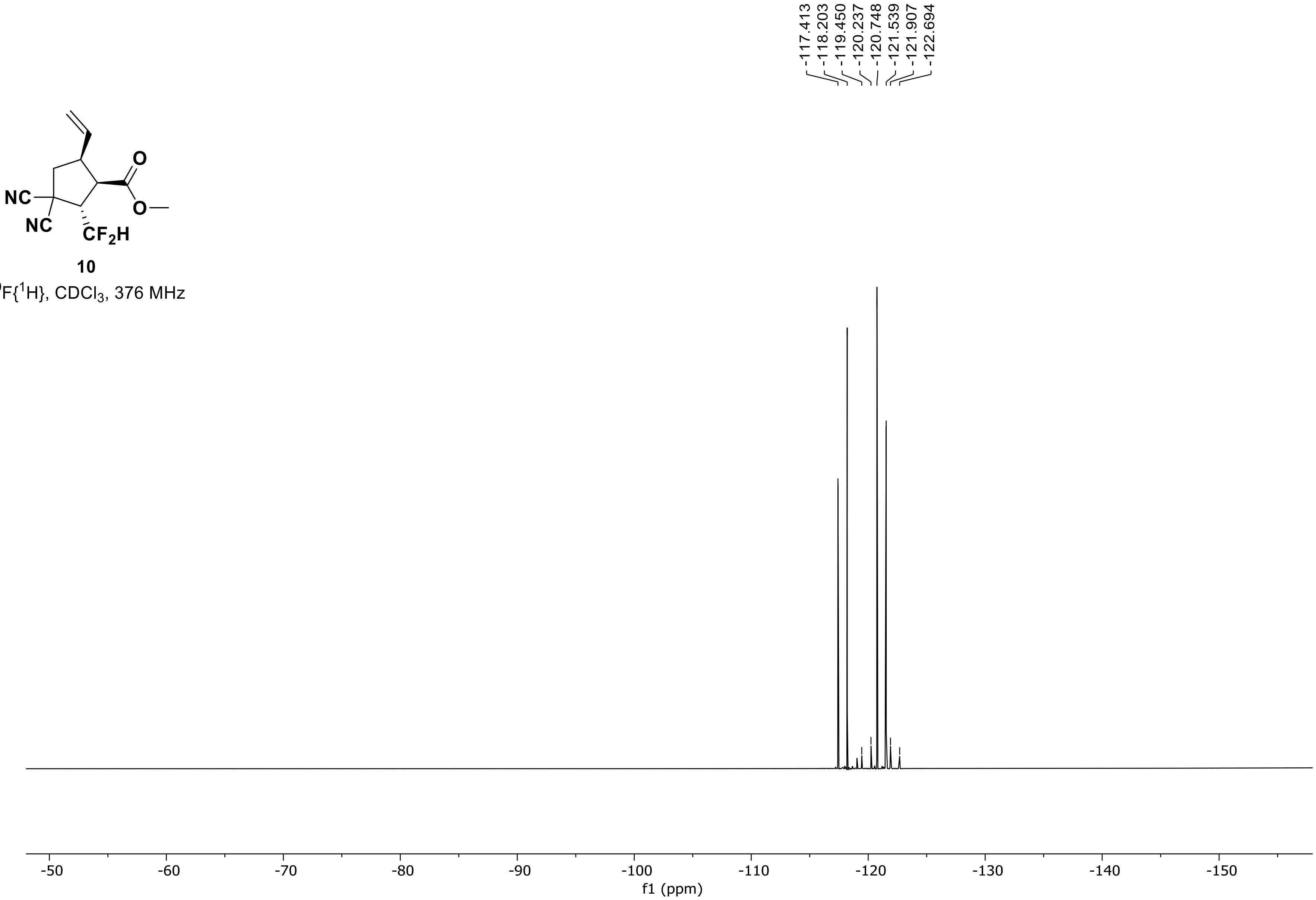

10

$^{19}F\{^{1}H\}$, $CDCl_3$, 376 MHz

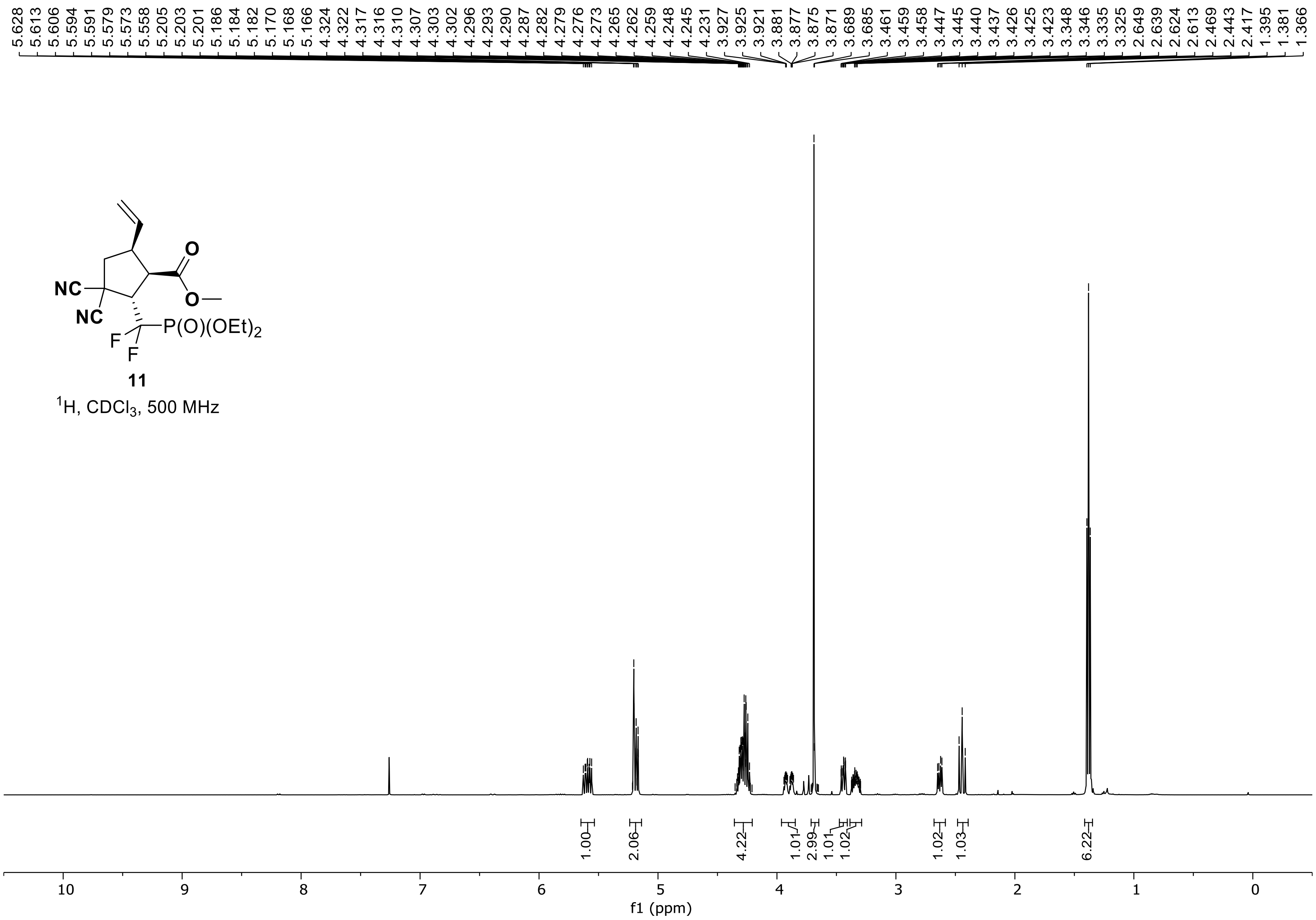

11

^1H, $CDCl_3$, 500 MHz

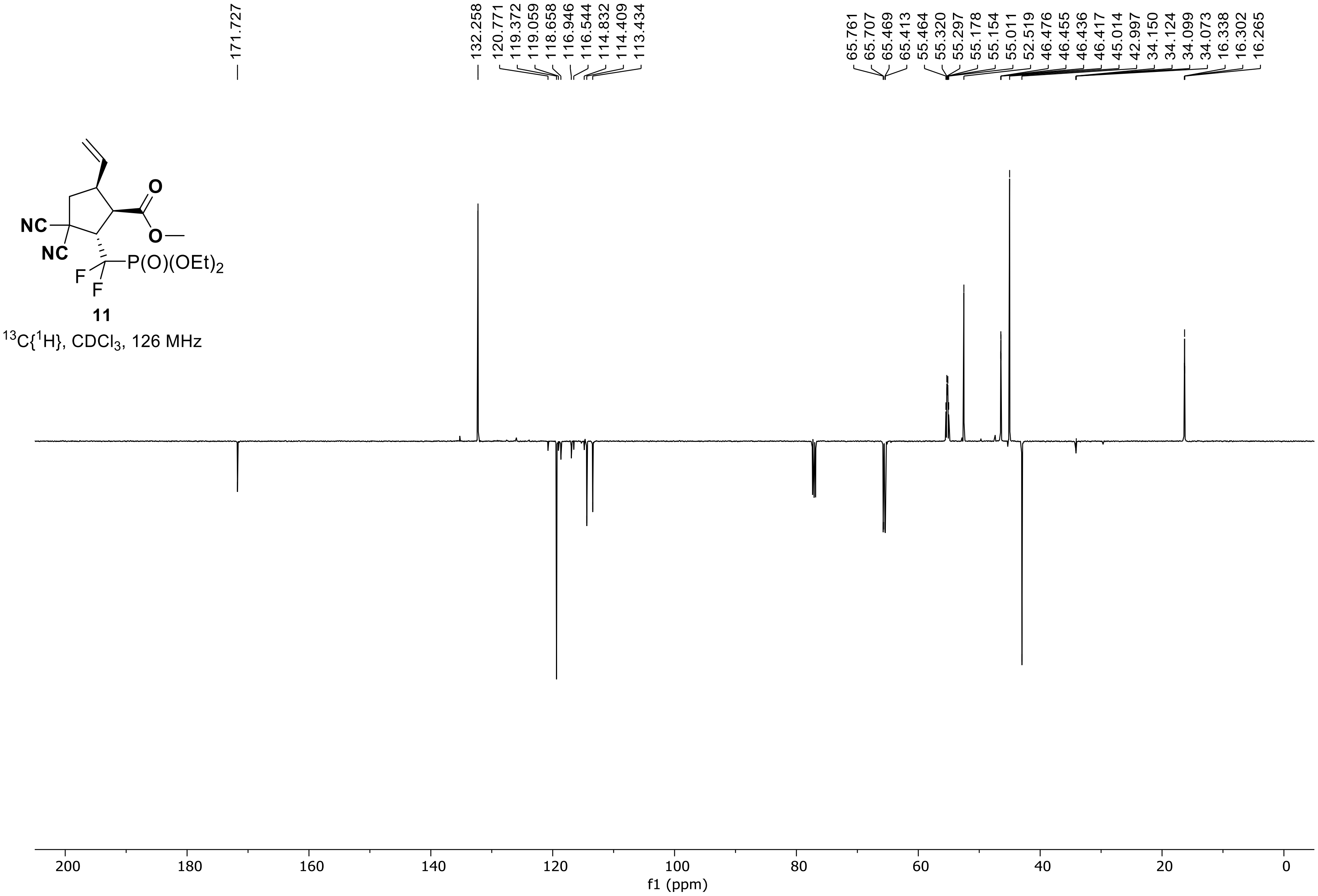

11

$^{13}C\{^1H\}$, $CDCl_3$, 126 MHz

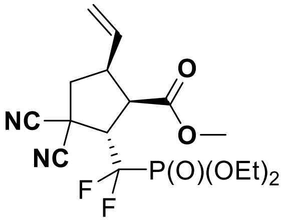

11

$^{19}F\{^{1}H\}$, $CDCl_3$, 376 MHz

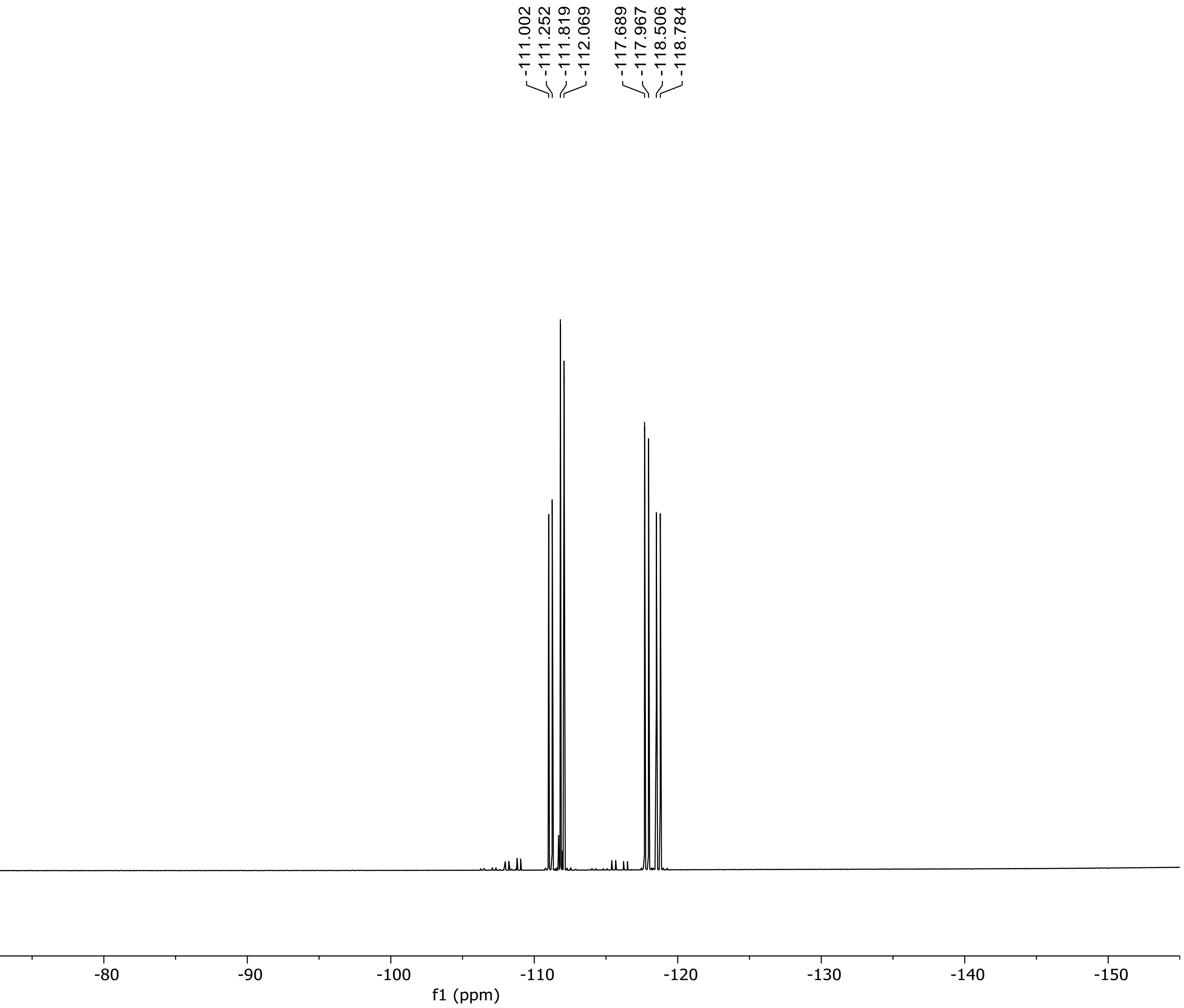

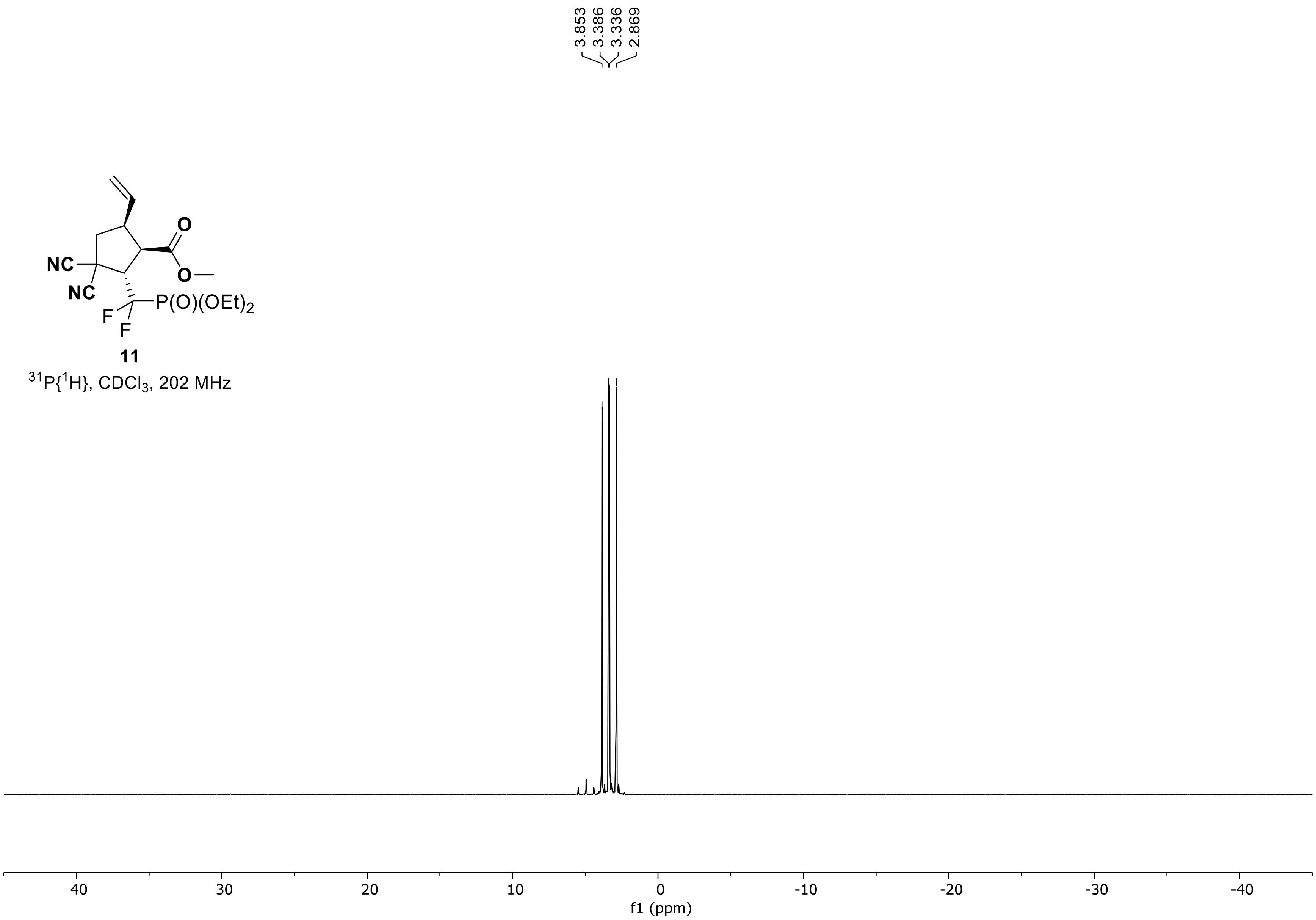
3.853
3.386
3.336
2.869
O
NC
O
NC
P(O)(OEt)$_2$
F
F
11
$^{31}P\{^{1}H\}$, $CDCl_3$, 202 MHz
40
30
20
10
0
-10
-20
-30
-40
f1 (ppm)

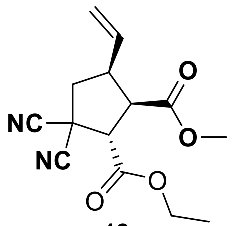

12

^{1}H, $CDCl_3$, 500 MHz

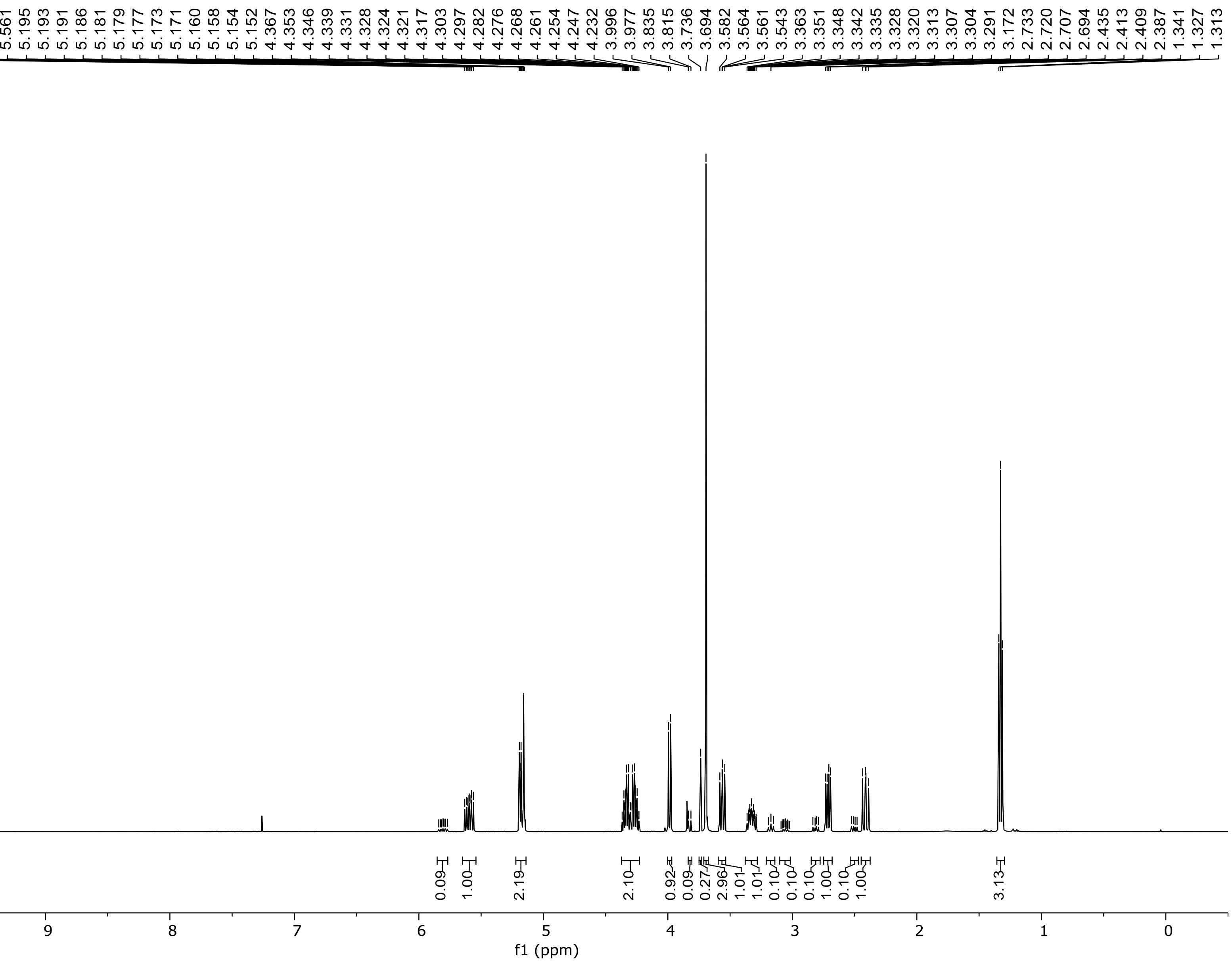

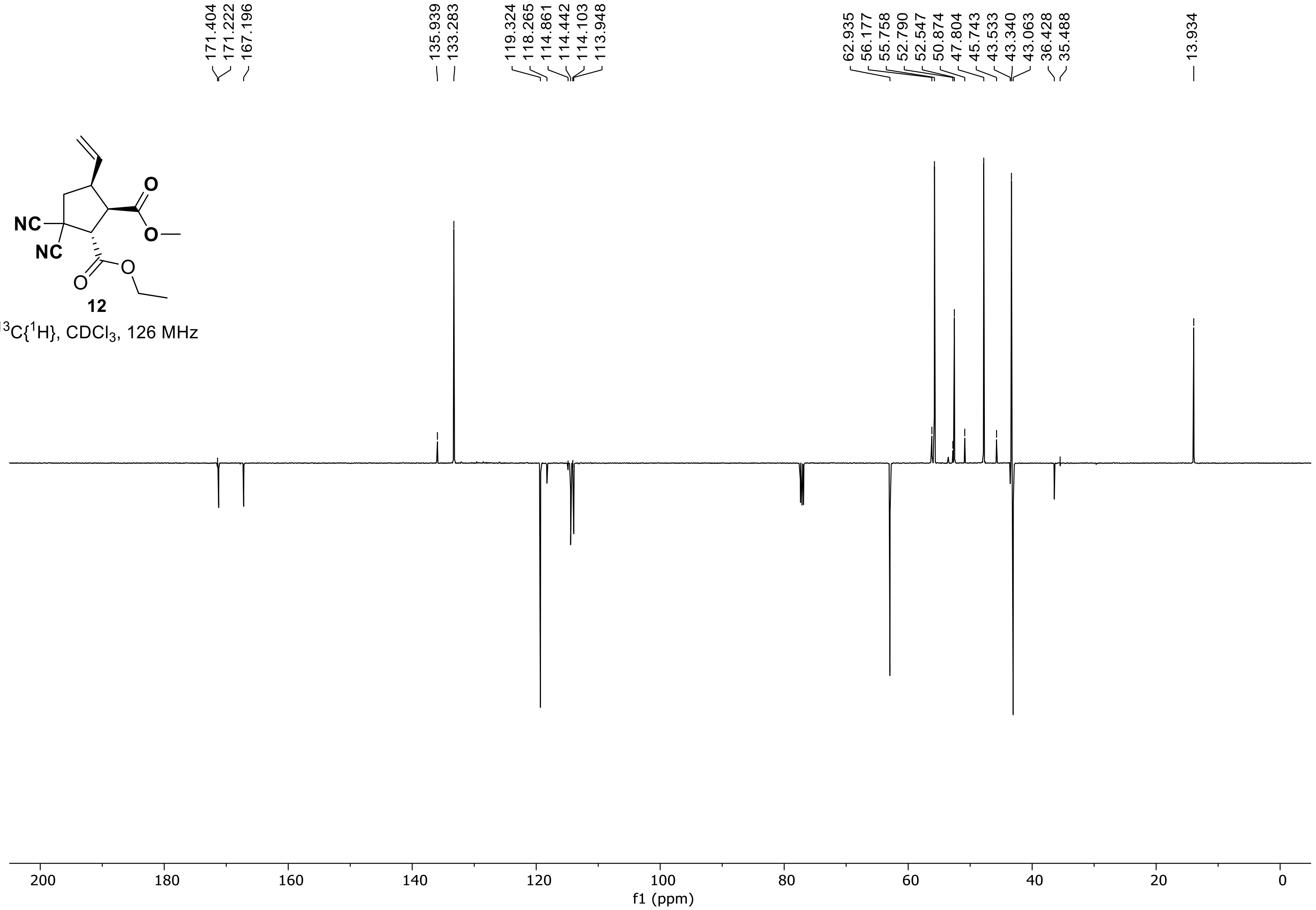

12

$^{13}C\{^1H\}$, $CDCl_3$, 126 MHz

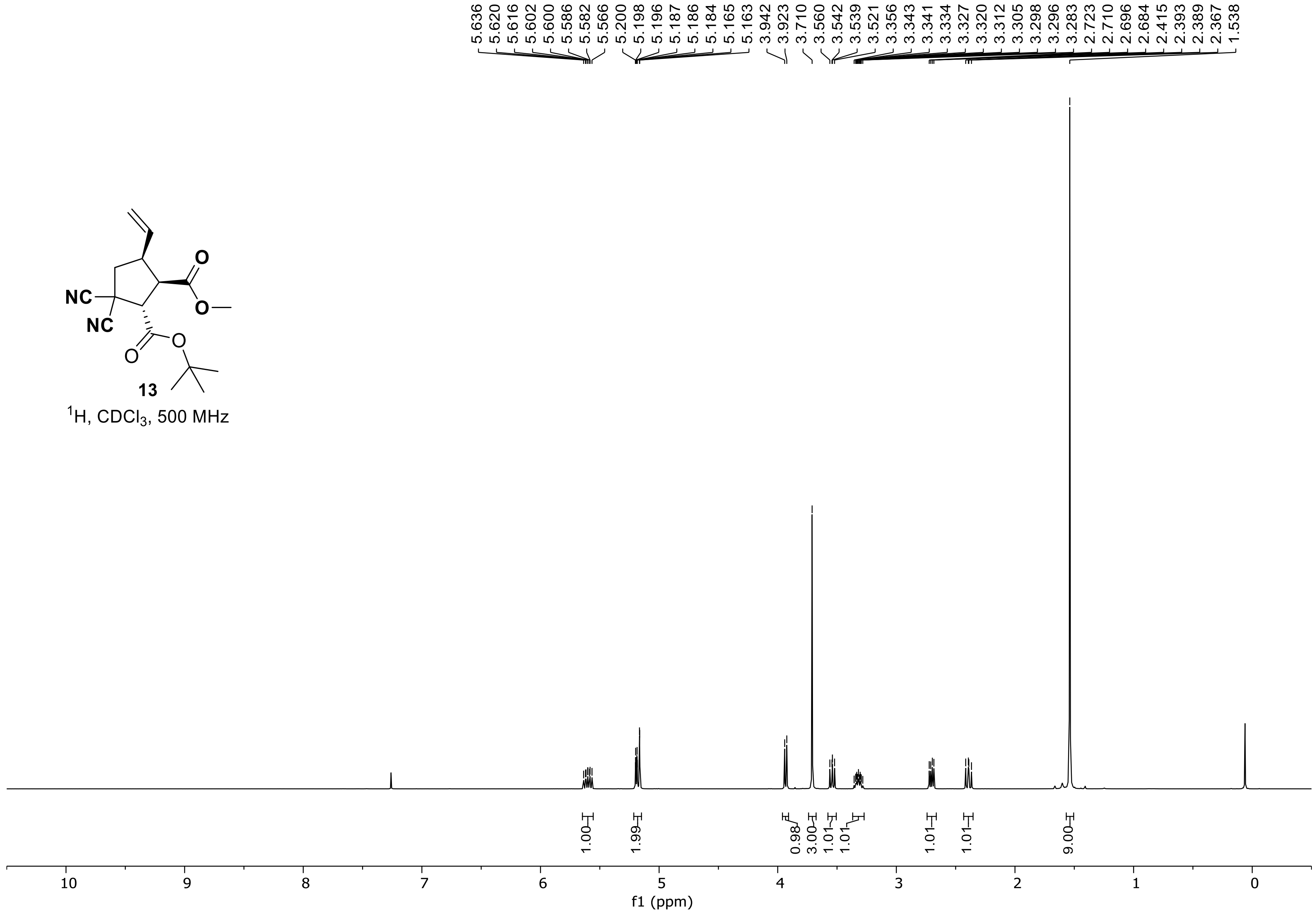

13

^{1}H, $CDCl_3$, 500 MHz

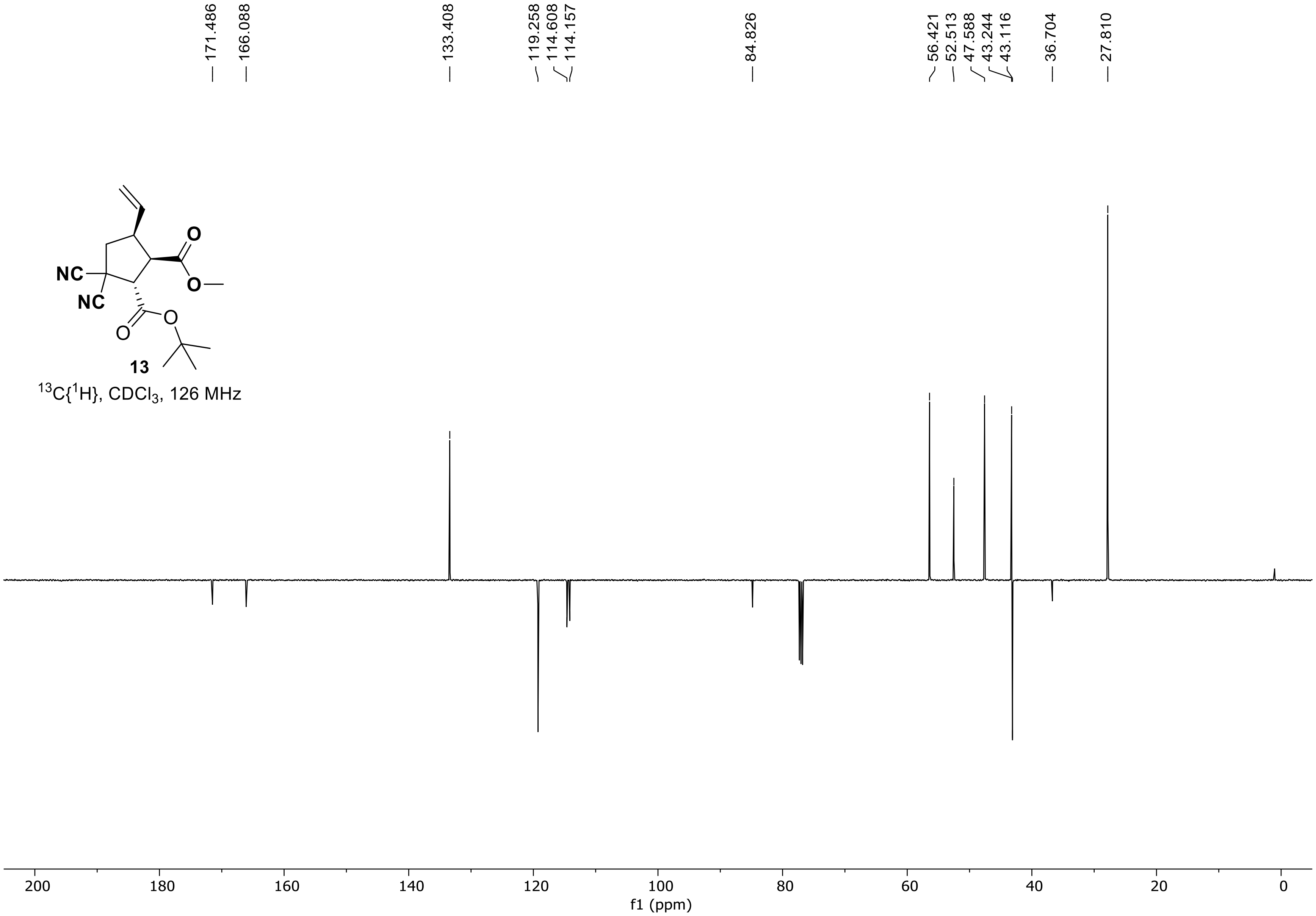
171.486
166.088
133.408
119.258
114.608
114.157
84.826
56.421
52.513
47.588
43.244
43.116
36.704
27.810
NC
NC
O
O
O
O
13
13C{1H}, CDCl3, 126 MHz
200
180
160
140
120
100
80
60
40
20
0
f1 (ppm)

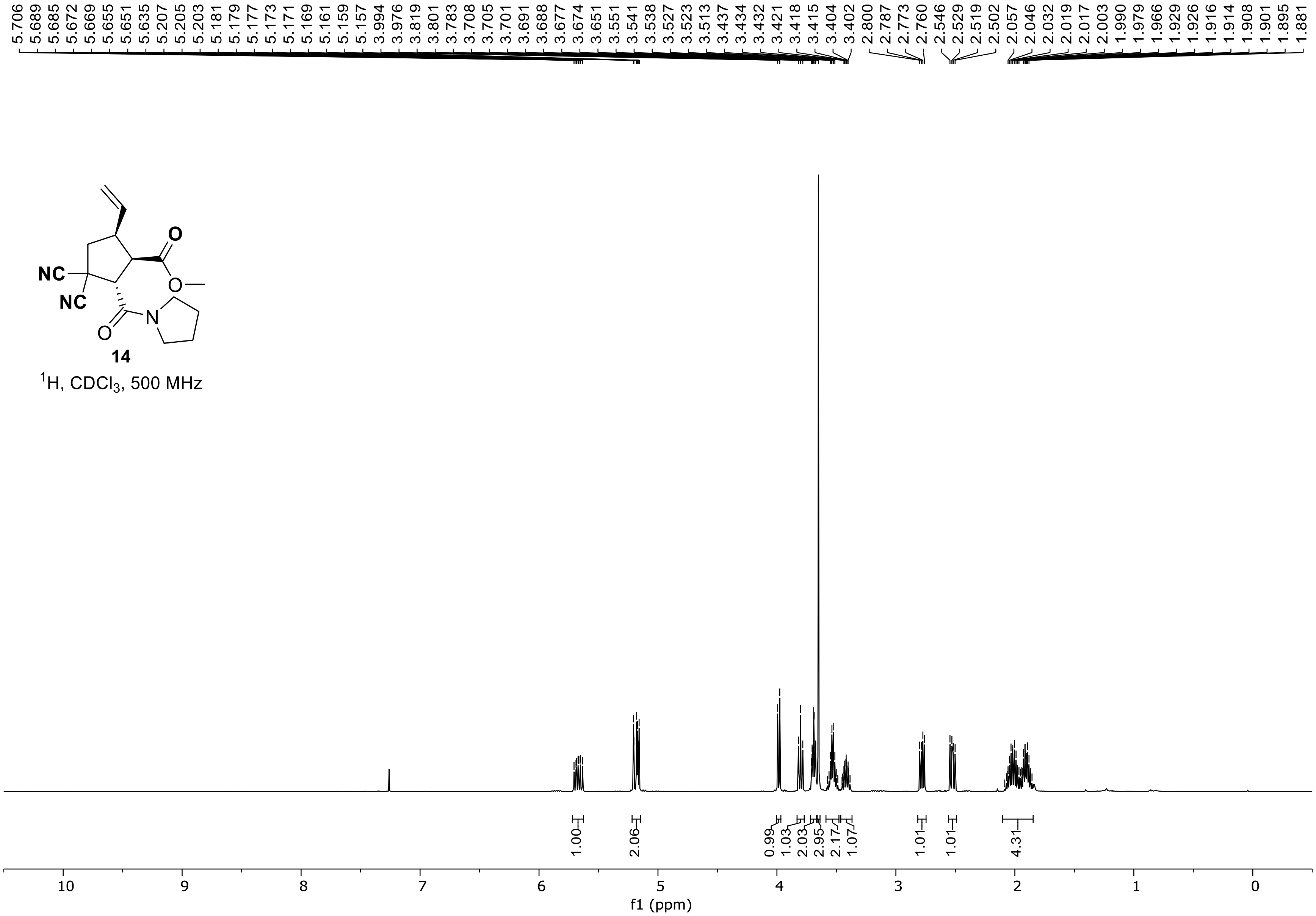
5.706 5.689 5.685 5.672 5.669 5.655 5.651 5.635 5.207 5.205 5.203 5.181 5.179 5.177 5.173 5.171 5.169 5.161 5.159 5.157 3.994 3.976 3.819 3.801 3.783 3.708 3.705 3.701 3.691 3.688 3.677 3.674 3.651 3.551 3.541 3.538 3.527 3.523 3.513 3.437 3.434 3.432 3.421 3.418 3.415 3.404 3.402 2.800 2.787 2.773 2.760 2.546 2.529 2.519 2.502 2.057 2.046 2.032 2.019 2.017 2.003 1.990 1.979 1.966 1.929 1.926 1.916 1.914 1.908 1.901 1.895 1.881
NC
NC
O
O
O
N
14
^{1}H, CDCl$_3$, 500 MHz
1.00 2.06 0.99 1.03 2.03 2.95 2.17 1.07 1.01 1.01 4.31
10 9 8 7 6 5 4 3 2 1 0
f1 (ppm)

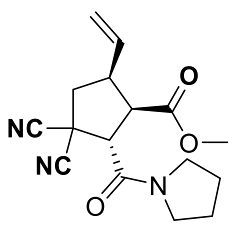

14

$^{13}C\{^1H\}$, $CDCl_3$, 126 MHz

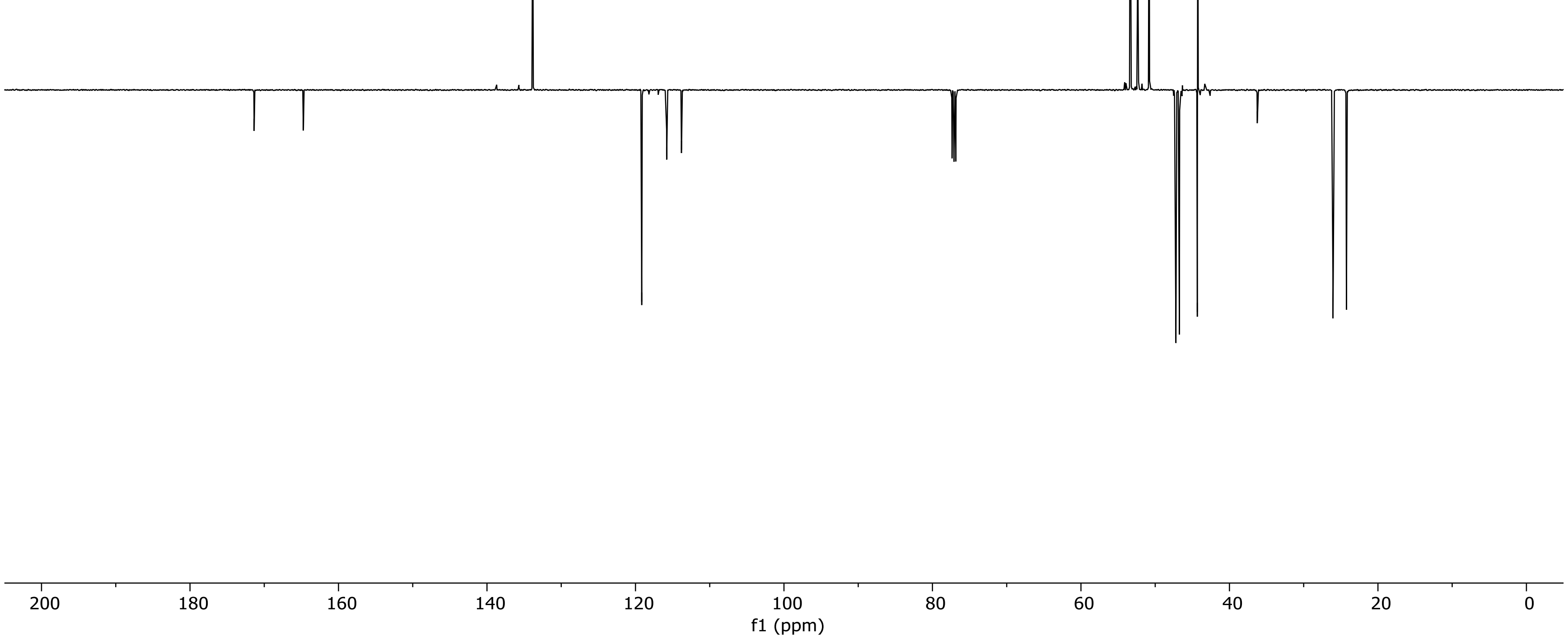

15

^1H, $CDCl_3$, 500 MHz

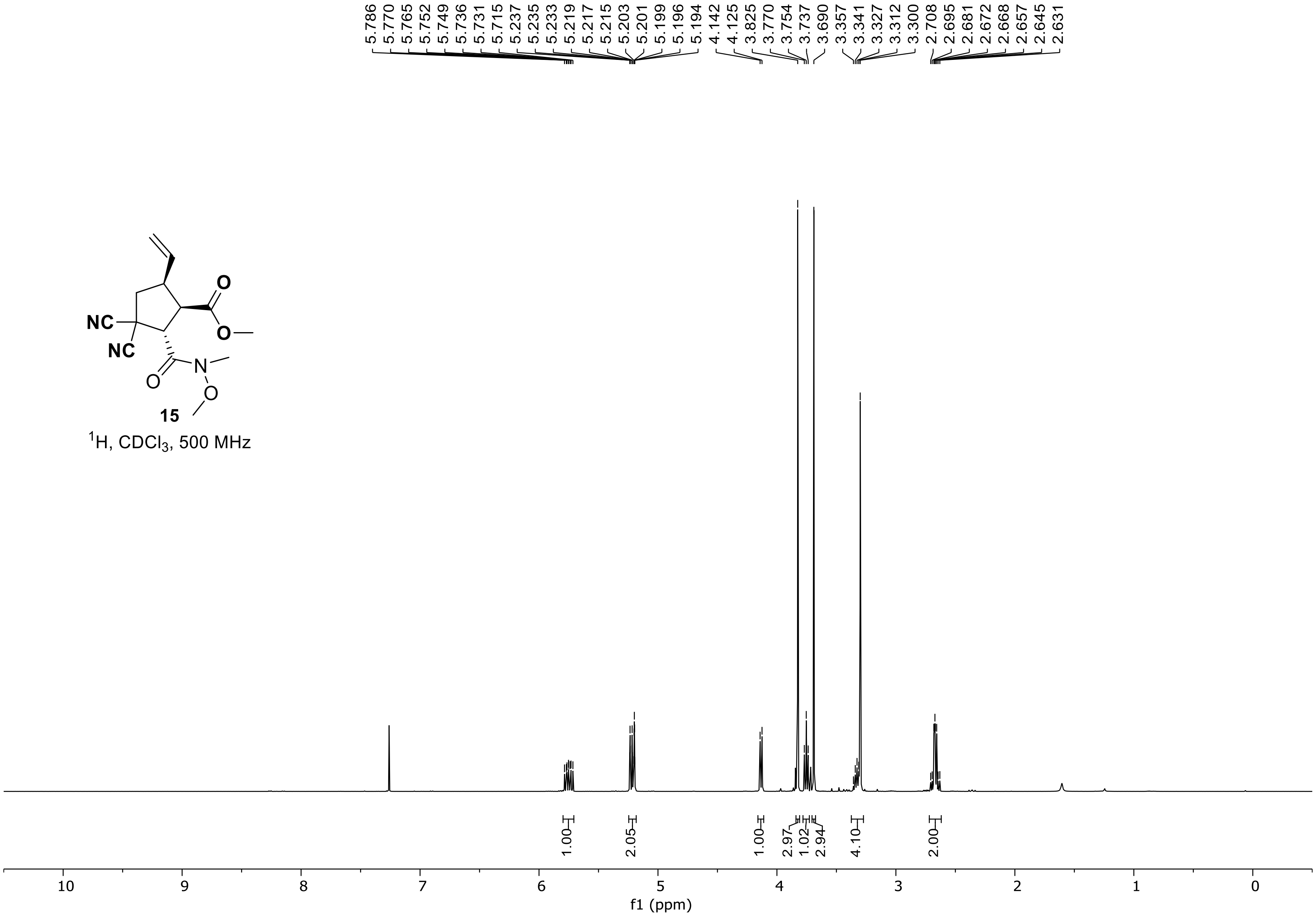

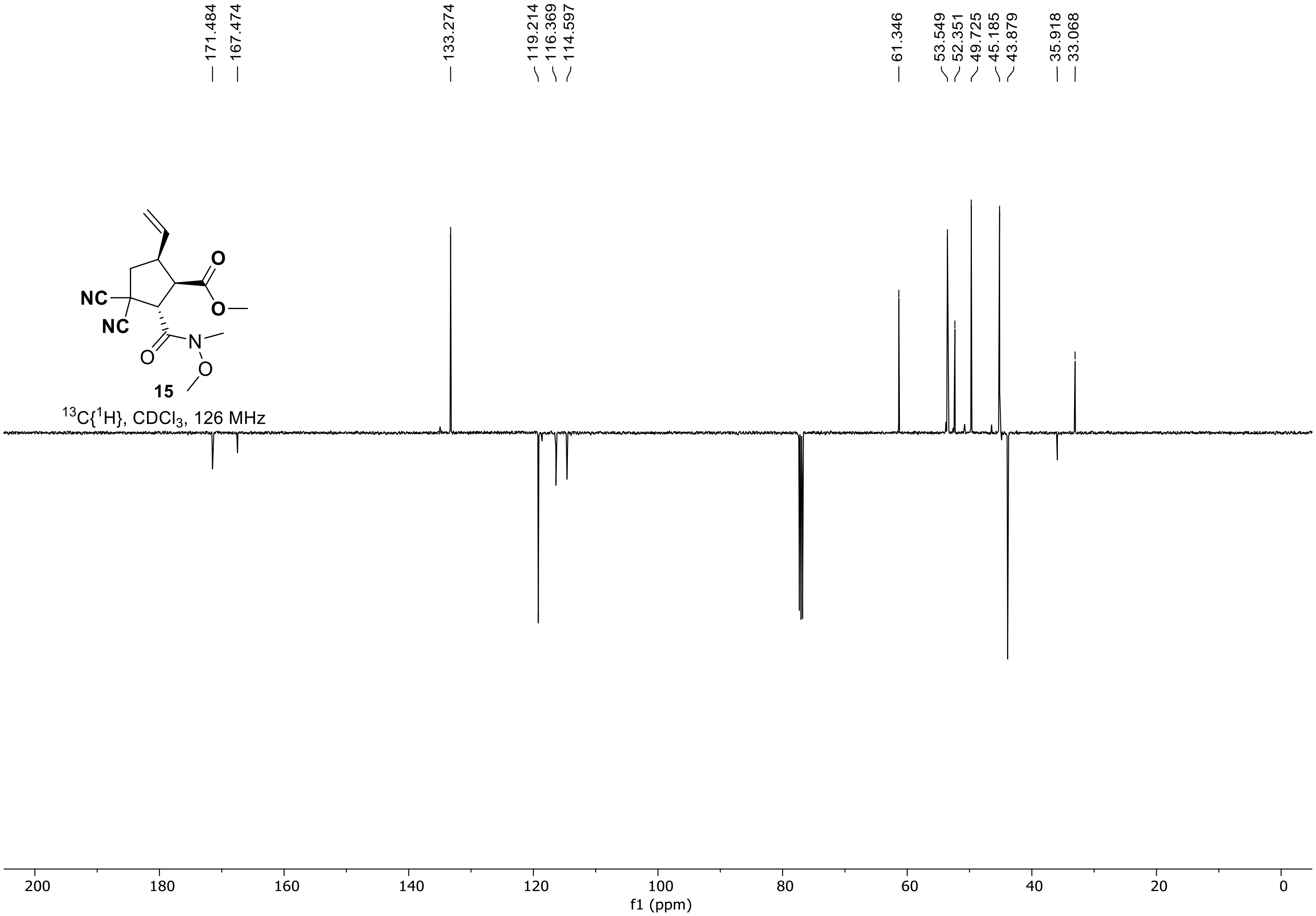
171.484
167.474
133.274
119.214
116.369
114.597
61.346
53.549
52.351
49.725
45.185
43.879
35.918
33.068
NC
NC
O
O
O
N
O
15
$^{13}C\{^{1}H\}$, $CDCl_3$, 126 MHz
200
180
160
140
120
100
80
60
40
20
0
f1 (ppm)

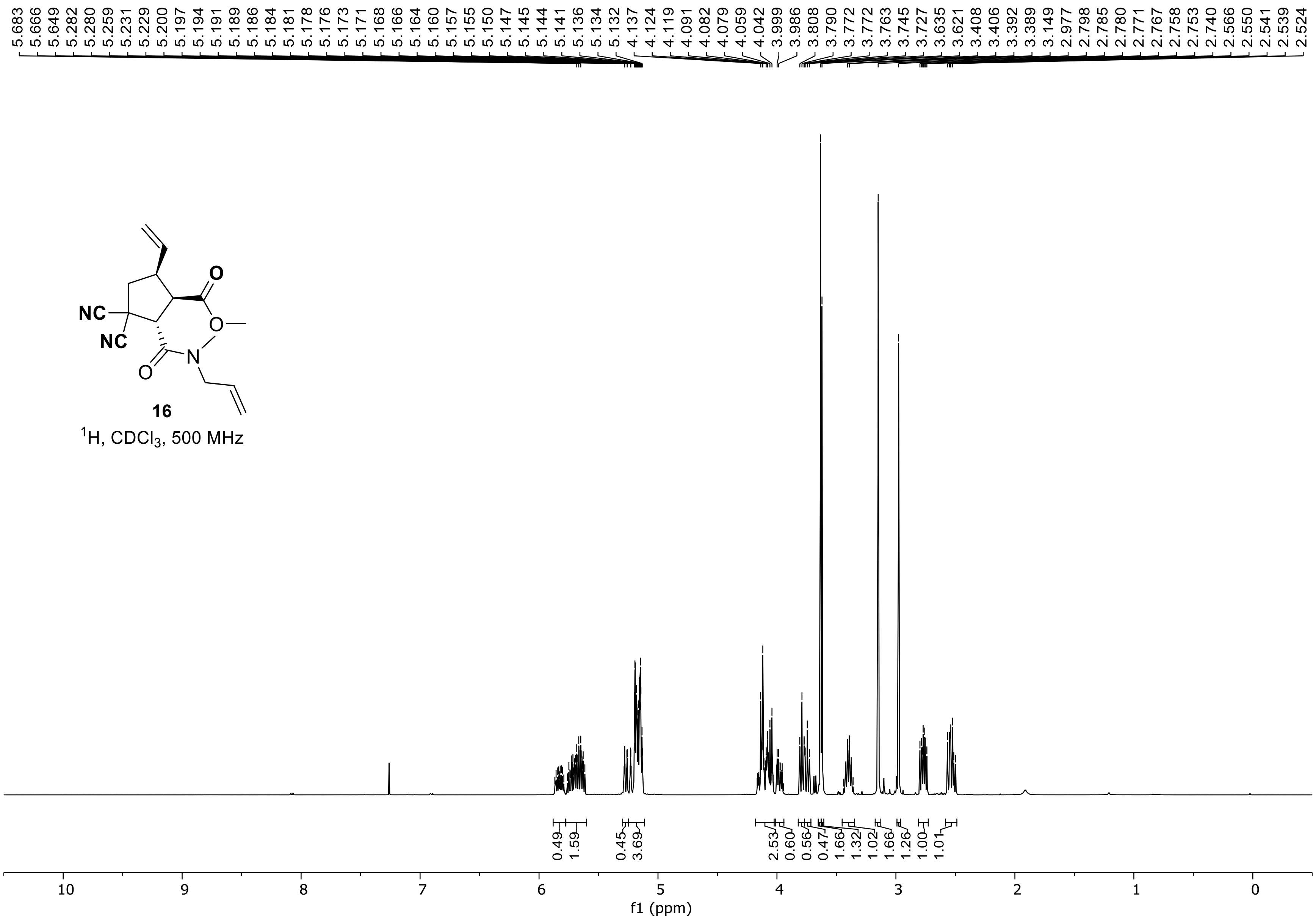
5.683 5.666 5.649 5.282 5.280 5.259 5.231 5.229 5.200 5.197 5.194 5.191 5.189 5.186 5.184 5.181 5.178 5.176 5.173 5.171 5.168 5.166 5.164 5.160 5.157 5.155 5.150 5.147 5.145 5.144 5.141 5.136 5.134 5.132 4.137 4.124 4.119 4.091 4.082 4.079 4.059 4.042 3.999 3.986 3.808 3.790 3.772 3.772 3.763 3.745 3.727 3.635 3.621 3.408 3.406 3.392 3.389 3.149 2.977 2.798 2.785 2.780 2.771 2.767 2.758 2.753 2.740 2.566 2.550 2.541 2.539 2.524
NC
NC
O
O
N
O
16
^{1}H, $CDCl_3$, 500 MHz
0.49 1.59 0.45 3.69 2.53 0.60 0.56 0.47 1.66 1.32 1.02 1.66 1.26 1.00 1.01
10 9 8 7 6 5 4 3 2 1 0
f1 (ppm)

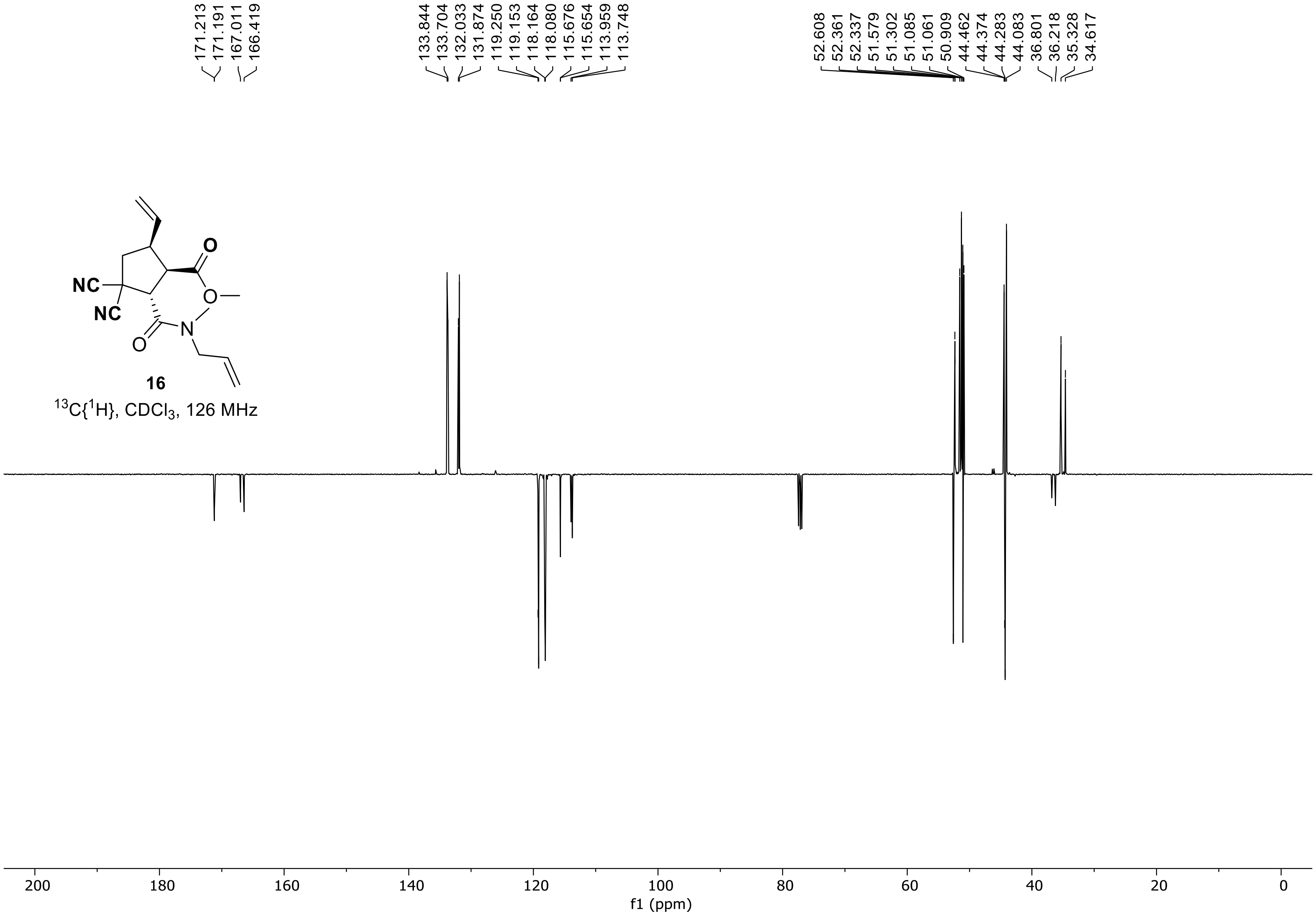
171.213
171.191
167.011
166.419
133.844
133.704
132.033
131.874
119.250
119.153
118.164
118.080
115.676
115.654
113.959
113.748
52.608
52.361
52.337
51.579
51.302
51.085
51.061
50.909
44.462
44.374
44.283
44.083
36.801
36.218
35.328
34.617
O
NC
NC
O
N
O
16
$^{13}C\{^1H\}$, $CDCl_3$, 126 MHz
200
180
160
140
120
100
80
60
40
20
0
f1 (ppm)

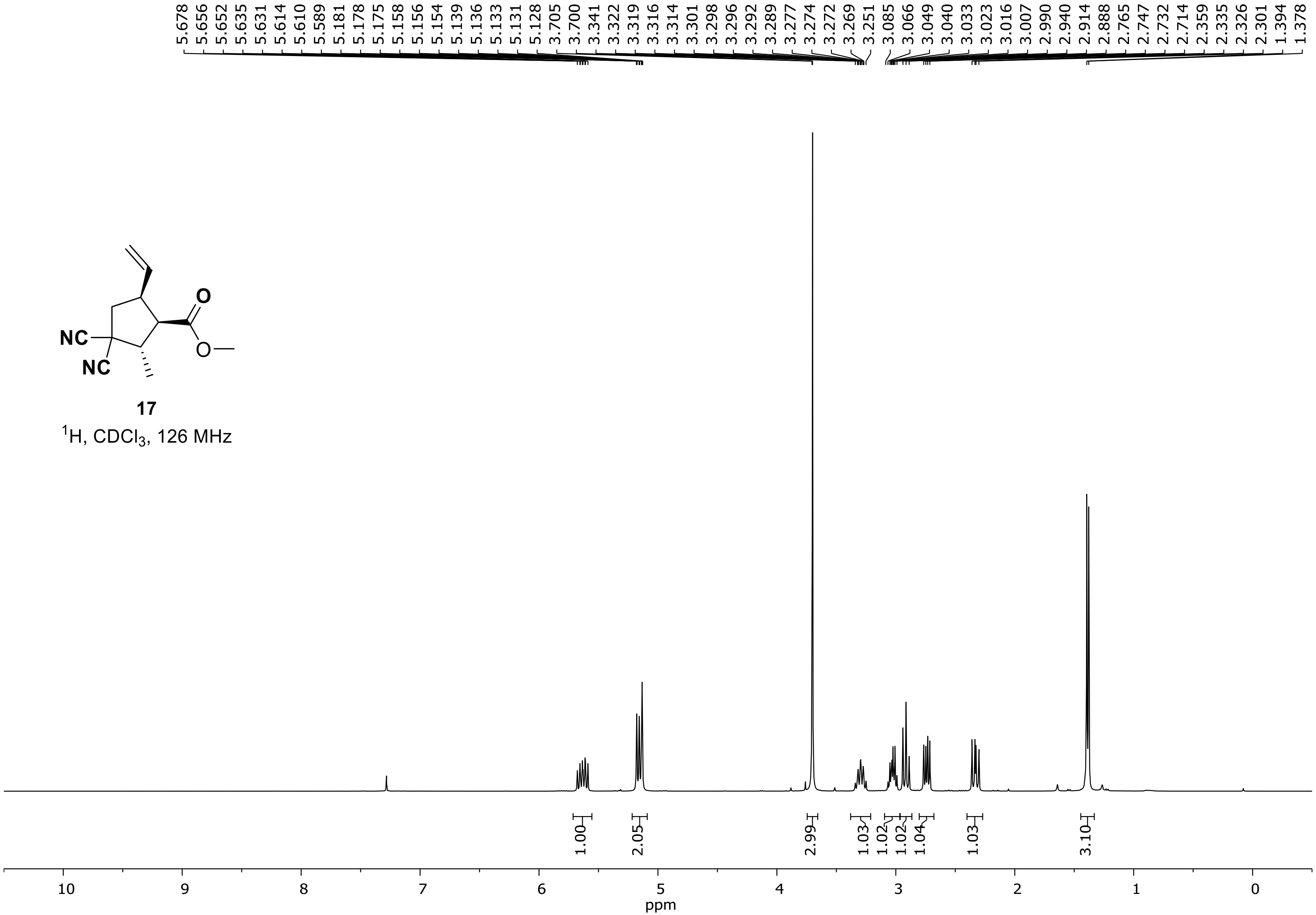
O
O
NC
NC
17
^{1}H, $CDCl_3$, 126 MHz
5.678
5.656
5.652
5.635
5.631
5.614
5.610
5.589
5.181
5.178
5.175
5.158
5.156
5.154
5.139
5.136
5.133
5.131
5.128
3.705
3.700
3.341
3.322
3.319
3.316
3.314
3.301
3.298
3.296
3.292
3.289
3.277
3.274
3.272
3.269
3.251
3.085
3.066
3.049
3.040
3.033
3.023
3.016
3.007
2.990
2.940
2.914
2.888
2.765
2.747
2.732
2.714
2.359
2.335
2.326
2.301
1.394
1.378
1.00
2.05
2.99
1.03
1.02
1.02
1.04
1.03
3.10
10
9
8
7
6
5
4
3
2
1
0
ppm

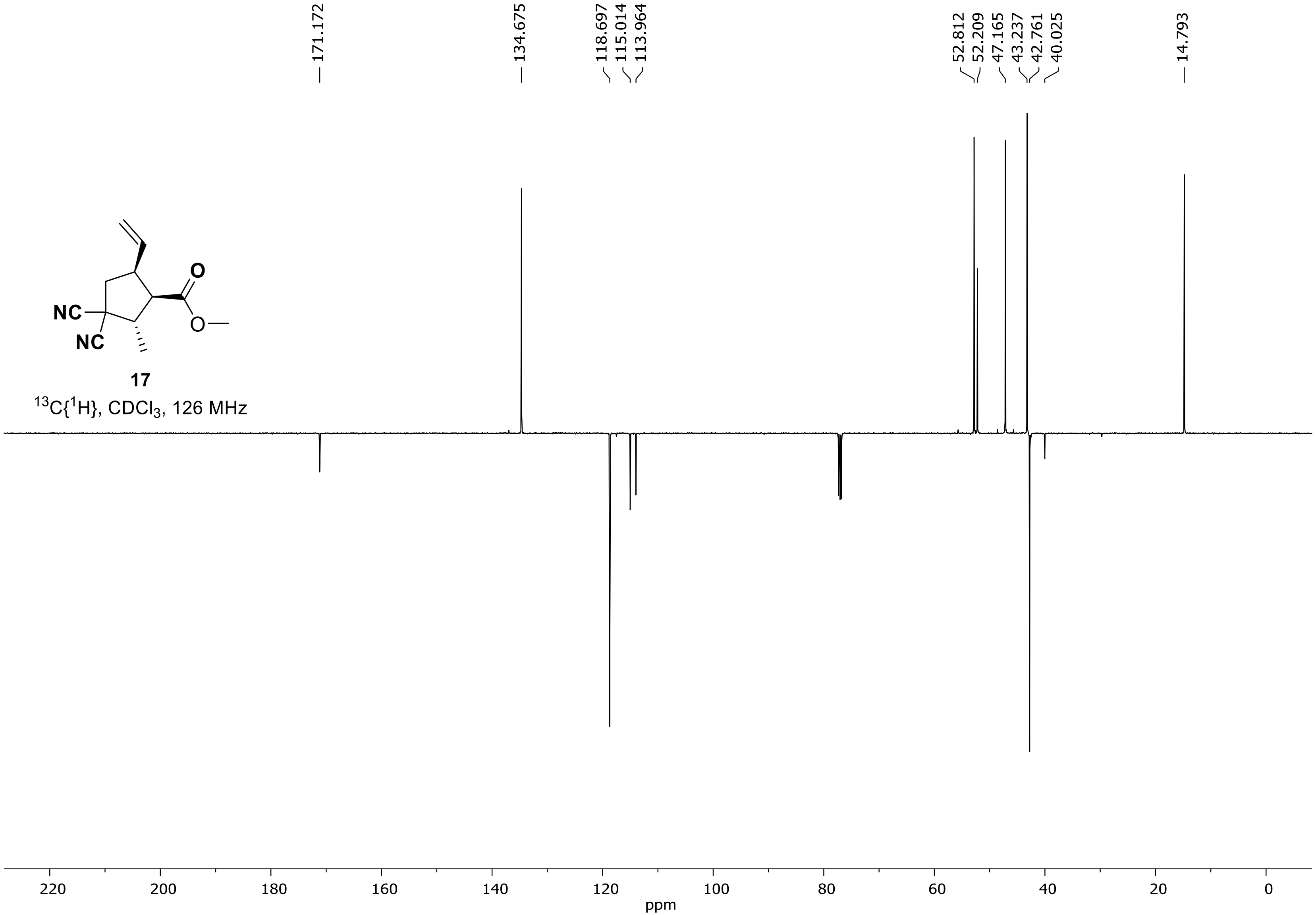

17

$^{13}C\{^{1}H\}$, $CDCl_3$, 126 MHz

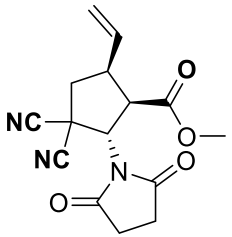

18

^{1}H, $CDCl_3$, 126 MHz

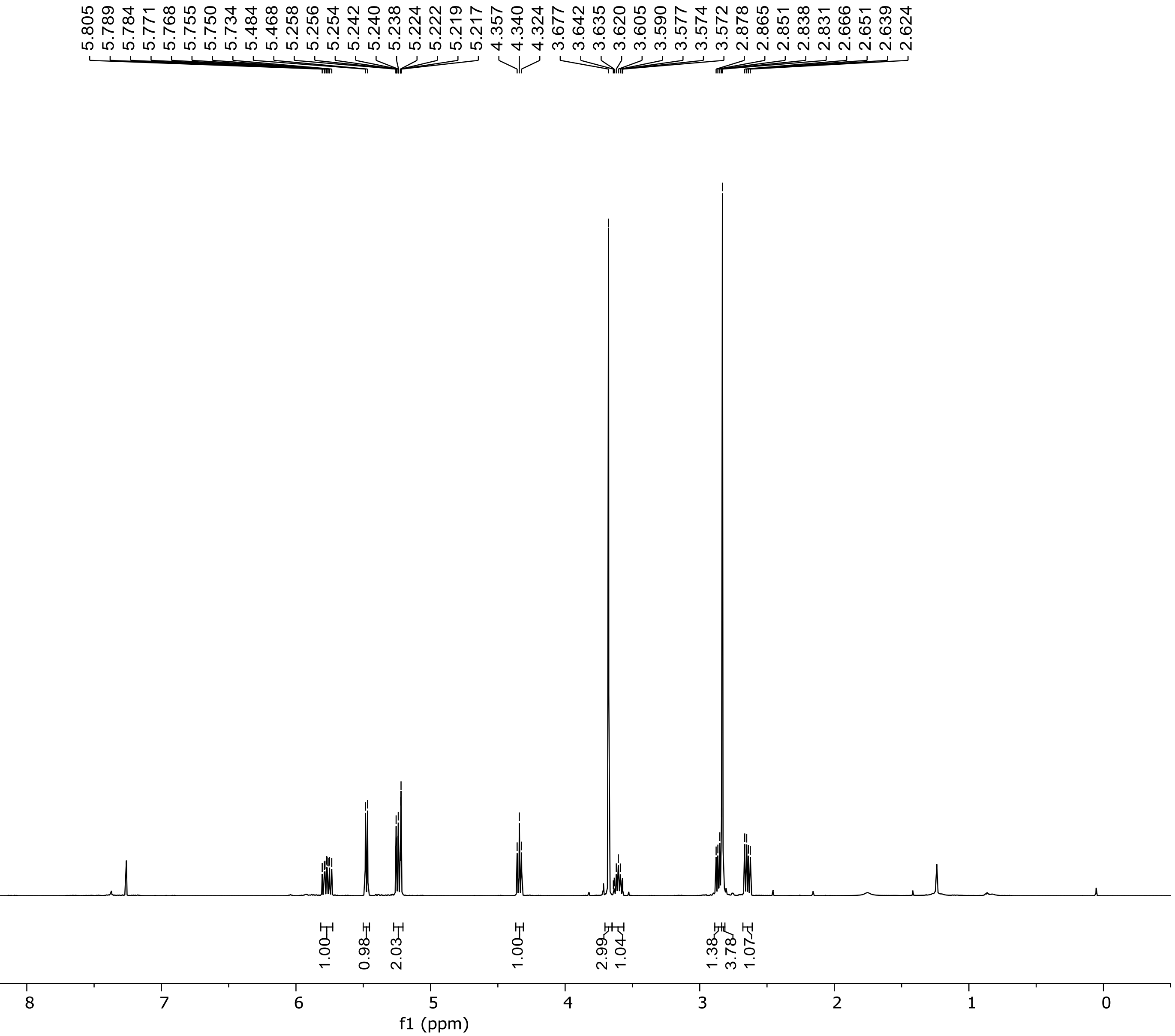

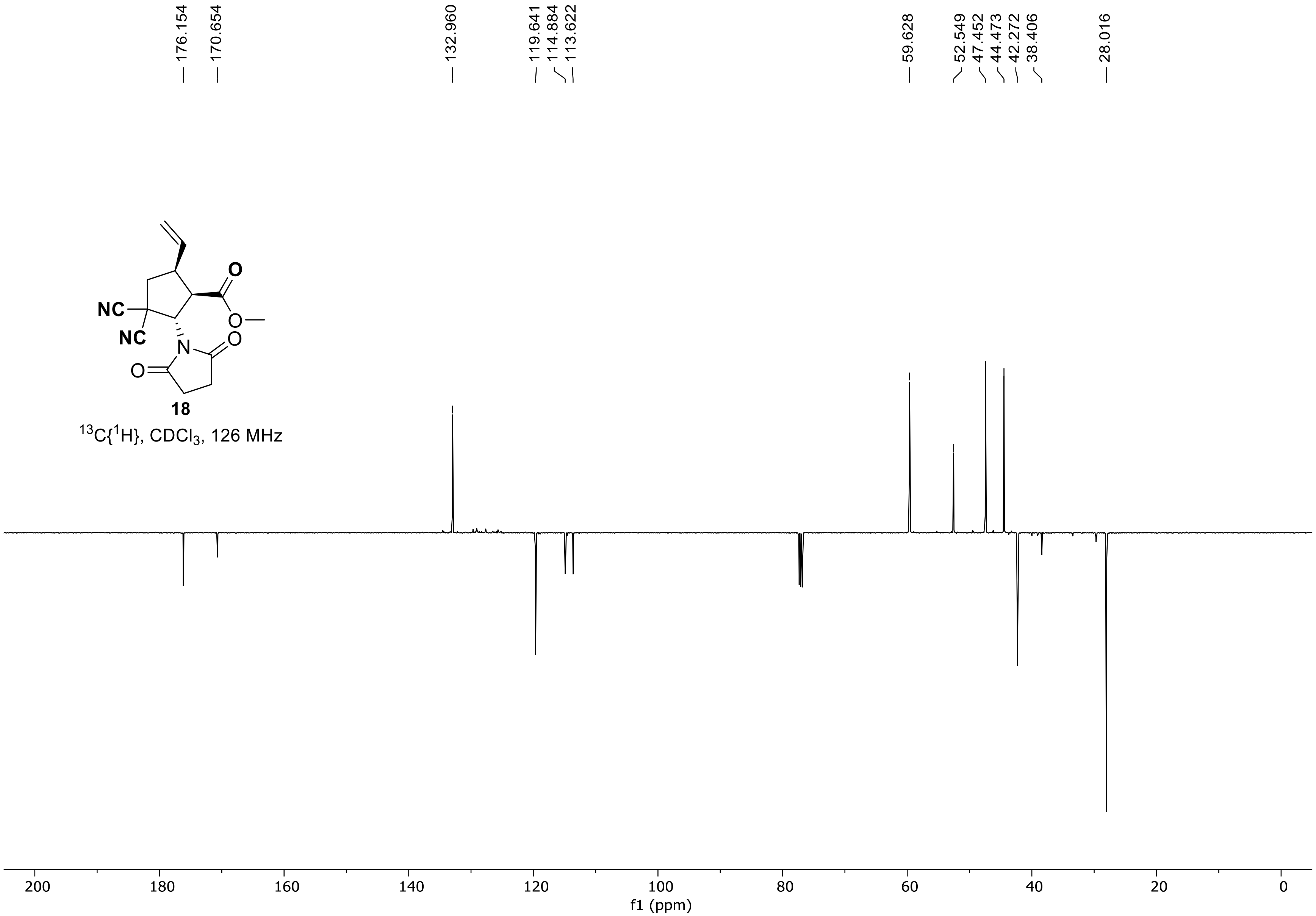
176.154
170.654
132.960
119.641
114.884
113.622
59.628
52.549
47.452
44.473
42.272
38.406
28.016
NC
NC
O
O
N
O
O
18
$^{13}C\{^1H\}$, $CDCl_3$, 126 MHz
200
180
160
140
120
100
80
60
40
20
0
f1 (ppm)

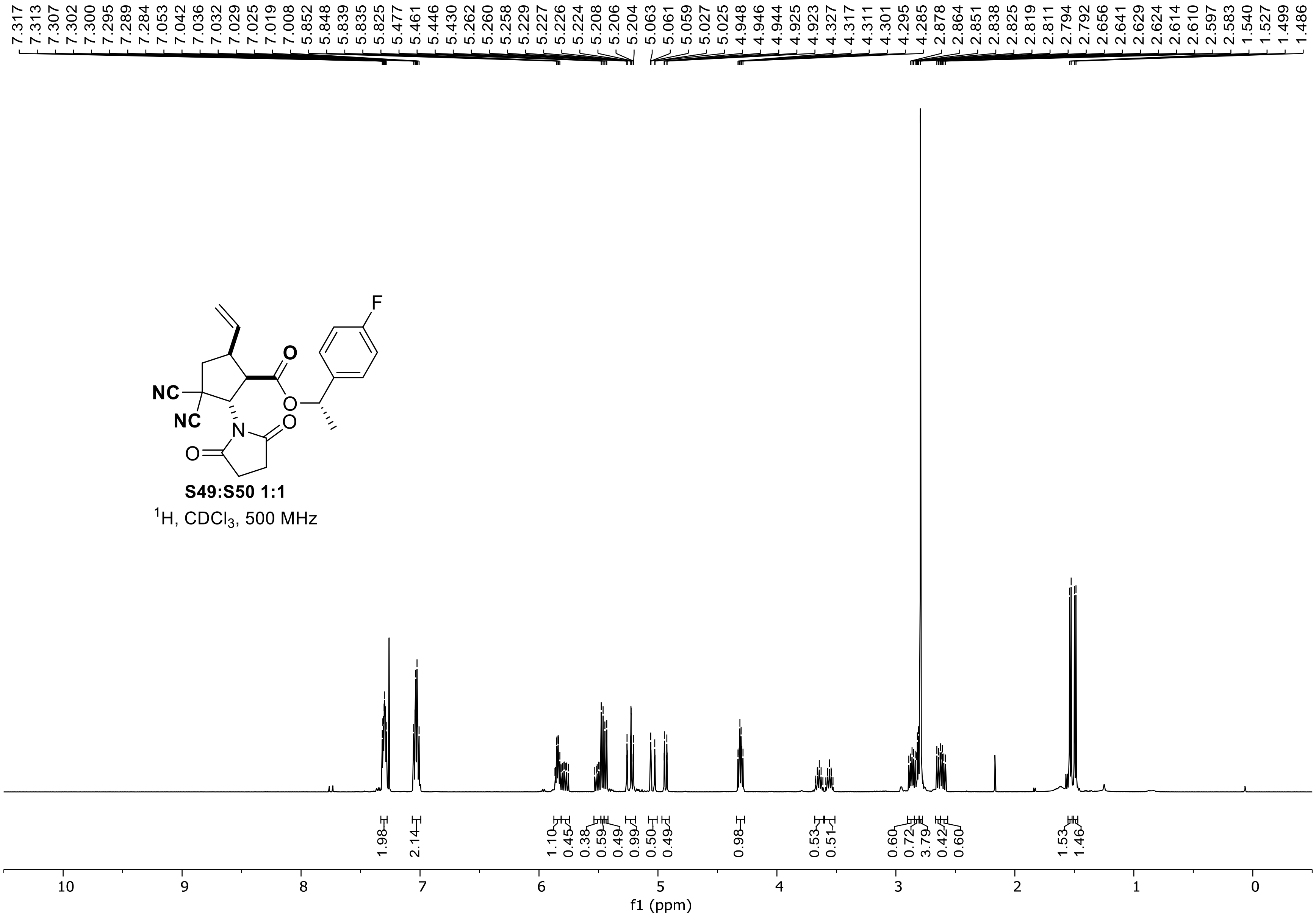
7.317 7.313 7.307 7.302 7.300 7.295 7.289 7.284 7.053 7.042 7.036 7.032 7.029 7.025 7.019 7.008 5.852 5.848 5.839 5.835 5.825 5.477 5.461 5.446 5.430 5.262 5.260 5.258 5.229 5.227 5.226 5.224 5.208 5.206 5.204 5.063 5.061 5.059 5.027 5.025 4.948 4.946 4.944 4.925 4.923 4.327 4.317 4.311 4.301 4.295 4.285 2.878 2.864 2.851 2.838 2.825 2.819 2.811 2.794 2.792 2.656 2.641 2.629 2.624 2.614 2.610 2.597 2.583 1.540 1.527 1.499 1.486
F
O
O
NC
NC
N
O
O
S49:S50 1:1
1H, CDCl3, 500 MHz
1.98
2.14
1.10
0.45
0.38
0.59
0.49
0.99
0.50
0.49
0.98
0.53
0.51
0.60
0.72
3.79
0.42
0.60
1.53
1.46
10
9
8
7
6
5
4
3
2
1
0
f1 (ppm)

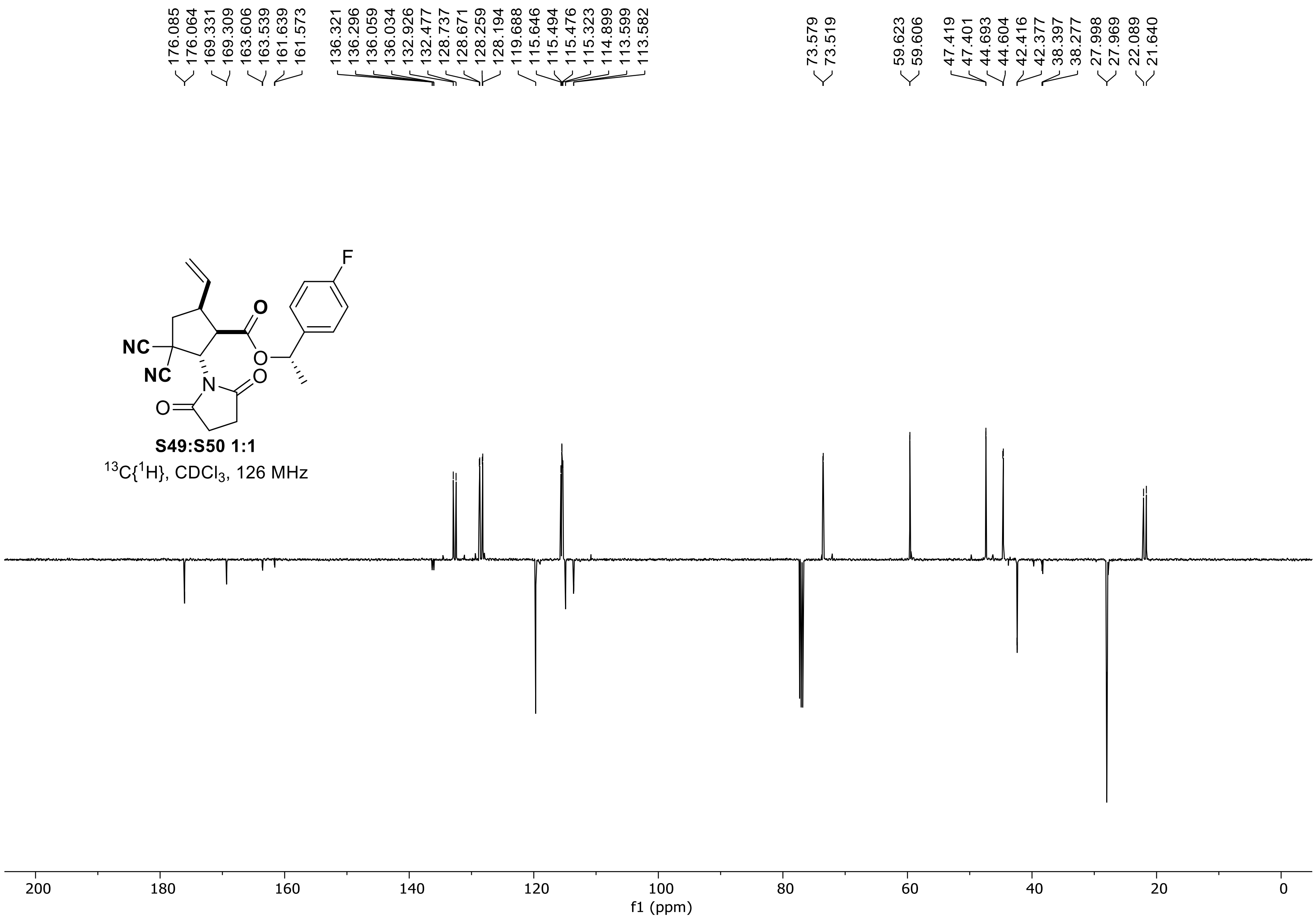
176.085
176.064
169.331
169.309
163.606
163.539
161.639
161.573
136.321
136.296
136.059
136.034
132.926
132.477
128.737
128.671
128.259
128.194
119.688
115.646
115.494
115.476
115.323
114.899
113.599
113.582
73.579
73.519
59.623
59.606
47.419
47.401
44.693
44.604
42.416
42.377
38.397
38.277
27.998
27.969
22.089
21.640
F
O
NC
NC
N
O
O
O
S49:S50 1:1
$^{13}C\{^{1}H\}$, $CDCl_3$, 126 MHz
200
180
160
140
120
100
80
60
40
20
0
f1 (ppm)

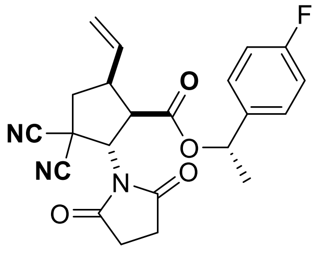

S49:S50 1:1

$^{19}F\{^{1}H\}$, $CDCl_3$, 377 MHz

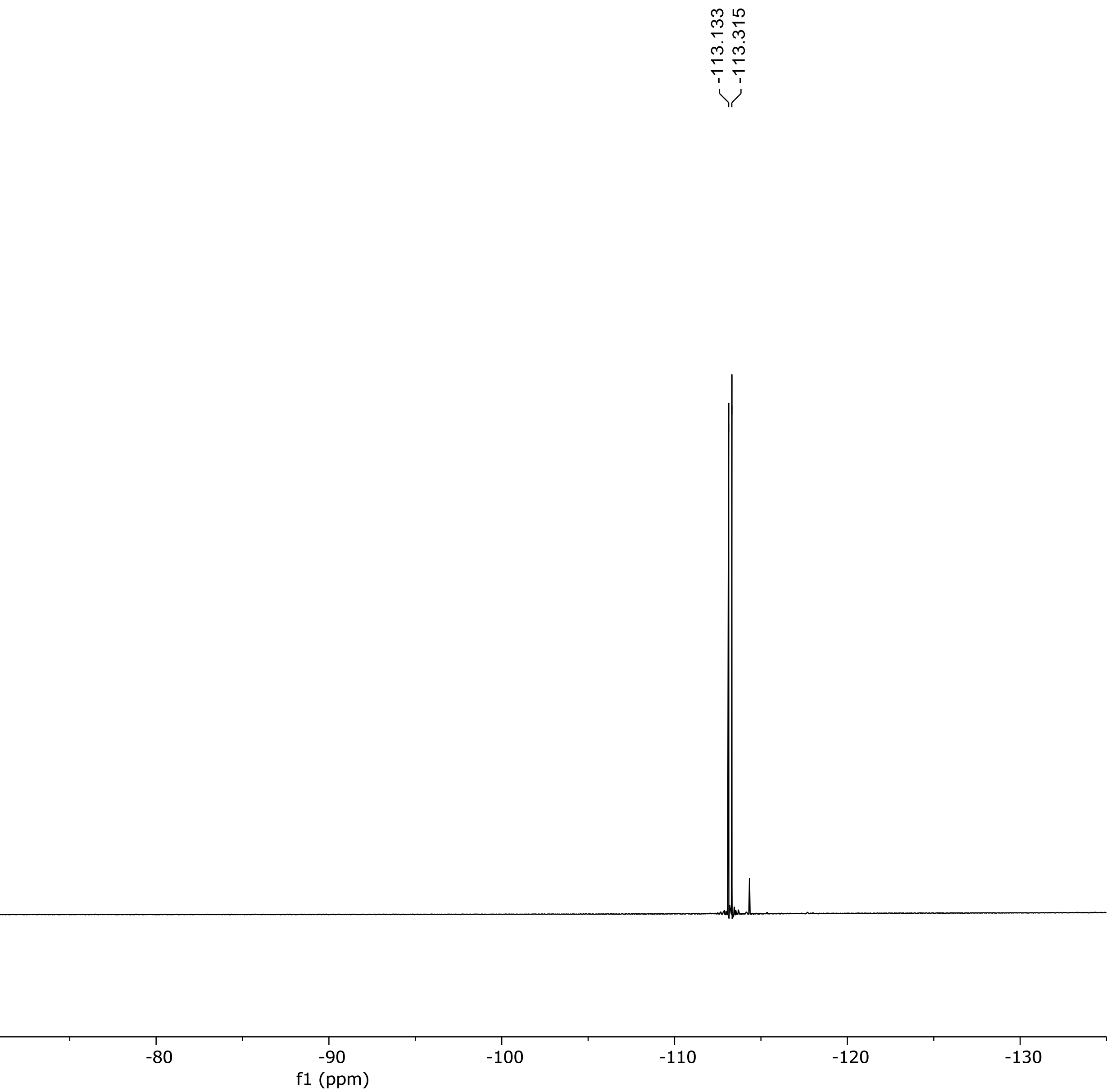

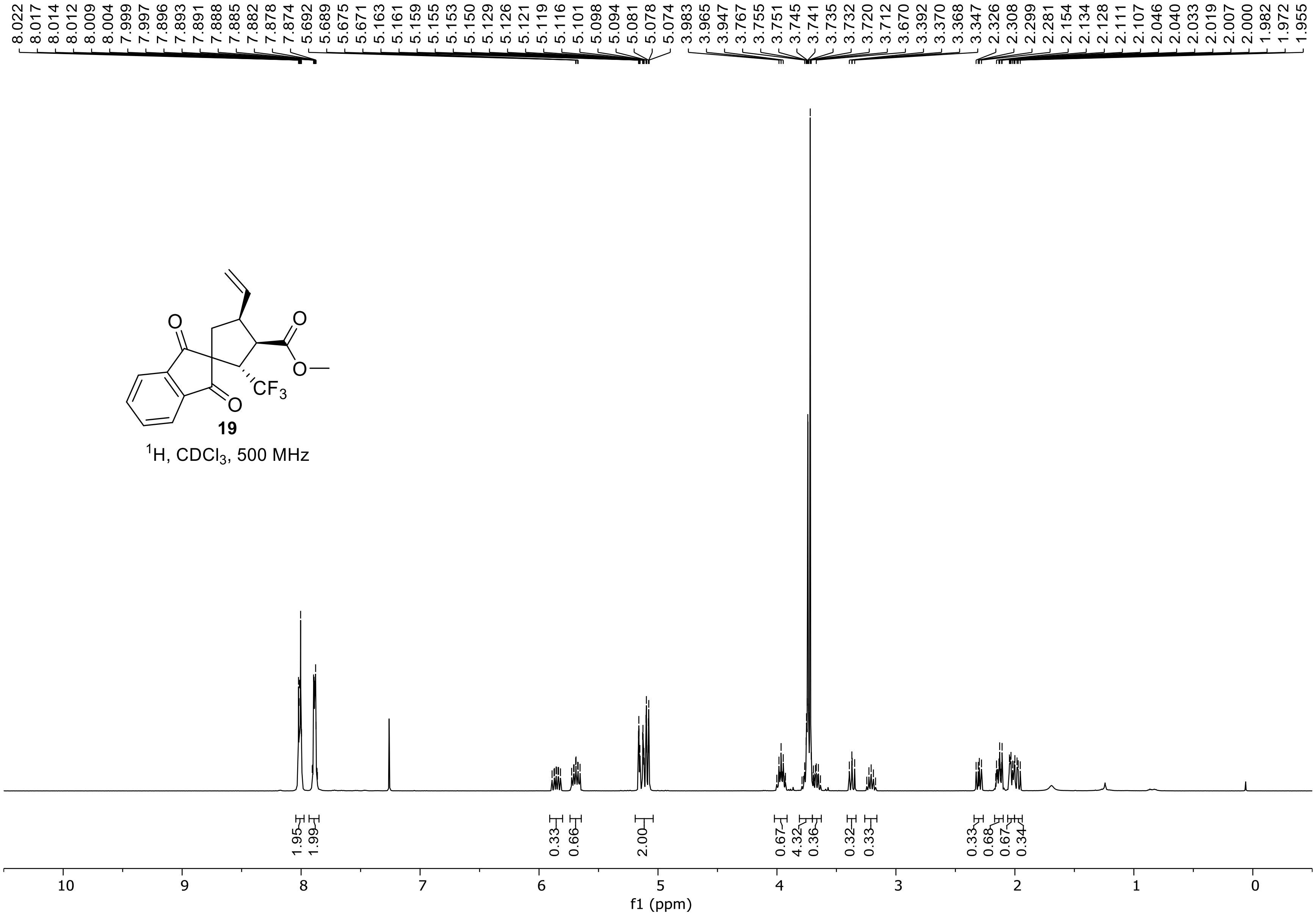

19

^{1}H, $CDCl_3$, 500 MHz

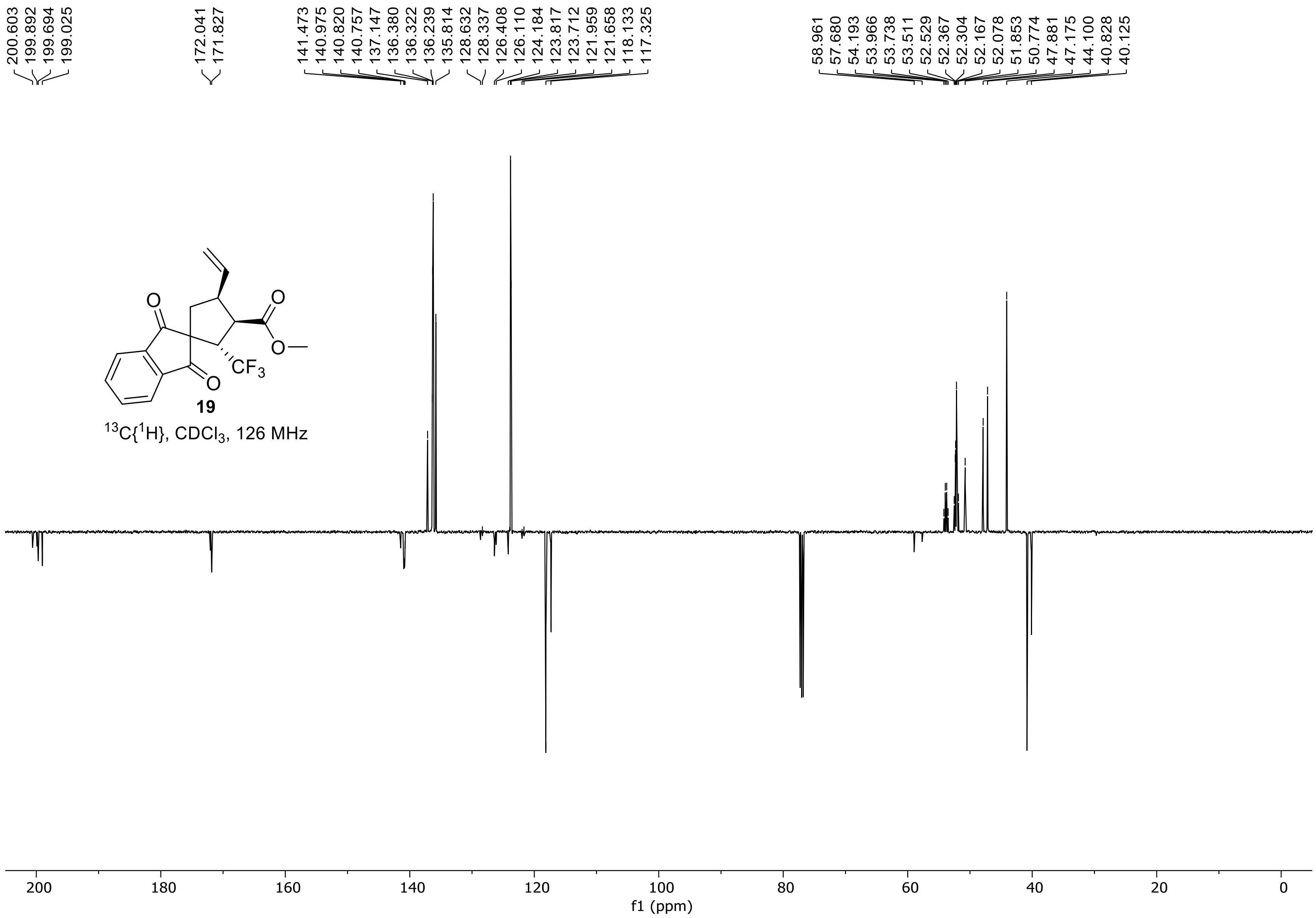
200.603
199.892
199.694
199.025
172.041
171.827
141.473
140.975
140.820
140.757
137.147
136.380
136.322
136.239
135.814
128.632
128.337
126.408
126.110
124.184
123.817
123.712
121.959
121.658
118.133
117.325
58.961
57.680
54.193
53.966
53.738
53.511
52.529
52.367
52.304
52.167
52.078
51.853
50.774
47.881
47.175
44.100
40.828
40.125
O
O
O
O
CF3
19
13C{1H}, CDCl3, 126 MHz
200
180
160
140
120
100
80
60
40
20
0
f1 (ppm)

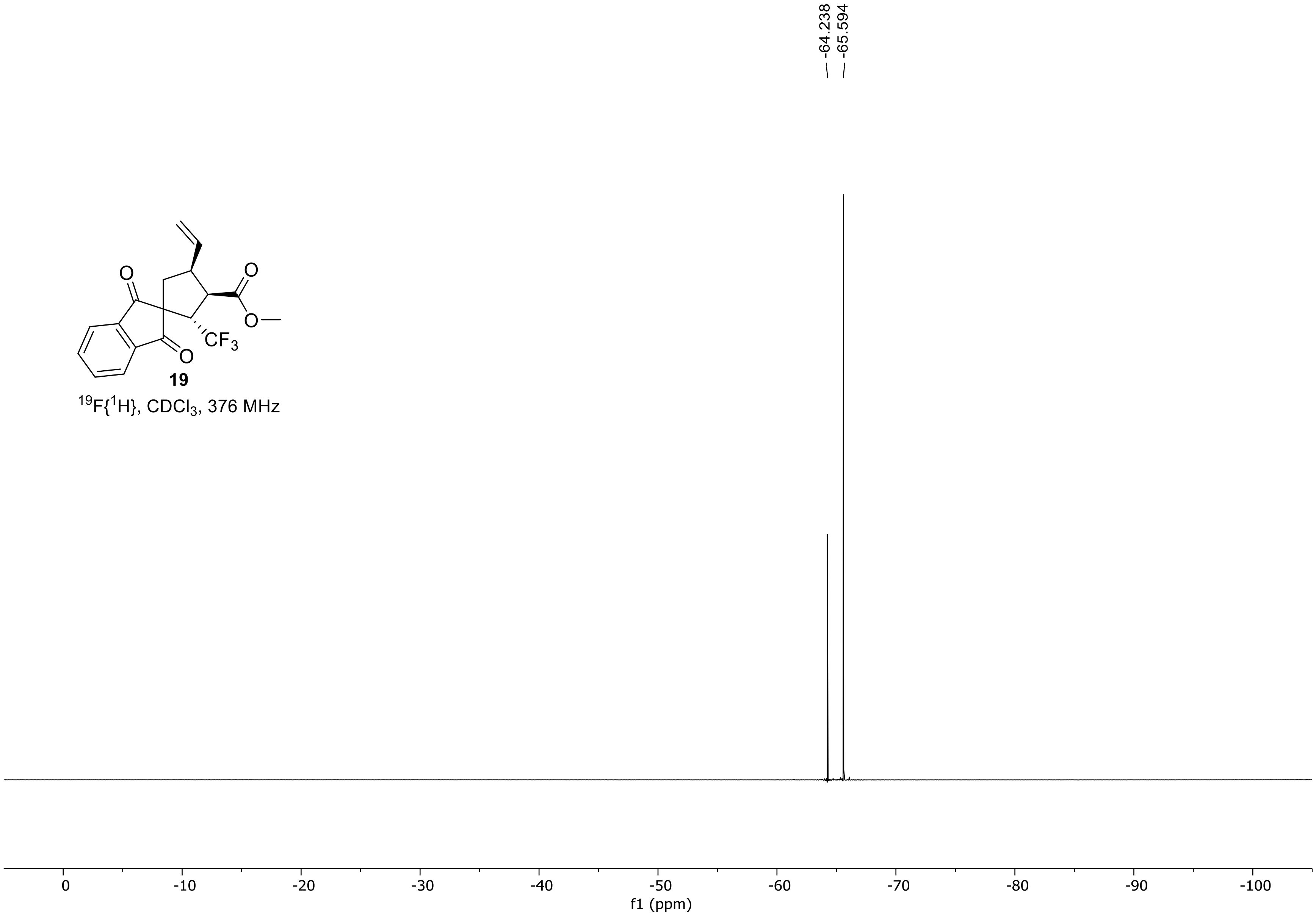
O
O
O
O
CF_3
19
$^{19}F\{^1H\}$, $CDCl_3$, 376 MHz
-64.238
-65.594
0
-10
-20
-30
-40
-50
-60
-70
-80
-90
-100
f1 (ppm)

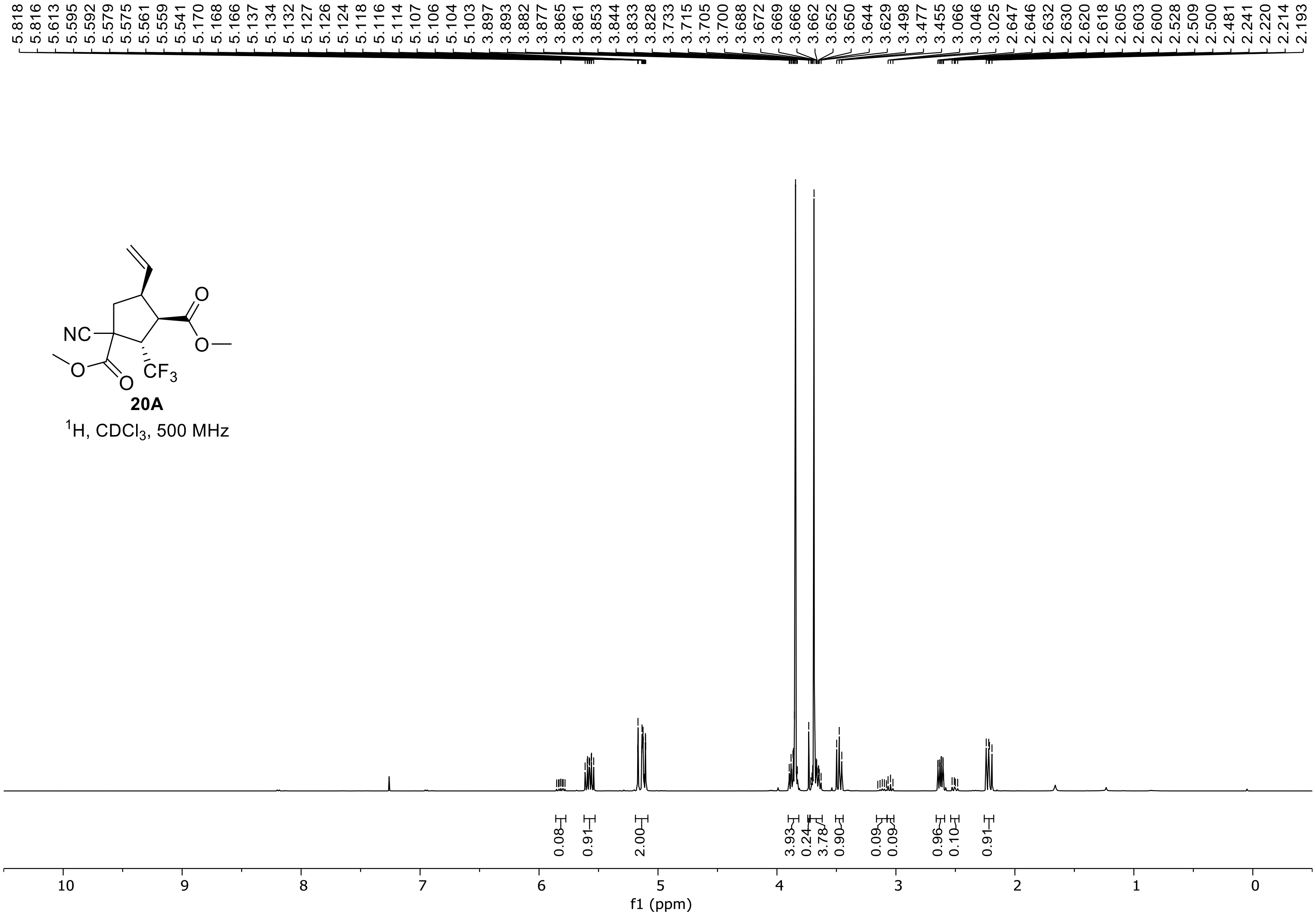
NC
O
O
O
O
CF3
20A
1H, CDCl3, 500 MHz
5.818
5.816
5.613
5.595
5.592
5.579
5.575
5.561
5.559
5.541
5.170
5.168
5.166
5.137
5.134
5.132
5.127
5.126
5.124
5.118
5.116
5.114
5.107
5.106
5.104
5.103
3.897
3.893
3.882
3.877
3.865
3.861
3.853
3.844
3.833
3.828
3.733
3.715
3.705
3.700
3.688
3.672
3.669
3.666
3.662
3.652
3.650
3.644
3.629
3.498
3.477
3.455
3.066
3.046
3.025
2.647
2.646
2.632
2.630
2.620
2.618
2.605
2.603
2.600
2.528
2.509
2.500
2.481
2.241
2.220
2.214
2.193
0.08
0.91
2.00
3.93
0.24
3.78
0.90
0.09
0.09
0.96
0.10
0.91
10
9
8
7
6
5
4
3
2
1
0
f1 (ppm)

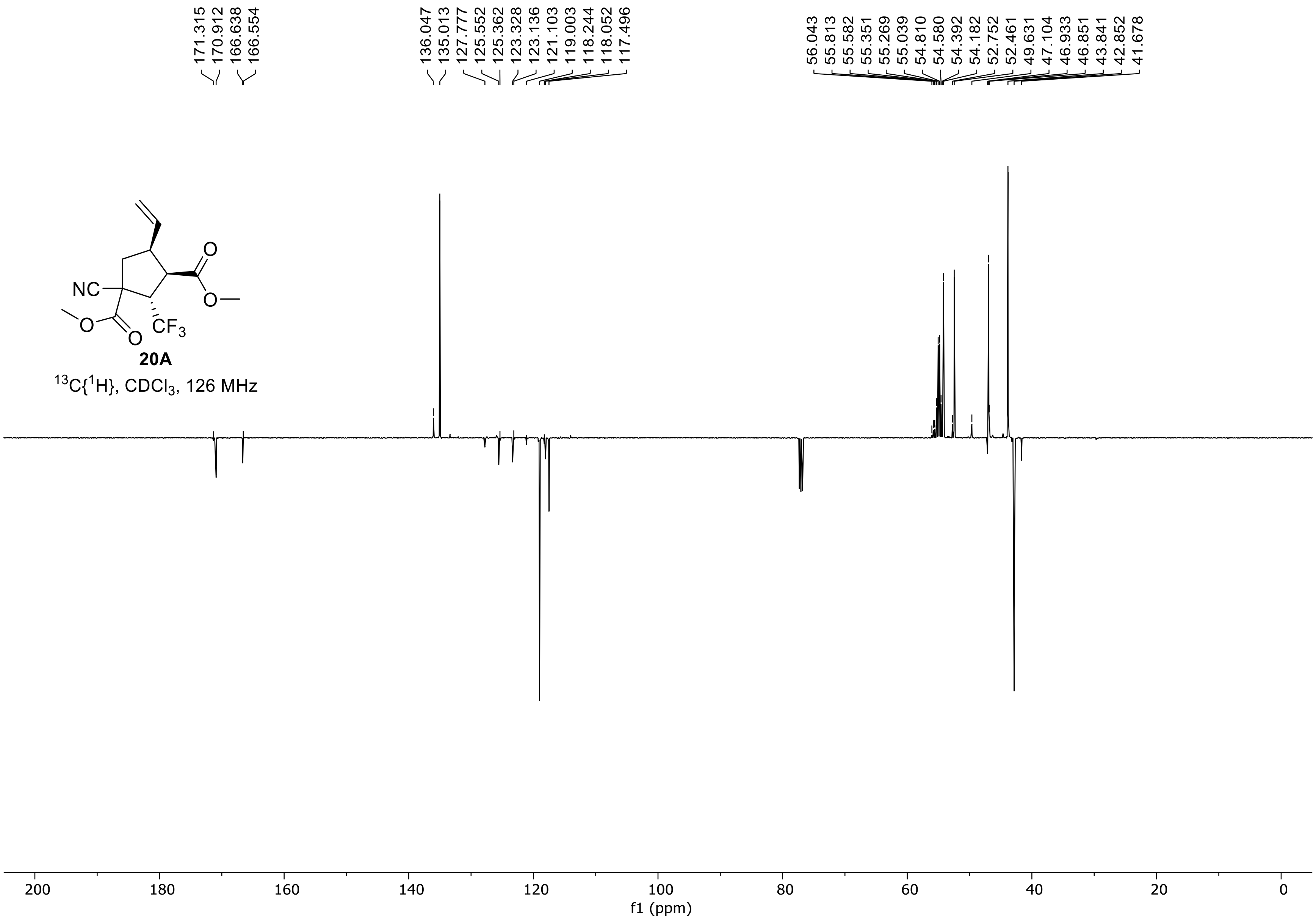
171.315
170.912
166.638
166.554
136.047
135.013
127.777
125.552
125.362
123.328
123.136
121.103
119.003
118.244
118.052
117.496
56.043
55.813
55.582
55.351
55.269
55.039
54.810
54.580
54.392
54.182
52.752
52.461
49.631
47.104
46.933
46.851
43.841
42.852
41.678
NC
O
O
O
O
CF3
20A
$^{13}C\{^{1}H\}$, $CDCl_3$, 126 MHz
200
180
160
140
120
100
80
60
40
20
0
f1 (ppm)

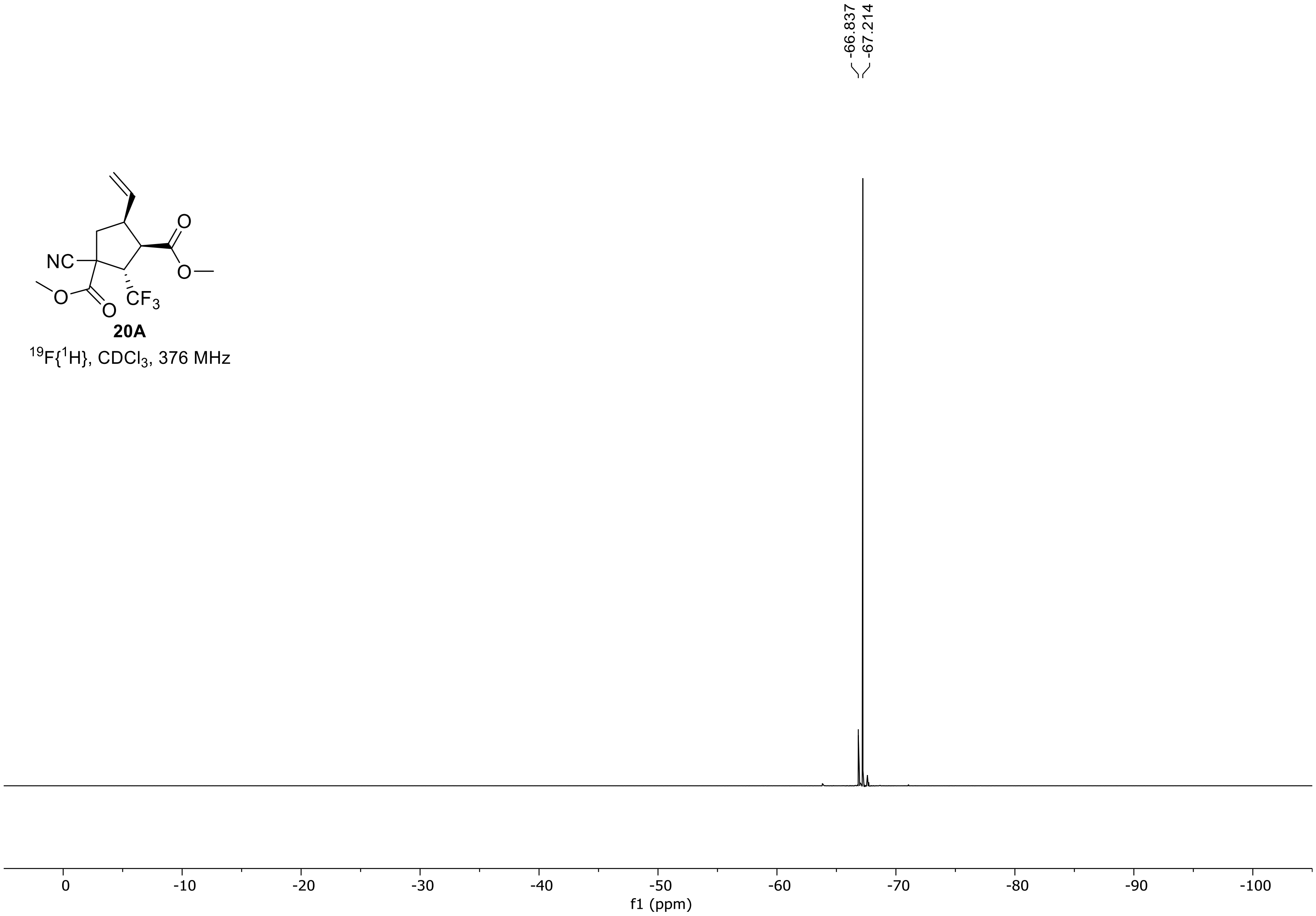
-66.837
-67.214
NC
O
O
O
O
CF3
20A
19F{1H}, CDCl3, 376 MHz
0
-10
-20
-30
-40
-50
-60
-70
-80
-90
-100
f1 (ppm)

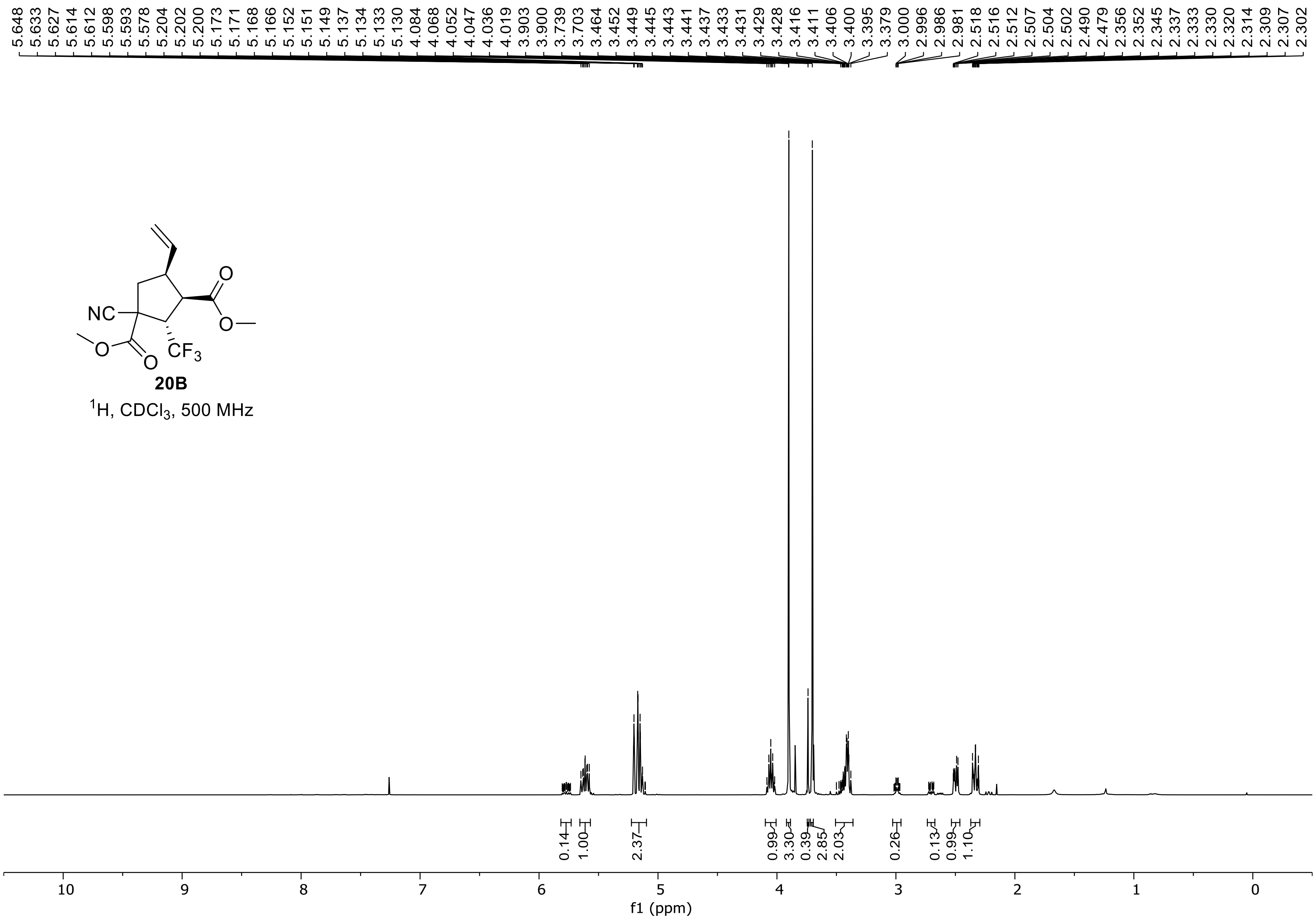
5.648 5.633 5.627 5.614 5.612 5.598 5.593 5.578 5.204 5.202 5.200 5.173 5.171 5.168 5.166 5.152 5.151 5.149 5.137 5.134 5.133 5.130 4.084 4.068 4.052 4.047 4.036 4.019 3.903 3.900 3.739 3.703 3.464 3.452 3.449 3.445 3.443 3.441 3.437 3.433 3.431 3.429 3.428 3.416 3.411 3.406 3.400 3.395 3.379 3.000 2.996 2.986 2.981 2.518 2.516 2.512 2.507 2.504 2.502 2.490 2.479 2.356 2.352 2.345 2.337 2.333 2.330 2.320 2.314 2.309 2.307 2.302
NC
O
O
O
O
CF3
20B
1H, CDCl3, 500 MHz
0.14 1.00 2.37 0.99 3.30 0.39 2.85 2.03 0.26 0.13 0.99 1.10
10 9 8 7 6 5 4 3 2 1 0
f1 (ppm)

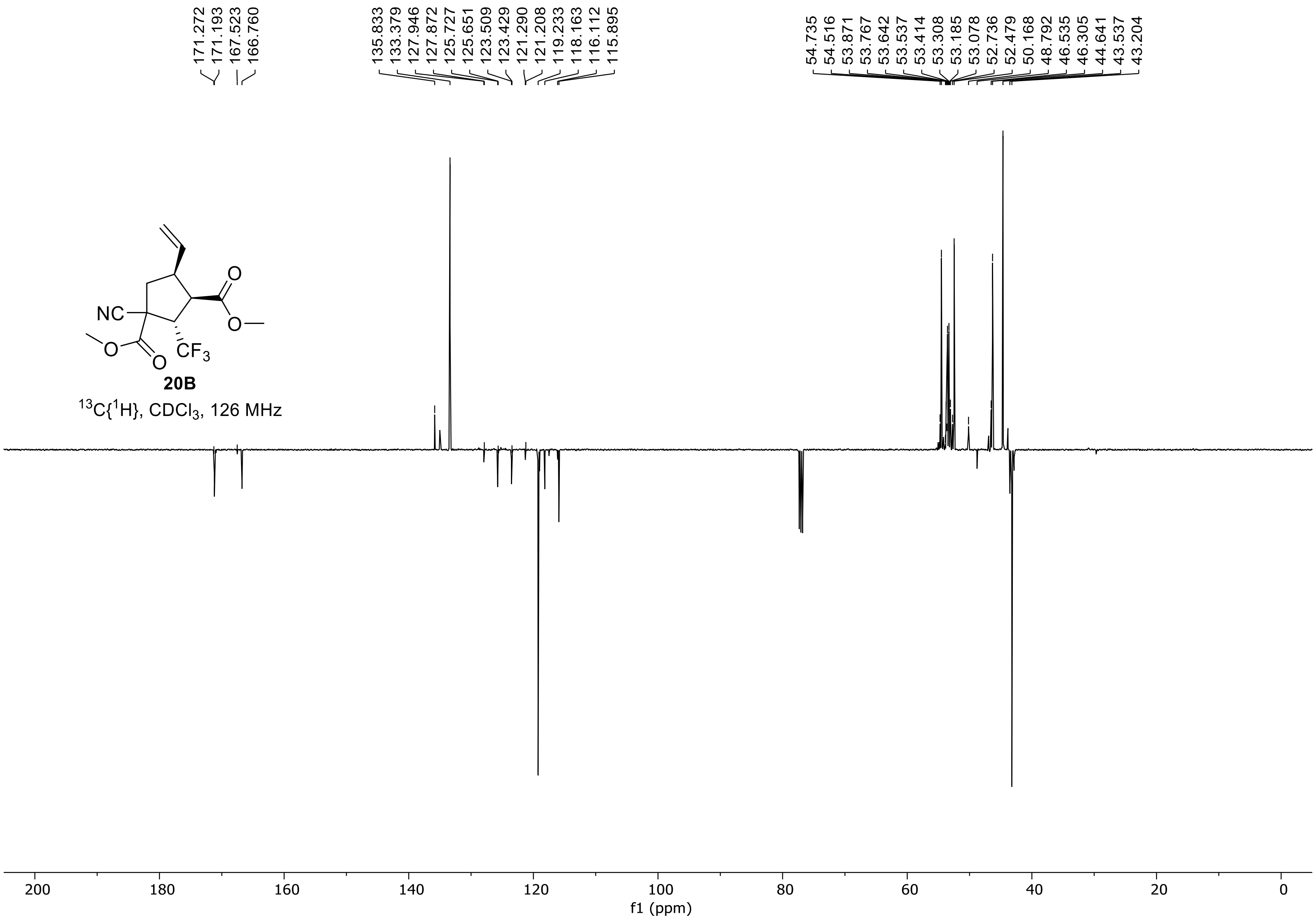

20B

$^{13}C\{^{1}H\}$, $CDCl_3$, 126 MHz

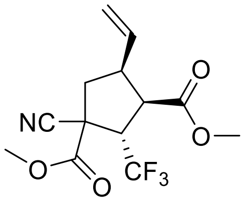

20B

$^{19}F\{^1H\}$, $CDCl_3$, 376 MHz

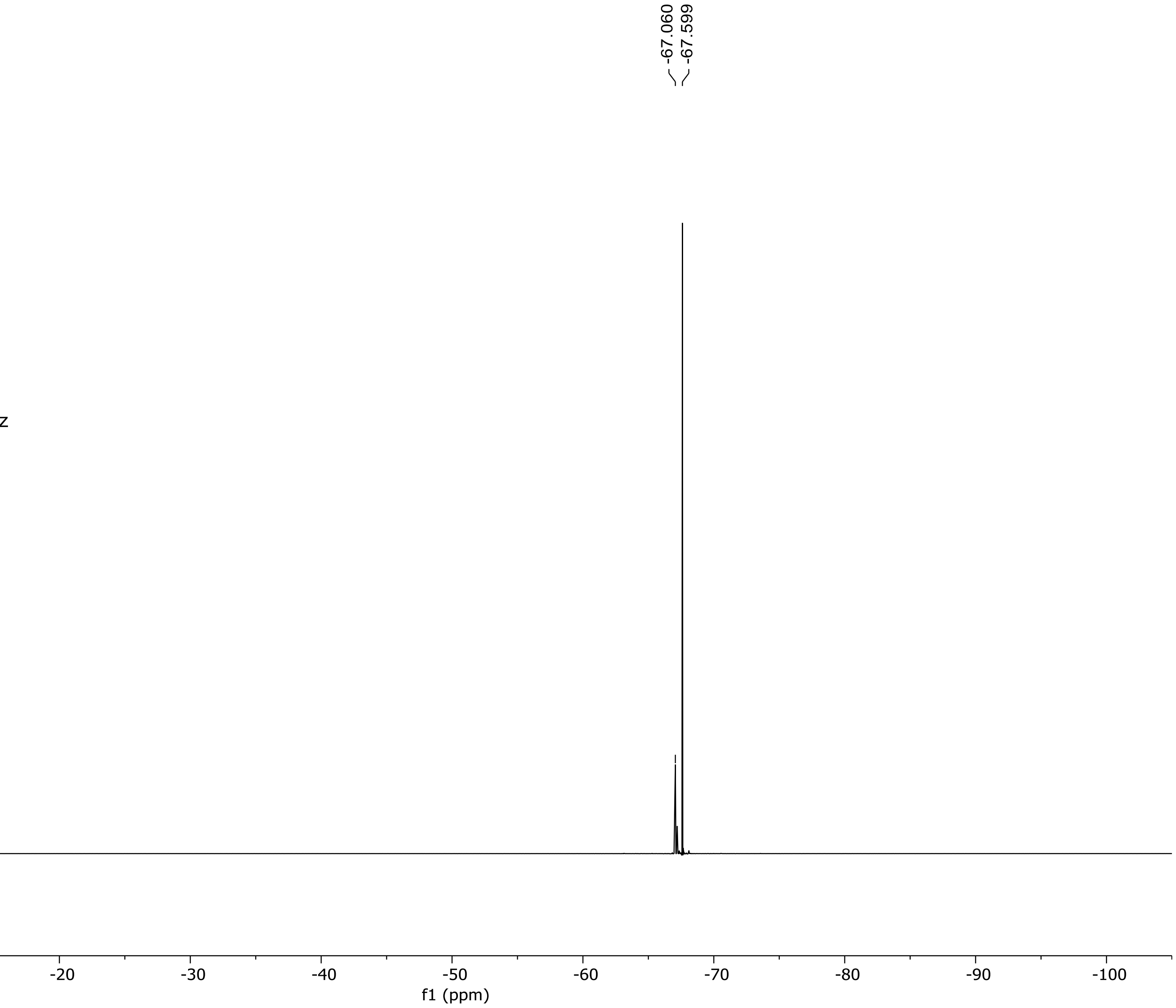

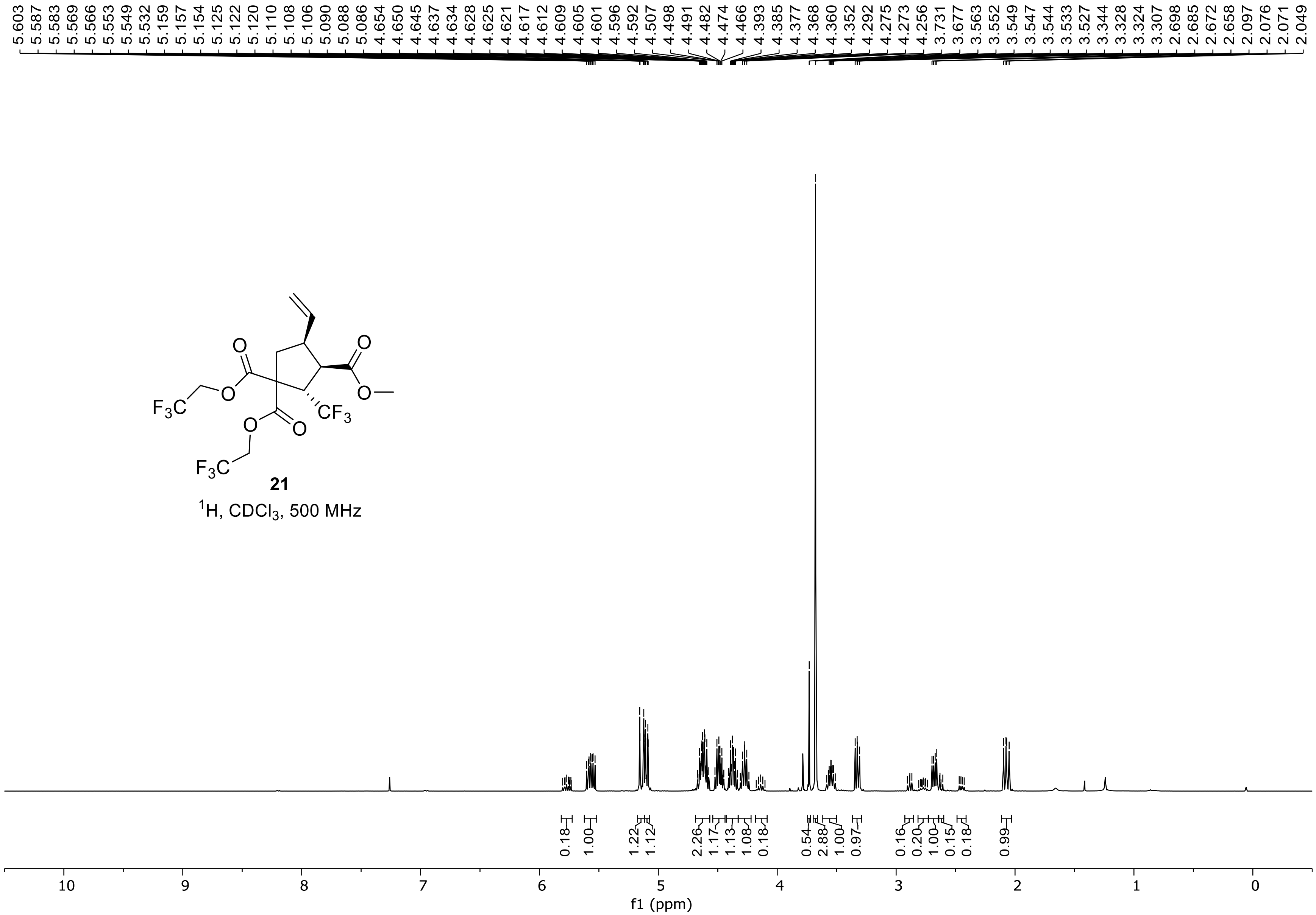

21

^1H, $CDCl_3$, 500 MHz

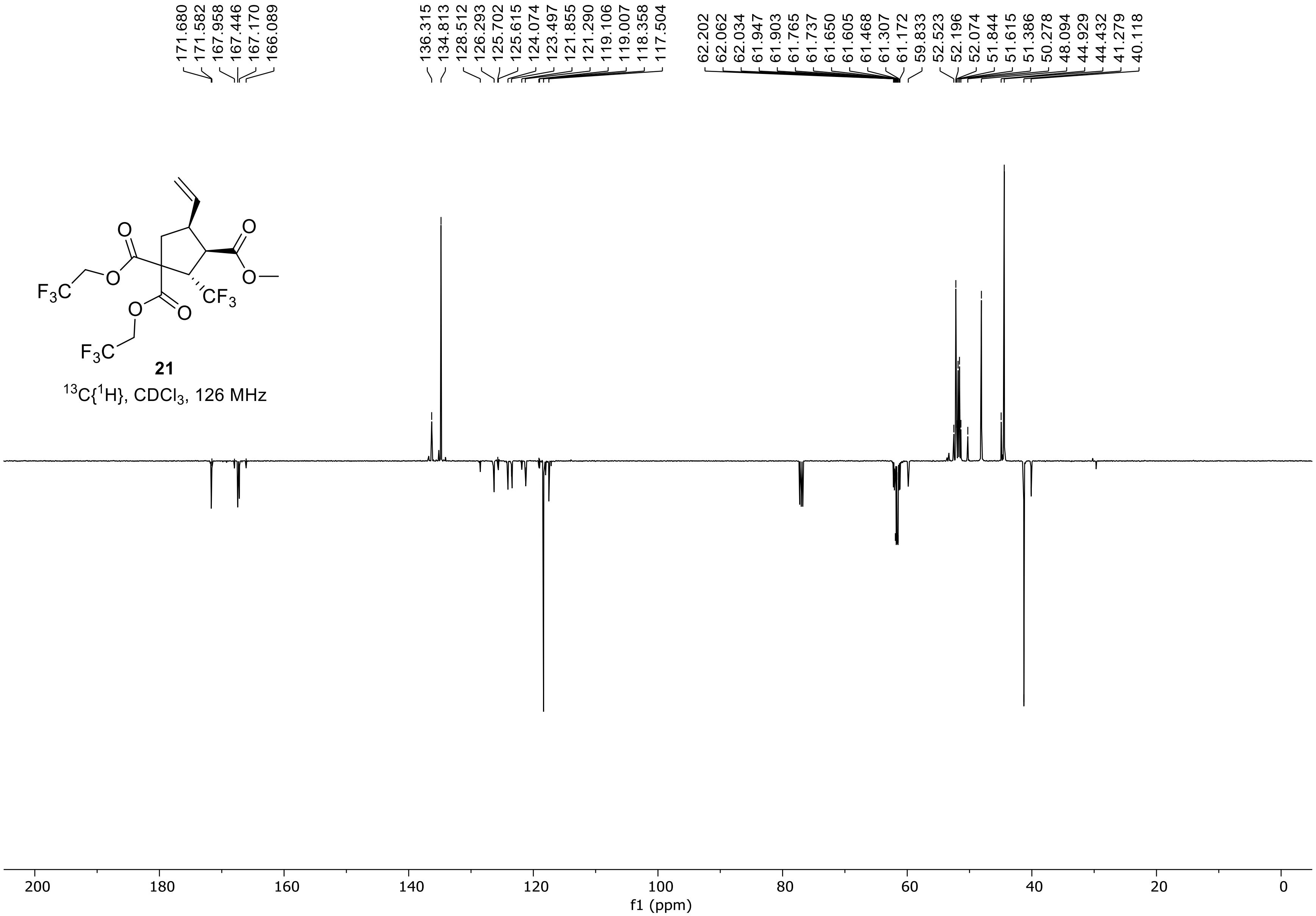

171.680
171.582
167.958
167.446
167.170
166.089
136.315
134.813
128.512
126.293
125.702
125.615
124.074
123.497
121.855
121.290
119.106
119.007
118.358
117.504
62.202
62.062
62.034
61.947
61.903
61.765
61.737
61.650
61.605
61.468
61.307
61.172
59.833
52.523
52.196
52.074
51.844
51.615
51.386
50.278
48.094
44.929
44.432
41.279
40.118
F3C
O
CF3
21
13C{1H}, CDCl3, 126 MHz
200
180
160
140
120
100
80
60
40
20
0
f1 (ppm)

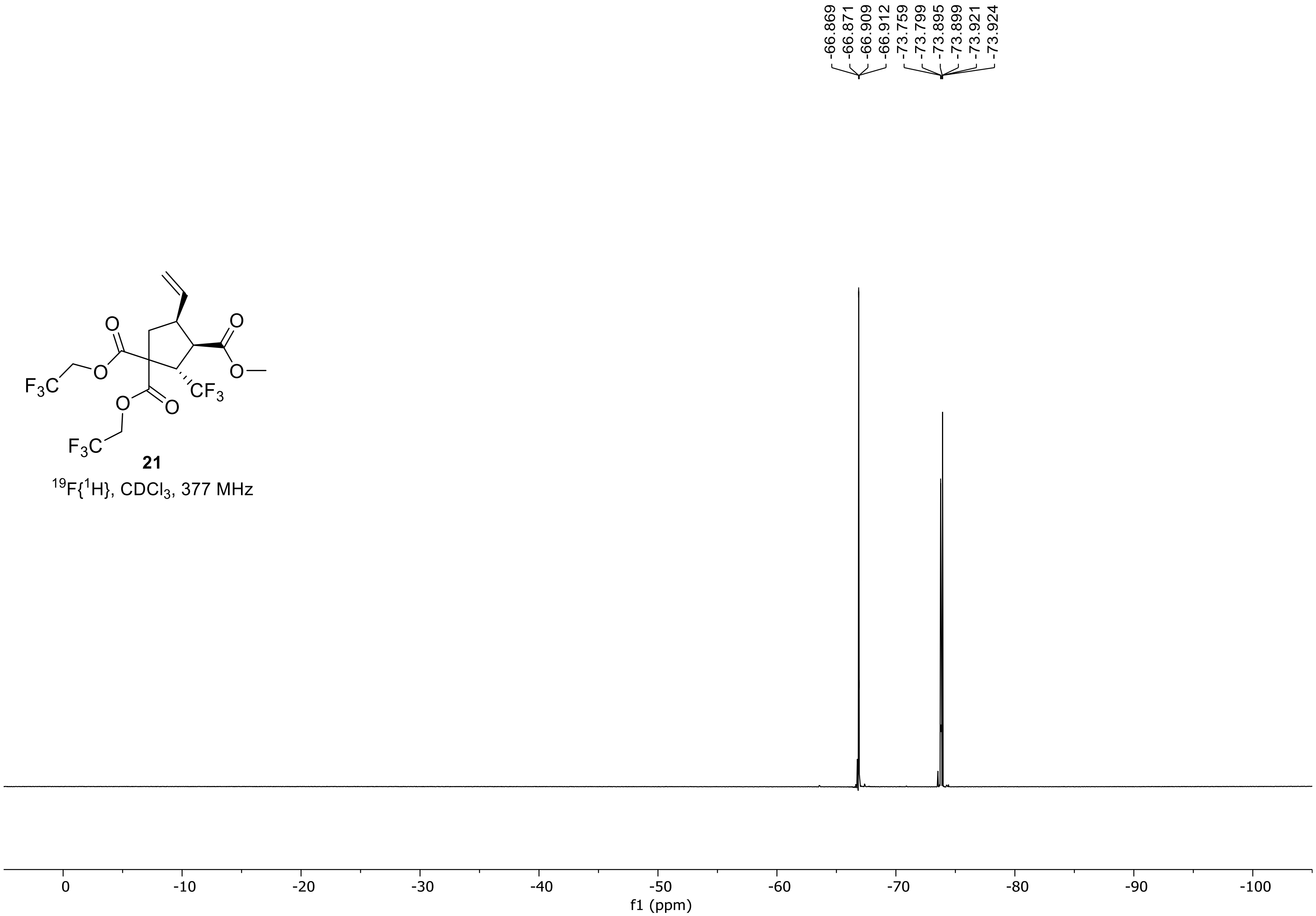
-66.869
-66.871
-66.909
-66.912
-73.759
-73.799
-73.895
-73.899
-73.921
-73.924
F3C
O
O
O
O
F3C
CF3
O
O
21
19F{1H}, CDCl3, 377 MHz
0
-10
-20
-30
-40
-50
-60
-70
-80
-90
-100
f1 (ppm)

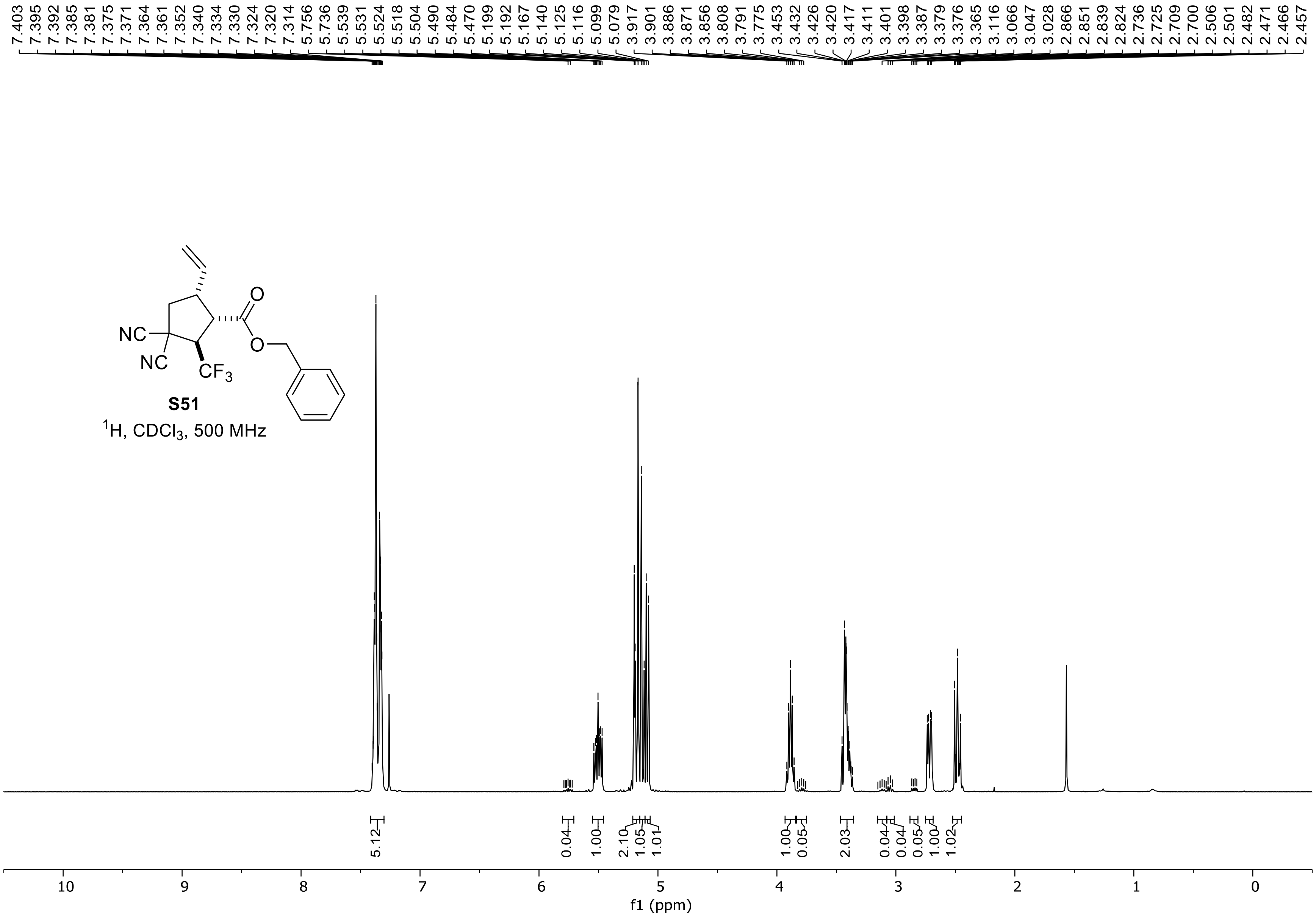
7.403 7.395 7.392 7.385 7.381 7.375 7.371 7.364 7.361 7.352 7.340 7.334 7.330 7.324 7.320 7.314 5.756 5.736 5.539 5.531 5.524 5.518 5.504 5.490 5.484 5.470 5.199 5.192 5.167 5.140 5.125 5.116 5.099 5.079 3.917 3.901 3.886 3.871 3.856 3.808 3.791 3.775 3.453 3.432 3.426 3.420 3.417 3.411 3.401 3.398 3.387 3.379 3.376 3.365 3.116 3.066 3.047 3.028 2.866 2.851 2.839 2.824 2.736 2.725 2.709 2.700 2.506 2.501 2.482 2.471 2.466 2.457
NC
NC
CF3
O
O
S51
1H, CDCl3, 500 MHz
5.12
0.04
1.00
2.10
1.05
1.01
1.00
0.05
2.03
0.04
0.04
0.05
1.00
1.02
10 9 8 7 6 5 4 3 2 1 0
f1 (ppm)

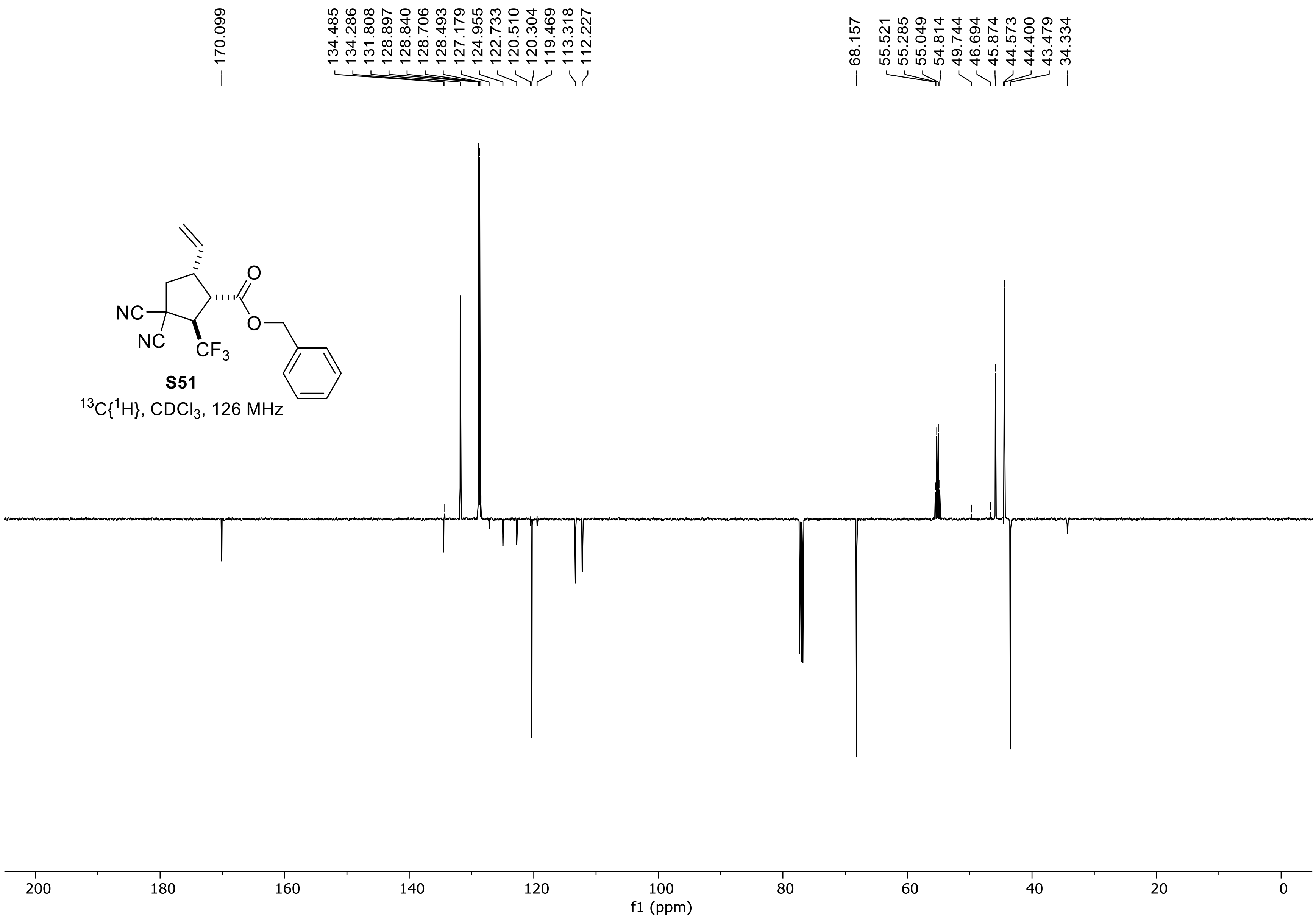
170.099
134.485
134.286
131.808
128.897
128.840
128.706
128.493
127.179
124.955
122.733
120.510
120.304
119.469
113.318
112.227
68.157
55.521
55.285
55.049
54.814
49.744
46.694
45.874
44.573
44.400
43.479
34.334
NC
NC
CF3
O
O
S51
13C{1H}, CDCl3, 126 MHz
200
180
160
140
120
100
80
60
40
20
0
f1 (ppm)

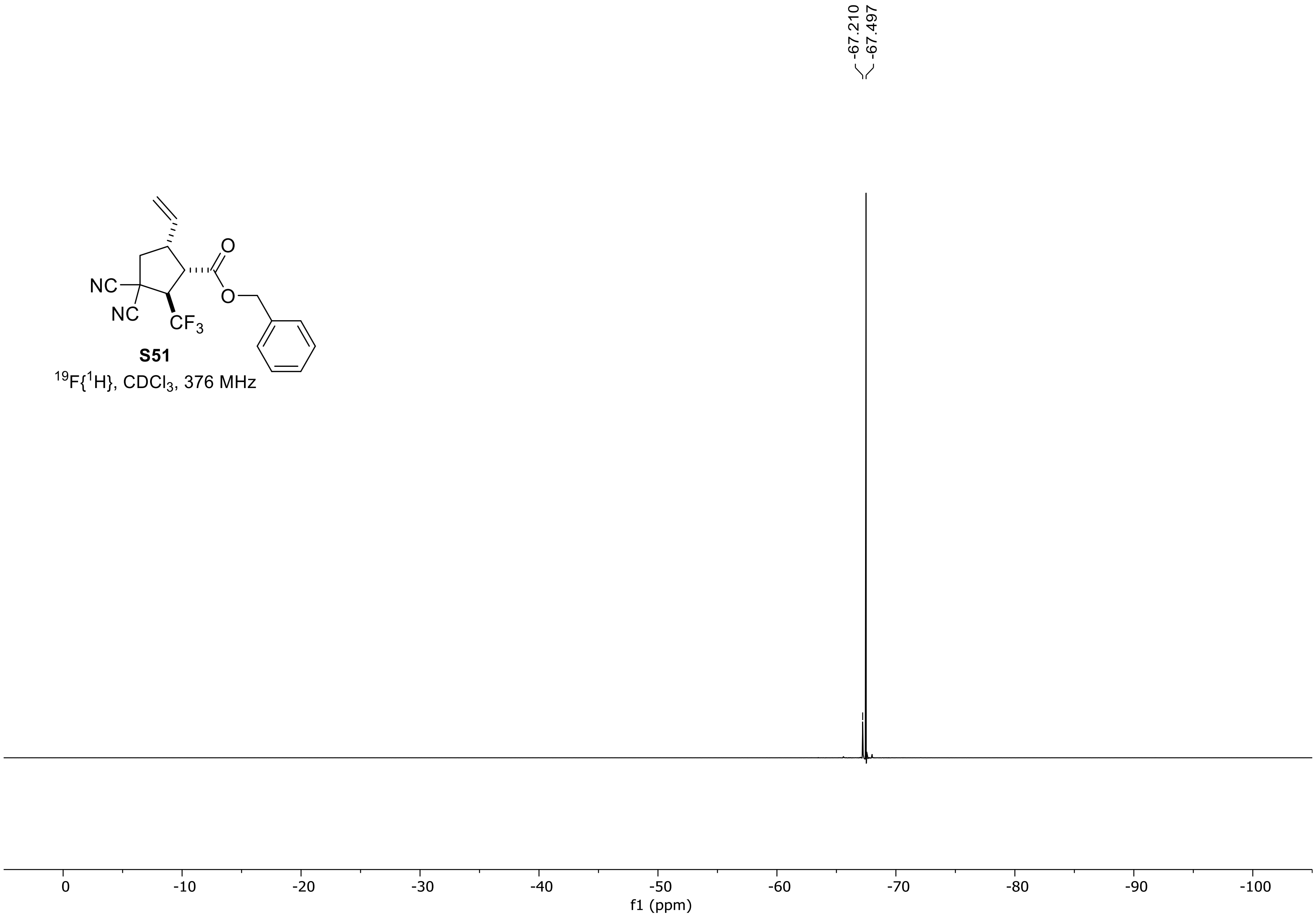

S51

$^{19}F\{^{1}H\}$, $CDCl_3$, 376 MHz

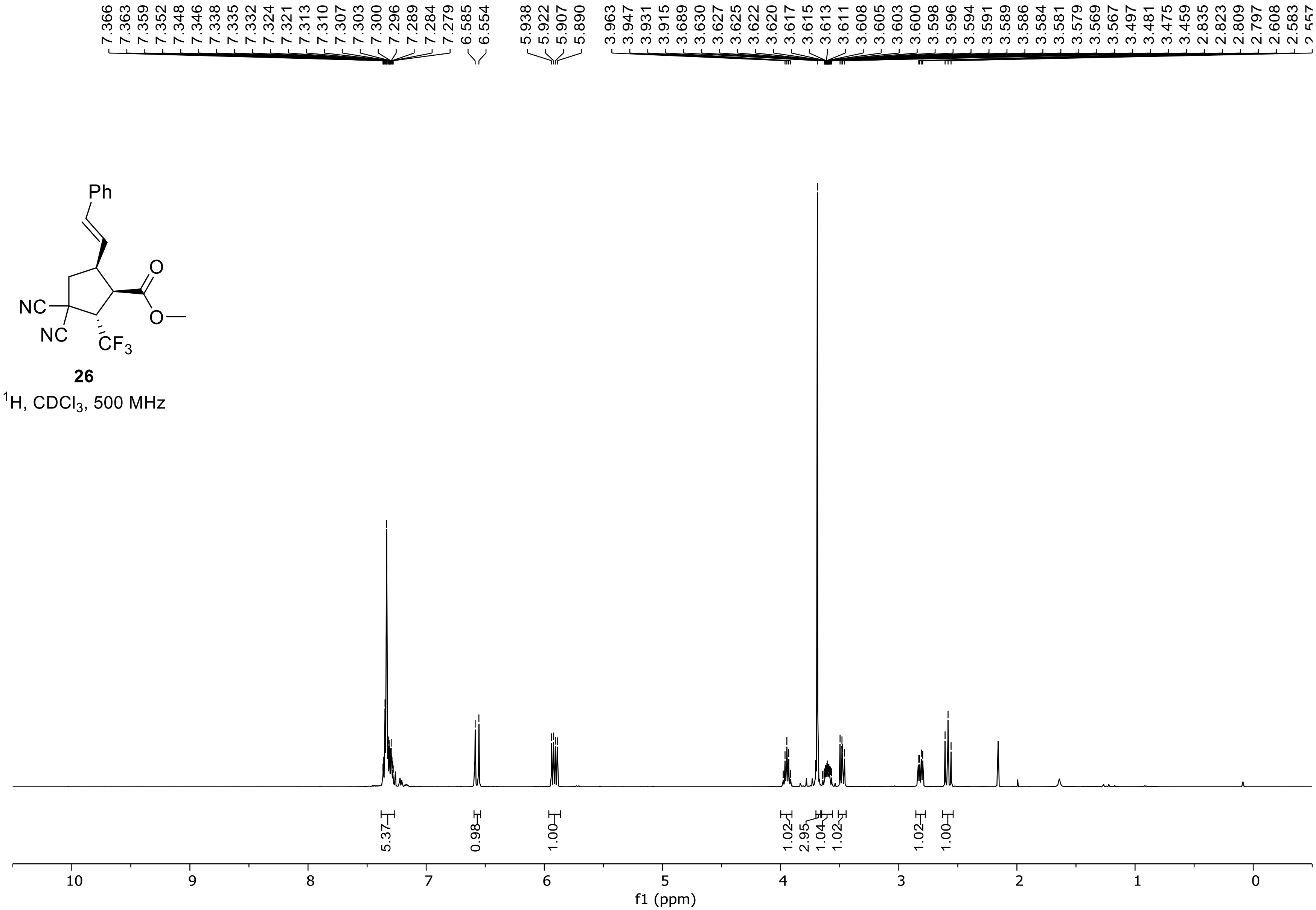

26

^{1}H, $CDCl_3$, 500 MHz

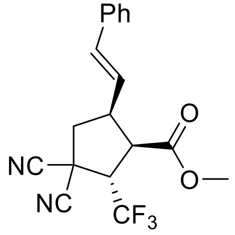

26

$^{13}C\{^{1}H\}$, $CDCl_3$, 126 MHz

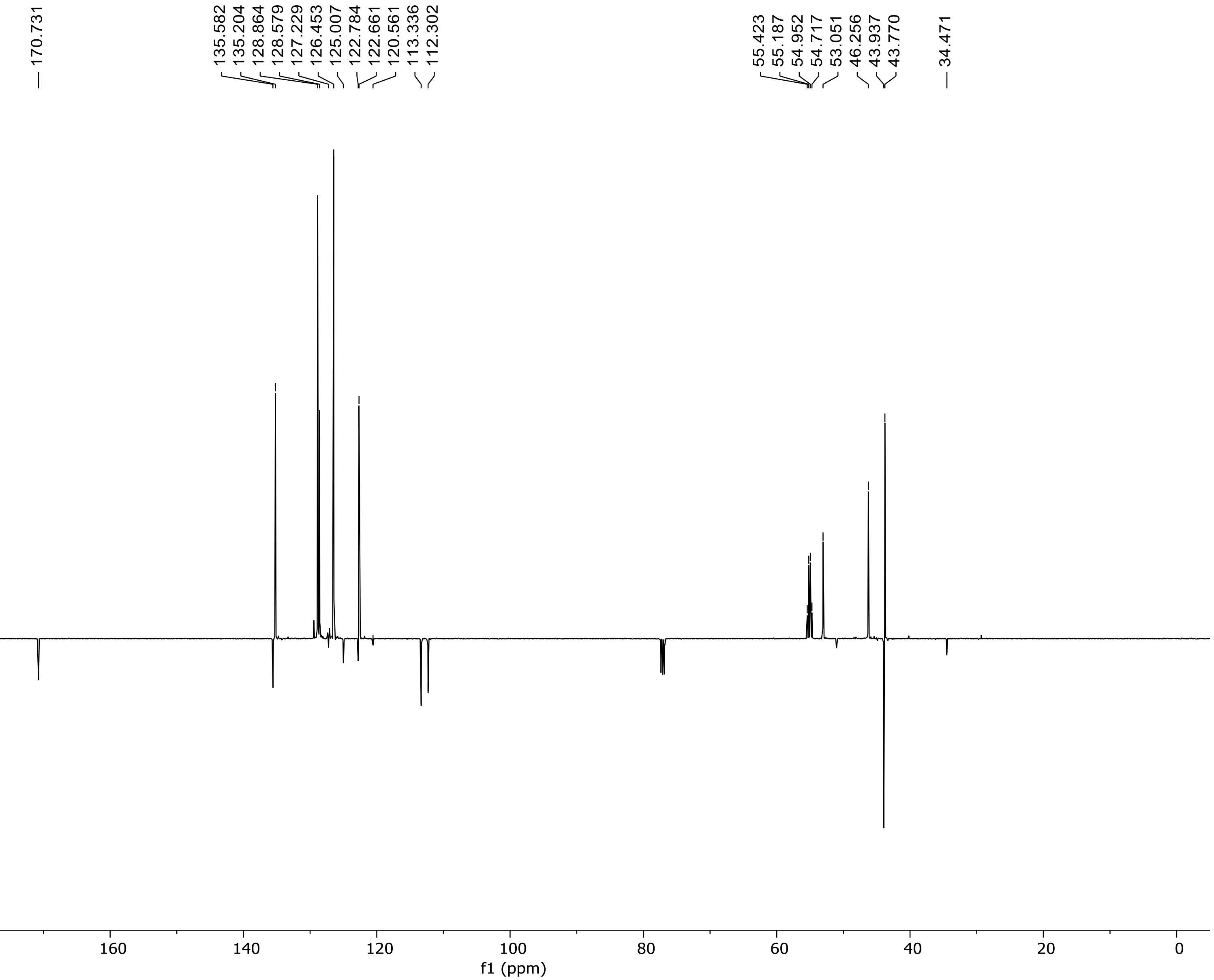

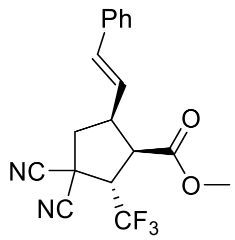

26

$^{19}F\{^{1}H\}$, $CDCl_3$, 376 MHz

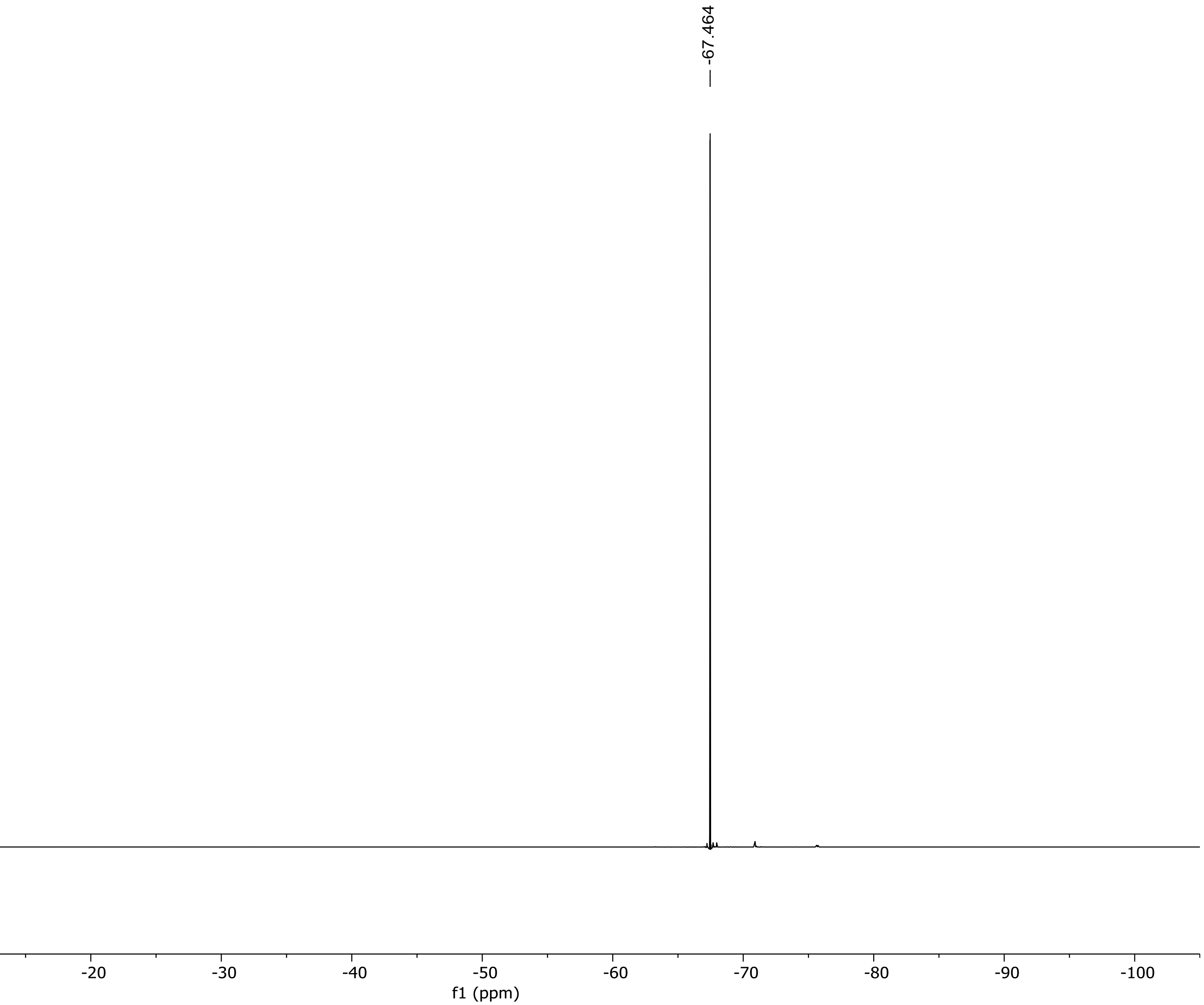

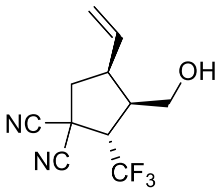

S52

^{1}H, $CDCl_3$, 500 MHz

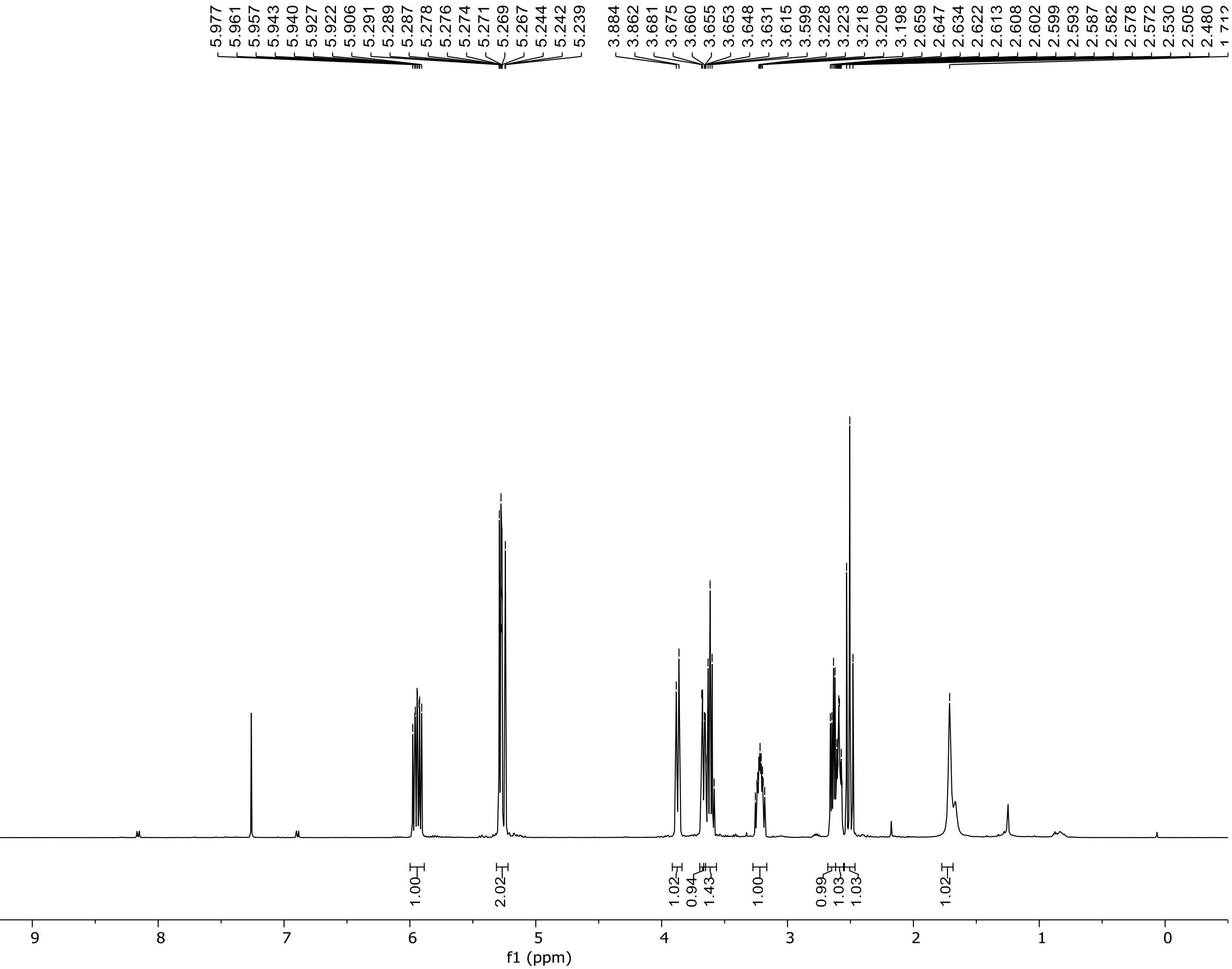

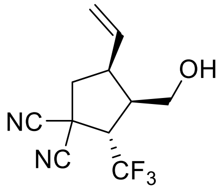

S52

$^{13}C\{^{1}H\}$, $CDCl_3$, 126 MHz

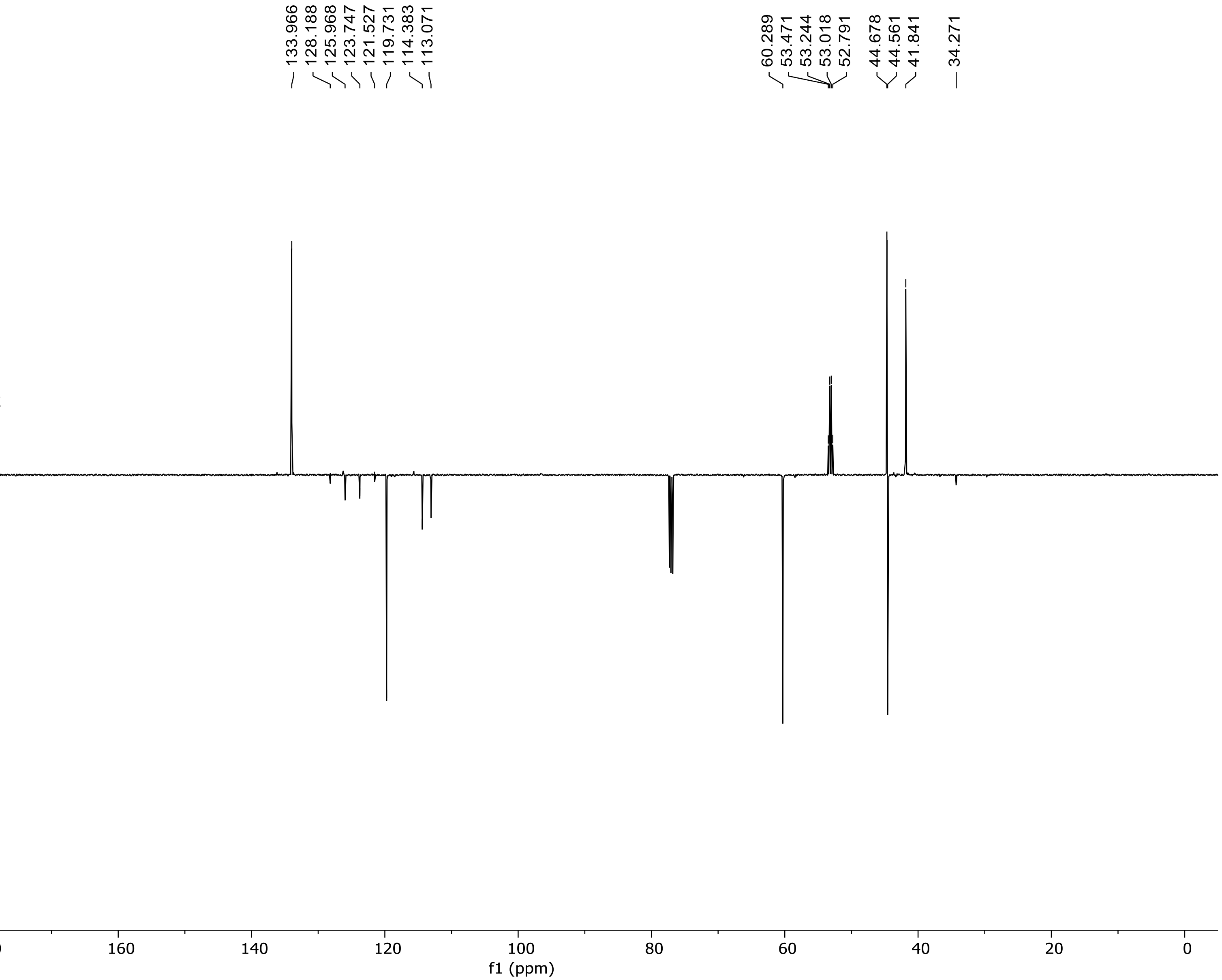

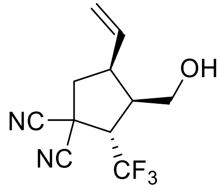

S52

$^{19}F\{^{1}H\}$, $CDCl_3$, 376 MHz

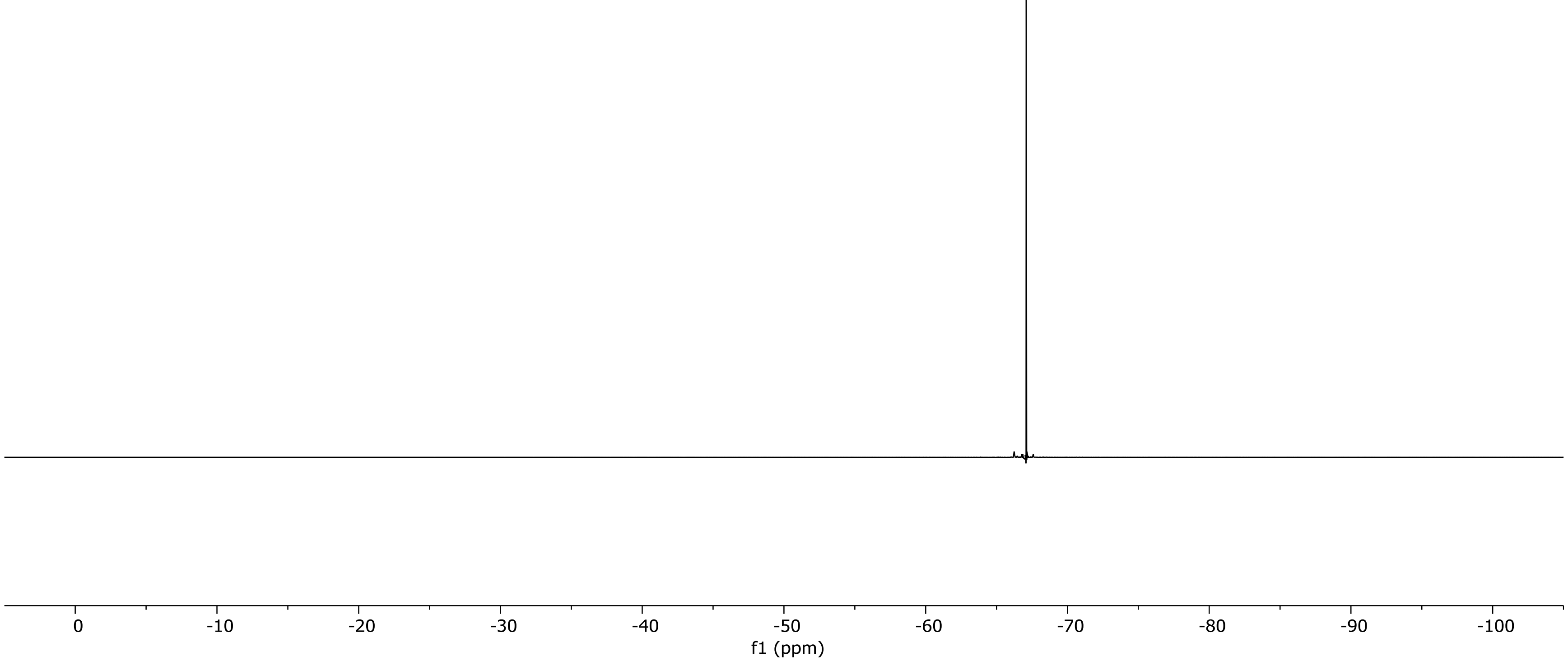

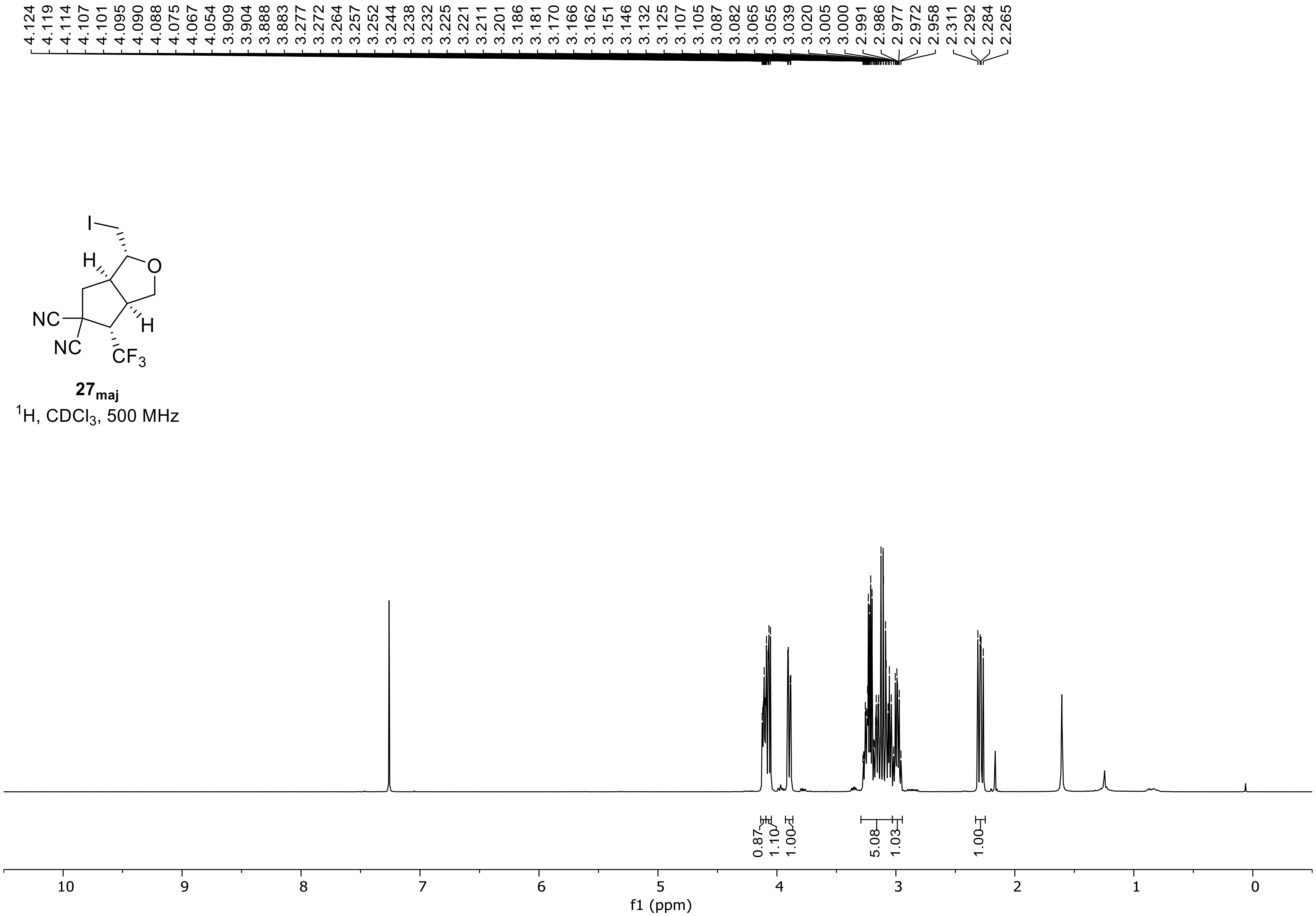

27$_{maj}$

^1H, $CDCl_3$, 500 MHz

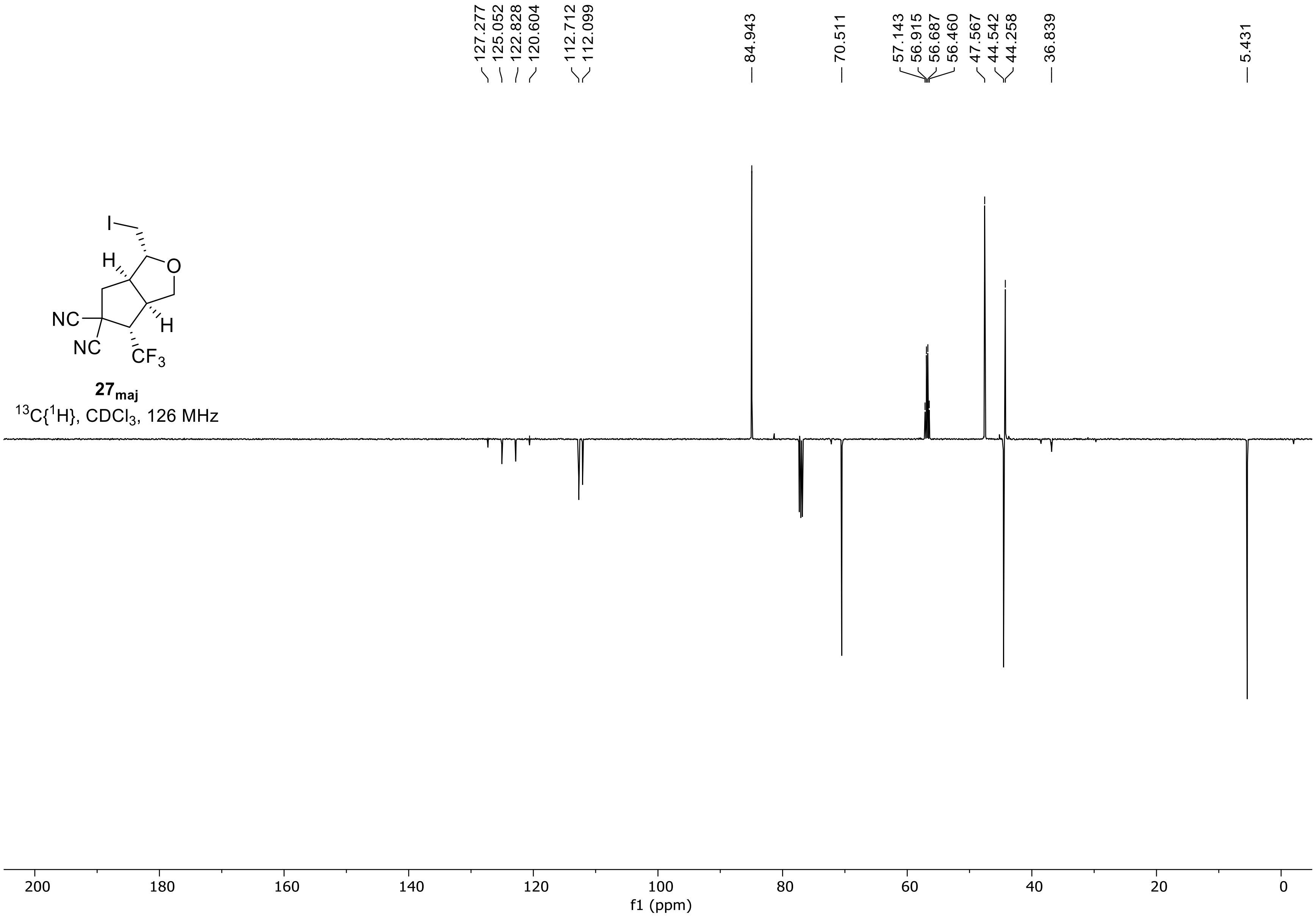

27$_{maj}$

$^{13}C\{^{1}H\}$, $CDCl_3$, 126 MHz

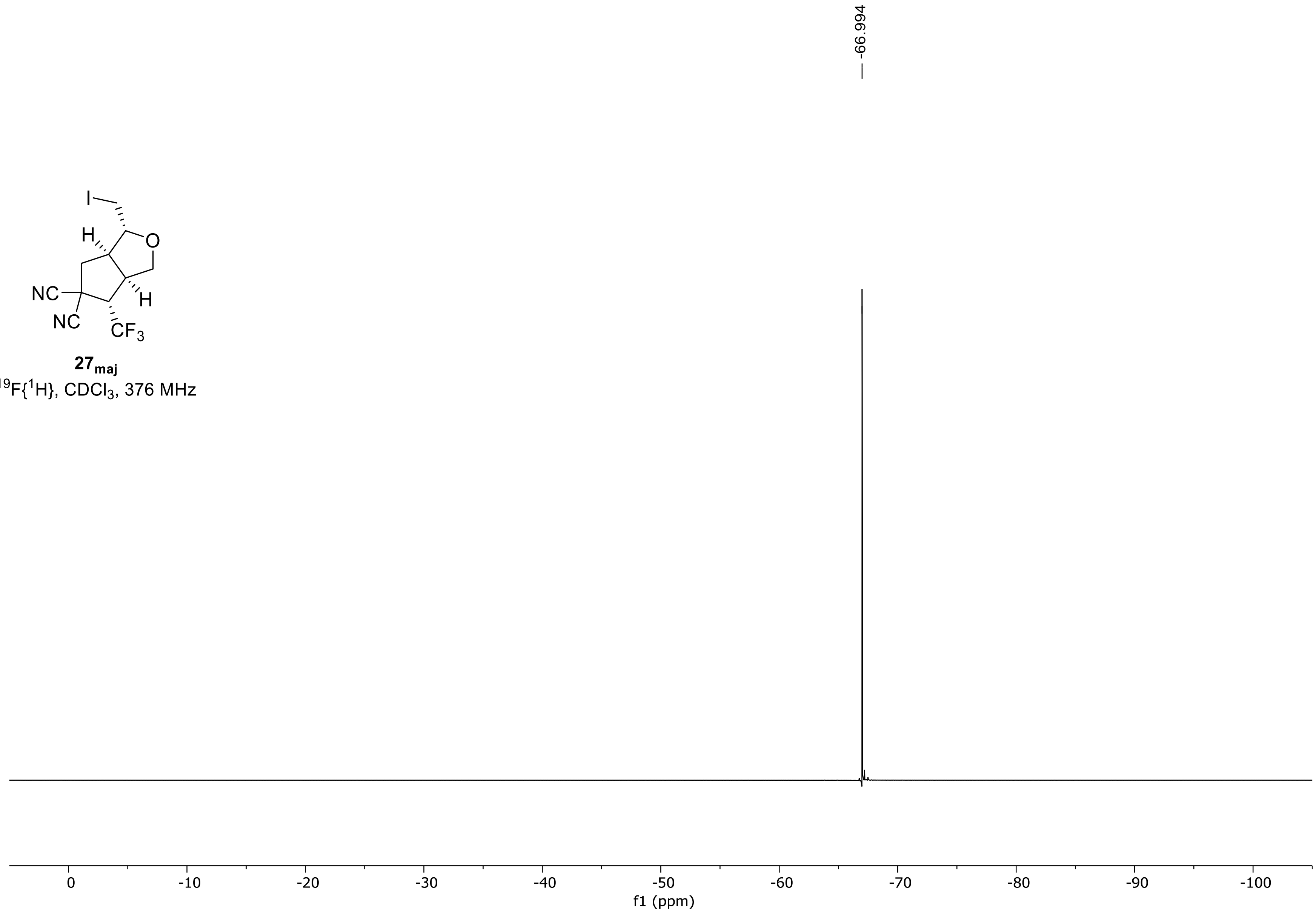

27_{maj}
$^{19}F\{^{1}H\}$, $CDCl_3$, 376 MHz

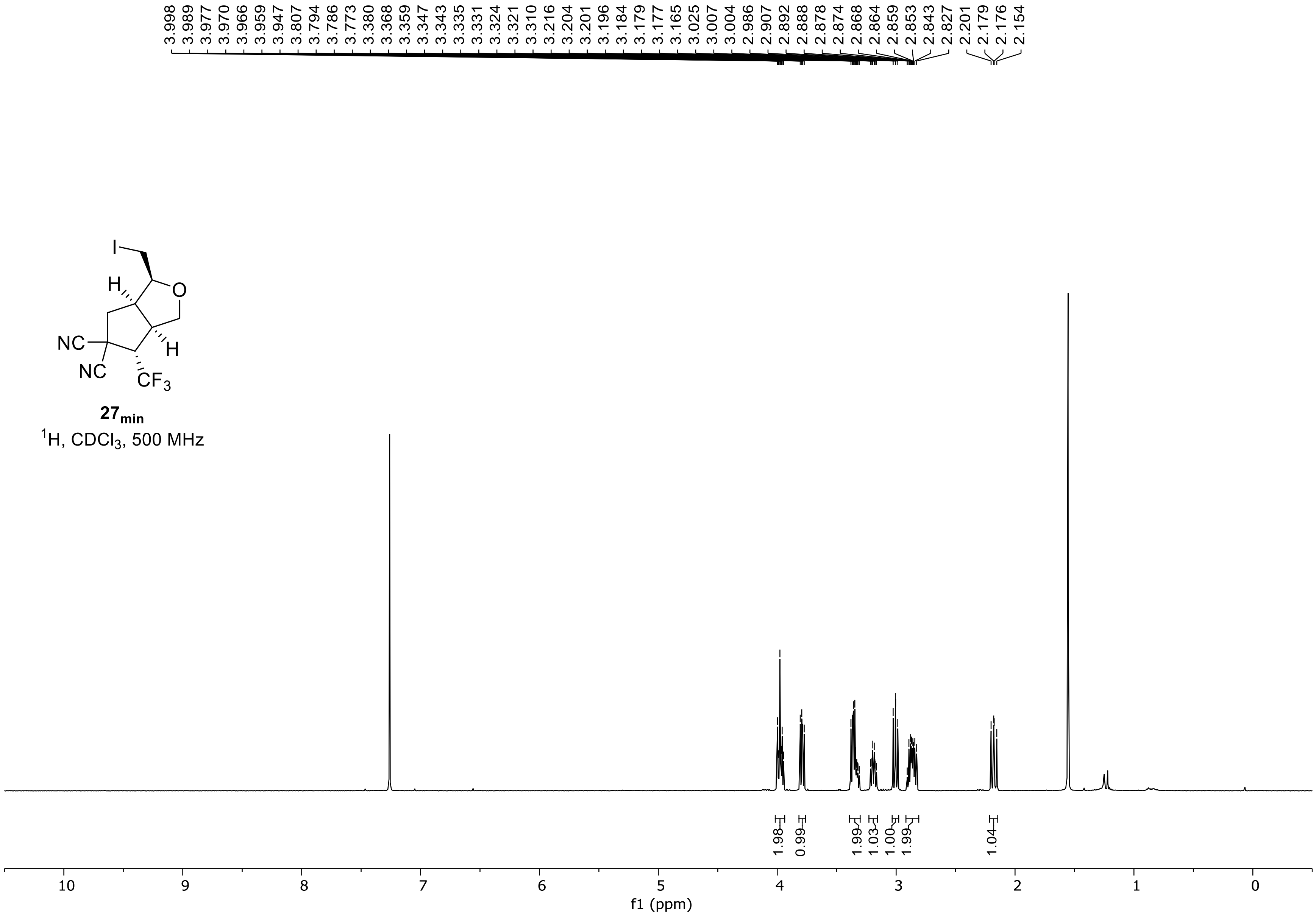

27$_{min}$

^{1}H, $CDCl_3$, 500 MHz

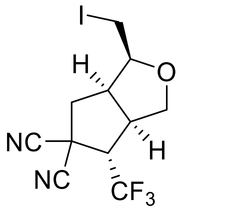

27$_{min}$

$^{13}C\{^{1}H\}$, $CDCl_3$, 126 MHz

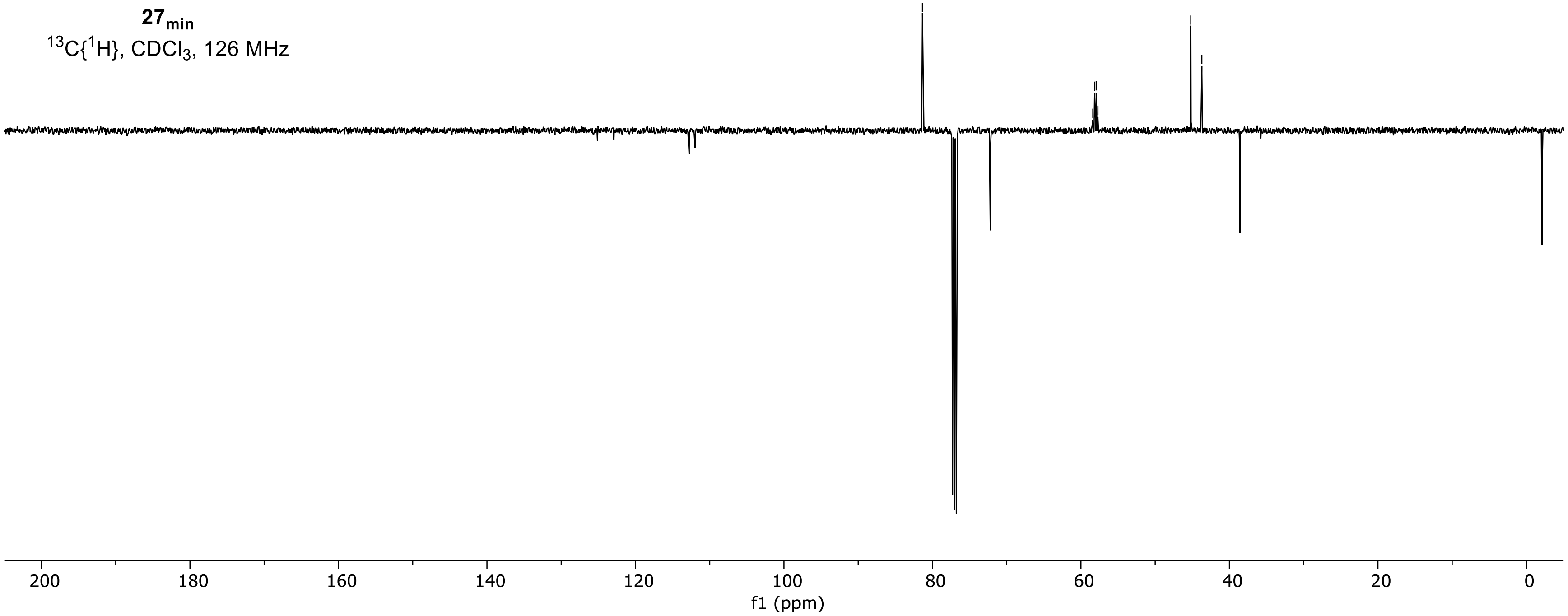

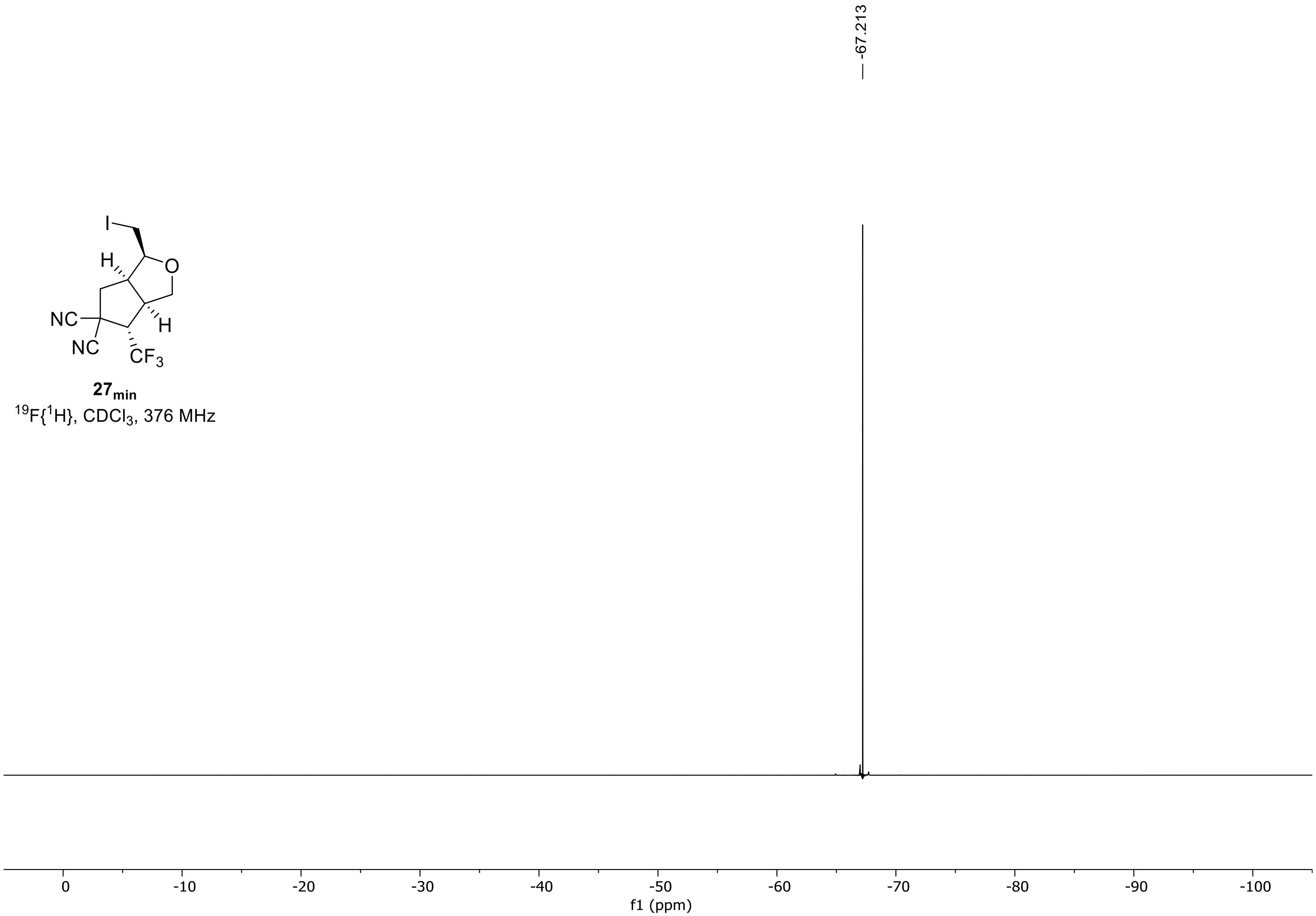

27_{min}

$^{19}F\{^{1}H\}$, $CDCl_3$, 376 MHz

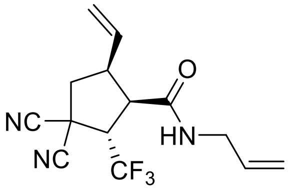

S53

^{1}H, $CDCl_3$, 500 MHz

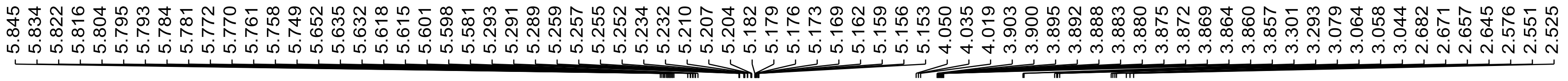

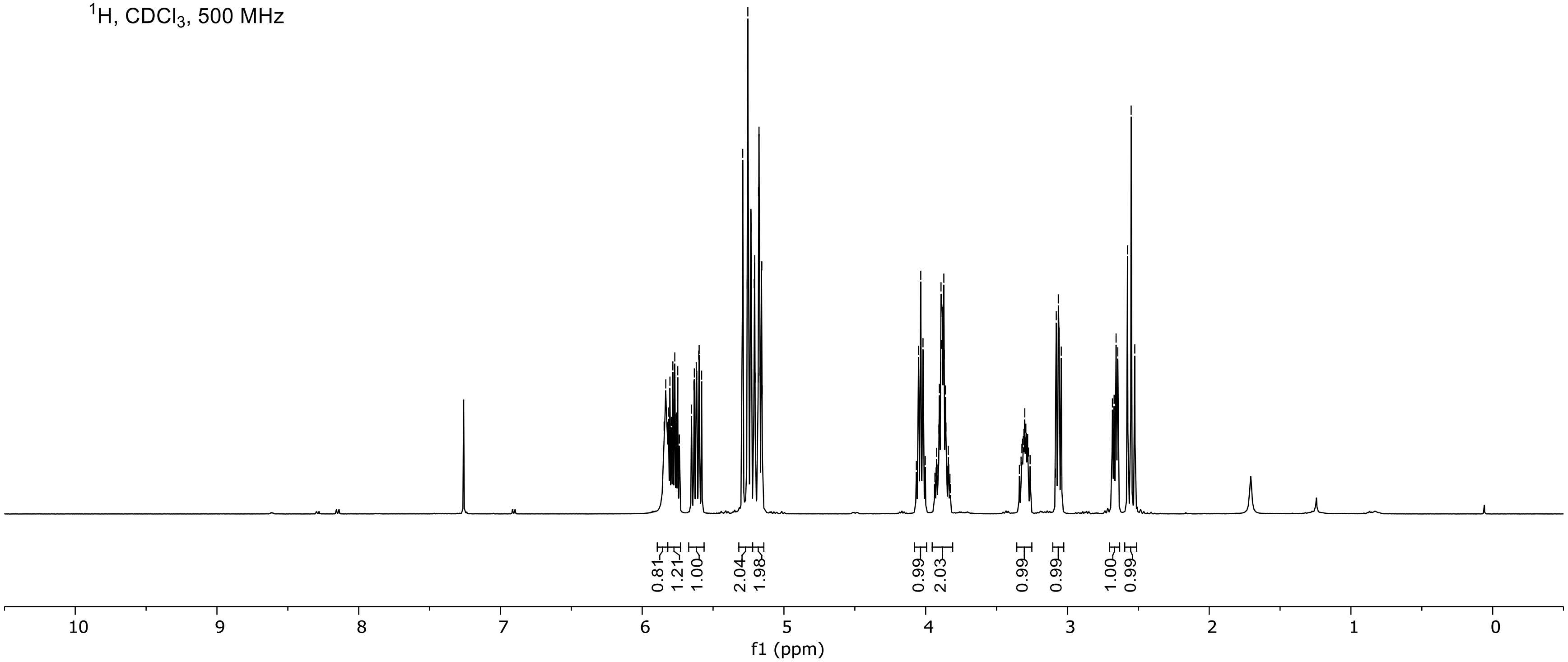

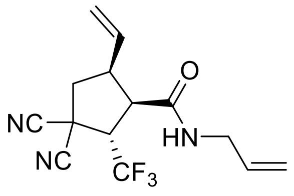

S53

$^{13}C\{^{1}H\}$, $CDCl_3$, 126 MHz

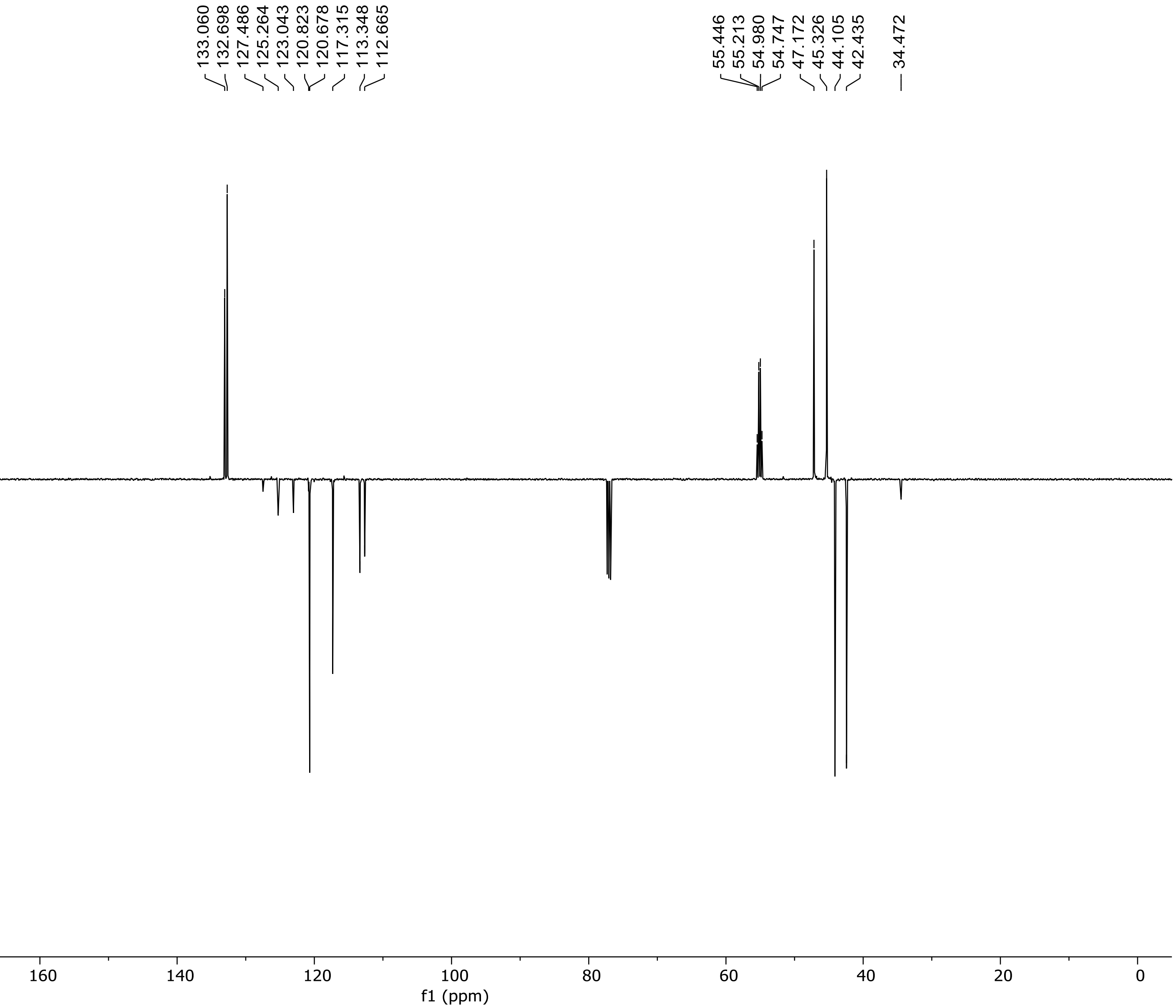

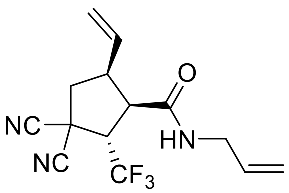

S53

$^{19}F\{^{1}H\}$, $CDCl_3$, 376 MHz

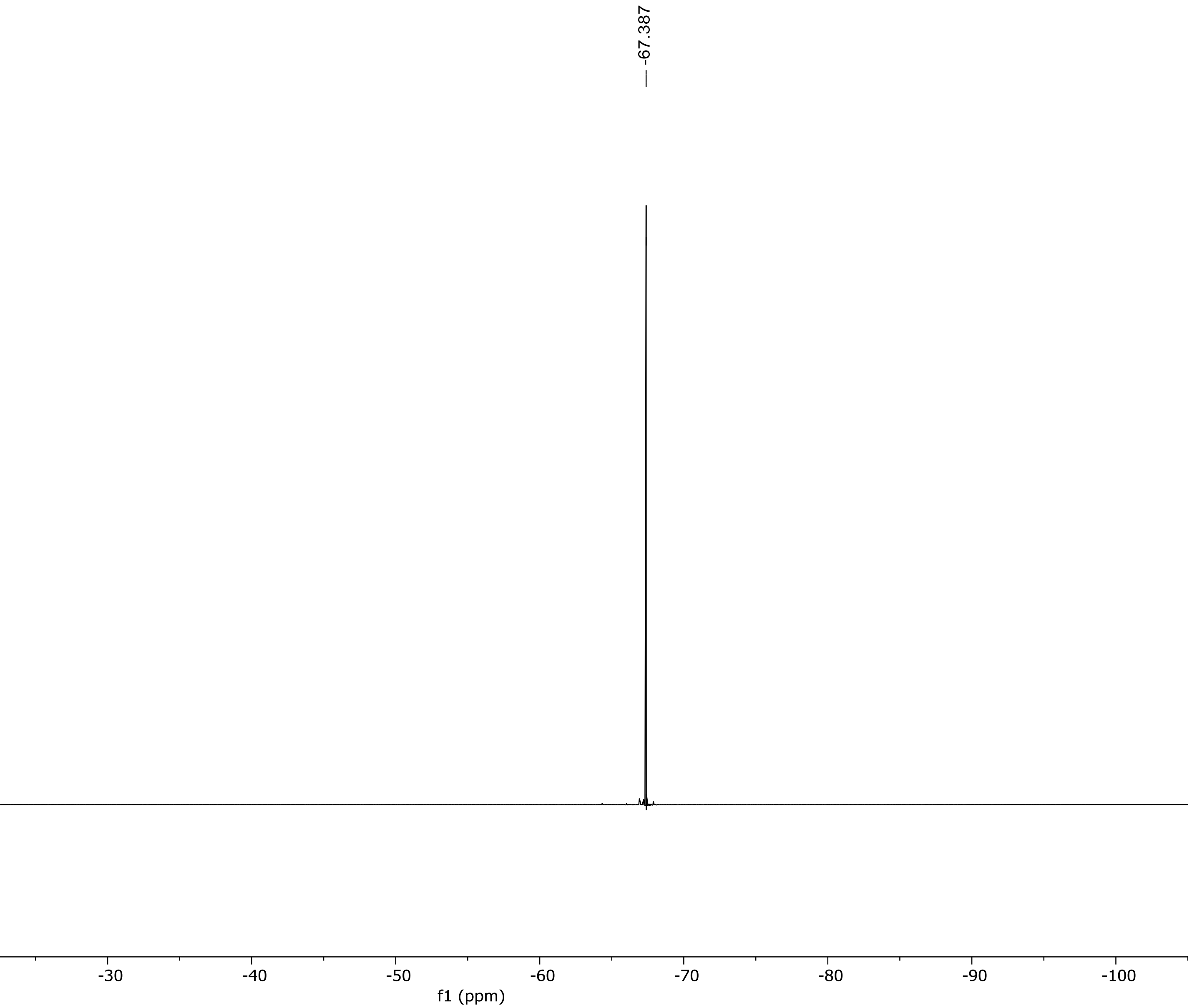

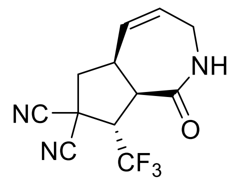

28

^{1}H, $CDCl_3$, 500 MHz

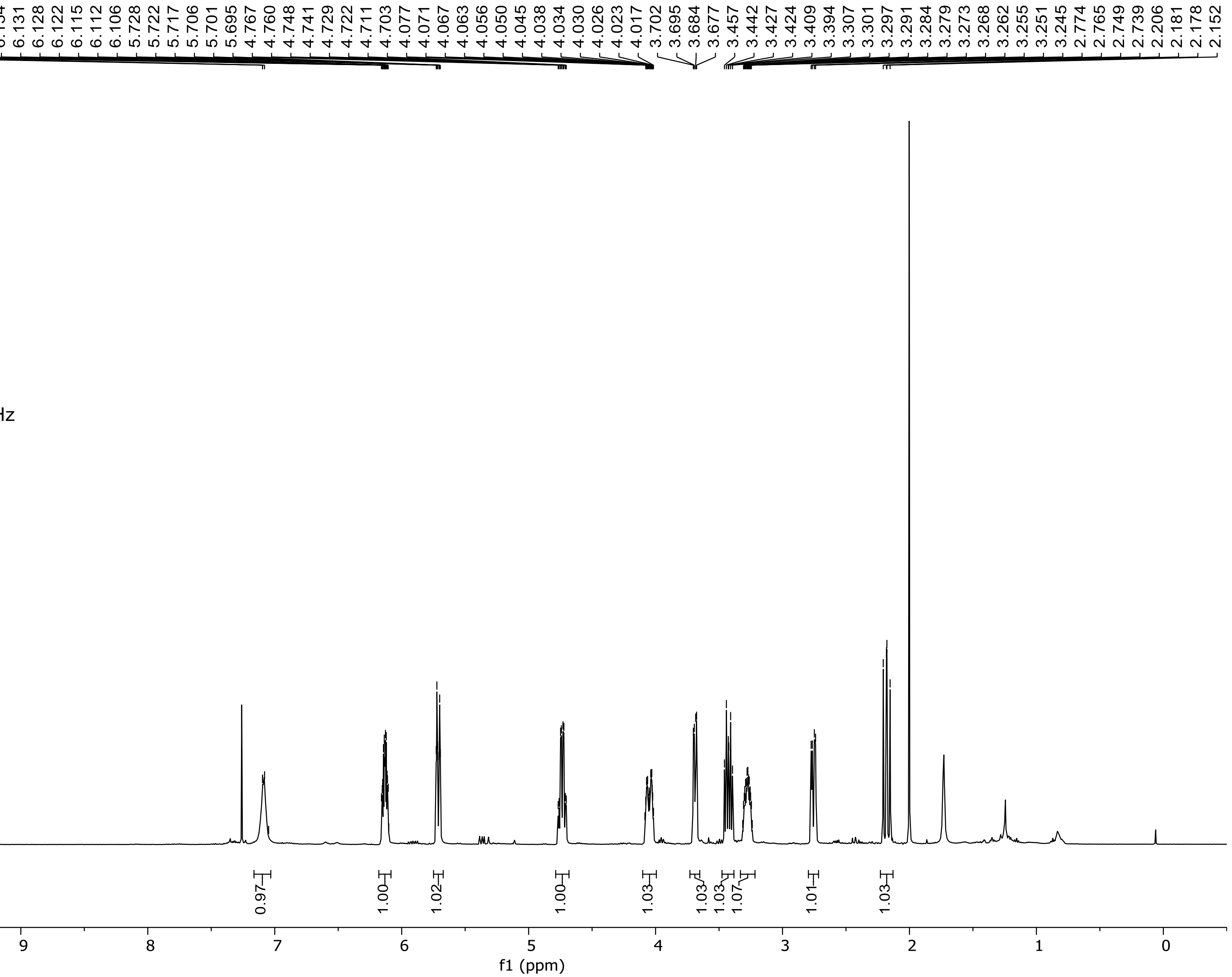

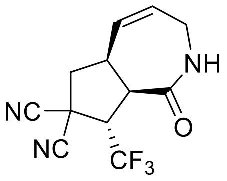

28

$^{13}C\{^{1}H\}$, $CDCl_3$, 126 MHz

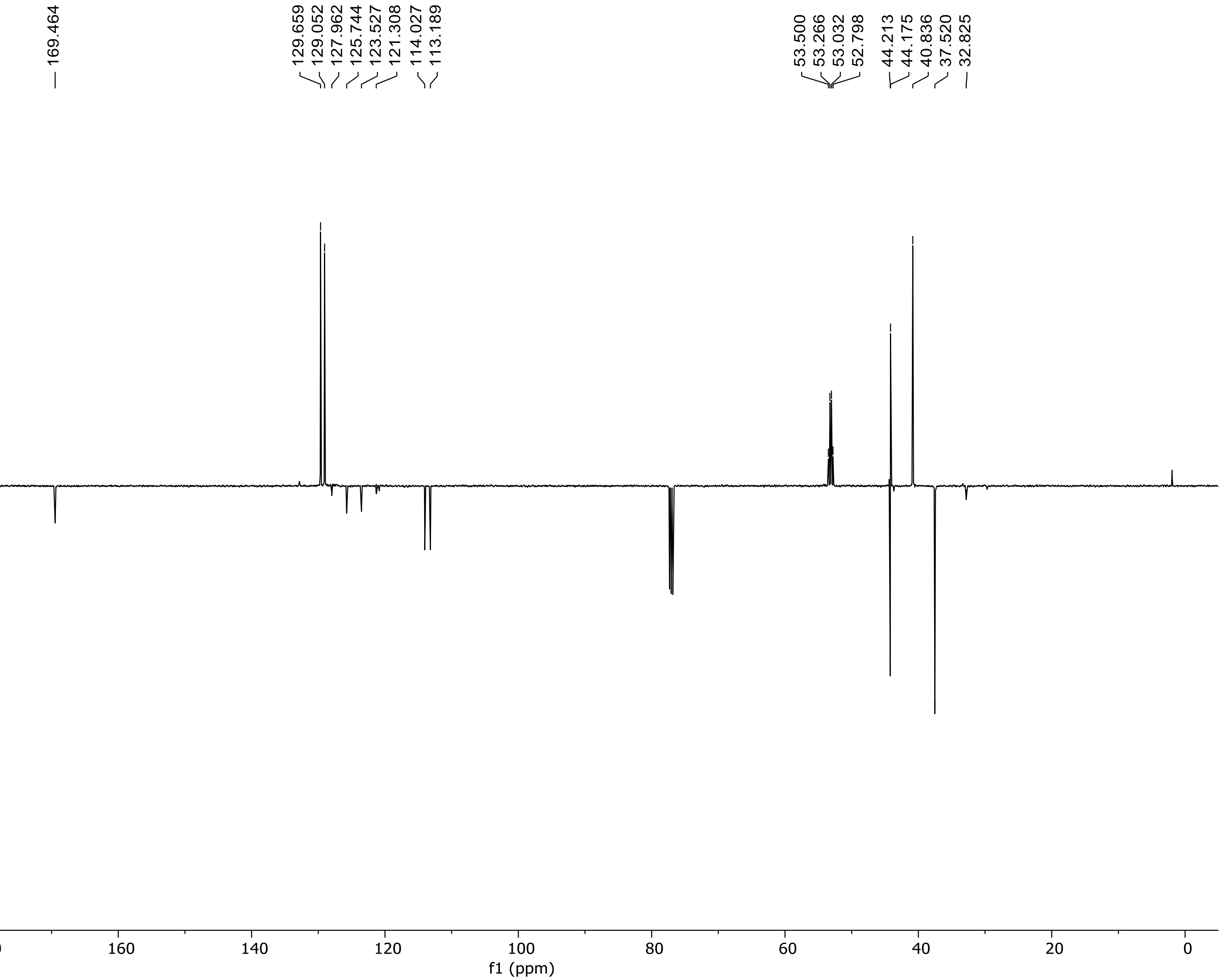

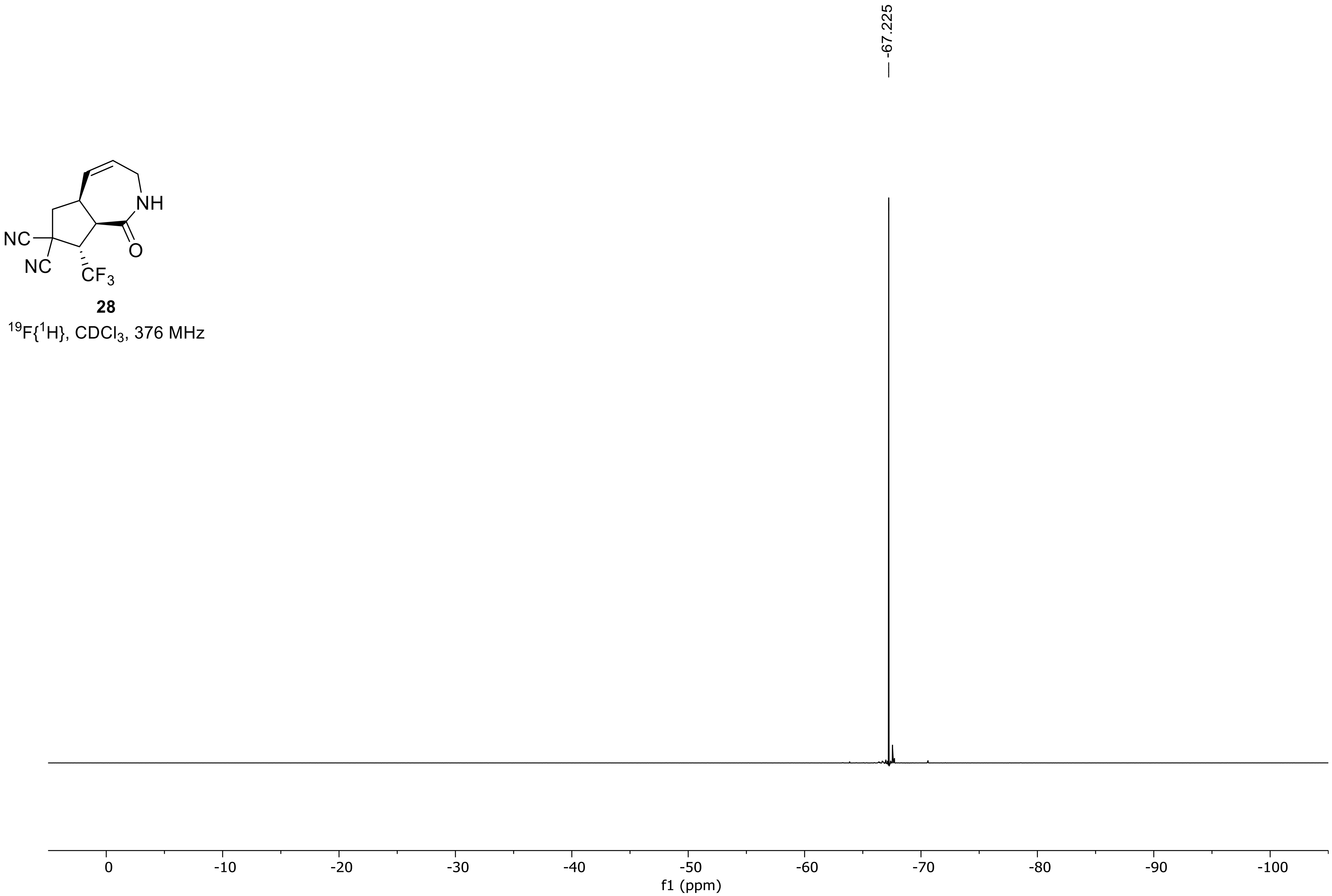

28

$^{19}F\{^1H\}$, $CDCl_3$, 376 MHz

Appendix II: HPLC and GC traces of novel compounds

HPLC Data for **4**: Chiralcel OD-H (hexane : *i*-PrOH 97:3, flow rate 1.0 mlmin^{-1}, 254 nm, 40 °C) *major diastereoisomer*: t_R (1*S*,2*R*,5*R*): 30.1 min, t_R (1*R*,2*S*,5*S*): 32.3 min, 7:93 er. *minor diastereoisomer*: t_R (1*S*,2*R*,5*S*): 40.4 min, t_R (1*R*,2*S*,5*R*): 45.2 min, <5:95 er.

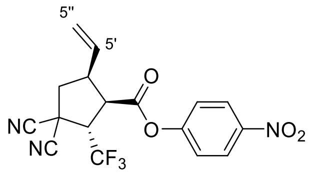

4

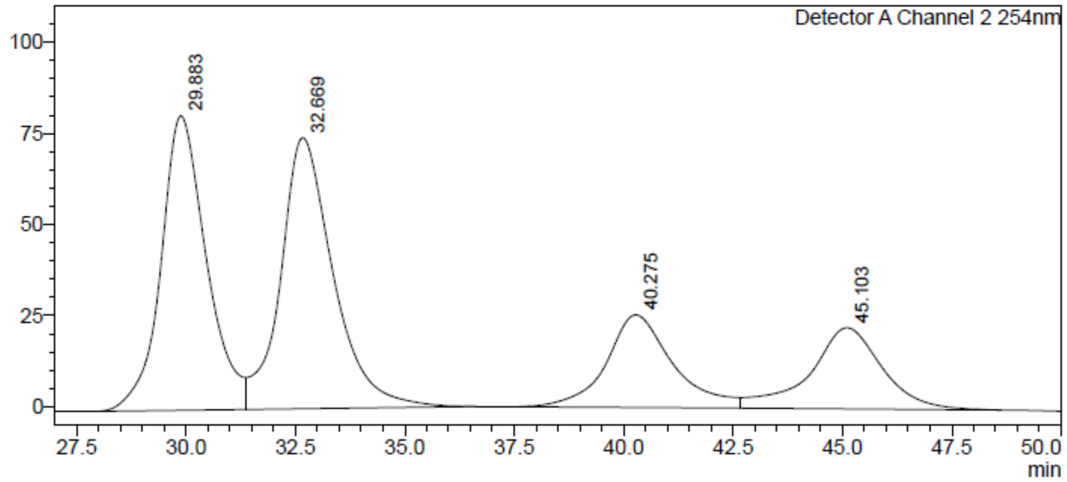

Detector A Channel 2 254nm

Peak#	Ret. Time	Area%
1	29.883	33.698
2	32.669	35.969
3	40.275	15.536
4	45.103	14.798
Total		100.000

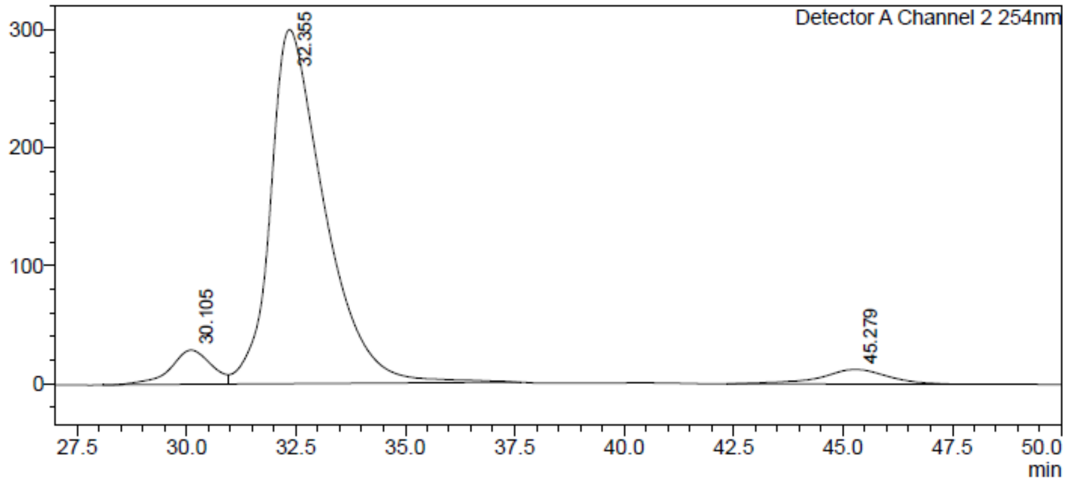

Detector A Channel 2 254nm

Peak#	Ret. Time	Area%
1	30.105	6.491
2	32.355	88.957
3	45.279	4.551
Total		100.000

GC Data for **8**: Restek Rt-βDEXcst (length: 30 m, thickness: 0.25 mm, film thickness: 0.25 μm, carrier gas: He, linear velocity: 28 cmsec^{-1}, temperature: 120 °C (60 min), 120 to 140 °C (20 min)) *major diastereoisomer*: t_R (1*R*,2*S*,5*S*): 64.5 min, t_R (1*S*,2*R*,5*R*): 65.5 min, 93:7 er. *minor diastereoisomer*: $t_{R,1}$: 69.8 min, $t_{R,2}$: 72.7 min, >99:1 er.

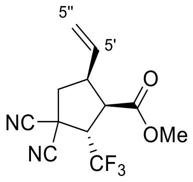

8

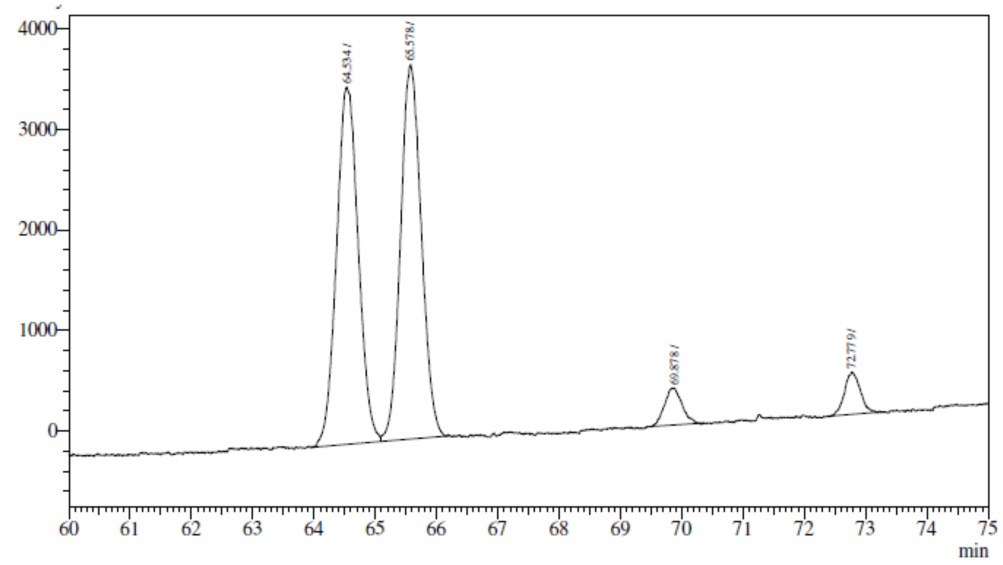

Peak#	Ret.Time	Area	Height	Conc.	Unit	Mark	ID#	Cmpd Name
1	64.534	87915	3558	46.141				
2	65.578	87883	3726	46.124		V		
3	69.878	7272	367	3.817				
4	72.779	7467	420	3.919				
**Total**		190537	8071					

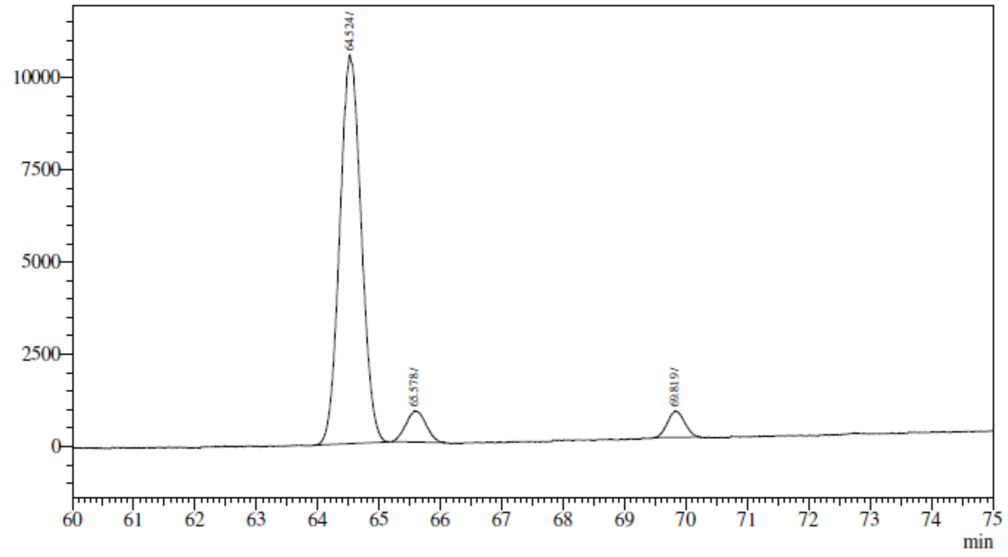

Peak#	Ret.Time	Area	Height	Conc.	Unit	Mark	ID#	Cmpd Name
1	64.524	256193	10551	88.576				
2	65.578	19181	848	6.632				
3	69.819	13861	719	4.792				
**Total**		289235	12118					

GC Data for **9**: Restek Rt-βDEXcst (length: 30 m, thickness: 0.25 mm, film thickness: 0.25 μm), carrier gas: He, linear velocity: 28 cmsec^{-1}, temperature: 60 to 140 °C (27 min), 140 °C (20 min)) t_R (1*S*,2*R*,5*R*): 37.8 min, t_R (1*R*,2*S*,5*S*): 38.2 min, 95:5 er.

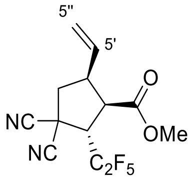

9

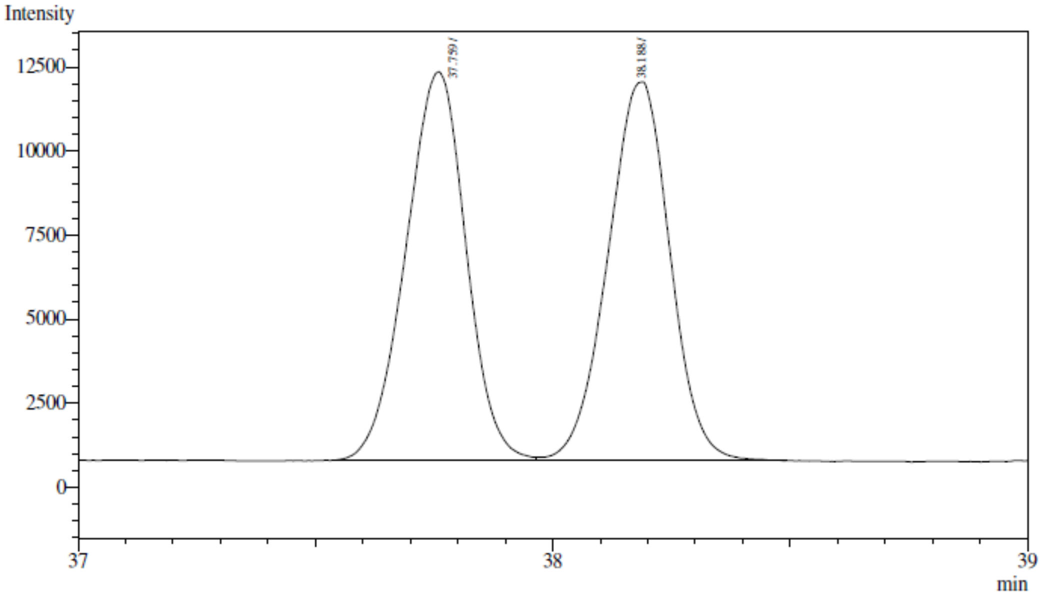

Peak#	Ret.Time	Area	Height	Conc.	Unit	Mark	ID#	Cmpd Name
1	37.759	102099	11552	49.922				
2	38.188	102417	11256	50.078		V		
**Total**		204516	22808					

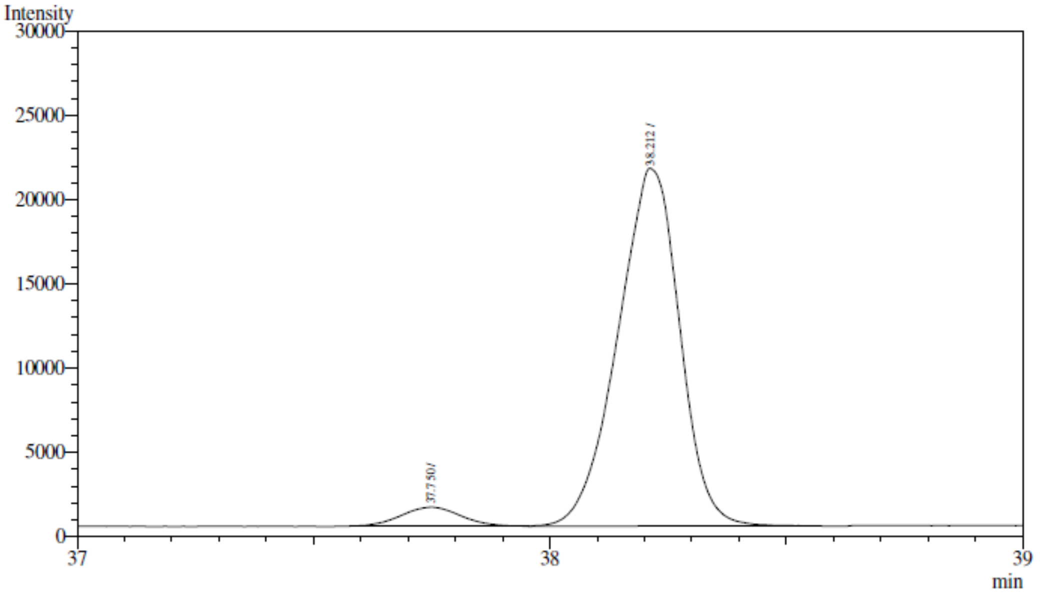

Peak#	Ret.Time	Area	Height	Conc.	Unit	Mark	ID#	Cmpd Name
1	37.750	9729	1118	4.794				
2	38.212	193238	21206	95.206				
**Total**		202967	22324					

GC data for **10:** Restek Rt-βDEXcst (length: 30 m, thickness: 0.25 mm, film thickness: 0.25 μm, carrier gas: He, linear velocity: 28 $cmsec^{-1}$, temperature: 90 to 135 °C (45 min), 135 °C (40 min), 135 to 170 °C (35 min)) *major diastereoisomer*: t_R (1*R*,2*S*,5*S*): 97.2 min, t_R (1*S*,2*R*,5*R*): 98.1 min, 90:10 er. *minor diastereoisomer*: t_R (1*R*,2*S*,5*R*): 100.1 min, t_R (1*S*,2*R*,5*S*): 102.9 min, 88:12 er.

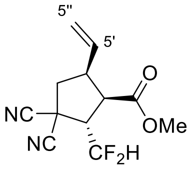

10

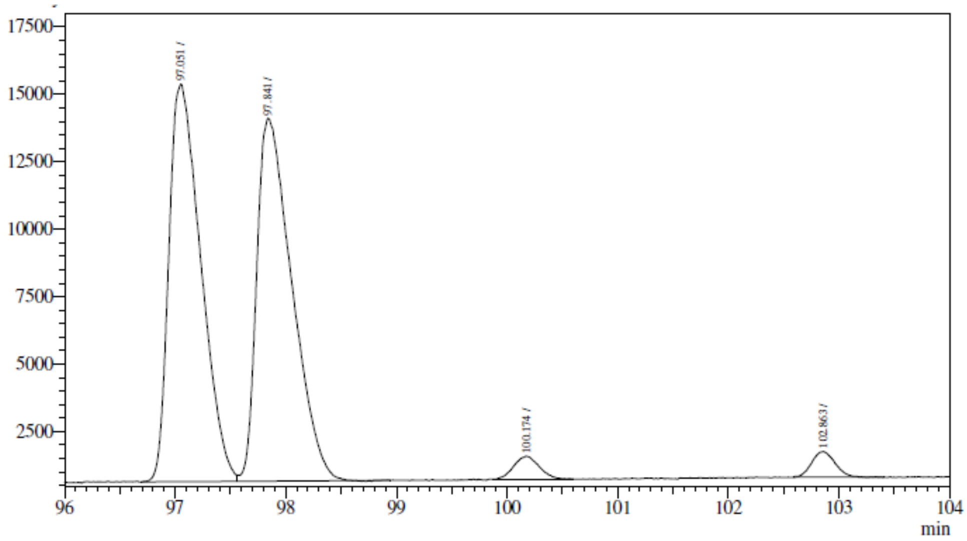

Peak#	Ret.Time	Area	Height	Conc.	Unit	Mark	ID#	Cmpd Name
1	97.051	296257	14739	47.751				
2	97.841	296905	13448	47.856		V		
3	100.174	13455	848	2.169				
4	102.863	13802	948	2.225				
Total		620419	29983					

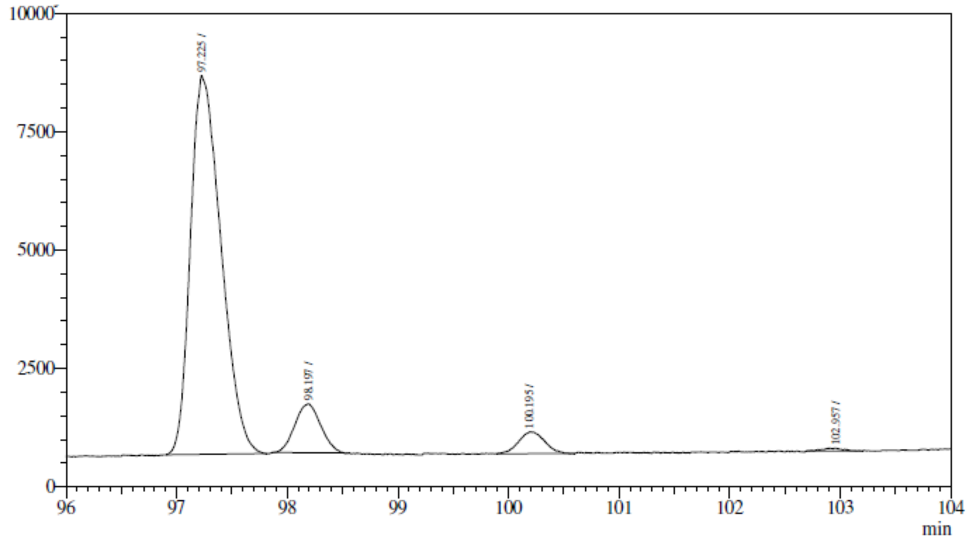

Peak#	Ret.Time	Area	Height	Conc.	Unit	Mark	ID#	Cmpd Name
1	97.225	153801	8003	86.009				
2	98.197	16810	1035	9.400				
3	100.195	7449	458	4.166				
4	102.957	760	62	0.425				
Total		178820	9558					

HPLC data for **11** (determined on intermediate PNP ester before addition of MeOH): Chiralpak AD-H, (*n*-hexane : *i*-PrOH 96:4, flow rate 1.5 mLmin^{-1}, 254 nm, 40 °C) t_R (1*R*,2*S*,5*S*): 26.8 min, t_R (1*S*,2*R*,5*R*): 30.9 min, 81:19 er.

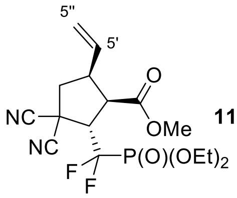

11

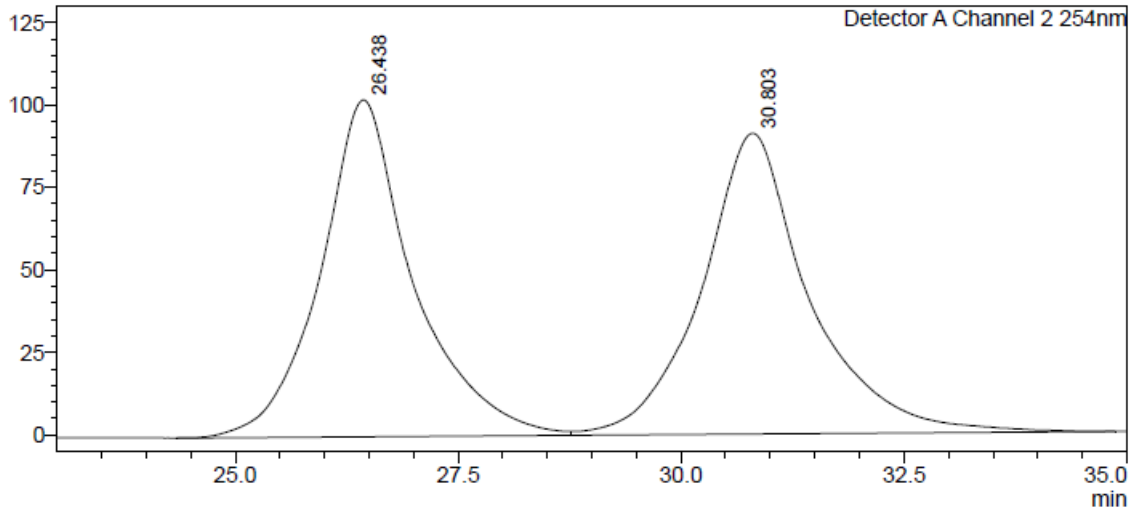

Detector A Channel 2 254nm

Peak#	Ret. Time	Area%
1	26.438	49.167
2	30.803	50.833
Total		100.000

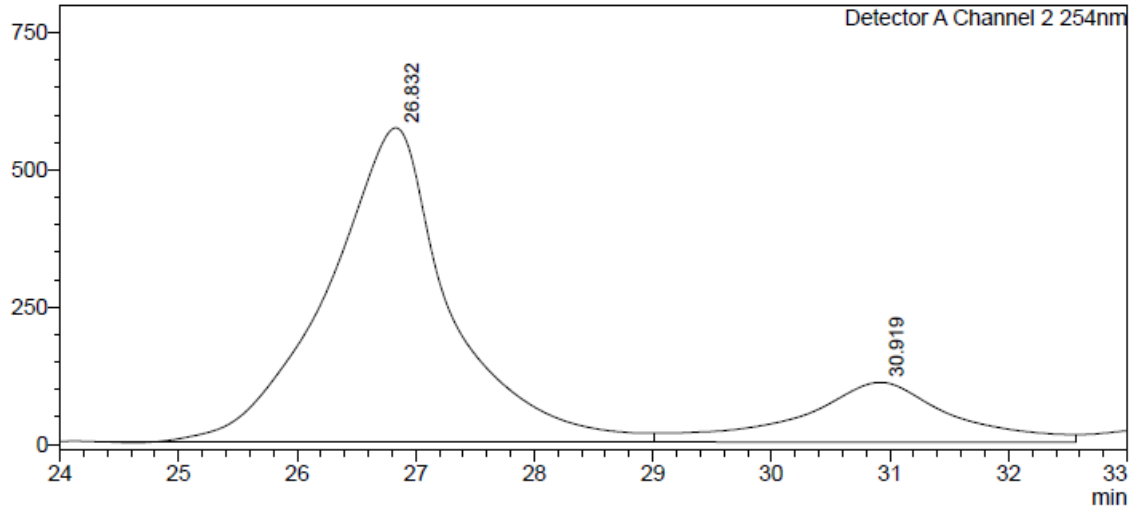

Detector A Channel 2 254nm

Peak#	Ret. Time	Area%
1	26.832	80.997
2	30.919	19.003
Total		100.000

HPLC Data for **12**: Chiralpak AD-H, (*n*-hexane : *i*-PrOH 95:5, flow rate 1.0 mLmin^{-1}, 254 nm, 40 °C) *major diastereoisomer*: t_R (1*S*,2*R*,5*R*): 27.9 min, t_R (1*R*,2*S*,5*S*): 36.8 min, 5:95 er. *minor diastereoisomer*: $t_{R,1}$: 27.9 min, $t_{R,2}$: 36.8 min, 25:75 er. (determined from crude reaction mixture as PNP ester)

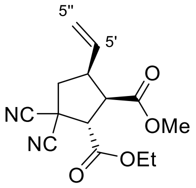

12

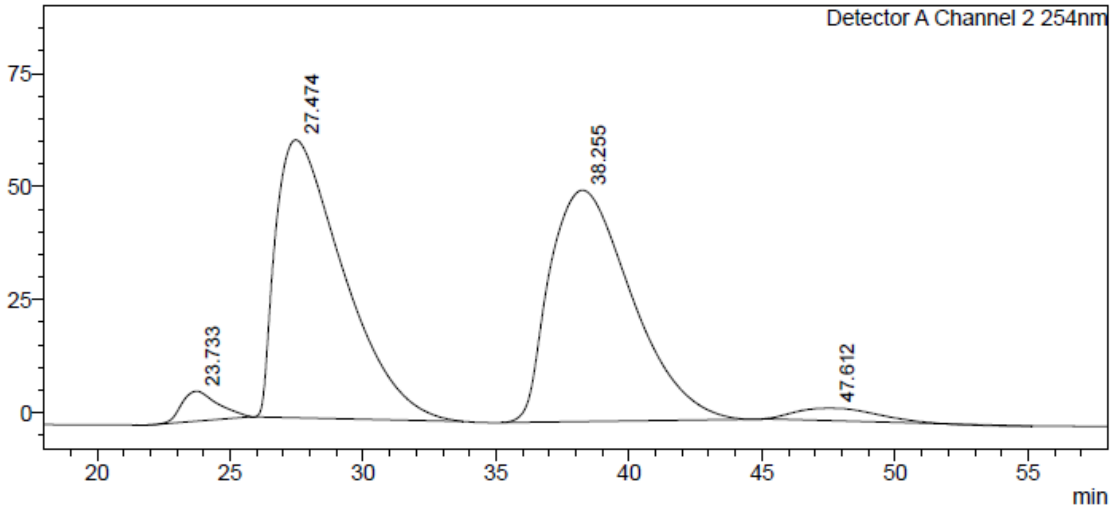

Detector A Channel 2 254nm		
Peak#	Ret. Time	Area%
1	23.733	2.688
2	27.474	47.281
3	38.255	47.605
4	47.612	2.426
Total		100.000

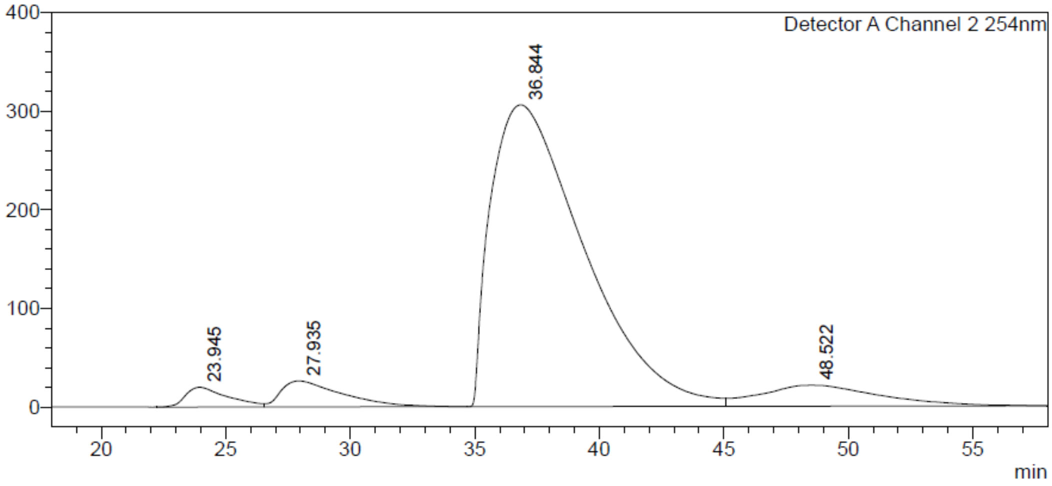

Detector A Channel 2 254nm		
Peak#	Ret. Time	Area%
1	23.945	2.523
2	27.935	4.795
3	36.844	85.081
4	48.522	7.601
Total		100.000

HPLC Data for **13**: Chiralcel OD-H, (*n*-hexane : *i*-PrOH 96:4, flow rate 1.0 mLmin^{-1}, 254 nm, 40 °C) t_R (1*S*,2*R*,5*R*): 17.4 min, t_R (1*R*,2*S*,5*S*): 34.1 min, 8:92 er. (determined from crude reaction mixture as PNP ester)

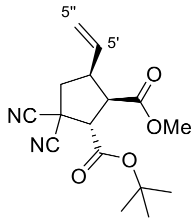

13

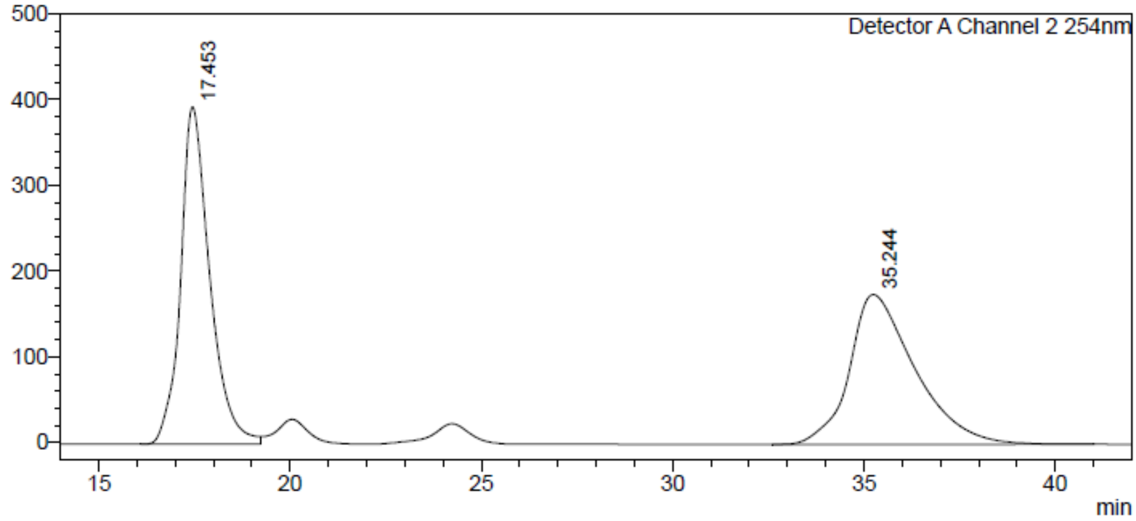

Detector A Channel 2 254nm

Peak#	Ret. Time	Area%
1	17.453	49.946
2	35.244	50.054
Total		100.000

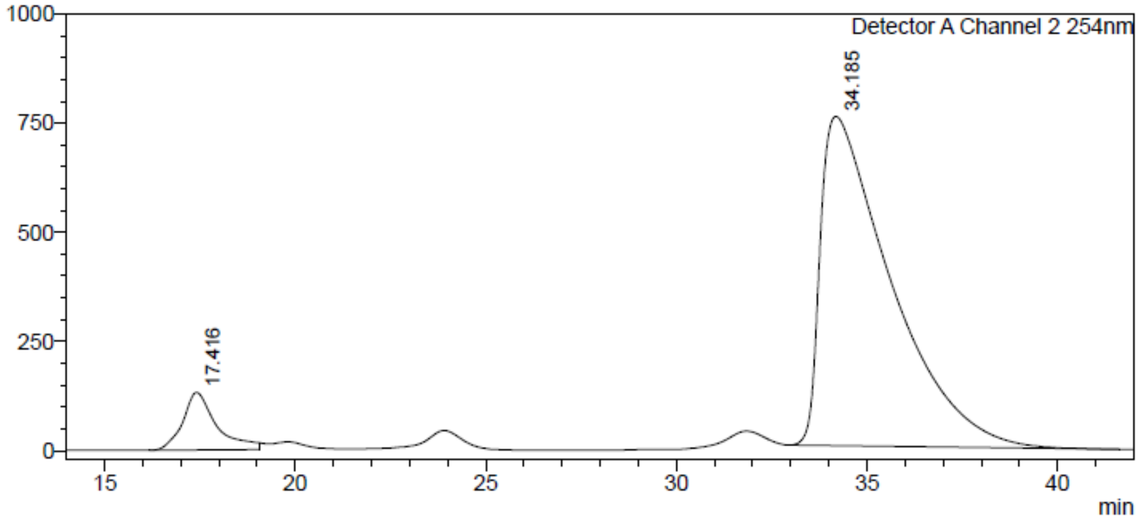

Detector A Channel 2 254nm

Peak#	Ret. Time	Area%
1	17.416	7.699
2	34.185	92.301
Total		100.000

HPLC Data for **14**: Chiralpak IA, (n-hexane : i-PrOH 95:5, flow rate 1.0 mLmin^{-1}, 211 nm, 30 °C) t_R (1*S*,2*R*,5*R*): 30.7 min, t_R (1*R*,2*S*,5*S*): 39.4 min, 9:91 er.

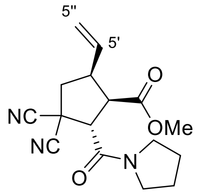

14

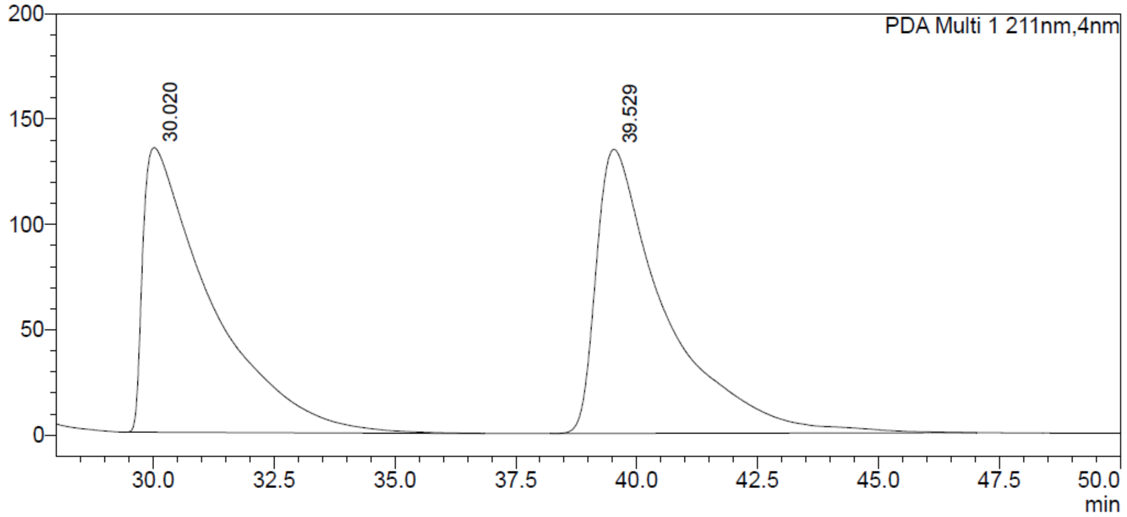

PDA Ch1 211nm

Peak#	Ret. Time	Area%
1	30.020	49.562
2	39.529	50.438
Total		100.000

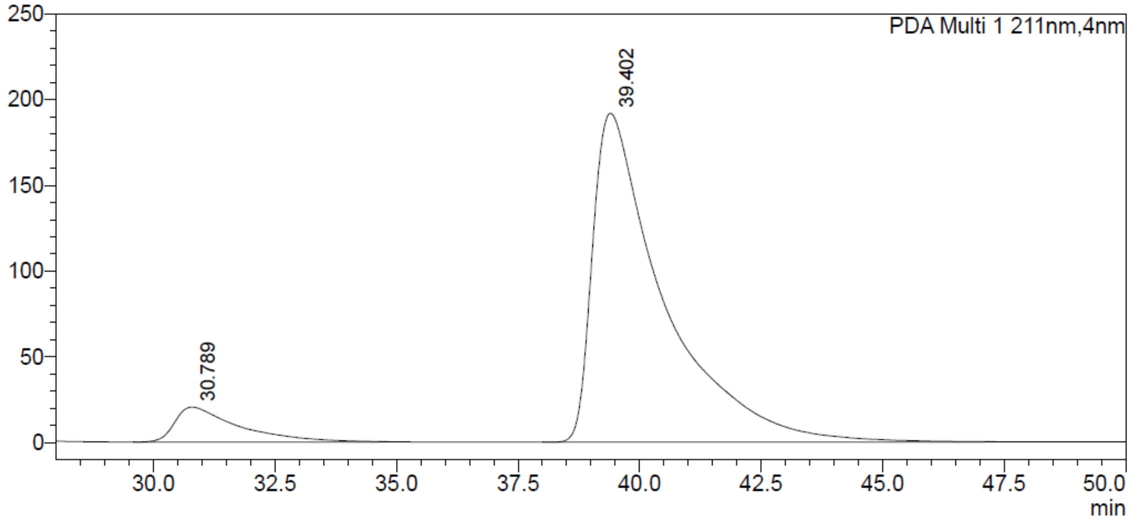

PDA Ch1 211nm

Peak#	Ret. Time	Area%
1	30.789	8.745
2	39.402	91.255
Total		100.000

HPLC data for **15**: Chiralpak AD-H, (*n*-hexane : *i*-PrOH 95:5, flow rate 1.0 mLmin^{-1}, 211 nm, 40 °C) t_R (1*S*,2*R*,5*R*): 15.5 min, t_R (1*R*,2*S*,5*S*): 18.9 min, 8:92 er.

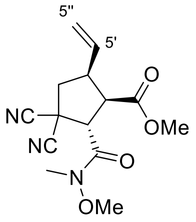

15

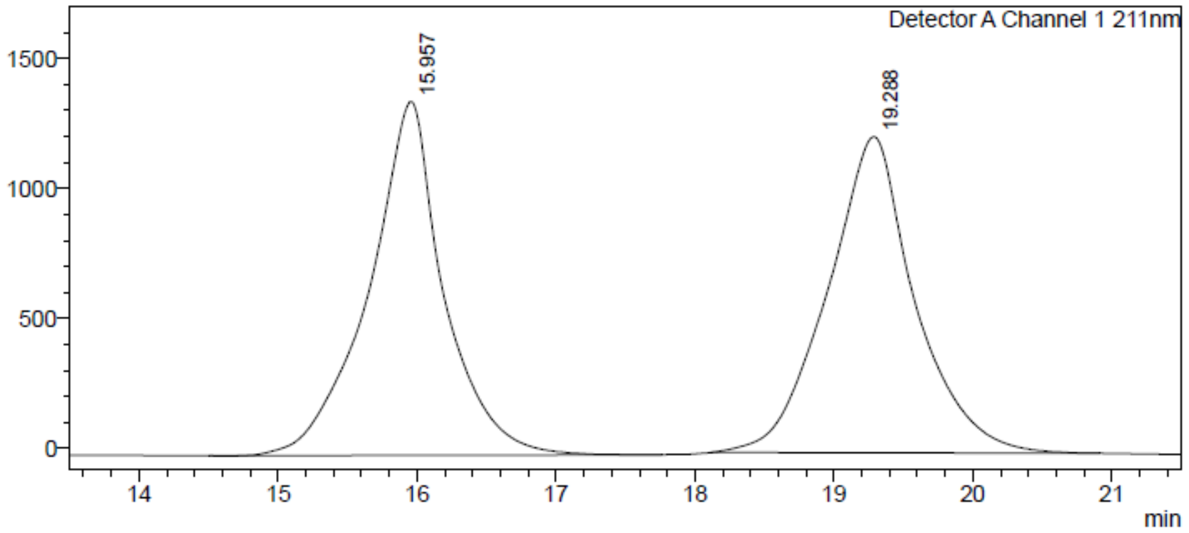

Peak#	Ret. Time	Area%
1	15.957	48.948
2	19.288	51.052
Total		100.000

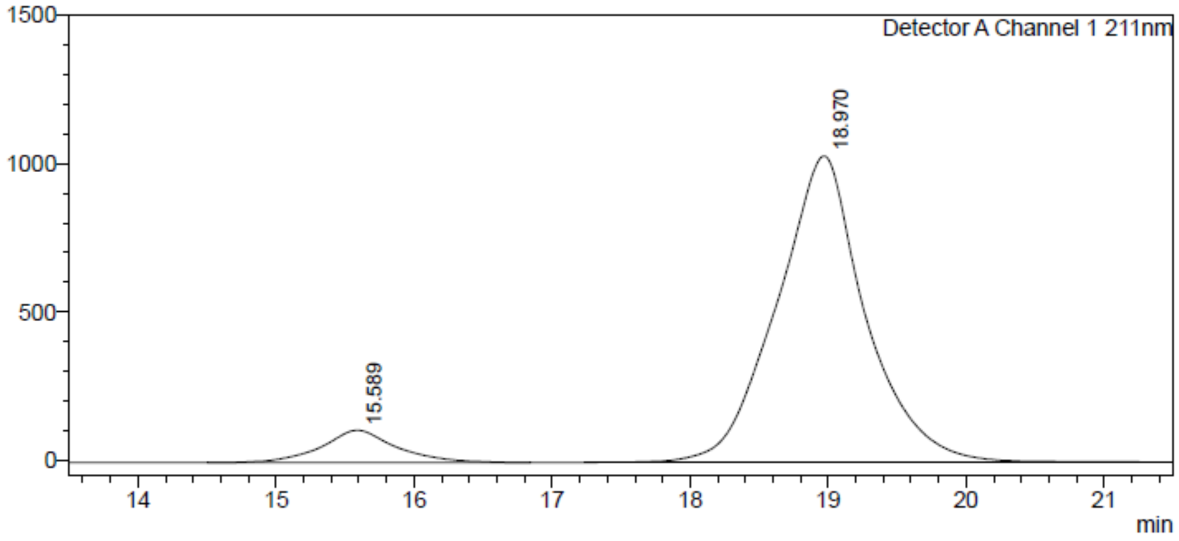

Peak#	Ret. Time	Area%
1	15.589	8.216
2	18.970	91.784
Total		100.000

HPLC data for **16**: Chiralpak IA, (*n*-hexane : *i*-PrOH 96:4, flow rate 1.0 mLmin^{-1}, 211 nm, 40 °C) t_R (1*S*,2*R*,5*R*): 17.8 min, t_R (1*R*,2*S*,5*S*): 25.9 min, 7:93 er.

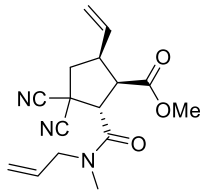

16

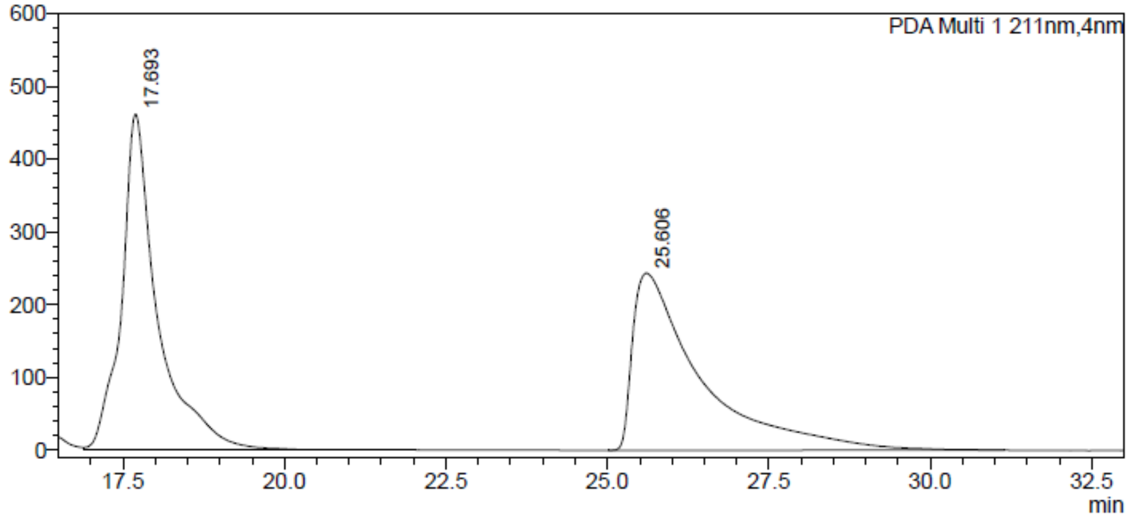

PDA Ch1 211nm

Peak#	Ret. Time	Area%
1	17.693	49.981
2	25.606	50.019
Total		100.000

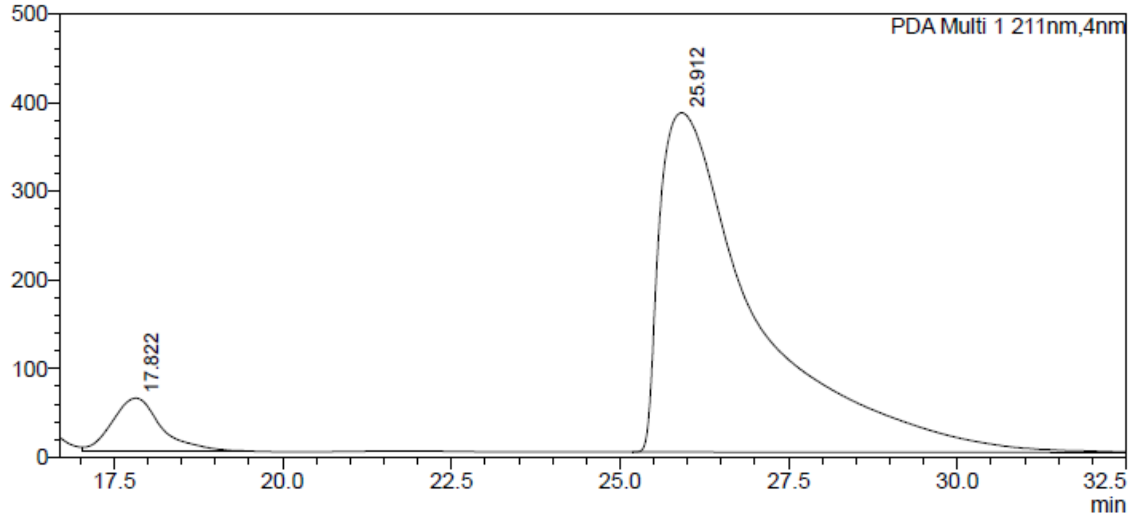

PDA Ch1 211nm

Peak#	Ret. Time	Area%
1	17.822	7.269
2	25.912	92.731
Total		100.000

GC data for **17**: Restek Rt-βDEXcst (length: 30 m, thickness: 0.25 mm, film thickness: 0.25 μm), carrier gas: He, linear velocity: 28 $cmsec^{-1}$, temperature: 60 to 220 °C (53 min)) t_R (1*R*,2*S*,5*S*): 38.3 min, t_R (1*S*,2*R*,5*R*): 38.7 min, 73:27 er.

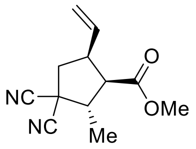

17

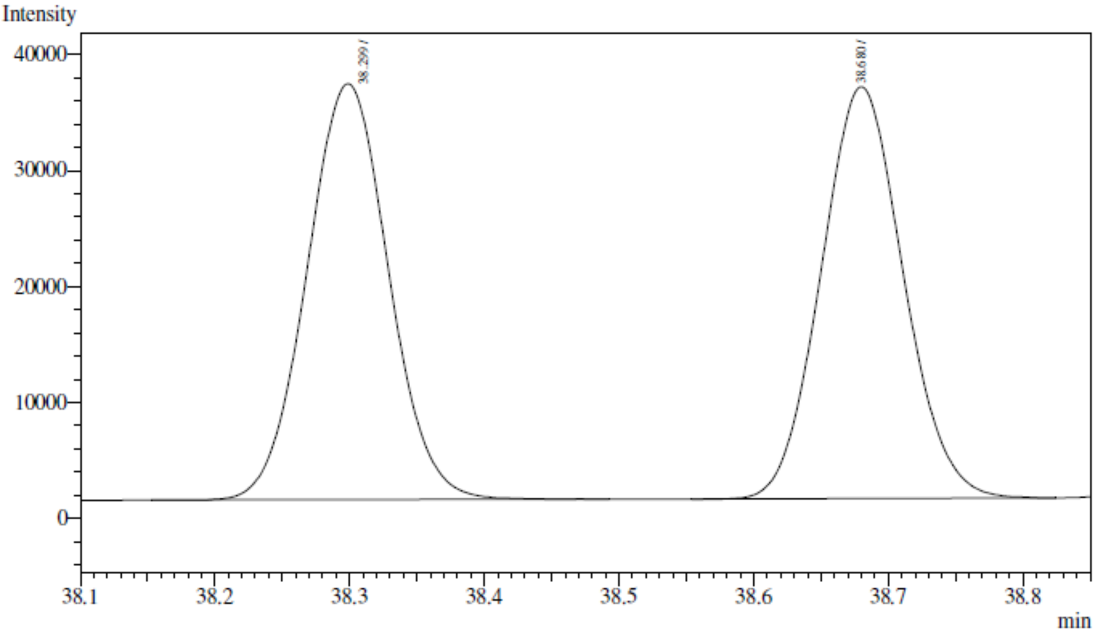

Peak#	Ret.Time	Area	Height	Conc.	Unit	Mark	ID#	Cmpd Name
1	38.299	151050	35812	50.068				
2	38.680	150641	35433	49.932				
**Total**		301691	71245					

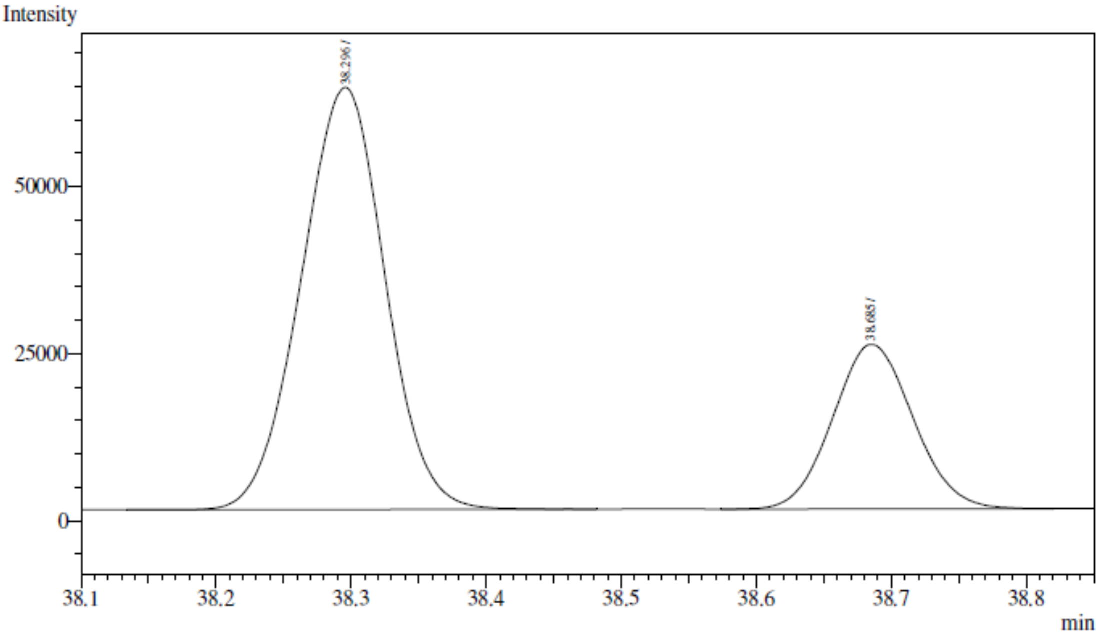

Peak#	Ret.Time	Area	Height	Conc.	Unit	Mark	ID#	Cmpd Name
1	38.296	273359	63202	72.751				
2	38.685	102389	24644	27.249				
**Total**		375748	87846					

$^{19}F\{^{1}H\}$ NMR data for **18**: (377 MHz, $CDCl_3$) δ_F (1*R*,2*S*,5*S*): −113.1, δ_F (1*S*,2*R*,5*R*): −113.3, 96:4 er.

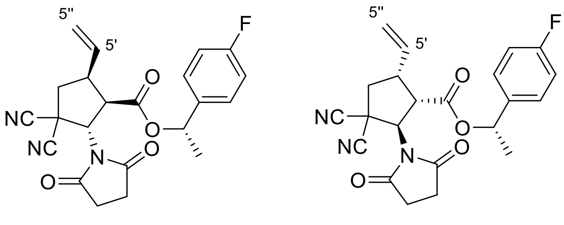

(1*R*,2*S*,5*S*)-**S49** (1*S*,2*R*,5*R*)-**S50**

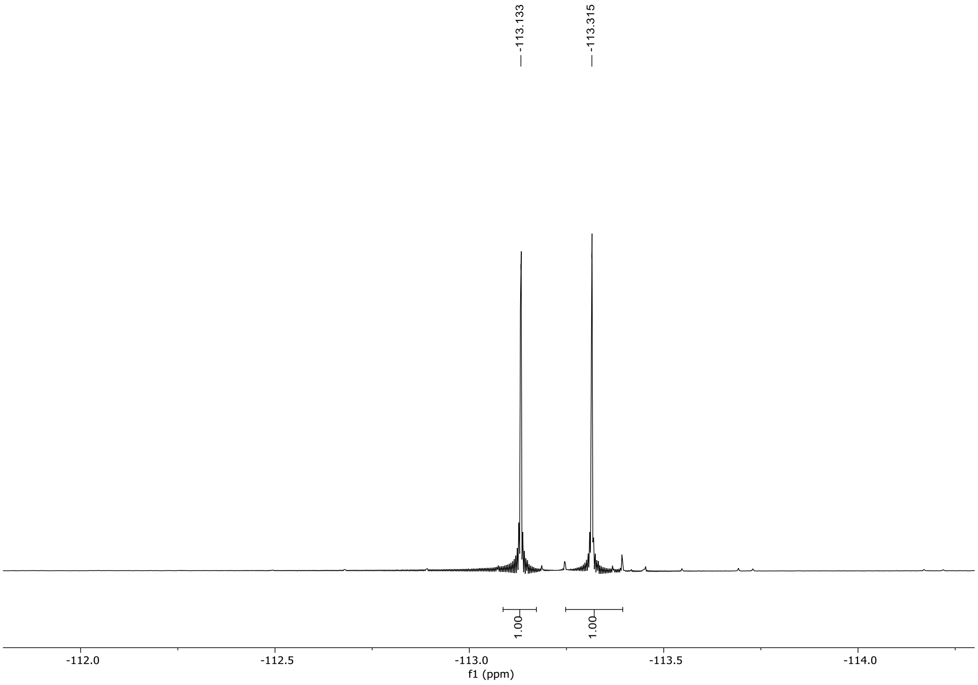

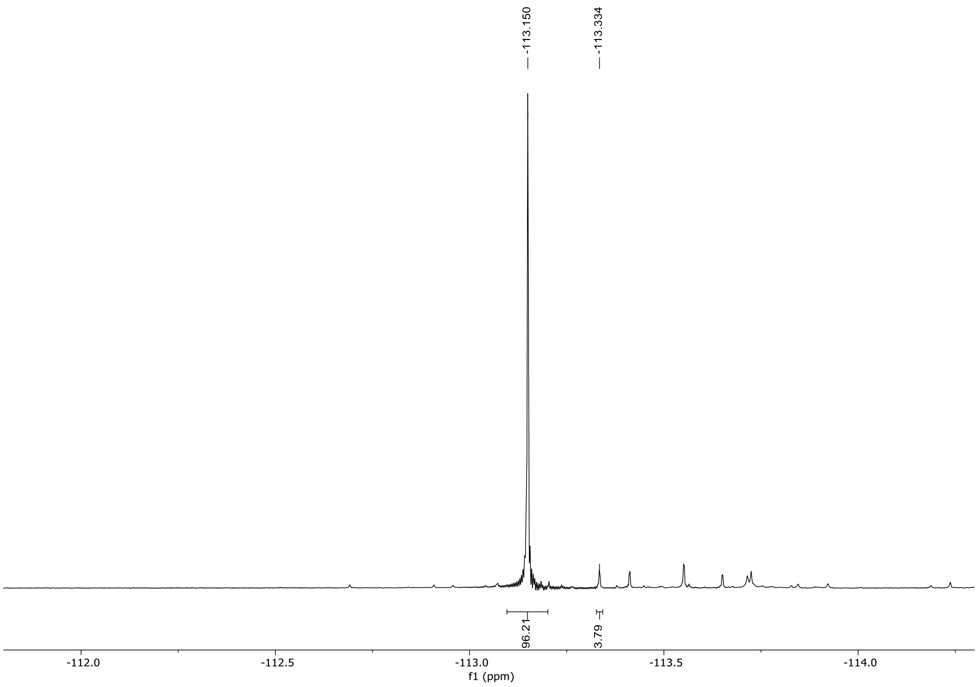

HPLC data for **19**$_{\mathbf{maj}}$**:** Chiralpak AD-H, (*n*-hexane : *i*-PrOH 99:1, flow rate 1.0 mLmin^{-1}, 211 nm, 30 °C) t_R (2*S*,3*R*,4*S*): 26.3 min, Chiralpak AD-H, (*n*-hexane : *i*-PrOH 94:6, flow rate 1.0 mLmin^{-1}, 211 nm, 30 °C) t_R (2*R*,3*S*,4*R*): 14.4 min, 89:11 er. HPLC data for **19**$_{\mathbf{min}}$**:** **C**hiralpak AD-H, (*n*-hexane : *i*-PrOH 99:1, flow rate 1.0 mLmin^{-1}, 211 nm, 30 °C) t_R (2*S*,3*R*,4*R*): 28.4 min, Chiralpak AD-H, (*n*-hexane : *i*-PrOH 94:6, flow rate 1.0 mLmin^{-1}, 211 nm, 30 °C) t_R (2*R*,3*S*,4*S*): 15.5 min, 98:2 er.

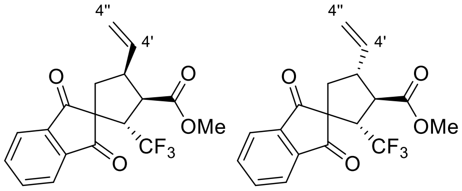

19$_{\mathbf{maj}}$ **19**$_{\mathbf{min}}$

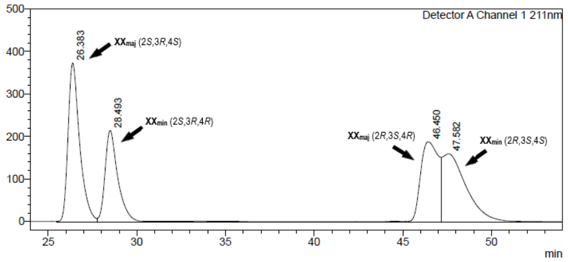

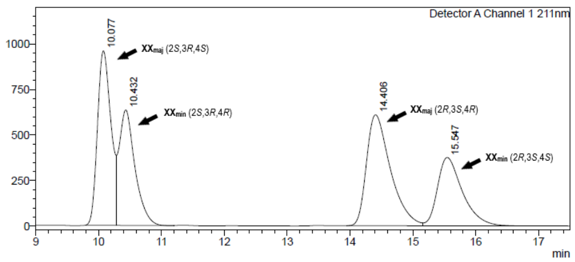

Detector A Channel 1 211nm

Peak#	Ret. Time	Area%
1	26.383	30.181
2	28.493	19.863
3	46.450	23.134
4	47.582	26.823
Total		100.000

Detector A Channel 1 211nm

Peak#	Ret. Time	Area%
1	10.077	27.692
2	10.432	21.248
3	14.406	31.072
4	15.547	19.988
Total		100.000

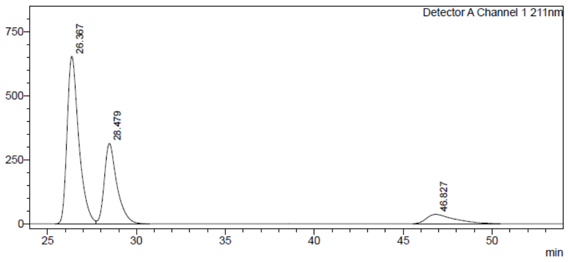

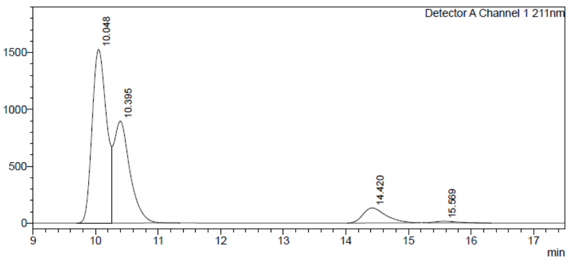

Detector A Channel 1 211nm

Peak#	Ret. Time	Area%
1	26.367	60.788
2	28.479	31.292
3	46.827	7.921
Total		100.000

Detector A Channel 1 211nm

Peak#	Ret. Time	Area%
1	10.048	55.249
2	10.395	36.370
3	14.420	7.553
4	15.569	0.828
Total		100.000

Calculation of er for **19**$_{\mathbf{maj}}$: major enantiomer = 60.788 Area%; minor enantiomer = 7.129 Area% (calculated from Area% at 6% *i*-PrOH and applied to area at 1% *i*-PrOH); 60.788 Area% : 7.129 Area% = 89:11 er.

Calculation of er for **19**$_{\mathbf{min}}$: major enantiomer = 31.292 Area%; minor enantiomer = 0.792 Area% (calculated from Area% at 6% *i*-PrOH and applied to area at 1% *i*-PrOH); 31.292 Area% : 0.792 Area% = 98:2 er.

HPLC data for **20B$_{maj}$**: Chiralpak AD-H, (*n*-hexane : *i*-PrOH 95:5, flow rate 1.0 mLmin^{-1}, 254 nm, 40 °C) t_R (2*S*,3*R*,4*S*): 15.5 min, t_R (2*R*,3*S*,4*R*): 25.7 min, 97:3 er. (determined from the crude reaction mixture as PNP ester) The enantiomeric ratio for **20A** could not be determined.

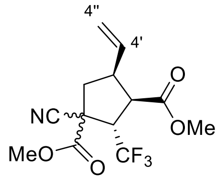

20

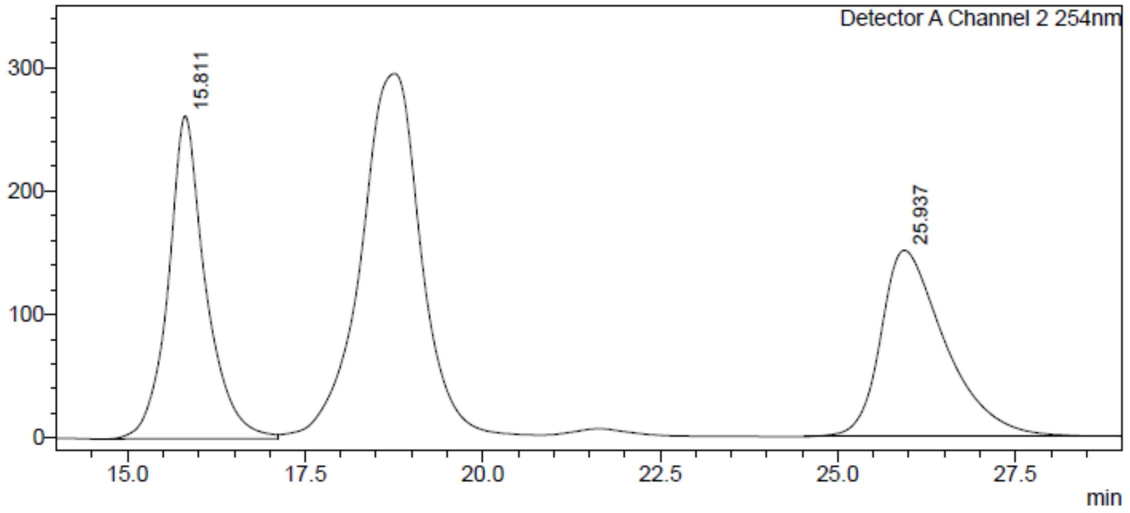

Detector A Channel 2 254nm

Peak#	Ret. Time	Area%
1	15.811	49.970
2	25.937	50.030
Total		100.000

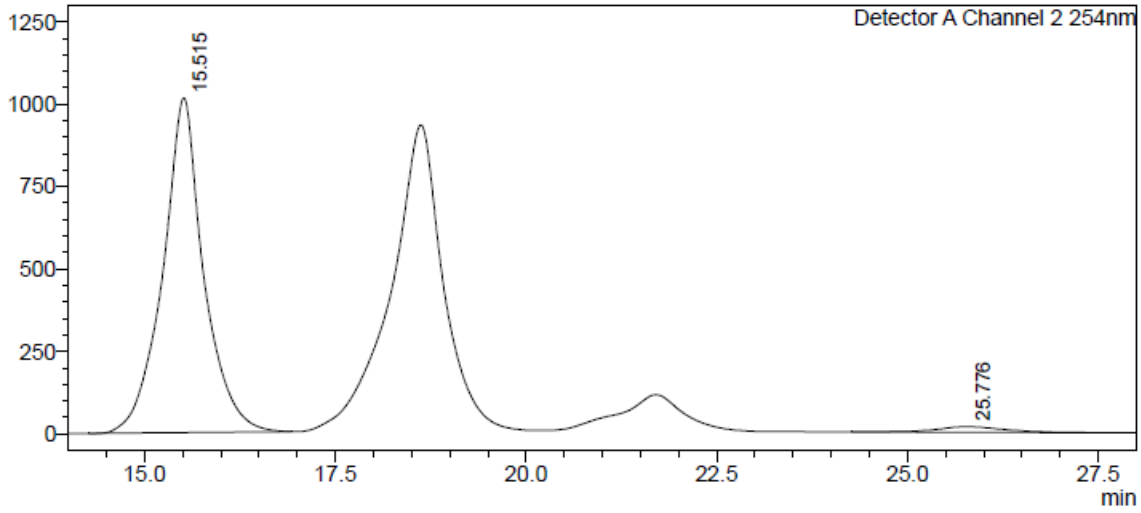

Detector A Channel 2 254nm

Peak#	Ret. Time	Area%
1	15.515	97.264
2	25.776	2.736
Total		100.000

GC data for **21:** Restek Rt-βDEXcst (length: 30 m, thickness: 0.25 mm, film thickness: 0.25 μm, carrier gas: He, linear velocity: 28 cmsec^{-1}, temperature: 110 °C (95 min)) *major diastereoisomer*: t_R (2*R*,3*S*,4*R*): 85.4 min, t_R (2*S*,3*R*,4*S*): 88.3 min, 3:97 er. *minor diastereoisomer*: t_R (2*R*,3*S*,4*S*): 79.2 min, t_R (2*S*,3*R*,4*R*): 81.9 min, 3:97 er.

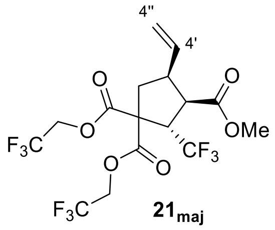

21$_{maj}$

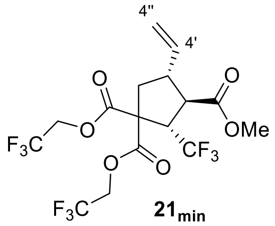

21$_{min}$

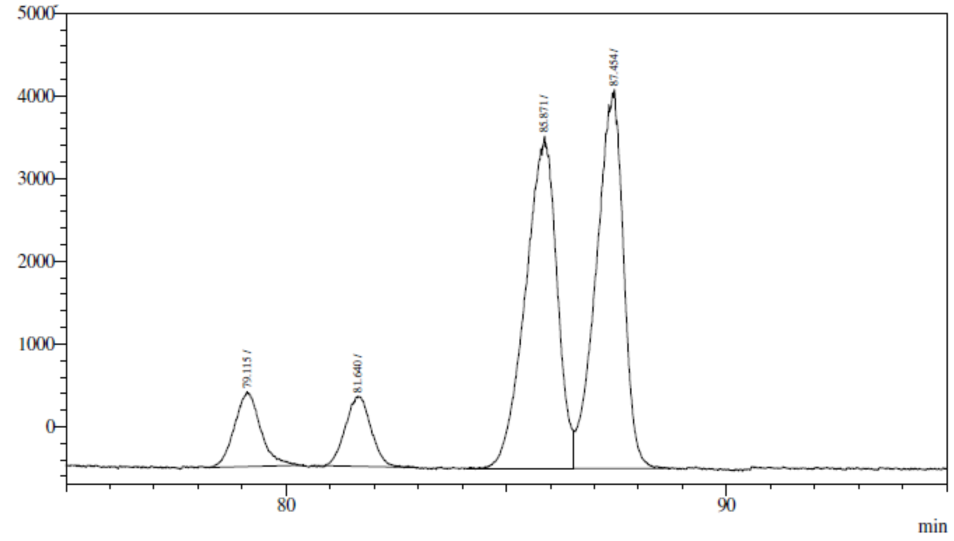

Peak#	Ret.Time	Area	Height	Conc.	Unit	Mark	ID#	Cmpd Name
1	79.115	37129	906	7.912				
2	81.640	34345	851	7.319				
3	85.871	200011	4017	42.623				
4	87.454	197775	4584	42.146		V		
**Total**		469260	10358					

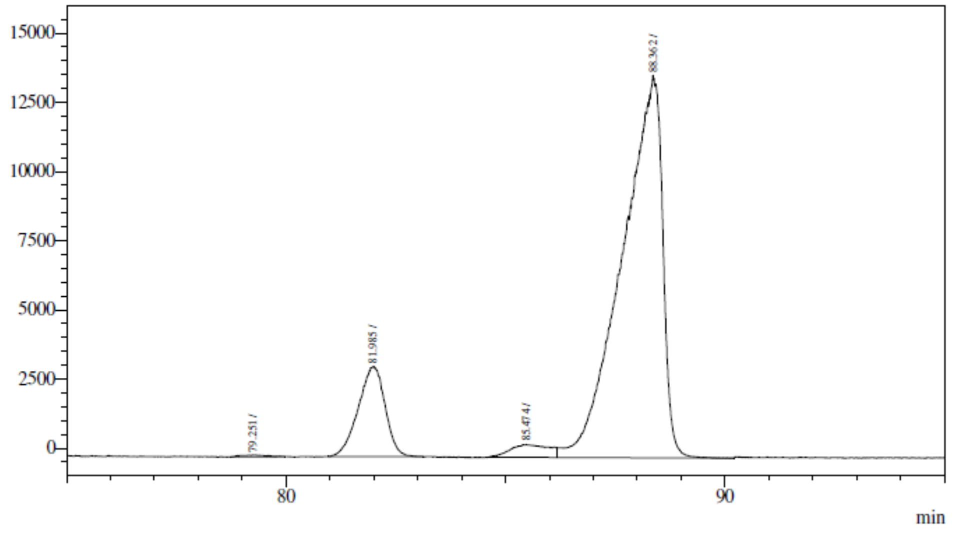

Peak#	Ret.Time	Area	Height	Conc.	Unit	Mark	ID#	Cmpd Name
1	79.251	2538	67	0.242				
2	81.985	138416	3257	13.173				
3	85.474	28733	465	2.735				
4	88.362	881069	13831	83.851		V		
**Total**		1050756	17620					

HPLC data for **26:** Chiralpak AD-H, (*n*-hexane : *i*-PrOH 93:7, flow rate 1.0 mLmin^{-1}, 254 nm, 40 °C) t$_R$ (1*R*,2*S*,5*S*): 10.4 min, t$_R$ (1*S*,2*R*,5*R*): 22.7 min, 93:7 er.

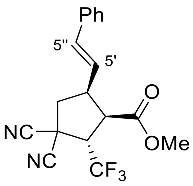

26

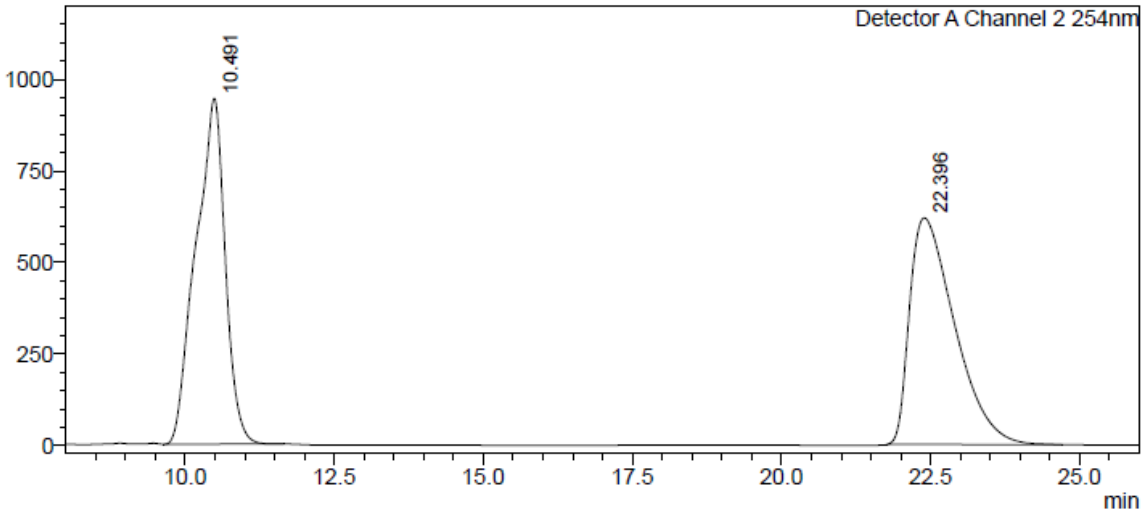

Detector A Channel 2 254nm

Peak#	Ret. Time	Area%
1	10.491	49.896
2	22.396	50.104
Total		100.000

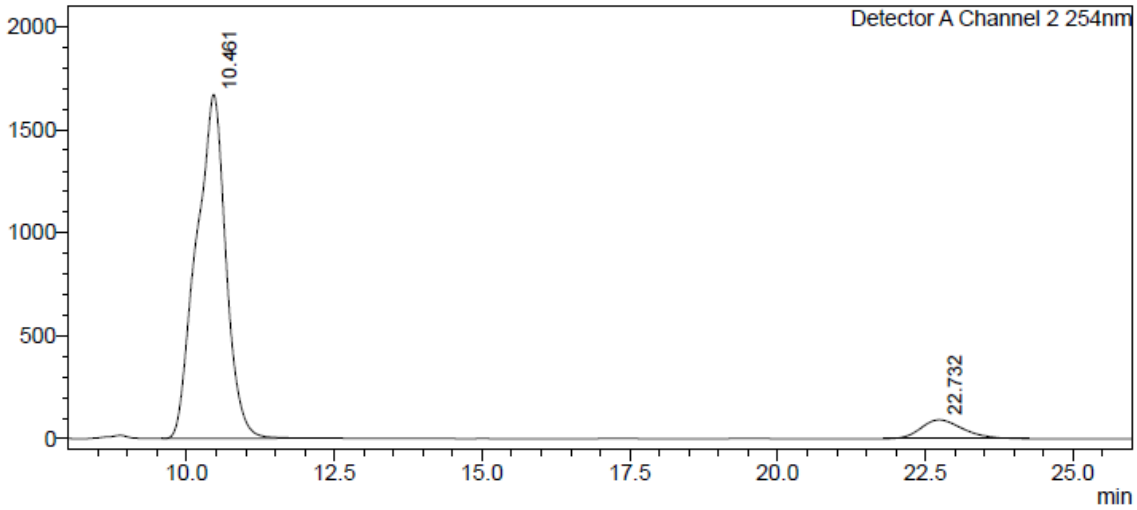

Detector A Channel 2 254nm

Peak#	Ret. Time	Area%
1	10.461	92.775
2	22.732	7.225
Total		100.000

GC data for **S52:** Restek Rt-βDEXcst (length: 30 m, thickness: 0.25 mm, film thickness: 0.25 μm, carrier gas: He, linear velocity: 28 cmsec^{-1}, temperature: 60 to 190 °C (43 min), 190 °C (15 min)) t_R (2*S*,3*R*,4*S*): 51.1 min, t_R (2*R*,3*S*,4*R*): 51.6 min, 93:7 er.

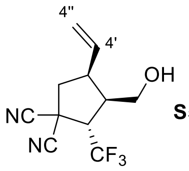

S52

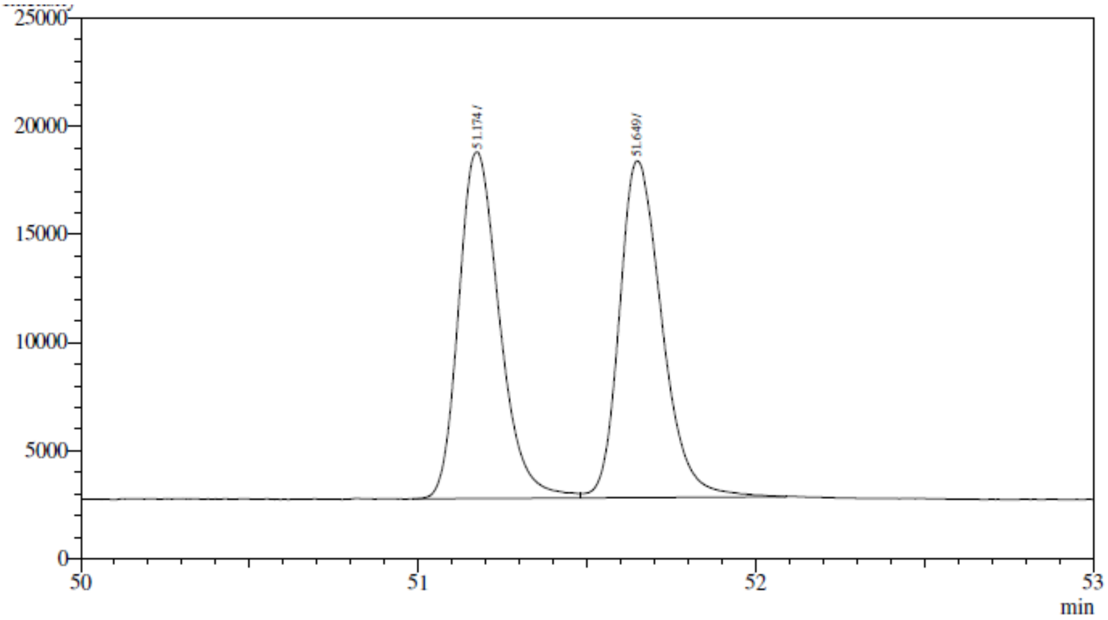

Peak#	Ret.Time	Area	Height	Conc.	Unit	Mark	ID#	Cmpd Name
1	51.174	134834	15999	49.729				
2	51.649	136303	15552	50.271		V		
Total		271137	31551					

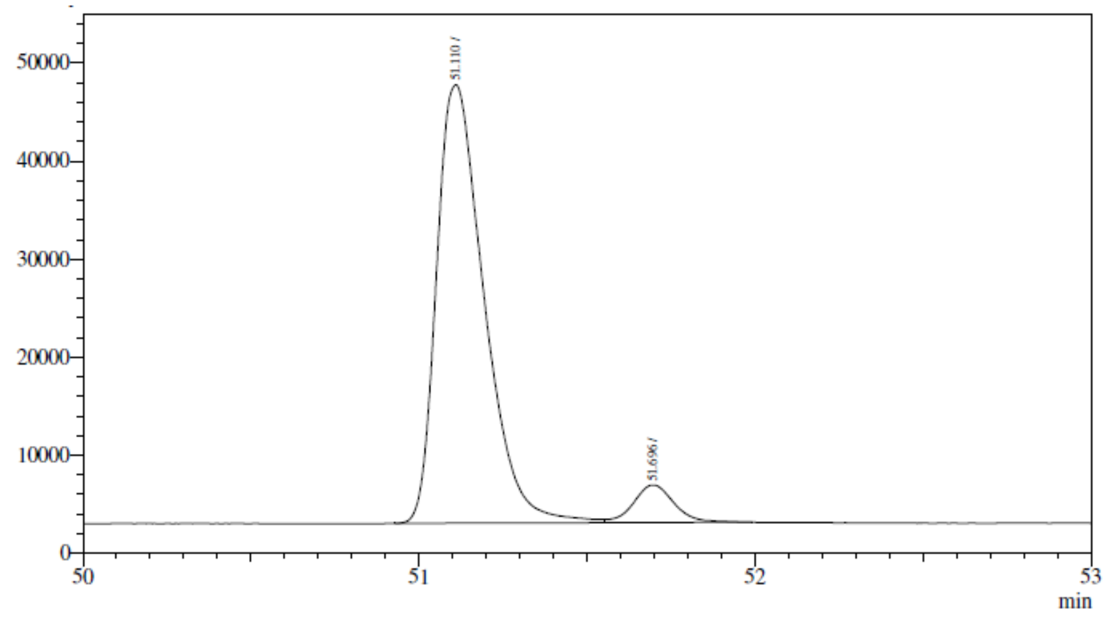

Peak#	Ret.Time	Area	Height	Conc.	Unit	Mark	ID#	Cmpd Name
1	51.110	439397	44681	93.193				
2	51.696	32096	3837	6.807		V		
Total		471493	48518					

HPLC data for **S53:** Chiralpak IA, (*n*-hexane : *i*-PrOH 97:3, flow rate 1.0 mLmin^{-1}, 211 nm, 40 °C) t_R (1*R*,2*S*,5*S*): 13.8 min, t_R (1*S*,2*R*,5*R*): 23.3 min, 92:8 er.

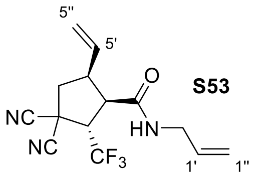

S53

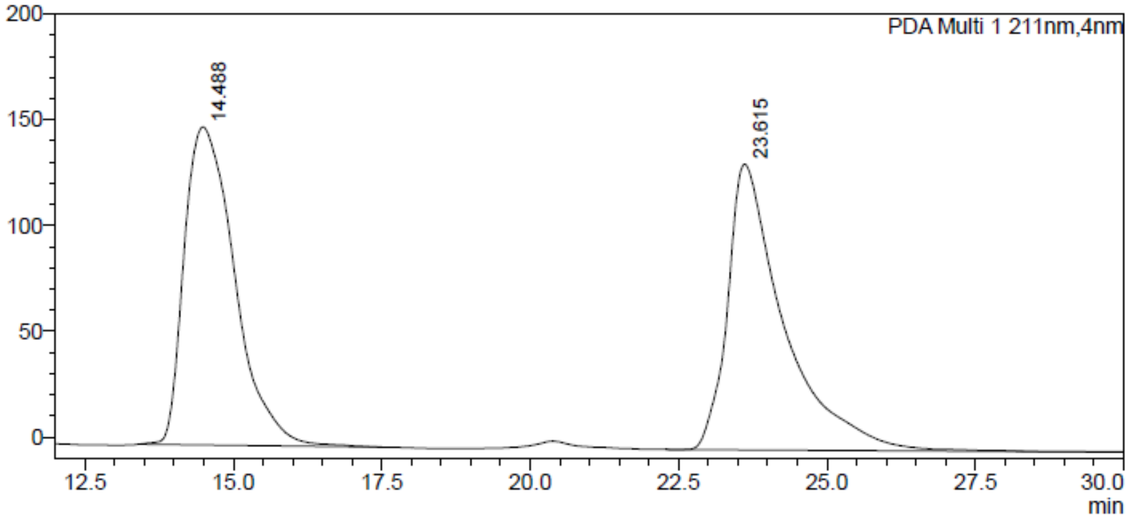

PDA Ch1 211nm

Peak#	Ret. Time	Area%
1	14.488	49.972
2	23.615	50.028
Total		100.000

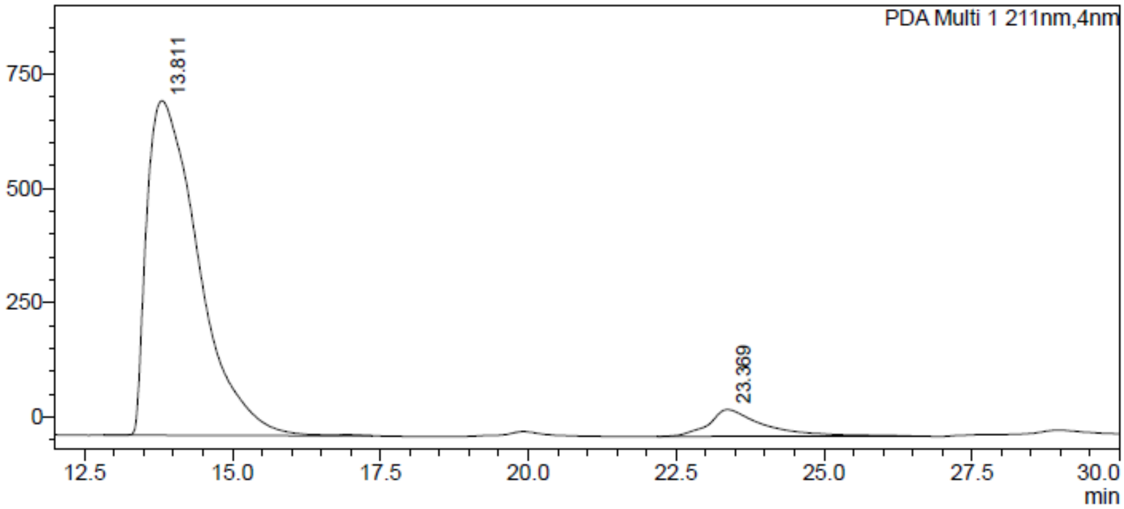

PDA Ch1 211nm

Peak#	Ret. Time	Area%
1	13.811	92.183
2	23.369	7.817
Total		100.000

HPLC data for **28**: Chiralpak AD-H (hexane : *i*-PrOH 92:8, flow rate 1.0 mlmin^{-1}, 211 nm, 40 °C) t_R (5a*S*,8*S*,8a*R*): 14.3 min, t_R (5a*R*,8*R*,8a*S*): 23.2 min, 94:6 er.

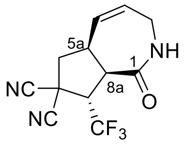

28

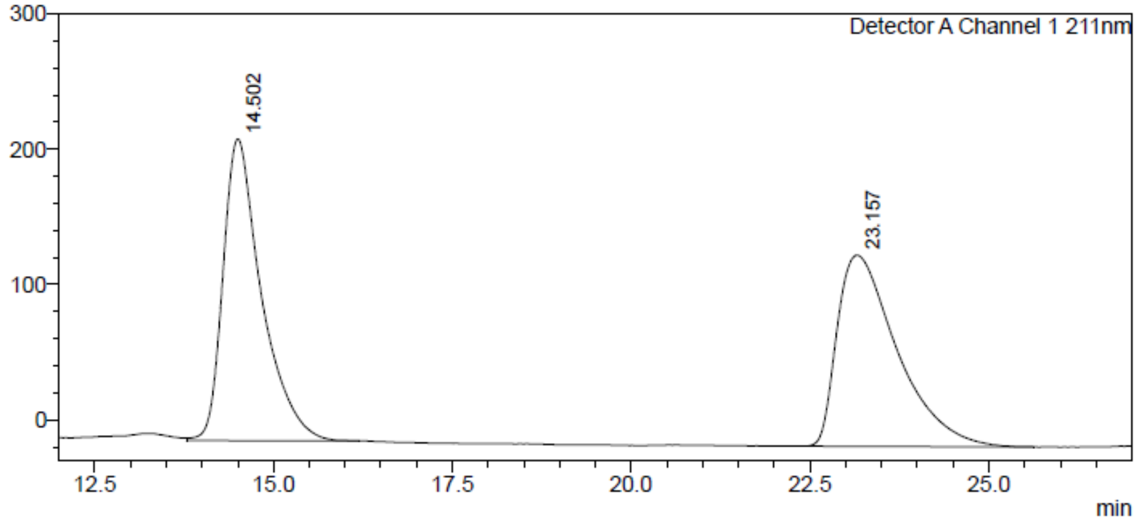

Detector A Channel 1 211nm

Peak#	Ret. Time	Area%
1	14.502	50.198
2	23.157	49.802
Total		100.000

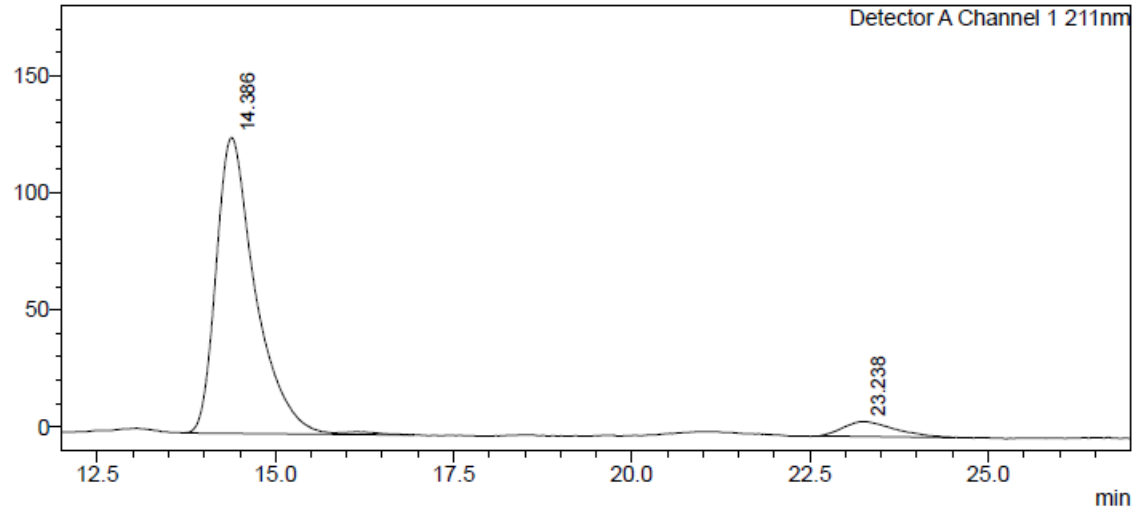

Detector A Channel 1 211nm

Peak#	Ret. Time	Area%
1	14.386	93.929
2	23.238	6.071
Total		100.000
